# Analysis of H3K4me3-ChIP-Seq and RNA-Seq data to understand the putative role of miRNAs and their target genes in breast cancer cell lines

**DOI:** 10.5808/gi.21020

**Published:** 2021-06-30

**Authors:** Aneesh Kotipalli, Ruma Banerjee, Sunitha Manjari Kasibhatla, Rajendra Joshi

**Affiliations:** HPC-Medical and Bioinformatics Applications Group, Centre for Development of Advanced Computing, Pune 411008, India

**Keywords:** breast neoplasms, ChIP-Seq, luminal-A/triple-negative, miRNA, RNA-Seq

## Abstract

Breast cancer is one of the leading causes of cancer in women all over the world and accounts for ~25% of newly observed cancers in women. Epigenetic modifications influence differential expression of genes through non-coding RNA and play a crucial role in cancer regulation. In the present study, epigenetic regulation of gene expression by *in-silico* analysis of histone modifications using chromatin immunoprecipitation sequencing (ChIP-Seq) has been carried out. Histone modification data of H3K4me3 from one normal-like and four breast cancer cell lines were used to predict miRNA expression at the promoter level. Predicted miRNA promoters (based on ChIP-Seq) were used as a probe to identify gene targets. Five triple-negative breast cancer (TNBC)‒specific miRNAs (miR153-1, miR4767, miR4487, miR6720, and miR-LET7I) were identified and corresponding 13 gene targets were predicted. Eight miRNA promoter peaks were predicted to be differentially expressed in at least three breast cancer cell lines (miR4512, miR6791, miR330, miR3180-3, miR6080, miR5787, miR6733, and miR3613). A total of 44 gene targets were identified based on the 3′-untranslated regions of downregulated mRNA genes that contain putative binding targets to these eight miRNAs. These include 17 and 15 genes in luminal-A type and TNBC respectively, that have been reported to be associated with breast cancer regulation. Of the remaining 12 genes, seven (*A4GALT, C2ORF74, HRCT1, ZC4H2, ZNF512, ZNF655*, and *ZNF608*) show similar relative expression profiles in large patient samples and other breast cancer cell lines thereby giving insight into predicted role of H3K4me3 mediated gene regulation via the miRNA-mRNA axis.

## Introduction

Breast cancer is one of the leading causes of death in women all over the world [1]. There are many subtypes in breast cancer identified based on the origin, hormone receptors expression, and response to treatment. There are four basic subtypes namely luminal-A, luminal-B, human epidermal growth factor receptor-2 (HER2) and triple-negative breast cancer (TNBC) [2]. Among all the subtypes, the TNBC is very aggressive and has poor prognosis compared to other subtypes and very few systemic treatment options are available other than chemotherapy [3]. Luminal-A (estrogen receptor [ER]+ and/or progesterone receptor [PR]+, HER2−) because of ER expression, has better prognosis compared to TNBC [2]. Epigenetic regulation via histone modification of microRNA (miRNA) promoters is known to play a crucial role in breast cancer regulation [4]. Histone modifications wrapped around genes play an important role in gene regulation by providing access to transcription factors, RNA-polymerases, and other regulatory mechanisms [5]. There are several histone post-translational modifications identified earlier with a unique regulatory function for each of them. Using chromatin immunoprecipitation followed by sequencing (ChIP-Seq), one can identify the position of targeted protein binding (transcription factors, histone modifications) regions in the genome [6].

Epigenetic gene regulation occurs in three major ways namely DNA methylation of CpG islands, histone modifications, and non-coding RNA mediated [7]. Each regulatory level has a crucial role in normal cell development and diseases such as cancer and other non-communicable diseases. Gene level histone modifications can be used to predict the status of a gene whether it is active or inactive. H3K4me3 modification at the promoter level provides information about active genes, whereas H3K27me3 and H3K9me3 modifications provide the inactive status of the gene [8]. Previous studies provided evidence for correlation of histone modifications (H3K36me3, H3K9ac, H3K27ac, and H3K4me1) with gene expression. Combinations of two or more histone modifications at gene promoter and gene body level provides more resolution to predict gene activity [8].

Non-coding (nc) RNAs (LncRNAs, long intervening/intergenic noncoding RNA, miRNA, and small interfering RNA) play a major role in gene regulation of different biological processes such as cell cycle and proliferation along with developmental and metabolic processes [9]. The most widely studied ncRNAs are miRNAs which are small ncRNA ~22nt in length, evolutionarily conserved, and have a wide regulatory role in development and diseases. They play an important role in gene regulation by targeting complementary binding sites in untranslated region (UTR) regions of gene transcripts or by targeting promoters or other miRNA or LncRNA [10]. Interestingly, miRNAs are also involved in the upregulation of specific genes via binding to their promoters in the nucleus and thereby controlling gene expression [11]. An actively transcribed miRNA is able to regulate ~100‒1,000 genes by complementary binding to their targeted genes. Based on conserved base-pairing homology, it has been predicted that ~60% of human genes are targeted by miRNAs [12]. In many cancers dysregulation of miRNA causes cancer progression and drug resistance. The role of miRNA deregulation in breast cancer was first reported in 2005 wherein the role of miR548 as an oncogenic regulator in breast cancer was elaborated [13]. There are other miRNAs such as let-7, miR145, miR200, and miR497 with a definitive role in breast cancer [14].

The present study is limited to normal-like, luminal-A and TNBC-claudin subtypes of breast cancer based on hormonal receptor expression. One each of normal subtype, ER positive subtype (Luminal-A) and ER negative (TNBC-claudin) were chosen for analysis. In the current study, ChIP-Seq data corresponding to histone modification H3K4me3 for one normal-like (MCF10A) and four breast cancer cell lines (luminal-A [MCF7, ZR751], TNBC [MB231, MB436]) were chosen to understand role of miRNA-gene promoter regulation of miRNA-mRNA axis. An attempt has been made to map the epigenetic expression patterns (histone H3K4me3) with RNA-sequencing (RNA-Seq) expression of miRNA targeted genes. Analysis of such combined data promises to provide insights into understanding epigenetic gene regulation (chromatin) as well as gene expression [15].

## Methods

ChIP-Seq data were downloaded from Gene Expression Omnibus (GEO) for breast cancer pertaining to six cell lines, viz., normal-like (MCF10A and 76NF2V), luminal-A subtype (MCF7 and ZR751), and TNBC subtype (MB231 and MB436), each with one activation histone modification H3K4me3 and two replicates (sequenced as Illumina single-end reads) (Supplementary Tables 1, 2) [16]. RNA-Seq data for the above-mentioned cell lines with four replicates were also downloaded from GEO (Supplementary Table 3) [16].

**Fig. 1.**
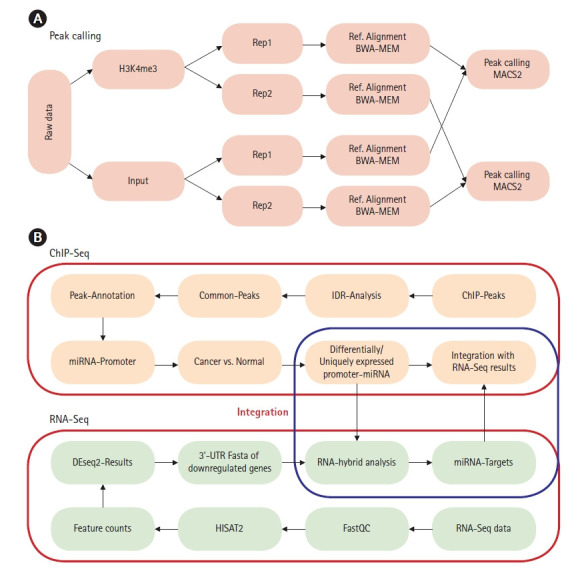
(A) Chromatin immunoprecipitation sequencing (ChIP-Seq) peak calling workflow for miRNA promoter prediction. (B) ChIP-Seq and RNA sequencing (RNA-Seq) data integration workflow for prediction of miRNA-mRNA interaction via 3′-untranslated region (3′-UTR) binding target prediction.

The raw reads were checked for quality using FastQC (version 0.11.7) [17]. BWA-MEM (Burrows-Wheeler alignment-Maximal Exact Matches) version 0.7.17 was used for alignment with reference genome build hg38 [18]. Samtools (version 1.8) was used to manage replicates and for sam to bam conversion [19]. ChIP-Seq analysis was done using model-based analysis of ChIP-Seq (MACS2, version 2.1.1.20160309) [20]. Peak calling was done using narrowpeak as H3K4me3 generates narrow histone marks [21]. p-value thresholds for peak calling were set to 0.001 for all samples and all replicates (Fig. 1A) [22]. Raw ChIP-Seq data of both H3K4me3 and corresponding input sequence was used for peak calling. To identify the reproducibility within the biological replicates IDR2.0.3 tool was used with a threshold of 0.05 to obtain statistically significant peaks [23,24]. Pseudo replicate analysis was carried out to identify low reproducible replicates which satisfy the criteria of N1/N2 ≥ 2 and Np/Nt ≥ 2 (where N1 represents the number of replicate1 self-consistent peaks, and N2 represents the number of replicate two self-consistent peaks; Np represents the number of peaks consistent between pooled pseudoreplicates, and, Nt represents the number of peaks consistent between true replicates) [24]. Peaks were annotated using HOMER (v3.12) tool [25]. Peaks corresponding to miRNA promoter genes were extracted using *in-house* scripts. Cis-regulatory Element Annotation System (CEAS -0.9.9.7) was used to get statistics on ChIP enrichment for genomic features such as chromosomes, promoters, gene bodies, or exons, to infer genes that are most likely to be regulated by a binding factor [26]. For RNA-Seq analysis HISAT2 (v2.1.0) was used for alignment of raw reads [27] and feature counts (v1.5.0p1) tool was used to count reads mapped on to each gene [28]. DEseq2 (v1.24) was used for differential gene expression of subtypes [29]. Downregulated and upregulated genes were selected based on log2fold change > 2 as per guidelines for analysis of multi-omics data [30]. Differential expression of genes for normal-like (MCF10A) vs. luminal-A (MCF7 and ZR751) and normal-like (MCF10A) vs. TNBC (MB231 and MB436) was carried out (Supplementary Fig. 1). miRNA sequences were extracted and checked for complementarity with 3′-UTRs of downregulated genes. RNAhybrid server (https://bibiserv.cebitec.uni-bielefeld.de/rnahybrid) was used to predict miRNA-mRNA interactions with seedmatch (2‒8 bp) using the helix constraint “from” and “to” parameter along with binding energy cutoff of ≤‒25 kcal/mol [31]. In the current analysis, we used stringent minimum free energy (≤‒25 kcal/mol, except for miR3613 (≤‒18 kcal/mol) due to low GC content) to predict strong putative targets with high miRNA-mRNA duplex binding stability [31]. These energy cutoffs were used based on previous studies of miR1306-ADAM10 duplex that have been experimentally validated [32]. The workflow for ChIP-Seq and RNA-Seq data integration is depicted in Fig. 1B.

Relative expressions of the gene targets identified were verified using the UALCAN (http://ualcan.path.uab.edu) and CCLE (Broad Institute Cancer Cell Line Encyclopedia, https://portals.broadinstitute.org/ccle) databases. *ACTB* gene was used as a control for relative expression analysis using the CCLE database. The UALCAN hosts the relative expression of genes across normal versus different cancer types from the TCGA (The Cancer Genome Atlas) cancer resource associated with clinicopathological data [33]. The breast cancer cell line data (60) available in CCLE database was also incorporated into the study for validation. Kaplan-Meier (KM) plots from Human Protein Atlas were used for survival analysis (https://www.proteinatlas.org/).

## Results

### ChIP-Seq analysis

**Fig. 2.**
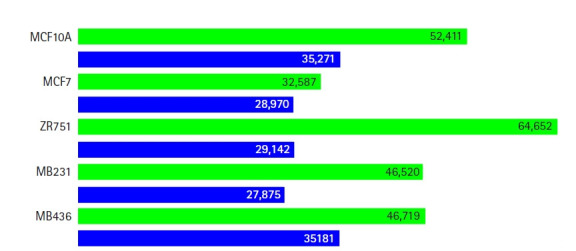
Total number of peaks predicted using MACS2 for biological replicates with H3K4me3 histone modification (p = 0.001). Replicate 1 and 2 are coloured as green and blue respectively.

All the ChIP-Seq datasets passed the quality check (Supplementary Fig. 2) and >86% of reads were mapped to the reference genome for all replicates of H3K4me3 (for each cell line) used in the study. The number of peaks in the biological replicates varied from 27875 to 64652 for different cell lines (Fig. 2). Reproducibility analysis of peaks (obtained for the replicates) enabled identification of statistically significant peaks (threshold 0.05) (Table 1, Supplementary Figs. 3, 4) which are common between biological replicates [21]. In the normal cell line (MCF10A) and luminal-A subtype (cell lines MCF7 and ZR751), 16,601 peaks (59.7%), 13,008 peaks (53.4%) and 10,158 peaks (48.1%) passed the reproducibility threshold respectively. In the triple-negative subtype (cell lines MB231 and MB436), 14,339 peaks (65.7%) and 16,549 peaks (61.2%) passed the threshold. The highest percentage of overlapping peaks between the replicates was observed in cell line MB231 (65.7%) whereas the least percentage of peaks that passed the threshold was seen in cell line ZR751 (48.1%). All cell lines except 76NF2V cell line generated reproducibility ≥ 2, which indicated that the peaks were reproducible and statistically significant. Hence, cell line 76NF2V was not used for further analysis because of the low reproducibility of replicates, N1/N2 was 3.370 (Supplementary Table 4). Chromosomal-level distribution of ChIP-peaks is available in Supplementary Fig. 5.

### miRNA promoter prediction analysis

**Fig. 3.**
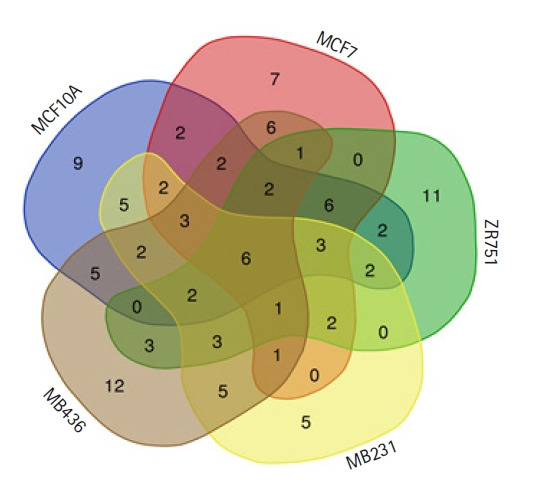
Common and unique miRNAs predicted across different cell lines.

miRNA promoter regions were identified for each cell-line (Table 2, Supplementary Table 5). Peaks corresponding to miRNA-gene promoters that are common and unique between normal versus cancerous cell lines were identified (normal vs. TNBC, normal vs. luminal-A, and TNBC vs. luminal-A) (Fig. 3, Tables 3,4). The majority of the miRNAs predicted have been reported to have a role in breast cancer (Supplementary Table 6).

Cell line‒specific miRNAs obtained in this study have been listed in Table 3. Few of these miRNAs have been validated previously [34]. It is interesting to note that there are no common miRNAs between both the luminal-A cell lines used in this study; however, five TNBC-specific miRNAs viz., miR153-1, miR4767, miR4487, miR6720, and miR-LET7I were exclusively found in both the TNBC cell lines. Identification of target genes belonging to TNBC-specific miRNAs was carried out (Supplementary Tables 7, 8). It is to be mentioned that with the cutoff criteria for target-gene identification used in this study (refer to Methods section), no targets were found for miR153-1. Of the five miRNA promoters found to be upregulated in the TNBC cell lines, 3 miRNAs, viz., miR-153-1, miR-6720, and miR-LET7I were found to have similar relative expression in TCGA data samples. Of these, miR-LET7I was found to have higher expression in TNBC (Supplementary Fig. 6).

Eight miRNAs obtained are found to be common across two cancer subtypes (Table 4). miR4512 was observed in all cancerous cell lines, both luminal-A (MCF7 and ZR751) and TNBC (MB231 and MB436) subtypes. miR3180-3 was observed in luminal-A (MCF7 and ZR751) and TNBC subtypes (MB231 and MB436). miR6791 and miR330 were observed to be common in three cancer cell lines, two luminal-A (MCF7 and ZR751) and one TNBC (MB231) subtypes. miR5787, miR6733, and miR3613 were observed in three cancer cell lines, two TNBC (MB231 and MB436) and luminal-A (ZR751) subtypes. miR6080 was observed to be present in three cancer cell lines, two TNBC (MB231 and MB436) and luminal-A (MCF7) subtypes. All the eight miRNAs listed above have been used for further downstream analysis to identify their putative gene targets based on mRNA expression data. Of the eight miRNA promoters found to be upregulated across breast cancer cell lines, the relative expression of three miRNAs, viz., miR-330, miR-3613, and miR-6733 were found to be complementary in studies reported in TCGA data samples using UALCAN webserver (Supplementary Fig. 7). The relative expression of the other five miRNAs in this resource was found to be insufficient to draw any conclusion.

### RNA-Seq analysis

All the RNA-seq datasets passed the quality check (>28) and hence were retained for further analyses (Supplementary Fig. 8). About 96% reads mapped for cell lines MCF10A, MCF7, and MB231 whereas, for cell lines ZR751 and MB436 >93% mapping was observed (Supplementary Table 9). In normal-like vs. luminal-A type, a total of 1,189 genes were upregulated (Supplementary Table 10) and 687 genes were downregulated (Supplementary Table 11). In normal-like vs. TNBC type, a total of 954 genes were upregulated (Supplementary Table 12) and 167 genes were downregulated (Supplementary Tables 13, 14, Supplementary Fig. 9). Five miRNAs specific to the TNBC cell lines were further studied to identify their binding to downregulated 3′-UTR gene targets (Supplementary Tables 8, 15).

TNBC-specific miRNA target analysis of the downregulated genes helped in the identification of 13 genes (Supplementary Table 7). Of the 13 genes, *ADAMTSL1*, *STC2*, *CPA4*, and *NUPR1* have been previously reported (Supplementary Table 6). It is interesting to note that *FOXL2* is a target for multiple miRNAs, viz., miR4767, miR4487, and miR6720 in TNBC cell lines. Comparison of these target genes to other breast cancer cell lines from the CCLE database revealed that all of them have low expression as compared to the *ACTB* control gene (Supplementary Fig. 10). Genes *STC2*, *CPA4*, and *NUPR1* were found to have a relatively higher expression amongst the 13 target genes of TNBC cell lines. In larger breast cancer samples obtained from TCGA, with the exception of *SPOCK2*, *CPA4*, *C1orf228*, and *NFE2*, the other target genes are found to have relatively low expression in TNBC as compared to normal samples (Supplementary Fig. 11). Relative expression of these genes in other cancer subtypes hints at the down-regulatory effect of TNBC-specific miRNAs. Survival plots of most of the downregulated genes (with the exception of *NUPR1*, *CPA4*, *EPHA3*, *ADAMTSL1*, and *ATP13A4*) were found to be associated with poor patient survival (Supplementary Fig. 12).

**Fig. 4.**
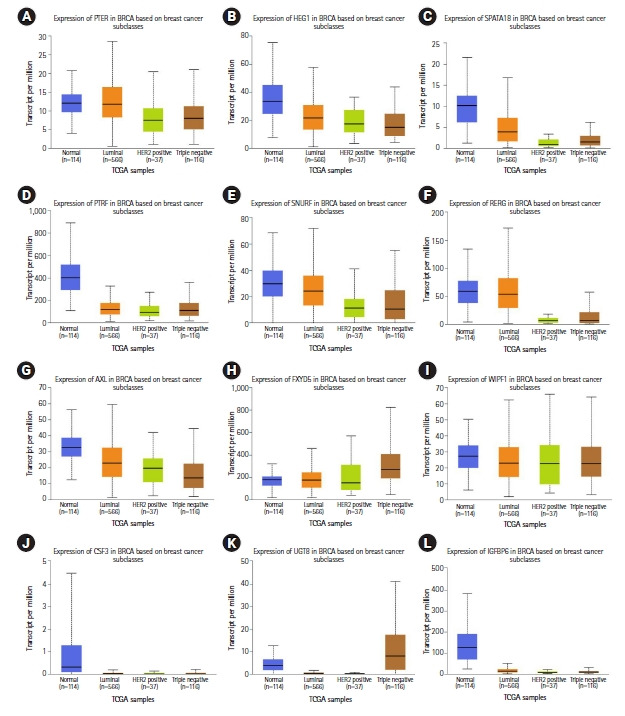
Relative gene expression (The Cancer Genome Atlas [TCGA] breast cancer samples) of luminal-A downregulated gene targets (12 of the total 17 genes) previously reported in breast cancer that correlate with predicted miRNA binding analysis: (A) PTER, (B) HEG1, (C) SPATA18, (D) PTRF, (E) SNURF, (F) RERG, (G) AXL, (H) FXYD5, (I) WIPF1, (J) CSF3, (K) UGT8, and (L) IGFBP6.

**Fig. 5.**
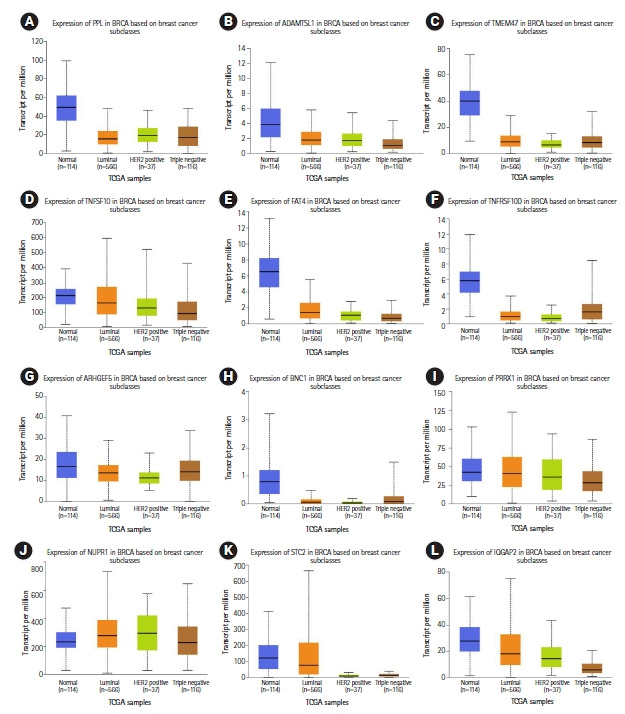
Relative gene expression (TCGA breast cancer samples) of triple-negative breast cancer downregulated gene targets (12 of the total 15 genes) previously reported in breast cancer that correlate with predicted miRNA binding analysis: (A) PPL, (B) ADAMTSL1, (C) TMEM47, (D) TNFSF10, (E) FAT4, (F) TNFRSF10D, (G) ARHGEF5, (H) BNC1, (I) PRRX1, (J) NUPR1, (K) STC2, and (L) IQGAP2.

Eight miRNA promoters that are common across more than three cancer cell lines were also used as probes to identify the gene targets (Table 4, Supplementary Tables 16‒18). A total of 44 downregulated gene targets were identified across luminal-A and TNBC subtypes. In normal-like (MCF10A) vs. luminal-A (MCF7 and ZR751) downregulated genes, 17 genes have been predicted and their role in breast cancer has been reported earlier (*RERG*, *IGFBP6*, *SPATA18*, *AXL*, *BMF*, *FXYD5*, *PTRF*, *RUNX2*, *UGT8*, *CFB*, *CSF3*, *HEG1*, *PLAU*, *PTER*, *S100A3*, *SNURF*, and *WIPF1*) [35-51] (Fig. 4, Supplementary Tables 6, 19, Supplementary Fig. 13). In normal-like (MCF10A) vs. TNBC (MB231 and MB436) downregulated genes, 15 genes have been predicted in this study and their role in breast cancer have also been previously reported (*TNFSF10*, *TMEM47*, *IQGAP2*, *FAT4*, *NUPR1*, *HOXC13*, *PRRX1*, *STC2*, AC108941.2, *ADAMTSL1*, *ARHGEF5*, *BNC1*, *CPA4*, *PPL*, and *TNFRSF10D*) [52-66] (Fig. 5, Supplementary Tables 6, 20, Supplementary Fig. 14).

Of the remaining 12 target genes identified, nine genes in luminal-A were identified to be regulated by their corresponding miRNAs (gene *A4GALT* targeted by miR3180-3, miR4512, and miR6791; gene *C10orf55* targeted by miR330, miR3180-3, miR5787, and miR6791; gene *C2orf74* targeted by miR330 and miR5787; gene *ZC4H2* targeted by miR330 and miR5787; gene *ZNF512* targeted by miR330, miR3180-3, miR5787, and miR6791; gene *ZNF655* targeted by miR5787; gene *ZNF71* targeted by miR5787 and miR6791; gene *HCG2042738* targeted by miR6791; gene *HRCT1* targeted by miR4512 and miR5787) (Table 5). Similarly, three genes in TNBC were also identified to be regulated by their corresponding miRNAs (gene *HIST3H2A* targeted by miR6791; *ZNF608* targeted by miR5787; *ELOVL4* targeted by miR5787) (Supplementary Table 18). Comparison of these 12 target genes to other breast cancer cell lines from the CCLE database revealed that all of them have low expression as compared to the *ACTB* control gene (Supplementary Fig. 15). Genes *HIST3H2A* and *C2ORF74* were found to have a relatively higher expression amongst the 12 target genes. In larger datasets of breast cancer, with the exception of *ZNF71* and *HIST3H2A*, all other gene targets were found to be downregulated (Supplementary Figs. 13, 14, 16‒18). This observation supports the probable role of miRNA-mRNA axis in gene regulation. The down-regulation of *A4GALT*, *C2ORF74*, *HRCT1*, *ZC4H2*, *ZNF512*, *ZNF655*, *ZNF608*, and *HIST3H2A* genes were found to be independently associated with poor survival in breast cancer patients (Table 5, Supplementary Fig. 19). It needs to be mentioned that relative expression data and survival plots for gene *HCG2042738* could not be obtained due to insufficient annotation.

## Discussion

The interplay between epigenetic gene regulation through histone modifications and other regulatory mechanisms like ncRNA is of great interest in cancer biology. In the present analysis, the role of H3K4me3 in miRNA expression based on promoter level peaks has been studied using ChIP-Seq and RNA-seq data integration. To achieve the same, a novel approach of mapping data derived from ChIP-Seq (miRNA promoter peaks) and RNA-Seq (targets of 3′-UTRs of genes binding to miRNA) was used to understand epigenetic regulation that may aid in the identification of subtype and cell line specific miRNAs [15,16].

In normal-like cell line MCF10A, of the nine unique miRNAs identified, miR4530 was found to have a role in the suppression of cell proliferation, promote angiogenesis and induce apoptosis by targeting gene VASH1 (Vasohibin 1) in breast carcinoma [67]. Hence, promoter-level epigenetic regulation of miR4530 by H3K4me3 may have a protective role in normal-like subtypes. miR34B was observed to be present only in cell line MB436 (TNBC subtype). miR34B has high expression in TNBC tumors compared to normal types. Expression of miR34B highly correlates with clinical outcome of patients. Notch2 (notch receptor 2) gene that has a role controlling cell differentiation, is a direct target for miR34B [68]. miR6875 was observed in TNBC cell line MB436. According to previous reports, a high expression of miR6875 was observed in early breast cancer patients [69]. miR574-5p attenuates proliferation, migration, and epithelial mesenchymal transition (EMT) in TNBC cells by targeting genes BCL11A (BAF chromatin remodeling complex subunit) and SOX2 (SRY-Box transcription factor 2) to inhibit the SKIL (SKI like proto-oncogene)/TAZ (Tafazzin)/CTGF (connective tissue growth factor) axis [70].

Of the five TNBC subtype‒specific miRNAs, mir153, miR6720, and miR-LET7I were found to be upregulated in larger breast cancer datasets belonging to TCGA. miR153 has been reported to have a tumor suppressor role and has been suggested as a prognostic marker for TNBC [34].

The majority of the predicted gene targets (total 44) overlap with previous experimental studies and include 32 gene targets (Figs. 4,5) of eight miRNAs (miR4512, miR6791, miR330, miR3180-3, miR6080, miR5787, miR6733, and miR3613) which are identified in more than three breast cancer cell lines and absent in normal-like cell lines. Overexpression of miR330-3p in breast cancer cell lines has been reported earlier, which results in greater invasiveness in-vitro, and miR330-3p-overexpressing cells also metastasize more aggressively *ex-ovo* [71]. Gene *CCBE1* (collagen and calcium binding EGF domains 1) is a direct target of miR330-3p, and knockout of *CCBE1* results in a greater invasive capacity [71]. Exosomal expression of miR3613-3p promotes breast cancer cell proliferation and metastasis. It has been previously reported that miR3613-3p levels were negatively correlated to *SOCS2* (suppressor of cytokine signaling 2) expression in breast cancer tissues [72]. Few genes were observed to be targeted by multiple miRNAs (like *A4GALT* and *FOXL2* targeted by three miRNAs each) as it is known that miRNAs can regulate multiple targets based on seed match and sequence similarity between miRNA-mRNA [10].

Of the remaining 12 gene targets, relative gene expression of genes *A4GALT*, *C2ORF74*, *HRCT1*, *ZC4H2*, *ZNF512*, *ZNF655*, and *ZNF608* agree with the proposed hypothesis of H3K4me3 regulated miRNA-mRNA axis in large patient data (TCGA samples) along with their relative expression in other breast cancer cell lines (CCLE database). These genes were associated with poor survival based on KM plots (Human Protein Atlas). The proposed methodology of miRNA-mRNA regulation when analyzed in the context of other histone modifications like H3K27me3, H3K4me1, H3K9me3 will enable better insights into the underlying mechanism of breast cancer regulation.

## Figures and Tables

**Table 1.** Details of overlapping peaks obtained after IDR analysis of all cell lines (biological replicates)

Type	Cell line	Common peaks between replicates/IDR-pass peaks	Percentage of common peaks
Normal-like	MCF10A	16,601/27,802	59.7
Luminal-A type	MCF7	13,008/24,382	53.4
	ZR751	10,158/21,103	48.1
Triple-negative type	MB231	14,339/21,821	65.7
	MB436	16,577/27,067	61.2

**Table 2.** List of H3K4me3 regulated miRNA promoter-specific peaks across cell lines

Subtype	Cell line	No. of peaks
Normal	MCF10A	53
Luminal-A	MCF7	44
	ZR751	44
TNBC	MB231	42
	MB436	54

**Table 3.** Predicted cell line specific miRNAs

Cell line	No. of unique miRNAs	miRNA
MCF10A - Normal-like	9	miR4790
		miR4687
		miR4530
		miR6892
		miR4520-1
		miR548AJ1
		miR4279
		miR1470
		miR3675
MCF7 - Luminal-A	7	miR4734
		miR4520-2
		miR4521
		miR4519
		miR4497
		miR4477B
		miR1244-3
ZR751 - Luminal-A	11	miR6850
		miR4761
		miR200C
		miR4738
		miR1282
		miR4781
		miR7706
		miR4756
		miR6090
		miR375
		miR6515
MB231 - TNBC	5	miR1260B
		miR1258
		miR7704
		miR574
		miR4651
MB436 - TNBC	12	miR34B
		miR1184-3
		miR6875
		miR6790
		miR11401
		miR4482
		miR6743
		miR148A
		miR544B
		miR4799
		miR4466
		miR9-3

TNBC, triple-negative breast cancer.

**Table 4.** Predicted TNBC and luminal-A specific miRNAs common across (≥3) cancer cell lines

Cell line	No. of common miRNAs	miRNA
MB231 (TNBC)	1	miR4512
MB436 (TNBC)	-	-
MCF7 (Luminal-A)	-	-
ZR751 (Luminal-A)	-	-
MB231 (TNBC)	2	miR6791
MCF7 (Luminal-A)		miR330
ZR751 (Luminal-A)		-
MB436 (TNBC)	1	miR3180-3
MCF7 (Luminal-A)	-	-
ZR751 (Luminal-A)	-	-
MB231 (TNBC)	1	miR6080
MB436 (TNBC)	-	-
MCF7 (Luminal-A)	-	-
MB231 (TNBC)	3	miR5787
MB436 (TNBC)	-	miR6733
ZR751 (Luminal-A)	-	miR3613

TNBC, triple-negative breast cancer.

**Table 5.** Predicted gene targets of differentially regulated miRNAs in breast cancer cell lines (TNBC and luminal-A) proposed using ChIP-Seq‒RNA-Seq integrated analysis

No.		Gene name	miRNA	Status in TCGA/CCLE/survival plot
Luminal-A				
1	A4GALT	Alpha 1,4-galactosyltransferase	miR4512, miR6791, miR3180-3	Down/down/poor-survival
2	C10orf55	Chromosome 10 open reading frame 55	miR6791, miR330, miR3180-3, miR5787	Down/down/high-survival
3	C2ORF74	Chromosome 2 open reading frame 74	miR330, miR5787	Down/down/poor-survival
4	HCG2042738	Isoform CRA_b and AC124312.1	miR6791	Down/down/poor-survival
5	HRCT1	Histidine rich carboxyl terminus 1	miR4512, miR5787	Down/down/poor-survival
6	ZC4H2	Zinc-finger family of protein	miR330, miR5787	Down/down/poor-survival
7	ZNF512	Zinc-finger protein 512	miR3180-3, miR6080, miR5787, miR6733	Down/down/poor-survival
8	ZNF655	Zinc-finger protein 655	miR5787	Down/down/poor-survival
9	ZNF71	Zinc-finger protein 71	miR6791, miR5787	Up/up/high-survival
TNBC				
1	HIST3H2A	Histone cluster 3 H2A	miR6791	Up/up/poor-survival
2	ZNF608	Zinc-finger protein 608	miR5787	Down/down/poor-survival
3	ELOVL4	ELOngation of very long chain fatty acids-4	miR5787	Down/down/high-survival

TNBC, triple-negative breast cancer; ChIP-Seq, chromatin immunoprecipitation sequencing; RNA-Seq, RNA-sequencing; TCGA, The Cancer Genome Atlas; CCLE, Broad Institute Cancer Cell Line Encyclopedia.

## Supplementary Materials

Supplementary Table 1. Gene Expression Omnibus (GEO) accession numbers for H3K4me3 chromatin immunoprecipitation sequencing data

Supplementary Table 2. Gene Expression Omnibus (GEO) accession numbers for input (control) chromatin immunoprecipitation sequencing data

Supplementary Table 3. Gene Expression Omnibus (GEO) accession numbers for RNA-sequencing data pertaining to breast cancer cell lines

Supplementary Table 4. Identification of significant threshold for peaks predicted using the IDR tool based on pseudoreplicates

Supplementary Table 5. miRNA promoter peaks identified in each cell line

Supplementary Table 6. RNA hybrid results of predicted differentially expressed miRNAs observed in more than three cancer cell lines with binding energy ≤ ‒25 kcal/mol. Genes that have been validated previously for their role in breast cancer (BC) are represented with underline. Genes that are highlighted in bold represent no previous literature support in BC.

Supplementary Table 7. Target gene list of five miRNAs present exclusively in TNBC subtype

Supplementary Table 8. RNA hybrid analysis of triple-negative breast cancer exclusive miRNA target genes

Supplementary Table 9. Details of reference mapping of RNA-sequencing data

Supplementary Table 10. List of genes upregulated in MCF10A vs. luminal-A cell lines

Supplementary Table 11. List of genes downregulated in normal-like vs. luminal-A cell lines

Supplementary Table 12. List of genes upregulated in MCF10A vs. triple-negative breast cancer cell lines

Supplementary Table 13. Differentially expressed genes in normal-like and cancer cell lines

Supplementary Table 14. List of genes downregulated in normal-like vs. triple-negative breast cancer cell lines

Supplementary Table 15. List of five miRNA sequences (exclusively present in triple-negative breast cancer subtype) used for target-gene identification

Supplementary Table 16. List of eight miRNA sequences present in at least three breast cell lines used for target-gene identification

Supplementary Table 17. RNA hybrid analysis of miRNAs (present in at least three breast cancer cell lines) target genes (17 luminal-A and 15 triple-negative breast cancer) previously reported to have role in breast cancer

Supplementary Table 18. RNA hybrid analysis of miRNAs (present in at least three breast cancer cell lines) target genes with a probable role in breast cancer

Supplementary Table 19. Details of eight miRNAs and their 3'-untranslated region targets based on downregulated genes obtained from differential expression analysis of normal-like vs. luminal-A cell lines

Supplementary Table 20. Details of eight miRNAs and their 3'-untranslated region targets based on downregulated genes obtained from differential expression analysis of normal-like vs. triple-negative breast cancer cell lines

Supplementary Fig. 1. Workflow for differential expression analysis using RNA-Seq data.

Supplementary Fig. 2. FastQC output of chromatin immunoprecipitation sequencing data pertaining to each cell line and their corresponding biological replicates.

Supplementary Fig. 3. Reproducibility analysis of replicates belonging to normal-like cell line (MCF10A).

Supplementary Fig. 4. Reproducibility analysis of replicates belonging to luminal-A and triple-negative breast cancer cell lines. (A) Replicate 1 peak ranks versus Replicate 2 peak ranks - peaks that do not pass the threshold are colored red. (B) Replicate 1 log10 peak scores versus Replicate 2 log10 peak scores - peaks that do not pass the threshold are colored red. (C, D) Peak rank versus IDR scores are plotted in black.

Supplementary Fig. 5. Annotation of peaks identified for each breast cancer cell line. ChIP, chromatin immunoprecipitation.

Supplementary Fig. 6. Relative gene expression of triple-negative breast cancer subtype exclusive miRNAs from The Cancer Genome Atlas (TCGA) data samples. (A) miR-153-1. (B) miR-6720. (C) miR-Let7i.

Supplementary Fig. 7. Relative gene expression of triple-negative breast cancer and luminal-A specific miRNAs from The Cancer Genome Atlas (TCGA) data samples. (A) miR-330. (B) miR-3613. (C) miR-6733.

Supplementary Fig. 8. FastQC output of RNA-sequencing (RNA-Seq) data pertaining to each cell-line and their corresponding biological replicates.

Supplementary Fig. 9. Differential expression analysis of RNA sequencing data: Volcano plots.

Supplementary Fig. 10. Relative gene expression of triple-negative breast cancer subtype exclusive miRNA targets from the Broad Institute Cancer Cell Line Encyclopedia (CCLE) database.

Supplementary Fig. 11. Relative gene expression of triple-negative breast cancer subtype exclusive miRNA targets from The Cancer Genome Atlas (TCGA) data samples.

Supplementary Fig. 12. Five-year KM-survival plots from human protein atlas web server: triple-negative breast cancer subtype exclusive miRNA target genes.

Supplementary Fig. 13. Relative gene expression of (The Cancer Genome Atlas [TCGA] breast cancer samples) luminal-A downregulated gene targets (5 of the total 17 genes) previously reported in breast cancer that do not correlate with predicted miRNA binding analysis.

Supplementary Fig. 14. Relative gene expression of (The Cancer Genome Atlas [TCGA] breast cancer samples) triple-negative breast cancer downregulated gene targets (2 out of total 15 genes) previously reported in breast cancer predicted that do not correlate with predicted miRNA binding analysis.

Supplementary Fig. 15. Relative gene expression of triple-negative breast cancer and luminal-A specific miRNAs gene targets from Broad Institute Cancer Cell Line Encyclopedia (CCLE) database (*ACTB* gene expression as control).

Supplementary Fig. 16. Relative gene expression of triple-negative breast cancer and luminal-A specific miRNAs gene targets in The Cancer Genome Atlas (TCGA) samples: stage-wise expression.

Supplementary Fig. 17. Relative gene expression of triple-negative breast cancer and luminal-A specific miRNAs gene targets in The Cancer Genome Atlas (TCGA) samples.

Supplementary Fig. 18. Relative gene expression of triple-negative breast cancer and luminal-A specific miRNAs gene targets in The Cancer Genome Atlas (TCGA) samples:across cancer subtypes.

Supplementary Fig. 19. Five-year KM-survival plots from Human Protein Atlas: triple-negative breast cancer and luminal-A specific miRNAs gene targets.
