# Supplementary material for: Analysis of H3K4me3-ChIP-Seq and RNA-Seq data to understand the putative role of miRNAs and their target genes in breast cancer cell lines
Source: Genomics Inform. 2021 Jun 30;19(2):e17. doi: 10.5808/gi.21020 (PMC8261273; doi:10.5808/gi.21020)
Supplement: Supplementary Table 1. — Gene Expression Omnibus (GEO) accession numbers for H3K4me3 chromatin immunoprecipitation sequencing data [file gi-21020suppl1.docx]

**Supplementary Table 1.** Gene Expression Omnibus (GEO) accession numbers for H3K4me3 chromatin immunoprecipitation sequencing data

| Cell line | Histone modification | GEO accession ID  Rep1 | GEO accession ID  Rep2 |
| --- | --- | --- | --- |
| MCF10A | H3K4me3 | SRR3997201 | SRR3997202 |
| MCF7 | H3K4me3 | SRR3997219 | SRR3997220 |
| ZR751 | H3K4me3 | SRR3997237 | SRR3997238 |
| MB231 | H3K4me3 | SRR3997345 | SRR3997346 |
| MB436 | H3K4me3 | SRR3997363 | SRR3997364 |
