# Supplementary material for: Analysis of H3K4me3-ChIP-Seq and RNA-Seq data to understand the putative role of miRNAs and their target genes in breast cancer cell lines
Source: Genomics Inform. 2021 Jun 30;19(2):e17. doi: 10.5808/gi.21020 (PMC8261273; doi:10.5808/gi.21020)
Supplement: Supplementary Table 2. — Gene Expression Omnibus (GEO) accession numbers for input (control) chromatin immunoprecipitation sequencing data [file gi-21020suppl2.docx]

**Supplementary Table 2.** Gene Expression Omnibus (GEO) accession numbers for input (control) chromatin immunoprecipitation sequencing data

| Cell line | Histone modification | GEO accession ID  Rep1 | GEO accession ID  Rep2 |
| --- | --- | --- | --- |
| MCF10A | Input | SRR3997209 | SRR3997210 |
| MCF7 | Input | SRR3997227 | SRR3997228 |
| ZR751 | Input | SRR3997245 | SRR3997246 |
| MB231 | Input | SRR3997353 | SRR3997354 |
| MB436 | Input | SRR3997371 | SRR3997372 |
