# Supplementary material for: Analysis of H3K4me3-ChIP-Seq and RNA-Seq data to understand the putative role of miRNAs and their target genes in breast cancer cell lines
Source: Genomics Inform. 2021 Jun 30;19(2):e17. doi: 10.5808/gi.21020 (PMC8261273; doi:10.5808/gi.21020)
Supplement: Supplementary Table 3. — Gene Expression Omnibus (GEO) accession numbers for RNA-sequencing data pertaining to breast cancer cell lines [file gi-21020suppl3.docx]

**Supplementary Table 3.** Gene Expression Omnibus (GEO) accession numbers for RNA-sequencing data

pertaining to breast cancer cell lines

| Cell line | GEO accession ID  Rep1 | GEO accession ID  Rep2 | GEO accession ID  Rep3 | GEO accession ID  Rep4 |
| --- | --- | --- | --- | --- |
| MCF10A | SRR5364106 | SRR5364107 | SRR5364108 | SRR5364109 |
| MCF7 | SRR5364110 | SRR5364111 | SRR5364112 | SRR5364113 |
| ZR751 | SRR5364114 | SRR5364115 | SRR5364116 | SRR5364117 |
| MB231 | SRR5364146 | SRR5364147 | SRR5364148 | SRR5364149 |
| MB436 | SRR5364150 | SRR5364151 | SRR5364152 | SRR5364153 |
