# Supplementary material for: Analysis of H3K4me3-ChIP-Seq and RNA-Seq data to understand the putative role of miRNAs and their target genes in breast cancer cell lines
Source: Genomics Inform. 2021 Jun 30;19(2):e17. doi: 10.5808/gi.21020 (PMC8261273; doi:10.5808/gi.21020)
Supplement: Supplementary Table 4. — Identification of significant threshold for peaks predicted using the IDR tool based on pseudoreplicates [file gi-21020suppl4.docx]

**Supplementary Table 4.** Identification of significant threshold for peaks predicted using the IDR tool based on pseudoreplicates

| Cell line | IDR threshold | N1 | N2 | N1/N2 | Np | Nt | Np/Nt |
| --- | --- | --- | --- | --- | --- | --- | --- |
| 76NF2V | 0.05 | 18,718 | 5554 | 3.370 | 20,478 | 15,451 | 1.325 |
| MCF10A | 0.05 | 17,496 | 16,871 | 1.037 | 20,170 | 16,601 | 1.215 |
| MCF7 | 0.05 | 15,989 | 13,885 | 1.151 | 16,717 | 13,008 | 1.285 |
| ZR751 | 0.05 | 18,789 | 17,011 | 1.104 | 18,914 | 10,158 | 1.862 |
| MB231 | 0.05 | 18,545 | 15,079 | 1.229 | 19,739 | 14,339 | 1.376 |
| MB436 | 0.05 | 18,040 | 17,579 | 1.02 | 19,845 | 16,577 | 1.197 |

Cell line 76NF2V did not satisfy the N1/N2 ≤ 2 criterion and hence has been excluded for further analysis. N1, number of Rep1 self-consistent peaks; N2, number of Rep2 self-consistent peaks; Np, number of peaks consistent between pooled pseudoReps; Nt, number of peaks consistent between true replicates.
