# Supplementary material for: Analysis of H3K4me3-ChIP-Seq and RNA-Seq data to understand the putative role of miRNAs and their target genes in breast cancer cell lines
Source: Genomics Inform. 2021 Jun 30;19(2):e17. doi: 10.5808/gi.21020 (PMC8261273; doi:10.5808/gi.21020)
Supplement: Supplementary Table 5. — miRNA promoter peaks identified in each cell line [file gi-21020suppl5.pdf]

miRNA promoter peaks identified in each cell-line				
**Normal-like**	**Luminal-A**		**TNBC**	
**MCF10A**	**MCF7**	**ZR751**	**MB231**	**MB436**
MIR7111	MIR330	MIR6850	MIR636	MIR6743
MIR6892	MIR1184-1	MIR1282	MIR4651	MIR22
MIR22	MIR3184	MIR3665	MIR2277	MIR132
MIR4530	MIR4634	MIR4512	MIR12115	MIR8072
MIR4757	MIR6080	MIR6791	MIR6733	MIR92B
MIR4687	MIR4521	MIR4665	MIR6791	MIR544B
MIR616	MIR12115	MIR616	MIR4757	MIR3665
MIR212	MIR3143	MIR6515	MIR574	MIR6875
MIR92B	MIR301B	MIR203A	MIR5187	MIR4426
MIR5187	MIR132	MIR636	MIRLET7I	MIR1184-2
MIR4665	MIR3665	MIR200C	MIR616	MIR301B
MIR933	MIR615	MIR301B	MIR301B	MIR4512
MIR762	MIR6791	MIR4757	MIR6720	MIR4466
MIR760	MIR4664	MIR4634	MIR330	MIR137
MIR9899	MIR203A	MIR330	MIR3184	MIR3613
MIR4664	MIR3180-1	MIR933	MIR5787	MIR4767
MIR1470	MIR3180-3	MIR3184	MIR1260B	MIR6790
MIR12115	MIR8069-2	MIR6733	MIR4512	MIR2277
MIR301B	MIR616	MIR4781	MIR3613	MIR1184-3
MIR137	MIR762	MIR5087	MIR6080	MIR148A
MIR4634	MIR3180-5	MIR5187	MIR762	MIR9718
MIR3178	MIR4497	MIR3178	MIR760	MIRLET7I
MIR8072	MIR2277	MIR212	MIR4767	MIR1184-1
MIR5087	MIR3180-4	MIR7111	MIR3652	MIR6733
MIR2277	MIR5087	MIR4664	MIR3178	MIR6720
MIR203A	MIR4512	MIR9718	MIR7704	MIR4482
MIR615	MIR12124	MIR375	MIR5087	MIR4757
MIR636	MIR9899	MIR6090	MIR933	MIR4634
MIR3652	MIR4519	MIR3613	MIR4710	MIR6821

MIR3184	MIR4665	MIR4756	MIR153-1	MIR5787
MIR3665	MIR4426	MIR6724-4	MIR3181	MIR615
MIR4710	MIR636	MIR6724-2	MIR4787	MIR4783
MIR3180-1	MIR4477B	MIR6724-3	MIR1258	MIR5087
MIR3180-4	MIR6724-4	MIR5787	MIR7155	MIR8069-2
MIR4787	MIR4783	MIR3180-1	MIR9-2	MIR34B
MIR4790	MIR4645	MIR6724-1	MIR3180-1	MIR7155
MIR3180-5	MIR4734	MIR8072	MIR615	MIR6080
MIR6724-3	MIR6724-2	MIR7706	MIR4487	MIR4487
MIR6724-4	MIR4520-2	MIR4738	MIR8072	MIR3143
MIR7155	MIR4757	MIR1184-2	MIR4634	MIR9-3
MIR6724-2	MIR6724-1	MIR3180-5	MIR3180-4	MIR3180-1
MIR4783	MIR1244-3	MIR3180-3	MIR3665	MIR5187
MIR548AJ1	MIR6724-3	MIR4761		MIR3180-3
MIR9-2	MIR1244-1	MIR6821		MIR3180-4
MIR4279				MIR203A
MIR3181				MIR4710
MIR4520-1				MIR153-1
MIR1244-1				MIR12124
MIR30A				MIR4665
MIR6724-1				MIR4323
MIR4323				MIR4799
MIR3675				MIR30A
MIR8069-2				MIR4645
				MIR11401
