# Supplementary material for: Analysis of H3K4me3-ChIP-Seq and RNA-Seq data to understand the putative role of miRNAs and their target genes in breast cancer cell lines
Source: Genomics Inform. 2021 Jun 30;19(2):e17. doi: 10.5808/gi.21020 (PMC8261273; doi:10.5808/gi.21020)
Supplement: Supplementary Table 6. — RNA hybrid results of predicted differentially expressed miRNAs observed in more than three cancer cell lines with binding energy ≤ ‒25 kcal/mol. Genes that have been validated previously for their role in breast cancer (BC) are represented with underline. Genes that are highlighted in bold represent no previous literature support in BC. [file gi-21020suppl6.docx]

**Supplementary Table 6.** RNA hybrid results of predicted differentially expressed miRNAs observed in more than three cancer cell lines with binding energy ≤ ‒25 kcal/mol

| miRNA | Predicted expression in cell-lines | Normal-like vs. luminal-A downregulated  3′ UTR targets (#, genes) | Normal-like vs. TNBC downregulated  3’ UTR targets (#, genes) |
| --- | --- | --- | --- |
| miR4512 | MB-231 MB-436  MCF7  ZR751 | 6 (**A4GALT**[[1]](https://www.zotero.org/google-docs/?ZS92np), BMF[[2]](https://www.zotero.org/google-docs/?sZWyJx), CSF3[[3]](https://www.zotero.org/google-docs/?mLrTBr),**HRCT1**, S100A3[[4]](https://www.zotero.org/google-docs/?kXaoMf), SNURF[[5]](https://www.zotero.org/google-docs/?8PNcJX)) | 1 (PRRX1[[6]](https://www.zotero.org/google-docs/?n2u45Q)) |
| miR6791 | MB-231 MCF7 ZR751 | 15 (**A4GALT**[[1]](https://www.zotero.org/google-docs/?YPRNMY), BMF[[2]](https://www.zotero.org/google-docs/?D0IxAz), **C10orf55**[[7]](https://www.zotero.org/google-docs/?mb57lv), CSF3[[3]](https://www.zotero.org/google-docs/?unmPpC), CFB[[8]](https://www.zotero.org/google-docs/?1MertW), FXYD5[[9]](https://www.zotero.org/google-docs/?rW1xwf), HEG1[[10]](https://www.zotero.org/google-docs/?4nQfKg), PTRF[[11]](https://www.zotero.org/google-docs/?xgdu3j), RUNX2[[12]](https://www.zotero.org/google-docs/?upnevb), S100A3[[4]](https://www.zotero.org/google-docs/?ClqSRg), SNURF[[5]](https://www.zotero.org/google-docs/?TLgjI2), **HCG2042738**, UGT8[[13]](https://www.zotero.org/google-docs/?UjqQU9), WIPF1[[14]](https://www.zotero.org/google-docs/?VkcxNf), **ZNF71**[[15]](https://www.zotero.org/google-docs/?yShet8)) | 8 (AC108941.2[[16]](https://www.zotero.org/google-docs/?9GdjHG), ADAMTSL1[[17]](https://www.zotero.org/google-docs/?WrCmGw), BNC1[[18]](https://www.zotero.org/google-docs/?MEYwnO), **HIST3H2A**[[19]](https://www.zotero.org/google-docs/?QlvRDY), NUPR1[[20]](https://www.zotero.org/google-docs/?uVJ1hV), PRRX1[[6]](https://www.zotero.org/google-docs/?9dKEHT), STC2[[21]](https://www.zotero.org/google-docs/?oMCcvL), TNFRSF10D[[22]](https://www.zotero.org/google-docs/?ERXpGR)) |
| miR330 | MB-231 MCF7 ZR751 | 9 (**C10orf55**[[7]](https://www.zotero.org/google-docs/?9G1Wph), **C2orf74**[[23]](https://www.zotero.org/google-docs/?xF9Wq2), CSF3[[3]](https://www.zotero.org/google-docs/?jGrnul), CFB[[8]](https://www.zotero.org/google-docs/?e0HnR5), FXYD5[[9]](https://www.zotero.org/google-docs/?swOnxv), HEG1[[10]](https://www.zotero.org/google-docs/?K4milM), PTRF[[11]](https://www.zotero.org/google-docs/?9xLThM), S100A3[[4]](https://www.zotero.org/google-docs/?dK4yPc), **ZC4H2**[[24]](https://www.zotero.org/google-docs/?fp8ax6)) | 6 (ADAMTSL1[[17]](https://www.zotero.org/google-docs/?unK9ia), BNC1[[18]](https://www.zotero.org/google-docs/?eYkJl2), HOXC13[[25]](https://www.zotero.org/google-docs/?LH6ox4), NUPR1[[20]](https://www.zotero.org/google-docs/?3eugh5), ARHGEF5[[26]](https://www.zotero.org/google-docs/?4HvXxZ),STC2[[21]](https://www.zotero.org/google-docs/?5gDICk)) |
| miR3180-3 | MB-436 MCF7 ZR751 | 7 (**A4GALT**[[1]](https://www.zotero.org/google-docs/?Tttgql), **C10orf55**[[7]](https://www.zotero.org/google-docs/?hhwW1h), HEG1[[10]](https://www.zotero.org/google-docs/?hrozuM), PLAU[[27]](https://www.zotero.org/google-docs/?Utr8Dh), S100A3[[4]](https://www.zotero.org/google-docs/?ksl5VJ), WIPF1[[14]](https://www.zotero.org/google-docs/?86xZ3C), **ZNF512**[[28]](https://www.zotero.org/google-docs/?jP6NSM)) | 2 (FAT4[[29]](https://www.zotero.org/google-docs/?Dn5Ie6), NUPR1[[20]](https://www.zotero.org/google-docs/?6nXOrq)) |
| miR6080 | MB-231 MB-436 MCF7 | 1 (**ZNF512**[[28]](https://www.zotero.org/google-docs/?AapWfG)) | 1 (ARHGEF5[[26]](https://www.zotero.org/google-docs/?X2Esbp)) |
| miR5787 | MB-231 MB-436 ZR751 | 21 (AXL[[30]](https://www.zotero.org/google-docs/?LsVXCy), BMF[[2]](https://www.zotero.org/google-docs/?b4DfGv), **C10orf55**[[7]](https://www.zotero.org/google-docs/?DB7yjl), **C2orf74**[[23]](https://www.zotero.org/google-docs/?Uk5rCK), CFB[[8]](https://www.zotero.org/google-docs/?u1TUnq), FXYD5[[9]](https://www.zotero.org/google-docs/?ESFjva), HEG1[[10]](https://www.zotero.org/google-docs/?7FViHU), **HRCT1**, IGFBP6[[31]](https://www.zotero.org/google-docs/?tnRC50), PTER[[32]](https://www.zotero.org/google-docs/?Q7XrZk), PTRF[[11]](https://www.zotero.org/google-docs/?kCCLXI), RERG[[33]](https://www.zotero.org/google-docs/?h8JJzh), RUNX2[[12]](https://www.zotero.org/google-docs/?hrnava), S100A3[[4]](https://www.zotero.org/google-docs/?hSUISL), SPATA18[[34]](https://www.zotero.org/google-docs/?Gdl1na), UGT8[[13]](https://www.zotero.org/google-docs/?oAAvA1), WIPF1[[14]](https://www.zotero.org/google-docs/?C9UyDC), **ZC4H2**[[24]](https://www.zotero.org/google-docs/?O7zNlT), **ZNF512**[[28]](https://www.zotero.org/google-docs/?qxJsGP), **ZNF655**[[35]](https://www.zotero.org/google-docs/?ZF08R9), **ZNF71**[[15]](https://www.zotero.org/google-docs/?1IoeOo)) | 14 (ADAMTSL1[[17]](https://www.zotero.org/google-docs/?G8yI7P), BNC1[[18]](https://www.zotero.org/google-docs/?8hTcZC), CPA4[[36]](https://www.zotero.org/google-docs/?GLJ9ym), **ELOVL4**[[37]](https://www.zotero.org/google-docs/?lTjqzH), HOXC13[[25]](https://www.zotero.org/google-docs/?TFkLvj), NUPR1[[20]](https://www.zotero.org/google-docs/?EZZh08), PRRX1[[6]](https://www.zotero.org/google-docs/?geY8RZ), PPL[[38]](https://www.zotero.org/google-docs/?czVTIG), ARHGEF5[[26]](https://www.zotero.org/google-docs/?5fMxZ8), STC2[[21]](https://www.zotero.org/google-docs/?AX95xV), TNFRSF10D[[22]](https://www.zotero.org/google-docs/?ll7Svo), TMEM47[[39]](https://www.zotero.org/google-docs/?usjVjj), TNFSF10[[40]](https://www.zotero.org/google-docs/?lT2llh), **ZNF608**[[41]](https://www.zotero.org/google-docs/?Hry4zC)) |
| miR6733 | MB-231 MB-436 ZR751 | 1 (**ZNF512**[[28]](https://www.zotero.org/google-docs/?pvQlft)) | 1 (TNFRSF10D[[22]](https://www.zotero.org/google-docs/?BX6EwH)) |
| miR3613 | MB-231 MB-436 ZR751 | 2 (≤ -18 kcal/mol)  (UGT8[[13]](https://www.zotero.org/google-docs/?WZLzBX), WIPF1[[14]](https://www.zotero.org/google-docs/?jLKzrS)) | 3 (≤ -18 kcal/mol)  (ADAMTSL1[[17]](https://www.zotero.org/google-docs/?Ffm350), IQGAP2[[42]](https://www.zotero.org/google-docs/?QQr0Za), PRRX1[[6]](https://www.zotero.org/google-docs/?chlgeS)) |

Genes that have been validated previously for their role in breast cancer (BC) are represented with underline. Genes that are highlighted in bold represent no previous literature support in BC.

TNBC, triple-negative breast cancer; UTR, untranslated region.

**References**

1. Jacob F, Alam S, Konantz M, Liang CY, Kohler RS, Everest-Dass AV, et al. Transition of mesenchymal and epithelial cancer cells depends on alpha1-4 galactosyltransferase-mediated glycosphingolipids. Cancer Res 2018;78:2952-2965.

2. Hornsveld M, Tenhagen M, van de Ven RA, Smits AM, van Triest MH, van Amersfoort M, et al. Restraining FOXO3-dependent transcriptional BMF activation underpins tumour growth and metastasis of E-cadherin-negative breast cancer. Cell Death Differ 2016;23:1483-1492.

3. Hollmen M, Karaman S, Schwager S, Lisibach A, Christiansen AJ, Maksimow M, et al. G-CSF regulates macrophage phenotype and associates with poor overall survival in human triple-negative breast cancer. Oncoimmunology 2016;5:e1115177.

4. Gianni M, Terao M, Kurosaki M, Paroni G, Brunelli L, Pastorelli R, et al. S100A3 a partner protein regulating the stability/activity of RARalpha and PML-RARalpha in cellular models of breast/lung cancer and acute myeloid leukemia. Oncogene 2019;38:2482-2500.

5. Saville B, Poukka H, Wormke M, Janne OA, Palvimo JJ, Stoner M, et al. Cooperative coactivation of estrogen receptor alpha in ZR-75 human breast cancer cells by SNURF and TATA-binding protein. J Biol Chem 2002;277:2485-2497.

6. Dong J, Lv Z, Chen Q, Wang X, Li F. PRRX1 drives tamoxifen therapy resistance through induction of epithelial-mesenchymal transition in MCF-7 breast cancer cells. Int J Clin Exp Pathol 2018;11:2629-2635.

7. Hayward CP, Liang M, Tasneem S, Soomro A, Waye JS, Paterson AD, et al. The duplication mutation of Quebec platelet disorder dysregulates PLAU, but not C10orf55, selectively increasing production of normal PLAU transcripts by megakaryocytes but not granulocytes. PLoS One 2017;12:e0173991.

8. Suman S, Basak T, Gupta P, Mishra S, Kumar V, Sengupta S, et al. Quantitative proteomics revealed novel proteins associated with molecular subtypes of breast cancer. J Proteomics 2016;148:183-193.

9. Lee YK, Lee SY, Park JR, Kim RJ, Kim SR, Roh KJ, et al. Dysadherin expression promotes the motility and survival of human breast cancer cells by AKT activation. Cancer Sci 2012;103:1280-1289.

10. Zhao YR, Wang JL, Xu C, Li YM, Sun B, Yang LY. HEG1 indicates poor prognosis and promotes hepatocellular carcinoma invasion, metastasis, and EMT by activating Wnt/beta-catenin signaling. Clin Sci (Lond) 2019;133:1645-1662.

11. Bai L, Deng X, Li Q, Wang M, An W, Deli A, et al. Down-regulation of the cavin family proteins in breast cancer. J Cell Biochem 2012;113:322-328.

12. McDonald L, Ferrari N, Terry A, Bell M, Mohammed ZM, Orange C, et al. RUNX2 correlates with subtype-specific breast cancer in a human tissue microarray, and ectopic expression of Runx2 perturbs differentiation in the mouse mammary gland. Dis Model Mech 2014;7:525-534.

13. Cao Q, Chen X, Wu X, Liao R, Huang P, Tan Y, et al. Inhibition of UGT8 suppresses basal-like breast cancer progression by attenuating sulfatide-alphaVbeta5 axis. J Exp Med 2018;215:1679-1692.

14. Garcia E, Machesky LM, Jones GE, Anton IM. WIP is necessary for matrix invasion by breast cancer cells. Eur J Cell Biol 2014;93:413-423.

15. Guo NL, Dowlati A, Raese RA, Dong C, Chen G, Beer DG, et al. A predictive 7-gene assay and prognostic protein biomarkers for non-small cell lung cancer. EBioMedicine 2018;32:102-110.

16. Ji XW, Zhou TY, Lu Y, Wei MJ, Lu W, Cho WC. Breast cancer treatment and sulfotransferase. Expert Opin Ther Targets 2015;19:821-834.

17. Li Z, Guo X, Wu Y, Li S, Yan J, Peng L, et al. Methylation profiling of 48 candidate genes in tumor and matched normal tissues from breast cancer patients. Breast Cancer Res Treat 2015;149:767-779.

18. Pangeni RP, Channathodiyil P, Huen DS, Eagles LW, Johal BK, Pasha D, et al. The GALNT9, BNC1 and CCDC8 genes are frequently epigenetically dysregulated in breast tumours that metastasise to the brain. Clin Epigenetics 2015;7:57.

19. Ye XY, Xu L, Lu S, Chen ZW. MiR-516a-5p inhibits the proliferation of non-small cell lung cancer by targeting HIST3H2A. Int J Immunopathol Pharmacol 2019;33:2058738419841481.

20. Chowdhury UR, Samant RS, Fodstad O, Shevde LA. Emerging role of nuclear protein 1 (NUPR1) in cancer biology. Cancer Metastasis Rev 2009;28:225-232.

21. Hou J, Wang Z, Xu H, Yang L, Yu X, Yang Z, et al. Stanniocalicin 2 suppresses breast cancer cell migration and invasion via the PKC/claudin-1-mediated signaling. PLoS One 2015;10:e0122179.

22. Sanlioglu AD, Korcum AF, Pestereli E, Erdogan G, Karaveli S, Savas B, et al. TRAIL death receptor-4 expression positively correlates with the tumor grade in breast cancer patients with invasive ductal carcinoma. Int J Radiat Oncol Biol Phys 2007;69:716-723.

23. Pintarelli G, Dassano A, Cotroneo CE, Galvan A, Noci S, Piazza R, et al. Read-through transcripts in normal human lung parenchyma are down-regulated in lung adenocarcinoma. Oncotarget 2016;7:27889-27898.

24. Ma P, Ren B, Yang X, Sun B, Liu X, Kong Q, et al. ZC4H2 stabilizes Smads to enhance BMP signalling, which is involved in neural development in *Xenopus*. Open Biol 2017;7:170122.

25. Li C, Cui J, Zou L, Zhu L, Wei W. Bioinformatics analysis of the expression of HOXC13 and its role in the prognosis of breast cancer. Oncol Lett 2020;19:899-907.

26. Debily MA, Camarca A, Ciullo M, Mayer C, El Marhomy S, Ba I, et al. Expression and molecular characterization of alternative transcripts of the ARHGEF5/TIM oncogene specific for human breast cancer. Hum Mol Genet 2004;13:323-334.

27. Lin M, Zhang Z, Gao M, Yu H, Sheng H, Huang J. MicroRNA-193a-3p suppresses the colorectal cancer cell proliferation and progression through downregulating the PLAU expression. Cancer Manag Res 2019;11:5353-5363.

28. Bao L, Zhang Y, Wang J, Wang H, Dong N, Su X, et al. Variations of chromosome 2 gene expressions among patients with lung cancer or non-cancer. Cell Biol Toxicol 2016;32:419-435.

29. Hou L, Chen M, Zhao X, Li J, Deng S, Hu J, et al. FAT4 functions as a tumor suppressor in triple-negative breast cancer. Tumour Biol 2016;37:16337-16343.

30. Colavito SA. AXL as a target in breast cancer therapy. J Oncol 2020;2020:5291952.

31. Nikulin SV, Raigorodskaya MP, Poloznikov AA, Zakharova GS, Schumacher U, Wicklein D, et al. In vitro model for studying of the role of *IGFBP6* gene in breast cancer metastasizing. Bull Exp Biol Med 2018;164:688-692.

32. Hung CM, Liu LC, Ho CT, Lin YC, Way TD. Pterostilbene enhances TRAIL-induced apoptosis through the induction of death receptors and downregulation of cell survival proteins in TRAIL-resistance triple negative breast cancer cells. J Agric Food Chem 2017;65:11179-11191.

33. Hsu PC, Ho JY, Yu CP. RERG involvement in the RAS pathway and ER-dependent transcription in breast cancer. J Clin Oncol 2019;37(15 Suppl):e14638.

34. Bornstein C, Brosh R, Molchadsky A, Madar S, Kogan-Sakin I, Goldstein I, et al. SPATA18, a spermatogenesis-associated gene, is a novel transcriptional target of p53 and p63. Mol Cell Biol 2011;31:1679-1689.

35. Yang C, Zheng J, Liu X, Xue Y, He Q, Dong Y, et al. Role of ANKHD1/LINC00346/ZNF655 feedback loop in regulating the glioma angiogenesis via staufen1-mediated mRNA decay. Mol Ther Nucleic Acids 2020;20:866-878.

36. Bademler S, Ucuncu MZ, Tilgen Vatansever C, Serilmez M, Ertin H, Karanlik H. Diagnostic and prognostic significance of carboxypeptidase A4 (CPA4) in breast cancer. Biomolecules 2019;9:103.

37. Hopiavuori BR, Anderson RE, Agbaga MP. ELOVL4: Very long-chain fatty acids serve an eclectic role in mammalian health and function. Prog Retin Eye Res 2019;69:137-158.

38. Sun C, Gu Y, Chen G, Du Y. Bioinformatics analysis of stromal molecular signatures associated with breast and prostate cancer. J Comput Biol 2019;26:1130-1139.

39. Guo L, Zhang K, Bing Z. Application of a coexpression network for the analysis of aggressive and nonaggressive breast cancer cell lines to predict the clinical outcome of patients. Mol Med Rep 2017;16:7967-7978.

40. He W, Wang Q, Xu J, Xu X, Padilla MT, Ren G, et al. Attenuation of TNFSF10/TRAIL-induced apoptosis by an autophagic survival pathway involving TRAF2- and RIPK1/RIP1-mediated MAPK8/JNK activation. Autophagy 2012;8:1811-1821.

41. Baez-Vega PM, Echevarria Vargas IM, Valiyeva F, Encarnacion-Rosado J, Roman A, Flores J, et al. Targeting miR-21-3p inhibits proliferation and invasion of ovarian cancer cells. Oncotarget 2016;7:36321-36337.

42. Kumar D, Hassan MK, Pattanaik N, Mohapatra N, Dixit M. IQGAP2 acts as a tumor suppressor in breast cancer and its reduced expression promotes cancer growth and metastasis by MEK/ERK signalling pathways. Preprint at: https://doi.org/10.1101/651034 (2019).
