# Supplementary material for: Analysis of H3K4me3-ChIP-Seq and RNA-Seq data to understand the putative role of miRNAs and their target genes in breast cancer cell lines
Source: Genomics Inform. 2021 Jun 30;19(2):e17. doi: 10.5808/gi.21020 (PMC8261273; doi:10.5808/gi.21020)
Supplement: Supplementary Table 7. — Target gene list of five miRNAs present exclusively in TNBC subtype [file gi-21020suppl7.docx]

**Supplementary Table 7.** Target gene list of five miRNAs present exclusively in TNBC subtype

| miRNA | TNBC downregulated 3′-UTR targets from RNA hybrid analysis | Gene names |
| --- | --- | --- |
| miR153-1 | No hits | NA |
| miR4767 | 3 | STC2, **FOXL2**, MGAT4C |
| miR4487 | 5 | CPA4, ATP13A4, ADAMTSL1, SPOCK2, **FOXL2** |
| miR6720 | 6 | GAL3ST3, NFE2, NUPR1, **FOXL2**, EPHA3, COL25A1 |
| miR-LET7I | 1 | C1orf228 |

TNBC, triple-negative breast cancer; UTR, untranslated region.
