# Supplementary material for: Analysis of H3K4me3-ChIP-Seq and RNA-Seq data to understand the putative role of miRNAs and their target genes in breast cancer cell lines
Source: Genomics Inform. 2021 Jun 30;19(2):e17. doi: 10.5808/gi.21020 (PMC8261273; doi:10.5808/gi.21020)
Supplement: Supplementary Table 8. — RNA hybrid analysis of triple-negative breast cancer exclusive miRNA target genes [file gi-21020suppl8.docx]

**Supplementary Table 8.** RNA hybrid analysis of triple-negative breast cancer exclusive miRNA target genes

| **miRNA** | **Putative target gene** | **RNA hybrid result** | **Binding energy (kcal/mol)** |
| --- | --- | --- | --- |
| TNBC - Thirteen gene targets by miRNAs | | | |
| miR4767 | STC2 | **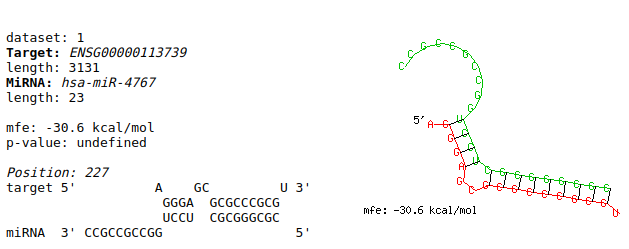** | -30.6 |
| miR4767 | FOXL2 | **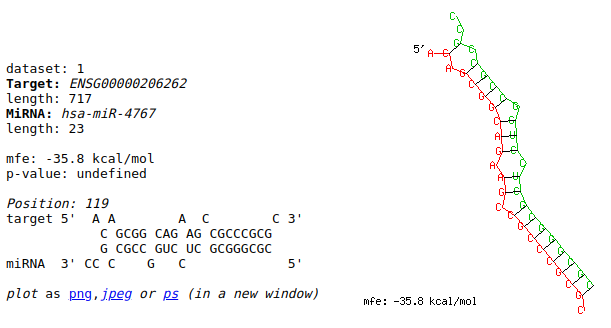** | -35.8 |
| miR4767 | MGAT4C | **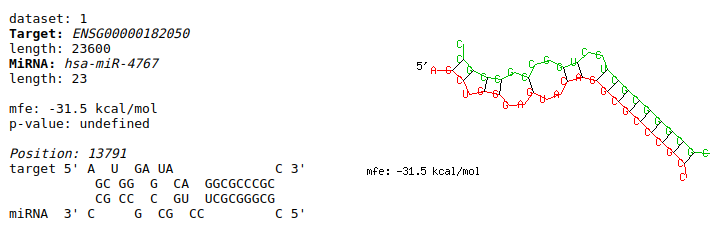** | -31.5 |
| miR4487 | CPA4 | **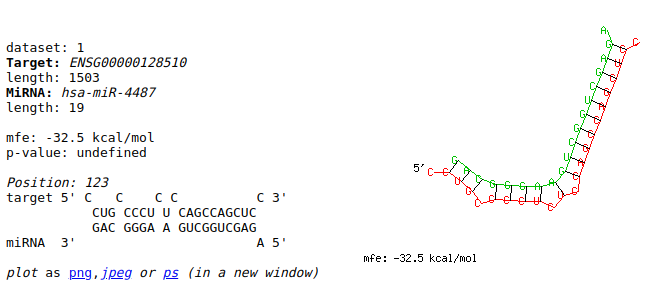** | -32.5 |
| miR4487 | ATP13A4 | **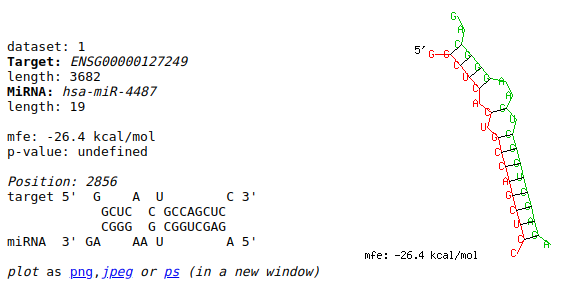** | -26.4 |
| miR4487 | ADAMTSL1 | **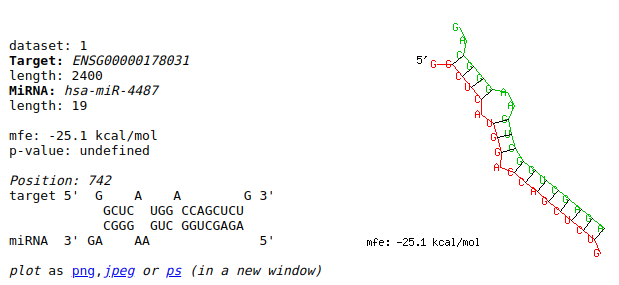** | -25.1 |
| miR4487 | SPOCK2 | **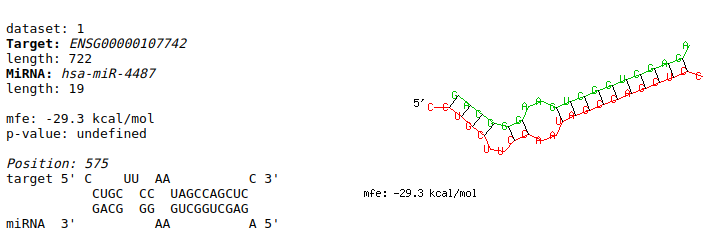** | -29.3 |
| miR4487 | FOXL2 | **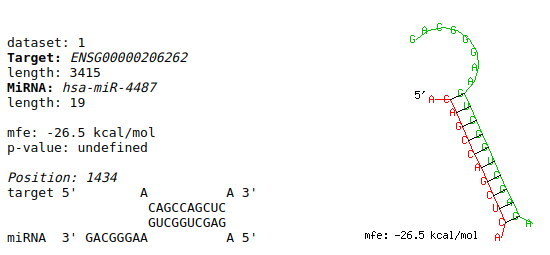** | -26.5 |
| miR6720 | GAL3ST3 | **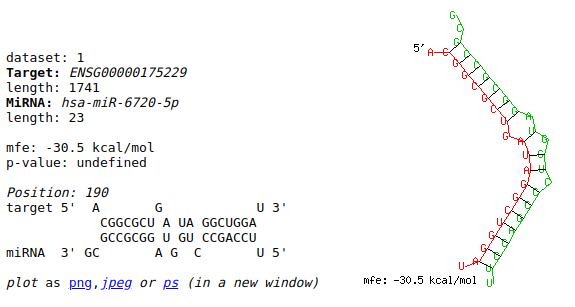** | -30.5 |
| miR6720 | NFE2 | **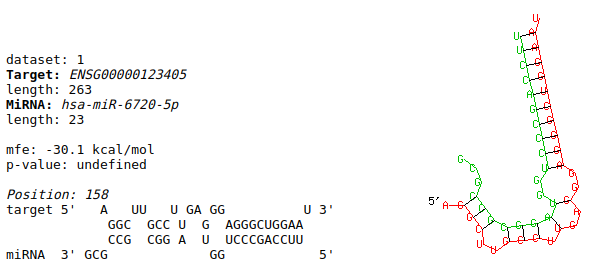** | -30.1 |
| miR6720 | NUPR1 | **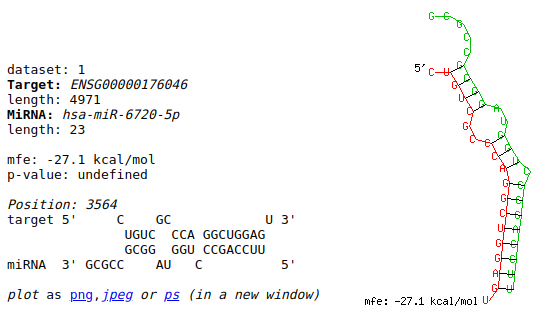** | -27.1 |
| miR6720 | FOXL2 | **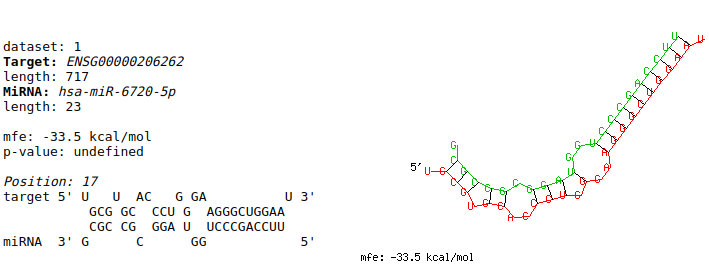** | -33.5 |
| miR6720 | EPHA3 | **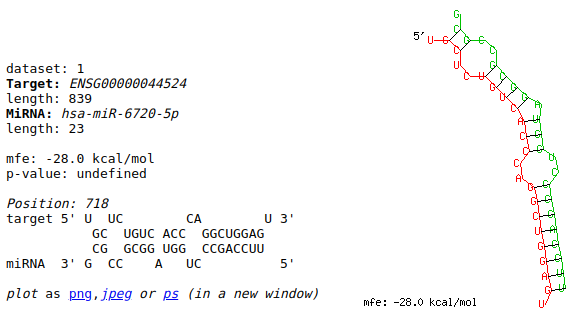** | -28.0 |
| miR6720 | COL25A1 | **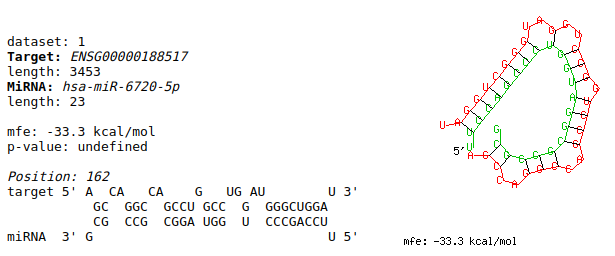** | -33.3 |
| miR-LET7i | C1orf228 | **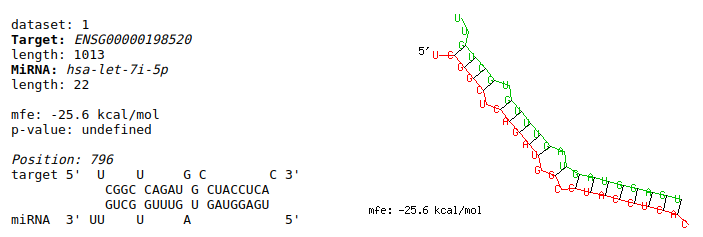** | -25.6 |
