# Supplementary material for: Analysis of H3K4me3-ChIP-Seq and RNA-Seq data to understand the putative role of miRNAs and their target genes in breast cancer cell lines
Source: Genomics Inform. 2021 Jun 30;19(2):e17. doi: 10.5808/gi.21020 (PMC8261273; doi:10.5808/gi.21020)
Supplement: Supplementary Table 9. — Details of reference mapping of RNA-sequencing data [file gi-21020suppl9.docx]

**Supplementary Table 9.** Details of reference mapping of RNA-sequencing data

| Cell line | Total reads | Uniquely  mapped reads | Multi-map reads | Total percentage of reads mapped |
| --- | --- | --- | --- | --- |
| MCF10A-Rep1 | 13,832,765 | 11,731,517 | 1,756,650 | 97.5 |
| MCF10A-Rep2 | 21,837,111 | 18,697,098 | 2,598,911 | 97.52 |
| MCF10A-Rep3 | 17,858,623 | 14,951,792 | 2,209,664 | 96.10 |
| MCF10A-Rep4 | 17,101,361 | 14,451,931 | 1,979,449 | 96.08 |
| MCF7-Rep1 | 21,993,778 | 17,891,772 | 3,570,084 | 97.58 |
| MCF7-Rep2 | 15,784,294 | 12,869,205 | 2,524,867 | 97.53 |
| MCF7-Rep3 | 17,635,434 | 14,186,360 | 2,790,355 | 96.26 |
| MCF7-Rep4 | 23,596,085 | 18,995,772 | 3,682,098 | 96.11 |
| ZR751-Rep1 | 9,069,714 | 7,200,458 | 1,253,622 | 93.21 |
| ZR751-Rep2 | 9,136,186 | 7,244,782 | 1,270,735 | 93.21 |
| ZR751-Rep3 | 22,269,097 | 17,863,576 | 3,117,151 | 94.21 |
| ZR751-Rep4 | 24,382,514 | 19,552,538 | 3,408,990 | 94.17 |
| MB231-Rep1 | 13,392,716 | 11,040,679 | 2,015,824 | 97.49 |
| MB231-Rep2 | 12,522,520 | 10,408,467 | 1,803,868 | 97.52 |
| MB231-Rep3 | 17,610,257 | 14,331,579 | 2,575,268 | 96.01 |
| MB231-Rep4 | 20,218,332 | 16,597,412 | 2,826,409 | 96.07 |
| MB436-Rep1 | 10,080,126 | 7,968,342 | 1,503,548 | 93.97 |
| MB436-Rep2 | 8,925,410 | 7,102,612 | 1,332,821 | 94.51 |
| MB436-Rep3 | 3,354,446 | 2,677,383 | 498,907 | 94.69 |
| MB436-Rep4 | 25,726,444 | 20,688,201 | 3,833,483 | 95.32 |
