# Supplementary material for: Analysis of H3K4me3-ChIP-Seq and RNA-Seq data to understand the putative role of miRNAs and their target genes in breast cancer cell lines
Source: Genomics Inform. 2021 Jun 30;19(2):e17. doi: 10.5808/gi.21020 (PMC8261273; doi:10.5808/gi.21020)
Supplement: Supplementary Table 10. — List of genes upregulated in MCF10A vs. luminal-A cell lines [file gi-21020suppl10.docx]

**Supplementary Table 10.**  List of genes upregulated in MCF10A vs. luminal-A cell lines

| List of genes upregulated in MCF10A vs Luminal-A cell lines | | | | | | pvalue  0.05019422026  0.05038856835  0.05040792644  0.05047420333  0.05053087065  0.05061936518 | padj  0.1366152659  0.1370573316  0.1370810332  0.1372322903  0.1373536346  0.1375592019 | MCF10A.RNA.S  0  0  0  0  0  1606.863014 | MCF10A.RNA.S  0  0  0  0  0.917401664  949.5107223 | MCF10A.RNA.S  0  0  0  0  0  1711.00147 | MCF10A.RNA.S  0  0  0  1.182214954  1.182214954  1024.980365 | MB231.RNA.Seq  2.919735942  2.189801957  0.7299339855  0  1.459867971  100.73089 | MB231.RNA.Seq  2.076666938  4.153333876  0  0  5.191667345  94.48834569 | MB231.RNA.Seq  2.762748711  1.841832474  0  0.9209162369  5.525497422  119.7191108 | MB231.RNA.Seq  1.404229425  1.404229425  0.7021147127  0  4.914802989  98.99817449 | MB436.RNA.Seq  0  0  4.983360138  8.305600229  1.661120046  12606.24003 | MB436.RNA.Seq  0  0  1.754594564  8.772972819  1.754594564  15207.07108 |  |  |
| --- | --- | --- | --- | --- | --- | --- | --- | --- | --- | --- | --- | --- | --- | --- | --- | --- | --- | --- | --- |
| 14195  14204  14207  14210  14214  14217 | Gene ENSG00000259723 ENSG00000274356  ENSG00000253266 ENSG00000258595 ENSG00000213180 ENSG00000138061 | baseMean  0.8753670584  0.9108517847  1.126450835  2.434529142  2.104653174  4983.78595 | log2FoldChange  2.807156948  2.839852898  3.032491051  3.375099446  2.444581085  2.364611572 | lfcSE  1.433462497  1.451384821  1.549968159  1.725578728  1.250142183  1.209710204 | stat  1.958305121  1.956650543  1.956486031  1.955923188  1.955442443  1.95469259 |  |  |  |  |  |  |  |  |  |  |  |  | MB436.RNA.Seq | MB436.RNA.Seq |
|  |  |  |  |  |  |  |  |  |  |  |  |  |  |  |  |  |  | 0.6788559006  0.6788559006  2.036567702  4.073135404  0  11841.96233 | 0.6621677839  0.6621677839  3.31083892  5.959510055  2.648671136  14443.86587 |
| 14224 ENSG00000234787 2.617626309 4.301937294 2.201377026 1.954202867 0.05067723046 0.1376486782 0 0 0 0 0 0 0 0 6.644480183 5.263783691 12.21940621 7.283845623 | | | | | | | | | | | | | | | | | | | |
| 14225  14228  14236  14241  14246  14253  14261  14263  14278 | ENSG00000267272 ENSG00000235579 ENSG00000275661 ENSG00000213265 ENSG00000244144 ENSG00000133169 ENSG00000214870 ENSG00000151062 ENSG00000236383 | 2.829942936  2.022006557  2.289866027  1.218179485  16.66174346  0.9575588768  6.927587594  1.418958653  16.68862109 | 2.910946673  2.255262845  4.123830388  2.414261272  3.138517973  2.806873798  3.267534071  2.599514366  2.335822422 | 1.489619514  1.154460507  2.112603502  1.237279131  1.609360494  1.439786648  1.677563695  1.334717972  1.200777357 | 1.954154498  1.953521001  1.952013421  1.951266462  1.950164668  1.949506756  1.947785399  1.947613219  1.945258552 | 0.05068294869  0.05075789131  0.05093661126  0.05102535642  0.05115649547  0.05123493675  0.05144064653  0.05146126087  0.05174386795 | 0.1376545324  0.1378290084  0.1382365816  0.1384288073  0.1387163955  0.1388803607  0.1393597489  0.1393960467  0.1400141866 | 0  0  0  0  2.530492935  0  1.265246467  0  6.326232337 | 0  0  0  0.917401664  3.669606656  0  2.752204992  0  8.256614976 | 1.982620475  0.9913102377  0  0  0.9913102377  0  0  0.9913102377  0.9913102377 | 0  1.182214954  0  0  3.546644862  0  0  0  2.364429908 | 5.109537899  2.189801957  6.56940587  2.189801957  0  0  0.7299339855  0.7299339855  0.7299339855 | 6.230000815  3.115000407  7.268334284  1.038333469  0  2.076666938  0  2.076666938  0 | 10.13007861  2.762748711  10.13007861  0.9209162369  0  1.841832474  0  0  2.762748711 | 6.319032415  3.510573564  3.510573564  2.808458851  0  0.7021147127  0  1.404229425  1.404229425 | 0  4.983360138  0  1.661120046  33.22240092  0  23.25568064  1.661120046  33.22240092 | 3.509189128  3.509189128  0  1.754594564  42.11026953  3.509189128  17.54594564  3.509189128  63.1654043 | 0.6788559006  1.357711801  0  0.6788559006  52.95076025  2.036567702  20.36567702  1.357711801  37.33707453 | 0  0.6621677839  0  2.648671136  60.91943612  1.324335568  17.21636238  5.297342272  43.70307374 |
| 14281 ENSG00000222328 0.8576586843 2.649261772 1.362064319 1.945034264 0.05177085453 0.1400579066 0 0 0 0 0.7299339855 2.076666938 0 1.404229425 1.661120046 1.754594564 0.6788559006 1.986503352 | | | | | | | | | | | | | | | | | | | |
| 14286  14313  14323  14327  14331  14333  14337  14340  14341 | ENSG00000274215 ENSG00000253489 ENSG00000277249 ENSG00000206838 ENSG00000137877 ENSG00000256417 ENSG00000247287 ENSG00000145536 ENSG00000275897 | 0.849060466  2.165889123  1.273204696  2.645519618  22.43189024  2.302591445  1.646934832  2.915927033  1.662951643 | 2.647848984  4.046114273  2.528643572  2.26393735  2.310668437  4.135723006  2.829634632  3.71782968  3.605849052 | 1.361713952  2.085983989  1.304466573  1.168701876  1.193059374  2.135505914  1.461507125  1.921327229  1.863616937 | 1.944497213  1.939666984  1.938450264  1.937138458  1.936758964  1.936647882  1.936107313  1.935032005  1.934866001 | 0.05183552124  0.05242017449  0.05256831313  0.05272842061  0.05277481432  0.05278840064  0.052854559  0.05298636838  0.05300674123 | 0.1401837717  0.1414974807  0.1417982809  0.1421805228  0.1422758322  0.1422926016  0.1424311841  0.1427565092  0.1428014398 | 0  0  1.265246467  0  2.530492935  0  0  0  0 | 0  0  0  1.834803328  10.0914183  0  0  0.917401664  0 | 0  0  0  0  5.947861426  0  0.9913102377  0  0 | 0  0  0  1.182214954  5.91107477  0  0  0  0 | 1.459867971  5.839471884  2.919735942  2.919735942  0.7299339855  11.67894377  1.459867971  7.299339855  0.7299339855 | 1.038333469  8.306667753  1.038333469  6.230000815  0  4.153333876  1.038333469  5.191667345  0 | 0.9209162369  5.525497422  4.604581185  3.683664948  1.841832474  8.288246132  0.9209162369  11.05099484  0 | 2.106344138  6.319032415  2.106344138  10.53172069  2.106344138  3.510573564  0  10.53172069  0 | 3.322240092  0  0  0  61.4614417  0  6.644480183  0  6.644480183 | 0  0  0  0  54.39243148  0  0  0  5.263783691 | 0.6788559006  0  1.357711801  2.715423602  67.88559006  0  4.073135404  0  1.357711801 | 0.6621677839  0  1.986503352  2.648671136  56.28426163  0  4.635174488  0  5.959510055 |
| 14342 ENSG00000226400 4.613974108 2.103328771 1.087090568 1.934823862 0.05301191391 0.1428054172 1.265246467 0 1.982620475 2.364429908 6.56940587 1.038333469 2.762748711 1.404229425 6.644480183 24.56432389 6.109703105 0.6621677839 | | | | | | | | | | | | | | | | | | | |
| 14352  14366  14369  14376  14377  14383  14386  14395  14404 | ENSG00000101188 ENSG00000126233 ENSG00000284065 ENSG00000207704 ENSG00000283172 ENSG00000261346 ENSG00000226251 ENSG00000235169 ENSG00000213157 | 145.2360556  2.528817582  2.572743157  2.040627399  2.040627399  2.546312059  1.518589186  46.78666939  1.410782054 | 3.117970278  4.255187005  3.463706108  3.19181137  3.19181137  4.26198376  3.476234881  2.093067659  2.613539504 | 1.612681484  2.202728737  1.793199319  1.653925274  1.653925274  2.209014622  1.802391716  1.085738893  1.356309316 | 1.933407376  1.931779857  1.931578979  1.929840133  1.929840133  1.929359687  1.928678905  1.927781783  1.926949459 | 0.05318603327  0.05338668332  0.05341149253  0.05362664909  0.05362664909  0.05368622465  0.05377073669  0.05388227458  0.0539859288 | 0.1431832977  0.1435647324  0.1436114562  0.14410973  0.14410973  0.1442096426  0.1443964977  0.1446156081  0.1447932217 | 24.03968288  0  0  0  0  0  0  16.44820408  0 | 21.10023827  0  0.917401664  0.917401664  0.917401664  0  0  11.92622163  0.917401664 | 23.79144571  0  0  0  0  0  0  19.82620475  0 | 26.00872899  0  0  0  0  0  0  10.63993459  0 | 467.1577507  0  0.7299339855  7.299339855  7.299339855  0  0  5.839471884  2.919735942 | 360.3017138  0  0  6.230000815  6.230000815  0  0  1.038333469  3.115000407 | 442.0397937  0  0  2.762748711  2.762748711  0  0  4.604581185  0.9209162369 | 377.0356007  0  0  5.616917702  5.616917702  0  0.7021147127  1.404229425  3.510573564 | 0  4.983360138  16.61120046  1.661120046  1.661120046  8.305600229  6.644480183  107.972803  0 | 0  5.263783691  5.263783691  0  0  3.509189128  3.509189128  101.7664847  3.509189128 | 1.357711801  9.503982608  2.715423602  0  0  8.146270807  3.394279503  129.661477  2.036567702 | 0  10.59468454  4.635174488  0  0  10.59468454  3.973006704  150.312087  0 |
| 14408 ENSG00000082074 12.15284409 2.655744316 1.378892224 1.925998471 0.05410456474 0.145080125 2.530492935 0 6.939171664 1.182214954 0 0 1.841832474 2.106344138 26.57792073 43.86486409 21.72338882 39.06789925 | | | | | | | | | | | | | | | | | | | |
| 14410  14411  14419  14424  14430  14443  14450  14453  14461 | ENSG00000131050 ENSG00000265489 ENSG00000259711 ENSG00000018869 ENSG00000185499 ENSG00000173391 ENSG00000182103 ENSG00000145626 ENSG00000283342 | 2.499316536  1.092725578  3.53289259  2.466668356  4534.698658  1.5816892  2.124428805  1.49921631  11.35737354 | 4.224836378  3.068189827  2.080047843  4.203510049  2.287582213  3.514928836  4.022792799  3.447693304  2.639022797 | 2.193665514  1.593316887  1.080584806  2.184618483  1.189414863  1.83053302  2.095602486  1.796974317  1.375903506 | 1.925925512  1.925662027  1.924927902  1.924139195  1.923283695  1.920166857  1.91963544  1.918610228  1.918028979 | 0.05411367539  0.05414658827  0.05423837869  0.05433713834  0.05444443115  0.05483682656  0.05490396427  0.0550336802  0.05510733673 | 0.1450854857  0.1451636554  0.1453303573  0.1455432155  0.1457699652  0.1466884161  0.1467968622  0.1470780567  0.1472267639 | 0  0  1.265246467  0  1203.249391  0  0  0  0 | 0  0  0.917401664  0  1358.671864  0  0  0  6.421811648 | 0  0  0  0  1179.659183  0  0  0  0 | 0  0  2.364429908  0  1313.440814  0  0  0  3.546644862 | 0  0.7299339855  0  0  124.8187115  0  6.56940587  0  2.189801957 | 0  0  2.076666938  0  83.06667753  0  5.191667345  1.038333469  1.038333469 | 0  0.9209162369  0.9209162369  0  137.2165193  0  4.604581185  0  0 | 0  0  3.510573564  0  84.95588024  2.106344138  9.127491265  0  0.7021147127 | 8.305600229  1.661120046  9.966720275  4.983360138  9938.481234  1.661120046  0  4.983360138  19.93344055 | 7.018378255  1.754594564  5.263783691  10.52756738  12241.80627  10.52756738  0  5.263783691  42.11026953 | 4.073135404  4.073135404  8.825126708  7.467414906  12260.13756  2.036567702  0  3.394279503  30.54851553 | 10.59468454  3.973006704  7.283845623  6.621677839  14490.87978  2.648671136  0  3.31083892  29.79755028 |
| 14462 ENSG00000233578 2.484392926 2.020830563 1.053611138 1.918004176 0.05511048169 0.1472267639 0 0 0 3.546644862 0.7299339855 4.153333876 2.762748711 3.510573564 3.322240092 1.754594564 4.073135404 5.959510055 | | | | | | | | | | | | | | | | | | | |
| 14475  14479  14486  14491  14507  14513  14516  14523  14525 | ENSG00000182795 ENSG00000139182 ENSG00000234043 ENSG00000260625 ENSG00000170498 ENSG00000244040 ENSG00000231672 ENSG00000274307 ENSG00000257527 | 25.86825393  1371.987863  3.229595029  1.069681041  0.8876099106  4.450430088  4.655383747  1.25226424  2.733295042 | 2.879343873  2.225999192  3.082555501  2.954943996  2.794295617  2.093847935  3.535052688  3.209715707  2.118169493 | 1.50259635  1.16187628  1.610334344  1.543962671  1.461782776  1.095971699  1.851132736  1.681746282  1.109978776 | 1.916245752  1.91586594  1.914233222  1.913870103  1.911566932  1.910494529  1.90967002  1.908561203  1.908297293 | 0.05533382225  0.05538216187  0.05559036255  0.05563675538  0.05593176418  0.05606957019  0.05617571342  0.05631872081  0.05635280267 | 0.147680452  0.14777884  0.1482627128  0.1483352456  0.1489548538  0.1492625814  0.149507179  0.1498226109  0.1498926355 | 2.530492935  559.2389386  1.265246467  0  0  1.265246467  0  0  0 | 8.256614976  284.3945158  0  0  0  0  0  0  0 | 2.973930713  515.4813236  0.9913102377  0  0  1.982620475  0  0  0.9913102377 | 5.91107477  230.531916  0  0  0  2.364429908  2.364429908  0  2.364429908 | 0.7299339855  89.05194623  10.2190758  0.7299339855  0.7299339855  0  0.7299339855  0.7299339855  1.459867971 | 0  26.9966702  2.076666938  0  0  1.038333469  0  0  3.115000407 | 0  74.59421519  11.05099484  2.762748711  2.762748711  1.841832474  0  0.9209162369  3.683664948 | 0  16.85075311  9.829605978  0.7021147127  0.7021147127  2.808458851  0  0  0 | 58.13920161  2787.359437  3.322240092  0  0  6.644480183  8.305600229  4.983360138  8.305600229 | 89.48432275  4279.456141  0  5.263783691  1.754594564  14.03675651  21.05513477  1.754594564  3.509189128 | 54.98732795  3105.765745  0  2.715423602  2.715423602  9.503982608  9.503982608  0.6788559006  4.073135404 | 87.40614748  4494.13275  0  0.6621677839  1.986503352  11.91902011  13.90552346  5.959510055  5.297342272 |
| 14533 ENSG00000223502 2.377023467 4.153202002 2.17853379 1.906420741 0.05659563984 0.1504556902 0 0 0 0 0 0 0 0 4.983360138 8.772972819 8.146270807 6.621677839 | | | | | | | | | | | | | | | | | | | |
| 14553  14556  14558  14560  14565  14566  14567  14569  14570 | ENSG00000170927 ENSG00000261976 ENSG00000259495 ENSG00000207357 ENSG00000158473 ENSG00000189269 ENSG00000264589 ENSG00000276434 ENSG00000270035 | 4.522204995  2.357272351  6.161491836  1.4583051  1.516106237  2.048553065  5.173626986  0.9626788884  0.889272234 | 3.560856737  4.142258599  3.443735596  2.710940063  3.449640399  3.127072026  2.351548264  2.824543058  2.76158245 | 1.871900319  2.177587983  1.810453486  1.425436467  1.814655871  1.645021811  1.237156635  1.486296104  1.453207292 | 1.902268353  1.902223299  1.902139781  1.901831562  1.900988752  1.900930435  1.900768421  1.900390542  1.900336218 | 0.05713608076  0.05714196806  0.05715288301  0.05719317868  0.05730348597  0.05731112504  0.05733235204  0.05738188717  0.05738901134 | 0.1516677471  0.1516680363  0.1516761667  0.1517622567  0.1520027587  0.1520125852  0.1520584486  0.1521689348  0.1521773818 | 0  0  0  0  0  0  1.265246467  0  0 | 0  0  0.917401664  0  0  0.917401664  0  0  0 | 0.9913102377  0  0  0.9913102377  0  0  1.982620475  0  0 | 1.182214954  0  2.364429908  0  0  0  2.364429908  0  0 | 0  0  19.70821761  0.7299339855  0  0  0  0.7299339855  0.7299339855 | 0  0  20.76666938  4.153333876  0  0  1.038333469  3.115000407  2.076666938 | 0  0  14.73465979  5.525497422  0.9209162369  0  0.9209162369  0.9209162369  2.762748711 | 0  0  15.44652368  0.7021147127  0  1.404229425  1.404229425  2.106344138  1.404229425 | 13.28896037  8.305600229  0  0  6.644480183  8.305600229  4.983360138  3.322240092  1.661120046 | 14.03675651  5.263783691  0  0  5.263783691  5.263783691  14.03675651  0  0 | 10.86169441  6.109703105  0  4.073135404  2.715423602  3.394279503  12.89826211  1.357711801  2.036567702 | 13.90552346  8.608181191  0  1.324335568  2.648671136  5.297342272  21.18936909  0  0 |
| 14571 ENSG00000284791 1.743941386 2.80746092 1.477432215 1.900229934 0.05740295162 0.1522039006 0 0 0.9913102377 0 0.7299339855 1.038333469 0 0.7021147127 9.966720275 3.509189128 0.6788559006 3.31083892 | | | | | | | | | | | | | | | | | | | |
| 14572 | ENSG00000207449 | 0.9199803813 | 2.790107614 | 1.468570788 | 1.899879553 | 0.05744892783 | 0.1523153532 | 0 | 0 | 0 | 0 | 0 | 3.115000407 | 2.762748711 | 1.404229425 | 0 | 1.754594564 | 0.6788559006 | 1.324335568 |

| 14575  14589  14593  14600  14601  14604  14617  14639 | ENSG00000128242 ENSG00000250711 ENSG00000246214 ENSG00000248663 ENSG00000253955 ENSG00000255566 ENSG00000225025 ENSG00000218690 | 2.031618738  1.445027159  1.141680033  2.302282781  1.962686195  1.804458758  1.490065734  2.22974893 | 3.954747687  2.541902941  3.158921726  3.280520464  3.112101757  2.173772533  2.609724421  4.081650182 | 2.081883737  1.339908866  1.665480743  1.730888664  1.642096816  1.147189086  1.378010584  2.159838767 | 1.899600644  1.897071514  1.896702642  1.895281038  1.895199922  1.894868562  1.893834816  1.889793925 | 0.05748554764  0.05781850029  0.05786719476  0.05805517898  0.05806592052  0.05810981711  0.05824693881  0.05878552632 | 0.1523810726  0.1531165781  0.1532134123  0.1536275233  0.1536454242  0.1537299907  0.1539557009  0.1551193333 | 0  0  0  1.265246467  0  0  0  0 | 0  0.917401664  0  0  0  0  0  0 | 0  0  0  0  0  0.9913102377  0.9913102377  0 | 0  0  0  0  1.182214954  1.182214954  0  0 | 6.56940587  0.7299339855  3.649669928  0  0  1.459867971  1.459867971  2.919735942 | 6.230000815  0  3.115000407  0  0  2.076666938  0  14.53666857 | 7.367329896  0.9209162369  2.762748711  0  0.9209162369  1.841832474  0.9209162369  3.683664948 | 4.212688276  2.808458851  3.510573564  0.7021147127  2.808458851  4.212688276  0.7021147127  5.616917702 | 0  3.322240092  0  9.966720275  0  1.661120046  4.983360138  0 | 0  5.263783691  0  7.018378255  5.263783691  3.509189128  3.509189128  0 | 0  2.715423602  0  2.715423602  5.430847205  3.394279503  0.6788559006  0 | 0  0.6621677839  0.6621677839  5.959510055  7.946013407  1.324335568  4.635174488  0 |
| --- | --- | --- | --- | --- | --- | --- | --- | --- | --- | --- | --- | --- | --- | --- | --- | --- | --- | --- | --- |
| 14645 ENSG00000230118 0.9536331101 2.846896733 1.50662333 1.88958758 0.05881313953 0.1551217482 0 0 0 0 0 0 1.841832474 2.808458851 1.661120046 1.754594564 2.715423602 0.6621677839 | | | | | | | | | | | | | | | | | | | |
| 14646  14651  14652  14658  14662  14673  14692  14703  14710 | ENSG00000168314 ENSG00000248565 ENSG00000177875 ENSG00000184672 ENSG00000227619 ENSG00000225913 ENSG00000261305 ENSG00000186642 ENSG00000235415 | 1.121545479  4.579105274  2.108995195  2.566503753  2.077665437  1.882514217  1.009038978  7.758200217  1.959667091 | 3.001898227  2.181809993  2.400082011  4.279909814  3.992773921  3.057044895  2.891820667  3.042654412  3.906691971 | 1.58869942  1.154827472  1.270499906  2.267670778  2.116905724  1.621957947  1.537972619  1.619494367  2.080406107 | 1.889531896  1.889295195  1.889084761  1.887359424  1.886136862  1.884786779  1.880280982  1.878768135  1.877850655 | 0.058820593  0.05885228506  0.05888047199  0.05911199874  0.05927651403  0.05945863062  0.06006979374  0.06027615979  0.0604015984 | 0.1551217482  0.155194733  0.1552584654  0.1558051625  0.1561961615  0.1565479211  0.1579632781  0.1583873654  0.1586414517 | 0  0  1.265246467  0  0  0  0  0  0 | 0  0  0  0  0  0  0  0  0 | 0  3.965240951  0  0  0  0  0  2.973930713  0 | 0  1.182214954  1.182214954  0  0  1.182214954  0  2.364429908  0 | 0  1.459867971  6.56940587  0  9.489141812  2.919735942  0.7299339855  21.89801957  7.299339855 | 0  0  3.115000407  0  3.115000407  8.306667753  0  18.69000244  4.153333876 | 1.841832474  3.683664948  6.446413659  0  4.604581185  4.604581185  0.9209162369  33.15298453  6.446413659 | 0.7021147127  2.808458851  1.404229425  0  7.72326184  4.914802989  0  13.34017954  5.616917702 | 1.661120046  14.95008041  3.322240092  1.661120046  0  0  3.322240092  0  0 | 5.263783691  17.54594564  0  7.018378255  0  0  1.754594564  0  0 | 0.6788559006  3.394279503  0.6788559006  10.86169441  0  0  3.394279503  0.6788559006  0 | 3.31083892  5.959510055  1.324335568  11.25685233  0  0.6621677839  1.986503352  0  0 |
| 14714 ENSG00000235529 1.970938822 2.319638703 1.235677672 1.87721989 0.06048796259 0.1588143007 0 0 0.9913102377 1.182214954 3.649669928 5.191667345 3.683664948 4.212688276 1.661120046 1.754594564 0 1.324335568 | | | | | | | | | | | | | | | | | | | |
| 14719  14723  14727  14742  14746  14750  14753  14761  14772 | ENSG00000168899 ENSG00000270933 ENSG00000230286 ENSG00000236875 ENSG00000228620 ENSG00000260167 ENSG00000228140 ENSG00000241158 ENSG00000123360 | 52.27635912  2.124602237  0.952482662  7.262104084  2.193196617  0.8683165063  1.493908625  5.159341089  2.232593512 | 2.29562557  3.183320197  2.865484485  2.302702571  4.064918957  2.824167024  2.706951477  3.333469475  4.053549399 | 1.223510742  1.696922562  1.527918651  1.229547618  2.171033216  1.508487699  1.446060945  1.781980748  2.168468213 | 1.876261067  1.875937222  1.875416916  1.87280471  1.872343052  1.872184324  1.871948403  1.870654034  1.86931465 | 0.06061944035  0.06066390073  0.06073538973  0.06109535735  0.06115915817  0.06118110705  0.06121374226  0.06139304985  0.06157905104 | 0.1591162496  0.1591896899  0.1593339976  0.1601152579  0.1602389852  0.1602534734  0.1602841703  0.1606798656  0.1610551474 | 26.57017582  0  0  3.795739402  0  0  0  0  0 | 5.504409984  0  0  0.917401664  0  0  0  1.834803328  0 | 18.83489452  0  0  0.9913102377  0  0  0.9913102377  0.9913102377  0 | 7.093289725  1.182214954  0  2.364429908  0  0  0  0  0 | 5.109537899  0  3.649669928  1.459867971  8.759207826  2.189801957  2.919735942  12.40887775  0 | 0  0  3.115000407  0  2.076666938  0  6.230000815  15.57500204  0 | 3.683664948  0.9209162369  0.9209162369  1.841832474  11.97191108  2.762748711  1.841832474  15.65557603  0 | 1.404229425  0  1.404229425  0  3.510573564  2.106344138  3.510573564  15.44652368  0 | 107.972803  4.983360138  1.661120046  29.90016083  0  0  0  0  6.644480183 | 164.931889  7.018378255  0  12.28216195  0  0  1.754594564  0  8.772972819 | 114.0477913  5.430847205  0.6788559006  19.68682112  0  2.036567702  0.6788559006  0  4.751991304 | 172.1636238  5.959510055  0  13.90552346  0  1.324335568  0  0  6.621677839 |
| 14775 ENSG00000284082 1.245543859 2.478456152 1.326123547 1.868948152 0.06163002816 0.1611557454 0 0.917401664 0 0 2.919735942 1.038333469 2.762748711 4.212688276 0 1.754594564 0.6788559006 0.6621677839 | | | | | | | | | | | | | | | | | | | |
| 14777  14780  14788  14795  14802  14822  14823  14826  14831 | ENSG00000260253 ENSG00000250254 ENSG00000156140 ENSG00000143839 ENSG00000224732 ENSG00000112486 ENSG00000282432 ENSG00000011590 ENSG00000269397 | 6.205880333  1.274632737  14.27942891  2.255650695  2.225799063  1.083222754  1.931905566  1.758698945  1.348448008 | 2.585446762  2.319046801  3.25920436  4.064635479  4.061502706  3.019027649  3.02597573  2.144302372  2.510408156 | 1.383440438  1.241157585  1.745587392  2.177931918  2.177631025  1.621188291  1.624948097  1.151912817  1.348777138 | 1.868852963  1.868454763  1.867110392  1.866282157  1.865101415  1.862231343  1.86219839  1.861514465  1.861247559 | 0.0616432739  0.06169870992  0.06188617345  0.06200189978  0.06216719028  0.06257048823  0.06257513124  0.06267155985  0.06270922495 | 0.1611685651  0.1612807617  0.1616911729  0.1619116533  0.1622638425  0.1630961282  0.163097227  0.1633155075  0.1633585669 | 3.795739402  1.265246467  0  0  0  0  0  0  0 | 0  0  2.752204992  0  0  0  0  0.917401664  0 | 1.982620475  0  0.9913102377  0  0  0  0  0.9913102377  0.9913102377 | 0  0  4.728859816  0  0  0  1.182214954  0  0 | 1.459867971  0.7299339855  0  0  0  0.7299339855  0  2.189801957  2.189801957 | 0  2.076666938  0  0  0  0  0  4.153333876  4.153333876 | 0  2.762748711  0  0  0  5.525497422  0  0.9209162369  2.762748711 | 2.106344138  0.7021147127  0  0  0  0  2.106344138  3.510573564  1.404229425 | 9.966720275  3.322240092  38.20576106  9.966720275  6.644480183  1.661120046  8.305600229  3.322240092  3.322240092 | 26.31891846  1.754594564  42.11026953  7.018378255  5.263783691  1.754594564  3.509189128  1.754594564  0 | 14.93482981  1.357711801  45.48334534  6.109703105  9.503982608  0.6788559006  5.430847205  1.357711801  1.357711801 | 13.90552346  1.324335568  37.0813959  3.973006704  5.297342272  2.648671136  2.648671136  1.986503352  0 |
| 14836 ENSG00000250790 3.197869362 2.033008536 1.09270504 1.860528195 0.06281083317 0.1635675959 1.265246467 0.917401664 1.982620475 0 5.839471884 8.306667753 8.288246132 5.616917702 0 3.509189128 0 2.648671136 | | | | | | | | | | | | | | | | | | | |
| 14843  14844  14870  14881  14884  14886  14894  14896  14902 | ENSG00000065609 ENSG00000283345 ENSG00000186417 ENSG00000234593 ENSG00000230992 ENSG00000228126 ENSG00000284293 ENSG00000232128 ENSG00000257480 | 2.067728808  2.174297317  9.215808671  0.8058660318  0.9189697433  1.524405643  1.353626385  1.847796196  1.414210633 | 3.980717392  2.506059549  3.159586198  2.668084302  2.789695299  2.762844948  2.482006074  3.821835197  2.595076713 | 2.140621386  1.347712939  1.702596954  1.439761498  1.505444076  1.491154522  1.340427481  2.064344658  1.402778494 | 1.859608345  1.859490605  1.855745243  1.85314325  1.853071358  1.8528227  1.851652632  1.85135519  1.849954732 | 0.06294095781  0.06295762973  0.06348987869  0.06386182919  0.06387213146  0.06390777543  0.06407571995  0.06411847108  0.06432007435 | 0.1638296776  0.1638509952  0.1649584037  0.1657910073  0.1657993298  0.1658657063  0.1662122627  0.1663008278  0.1667565476 | 0  0  1.265246467  0  0  0  0  0  1.265246467 | 0  0.917401664  0  0  0  0  0  0  0 | 0  0  0  0  0  0  0  0  0 | 0  1.182214954  4.728859816  0  0  1.182214954  1.182214954  0  0 | 8.759207826  2.919735942  44.52597312  2.189801957  0.7299339855  0  0.7299339855  5.839471884  4.379603913 | 3.115000407  0  12.46000163  0  3.115000407  3.115000407  3.115000407  5.191667345  3.115000407 | 10.13007861  0.9209162369  34.994817  0.9209162369  2.762748711  0.9209162369  0  5.525497422  0 | 2.808458851  1.404229425  11.93595012  2.106344138  0  0.7021147127  1.404229425  5.616917702  2.808458851 | 0  6.644480183  0  0  0  1.661120046  3.322240092  0  1.661120046 | 0  0  0  1.754594564  1.754594564  0  1.754594564  0  1.754594564 | 0  7.467414906  0.6788559006  2.036567702  0.6788559006  4.751991304  4.073135404  0  0 | 0  4.635174488  0  0.6621677839  1.986503352  5.959510055  0.6621677839  0  1.986503352 |
| 14913 ENSG00000267472 1.324981867 3.27717889 1.773195476 1.848176884 0.06457675825 0.1672985352 0 0 0 0 0 0 0 0.7021147127 4.983360138 3.509189128 3.394279503 3.31083892 | | | | | | | | | | | | | | | | | | | |
| 14916  14927  14928  14929  14930  14931  14934  14941  14943 | ENSG00000241101 ENSG00000168481 ENSG00000236717 ENSG00000279535 ENSG00000280079 ENSG00000117834 ENSG00000283667 ENSG00000278702 ENSG00000227002 | 1.9717731  2.440418903  2.381765207  8.267519279  1.890747419  8.336451058  6.603334189  1.455618994  2.67073508 | 3.911316358  2.013346502  4.172514356  3.476637005  3.851538994  2.901077796  2.155610907  3.414069745  2.106952542 | 2.116554663  1.090272219  2.259655845  1.883001549  2.086220819  1.571440211  1.168179003  1.851300845  1.142721757 | 1.847963781  1.846645698  1.846526481  1.846327215  1.846179925  1.846126742  1.845274484  1.844146376  1.843801896 | 0.0646075826  0.06479850655  0.06481579797  0.06484470847  0.06486608483  0.06487380474  0.06499762027  0.06516181077  0.06521201634 | 0.167333509  0.1677155691  0.1677490859  0.1678126674  0.167856744  0.1678654776  0.1681520731  0.1684978622  0.1685938337 | 0  1.265246467  0  1.265246467  0  1.265246467  3.795739402  0  1.265246467 | 0  0  0  0  0  2.752204992  0  0  0 | 0  1.982620475  0  2.973930713  0  0.9913102377  2.973930713  0  0.9913102377 | 0  0  0  0  0  1.182214954  1.182214954  0  1.182214954 | 6.56940587  1.459867971  0  0  4.379603913  0  0.7299339855  0  0.7299339855 | 4.153333876  1.038333469  0  0  8.306667753  0  0  0  0 | 10.13007861  0.9209162369  0  0  3.683664948  0  1.841832474  0  2.762748711 | 2.808458851  2.106344138  0  0  6.319032415  0  2.106344138  0.7021147127  7.72326184 | 0  4.983360138  6.644480183  23.25568064  0  28.23904078  13.28896037  8.305600229  8.305600229 | 0  3.509189128  1.754594564  22.80972933  0  24.56432389  29.82810758  1.754594564  1.754594564 | 0  4.073135404  12.89826211  23.08110062  0  26.47538012  12.89826211  3.394279503  2.036567702 | 0  7.946013407  7.283845623  25.82454357  0  14.56769125  10.59468454  3.31083892  5.297342272 |
| 14945 ENSG00000228930 1.88619374 2.979759541 1.616256127 1.843618404 0.06523877201 0.1686517201 0 0 0 1.182214954 0 0 0 1.404229425 4.983360138 7.018378255 4.073135404 3.973006704 | | | | | | | | | | | | | | | | | | | |
| 14946  14947  14948  14949  14950  14958  14959  14970  14985 | ENSG00000171840 ENSG00000164176 ENSG00000234350 ENSG00000169059 ENSG00000256802 ENSG00000113600 ENSG00000197272 ENSG00000152208 ENSG00000231128 | 2.534223809  3.659614794  1.791922133  1.475243131  1.923283026  5.940619619  2.167282546  4.150223907  1.092516826 | 2.081609892  2.67913504  2.12782001  3.433391882  3.097523435  2.530916082  4.021260853  3.427413267  3.006651747 | 1.129201749  1.453365833  1.154311042  1.862788921  1.68063201  1.374777422  2.184295442  1.863406498  1.637074185 | 1.843434881  1.843400319  1.843367976  1.843145964  1.843070593  1.840964247  1.840987613  1.839326669  1.836600793 | 0.06526554129  0.06527058353  0.06527530237  0.06530770185  0.06531870423  0.06562679819  0.06562337393  0.06586715271  0.06626885019 | 0.1687096339  0.1687113799  0.1687122897  0.1687847388  0.1688018821  0.1694960457  0.1694960457  0.1699828658  0.1708459247 | 0  1.265246467  1.265246467  0  0  2.530492935  0  1.265246467  0 | 0.917401664  0  0  0  0  0  0  0  0 | 1.982620475  1.982620475  0.9913102377  0  0.9913102377  1.982620475  0  0.9913102377  0 | 0  0  0  0  0  1.182214954  0  0  0 | 4.379603913  13.86874572  2.919735942  0  8.759207826  0.7299339855  0  0  2.919735942 | 0  6.230000815  3.115000407  0  3.115000407  0  0  0  5.191667345 | 4.604581185  10.13007861  1.841832474  0.9209162369  6.446413659  0  0  0  0.9209162369 | 0.7021147127  7.72326184  1.404229425  0  2.106344138  0.7021147127  0  0  0 | 8.305600229  0  0  8.305600229  1.661120046  13.28896037  4.983360138  14.95008041  1.661120046 | 3.509189128  0  5.263783691  1.754594564  0  22.80972933  7.018378255  10.52756738  1.754594564 | 2.036567702  2.715423602  2.715423602  4.073135404  0  10.18283851  4.073135404  8.825126708  0 | 3.973006704  0  1.986503352  2.648671136  0  17.87853017  9.932516759  13.24335568  0.6621677839 |
| 14986 ENSG00000234493 2.319502044 4.126795596 2.246940833 1.83662851 0.06626475549 0.1708459247 0 0 0 0 0 0 0 0 1.661120046 8.772972819 7.467414906 9.932516759 | | | | | | | | | | | | | | | | | | | |
| 14988 | ENSG00000255741 | 1.542894517 | 2.667490724 | 1.452532933 | 1.836440788 | 0.06629249187 | 0.1708840688 | 1.265246467 | 0 | 0 | 0 | 1.459867971 | 0 | 0 | 0.7021147127 | 6.644480183 | 1.754594564 | 2.715423602 | 3.973006704 |

| 14990  14993  14994  14999  15004  15007  15013  15017 | ENSG00000239405 ENSG00000263574 ENSG00000168913 ENSG00000170409 ENSG00000273786 ENSG00000272716 ENSG00000278962 ENSG00000237751 | 4.107479499  2.132377728  2.059911729  1.641866267  1.482894612  2.242123155  1.799071406  0.8874624225 | 3.41849471  3.991702061  3.144020132  2.786763874  2.616232584  2.571268715  3.782484306  2.7323464 | 1.861602458  2.173918107  1.71237072  1.518144505  1.426285562  1.402000518  2.06346817  1.49115949 | 1.836318327  1.836178671  1.836062773  1.835638086  1.83429788  1.833999832  1.833071312  1.832363619 | 0.06631059088  0.0663312361  0.06634837325  0.06641120042  0.06660978863  0.06665401909  0.0667919667  0.06689726452 | 0.1708961216  0.1709269197  0.1709596772  0.1710645195  0.1715188739  0.1715956199  0.1718848753  0.1721099963 | 0  0  0  1.265246467  0  0  0  0 | 0  0  0  0  0.917401664  0.917401664  0  0 | 0.9913102377  0  0.9913102377  0  0  0  0  0 | 1.182214954  0  0  0  0  1.182214954  0  0 | 0  0  0  1.459867971  0  6.56940587  5.839471884  2.189801957 | 0  0  0  6.230000815  0  5.191667345  6.230000815  0 | 0  0  0.9209162369  0  0.9209162369  3.683664948  4.604581185  0.9209162369 | 0  0  0  4.914802989  1.404229425  7.021147127  4.914802989  0.7021147127 | 9.966720275  8.305600229  3.322240092  1.661120046  4.983360138  1.661120046  0  0 | 12.28216195  5.263783691  8.772972819  3.509189128  3.509189128  0  0  3.509189128 | 14.93482981  4.073135404  4.751991304  0  4.073135404  0.6788559006  0  0.6788559006 | 9.932516759  7.946013407  5.959510055  0.6621677839  1.986503352  0  0  2.648671136 |
| --- | --- | --- | --- | --- | --- | --- | --- | --- | --- | --- | --- | --- | --- | --- | --- | --- | --- | --- | --- |
| 15018 ENSG00000283283 0.9276272783 2.877290908 1.570421899 1.832177016 0.06692505203 0.1721585582 0 0 0 0 2.189801957 4.153333876 0 2.106344138 0 0 1.357711801 1.324335568 | | | | | | | | | | | | | | | | | | | |
| 15019  15027  15028  15033  15041  15045  15065  15078  15079 | ENSG00000283167 ENSG00000226051 ENSG00000114547 ENSG00000257398 ENSG00000259469 ENSG00000228146 ENSG00000234998 ENSG00000175877 ENSG00000219758 | 0.9276272783  2.516505791  1.606330326  2.083200363  0.8423815508  0.8328736235  3.147782907  2.056085088  1.331542152 | 2.877290908  2.703817695  2.799292253  3.968653174  2.703645793  2.693850466  3.003605507  3.942604439  2.502277314 | 1.570421899  1.476983417  1.529292337  2.168856004  1.478369323  1.473259091  1.644949666  2.161478457  1.371863821 | 1.832177016  1.830635106  1.830449407  1.829837097  1.828802689  1.828497433  1.825955875  1.824031337  1.823998326 | 0.06692505203  0.06715502495  0.06718276543  0.06727430178  0.06742917205  0.06747493064  0.06785690862  0.06814733484  0.06815232528 | 0.1721585582  0.1726581745  0.1726835302  0.1728958058  0.173201653  0.1732731104  0.1740226794  0.1746168113  0.1746180176 | 0  0  0  0  0  0  1.265246467  0  0 | 0  0.917401664  0  0  0  0  0  0  0.917401664 | 0  0.9913102377  0.9913102377  0  0  0  0.9913102377  0  0 | 0  0  0  0  0  0  0  0  0 | 2.189801957  0.7299339855  2.189801957  0  0  2.919735942  0  0  0.7299339855 | 4.153333876  1.038333469  0  0  1.038333469  1.038333469  1.038333469  0  4.153333876 | 0  0  0  0  1.841832474  0.9209162369  0  0  2.762748711 | 2.106344138  0  1.404229425  0  2.808458851  0  0  0  2.106344138 | 0  4.983360138  6.644480183  4.983360138  0  0  8.305600229  6.644480183  3.322240092 | 0  8.772972819  0  5.263783691  1.754594564  1.754594564  8.772972819  5.263783691  0 | 1.357711801  7.467414906  4.073135404  7.467414906  0.6788559006  2.036567702  7.467414906  7.467414906  0 | 1.324335568  5.297342272  3.973006704  7.283845623  1.986503352  1.324335568  9.932516759  5.297342272  1.986503352 |
| 15083 ENSG00000277342 4.317830159 2.913096931 1.597854399 1.823130401 0.06828364312 0.1749080788 0 0.917401664 0 2.364429908 5.839471884 12.46000163 9.209162369 20.36132667 0 0 0 0.6621677839 | | | | | | | | | | | | | | | | | | | |
| 15086  15092  15093  15097  15102  15112  15131  15136  15146 | ENSG00000249491 ENSG00000222036 ENSG00000247324 ENSG00000144229 ENSG00000116176 ENSG00000224384 ENSG00000183668 ENSG00000156925 ENSG00000212533 | 0.8720628194  0.7803905783  0.9385864334  2.158216263  1.774865322  0.8358278647  11.08971281  1.746692265  1.345219327 | 2.597581288  2.660513701  2.737465108  4.00530556  3.764696248  2.635380135  3.444091204  3.739595336  2.535275297 | 1.424965078  1.460518702  1.502915293  2.200375427  2.06871527  1.449054081  1.897735494  2.061372962  1.398696769 | 1.822908735  1.821622481  1.821436724  1.820282807  1.819823299  1.818689978  1.814842593  1.814128449  1.812598237 | 0.06831721466  0.06851228735  0.06854049701  0.06871594824  0.06878591837  0.06895874158  0.06954810277  0.06965795254  0.0698938099 | 0.1749556952  0.175389095  0.1754496854  0.1758405524  0.1759729808  0.1762983709  0.1775818486  0.1778035806  0.1782878216 | 0  0  0  0  0  0  0  0  0 | 0  0  0  0  0  0  4.58700832  0  0 | 0  0  0  0  0  0  0  0  0.9913102377 | 0  0  0  0  0  0  1.182214954  0  0 | 0.7299339855  2.919735942  0  0  4.379603913  0  46.71577507  5.109537899  1.459867971 | 1.038333469  2.076666938  2.076666938  0  4.153333876  1.038333469  11.42166816  5.191667345  4.153333876 | 0.9209162369  0.9209162369  0  0  6.446413659  0.9209162369  48.80856056  6.446413659  0 | 0  2.106344138  2.106344138  0  6.319032415  1.404229425  20.36132667  4.212688276  4.212688276 | 3.322240092  0  3.322240092  11.62784032  0  3.322240092  0  0  3.322240092 | 1.754594564  0  1.754594564  3.509189128  0  0  0  0  0 | 2.036567702  0.6788559006  0.6788559006  6.788559006  0  1.357711801  0  0  0.6788559006 | 0.6621677839  0.6621677839  1.324335568  3.973006704  0  1.986503352  0  0  1.324335568 |
| 15148 ENSG00000230002 6.226431898 2.017419769 1.113251809 1.812186383 0.06995740237 0.1784204228 0 3.669606656 0.9913102377 3.546644862 10.94900978 19.72833591 11.05099484 20.36132667 0 1.754594564 0.6788559006 1.986503352 | | | | | | | | | | | | | | | | | | | |
| 15153  15163  15169  15187  15192  15194  15200  15204  15213 | ENSG00000235787 ENSG00000269313 ENSG00000124575 ENSG00000188984 ENSG00000200222 ENSG00000261787 ENSG00000095713 ENSG00000272043 ENSG00000188763 | 1.182319701  37.1248438  0.7008018827  2.047741029  1.75380191  1.734589223  1.306583731  0.847871071  5.713945375 | 2.328528028  2.127190569  2.533208934  3.937056963  3.748918061  3.73039463  3.224791369  2.745448027  2.920424515 | 1.285521342  1.175635907  1.401152529  2.180565388  2.078091407  2.068077284  1.788845166  1.523690794  1.621786619 | 1.811349178  1.809395711  1.807946588  1.805521166  1.804019808  1.803798465  1.802722466  1.801840661  1.800745228 | 0.07008681758  0.07038954897  0.07061481388  0.07099316642  0.07122820176  0.07126290663  0.07143181203  0.07157047842  0.07174304546 | 0.1786975646  0.1793337631  0.1798538687  0.1806032123  0.1811414939  0.1812139775  0.181563688  0.1818699499  0.1821989457 | 0  7.591478805  0  0  0  0  0  0  1.265246467 | 0  11.00881997  0  0  0  0  0  0  1.834803328 | 0  12.88703309  0  0  0  0  0  0  0 | 1.182214954  14.18657945  0  0  0  0  0  0  1.182214954 | 2.919735942  2.919735942  1.459867971  0  6.56940587  4.379603913  0.7299339855  0.7299339855  18.24834964 | 0  0  1.038333469  0  3.115000407  4.153333876  0  1.038333469  17.65166897 | 1.841832474  1.841832474  1.841832474  0  6.446413659  7.367329896  0  0  10.13007861 | 2.808458851  2.106344138  1.404229425  0  4.914802989  4.914802989  0  0.7021147127  18.25498253 | 1.661120046  86.37824239  0  6.644480183  0  0  4.983360138  1.661120046  0 | 1.754594564  92.99351188  0  5.263783691  0  0  5.263783691  0  0 | 1.357711801  95.71868198  0.6788559006  3.394279503  0  0  2.715423602  3.394279503  0 | 0.6621677839  117.8658655  1.986503352  9.270348975  0  0  1.986503352  2.648671136  0 |
| 15222 ENSG00000227534 2.021066333 3.92384589 2.181339918 1.798823676 0.07204657689 0.1828616146 0 0 0 0 0 0 0 0 4.983360138 5.263783691 4.073135404 9.932516759 | | | | | | | | | | | | | | | | | | | |
| 15224  15231  15235  15237  15239  15240  15245  15250  15279 | ENSG00000268649 ENSG00000225472 ENSG00000137766 ENSG00000278328 ENSG00000229924 ENSG00000210678 ENSG00000114771 ENSG00000278287 ENSG00000166509 | 2.425470269  0.8760209221  2.048166413  1.680291432  3.708249204  2.18358517  1.983805544  1.049309708  2.598059324 | 3.435964291  2.725068272  3.927994223  2.120905355  2.641955403  2.542571291  3.879666336  2.99819866  3.507877012 | 1.910393458  1.515908331  2.185704731  1.180581634  1.470731559  1.415492655  2.160311626  1.669907063  1.958726324 | 1.798563681  1.797647138  1.797129396  1.796491911  1.796354601  1.796244779  1.795882728  1.795428456  1.790896957 | 0.07208772677  0.07223294341  0.07231507997  0.07241631809  0.07243813922  0.07245559591  0.07251316997  0.07258546201  0.07330983216 | 0.1829420207  0.1832262996  0.183386486  0.1836070645  0.1836503385  0.1836825425  0.1837561539  0.1838911033  0.1853737395 | 0  0  0  0  1.265246467  0  0  0  0 | 0  0  0  0.917401664  0  0.917401664  0  0  0 | 0  0  0  0.9913102377  1.982620475  0  0  0  0.9913102377 | 1.182214954  0  0  0  0  1.182214954  0  0  0 | 12.40887775  1.459867971  0  3.649669928  0.7299339855  3.649669928  0  2.919735942  0 | 4.153333876  1.038333469  0  3.115000407  0  5.191667345  0  0  0 | 6.446413659  0  0  1.841832474  0  5.525497422  0  0  0 | 4.914802989  0.7021147127  0  4.212688276  0.7021147127  7.021147127  0  2.106344138  0 | 0  3.322240092  3.322240092  1.661120046  6.644480183  0  4.983360138  0  6.644480183 | 0  0  10.52756738  1.754594564  15.79135107  0  8.772972819  3.509189128  8.772972819 | 0  0.6788559006  5.430847205  1.357711801  6.788559006  2.715423602  4.751991304  3.394279503  8.146270807 | 0  3.31083892  5.297342272  0.6621677839  10.59468454  0  5.297342272  0.6621677839  6.621677839 |
| 15286 ENSG00000249006 1.331900724 2.507736882 1.401107906 1.789824232 0.07348217235 0.185724436 0 0 0 1.182214954 1.459867971 1.038333469 1.841832474 4.914802989 0 3.509189128 2.036567702 0 | | | | | | | | | | | | | | | | | | | |
| 15288  15292  15294  15306  15310  15317  15318  15321  15326 | ENSG00000147381 ENSG00000224678 ENSG00000189367 ENSG00000279595 ENSG00000150625 ENSG00000241157 ENSG00000179934 ENSG00000196620 ENSG00000149654 | 1.290054547  1.839349857  2.324060078  1.091970495  11.14577718  2.649548337  2.115323829  1.877393559  1.700797426 | 3.265214543  2.335332321  3.380939817  2.241416806  2.801108011  2.814123597  3.996645308  2.978943073  3.699368008 | 1.824883502  1.305947538  1.89080645  1.255019214  1.569230164  1.577398015  2.240538173  1.670401923  2.075624927 | 1.789272872  1.788228282  1.788094078  1.785962145  1.7850205  1.78402887  1.78378809  1.783369039  1.782291183 | 0.07357088082  0.07373918521  0.07376083084  0.07410538766  0.07425799184  0.07441897416  0.07445810563  0.07452624973  0.07470175974 | 0.1859121578  0.1863009038  0.1863190389  0.1870548577  0.1873910852  0.1877115014  0.1877856852  0.1879330108  0.1883141386 | 0  0  0  0  2.530492935  0  0  0  0 | 0  0.917401664  0  0  0.917401664  0.917401664  0  0  0 | 0  0.9913102377  0.9913102377  0.9913102377  1.982620475  0  0  0  0 | 0  0  0  0  3.546644862  1.182214954  0  1.182214954  0 | 0.7299339855  1.459867971  5.109537899  0.7299339855  0  5.109537899  0  0.7299339855  2.919735942 | 0  1.038333469  4.153333876  2.076666938  0  9.345001222  0  0  7.268334284 | 0  1.841832474  9.209162369  2.762748711  0  11.05099484  0  0  4.604581185 | 0  1.404229425  8.425376553  2.106344138  0  3.510573564  0  0  5.616917702 | 4.983360138  0  0  0  43.18912119  0  1.661120046  6.644480183  0 | 1.754594564  1.754594564  0  1.754594564  19.3005402  0  7.018378255  5.263783691  0 | 2.715423602  3.394279503  0  1.357711801  28.51194782  0.6788559006  6.109703105  4.073135404  0 | 5.297342272  9.270348975  0  1.324335568  33.77055698  0  10.59468454  4.635174488  0 |
| 15330 ENSG00000234362 1.703270526 3.705635947 2.080959409 1.780734372 0.0749558551 0.188884987 0 0 0 0 2.919735942 5.191667345 4.604581185 7.72326184 0 0 0 0 | | | | | | | | | | | | | | | | | | | |
| 15333  15335  15336  15337  15341  15345  15352  15354  15357 | ENSG00000112333 ENSG00000212396 ENSG00000254480 ENSG00000179219 ENSG00000221676 ENSG00000215386 ENSG00000272727 ENSG00000168830 ENSG00000284746 | 1.347529531  1.997813548  1.274102501  2.392372974  2.125075611  19.33931665  2.19800014  2.057430768  1.336699844 | 3.372296551  2.382616408  2.491948882  3.417280525  2.500695526  2.587266971  4.029905109  3.918163589  2.526050428 | 1.893899458  1.338509993  1.400149928  1.920375119  1.405787404  1.454843979  2.267702193  2.20558019  1.422175317 | 1.780610126  1.780051266  1.779772889  1.779485941  1.778857542  1.778381055  1.77708745  1.776477503  1.776187786 | 0.07497616447  0.07506757111  0.07511313622  0.07516012786  0.07526312046  0.07534129203  0.07555385269  0.07565424667  0.07570197067 | 0.1889195927  0.1891252435  0.1892277007  0.189333738  0.1895437494  0.1897111575  0.1901395974  0.1903674495  0.1904503247 | 0  0  0  0  0  7.591478805  0  0  0 | 0  1.834803328  0  0.917401664  0.917401664  2.752204992  0  0  0 | 0  0  0.9913102377  0  0  3.965240951  0  0  0 | 0  0  0  0  1.182214954  3.546644862  0  0  1.182214954 | 0  0  3.649669928  3.649669928  3.649669928  0  0  0  1.459867971 | 0  3.115000407  1.038333469  11.42166816  5.191667345  1.038333469  0  0  6.230000815 | 0  2.762748711  4.604581185  9.209162369  5.525497422  0  0  0  0 | 0.7021147127  4.914802989  0  3.510573564  6.319032415  0  0  0  2.808458851 | 1.661120046  6.644480183  1.661120046  0  0  44.85024124  1.661120046  11.62784032  1.661120046 | 1.754594564  0  0  0  0  52.63783691  14.03675651  7.018378255  0 | 5.430847205  2.715423602  1.357711801  0  2.715423602  46.16220124  3.394279503  3.394279503  2.036567702 | 6.621677839  1.986503352  1.986503352  0  0  69.52761731  7.283845623  2.648671136  0.6621677839 |
| 15358 ENSG00000198019 8.41247999 2.691277282 1.515344541 1.776016747 0.07573015682 0.1904964261 1.265246467 0.917401664 4.956551189 0 0 0 1.841832474 0 21.5945606 28.07351302 23.75995652 18.54069795 | | | | | | | | | | | | | | | | | | | |
| 15359 | ENSG00000277200 | 1.762250601 | 3.754673365 | 2.114072572 | 1.776038067 | 0.07572664294 | 0.1904964261 | 0 | 0 | 0 | 0 | 6.56940587 | 2.076666938 | 8.288246132 | 4.212688276 | 0 | 0 | 0 | 0 |

| 15360  15361  15363  15364  15367  15377  15380  15384 | ENSG00000229808 ENSG00000257097 ENSG00000111452 ENSG00000259240 ENSG00000258837 ENSG00000100678 ENSG00000251689 ENSG00000277186 | 1.095665158  1.681252938  1.674585791  1.674585791  1.895551332  0.9638330819  1.673246745  1.775716665 | 2.37472874  2.090610835  3.67937646  3.67937646  3.818495171  2.914504194  3.682624704  2.099356867 | 1.337302085  1.177357954  2.072506814  2.072506814  2.151838075  1.643805884  2.077429036  1.184735111 | 1.775760889  1.775679884  1.775326592  1.775326592  1.774527189  1.77302212  1.772683755  1.772005276 | 0.07577233668  0.07578569482  0.07584397706  0.07584397706  0.07597598881  0.07622504151  0.07628112448  0.07639368148 | 0.190590119  0.1906113091  0.1907206492  0.1907206492  0.1910028844  0.191516842  0.191620367  0.1918532166 | 0  1.265246467  0  0  0  0  0  2.530492935 | 0.917401664  0  0  0  0  0  0  0 | 0  0.9913102377  0  0  0  0  0  0 | 0  0  0  0  0  0  0  0 | 1.459867971  2.189801957  6.56940587  6.56940587  0  2.919735942  8.029273841  0.7299339855 | 1.038333469  2.076666938  5.191667345  5.191667345  0  3.115000407  4.153333876  1.038333469 | 0.9209162369  2.762748711  5.525497422  5.525497422  0  2.762748711  3.683664948  3.683664948 | 2.106344138  3.510573564  2.808458851  2.808458851  0  2.106344138  4.212688276  2.808458851 | 0  3.322240092  0  0  4.983360138  0  0  1.661120046 | 0  0  0  0  7.018378255  0  0  3.509189128 | 3.394279503  3.394279503  0  0  6.109703105  0  0  2.036567702 | 3.31083892  0.6621677839  0  0  4.635174488  0.6621677839  0  3.31083892 |
| --- | --- | --- | --- | --- | --- | --- | --- | --- | --- | --- | --- | --- | --- | --- | --- | --- | --- | --- | --- |
| 15389 ENSG00000175302 9.40832911 3.315838933 1.87228398 1.77101282 0.07655856983 0.1922048441 0 1.834803328 0 3.546644862 0 0 0 0 23.25568064 33.33729671 24.43881242 26.48671136 | | | | | | | | | | | | | | | | | | | |
| 15397  15406  15412  15416  15418  15421  15425  15428  15430 | ENSG00000133116 ENSG00000223738 ENSG00000231651 ENSG00000109610 ENSG00000255452 ENSG00000164125 ENSG00000163638 ENSG00000173826 ENSG00000166589 | 1.420562542  1.361866108  2.930707772  1.130159784  1.975575422  900.7535471  81.41508184  2.23953794  1.120149072 | 3.370308395  2.341448857  2.209325934  3.049614966  3.86096125  2.688868129  2.990605025  3.323356511  3.1155744 | 1.904431736  1.324544835  1.250533561  1.726729067  2.186459873  1.522881377  1.693897868  1.883244612  1.765566163 | 1.769718668  1.767738468  1.76670663  1.766122447  1.765850496  1.765645157  1.76551673  1.764697209  1.764631915 | 0.0767740185  0.07710463581  0.07727737248  0.0773753083  0.07742093432  0.07745539899  0.07747696105  0.07761466791  0.07762564802 | 0.1926330825  0.1933621709  0.193730715  0.1939150906  0.1939916854  0.1940501611  0.1940565569  0.1943636696  0.1943659696 | 0  0  2.530492935  0  0  292.271934  10.12197174  0  0 | 0  0  0.917401664  0  0  87.15315808  14.67842662  0  0 | 0  0  0  0  0  288.4712792  18.83489452  0.9913102377  0 | 0  1.182214954  0  0  0  109.9459907  14.18657945  0  0 | 0.7299339855  0.7299339855  2.189801957  1.459867971  0  6.56940587  216.0604597  8.759207826  0.7299339855 | 0  1.038333469  1.038333469  0  0  0  235.7016975  8.306667753  4.153333876 | 0  2.762748711  0  0  0  3.683664948  253.2519652  4.604581185  3.683664948 | 0  0.7021147127  1.404229425  0  0  4.212688276  214.1449874  4.212688276  4.212688276 | 3.322240092  3.322240092  1.661120046  4.983360138  9.966720275  2303.973504  0  0  0 | 7.018378255  5.263783691  14.03675651  1.754594564  7.018378255  2779.277789  0  0  0 | 0.6788559006  0.6788559006  5.430847205  2.715423602  4.073135404  2276.203835  0  0  0 | 5.297342272  0.6621677839  5.959510055  2.648671136  2.648671136  2657.279317  0  0  0.6621677839 |
| 15440 ENSG00000229261 4.556118599 3.517437407 1.996612468 1.761702616 0.07811955569 0.1954633142 0 0 0 2.364429908 16.05854768 11.42166816 12.89282732 11.93595012 0 0 0 0 | | | | | | | | | | | | | | | | | | | |
| 15441  15443  15449  15450  15455  15470  15473  15476  15489 | ENSG00000142182 ENSG00000273238 ENSG00000242583 ENSG00000271046 ENSG00000228323 ENSG00000259362 ENSG00000171815 ENSG00000228354 ENSG00000130303 | 1.111456958  1.647903639  2.520586026  1.542449793  1.497071775  1.83399538  1.086435772  1.944814877  636.4479107 | 2.939387715  3.657756657  3.465626899  2.648137996  2.611882117  2.978775573  2.226797638  2.179003635  2.570196514 | 1.668487893  2.076753821  1.969030831  1.504762958  1.484737249  1.694788757  1.267037903  1.240080509  1.464123559 | 1.761707548  1.761285628  1.760067361  1.75983731  1.759154436  1.757608764  1.75748305  1.75714691  1.755450555 | 0.0781187219  0.07819007136  0.07839638601  0.07843539505  0.07855128099  0.0788141011  0.07883550855  0.07889277174  0.07918227117 | 0.1954633142  0.1956144148  0.1960543966  0.1961392549  0.196365496  0.1968338103  0.1968467571  0.1969515531  0.1975083638 | 0  0  0  1.265246467  0  0  0  0  193.5827095 | 0  0  0.917401664  0  0  0.917401664  0  0  111.923003 | 0  0  0  0  0  0  0  0  170.5053609 | 0  0  0  0  1.182214954  0  1.182214954  2.364429908  117.0392805 | 0  6.56940587  0  0.7299339855  1.459867971  0  2.189801957  0  4.379603913 | 1.038333469  6.230000815  0  0  4.153333876  0  1.038333469  3.115000407  1.038333469 | 0.9209162369  2.762748711  0  1.841832474  0.9209162369  0.9209162369  2.762748711  1.841832474  0.9209162369 | 0  4.212688276  0  0  3.510573564  0  2.106344138  1.404229425  2.106344138 | 1.661120046  0  8.305600229  1.661120046  4.983360138  6.644480183  0  3.322240092  1646.169965 | 7.018378255  0  7.018378255  7.018378255  1.754594564  3.509189128  1.754594564  5.263783691  1819.514563 | 2.036567702  0  4.073135404  1.357711801  0  3.394279503  0.6788559006  2.715423602  1769.098477 | 0.6621677839  0  9.932516759  4.635174488  0  6.621677839  1.324335568  3.31083892  1801.096372 |
| 15499 ENSG00000243885 1.840370683 3.773448726 2.151600639 1.753786766 0.07946705157 0.1980908147 0 0 0 0 0 0 0 0 4.983360138 7.018378255 6.109703105 3.973006704 | | | | | | | | | | | | | | | | | | | |
| 15504  15509  15510  15511  15514  15520  15527  15535  15543 | ENSG00000283235 ENSG00000162882 ENSG00000277453 ENSG00000259514 ENSG00000129151 ENSG00000238121 ENSG00000158270 ENSG00000251913 ENSG00000265100 | 1.262630631  1.828409111  0.9554389889  1.578784911  2.467946911  1.354205568  1.419696939  3.947137061  3.312993422 | 2.307820024  3.764843326  2.90489939  3.597588708  3.429130498  2.529592147  2.458656761  2.062574641  2.102164832 | 1.316641915  2.14902491  1.658770006  2.054475096  1.958485855  1.445440786  1.405427053  1.179984095  1.203679386 | 1.752807652  1.751884452  1.751236988  1.751098719  1.750908994  1.750048962  1.749401903  1.747968171  1.746449143 | 0.07963502927  0.07979367864  0.0799050966  0.07992890687  0.07996158736  0.08010986549  0.08022157224  0.08046953898  0.08073293691 | 0.1984455209  0.1987767602  0.1990414834  0.199087958  0.1991308449  0.1994229802  0.1996110288  0.2001268114  0.2006769395 | 1.265246467  0  0  0  0  0  0  0  0 | 0  0  0  0  0  0.917401664  0  2.752204992  0.917401664 | 0  0  0  0  0.9913102377  0  0.9913102377  1.982620475  1.982620475 | 0  0  0  0  0  0  0  0  1.182214954 | 0.7299339855  0  2.919735942  3.649669928  0  0  0.7299339855  0  3.649669928 | 0  0  2.076666938  4.153333876  0  2.076666938  1.038333469  2.076666938  16.61333551 | 0.9209162369  0  3.683664948  5.525497422  0  0  0  0.9209162369  0.9209162369 | 0.7021147127  0  2.106344138  5.616917702  0  0.7021147127  0.7021147127  2.808458851  8.425376553 | 3.322240092  6.644480183  0  0  9.966720275  1.661120046  4.983360138  16.61120046  1.661120046 | 3.509189128  5.263783691  0  0  5.263783691  3.509189128  5.263783691  3.509189128  1.754594564 | 2.715423602  4.073135404  0.6788559006  0  6.109703105  4.073135404  0.6788559006  6.109703105  0 | 1.986503352  5.959510055  0  0  7.283845623  3.31083892  2.648671136  10.59468454  2.648671136 |
| 15550 ENSG00000263427 1.96361385 3.853679944 2.209269082 1.744323484 0.08110269883 0.2015050012 0 0 0 0 0 0 0 0 11.62784032 5.263783691 2.036567702 4.635174488 | | | | | | | | | | | | | | | | | | | |
| 15558  15567  15572  15575  15580  15586  15587  15593  15595 | ENSG00000238142 ENSG00000158458 ENSG00000090402 ENSG00000276272 ENSG00000251544 ENSG00000237879 ENSG00000200924 ENSG00000253671 ENSG00000184451 | 10.24415379  43.17351904  1.848160227  1.355596244  3.350181684  2.461392281  0.9348872558  1.82884969  40.67355121 | 2.057656986  2.920393697  3.779215376  2.531221814  2.47723055  3.442182663  2.8809162  3.781865099  2.179873626 | 1.181079799  1.677653146  2.171951497  1.455014821  1.424206754  1.979458437  1.656827999  2.17553003  1.254155598 | 1.742182864  1.740761316  1.740009103  1.73965363  1.739375651  1.738951725  1.73881429  1.738364926  1.738120557 | 0.08147645152  0.08172542539  0.08185741955  0.08191985613  0.08196870832  0.08204325483  0.08206743427  0.08214653285  0.08218957333 | 0.2023295221  0.2028304625  0.2030667397  0.2032046372  0.2032412286  0.2033756381  0.2033787892  0.203535644  0.2036161696 | 3.795739402  16.44820408  0  0  1.265246467  0  0  0  24.03968288 | 1.834803328  1.834803328  0  0.917401664  0  0.917401664  0  0  7.339213312 | 3.965240951  13.87834333  0  0  0.9913102377  0  0  0  8.92179214 | 3.546644862  0  0  0  1.182214954  0  0  0  8.275504679 | 3.649669928  152.556203  0  0  8.029273841  0  2.189801957  0  1.459867971 | 0  105.9100138  0  2.076666938  9.345001222  0  2.076666938  0  2.076666938 | 0.9209162369  124.323692  0  0  9.209162369  0  2.762748711  0  1.841832474 | 0  101.8066333  0  0.7021147127  8.425376553  0  3.510573564  0  0 | 23.25568064  0  3.322240092  1.661120046  0  8.305600229  0  3.322240092  76.41152211 | 29.82810758  0  8.772972819  3.509189128  1.754594564  3.509189128  0  5.263783691  105.2756738 | 19.68682112  0  6.109703105  4.751991304  0  10.18283851  0.6788559006  4.751991304  114.0477913 | 32.44622141  1.324335568  3.973006704  2.648671136  0  6.621677839  0  8.608181191  138.3930668 |
| 15598 ENSG00000253660 8.701513758 3.266602153 1.880015904 1.737539638 0.08229196378 0.2038306206 0 2.752204992 0 2.364429908 0 0 0 0 24.91680069 10.52756738 38.69478633 25.16237579 | | | | | | | | | | | | | | | | | | | |
| 15604  15608  15609  15627  15631  15633  15640  15641  15661 | ENSG00000175318 ENSG00000151033 ENSG00000175868 ENSG00000188624 ENSG00000198788 ENSG00000158865 ENSG00000235062 ENSG00000238005 ENSG00000259658 | 5.162672519  1.795335548  1.98620651  1.604128016  1.077042357  1.309226505  1.72126308  9.023588923  1.629825006 | 2.041182256  3.746890384  3.91828628  3.622782169  3.072309041  3.252490059  2.151948747  2.167680036  3.646959076 | 1.174959776  2.157656446  2.256806245  2.0896645  1.77261423  1.876926813  1.242729517  1.252010955  2.110594184 | 1.737235858  1.7365556  1.736208542  1.733666897  1.73320793  1.732880599  1.731630832  1.731358681  1.727930032 | 0.08234554795  0.0824656423  0.08252696749  0.08297720325  0.083058718  0.08311689336  0.08333931305  0.08338781125  0.08400076926 | 0.2038851576  0.2041299391  0.204268652  0.2051464931  0.205295475  0.2054129837  0.2058704834  0.205977117  0.2072262129 | 2.530492935  0  0  0  0  0  0  2.530492935  0 | 0  0  0  0  0  0  1.834803328  0.917401664  0 | 2.973930713  0  0  0  0  0  0  4.956551189  0 | 1.182214954  0  0  0  0  0  0  2.364429908  0 | 0.7299339855  0  0  5.109537899  0.7299339855  0  5.839471884  0.7299339855  5.839471884 | 1.038333469  0  0  2.076666938  0  1.038333469  1.038333469  0  4.153333876 | 0  0  0  6.446413659  1.841832474  0  2.762748711  1.841832474  1.841832474 | 1.404229425  0  0  5.616917702  0  0  1.404229425  0  7.72326184 | 13.28896037  6.644480183  3.322240092  0  1.661120046  1.661120046  3.322240092  24.91680069  0 | 14.03675651  3.509189128  1.754594564  0  0  7.018378255  1.754594564  24.56432389  0 | 10.86169441  5.430847205  8.825126708  0  3.394279503  1.357711801  2.036567702  17.65025342  0 | 13.90552346  5.959510055  9.932516759  0  5.297342272  4.635174488  0.6621677839  27.81104693  0 |
| 15664 ENSG00000170965 2.482308671 2.600559261 1.505557427 1.727306587 0.08411261682 0.207449151 1.265246467 0 0 1.182214954 1.459867971 0 0 3.510573564 1.661120046 8.772972819 0.6788559006 11.25685233 | | | | | | | | | | | | | | | | | | | |
| 15694  15707  15708  15724  15725  15726  15727  15732  15738 | ENSG00000263938 ENSG00000068831 ENSG00000272046 ENSG00000175262 ENSG00000171557 ENSG00000080031 ENSG00000184454 ENSG00000231378 ENSG00000181291 | 1.097142939  1.622560533  1.885534951  1.895125949  3.830512221  8.871974474  1.107553732  0.7632125746  1.518936306 | 3.051247628  3.631758233  3.813757678  3.830175498  3.320213953  2.535380806  2.983896522  2.423751162  3.547047441 | 1.770142414  2.107753317  2.213431377  2.226211987  1.929897805  1.473872082  1.734684419  1.409534115  2.063825371 | 1.723730025  1.723047096  1.723006965  1.720490016  1.720409208  1.720217675  1.720137962  1.719540618  1.718676149 | 0.08475659205  0.08488000857  0.08488726559  0.08534340697  0.08535808447  0.08539288136  0.08540736669  0.08551597828  0.08567335731 | 0.2086511364  0.2087820164  0.2087865741  0.209694895  0.2097176212  0.2097897731  0.2097924066  0.2100120659  0.210330849 | 0  0  0  0  0  2.530492935  0  0  0 | 0  0  0  0  0  0  0  0  0 | 0  0  0  0  0.9913102377  5.947861426  0  0  0 | 0  0  0  0  1.182214954  0  0  0  0 | 1.459867971  5.109537899  0  0  0  27.00755746  0  0  5.109537899 | 3.115000407  8.306667753  0  0  0  23.88166979  3.115000407  2.076666938  3.115000407 | 5.525497422  1.841832474  0  0  0  21.18107345  0  0.9209162369  3.683664948 | 1.404229425  4.212688276  0  0  0  24.57401495  0  1.404229425  6.319032415 | 1.661120046  0  8.305600229  8.305600229  1.661120046  0  3.322240092  1.661120046  0 | 0  0  3.509189128  1.754594564  14.03675651  0  3.509189128  1.754594564  0 | 0  0  8.825126708  4.073135404  11.54055031  0.6788559006  1.357711801  0.6788559006  0 | 0  0  1.986503352  8.608181191  16.5541946  0.6621677839  1.986503352  0.6621677839  0 |
| 15742 ENSG00000215182 1.225721524 2.352389571 1.369167564 1.718116637 0.08577534279 0.2105152057 0 0 0.9913102377 0 1.459867971 0 0.9209162369 1.404229425 0 5.263783691 1.357711801 3.31083892 | | | | | | | | | | | | | | | | | | | |
| 15743 | ENSG00000135374 | 1.759020016 | 2.878177247 | 1.675262363 | 1.718045668 | 0.08578828569 | 0.210533597 | 1.265246467 | 0 | 0 | 0 | 0 | 0 | 0.9209162369 | 0 | 4.983360138 | 5.263783691 | 2.715423602 | 5.959510055 |

| 15745  15756  15757  15758  15761  15763  15767  15786 | ENSG00000135378 ENSG00000181626 ENSG00000259203 ENSG00000226558 ENSG00000147655 ENSG00000229846 ENSG00000213484 ENSG00000205176 | 368.2672557  2.377777308  1.725837357  1.563410335  1.51168256  1.805351928  1.030615427  1.107657117 | 2.252445952  3.381895408  3.674756901  2.635039739  2.573694363  3.753588358  2.990193363  3.053318317 | 1.311411039  1.971759292  2.142890305  1.536879506  1.501313891  2.190011514  1.74504586  1.785732755 | 1.717574341  1.715166462  1.714860015  1.714538927  1.714294645  1.713958276  1.713532825  1.709840572 | 0.08587428386  0.08631471187  0.08637089508  0.08642979418  0.08647462616  0.08653638901  0.0866145598  0.08729535814 | 0.210717876  0.211653383  0.2117750544  0.2119060222  0.2119755841  0.2121000691  0.2122378079  0.2136344027 | 73.38429511  0  0  0  1.265246467  0  0  0 | 117.427413  0  0  0  0  0  0  0 | 101.1136442  0.9913102377  0  0  0  0  0  0 | 127.679215  0  0  1.182214954  0  0  0  0 | 1.459867971  0  0  0.7299339855  0  0  0.7299339855  1.459867971 | 3.115000407  0  0  2.076666938  1.038333469  0  0  0 | 5.525497422  0  0  0.9209162369  0.9209162369  0  3.683664948  0 | 6.319032415  0  0  3.510573564  0  0  4.212688276  0 | 1014.944348  3.322240092  4.983360138  3.322240092  4.983360138  3.322240092  0  3.322240092 | 1014.155658  8.772972819  7.018378255  7.018378255  5.263783691  7.018378255  1.754594564  1.754594564 | 970.7639378  8.825126708  4.073135404  0  1.357711801  2.715423602  0  5.430847205 | 983.3191591  6.621677839  4.635174488  0  3.31083892  8.608181191  1.986503352  1.324335568 |
| --- | --- | --- | --- | --- | --- | --- | --- | --- | --- | --- | --- | --- | --- | --- | --- | --- | --- | --- | --- |
| 15797 ENSG00000233875 1.331382891 2.468910489 1.445104244 1.708465323 0.08755003539 0.2141226573 0 0 0.9913102377 0 0.7299339855 0 0 0.7021147127 3.322240092 3.509189128 4.073135404 2.648671136 | | | | | | | | | | | | | | | | | | | |
| 15802  15807  15811  15814  15817  15818  15832  15843  15846 | ENSG00000226453 ENSG00000233708 ENSG00000245080 ENSG00000151320 ENSG00000121742 ENSG00000277383 ENSG00000282121 ENSG00000182352 ENSG00000165379 | 1.406028344  0.9225710244  1.741986154  51.2208996  1.455635374  1.781341338  26.67807085  2.376803214  1.818704177 | 2.624419648  2.671352382  3.718873494  2.379164379  3.480845134  2.207880856  2.478198157  2.496324579  3.763676817 | 1.536626272  1.565229666  2.179273429  1.394628754  2.040890875  1.294671532  1.455469041  1.467349788  2.212780038 | 1.707910177  1.706683971  1.706474022  1.705948176  1.705551814  1.705359855  1.70268009  1.701247104  1.700881584 | 0.08765301059  0.08788080809  0.087919859  0.0880177289  0.08809155746  0.08812733079  0.08862795285  0.08889659603  0.08896522533 | 0.2143066741  0.2147956615  0.2148367436  0.2150350927  0.2151746426  0.2152484148  0.2162797472  0.2167846991  0.2169109858 | 1.265246467  0  0  18.97869701  0  0  11.38721821  1.265246467  0 | 0  0  0  11.00881997  0  0.917401664  3.669606656  0  0 | 0  0  0  10.90441262  0  0.9913102377  8.92179214  0  0 | 0  0  0  13.00436449  0  0  2.364429908  1.182214954  0 | 5.109537899  0.7299339855  0  0.7299339855  4.379603913  2.919735942  0  0  0 | 5.191667345  0  0  0  5.191667345  1.038333469  3.115000407  0  0 | 1.841832474  0.9209162369  0  2.762748711  3.683664948  6.446413659  0  0.9209162369  0 | 2.106344138  0  0  0  4.212688276  4.212688276  0  0.7021147127  0 | 0  4.983360138  3.322240092  111.2950431  0  0  58.13920161  4.983360138  1.661120046 | 0  1.754594564  3.509189128  152.649727  0  3.509189128  100.0118901  8.772972819  8.772972819 | 1.357711801  1.357711801  6.788559006  133.7346124  0  0.6788559006  57.70275155  4.073135404  5.430847205 | 0  1.324335568  7.283845623  159.5824359  0  0.6621677839  74.82495959  6.621677839  5.959510055 |
| 15847 ENSG00000260868 1.103309174 3.045192329 1.790691404 1.700567905 0.08902415517 0.2170409689 0 0 0 0 0 0 0 0.7021147127 1.661120046 3.509189128 3.394279503 3.973006704 | | | | | | | | | | | | | | | | | | | |
| 15850  15855  15857  15862  15871  15880  15883  15887  15889 | ENSG00000082684 ENSG00000204659 ENSG00000153902 ENSG00000237640 ENSG00000137976 ENSG00000206603 ENSG00000265460 ENSG00000102970 ENSG00000100060 | 1.507657664  2.314879765  0.7757181347  1.68497425  1.771397207  0.8352158524  2.507799471  1.802174993  1.497405345 | 3.536908844  2.558074145  2.586352628  3.650616484  3.699698317  2.589399285  3.475868975  3.767858409  3.522454064 | 2.081240919  1.505432124  1.522151113  2.149962722  2.180537732  1.527657341  2.051029765  2.224026128  2.079591083 | 1.699423076  1.699229147  1.699143144  1.697990596  1.696690803  1.695013151  1.694694555  1.694161036  1.693820527 | 0.08923949712  0.0892760166  0.08929221604  0.08950953666  0.08975513228  0.09007292595  0.09013337916  0.0902346867  0.09029939233 | 0.2175247931  0.2175572155  0.2175572155  0.2180179643  0.2184921893  0.2191415299  0.2192471891  0.2194515729  0.2195680674 | 0  1.265246467  0  0  0  0  1.265246467  0  0 | 0  0.917401664  0  0  0  0  0  0  0 | 0  0  0  0  0  0  0  0  0 | 0  0  0  0  0  0  0  0  0 | 7.299339855  0.7299339855  2.919735942  0  0  0.7299339855  10.94900978  0  2.189801957 | 3.115000407  1.038333469  1.038333469  0  0  3.115000407  1.038333469  0  4.153333876 | 2.762748711  0  0.9209162369  0  0  0  14.73465979  0  4.604581185 | 4.914802989  0  2.106344138  0  0  1.404229425  2.106344138  0  7.021147127 | 0  1.661120046  1.661120046  6.644480183  9.966720275  1.661120046  0  1.661120046  0 | 0  8.772972819  0  3.509189128  5.263783691  1.754594564  0  5.263783691  0 | 0  6.109703105  0  5.430847205  2.715423602  1.357711801  0  5.430847205  0 | 0  7.283845623  0.6621677839  4.635174488  3.31083892  0  0  9.270348975  0 |
| 15892 ENSG00000253302 1.352564478 2.445121624 1.444014333 1.693280717 0.09040204677 0.2197761815 1.265246467 0 0 0 0 1.038333469 1.841832474 0 4.983360138 1.754594564 2.036567702 3.31083892 | | | | | | | | | | | | | | | | | | | |
| 15904  15914  15919  15921  15922  15923  15926  15933  15935 | ENSG00000203886 ENSG00000259793 ENSG00000186094 ENSG00000151892 ENSG00000224885 ENSG00000206948 ENSG00000170950 ENSG00000248099 ENSG00000184388 | 1.631095001  1.759480631  1.730845547  3.691665104  0.7859143532  1.48839119  0.7876850759  0.8593441949  1.417832375 | 2.79878905  3.726716637  3.679113703  2.111511326  2.551829958  2.656979424  2.531705984  2.673004358  3.447119003 | 1.655372684  2.205923593  2.178204432  1.250370222  1.51113988  1.573547168  1.499873556  1.585271519  2.044546028 | 1.690730479  1.689413291  1.689058037  1.688708903  1.688678852  1.688528618  1.687946277  1.686149235  1.686007043 | 0.09088829036  0.09114025571  0.09120830838  0.09127522863  0.0912809905  0.09130980025  0.09142154222  0.09176705893  0.09179444278 | 0.220791568  0.2212645331  0.2213601982  0.2214947841  0.2214948542  0.2215508468  0.2217801886  0.222520575  0.2225610614 | 0  0  0  3.795739402  0  0  0  0  0 | 0  0  0  0.917401664  0  0.917401664  0  0  0 | 0.9913102377  0  0  0  0  0  0  0  0 | 0  0  0  0  0  0  0  0  0 | 0  0  0  0.7299339855  0.7299339855  2.189801957  0.7299339855  3.649669928  4.379603913 | 0  0  0  0  3.115000407  2.076666938  1.038333469  1.038333469  3.115000407 | 0  0  0  0.9209162369  1.841832474  4.604581185  0.9209162369  0.9209162369  4.604581185 | 0.7021147127  0  0  1.404229425  1.404229425  2.808458851  1.404229425  0.7021147127  4.914802989 | 4.983360138  6.644480183  3.322240092  8.305600229  1.661120046  0  3.322240092  3.322240092  0 | 3.509189128  1.754594564  8.772972819  8.772972819  0  5.263783691  0  0  0 | 4.751991304  5.430847205  2.715423602  10.18283851  0.6788559006  0  2.036567702  0.6788559006  0 | 4.635174488  7.283845623  5.959510055  9.270348975  0  0  0  0  0 |
| 15936 ENSG00000237166 0.7686084892 2.52870098 1.499948375 1.685858675 0.09182302316 0.2226143637 0 0 0 0 1.459867971 3.115000407 0.9209162369 1.404229425 1.661120046 0 0 0.6621677839 | | | | | | | | | | | | | | | | | | | |
| 15939  15940  15949  15950  15953  15956  15960  15961  15974 | ENSG00000232746 ENSG00000241418 ENSG00000259209 ENSG00000255145 ENSG00000272666 ENSG00000257135 ENSG00000224429 ENSG00000242220 ENSG00000261502 | 0.7669933014  0.7652795785  1.596177773  5.6730382  2.394561506  0.7483798027  1.00696216  2.844205938  0.7385950333 | 2.523235921  2.523746431  2.057398919  2.32523999  3.40366158  2.498852988  2.177254325  2.339635726  2.48802472 | 1.496994173  1.497481202  1.221916985  1.381032488  2.021995582  1.484921031  1.294128538  1.390693778  1.479615953 | 1.685534899  1.685327621  1.683746887  1.683696807  1.68331801  1.682818773  1.682409638  1.682351472  1.681534127 | 0.09188541753  0.09192537953  0.09223059583  0.09224027881  0.09231354571  0.09241017962  0.09248943376  0.09250070553  0.0926592135 | 0.2227237033  0.2228065896  0.2234202188  0.2234296659  0.2235791249  0.223743015  0.2238928116  0.2239060684  0.2241072188 | 0  0  0  0  0  0  0  0  0 | 0  0  0.917401664  0.917401664  0.917401664  0  0  0.917401664  0 | 0  0  0.9913102377  3.965240951  0  0  0.9913102377  1.982620475  0 | 0  0  0  1.182214954  0  0  0  0  0 | 2.189801957  1.459867971  1.459867971  0.7299339855  0  1.459867971  1.459867971  1.459867971  1.459867971 | 2.076666938  3.115000407  3.115000407  0  0  2.076666938  0  0  1.038333469 | 1.841832474  0.9209162369  5.525497422  0  0  0.9209162369  1.841832474  0.9209162369  1.841832474 | 0  0.7021147127  2.106344138  0.7021147127  0  2.106344138  2.106344138  0  2.106344138 | 0  1.661120046  1.661120046  11.62784032  1.661120046  0  1.661120046  3.322240092  0 | 1.754594564  0  0  12.28216195  8.772972819  1.754594564  0  8.772972819  1.754594564 | 0.6788559006  0  2.715423602  10.18283851  6.788559006  0  2.036567702  8.146270807  0 | 0.6621677839  1.324335568  0.6621677839  26.48671136  10.59468454  0.6621677839  1.986503352  8.608181191  0.6621677839 |
| 15975 ENSG00000277020 1.41517942 2.645343227 1.573367316 1.681325905 0.09269962876 0.2241909332 1.265246467 0 0 0 4.379603913 2.076666938 3.683664948 4.914802989 0 0 0 0.6621677839 | | | | | | | | | | | | | | | | | | | |
| 15977  15982  15987  15997  16010  16037  16044  16047  16063 | ENSG00000273492 ENSG00000255551 ENSG00000242296 ENSG00000235430 ENSG00000281641 ENSG00000206832 ENSG00000272537 ENSG00000241434 ENSG00000231482 | 3.283368389  0.7308054902  3.85360634  0.7165420961  1.677055575  0.9295296145  1.656468298  1.749709501  1.740975865 | 3.074328033  2.48311443  2.249225908  2.462496536  3.629793732  2.833636048  3.614817194  2.077019273  3.66918622 | 1.828802119  1.477747705  1.339078295  1.467128614  2.164793069  1.692595621  2.160504364  1.241755791  2.197422738 | 1.681061063  1.680337192  1.679682149  1.67844626  1.676739354  1.674136464  1.673135799  1.672647141  1.669768023 | 0.09275105452  0.09289172862  0.09301917481  0.0932600132  0.09359346143  0.09410378287  0.09430056557  0.09439678083  0.0949652694 | 0.2242872249  0.2245571227  0.2247863354  0.2252360199  0.2258578003  0.2267069683  0.2270819216  0.2272711178  0.2284120764 | 1.265246467  0  2.530492935  0  0  0  0  0  0 | 0  0  1.834803328  0  0  0  0  0.917401664  0 | 0.9913102377  0  0  0  0  0  0  0  0 | 0  0  0  0  0  0  0  1.182214954  0 | 0  1.459867971  0  1.459867971  0  3.649669928  0  1.459867971  0 | 0  1.038333469  1.038333469  2.076666938  0  3.115000407  0  3.115000407  0 | 0  1.841832474  0  0  0  0  0  6.446413659  0 | 0  2.106344138  2.106344138  0.7021147127  0  1.404229425  0  0.7021147127  0 | 8.305600229  1.661120046  8.305600229  1.661120046  3.322240092  1.661120046  8.305600229  1.661120046  4.983360138 | 8.772972819  0  12.28216195  0  8.772972819  0  3.509189128  3.509189128  10.52756738 | 8.146270807  0  10.86169441  2.036567702  3.394279503  0  4.751991304  0.6788559006  3.394279503 | 11.91902011  0.6621677839  7.283845623  0.6621677839  4.635174488  1.324335568  3.31083892  1.324335568  1.986503352 |
| 16071 ENSG00000277619 4.77158607 2.763015951 1.655916748 1.668571777 0.09520227606 0.2288539034 0 1.834803328 1.982620475 0 0 0 0 0.7021147127 3.322240092 10.52756738 19.68682112 19.20286573 | | | | | | | | | | | | | | | | | | | |
| 16083  16090  16097  16120  16122  16145  16147  16148  16150 | ENSG00000213578 ENSG00000163817 ENSG00000182583 ENSG00000274395 ENSG00000284424 ENSG00000231226 ENSG00000278530 ENSG00000135925 ENSG00000263884 | 1.064337159  1.694595049  1.671195475  1.582402496  1.09943555  1.690423019  1.710129139  2.282723725  1.085157642 | 3.053640938  3.676742222  2.772824042  3.552624078  2.996473271  3.673282626  3.650705499  2.552673255  2.145700911 | 1.831230692  2.206725745  1.665093556  2.137099385  1.802641914  2.214485015  2.201093306  1.539136919  1.293819325 | 1.667534817  1.666152774  1.665266214  1.662357915  1.662267613  1.658752533  1.658587343  1.658509535  1.658423916 | 0.0954081072  0.09568298942  0.09585965587  0.09644103187  0.09645912858  0.09716566893  0.09719897394  0.09721466465  0.09723193258 | 0.2291918312  0.2297521626  0.2300762754  0.2311413937  0.2311560869  0.232517536  0.232568425  0.2325915636  0.2326040691 | 0  0  0  0  0  0  0  1.265246467  1.265246467 | 0  0  0  0  0  0  0  0  0 | 0  0  0  0  0  0  0  0.9913102377  0 | 0  0  1.182214954  0  0  0  0  0  0 | 0  0  0  0  0  0  0  3.649669928  1.459867971 | 1.038333469  0  0  0  0  0  0  8.306667753  1.038333469 | 0  0  0.9209162369  0  0.9209162369  0  0  8.288246132  0.9209162369 | 0.7021147127  0  0  0  0  0  0  4.212688276  2.808458851 | 1.661120046  1.661120046  6.644480183  4.983360138  1.661120046  1.661120046  3.322240092  0  0 | 0  5.263783691  5.263783691  5.263783691  5.263783691  5.263783691  10.52756738  0  3.509189128 | 4.073135404  6.788559006  3.394279503  5.430847205  2.036567702  4.751991304  2.036567702  0.6788559006  1.357711801 | 5.297342272  6.621677839  2.648671136  3.31083892  3.31083892  8.608181191  4.635174488  0  0.6621677839 |
| 16158 ENSG00000232525 1.666714306 2.050738652 1.237608975 1.657016629 0.09751611315 0.2331684015 0 0.917401664 0 1.182214954 0 3.115000407 0.9209162369 1.404229425 3.322240092 1.754594564 4.073135404 3.31083892 | | | | | | | | | | | | | | | | | | | |
| 16162 | ENSG00000257444 | 1.011775315 | 2.930221033 | 1.769396756 | 1.65605652 | 0.09771037343 | 0.2335532265 | 0 | 0 | 0 | 0 | 0 | 4.153333876 | 2.762748711 | 2.808458851 | 0 | 1.754594564 | 0 | 0.6621677839 |

| 16165  16168  16183  16184  16189  16190  16191  16192 | ENSG00000153303 ENSG00000219790 ENSG00000196344 ENSG00000224939 ENSG00000225255 ENSG00000049540 ENSG00000263644 ENSG00000214076 | 1.674843933  1.067048464  1.732760938  0.8956338135  0.9901538241  6.458305334  2.398351635  6.626761804 | 3.662178813  2.070308215  3.677514313  2.825938703  2.907250319  2.789104672  2.658288738  2.041871481 | 2.211560139  1.25041592  2.225665867  1.710370035  1.760127598  1.688947802  1.609860521  1.236553115 | 1.655925493  1.655695662  1.652320937  1.652238197  1.651727024  1.651385951  1.65125407  1.651260635 | 0.09773690836  0.09778346618  0.09846914329  0.09848600256  0.09859021115  0.09865979174  0.09868670652  0.09868536664 | 0.233595141  0.2336564903  0.2350834426  0.2351091639  0.2352852436  0.2354367544  0.2354718939  0.2354718939 | 0  1.265246467  0  0  0  1.265246467  0  2.530492935 | 0  0  0  0  0  0.917401664  0.917401664  1.834803328 | 0  0  0  0  0  2.973930713  0  2.973930713 | 0  0  0  0  0  0  1.182214954  1.182214954 | 0  1.459867971  0  0  0.7299339855  0  0  0 | 0  2.076666938  0  3.115000407  0  0  8.306667753  1.038333469 | 0  1.841832474  0  0.9209162369  0.9209162369  0  5.525497422  0.9209162369 | 0  1.404229425  0  0.7021147127  0  0  9.829605978  0 | 4.983360138  1.661120046  9.966720275  0  0  9.966720275  1.661120046  16.61120046 | 1.754594564  1.754594564  3.509189128  0  3.509189128  26.31891846  0  17.54594564 | 4.751991304  0.6788559006  1.357711801  2.036567702  4.073135404  12.21940621  1.357711801  18.32910932 | 8.608181191  0.6621677839  5.959510055  3.973006704  2.648671136  23.83804022  0  16.5541946 |
| --- | --- | --- | --- | --- | --- | --- | --- | --- | --- | --- | --- | --- | --- | --- | --- | --- | --- | --- | --- |
| 16194 ENSG00000257660 1.635987964 2.754377357 1.668207303 1.651100167 0.0987181233 0.2355177654 1.265246467 0 0 0 0 0 0 0.7021147127 4.983360138 5.263783691 5.430847205 1.986503352 | | | | | | | | | | | | | | | | | | | |
| 16195  16199  16202  16213  16215  16216  16248  16250  16251 | ENSG00000205869 ENSG00000105251 ENSG00000121797 ENSG00000236347 ENSG00000274649 ENSG00000236478 ENSG00000265342 ENSG00000275664 ENSG00000240216 | 1.573662855  0.8312144986  2.861123784  1.395786769  1.0575677  0.9769535508  1.112752604  1.440077037  2.214317172 | 3.568201131  2.687797387  2.907397534  3.42555052  2.977058809  2.969105069  2.092558328  3.458877277  3.239776669 | 2.161415612  1.628420412  1.762090845  2.078290972  1.806283825  1.801557764  1.273611341  2.105364506  1.97204757 | 1.650863032  1.65055496  1.649970285  1.648253573  1.648167784  1.648076531  1.643011695  1.642887617  1.642849147 | 0.09876654589  0.09882948212  0.09894901382  0.0993006475  0.09931824573  0.09933696765  0.1003805135  0.1004061875  0.1004141487 | 0.2356187404  0.2357106637  0.2359520522  0.2366153026  0.236642641  0.2366664295  0.2386879087  0.2387195725  0.23872381 | 0  0  0  0  0  0  1.265246467  0  1.265246467 | 0  0  0  0  0  0  0  0  0 | 0  0  1.982620475  0  0  0  0  0  0 | 0  0  0  0  0  0  0  0  0 | 0  2.189801957  0  5.839471884  0  0.7299339855  0.7299339855  2.919735942  0 | 0  0  4.153333876  4.153333876  0  0  1.038333469  8.306667753  0 | 0  2.762748711  0  1.841832474  0  0.9209162369  1.841832474  1.841832474  0 | 0  0  0  4.914802989  2.106344138  0.7021147127  0.7021147127  4.212688276  0 | 3.322240092  1.661120046  1.661120046  0  1.661120046  0  3.322240092  0  8.305600229 | 3.509189128  0  10.52756738  0  3.509189128  0  1.754594564  0  7.018378255 | 5.430847205  2.036567702  4.751991304  0  4.751991304  4.073135404  2.036567702  0  2.036567702 | 6.621677839  1.324335568  11.25685233  0  0.6621677839  5.297342272  0.6621677839  0  7.946013407 |
| 16252 ENSG00000127472 1.621053961 3.613809519 2.199872129 1.642736171 0.1004375315 0.2387647076 0 0 0 0 0 0 0 0 4.983360138 1.754594564 5.430847205 7.283845623 | | | | | | | | | | | | | | | | | | | |
| 16254  16257  16261  16264  16274  16275  16278  16281  16300 | ENSG00000165511 ENSG00000231356 ENSG00000267107 ENSG00000182747 ENSG00000274868 ENSG00000277671 ENSG00000198300 ENSG00000279668 ENSG00000234134 | 3.63255865  2.870815361  2.518025552  1.370601457  1.371782708  1.412058807  1.188228765  0.9158248111  0.8982655078 | 2.624425052  2.078380201  2.658671127  3.396290907  3.389985389  3.433450368  3.080637332  2.768193434  2.808612527 | 1.597922573  1.265829327  1.61950202  2.069168944  2.067827445  2.094400447  1.880011612  1.689412423  1.716464035 | 1.642398134  1.641911873  1.641659654  1.64137922  1.639394717  1.63934761  1.638626758  1.63855397  1.63627811 | 0.1005075214  0.100608269  0.1006605573  0.1007187207  0.1011310813  0.1011408859  0.1012910161  0.1013061853  0.1017813953 | 0.2389016913  0.23908504  0.2391650703  0.2392565036  0.2400884432  0.2400969663  0.2404090434  0.2404244831  0.2412468839 | 1.265246467  2.530492935  1.265246467  0  0  0  0  0  0 | 0.917401664  0  0.917401664  0  0  0  0  0  0 | 0  0  0  0  0  0  0  0  0 | 1.182214954  1.182214954  0  0  0  0  0  0  0 | 11.67894377  2.919735942  0  2.189801957  2.919735942  2.919735942  1.459867971  0  2.919735942 | 10.38333469  2.076666938  0  3.115000407  7.268334284  7.268334284  0  2.076666938  4.153333876 | 5.525497422  0.9209162369  0  5.525497422  2.762748711  1.841832474  0  0  0.9209162369 | 12.63806483  0  0.7021147127  5.616917702  3.510573564  4.914802989  0.7021147127  0.7021147127  2.106344138 | 0  1.661120046  4.983360138  0  0  0  8.305600229  0  0 | 0  15.79135107  12.28216195  0  0  0  1.754594564  3.509189128  0 | 0  3.394279503  5.430847205  0  0  0  2.036567702  2.715423602  0.6788559006 | 0  3.973006704  4.635174488  0  0  0  0  1.986503352  0 |
| 16302 ENSG00000218153 1.263508968 3.146969832 1.923849558 1.63576711 0.1018883379 0.2414707359 0 0 0 0 1.459867971 1.038333469 0 0 4.983360138 7.018378255 0 0.6621677839 | | | | | | | | | | | | | | | | | | | |
| 16310  16315  16317  16322  16330  16334  16337  16342  16347 | ENSG00000224090 ENSG00000257803 ENSG00000186288 ENSG00000225978 ENSG00000120341 ENSG00000154783 ENSG00000188263 ENSG00000259771 ENSG00000201643 | 1.593938685  1.150057649  1.298494983  1.079631909  3.518337165  0.8508199433  1.774505202  0.8597728316  1.399996634 | 3.578641506  2.27545272  3.320906724  2.279636391  2.085911189  2.735492925  3.736766846  2.673411324  2.576068213 | 2.18959004  1.392829958  2.033276781  1.396133536  1.278403236  1.676946266  2.291008164  1.639557029  1.580717537 | 1.634388831  1.633690249  1.633278241  1.632821168  1.631653559  1.631234692  1.631057849  1.63056928  1.629682819 | 0.1021772319  0.1023239069  0.1024104909  0.1025066138  0.1027524886  0.1028408078  0.1028781137  0.1029812356  0.1031685503 | 0.242036625  0.242294934  0.2424850964  0.2426383424  0.2430863019  0.2432505577  0.2433026333  0.2434634707  0.2438317087 | 0  0  0  0  1.265246467  0  0  0  1.265246467 | 0  0  0  0  1.834803328  0  0  0  0 | 0  0  0  0.9913102377  0  0  0  0  0 | 0  1.182214954  0  0  1.182214954  0  0  0  0 | 0  2.919735942  3.649669928  4.379603913  0  0  0  2.189801957  2.189801957 | 0  3.115000407  3.115000407  2.076666938  0  1.038333469  0  2.076666938  5.191667345 | 0  2.762748711  4.604581185  2.762748711  1.841832474  0  0  0  3.683664948 | 0  1.404229425  4.212688276  1.404229425  0.7021147127  2.808458851  0  1.404229425  2.808458851 | 6.644480183  0  0  0  9.966720275  1.661120046  1.661120046  3.322240092  1.661120046 | 1.754594564  1.754594564  0  0  14.03675651  0  7.018378255  0  0 | 5.430847205  0  0  0.6788559006  5.430847205  2.715423602  1.357711801  0  0 | 5.297342272  0.6621677839  0  0.6621677839  5.959510055  1.986503352  11.25685233  1.324335568  0 |
| 16356 ENSG00000073282 4.514739621 2.968192223 1.822525058 1.628615316 0.10339448 0.2442162825 1.265246467 0 1.982620475 0 0 0 0 0 11.62784032 10.52756738 12.21940621 16.5541946 | | | | | | | | | | | | | | | | | | | |
| 16372  16373  16377  16382  16389  16395  16404  16416  16419 | ENSG00000120068 ENSG00000234108 ENSG00000255960 ENSG00000272927 ENSG00000231057 ENSG00000278431 ENSG00000253563 ENSG00000252332 ENSG00000256243 | 0.8253346336  1.13009085  2.041499661  1.040291839  1.339786744  9.796002714  0.753760708  1.028675815  1.955101457 | 2.694623698  2.985616917  3.158639129  2.984470745  2.523406487  2.08019332  2.482182812  2.895210006  2.134790692 | 1.656927886  1.836259285  1.943325752  1.836537132  1.554204181  1.282007172  1.531034205  1.787996607  1.319197886 | 1.626276992  1.625923388  1.625378106  1.62505331  1.623600372  1.622606616  1.621245824  1.619248043  1.618249025 | 0.1038907441  0.1039659542  0.1040820181  0.1041512003  0.104461126  0.1046735253  0.1049649287  0.1053939049  0.1056089414 | 0.2451636267  0.2453127613  0.2455400116  0.2456367495  0.2462389018  0.2466492834  0.2472153147  0.2480441957  0.2485048695 | 0  0  0  0  1.265246467  6.326232337  0  0  0 | 0  0  0.917401664  0  0  1.834803328  0  0  0 | 0  0  0  0  0  1.982620475  0  0  0 | 0  0  0  0  0  2.364429908  0  0  2.364429908 | 1.459867971  0  0  0  0  0  0  0  1.459867971 | 3.115000407  0  0  1.038333469  1.038333469  0  0  0  1.038333469 | 1.841832474  0  0  0  0.9209162369  0.9209162369  0.9209162369  0.9209162369  1.841832474 | 2.808458851  1.404229425  0  0  0  0.7021147127  0.7021147127  0  2.106344138 | 0  6.644480183  6.644480183  1.661120046  1.661120046  24.91680069  1.661120046  4.983360138  4.983360138 | 0  3.509189128  3.509189128  1.754594564  1.754594564  17.54594564  1.754594564  1.754594564  7.018378255 | 0.6788559006  0.6788559006  7.467414906  3.394279503  6.788559006  28.51194782  1.357711801  2.036567702  0 | 0  1.324335568  5.959510055  4.635174488  2.648671136  32.44622141  2.648671136  2.648671136  2.648671136 |
| 16422 ENSG00000189420 1.372408052 3.39775672 2.100013795 1.617968762 0.1056693299 0.2486015442 0 0 0 0 5.839471884 2.076666938 6.446413659 2.106344138 0 0 0 0 | | | | | | | | | | | | | | | | | | | |
| 16423  16424  16429  16432  16433  16434  16439  16462  16463 | ENSG00000141433 ENSG00000268104 ENSG00000122735 ENSG00000237679 ENSG00000147647 ENSG00000205502 ENSG00000233765 ENSG00000250138 ENSG00000242737 | 1.562126666  3.308414497  4.466952933  1.19978671  1.508120438  1.617702897  1.251454831  1.162628744  0.7069248463 | 3.543558869  2.394458135  2.955682667  2.27609207  2.68945891  3.575826921  2.323406533  2.377801393  2.449568993 | 2.190578896  1.480251691  1.828144647  1.408162203  1.663978737  2.212460765  1.43822415  1.474683325  1.519211213 | 1.617635811  1.617602026  1.616766305  1.616356457  1.616282018  1.61622162  1.615469003  1.612414918  1.612395283 | 0.1057411068  0.1057483922  0.1059287353  0.106017267  0.1060333529  0.106046406  0.1062091678  0.1068716807  0.1068759507 | 0.2487552616  0.2487572536  0.249078481  0.2492683245  0.2492909747  0.2493064925  0.249613188  0.2508140895  0.2508140895 | 0  1.265246467  1.265246467  0  0  0  0  1.265246467  0 | 0  0  0  0.917401664  0  0  0  0  0 | 0  0.9913102377  1.982620475  0  0.9913102377  0  0  0  0 | 0  1.182214954  0  0  0  0  1.182214954  0  0 | 0  0.7299339855  0  1.459867971  0  0  0  2.919735942  0.7299339855 | 0  0  0  3.115000407  0  0  0  0  1.038333469 | 0  0  0  1.841832474  0.9209162369  0  0.9209162369  3.683664948  0.9209162369 | 0  0  0  0  0  0  0.7021147127  0.7021147127  2.808458851 | 1.661120046  11.62784032  8.305600229  3.322240092  3.322240092  3.322240092  3.322240092  0  1.661120046 | 7.018378255  10.52756738  10.52756738  1.754594564  3.509189128  8.772972819  3.509189128  0  0 | 5.430847205  5.430847205  16.29254161  0  3.394279503  1.357711801  3.394279503  3.394279503  0 | 4.635174488  7.946013407  15.22985903  1.986503352  5.959510055  5.959510055  1.986503352  1.986503352  1.324335568 |
| 16464 ENSG00000236732 1.113478195 2.248955594 1.394852785 1.612324698 0.1068913017 0.2508348785 0 0.917401664 0 0 0.7299339855 0 3.683664948 0.7021147127 3.322240092 0 1.357711801 2.648671136 | | | | | | | | | | | | | | | | | | | |
| 16480  16483  16488  16501  16504  16509  16512  16515  16518 | ENSG00000276312 ENSG00000189068 ENSG00000168702 ENSG00000134765 ENSG00000186188 ENSG00000231357 ENSG00000113946 ENSG00000207922 ENSG00000258615 | 1.259554962  1.280106695  2.267597437  1.482635658  1.442585149  2.88913161  2.055728608  1.135497612  0.9741697027 | 3.273541789  3.294210731  2.503409624  2.635856914  3.422633454  2.38120377  3.115712373  2.254669437  2.791136667 | 2.03542329  2.048720258  1.557713009  1.641711508  2.132269855  1.483756348  1.941611293  1.405355348  1.740959668 | 1.608285512  1.607935841  1.607105808  1.605554265  1.605159612  1.604848244  1.604704497  1.604341166  1.603217305 | 0.1077726622  0.1078492311  0.1080311594  0.1083718811  0.1084586832  0.1085272058  0.1085588518  0.1086388719  0.1088866862 | 0.2526575732  0.2527910602  0.2531406989  0.2537390234  0.2538960993  0.2539795625  0.253997051  0.2541485204  0.2546819906 | 0  0  1.265246467  0  0  0  1.265246467  1.265246467  0 | 0  0  0.917401664  0.917401664  0  1.834803328  0  0  0 | 0  0  0  0  0  0  0  0  0 | 0  0  0  0  0  1.182214954  0  0  0 | 3.649669928  4.379603913  1.459867971  0  0  2.919735942  0  1.459867971  0 | 5.191667345  5.191667345  0  0  0  6.230000815  0  3.115000407  0 | 2.762748711  3.683664948  0  0.9209162369  0  9.209162369  0  1.841832474  0 | 3.510573564  2.106344138  0  0  0  11.93595012  0  3.510573564  1.404229425 | 0  0  8.305600229  3.322240092  3.322240092  0  8.305600229  0  1.661120046 | 0  0  5.263783691  5.263783691  5.263783691  0  7.018378255  1.754594564  5.263783691 | 0  0  2.715423602  3.394279503  4.751991304  1.357711801  5.430847205  0.6788559006  2.036567702 | 0  0  7.283845623  3.973006704  3.973006704  0  2.648671136  0  1.324335568 |
| 16531 ENSG00000164082 1.505768538 2.668804223 1.665846835 1.602070591 0.1091400004 0.255073735 1.265246467 0 0 0 0 2.076666938 2.762748711 6.319032415 4.983360138 0 0 0.6621677839 | | | | | | | | | | | | | | | | | | | |
| 16536 | ENSG00000167194 | 0.8501893437 | 2.732413752 | 1.706001807 | 1.60164763 | 0.109233552 | 0.2552151839 | 0 | 0 | 0 | 0 | 1.459867971 | 1.038333469 | 0 | 0 | 1.661120046 | 0 | 3.394279503 | 2.648671136 |

| 16539  16543  16544  16545  16549  16551  16554  16558 | ENSG00000284087 ENSG00000211687 ENSG00000211690 ENSG00000214252 ENSG00000249388 ENSG00000172188 ENSG00000223979 ENSG00000226913 | 1.56884819  1.544761321  1.544761321  1.439803796  1.510122953  1.436186282  0.7355173914  0.7283323981 | 2.002010052  3.552122382  3.552122382  3.419973383  3.465759977  3.417758635  2.399137807  2.397879062 | 1.25030166  2.218959655  2.218959655  2.136517594  2.165734311  2.13594499  1.499862191  1.499623323 | 1.601221622  1.600805302  1.600805302  1.600723248  1.600270153  1.600115476  1.599572162  1.598987576 | 0.1093278415  0.1094200488  0.1094200488  0.1094382296  0.1095386652  0.1095729684  0.1096935282  0.1098233632 | 0.2553737096  0.2555272961  0.2555272961  0.2555543066  0.2557270125  0.2557761847  0.2560112035  0.2562324743 | 0  0  0  0  0  0  0  0 | 0  0  0  0  0  0  0  0 | 0.9913102377  0  0  0  0  0  0  0 | 1.182214954  0  0  0  0  0  0  0 | 4.379603913  0  0  0  0  0  0.7299339855  0.7299339855 | 2.076666938  0  0  0  0  0  0  1.038333469 | 3.683664948  0  0  0  0  0  1.841832474  0.9209162369 | 3.510573564  0  0  0  0  0  1.404229425  1.404229425 | 1.661120046  3.322240092  3.322240092  3.322240092  3.322240092  4.983360138  0  3.322240092 | 0  1.754594564  1.754594564  5.263783691  8.772972819  3.509189128  3.509189128  0 | 0.6788559006  8.825126708  8.825126708  3.394279503  2.715423602  5.430847205  0.6788559006  0 | 0.6621677839  4.635174488  4.635174488  5.297342272  3.31083892  3.31083892  0.6621677839  1.324335568 |
| --- | --- | --- | --- | --- | --- | --- | --- | --- | --- | --- | --- | --- | --- | --- | --- | --- | --- | --- | --- |
| 16567 ENSG00000231705 1.479428202 3.472402099 2.173145955 1.597868791 0.1100721811 0.2566933491 0 0 0 0 0 0 0 0.7021147127 0 7.018378255 4.073135404 5.959510055 | | | | | | | | | | | | | | | | | | | |
| 16574  16579  16597  16600  16607  16612  16617  16625  16630 | ENSG00000124467 ENSG00000198732 ENSG00000212443 ENSG00000235903 ENSG00000260516 ENSG00000205754 ENSG00000167916 ENSG00000175985 ENSG00000277893 | 14.39875896  15.94562862  0.9704371393  1.093247389  1.842491902  1.641725373  1.574483821  3.466002206  1.944417213 | 2.768641392  2.576161545  2.095792411  2.086363223  2.089627007  3.605959896  3.532149859  2.115445809  3.043386591 | 1.73335775  1.613582282  1.315095256  1.309377476  1.312219623  2.26522198  2.220483185  1.330331281  1.914237042 | 1.597270611  1.596547988  1.593643047  1.593400881  1.592436944  1.591879263  1.590712275  1.590164674  1.58986924 | 0.1102053989  0.1103665007  0.1110160071  0.1110702883  0.1112865607  0.1114118359  0.1116743431  0.1117976909  0.1118642825 | 0.2568954741  0.2571934227  0.2584325494  0.2585060596  0.2589002392  0.2591136697  0.25964604  0.2598077467  0.2598712609 | 2.530492935  7.591478805  0  1.265246467  1.265246467  0  0  1.265246467  1.265246467 | 4.58700832  0.917401664  0  0  0  0  0  1.834803328  0 | 0  3.965240951  0  0  0  0  0  0  0 | 4.728859816  2.364429908  1.182214954  0  1.182214954  0  0  1.182214954  0 | 68.61379464  0  1.459867971  2.189801957  0  0  0  0.7299339855  0 | 12.46000163  0  3.115000407  0  1.038333469  0  0  0  0 | 67.2268853  0  1.841832474  1.841832474  1.841832474  0  0  0  0 | 12.63806483  0  0.7021147127  1.404229425  0.7021147127  0  0  1.404229425  0 | 0  31.56128087  0  3.322240092  1.661120046  9.966720275  1.661120046  13.28896037  4.983360138 | 0  59.65621517  0  1.754594564  7.018378255  1.754594564  10.52756738  1.754594564  7.018378255 | 0  21.72338882  1.357711801  0.6788559006  4.751991304  1.357711801  3.394279503  10.86169441  5.430847205 | 0  63.56810726  1.986503352  0.6621677839  2.648671136  6.621677839  3.31083892  9.270348975  4.635174488 |
| 16631 ENSG00000204420 0.9566052386 2.813091839 1.769391078 1.589864374 0.1118653796 0.2598712609 0 0 0 0 0 1.038333469 0 0 3.322240092 1.754594564 2.715423602 2.648671136 | | | | | | | | | | | | | | | | | | | |
| 16640  16651  16654  16662  16664  16668  16669  16674  16681 | ENSG00000101958 ENSG00000205822 ENSG00000166104 ENSG00000284435 ENSG00000261265 ENSG00000183230 ENSG00000205456 ENSG00000158352 ENSG00000106565 | 1.628798508  1.269339732  0.9608019099  0.7702722326  0.9639788451  4.593569255  1.53363591  3.318795554  1.491762514 | 3.581183589  3.282385213  2.872566221  2.65010963  2.781830356  2.895686535  3.541980344  2.633931725  3.450932848 | 2.254687174  2.067774454  1.809943323  1.670329574  1.753474925  1.826225301  2.233837346  1.662358558  2.17909758 | 1.588328364  1.587400021  1.587102858  1.586578884  1.586467144  1.585612976  1.585603513  1.584454637  1.583652279 | 0.112212107  0.1124220745  0.1124893506  0.1126080531  0.1126333797  0.1128271309  0.1128292788  0.1130902968  0.1132728701 | 0.2605357424  0.2608508107  0.2609598933  0.2611098386  0.261133742  0.2614972518  0.2614972518  0.262024489  0.2623522172 | 0  0  0  0  0  2.530492935  0  0  0 | 0  0  0  0  0  0  0  1.834803328  0 | 0  0  0  0  0  0.9913102377  0  0.9913102377  0 | 0  0  0  0  0  0  0  0  0 | 0  3.649669928  0.7299339855  1.459867971  0.7299339855  0  0  0  0 | 0  6.230000815  0  1.038333469  0  0  0  0  0 | 0  1.841832474  0  0  0  0  0  0.9209162369  0 | 0  3.510573564  0  0.7021147127  0  0  0  0  0 | 8.305600229  0  1.661120046  0  3.322240092  9.966720275  3.322240092  1.661120046  6.644480183 | 5.263783691  0  1.754594564  0  3.509189128  17.54594564  1.754594564  12.28216195  5.263783691 | 0.6788559006  0  4.073135404  3.394279503  1.357711801  10.18283851  3.394279503  11.54055031  1.357711801 | 5.297342272  0  3.31083892  2.648671136  2.648671136  13.90552346  9.932516759  10.59468454  4.635174488 |
| 16682 ENSG00000250696 1.447152761 3.398957502 2.146348147 1.583600269 0.1132847128 0.2623639179 0 0 0 0 0 0 0 0 4.983360138 7.018378255 2.715423602 2.648671136 | | | | | | | | | | | | | | | | | | | |
| 16685  16686  16688  16702  16703  16709  16719  16730  16748 | ENSG00000264745 ENSG00000274001 ENSG00000183760 ENSG00000173699 ENSG00000172421 ENSG00000235049 ENSG00000198765 ENSG00000260014 ENSG00000248936 | 0.6468000992  0.6991747529  3.397324716  1.62549244  1.004149704  1.452730662  1.437022444  1.009499575  1.136880304 | 2.310024611  2.274729761  2.519221516  3.583798312  2.034547283  3.450649118  3.417893019  2.213802475  2.356052908 | 1.459300652  1.437042406  1.591563021  2.267169836  1.287075923  2.185439812  2.165815918  1.404792002  1.49845375 | 1.582966888  1.582924589  1.582860046  1.580736589  1.580751568  1.57892663  1.578108736  1.575893422  1.572322741 | 0.1134290118  0.1134386534  0.113453367  0.1139382787  0.1139348524  0.1143528869  0.1145406317  0.1150503672  0.1158757216 | 0.2626524108  0.2626574598  0.2626600451  0.2635457941  0.2635457941  0.2644098263  0.2646855257  0.2657017264  0.26730491 | 0  0  1.265246467  0  0  0  0  0  0 | 0  0  0  0  0  0  0  0  0 | 0  0  1.982620475  0  0  0  0  0.9913102377  0.9913102377 | 0  0  0  0  1.182214954  0  0  0  0 | 1.459867971  0.7299339855  0  0  1.459867971  0  0  3.649669928  1.459867971 | 0  1.038333469  1.038333469  0  1.038333469  0  0  1.038333469  0 | 1.841832474  1.841832474  0  0  0.9209162369  0  0  0.9209162369  0 | 0.7021147127  0.7021147127  0  0  2.106344138  0  0  3.510573564  2.808458851 | 0  1.661120046  8.305600229  1.661120046  3.322240092  4.983360138  3.322240092  0  1.661120046 | 1.754594564  1.754594564  8.772972819  10.52756738  0  1.754594564  5.263783691  0  0 | 0.6788559006  0  8.146270807  1.357711801  1.357711801  4.073135404  2.036567702  0.6788559006  4.073135404 | 1.324335568  0.6621677839  11.25685233  5.959510055  0.6621677839  6.621677839  6.621677839  1.324335568  2.648671136 |
| 16750 ENSG00000253844 1.198162784 2.351999213 1.495985368 1.572207365 0.115902468 0.26730491 1.265246467 0 0 0 0.7299339855 0 0.9209162369 0 1.661120046 1.754594564 4.073135404 3.973006704 | | | | | | | | | | | | | | | | | | | |
| 16752  16755  16763  16764  16766  16773  16776  16786  16790 | ENSG00000258872 ENSG00000232117 ENSG00000248138 ENSG00000196090 ENSG00000129465 ENSG00000256482 ENSG00000236574 ENSG00000236238 ENSG00000070526 | 0.8891613596  1.684589357  1.264703186  1.408050572  1.914520584  1.063213275  1.043505661  0.5904405853  1.436626861 | 2.692042442  2.904974382  3.277095918  3.429720213  3.006279465  2.173822561  2.180353495  2.270494342  3.444118314 | 1.712106915  1.848189838  2.086971753  2.184281202  1.914902726  1.385118769  1.389597921  1.447804569  2.197192557 | 1.572356504  1.57179437  1.570263667  1.570182543  1.569938475  1.569412393  1.569053509  1.568232612  1.567508639 | 0.1158678958  0.1159982477  0.1163537832  0.1163726496  0.1164294255  0.1165518782  0.1166354713  0.1168268561  0.1169958486 | 0.26730491  0.2674779051  0.268169684  0.2681971675  0.2682960071  0.2684894897  0.2686019485  0.2688910749  0.2692158791 | 0  0  0  0  1.265246467  0  0  0  0 | 0  0.917401664  0  0  0  0  0  0  0 | 0  0  0  0  0  0.9913102377  0  0  0 | 0  0  0  0  0  0  1.182214954  0  0 | 1.459867971  3.649669928  2.189801957  2.919735942  0  1.459867971  0.7299339855  1.459867971  0 | 0  7.268334284  6.230000815  1.038333469  0  3.115000407  0  1.038333469  0 | 0  2.762748711  1.841832474  10.13007861  0  2.762748711  2.762748711  1.841832474  0 | 0  5.616917702  4.914802989  2.808458851  0  2.106344138  2.808458851  1.404229425  0 | 1.661120046  0  0  0  6.644480183  1.661120046  1.661120046  0  1.661120046 | 3.509189128  0  0  0  7.018378255  0  0  0  3.509189128 | 2.715423602  0  0  0  4.073135404  0  2.715423602  0.6788559006  6.109703105 | 1.324335568  0  0  0  3.973006704  0.6621677839  0.6621677839  0.6621677839  5.959510055 |
| 16793 ENSG00000240707 0.7270838409 2.536792919 1.618900999 1.56698459 0.1171182936 0.2694494892 0 0 0 0 1.459867971 1.038333469 2.762748711 2.106344138 0 0 1.357711801 0 | | | | | | | | | | | | | | | | | | | |
| 16796  16797  16805  16807  16815  16820  16821  16824  16829 | ENSG00000187536 ENSG00000224948 ENSG00000167011 ENSG00000230778 ENSG00000223669 ENSG00000259064 ENSG00000269814 ENSG00000183690 ENSG00000154928 | 1.939801001  0.8837461421  3.301186826  0.7204776502  1.008302076  0.7383212927  0.99439603  2.01658282  1.274056612 | 2.154736675  2.731609655  2.372713211  2.561747319  2.105592949  2.407791469  2.166389322  3.134099196  2.320790406 | 1.375385448  1.74360052  1.516476783  1.637579878  1.347138648  1.541242016  1.386799907  2.007076498  1.48634222 | 1.566642048  1.566648795  1.564622181  1.564349534  1.563011315  1.562241  1.56214989  1.561524535  1.561410538 | 0.1171983836  0.1171968056  0.1176715249  0.1177355054  0.1180499335  0.1182312251  0.1182526823  0.1184000394  0.118426917 | 0.2695695392  0.2695695392  0.2705289714  0.2706438537  0.2712375367  0.2715733284  0.2716064669  0.2718654734  0.2718824018 | 0  0  2.530492935  0  0  0  0  1.265246467  1.265246467 | 0.917401664  0  0  0  0  0  0  0  0 | 0  0  0  0  0  0  0.9913102377  0  0 | 1.182214954  0  1.182214954  0  1.182214954  0  0  0  0 | 0  0  10.2190758  2.919735942  2.189801957  0  2.189801957  0  3.649669928 | 3.115000407  0  8.306667753  0  1.038333469  2.076666938  1.038333469  0  1.038333469 | 1.841832474  1.841832474  8.288246132  0.9209162369  1.841832474  0  3.683664948  0  1.841832474 | 1.404229425  0  8.425376553  2.106344138  2.106344138  0.7021147127  0.7021147127  0  0 | 9.966720275  1.661120046  0  0  0  1.661120046  0  1.661120046  3.322240092 | 3.509189128  1.754594564  0  0  1.754594564  1.754594564  0  5.263783691  3.509189128 | 0.6788559006  2.036567702  0  2.036567702  0  0.6788559006  0.6788559006  4.751991304  0 | 0.6621677839  3.31083892  0.6621677839  0.6621677839  1.986503352  1.986503352  2.648671136  11.25685233  0.6621677839 |
| 16830 ENSG00000159409 1.823590512 2.227063707 1.426491116 1.561218071 0.1184723067 0.2719653934 0 0.917401664 0 1.182214954 0 0 1.841832474 0.7021147127 1.661120046 3.509189128 6.109703105 5.959510055 | | | | | | | | | | | | | | | | | | | |
| 16834  16844  16864  16866  16869  16880  16896  16899  16902 | ENSG00000073067 ENSG00000266709 ENSG00000233154 ENSG00000266850 ENSG00000233307 ENSG00000174156 ENSG00000188573 ENSG00000201900 ENSG00000241104 | 0.9784856451  15.37191875  2.570482751  0.9358663237  0.8017263523  1.827772276  0.7008983321  1.300580423  1.815319056 | 2.083322712  2.175412634  2.186219482  2.062445442  2.559283614  2.975883726  2.355464895  2.504239206  3.020537515 | 1.335032576  1.395375638  1.40541921  1.326106501  1.645752413  1.914623612  1.517298084  1.613782283  1.947031006 | 1.56050328  1.559015777  1.555563967  1.555263804  1.555084224  1.554291772  1.552407481  1.551782562  1.551355631 | 0.1186409956  0.1189926461  0.1198118125  0.1198832541  0.1199260118  0.1201148353  0.1205647547  0.1207142598  0.1208164819 | 0.2722879211  0.2729483254  0.2744858502  0.2746169526  0.2746660422  0.2749192336  0.2756876952  0.2759805566  0.2761652335 | 0  6.326232337  0  0  0  0  0  0  0 | 0  6.421811648  0.917401664  0  0  0  0  0  0.917401664 | 0  0.9913102377  1.982620475  0.9913102377  0  0.9913102377  0  0.9913102377  0 | 1.182214954  4.728859816  0  0  0  0  0  0  0 | 0.7299339855  48.17564304  0  2.189801957  1.459867971  0  0.7299339855  1.459867971  8.029273841 | 1.038333469  35.30333795  0  0  2.076666938  0  0  3.115000407  1.038333469 | 0.9209162369  46.04581185  0.9209162369  0.9209162369  0  0  0.9209162369  2.762748711  8.288246132 | 3.510573564  35.80785035  0.7021147127  2.106344138  1.404229425  0  0  5.616917702  3.510573564 | 1.661120046  0  4.983360138  1.661120046  3.322240092  4.983360138  1.661120046  1.661120046  0 | 0  0  5.263783691  0  0  5.263783691  1.754594564  0  0 | 2.036567702  0  7.467414906  2.036567702  1.357711801  4.073135404  1.357711801  0  0 | 0.6621677839  0.6621677839  8.608181191  1.324335568  0  6.621677839  1.986503352  0  0 |
| 16903 ENSG00000244268 0.6842800517 2.360725088 1.521932214 1.551136816 0.1208689001 0.2762523636 0 0 0 0 0.7299339855 3.115000407 0 0.7021147127 1.661120046 0 0.6788559006 1.324335568 | | | | | | | | | | | | | | | | | | | |
| 16907 | ENSG00000261744 | 0.9720180509 | 2.022196201 | 1.30402191 | 1.550737902 | 0.1209645075 | 0.2764218221 | 0 | 0.917401664 | 0 | 0 | 2.189801957 | 1.038333469 | 0 | 1.404229425 | 1.661120046 | 1.754594564 | 2.036567702 | 0.6621677839 |

| 16914  16926  16931  16934  16940  16941  16948  16954 | ENSG00000283366 ENSG00000204616 ENSG00000223510 ENSG00000186973 ENSG00000198400 ENSG00000272973 ENSG00000128713 ENSG00000139220 | 1.172142919  2.757200548  1.035063219  1.128783022  0.7374161682  1.033680794  1.171691095  1.032246702 | 3.183183692  2.755218265  2.154162109  2.351077051  2.547138519  2.120973335  3.173576637  2.058274965 | 2.053182176  1.778546625  1.390889291  1.518504539  1.64633985  1.371019461  2.052760575  1.331924158 | 1.550365929  1.549140307  1.548766047  1.548284506  1.54715232  1.547004544  1.546004281  1.545339464 | 0.1210537114  0.1213479954  0.1214379703  0.121553813  0.1218265203  0.12186215  0.122103534  0.1222641745 | 0.2765111823  0.2769868723  0.2771153399  0.2773255915  0.2778493276  0.2779141825  0.2783332371  0.278617222 | 0  0  0  0  0  0  0  0 | 0  0  0.917401664  0.917401664  0  0  0  0.917401664 | 0  0  0  0  0  0.9913102377  0  0 | 0  2.364429908  0  0  0  0  0  0 | 5.109537899  0  2.919735942  1.459867971  1.459867971  2.919735942  3.649669928  0.7299339855 | 0  1.038333469  2.076666938  0  2.076666938  0  2.076666938  0 | 5.525497422  0  2.762748711  0  1.841832474  0.9209162369  5.525497422  0.9209162369 | 2.106344138  0  1.404229425  2.106344138  2.808458851  0.7021147127  2.808458851  0.7021147127 | 0  4.983360138  1.661120046  1.661120046  0  0  0  3.322240092 | 0  5.263783691  0  0  0  3.509189128  0  1.754594564 | 0  9.503982608  0.6788559006  4.751991304  0  2.036567702  0  2.715423602 | 1.324335568  9.932516759  0  2.648671136  0.6621677839  1.324335568  0  1.324335568 |
| --- | --- | --- | --- | --- | --- | --- | --- | --- | --- | --- | --- | --- | --- | --- | --- | --- | --- | --- | --- |
| 16959 ENSG00000160181 1.337861589 3.339564522 2.161775849 1.544824605 0.1223886941 0.278818751 0 0 0 0 0 1.038333469 0 0 4.983360138 0 4.073135404 5.959510055 | | | | | | | | | | | | | | | | | | | |
| 16965  16972  16973  16986  16999  17004  17011  17013  17014 | ENSG00000259306 ENSG00000245384 ENSG00000260430 ENSG00000183862 ENSG00000257494 ENSG00000255245 ENSG00000240106 ENSG00000115705 ENSG00000272798 | 1.217942785  2.223012531  1.571025533  1.360729804  1.167325859  1.285880262  0.6259253116  14.07578171  1.393362789 | 3.222123504  2.467491647  2.807572445  3.347932784  2.297507005  3.305837927  2.307889671  3.02908151  3.339963855 | 2.087126978  1.599919022  1.820391849  2.173817087  1.493689943  2.150489752  1.502444223  1.972962189  2.176097783 | 1.543808086  1.542260335  1.542290165  1.540117061  1.538141846  1.537248863  1.536090083  1.535296281  1.534840888 | 0.122634832  0.1230103436  0.1230030979  0.1235318217  0.1240139364  0.1242323788  0.1245162882  0.1247110674  0.1248229164 | 0.2792806798  0.2800038076  0.2800038076  0.2809756229  0.2818564875  0.2822589737  0.2827985888  0.2832076699  0.2834331492 | 0  1.265246467  0  0  1.265246467  0  0  8.856725272  0 | 0  0  0.917401664  0  0  0  0  0  0 | 0  0.9913102377  0  0  0  0  0  0.9913102377  0 | 0  0  0  0  0  0  0  0  0 | 3.649669928  0  4.379603913  0  0.7299339855  5.839471884  0.7299339855  0  0 | 3.115000407  0  3.115000407  0  0  1.038333469  3.115000407  0  0 | 6.446413659  0  5.525497422  0  1.841832474  6.446413659  0.9209162369  0  0 | 1.404229425  0.7021147127  4.914802989  0  0  2.106344138  1.404229425  0  0 | 0  3.322240092  0  1.661120046  1.661120046  0  0  39.8668811  4.983360138 | 0  7.018378255  0  5.263783691  1.754594564  0  0  31.58270215  7.018378255 | 0  5.430847205  0  5.430847205  5.430847205  0  0.6788559006  35.30050683  3.394279503 | 0  7.946013407  0  3.973006704  1.324335568  0  0.6621677839  52.31125493  1.324335568 |
| 17015 ENSG00000276197 1.276806228 3.288089021 2.142311607 1.534832286 0.12482503 0.2834331492 0 0 0 0 2.919735942 7.268334284 0.9209162369 4.212688276 0 0 0 0 | | | | | | | | | | | | | | | | | | | |
| 17017  17023  17030  17031  17033  17034  17040  17046  17053 | ENSG00000253721 ENSG00000164451 ENSG00000121904 ENSG00000260608 ENSG00000232139 ENSG00000162881 ENSG00000164749 ENSG00000233242 ENSG00000239983 | 1.295287904  1.345986879  0.8821234997  0.9087981965  0.8811959034  0.9078706001  8.317524121  1.263552893  0.9030452715 | 2.28649756  3.334030859  2.68394443  2.67788959  2.682564819  2.676416461  2.095566331  2.394851366  2.051946071 | 1.489827498  2.173604008  1.750621558  1.746693191  1.749990032  1.746026602  1.367745055  1.563694736  1.34167495 | 1.534739803  1.533872245  1.533137998  1.533119613  1.532902913  1.532861217  1.532132267  1.531533816  1.529391357 | 0.1248477546  0.1250610852  0.1252418571  0.1252463859  0.125299778  0.1253100535  0.1254897988  0.1256375155  0.1261674539 | 0.2834514309  0.2838212711  0.2841227245  0.2841227245  0.2842104692  0.2842170903  0.2845245525  0.2847592052  0.2857423988 | 0  0  0  0  0  0  2.530492935  0  0 | 0  0  0  0  0  0  3.669606656  0.917401664  0.917401664 | 0  0  0  0  0  0  2.973930713  0  0 | 1.182214954  0  0  0  0  0  1.182214954  0  0 | 0.7299339855  0  0.7299339855  0.7299339855  0  0  0  0  2.189801957 | 0  0  0  0  0  0  0  0  2.076666938 | 0  0  0  0  0  0  0.9209162369  0  0.9209162369 | 0.7021147127  0  0  0  0.7021147127  0.7021147127  0  1.404229425  1.404229425 | 4.983360138  3.322240092  1.661120046  3.322240092  1.661120046  3.322240092  23.25568064  3.322240092  0 | 5.263783691  3.509189128  3.509189128  3.509189128  3.509189128  3.509189128  17.54594564  3.509189128  0 | 1.357711801  2.036567702  2.036567702  1.357711801  2.715423602  2.036567702  29.19080373  2.036567702  0.6788559006 | 1.324335568  7.283845623  2.648671136  1.986503352  1.986503352  1.324335568  18.54069795  3.973006704  2.648671136 |
| 17058 ENSG00000078898 1.353335843 3.310405081 2.164248654 1.529586296 0.1261191637 0.2857423988 0 0 0 0 0 0 0 0 4.983360138 5.263783691 1.357711801 4.635174488 | | | | | | | | | | | | | | | | | | | |
| 17061  17065  17066  17076  17091  17092  17095  17096  17100 | ENSG00000197888 ENSG00000258446 ENSG00000204936 ENSG00000165606 ENSG00000174473 ENSG00000140506 ENSG00000263307 ENSG00000243916 ENSG00000188906 | 1.369246727  0.6025436274  1.540562833  5.285652719  2.98643979  1.34793207  0.8656994962  1.788560171  379.8431644 | 2.473743075  2.279370971  2.768546203  2.744905972  2.31916473  3.33770039  2.62552962  2.146724239  2.397857078 | 1.617667816  1.491059977  1.811090255  1.797477975  1.520872558  2.189446466  1.722802601  1.408746278  1.574949228 | 1.529203369  1.528691673  1.528662747  1.527087403  1.524890904  1.524449417  1.523987495  1.523854417  1.522498018 | 0.1262140356  0.1263408975  0.1263480721  0.1267392789  0.127286311  0.1273964835  0.1275118351  0.1275450823  0.1278843416 | 0.2858143875  0.2860340891  0.2860340891  0.2867294385  0.2877366232  0.287968824  0.288178985  0.2882372634  0.2889363472 | 0  0  0  0  1.265246467  0  0  1.265246467  129.0551397 | 0  0  0  0.917401664  0  0  0  0.917401664  35.7786649 | 0  0  0.9913102377  0  1.982620475  0  0  0  186.3663247 | 1.182214954  0  0  3.546644862  0  0  0  0  43.7419533 | 0.7299339855  2.189801957  5.109537899  21.16808558  1.459867971  0  0  5.839471884  0.7299339855 | 0  2.076666938  5.191667345  7.268334284  0  0  1.038333469  1.038333469  0 | 0  0.9209162369  3.683664948  22.10198969  0  0  0  0  0.9209162369 | 0  0.7021147127  3.510573564  8.425376553  0  0  1.404229425  1.404229425  1.404229425 | 4.983360138  0  0  0  6.644480183  4.983360138  0  1.661120046  1109.628191 | 3.509189128  0  0  0  7.018378255  1.754594564  5.263783691  5.263783691  972.0453883 | 2.715423602  0.6788559006  0  0  10.18283851  6.788559006  1.357711801  4.073135404  1100.425415 | 3.31083892  0.6621677839  0  0  7.283845623  2.648671136  1.324335568  0  978.0218169 |
| 17101 ENSG00000274225 1.086499384 3.069410577 2.016544072 1.522114304 0.1279804425 0.2891365648 0 0 0 0 3.649669928 3.115000407 2.762748711 3.510573564 0 0 0 0 | | | | | | | | | | | | | | | | | | | |
| 17110  17116  17119  17121  17122  17129  17130  17150  17154 | ENSG00000253483 ENSG00000227110 ENSG00000259937 ENSG00000270866 ENSG00000271167 ENSG00000200013 ENSG00000273937 ENSG00000261420 ENSG00000240207 | 0.7178286451  2.634019796  1.265081631  1.088817657  1.088817657  0.572207125  1.124302383  1.349629052  0.7933470452 | 2.49465687  2.214121495  3.221098352  3.071930023  3.071930023  2.241966533  3.110701477  2.471001934  2.600699083 | 1.641093516  1.457433302  2.121221201  2.023203715  2.023203715  1.477307592  2.04970924  1.630243713  1.716394331 | 1.520118656  1.519192331  1.518511295  1.518349338  1.518349338  1.517603067  1.517630607  1.515725479  1.515210716 | 0.1284811566  0.1287140912  0.1288855543  0.1289263559  0.1289263559  0.1291144933  0.1291075466  0.1295887822  0.1297190503 | 0.2901151072  0.2905391981  0.2908582588  0.2909163509  0.2909163509  0.2912048132  0.2912048132  0.2919166579  0.2921590013 | 0  0  0  0  0  0  0  0  0 | 0  0.917401664  0  0  0  0  0  0  0 | 0  1.982620475  0  0  0  0  0  0.9913102377  0 | 0  0  0  0  0  0  0  0  0 | 0.7299339855  0.7299339855  0  4.379603913  4.379603913  1.459867971  3.649669928  0  2.919735942 | 0  1.038333469  0  3.115000407  3.115000407  1.038333469  5.191667345  0  0 | 0  0  0  2.762748711  2.762748711  0.9209162369  1.841832474  0  2.762748711 | 2.106344138  0  0  2.808458851  2.808458851  2.106344138  2.808458851  0.7021147127  1.404229425 | 0  1.661120046  4.983360138  0  0  0  0  4.983360138  0 | 1.754594564  10.52756738  3.509189128  0  0  0  0  3.509189128  1.754594564 | 2.036567702  7.467414906  2.715423602  0  0  0.6788559006  0  2.036567702  0.6788559006 | 1.986503352  7.283845623  3.973006704  0  0  0.6621677839  0  3.973006704  0 |
| 17161 ENSG00000278950 1.335005205 3.304091255 2.181356382 1.514695756 0.1298494696 0.2923334454 0 0 0 0 0 0 0 0 3.322240092 5.263783691 6.109703105 1.324335568 | | | | | | | | | | | | | | | | | | | |
| 17167  17174  17175  17177  17186  17196  17199  17203  17204 | ENSG00000258689 ENSG00000123685 ENSG00000242078 ENSG00000275297 ENSG00000257696 ENSG00000264727 ENSG00000143167 ENSG00000052850 ENSG00000266265 | 0.9893901988  3.498203954  0.7788996806  4.318713245  1.241188287  1.316644766  1.081866954  1.263191237  1.218454463 | 2.831475987  2.623695247  2.583709175  2.462832072  3.209159207  3.287170367  2.273034498  3.266490087  3.221075972 | 1.870934187  1.735164545  1.708831159  1.629015208  2.12409348  2.177318977  1.505887591  2.165895558  2.135895757 | 1.513402239  1.512072878  1.511974522  1.511853333  1.510837088  1.509733026  1.509431722  1.508147553  1.508067967 | 0.1301775166  0.1305153238  0.130540344  0.1305711782  0.1308299629  0.1311115607  0.1311884917  0.1315167668  0.1315371326 | 0.2929695551  0.2936100811  0.2936492688  0.2936939722  0.2941123948  0.2945740374  0.2946954693  0.2953641973  0.2953927643 | 0  0  0  0  0  0  0  0  0 | 0  0  0  0.917401664  0  0  0.917401664  0  0 | 0  1.982620475  0  0.9913102377  0  0  0  0  0 | 0  1.182214954  0  2.364429908  0  0  0  0  0 | 0  6.56940587  2.919735942  0  0  0  4.379603913  0  0.7299339855 | 1.038333469  6.230000815  0  0  0  0  3.115000407  0  4.153333876 | 0  11.97191108  2.762748711  0  0  0  1.841832474  0.9209162369  5.525497422 | 0.7021147127  14.04229425  0  0  0  0  1.404229425  0  4.212688276 | 4.983360138  0  1.661120046  11.62784032  3.322240092  6.644480183  0  0  0 | 1.754594564  0  0  10.52756738  3.509189128  1.754594564  0  3.509189128  0 | 3.394279503  0  0.6788559006  9.503982608  4.751991304  4.751991304  0  5.430847205  0 | 0  0  1.324335568  15.89202681  3.31083892  2.648671136  1.324335568  5.297342272  0 |
| 17212 ENSG00000128253 1.750809894 2.882355824 1.912407174 1.50718731 0.1317626521 0.2957616817 1.265246467 0 0 0 0 0 0 0 3.322240092 7.018378255 5.430847205 3.973006704 | | | | | | | | | | | | | | | | | | | |
| 17214  17216  17244  17245  17246  17265  17268  17281  17290 | ENSG00000121898 ENSG00000109072 ENSG00000204677 ENSG00000257764 ENSG00000255129 ENSG00000267892 ENSG00000225431 ENSG00000283691 ENSG00000214141 | 1.285333161  1.533089875  1.126029177  0.9602227526  2.557280316  1.119776182  0.70144589  1.827420726  2.140736982 | 3.254785275  2.748212664  2.145176692  2.819580877  2.682121748  2.33095016  2.35947987  2.242479019  4.01642697 | 2.160136988  1.824079933  1.428268992  1.877372867  1.786068025  1.554740359  1.574191108  1.498515414  2.685963619 | 1.506749476  1.506629514  1.501941654  1.5018758  1.501690703  1.499253652  1.498852241  1.496467102  1.495339305 | 0.1318748844  0.1319056478  0.1331121779  0.1331291876  0.1331770061  0.1338078413  0.1339119685  0.1345319747  0.1348259122 | 0.2959792121  0.2960138652  0.298246836  0.2982572435  0.2983167431  0.2994349211  0.2996113565  0.3007721105  0.3012723607 | 0  0  0  0  1.265246467  0  0  0  0 | 0  0  0.917401664  0  0  0.917401664  0  0.917401664  0 | 0  0  0  0  0.9913102377  0  0  0  0 | 0  1.182214954  0  0  0  0  0  1.182214954  0 | 0  6.56940587  0  0  0  5.839471884  0.7299339855  3.649669928  0 | 1.038333469  4.153333876  1.038333469  1.038333469  0  2.076666938  0  8.306667753  0 | 0  3.683664948  0  0  0  1.841832474  0.9209162369  3.683664948  0 | 0  2.808458851  0.7021147127  0  0  1.404229425  0.7021147127  3.510573564  0 | 0  0  3.322240092  1.661120046  6.644480183  0  1.661120046  0  8.305600229 | 7.018378255  0  3.509189128  3.509189128  7.018378255  0  1.754594564  0  0 | 3.394279503  0  2.036567702  0.6788559006  8.146270807  1.357711801  0  0.6788559006  6.788559006 | 3.973006704  0  1.986503352  4.635174488  6.621677839  0  2.648671136  0  10.59468454 |
| 17300 ENSG00000229298 1.283133317 3.280367131 2.195049629 1.494438708 0.1350609908 0.3016228718 0 0 0 0 1.459867971 7.268334284 0 4.914802989 0 1.754594564 0 0 | | | | | | | | | | | | | | | | | | | |
| 17306 | ENSG00000188848 | 1.062138473 | 3.03003328 | 2.02809598 | 1.494028542 | 0.1351681593 | 0.3017578778 | 0 | 0 | 0 | 0 | 2.919735942 | 3.115000407 | 4.604581185 | 2.106344138 | 0 | 0 | 0 | 0 |

| 17308  17317  17318  17323  17325  17332  17334  17339 | ENSG00000256897 ENSG00000169306 ENSG00000174950 ENSG00000273778 ENSG00000266401 ENSG00000239783 ENSG00000251332 ENSG00000235526 | 1.242895263  1.185381854  1.056008054  1.340371473  1.227112709  1.491989376  1.256895046  0.6721171738 | 2.430381363  2.258721553  3.022327879  2.453890633  2.42638542  2.70423258  2.405060159  2.454158433 | 1.627022437  1.513237236  2.024905816  1.644816547  1.626704688  1.815651322  1.615585978  1.649554874 | 1.493760202  1.492642065  1.492577015  1.491893207  1.491595517  1.489400827  1.488661199  1.487770107 | 0.1352383071  0.135530906  0.1355479436  0.1357271438  0.1358052141  0.136381851  0.1365766074  0.1368115315 | 0.301879593  0.3023755012  0.3023953978  0.3027084339  0.3028475871  0.3040106631  0.3044096703  0.3048453498 | 0  0  0  0  0  0  0  0 | 0  0.917401664  0  0.917401664  0  0  0  0 | 0.9913102377  0  0  0  0  0  0  0 | 0  0  0  0  1.182214954  1.182214954  1.182214954  0 | 4.379603913  0  3.649669928  3.649669928  2.189801957  3.649669928  1.459867971  0 | 4.153333876  1.038333469  4.153333876  1.038333469  4.153333876  3.115000407  0  1.038333469 | 0.9209162369  0  2.762748711  0  0.9209162369  6.446413659  0  0.9209162369 | 2.808458851  0.7021147127  2.106344138  0  5.616917702  3.510573564  0.7021147127  1.404229425 | 1.661120046  3.322240092  0  4.983360138  0  0  4.983360138  0 | 0  3.509189128  0  3.509189128  0  0  0  0 | 0  4.073135404  0  0  0  0  5.430847205  2.715423602 | 0  0.6621677839  0  1.986503352  0.6621677839  0  1.324335568  1.986503352 |
| --- | --- | --- | --- | --- | --- | --- | --- | --- | --- | --- | --- | --- | --- | --- | --- | --- | --- | --- | --- |
| 17353 ENSG00000227070 1.141196798 3.13733626 2.111497011 1.485835047 0.1373227574 0.3057376093 0 0 0 0 5.109537899 4.153333876 0.9209162369 3.510573564 0 0 0 0 | | | | | | | | | | | | | | | | | | | |
| 17358  17359  17365  17366  17374  17378  17384  17397  17413 | ENSG00000006606 ENSG00000170454 ENSG00000244158 ENSG00000123612 ENSG00000123453 ENSG00000075884 ENSG00000105131 ENSG00000275191 ENSG00000156959 | 1.025671552  1.774391095  1.056162882  5.152422004  10.25687063  1.184616962  0.9836181176  0.7266454325  1.648281739 | 2.986709953  2.022827386  3.033423931  2.096044994  2.193611783  3.13710072  2.035642247  2.313046739  2.814825362 | 2.010442039  1.361776471  2.043073797  1.411789831  1.47874879  2.115175774  1.373161762  1.561096282  1.903588817 | 1.485598637  1.485432763  1.48473537  1.484672114  1.483424228  1.483139491  1.482448975  1.481681025  1.478694 | 0.1373853158  0.1374292222  0.1376139383  0.1376307021  0.1379617342  0.1380373538  0.1382208709  0.1384251883  0.1392221167 | 0.305788782  0.3058688863  0.3061741725  0.3061938372  0.3067713152  0.306886475  0.3071884115  0.3074126085  0.3088983219 | 0  0  0  2.530492935  3.795739402  0  0  0  0 | 0  0.917401664  0  0.917401664  0.917401664  0  0  0  0.917401664 | 0  0.9913102377  0  2.973930713  3.965240951  0  0  0  0 | 0  0  0  0  3.546644862  0  1.182214954  0  0 | 2.919735942  0.7299339855  2.919735942  0  31.38716138  0  0.7299339855  0  0 | 3.115000407  1.038333469  2.076666938  0  19.72833591  0  2.076666938  0  0 | 2.762748711  0  2.762748711  0  38.67848195  0  2.762748711  0.9209162369  0 | 3.510573564  0.7021147127  4.914802989  1.404229425  21.06344138  0  1.404229425  0.7021147127  0 | 0  8.305600229  0  9.966720275  0  3.322240092  1.661120046  3.322240092  6.644480183 | 0  5.263783691  0  15.79135107  0  3.509189128  0  1.754594564  3.509189128 | 0  1.357711801  0  17.65025342  0  4.073135404  0  1.357711801  4.073135404 | 0  1.986503352  0  10.59468454  0  3.31083892  1.986503352  0.6621677839  4.635174488 |
| 17414 ENSG00000254403 1.11184249 3.105901203 2.101046254 1.478264078 0.1393371087 0.3091357065 0 0 0 0 5.109537899 1.038333469 3.683664948 3.510573564 0 0 0 0 | | | | | | | | | | | | | | | | | | | |
| 17422  17437  17439  17442  17458  17461  17470  17483  17488 | ENSG00000256394 ENSG00000238358 ENSG00000273782 ENSG00000065325 ENSG00000256955 ENSG00000259517 ENSG00000232448 ENSG00000188817 ENSG00000258857 | 1.23478942  1.082310008  0.6930443346  9.412964073  1.315193898  1.255901411  1.018205055  1.264616752  2.744035355 | 3.204459852  2.196089207  2.285220331  2.195306178  2.333976449  3.214545335  2.977781788  3.21123983  2.268296911 | 2.168909951  1.488799564  1.549261554  1.488851069  1.585190794  2.183524414  2.024367303  2.185006924  1.544451235 | 1.477451773  1.475073784  1.475038431  1.474496828  1.472363111  1.472181999  1.470969119  1.469670322  1.468674996 | 0.1395545768  0.1401927077  0.1402022117  0.1403478712  0.140922849  0.1409717372  0.1412994685  0.1416510643  0.1419209626 | 0.3094557155  0.3106072432  0.3106091202  0.31087834  0.3118658651  0.3119375755  0.3124704084  0.3130291638  0.3135359326 | 0  0  0  5.06098587  1.265246467  0  0  0  0 | 0  0  0  1.834803328  0  0  0  0  0.917401664 | 0  0.9913102377  0  1.982620475  0  0  0  0  0.9913102377 | 0  0  0  2.364429908  0  0  0  0  1.182214954 | 0  0.7299339855  1.459867971  43.06610514  0.7299339855  0  3.649669928  0  4.379603913 | 0  2.076666938  2.076666938  9.345001222  1.038333469  0  2.076666938  1.038333469  8.306667753 | 0  5.525497422  0  33.15298453  0  0  3.683664948  0  10.13007861 | 0  0  0.7021147127  16.14863839  0.7021147127  0  2.808458851  0  7.021147127 | 4.983360138  1.661120046  1.661120046  0  8.305600229  6.644480183  0  0  0 | 1.754594564  0  1.754594564  0  1.754594564  1.754594564  0  8.772972819  0 | 5.430847205  0.6788559006  0  0  0  2.036567702  0  2.715423602  0 | 2.648671136  1.324335568  0.6621677839  0  1.986503352  4.635174488  0  2.648671136  0 |
| 17492 ENSG00000187855 1.215904267 3.195766992 2.176739461 1.468144006 0.1420651104 0.3137826171 0 0 0 0 0 0 0 0 1.661120046 3.509189128 6.109703105 3.31083892 | | | | | | | | | | | | | | | | | | | |
| 17493  17503  17507  17514  17523  17534  17549  17550  17562 | ENSG00000233671 ENSG00000250764 ENSG00000156222 ENSG00000232943 ENSG00000178372 ENSG00000267452 ENSG00000175202 ENSG00000273141 ENSG00000224511 | 1.190632904  0.9941893677  0.8261050168  1.036572337  3.301366242  1.188114994  0.6565130908  0.8097401756  1.207119629 | 3.174752746  2.037344766  2.646412414  2.842891805  2.318619782  3.194313594  2.225404687  2.568282522  3.148964549 | 2.162474124  1.388637452  1.805141704  1.940093757  1.583064209  2.183984468  1.523031088  1.757734197  2.156499131 | 1.468111322  1.467153837  1.466041369  1.465337329  1.464640391  1.462608201  1.461168261  1.461132478  1.460220644 | 0.1420739865  0.1423342115  0.142637017  0.1428289067  0.1430190559  0.1435746179  0.143969271  0.1439790889  0.1442294436 | 0.3137842834  0.3141794128  0.3147758698  0.31508306  0.3153307781  0.3163570984  0.3169555408  0.3169590939  0.3173013014 | 0  1.265246467  0  0  2.530492935  0  0  0  0 | 0  0  0  0  0  0  0  0  0 | 0  0  0  0  0  0  0  0  0 | 0  0  0  0  1.182214954  0  0  0  0 | 0  1.459867971  2.189801957  0  0  1.459867971  0  0  0 | 4.153333876  1.038333469  0  0  1.038333469  4.153333876  1.038333469  1.038333469  0 | 2.762748711  3.683664948  4.604581185  0.9209162369  0  0.9209162369  0  1.841832474  0 | 5.616917702  1.404229425  0.7021147127  0.7021147127  0  7.72326184  1.404229425  0  0 | 0  0  0  6.644480183  9.966720275  0  1.661120046  0  4.983360138 | 1.754594564  1.754594564  1.754594564  3.509189128  8.772972819  0  1.754594564  3.509189128  3.509189128 | 0  0  0  0  9.503982608  0  1.357711801  0.6788559006  1.357711801 | 0  1.324335568  0.6621677839  0.6621677839  6.621677839  0  0.6621677839  2.648671136  4.635174488 |
| 17573 ENSG00000223829 2.623913722 2.862618226 1.962194275 1.458886239 0.1445964212 0.3179014814 0 1.834803328 0 0 8.759207826 5.191667345 12.89282732 2.808458851 0 0 0 0 | | | | | | | | | | | | | | | | | | | |
| 17577  17581  17590  17592  17594  17608  17609  17631  17635 | ENSG00000182968 ENSG00000213332 ENSG00000128610 ENSG00000250337 ENSG00000233384 ENSG00000253582 ENSG00000275812 ENSG00000187889 ENSG00000106013 | 1.225480069  0.6313606839  1.201161341  4.222522828  3.916618314  1.025786023  1.342982282  1.15611101  0.7008490182 | 3.167823787  2.217626047  3.1806217  2.119827623  2.463675003  2.096625594  2.431399519  3.078250268  2.456068881 | 2.171795334  1.521336266  2.184620915  1.456499779  1.693016025  1.443009995  1.673493972  2.122456374  1.69430382 | 1.458619851  1.457683023  1.455914698  1.455425983  1.455198868  1.452952926  1.452888125  1.4503244  1.449603579 | 0.1446697668  0.1449279336  0.145416202  0.1455513675  0.1456142142  0.1462368246  0.1462548185  0.1469680779  0.1471690974 | 0.3179903533  0.3184853372  0.3193948246  0.3196371901  0.3197570288  0.3208689072  0.3208901649  0.3220527303  0.322420078 | 0  0  0  1.265246467  0  0  0  0  0 | 0  0  0  1.834803328  1.834803328  0  0.917401664  0  0 | 0  0  0  0.9913102377  1.982620475  0  0  0  0 | 0  0  0  1.182214954  0  1.182214954  0  0  0 | 0  1.459867971  0  20.43815159  9.489141812  1.459867971  0  0  2.189801957 | 0  2.076666938  0  6.230000815  15.57500204  0  1.038333469  0  2.076666938 | 0  0.9209162369  0  13.81374355  8.288246132  0.9209162369  0  0  2.762748711 | 0  0.7021147127  0  4.914802989  9.829605978  0  0  0  0.7021147127 | 1.661120046  0  3.322240092  0  0  1.661120046  6.644480183  4.983360138  0 | 7.018378255  1.754594564  1.754594564  0  0  1.754594564  3.509189128  3.509189128  0 | 2.715423602  0  2.715423602  0  0  1.357711801  1.357711801  3.394279503  0.6788559006 | 3.31083892  0.6621677839  6.621677839  0  0  3.973006704  2.648671136  1.986503352  0 |
| 17636 ENSG00000236083 2.755785819 2.348504698 1.620205916 1.449510013 0.1471952062 0.3224589924 0 1.834803328 0.9913102377 0 0.7299339855 0 0 0 8.305600229 1.754594564 10.18283851 9.270348975 | | | | | | | | | | | | | | | | | | | |
| 17638  17641  17651  17652  17656  17661  17662  17667  17671 | ENSG00000113492 ENSG00000283209 ENSG00000250730 ENSG00000151025 ENSG00000256894 ENSG00000257283 ENSG00000237864 ENSG00000232936 ENSG00000260653 | 1.129436313  0.768125031  0.6033424542  0.8492233242  8.482394792  0.6875132284  0.6886913781  0.5969435874  1.592632108 | 3.062782628  2.505054655  2.176887923  2.660046121  2.067373047  2.413860459  2.436376316  2.173950673  2.744524084 | 2.11339188  1.729174846  1.503895292  1.837693238  1.428821292  1.668874076  1.684394339  1.503335603  1.898757535 | 1.449226079  1.448699454  1.447499659  1.447491924  1.446908062  1.446400596  1.446440575  1.44608474  1.44543157 | 0.1472744568  0.1474215329  0.1477570318  0.1477591966  0.1479226748  0.1480648747  0.1480536683  0.1481534354  0.1483367013 | 0.3225960222  0.3228632688  0.3234011195  0.3234011195  0.3236672448  0.3238866739  0.3238866739  0.3239886782  0.3243160236 | 0  0  0  0  3.795739402  0  0  0  1.265246467 | 0  0  0  0  0.917401664  0  0  0  0 | 0  0  0  0  4.956551189  0  0  0  0 | 0  0  0  0  1.182214954  0  0  0  0 | 0  1.459867971  1.459867971  4.379603913  0  2.189801957  1.459867971  1.459867971  0 | 0  0  1.038333469  0  1.038333469  1.038333469  3.115000407  1.038333469  0 | 0  0.9209162369  0.9209162369  0.9209162369  0  0  0.9209162369  0.9209162369  0 | 0  0  1.404229425  0.7021147127  0  0  2.106344138  1.404229425  0 | 3.322240092  0  0  0  6.644480183  1.661120046  0  1.661120046  4.983360138 | 3.509189128  3.509189128  1.754594564  3.509189128  26.31891846  0  0  0  3.509189128 | 4.073135404  0.6788559006  0  0.6788559006  26.47538012  2.036567702  0  0.6788559006  3.394279503 | 2.648671136  2.648671136  0.6621677839  0  30.45971806  1.324335568  0.6621677839  0  5.959510055 |
| 17676 ENSG00000231530 0.9625769108 2.087564119 1.444596196 1.445084878 0.1484340459 0.3244367347 0 0 0.9913102377 0 1.459867971 3.115000407 1.841832474 2.106344138 0 0 2.036567702 0 | | | | | | | | | | | | | | | | | | | |
| 17680  17683  17691  17692  17702  17718  17722  17723  17724 | ENSG00000206693 ENSG00000273451 ENSG00000150048 ENSG00000237451 ENSG00000186446 ENSG00000228484 ENSG00000235241 ENSG00000260581 ENSG00000164694 | 1.188646552  1.02384063  1.196974117  2.632565283  10.61288585  0.6562692765  5.554830403  1.125264284  1.125264284 | 2.364067697  2.185495972  3.109709115  2.080990106  2.154405617  2.390742665  2.953649577  3.057940616  3.057940616 | 1.636778907  1.513967937  2.155050191  1.442501398  1.495225811  1.662651444  2.05497068  2.127500231  2.127500231 | 1.444341497  1.44355499  1.442986863  1.442626059  1.440856358  1.437909716  1.437319571  1.437339734  1.437339734 | 0.1486429384  0.1488641935  0.1490241719  0.1491258386  0.1496252676  0.1504596727  0.1506272103  0.1506214839  0.1506214839 | 0.3248201314  0.3252484373  0.325450731  0.3256543507  0.3265604008  0.3280849675  0.3283020575  0.3283020575  0.3283020575 | 1.265246467  0  0  1.265246467  2.530492935  0  0  0  0 | 0  0.917401664  0  0.917401664  2.752204992  0  0  0  0 | 0  0  0  0  3.965240951  0  3.965240951  0  0 | 0  0  0  1.182214954  3.546644862  0  0  0  0 | 0  2.919735942  0  0  0  0.7299339855  0  0  0 | 1.038333469  2.076666938  0  0  0  1.038333469  0  0  0 | 6.446413659  3.683664948  0  0.9209162369  0  2.762748711  0  0  0 | 3.510573564  0.7021147127  0  0  0  0  0  0  0 | 0  0  3.322240092  6.644480183  29.90016083  0  9.966720275  3.322240092  3.322240092 | 0  0  7.018378255  5.263783691  29.82810758  0  19.3005402  3.509189128  3.509189128 | 0.6788559006  0  2.036567702  6.788559006  21.72338882  1.357711801  12.89826211  2.036567702  2.036567702 | 1.324335568  1.986503352  1.986503352  8.608181191  33.1083892  1.986503352  20.5272013  4.635174488  4.635174488 |
| 17726 ENSG00000235621 0.6545973282 2.390414764 1.663082161 1.43734015 0.1506213657 0.3283020575 0 0 0 0 2.189801957 1.038333469 1.841832474 2.106344138 0 0 0.6788559006 0 | | | | | | | | | | | | | | | | | | | |
| 17740 | ENSG00000087128 | 2.783680843 | 2.874175977 | 2.002742794 | 1.43511987 | 0.1512529423 | 0.3294057174 | 0 | 0 | 1.982620475 | 0 | 0 | 0 | 0 | 0 | 4.983360138 | 7.018378255 | 8.825126708 | 10.59468454 |

| 17746  17759  17763  17768  17777  17781  17782  17783 | ENSG00000242199 ENSG00000130561 ENSG00000244953 ENSG00000276032 ENSG00000165443 ENSG00000260337 ENSG00000267295 ENSG00000011083 | 0.5697340329  0.7038570528  0.9817381347  1.559038118  1.543129392  0.8499085987  1.045241091  1.177267997 | 2.132675048  2.434555249  2.931653338  2.733605395  2.733618355  2.566300459  3.006640668  3.16734187 | 1.486293244  1.699380833  2.047104768  1.909495223  1.911018869  1.794365305  2.102455636  2.214908041 | 1.434895205  1.432613103  1.432097363  1.431585354  1.43045074  1.430199554  1.430061409  1.430010552 | 0.1513169624  0.151968435  0.1521159592  0.1522625236  0.1525876944  0.1526597534  0.152699395  0.1527139906 | 0.3294337226  0.3306242779  0.3308562789  0.3310818662  0.3316385231  0.3317029173  0.3317703929  0.3317834464 | 0  0  0  0  0  0  0  0 | 0  0  0  0  0  0  0  0 | 0  0  0  0  0.9913102377  0  0  0 | 0  0  0  1.182214954  0  0  0  0 | 0.7299339855  0  3.649669928  0  0  0  2.919735942  0 | 1.038333469  2.076666938  2.076666938  0  0  0  5.191667345  0 | 0  0  1.841832474  0  0  0  0.9209162369  0 | 1.404229425  0.7021147127  4.212688276  0  0  0.7021147127  3.510573564  0 | 1.661120046  1.661120046  0  3.322240092  3.322240092  3.322240092  0  1.661120046 | 0  0  0  3.509189128  3.509189128  3.509189128  0  1.754594564 | 0.6788559006  1.357711801  0  4.073135404  4.073135404  0.6788559006  0  4.751991304 | 1.324335568  2.648671136  0  6.621677839  6.621677839  1.986503352  0  5.959510055 |
| --- | --- | --- | --- | --- | --- | --- | --- | --- | --- | --- | --- | --- | --- | --- | --- | --- | --- | --- | --- |
| 17784 ENSG00000220326 15.48970834 2.383309074 1.666799926 1.429871118 0.1527540127 0.3318517363 8.856725272 6.421811648 0.9913102377 0 64.23419072 70.6066759 0 0.7021147127 18.2723205 15.79135107 0 0 | | | | | | | | | | | | | | | | | | | |
| 17798  17800  17801  17808  17819  17827  17828  17840  17848 | ENSG00000271964 ENSG00000119698 ENSG00000232732 ENSG00000207088 ENSG00000162456 ENSG00000261294 ENSG00000230729 ENSG00000240156 ENSG00000213640 | 0.7036430712  12.34806474  1.16287223  1.085510915  1.137225856  0.9550589505  1.17029942  15.43780946  0.6019489339 | 2.397966063  2.11943483  2.27770279  2.214877022  3.071704776  2.886079417  3.094439701  2.378115839  2.314054939 | 1.679399171  1.484508503  1.59548168  1.552224307  2.153986641  2.024427098  2.170951245  1.669165493  1.625625428 | 1.42787141  1.427701374  1.427595702  1.426905256  1.42605563  1.425627734  1.425384245  1.424733406  1.423485939 | 0.153328874  0.1533778304  0.1534082613  0.1536072056  0.153852285  0.1539758264  0.1540461597  0.1542342787  0.1545953353 | 0.33283858  0.3329074426  0.3329521463  0.3331782878  0.333581179  0.3336992232  0.3338329245  0.3340157712  0.3346476232 | 0  6.326232337  0  1.265246467  0  0  0  8.856725272  0 | 0  5.504409984  0.917401664  0  0  0  0  6.421811648  0 | 0  0  0  0  0  0  0  0.9913102377  0 | 0  3.546644862  0  0  0  0  0  0  0 | 1.459867971  32.11709536  0  2.919735942  0  2.189801957  0  64.23419072  1.459867971 | 2.076666938  37.38000489  1.038333469  5.191667345  0  2.076666938  0  71.64500937  1.038333469 | 1.841832474  28.54840335  0  0.9209162369  0  3.683664948  0  0  0 | 1.404229425  32.9993915  1.404229425  1.404229425  0  3.510573564  0  0.7021147127  0.7021147127 | 1.661120046  0  0  0  1.661120046  0  1.661120046  16.61120046  0 | 0  1.754594564  5.263783691  0  5.263783691  0  7.018378255  15.79135107  0 | 0  0  1.357711801  0  4.073135404  0  2.715423602  0  2.036567702 | 0  0  3.973006704  1.324335568  2.648671136  0  2.648671136  0  1.986503352 |
| 17853 ENSG00000124657 1.541623488 2.689456065 1.89031904 1.42275246 0.154807927 0.3350139617 1.265246467 0 0 0 0 0 0 0 4.983360138 3.509189128 5.430847205 3.31083892 | | | | | | | | | | | | | | | | | | | |
| 17869  17879  17882  17887  17899  17901  17909  17916  17927 | ENSG00000255020 ENSG00000263325 ENSG00000139973 ENSG00000250692 ENSG00000233694 ENSG00000128710 ENSG00000278463 ENSG00000277653 ENSG00000105852 | 1.110077739  1.862492964  1.242049107  2.465848396  1.071474312  1.026653746  1.022074432  0.9779289563  2.33461253 | 3.073632575  3.821956487  2.318772511  2.708326776  2.979950155  2.985889989  2.011290239  2.914499509  2.529423926 | 2.164633457  2.693980504  1.635287897  1.910642629  2.103889375  2.108205474  1.421448849  2.061441489  1.791291134 | 1.41993212  1.418702356  1.417959807  1.417495211  1.416400591  1.416318298  1.414957872  1.413816266  1.412067462 | 0.1556274437  0.1559858102  0.1562024998  0.1563381935  0.1566582509  0.1566823328  0.1570808482  0.1574158564  0.1579300982 | 0.3364858854  0.3370720833  0.3374837031  0.3376824569  0.3381469089  0.3381611043  0.3388697622  0.3394597909  0.3403597559 | 0  0  0  0  0  0  0  0  1.265246467 | 0  0  0  0  0  0  0.917401664  0  0 | 0  0  0.9913102377  1.982620475  0  0  0  0  0.9913102377 | 0  0  0  0  0  0  0  0  0 | 1.459867971  0  0  8.759207826  0  3.649669928  0.7299339855  3.649669928  0 | 5.191667345  0  0  7.268334284  0  1.038333469  2.076666938  2.076666938  0 | 0  0  0  7.367329896  0  5.525497422  0  4.604581185  0 | 4.914802989  0  0.7021147127  4.212688276  0  2.106344138  2.106344138  1.404229425  0 | 0  4.983360138  4.983360138  0  3.322240092  0  3.322240092  0  4.983360138 | 1.754594564  0  3.509189128  0  3.509189128  0  1.754594564  0  8.772972819 | 0  6.109703105  3.394279503  0  2.715423602  0  1.357711801  0  3.394279503 | 0  11.25685233  1.324335568  0  3.31083892  0  0  0  8.608181191 |
| 17928 ENSG00000227857 1.632687799 2.793253812 1.978250112 1.411982133 0.1579552222 0.3403949136 0 0.917401664 0 0 0.7299339855 0 0 0 0 10.52756738 5.430847205 1.986503352 | | | | | | | | | | | | | | | | | | | |
| 17932  17943  17954  17959  17962  17971  17977  17979  17990 | ENSG00000215498 ENSG00000251323 ENSG00000281756 ENSG00000273272 ENSG00000261447 ENSG00000232504 ENSG00000202358 ENSG00000275799 ENSG00000273739 | 1.507073716  0.9343523902  0.5094410585  1.564085619  0.7335763131  1.511206434  0.9742091949  0.5924281256  1.056519733 | 2.684363726  2.844387972  2.081494545  2.776641444  2.508026741  2.640927089  2.013200601  2.158346126  3.019054028 | 1.902061077  2.017497511  1.47778164  1.971930352  1.781581371  1.877179599  1.431693331  1.535077779  2.149271322 | 1.411292076  1.40985947  1.408526462  1.408082918  1.407753124  1.40685904  1.406167478  1.406017438  1.404687252 | 0.1581585101  0.1585811823  0.1589752369  0.1591065185  0.1592041851  0.1594691913  0.1596743988  0.1597189468  0.1601142981 | 0.3407569729  0.3414581719  0.3420969297  0.3423026093  0.3424370167  0.3428417139  0.3431748205  0.3432193955  0.3438585829 | 0  0  0  0  0  0  0  0  0 | 0.917401664  0  0  0.917401664  0  0  0  0  0 | 0  0  0  0  0  0  0.9913102377  0  0 | 0  0  0  0  0  1.182214954  0  0  0 | 0  2.189801957  0.7299339855  0  0.7299339855  0  1.459867971  0.7299339855  0.7299339855 | 0  4.153333876  1.038333469  0  0  0  1.038333469  1.038333469  5.191667345 | 0  2.762748711  0.9209162369  0  0.9209162369  0  0  0.9209162369  1.841832474 | 0  2.106344138  1.404229425  0  0  0  0.7021147127  0  4.914802989 | 4.983360138  0  0  1.661120046  0  4.983360138  0  0  0 | 3.509189128  0  0  3.509189128  1.754594564  5.263783691  3.509189128  1.754594564  0 | 2.715423602  0  1.357711801  4.073135404  4.073135404  3.394279503  0.6788559006  0.6788559006  0 | 5.959510055  0  0.6621677839  8.608181191  1.324335568  3.31083892  3.31083892  1.986503352  0 |
| 17995 ENSG00000259767 0.6118840352 2.279631188 1.623268224 1.404346586 0.1602156683 0.3439806805 0 0 0 0 0 2.076666938 1.841832474 1.404229425 0 0 1.357711801 0.6621677839 | | | | | | | | | | | | | | | | | | | |
| 18004  18016  18018  18019  18021  18023  18026  18035  18041 | ENSG00000226155 ENSG00000144407 ENSG00000215110 ENSG00000234884 ENSG00000258603 ENSG00000226101 ENSG00000116147 ENSG00000149295 ENSG00000229652 | 0.9138672739  1.104547875  1.104547875  0.7602637101  3.060159993  1.132613248  0.8444373283  1.15239738  3.926095004 | 2.829899114  3.003119069  3.003119069  2.440155835  2.879587209  3.021976016  2.567533808  2.28466696  2.22764874 | 2.016571082  2.14250687  2.14250687  1.740901999  2.054635188  2.156956511  1.833194164  1.632220347  1.592621453 | 1.403322273  1.401684686  1.401684686  1.401661803  1.401507784  1.401037063  1.400579304  1.399729494  1.398730838 | 0.1605207593  0.1610094256  0.1610094256  0.1610162621  0.1610622816  0.1612029901  0.1613399132  0.1615943382  0.1618937129 | 0.3444634268  0.3452390969  0.3452390969  0.3452390969  0.3452994423  0.3455627545  0.3457795278  0.3461711813  0.3466635291 | 0  0  0  0  2.530492935  0  0  1.265246467  0 | 0  0  0  0  0  0  0  0  0 | 0  0  0  0  0  0  0  0  0.9913102377 | 0  0  0  0  0  0  0  0  3.546644862 | 0  0  0  0  0  0  0.7299339855  2.189801957  0.7299339855 | 3.115000407  0  0  0  0  0  0  6.230000815  0 | 1.841832474  0  0  0  0  0  0  2.762748711  0 | 0  0  0  0.7021147127  0  0  0  0.7021147127  0 | 0  3.322240092  3.322240092  3.322240092  11.62784032  4.983360138  4.983360138  0  13.28896037 | 0  5.263783691  5.263783691  1.754594564  5.263783691  5.263783691  1.754594564  0  10.52756738 | 2.036567702  1.357711801  1.357711801  1.357711801  3.394279503  1.357711801  0.6788559006  0.6788559006  6.109703105 | 3.973006704  3.31083892  3.31083892  1.986503352  13.90552346  1.986503352  1.986503352  0  11.91902011 |
| 18042 ENSG00000240040 2.241895154 2.530750458 1.809444847 1.398633654 0.1619228691 0.3466635291 0 0 0.9913102377 1.182214954 9.489141812 5.191667345 0.9209162369 9.127491265 0 0 0 0 | | | | | | | | | | | | | | | | | | | |
| 18043  18049  18050  18052  18064  18065  18069  18071  18072 | ENSG00000283270 ENSG00000268333 ENSG00000245526 ENSG00000250986 ENSG00000141028 ENSG00000196844 ENSG00000187833 ENSG00000279493 ENSG00000267441 | 2.241895154  4.341825775  0.7296660747  9.096362846  1.126227553  0.6966927404  0.9487579802  0.8268140736  2.001760914 | 2.530750458  2.18546815  2.436808014  2.003798499  2.276778459  2.476303651  2.861513407  2.548462489  2.267195353 | 1.809444847  1.562772238  1.742588217  1.43311616  1.63098655  1.773976208  2.050613033  1.826229569  1.625008027 | 1.398633654  1.39845596  1.398384306  1.398210805  1.395951707  1.395905785  1.395442904  1.395477618  1.395190248 | 0.1619228691  0.1619761889  0.1619976934  0.162049773  0.1627290383  0.1627428684  0.1628823209  0.1628718596  0.1629584769 | 0.3466635291  0.346720043  0.3467468634  0.3468233657  0.3480423159  0.3480526279  0.3482352093  0.3482352093  0.3483594729 | 0  1.265246467  0  0  0  0  0  0  1.265246467 | 0  0.917401664  0  7.339213312  0.917401664  0  0  0  0 | 0.9913102377  2.973930713  0  0  0  0  0  0  0 | 1.182214954  0  0  4.728859816  0  0  0  0  1.182214954 | 9.489141812  7.299339855  1.459867971  34.30689732  4.379603913  0.7299339855  0  0  0.7299339855 | 5.191667345  16.61333551  2.076666938  16.61333551  3.115000407  2.076666938  5.191667345  1.038333469  0 | 0.9209162369  8.288246132  2.762748711  23.02290592  2.762748711  0  2.762748711  0.9209162369  0 | 9.127491265  14.74440897  0.7021147127  18.95709724  0  4.212688276  2.106344138  0  0 | 0  0  0  0  1.661120046  0  0  0  4.983360138 | 0  0  1.754594564  3.509189128  0  0  0  5.263783691  1.754594564 | 0  0  0  0.6788559006  0.6788559006  0.6788559006  0  2.036567702  8.146270807 | 0  0  0  0  0  0.6621677839  1.324335568  0.6621677839  5.959510055 |
| 18094 ENSG00000204538 3.029105154 2.17965688 1.566145886 1.391732979 0.1640032718 0.3501860509 2.530492935 0 0 1.182214954 0.7299339855 0 0 0 6.644480183 10.52756738 6.788559006 7.946013407 | | | | | | | | | | | | | | | | | | | |
| 18109  18125  18129  18146  18149  18154  18172  18177  18183 | ENSG00000230646 ENSG00000121075 ENSG00000225473 ENSG00000233539 ENSG00000175879 ENSG00000284713 ENSG00000169903 ENSG00000280233 ENSG00000273650 | 0.7602165666  0.9555938162  1.19793941  1.592621543  0.9900319984  1.077309149  2.953841593  1.925314667  0.6907905162 | 2.496700354  2.897536073  3.143804394  2.728660884  2.928117729  2.192830824  2.816880961  2.323710482  2.353262819 | 1.795727881  2.08659413  2.265056835  1.968304131  2.112662232  1.582140841  2.034786252  1.679163294  1.701912952 | 1.390355621  1.388643834  1.387958282  1.386300441  1.385984794  1.385989646  1.384362096  1.383850213  1.382716323 | 0.1644209155  0.1649410811  0.1651497488  0.1656551823  0.1657515467  0.1657500651  0.1662476089  0.1664043241  0.166751865 | 0.3507870159  0.3515980121  0.3519218258  0.3526996565  0.3527941957  0.3527941957  0.3534351164  0.353707108  0.3543121764 | 0  0  0  0  0  0  2.530492935  0  0 | 0  0  0  0  0  0  0  0.917401664  0 | 0  0  0  0  0  0.9913102377  0  0  0 | 0  0  0  1.182214954  0  0  0  1.182214954  0 | 0  2.189801957  0  0.7299339855  4.379603913  2.189801957  0  5.839471884  0 | 1.038333469  0  0  0  3.115000407  2.076666938  0  7.268334284  0 | 0  3.683664948  0  0  3.683664948  4.604581185  0  3.683664948  0.9209162369 | 0  4.914802989  0  0  0.7021147127  1.404229425  0  4.212688276  0.7021147127 | 1.661120046  0  6.644480183  0  0  1.661120046  6.644480183  0  3.322240092 | 1.754594564  0  1.754594564  10.52756738  0  0  12.28216195  0  0 | 1.357711801  0.6788559006  0.6788559006  2.036567702  0  0  3.394279503  0  1.357711801 | 3.31083892  0  5.297342272  4.635174488  0  0  10.59468454  0  1.986503352 |
| 18187 ENSG00000231527 4.789732671 2.248597627 1.626659313 1.382340856 0.1668670669 0.3544789757 0 0.917401664 0.9913102377 3.546644862 0 0 0 0 11.62784032 12.28216195 12.21940621 15.89202681 | | | | | | | | | | | | | | | | | | | |
| 18193 | ENSG00000261334 | 0.6779431142 | 2.359140508 | 1.708042049 | 1.381195802 | 0.1672187652 | 0.3551209361 | 0 | 0 | 0 | 0 | 2.189801957 | 1.038333469 | 1.841832474 | 1.404229425 | 1.661120046 | 0 | 0 | 0 |

| 18195  18198  18222  18235  18248  18257  18268  18284 | ENSG00000248993 ENSG00000264229 ENSG00000280055 ENSG00000185290 ENSG00000270806 ENSG00000225720 ENSG00000284636 ENSG00000224565 | 2.16794711  1.29492677  1.094589852  1.699603239  1.014902987  1.98098738  0.7024663764  0.9011859366 | 2.486559166  2.485152781  2.228988456  3.68045978  2.893923459  2.329811219  2.429986263  2.800366566 | 1.800514784  1.799935289  1.618873688  2.676097257  2.106347383  1.697430225  1.771821295  2.044869256 | 1.381026798  1.38069007  1.376876079  1.375308678  1.373906071  1.372551981  1.371462387  1.369459958 | 0.1672707213  0.1673742757  0.1685505641  0.1690357674  0.1694708445  0.1698916685  0.1702308609  0.1708555431 | 0.3551802318  0.3553415288  0.3573675252  0.3581407663  0.3588206676  0.3595204368  0.3600213111  0.3610407274 | 0  0  0  0  0  1.265246467  0  0 | 0  0  0  0  0  0  0  0 | 1.982620475  0  0  0  0  0.9913102377  0  0 | 0  1.182214954  1.182214954  0  0  0  0  0 | 0  2.189801957  0.7299339855  0  0  6.56940587  0  3.649669928 | 0  5.191667345  5.191667345  0  0  7.268334284  2.076666938  2.076666938 | 0  2.762748711  1.841832474  0  0  2.762748711  0  3.683664948 | 0.7021147127  4.212688276  3.510573564  0  0  4.914802989  0.7021147127  1.404229425 | 1.661120046  0  0  0  3.322240092  0  1.661120046  0 | 7.018378255  0  0  7.018378255  3.509189128  0  0  0 | 3.394279503  0  0.6788559006  5.430847205  2.036567702  0  0.6788559006  0 | 11.25685233  0  0  7.946013407  3.31083892  0  3.31083892  0 |
| --- | --- | --- | --- | --- | --- | --- | --- | --- | --- | --- | --- | --- | --- | --- | --- | --- | --- | --- | --- |
| 18301 ENSG00000173093 1.259262081 2.471035365 1.806749739 1.367668865 0.17141575 0.3618830052 0 0.917401664 0 0 3.649669928 5.191667345 1.841832474 3.510573564 0 0 0 0 | | | | | | | | | | | | | | | | | | | |
| 18304  18335  18341  18350  18362  18369  18378  18386  18389 | ENSG00000196329 ENSG00000253182 ENSG00000229832 ENSG00000212464 ENSG00000262117 ENSG00000229949 ENSG00000269978 ENSG00000145681 ENSG00000249868 | 0.8942311184  1.103157199  1.095059016  1.590855296  0.968591453  1.108012138  0.6044818618  1.171333996  0.5986671666 | 2.790638656  3.00490796  2.05192332  2.009913994  2.875885066  2.2908684  2.265779782  3.061694284  2.232218104 | 2.041061544  2.204758066  1.506175736  1.476171998  2.114130642  1.685656997  1.668684504  2.256557634  1.646386052 | 1.367248657  1.36291959  1.362339912  1.361571684  1.360315682  1.359035915  1.357823948  1.35679862  1.355829091 | 0.1715473788  0.1729078539  0.1730906373  0.1733330965  0.1737300479  0.1741352082  0.1745195539  0.1748452062  0.1751535532 | 0.3620920038  0.3643267307  0.3646124405  0.3649242104  0.3655437328  0.3662536756  0.366867843  0.3674069694  0.3679948625 | 0  0  1.265246467  1.265246467  0  0  0  0  0 | 0  0  0  0  0  0.917401664  0  0  0 | 0  0  0  0.9913102377  0  0  0  0  0 | 0  0  0  0  0  0  0  0  0 | 1.459867971  0  0  2.919735942  0.7299339855  0  2.189801957  0.7299339855  1.459867971 | 2.076666938  0  0  4.153333876  0  1.038333469  2.076666938  0  0 | 3.683664948  0  0.9209162369  2.762748711  0  0.9209162369  0.9209162369  0  0 | 3.510573564  0  0.7021147127  6.319032415  0  0  1.404229425  0  0.7021147127 | 0  3.322240092  1.661120046  0  0  1.661120046  0  4.983360138  1.661120046 | 0  5.263783691  5.263783691  0  3.509189128  0  0  7.018378255  0 | 0  0.6788559006  0.6788559006  0.6788559006  4.073135404  6.109703105  0  0  2.036567702 | 0  3.973006704  2.648671136  0  3.31083892  2.648671136  0.6621677839  1.324335568  1.324335568 |
| 18394 ENSG00000277942 1.75112168 2.047706457 1.511285403 1.354943582 0.1754355328 0.368487105 0 0 0 2.364429908 0.7299339855 1.038333469 0 0 3.322240092 3.509189128 4.751991304 5.297342272 | | | | | | | | | | | | | | | | | | | |
| 18396  18397  18399  18413  18458  18461  18467  18470  18490 | ENSG00000227482 ENSG00000257046 ENSG00000244004 ENSG00000257159 ENSG00000270670 ENSG00000274378 ENSG00000268170 ENSG00000228839 ENSG00000278954 | 0.6700741075  1.55355526  0.935803243  0.7722379747  0.9943494161  0.9002037422  0.9804387468  2.286386298  1.574256473 | 2.162337372  2.692335104  2.056638358  2.423915836  2.12620978  2.796445129  2.872101775  2.059904319  2.677421906 | 1.59612233  1.987357802  1.518589105  1.791997879  1.579551068  2.077825916  2.135762516  1.532020993  1.995052179 | 1.354744139  1.35473094  1.354308648  1.352633206  1.346084861  1.345851501  1.344766449  1.34456664  1.342031018 | 0.1754990897  0.1755032963  0.1756379283  0.1761728388  0.1782751504  0.1783504123  0.1787006682  0.1787652224  0.1795859387 | 0.368569324  0.368569324  0.3687951826  0.3696606164  0.3731717371  0.3732499962  0.3738614998  0.3739575046  0.3752462273 | 0  1.265246467  0  0  0  0  0  0  1.265246467 | 0  0  0.917401664  0  0.917401664  0  0  2.752204992  0 | 0  0  0  0  0  0  0  0  0 | 0  0  0  0  0  0  0  0  0 | 0  0  1.459867971  0  0.7299339855  2.919735942  0  0  0 | 0  0  2.076666938  2.076666938  0  4.153333876  0  1.038333469  0 | 0.9209162369  0  2.762748711  0  1.841832474  0.9209162369  0  0.9209162369  0 | 0.7021147127  0  0.7021147127  0  0  2.808458851  0  0  0 | 3.322240092  8.305600229  0  1.661120046  0  0  3.322240092  3.322240092  8.305600229 | 1.754594564  1.754594564  0  3.509189128  1.754594564  0  1.754594564  5.263783691  5.263783691 | 0.6788559006  1.357711801  0  1.357711801  2.715423602  0  2.715423602  9.503982608  3.394279503 | 0.6621677839  5.959510055  3.31083892  0.6621677839  3.973006704  0  3.973006704  4.635174488  0.6621677839 |
| 18526 ENSG00000235939 0.9868376136 2.878785215 2.15294732 1.337136858 0.1811779679 0.3778371364 0 0 0 0 0 0 0 0 1.661120046 3.509189128 2.036567702 4.635174488 | | | | | | | | | | | | | | | | | | | |
| 18528  18530  18533  18536  18538  18539  18542  18543  18545 | ENSG00000206828 ENSG00000201772 ENSG00000256542 ENSG00000236988 ENSG00000264801 ENSG00000254811 ENSG00000260919 ENSG00000262332 ENSG00000239093 | 0.8295911407  1.326757146  0.9994599166  1.198476732  0.9790480704  0.9790480704  0.6259647692  0.6259647692  0.8221246441 | 2.683787516  2.515145038  2.948977601  2.193847724  2.870343992  2.870343992  2.09955138  2.09955138  2.674001099 | 2.008006526  1.882837844  2.20843919  1.64334245  2.150266635  2.150266635  1.573436939  1.573436939  2.004164699 | 1.336543224  1.335826686  1.335322075  1.334991208  1.33487817  1.33487817  1.334372753  1.334372753  1.334222232 | 0.1813717824  0.1816059284  0.1817709568  0.1818792239  0.1819162235  0.1819162235  0.1820817249  0.1820817249  0.1821310354 | 0.3782004974  0.3786478706  0.3789306058  0.3790949404  0.3791258953  0.3791258953  0.3793737498  0.3793737498  0.3794151058 | 0  0  0  1.265246467  0  0  0  0  0 | 0  0  0  0  0  0  0  0  0 | 0  0  0  0  0  0  0  0  0 | 0  1.182214954  0  0  0  0  0  0  0 | 2.189801957  0.7299339855  6.56940587  0  0  0  0  0  2.919735942 | 3.115000407  5.191667345  1.038333469  0  0  0  1.038333469  1.038333469  2.076666938 | 1.841832474  4.604581185  3.683664948  1.841832474  0  0  0.9209162369  0.9209162369  2.762748711 | 2.808458851  4.212688276  0.7021147127  0.7021147127  0  0  0.7021147127  0.7021147127  2.106344138 | 0  0  0  3.322240092  3.322240092  3.322240092  0  0  0 | 0  0  0  5.263783691  1.754594564  1.754594564  3.509189128  3.509189128  0 | 0  0  0  0  2.036567702  2.036567702  0.6788559006  0.6788559006  0 | 0  0  0  1.986503352  4.635174488  4.635174488  0.6621677839  0.6621677839  0 |
| 18549 ENSG00000236283 1.349081494 2.489077534 1.866096905 1.333841521 0.1822558002 0.3796136093 0 0 0.9913102377 0 0 0 0 0 4.983360138 3.509189128 3.394279503 3.31083892 | | | | | | | | | | | | | | | | | | | |
| 18550  18558  18568  18569  18570  18583  18585  18601  18619 | ENSG00000284084 ENSG00000276874 ENSG00000248319 ENSG00000130487 ENSG00000268981 ENSG00000233633 ENSG00000233569 ENSG00000262769 ENSG00000261068 | 0.6952511486  1.602989773  2.394194371  0.5968493084  0.8721855125  0.6381472828  1.03378814  4.152523729  12.80485747 | 2.357256521  3.579797398  2.619315567  2.290940636  2.758258153  2.29286517  2.905189646  2.045345316  2.026024968 | 1.76744119  2.685936186  1.968183373  1.721616408  2.073052437  1.724715762  2.185591926  1.54071617  1.529098692 | 1.333711432  1.332793168  1.330829029  1.330691684  1.330529853  1.329416256  1.329246147  1.327528948  1.324979858 | 0.1822984467  0.1825996884  0.183245274  0.1832904806  0.1833437572  0.1837106777  0.1837667749  0.1843337707  0.185177832 | 0.3796819671  0.3801454339  0.3812839918  0.3813575162  0.3814478223  0.3819271289  0.3820193354  0.3828684066  0.3842497202 | 0  0  0  0  0  0  0  0  1.265246467 | 0  0  0  0  0  0  0  0.917401664  4.58700832 | 0  0  1.982620475  0  0  0  0  0.9913102377  4.956551189 | 0  0  0  0  0  0  0  3.546644862  5.91107477 | 0  0  0  0.7299339855  2.919735942  2.189801957  0  12.40887775  0 | 0  0  0  1.038333469  3.115000407  1.038333469  0  10.38333469  0 | 0.9209162369  0  0  0  0.9209162369  0  0  11.05099484  0 | 0  0  0  1.404229425  3.510573564  2.106344138  0  10.53172069  0 | 1.661120046  0  8.305600229  0  0  1.661120046  6.644480183  0  33.22240092 | 1.754594564  10.52756738  7.018378255  0  0  0  1.754594564  0  33.33729671 | 1.357711801  4.073135404  6.788559006  0.6788559006  0  0  1.357711801  0  34.62165093 | 2.648671136  4.635174488  4.635174488  3.31083892  0  0.6621677839  2.648671136  0  35.75706033 |
| 18632 ENSG00000172817 0.9955772544 2.831431339 2.140028544 1.323081109 0.1858084064 0.385270115 0 0 0 0 0 0 0 0 3.322240092 5.263783691 2.036567702 1.324335568 | | | | | | | | | | | | | | | | | | | |
| 18640  18641  18657  18658  18660  18661  18662  18670  18671 | ENSG00000173198 ENSG00000186160 ENSG00000257258 ENSG00000119614 ENSG00000160183 ENSG00000254238 ENSG00000258973 ENSG00000214526 ENSG00000259418 | 1.417059876  7.336133649  0.9583316617  0.9877877112  1.744731579  0.9863970348  0.9863970348  0.6786800117  0.5901569964 | 2.571140165  2.470797934  2.802537738  2.822355988  2.027979326  2.820174846  2.820174846  2.289173414  2.044319547 | 1.944571466  1.868918253  2.122664901  2.137770852  1.536435284  2.137034713  2.137034713  1.736202493  1.550580339 | 1.322214282  1.322047088  1.320292118  1.32023317  1.319924989  1.319667307  1.319667307  1.318494487  1.318422204 | 0.1860968065  0.1861524713  0.1867375062  0.1867571806  0.1868600634  0.1869461198  0.1869461198  0.1873381695  0.187362352 | 0.3857215728  0.3858162506  0.3866968726  0.3867168867  0.3868884538  0.3870251495  0.3870251495  0.3876790426  0.3876791168 | 0  1.265246467  0  0  2.530492935  0  0  0  0 | 0  0  0  0  0  0  0  0  0 | 0.9913102377  5.947861426  0  0  0  0  0  0  0 | 0  0  0  0  0  0  0  0  0 | 0  0  0  0  0.7299339855  0  0  0  0 | 0  0  0  0  0  0  0  2.076666938  0 | 0  0  0  0  0  0  0  0  0.9209162369 | 0  0  0  0  0.7021147127  0  0  1.404229425  1.404229425 | 1.661120046  19.93344055  3.322240092  4.983360138  1.661120046  4.983360138  4.983360138  3.322240092  1.661120046 | 7.018378255  22.80972933  3.509189128  3.509189128  5.263783691  3.509189128  3.509189128  0  1.754594564 | 2.036567702  13.57711801  1.357711801  2.036567702  4.751991304  1.357711801  1.357711801  0.6788559006  0.6788559006 | 5.297342272  24.50020801  3.31083892  1.324335568  5.297342272  1.986503352  1.986503352  0.6621677839  0.6621677839 |
| 18677 ENSG00000279617 0.6002648123 2.04973551 1.555403056 1.317816306 0.1875651479 0.3879948326 0 0 0 0 0.7299339855 0 0.9209162369 0.7021147127 0 3.509189128 0.6788559006 0.6621677839 | | | | | | | | | | | | | | | | | | | |
| 18682  18687  18688  18689  18698  18700  18704  18705  18715 | ENSG00000250334 ENSG00000010610 ENSG00000228952 ENSG00000125820 ENSG00000215808 ENSG00000129221 ENSG00000105989 ENSG00000254714 ENSG00000250820 | 0.9854469372  1.64159852  0.8244429168  0.8244429168  0.5607480824  0.977657394  0.9689025576  0.6713606486  0.9344383177 | 2.877363484  2.027073942  2.676397905  2.676397905  2.029965506  2.868830191  2.816716989  2.266880417  2.792904568 | 2.184904222  1.540080007  2.033529123  2.033529123  1.544363573  2.182638425  2.143617824  1.725365007  2.128115427 | 1.316928887  1.3162134  1.316134534  1.316134534  1.314434982  1.314386367  1.314001478  1.313855566  1.312383968 | 0.1878624627  0.1881024274  0.1881288917  0.1881288917  0.1886998645  0.1887162159  0.1888457068  0.1888948143  0.1893906135 | 0.3885058476  0.3888980191  0.3889111097  0.3889111097  0.3899036938  0.38991517  0.3900798696  0.3901604464  0.3909337155 | 0  0  0  0  0  0  0  0  0 | 0  0.917401664  0  0  0  0  0  0  0 | 0  0  0  0  0  0  0  0  0 | 0  1.182214954  0  0  0  0  0  0  0 | 0  0  3.649669928  3.649669928  0.7299339855  0  0  0  0 | 0  0  2.076666938  2.076666938  1.038333469  0  0  0  0 | 0  0  2.762748711  2.762748711  1.841832474  0  0  1.841832474  0 | 0  0.7021147127  1.404229425  1.404229425  0.7021147127  0  0  0.7021147127  0 | 1.661120046  3.322240092  0  0  0  3.322240092  1.661120046  0  1.661120046 | 3.509189128  3.509189128  0  0  1.754594564  1.754594564  5.263783691  3.509189128  3.509189128 | 1.357711801  5.430847205  0  0  0  1.357711801  2.715423602  0.6788559006  3.394279503 | 5.297342272  4.635174488  0  0  0.6621677839  5.297342272  1.986503352  1.324335568  2.648671136 |
| 18716 ENSG00000259385 0.9344383177 2.792904568 2.128115427 1.312383968 0.1893906135 0.3909337155 0 0 0 0 0 0 0 0 1.661120046 3.509189128 3.394279503 2.648671136 | | | | | | | | | | | | | | | | | | | |
| 18721 | ENSG00000269903 | 1.354442168 | 2.494478938 | 1.901666885 | 1.311732858 | 0.1896102861 | 0.391303531 | 0 | 0 | 0 | 1.182214954 | 0 | 0 | 0 | 0.7021147127 | 0 | 7.018378255 | 2.715423602 | 4.635174488 |

| 18722  18723  18730  18734  18737  18739  18750  18755 | ENSG00000104760 ENSG00000148948 ENSG00000236733 ENSG00000158113 ENSG00000186075 ENSG00000166866 ENSG00000249937 ENSG00000268873 | 0.95376405  0.95376405  0.6302184324  1.330696755  0.9252580981  0.9533234713  0.570912898  0.570912898 | 2.857163713  2.857163713  2.271583004  2.505685023  2.782031238  2.798782659  2.006887658  2.006887658 | 2.178321571  2.178321571  1.733893057  1.913285878  2.124762071  2.138181836  1.535272869  1.535272869 | 1.311635413  1.311635413  1.310105601  1.309623958  1.309337773  1.308954464  1.307186298  1.307186298 | 0.1896431784  0.1896431784  0.1901601141  0.1903230796  0.1904199599  0.1905497761  0.1911494478  0.1911494478 | 0.3913296052  0.3913296052  0.3922496533  0.3925019847  0.3926389043  0.3928722112  0.3937471265  0.3937471265 | 0  0  0  0  0  0  0  0 | 0  0  0  0  0  0  0  0 | 0  0  0  0.9913102377  0  0  0  0 | 0  0  0  0  0  0  0  0 | 0  0  0.7299339855  0  0  0  0.7299339855  0.7299339855 | 0  0  1.038333469  1.038333469  0  0  0  0 | 0  0  0  0  0  0  0  0 | 0  0  0  0  0  0  0.7021147127  0.7021147127 | 1.661120046  1.661120046  0  0  3.322240092  4.983360138  1.661120046  1.661120046 | 1.754594564  1.754594564  1.754594564  5.263783691  1.754594564  1.754594564  1.754594564  1.754594564 | 3.394279503  3.394279503  2.715423602  2.715423602  2.715423602  2.715423602  0.6788559006  0.6788559006 | 4.635174488  4.635174488  1.324335568  5.959510055  3.31083892  1.986503352  1.324335568  1.324335568 |
| --- | --- | --- | --- | --- | --- | --- | --- | --- | --- | --- | --- | --- | --- | --- | --- | --- | --- | --- | --- |
| 18764 ENSG00000271366 0.6371680556 2.243179209 1.717696174 1.305923156 0.1915786911 0.394459749 0 0 0 0 0 2.076666938 1.841832474 1.404229425 1.661120046 0 0 0.6621677839 | | | | | | | | | | | | | | | | | | | |
| 18786  18789  18791  18800  18801  18814  18833  18837  18843 | ENSG00000244155 ENSG00000205642 ENSG00000249352 ENSG00000239959 ENSG00000251172 ENSG00000259669 ENSG00000228503 ENSG00000152213 ENSG00000269155 | 0.5693412943  2.137602294  0.970293234  0.6012359927  0.8879458728  0.9661212048  0.8343825134  0.9593267556  1.003366798 | 2.206788534  2.28854169  2.8201023  2.255054116  2.76383024  2.813755151  2.705256252  2.864716496  2.845059782 | 1.694554879  1.758199434  2.16672188  1.733334713  2.124322688  2.165722345  2.085829123  2.209346311  2.196242306 | 1.302282128  1.301639419  1.301552509  1.300991724  1.301040683  1.299222478  1.296969259  1.296635336  1.295421627 | 0.1928199588  0.193039678  0.1930694033  0.1932612875  0.1932445297  0.1938675895  0.1946417643  0.1947566882  0.1951748213 | 0.3965505753  0.396917933  0.3969475585  0.3971318025  0.3971318025  0.3981117424  0.399298283  0.3994367365  0.4001793357 | 0  0  0  0  0  0  0  0  0 | 0  0  0  0  0  0  0  0  0 | 0  0  0  0  0  0  0  0  0 | 0  2.364429908  0  0  0  0  0  0  0 | 0.7299339855  0  0  0.7299339855  2.919735942  0  2.919735942  0  0 | 1.038333469  0  0  2.076666938  5.191667345  0  1.038333469  0  0 | 0.9209162369  0.9209162369  0  0.9209162369  1.841832474  0  1.841832474  0  0 | 2.106344138  0  0  2.808458851  0.7021147127  0  4.212688276  0  0 | 0  9.966720275  1.661120046  0  0  1.661120046  0  1.661120046  1.661120046 | 0  7.018378255  5.263783691  0  0  5.263783691  0  1.754594564  7.018378255 | 2.036567702  3.394279503  3.394279503  0.6788559006  0  1.357711801  0  6.109703105  2.036567702 | 0  1.986503352  1.324335568  0  0  3.31083892  0  1.986503352  1.324335568 |
| 18845 ENSG00000189056 0.8286321336 2.696410421 2.082606647 1.294728615 0.1954138649 0.4006269393 0 0 0 0 4.379603913 2.076666938 0 2.808458851 0 0 0.6788559006 0 | | | | | | | | | | | | | | | | | | | |
| 18851  18855  18860  18863  18885  18898  18907  18909  18915 | ENSG00000275869 ENSG00000279686 ENSG00000206142 ENSG00000253084 ENSG00000252699 ENSG00000102032 ENSG00000084453 ENSG00000259783 ENSG00000131401 | 0.9509826972  0.9948255711  1.259676424  0.9925072984  1.122767787  1.080714333  0.6994231778  1.026365931  1.419657098 | 2.852923189  2.837495573  2.406181591  2.833951647  2.165123179  2.263217942  2.367618899  2.014849951  3.427237331 | 2.205927221  2.194396748  1.861603231  2.193317175  1.678381889  1.757088106  1.839348275  1.565734285  2.665797309 | 1.293298873  1.293064062  1.292531916  1.292084737  1.290006281  1.288050345  1.287205328  1.286840284  1.285633127 | 0.1959077103  0.1959889034  0.1961730006  0.1963278009  0.1970484772  0.1977284386  0.1980227307  0.1981499629  0.1985711297 | 0.4015115585  0.4016051767  0.4018634081  0.4021165555  0.4031630011  0.4042352749  0.4046442164  0.404861379  0.4055932116 | 0  0  0  0  0  0  0  0  0 | 0  0  0.917401664  0  0.917401664  0  0  0  0 | 0  0  0  0  0  0.9913102377  0  0  0 | 0  0  0  0  0  0  0  1.182214954  0 | 0  0.7299339855  0  0  0.7299339855  4.379603913  0  0.7299339855  0 | 0  0  0  0  3.115000407  0  0  2.076666938  0 | 0  0  0  0  0  2.762748711  0.9209162369  0  0 | 0  0  0  0.7021147127  1.404229425  3.510573564  0  0  0 | 1.661120046  4.983360138  3.322240092  4.983360138  6.644480183  0  1.661120046  4.983360138  4.983360138 | 1.754594564  3.509189128  3.509189128  3.509189128  0  0  1.754594564  0  0 | 2.036567702  2.715423602  3.394279503  2.715423602  0  0  3.394279503  1.357711801  5.430847205 | 5.959510055  0  3.973006704  0  0.6621677839  1.324335568  0.6621677839  1.986503352  6.621677839 |
| 18918 ENSG00000254153 0.8752376153 2.759252638 2.147064787 1.285127796 0.1987476297 0.4058893473 0 0 0 0 0 0 0 0.7021147127 0 1.754594564 4.073135404 3.973006704 | | | | | | | | | | | | | | | | | | | |
| 18923  18927  18935  18939  18953  18958  18968  18969  18974 | ENSG00000139144 ENSG00000183644 ENSG00000269392 ENSG00000263697 ENSG00000241359 ENSG00000250488 ENSG00000275327 ENSG00000278647 ENSG00000140481 | 94.15100824  0.8554488029  1.126301017  0.8531305302  0.9224767454  0.9569409853  0.89371944  2.563611806  1.825495689 | 2.229452329  2.688391598  2.142782568  2.684786774  2.778730964  2.803912073  2.786621511  2.034344919  2.209174951 | 1.735494027  2.093390721  1.669345853  2.092075945  2.167548563  2.188564013  2.176266021  1.589223488  1.726269345 | 1.284621148  1.284228296  1.283606129  1.283312291  1.281969415  1.28116521  1.280459964  1.280087373  1.279739432 | 0.1989247049  0.1990620874  0.1992798043  0.1993826881  0.1998533754  0.2001356431  0.2003834166  0.2005144092  0.2006367916 | 0.4061436333  0.4063382336  0.4066107863  0.4067347883  0.4073938246  0.407861619  0.4081512706  0.4083965522  0.4085381281 | 29.10066875  0  0  0  0  0  0  1.265246467  1.265246467 | 22.9350416  0  0  0  0  0  0  0.917401664  0 | 36.6784788  0  0.9913102377  0  0  0  0  0  0.9913102377 | 20.09765422  0  0  0  0  0  0  1.182214954  0 | 0  0.7299339855  1.459867971  0  0  0  4.379603913  0  2.919735942 | 0  0  0  0  0  0  1.038333469  0  2.076666938 | 0  0  0  0  0  0  4.604581185  0  5.525497422 | 0  0  0  0.7021147127  0  0  0.7021147127  0  9.127491265 | 269.1014474  0  6.644480183  0  3.322240092  3.322240092  0  4.983360138  0 | 261.43459  3.509189128  1.754594564  3.509189128  1.754594564  3.509189128  0  7.018378255  0 | 234.2052857  2.715423602  0.6788559006  2.715423602  1.357711801  0.6788559006  0  6.788559006  0 | 256.2589324  3.31083892  1.986503352  3.31083892  4.635174488  3.973006704  0  8.608181191  0 |
| 18975 ENSG00000264663 0.903151013 2.704856149 2.11382519 1.279602571 0.2006849451 0.4086146432 0 0 0 0 0 0 0 0 3.322240092 3.509189128 1.357711801 2.648671136 | | | | | | | | | | | | | | | | | | | |
| 18988  18993  19008  19028  19041  19053  19061  19062  19065 | ENSG00000282048 ENSG00000277595 ENSG00000117595 ENSG00000107187 ENSG00000231046 ENSG00000137948 ENSG00000116711 ENSG00000259925 ENSG00000212190 | 0.7795302723  0.7823834107  729.243239  0.8154952662  0.6497982775  0.8384790402  120.9731475  0.9390059293  0.6312417627 | 2.590518083  2.614399231  2.068186883  2.658855029  2.229925668  2.668373901  2.237839987  2.733980114  2.225537849 | 2.027416108  2.047142902  1.621745375  2.088782624  1.754205785  2.10138436  1.76381986  2.15495385  1.755018646 | 1.277743662  1.277096595  1.275284589  1.272920887  1.271188185  1.269817151  1.268746337  1.268695435  1.268099262 | 0.2013398246  0.2015681467  0.2022085306  0.2030461168  0.2036617087  0.2041497699  0.20453155  0.2045497111  0.2047625051 | 0.4096673754  0.4100239745  0.4109686493  0.4122273632  0.4132382813  0.4139503692  0.414567779  0.4145828395  0.4149488269 | 0  0  293.5371805  0  0  0  32.89640815  0  0 | 0  0  156.8756845  0  0  0  32.10905824  0  0 | 0  0  322.1758273  0  0  0  32.71323785  0  0 | 0  0  159.5990188  0  0  0  41.37752339  0  0 | 2.189801957  2.919735942  0.7299339855  1.459867971  0  0.7299339855  0  0  0 | 2.076666938  0  0  4.153333876  0  0  0  0  3.115000407 | 3.683664948  3.683664948  1.841832474  0  0.9209162369  0  0  0  0 | 1.404229425  2.106344138  0.7021147127  3.510573564  0.7021147127  0  0  0  0.7021147127 | 0  0  1790.687409  0  0  3.322240092  335.5462493  3.322240092  0 | 0  0  2186.224826  0  3.509189128  0  380.7470203  5.263783691  1.754594564 | 0  0.6788559006  1803.041272  0  0.6788559006  2.036567702  283.0829105  1.357711801  0.6788559006 | 0  0  2035.503768  0.6621677839  1.986503352  3.973006704  313.2053618  1.324335568  1.324335568 |
| 19067 ENSG00000240069 0.6400705 2.217485065 1.749136595 1.267759803 0.204883741 0.415150959 0 0 0 0 0 0 0.9209162369 0 1.661120046 1.754594564 1.357711801 1.986503352 | | | | | | | | | | | | | | | | | | | |
| 19082  19090  19095  19104  19106  19114  19125  19132  19142 | ENSG00000232237 ENSG00000183570 ENSG00000270068 ENSG00000237361 ENSG00000224549 ENSG00000184956 ENSG00000236409 ENSG00000109181 ENSG00000230251 | 0.9512726955  127.5562957  0.7701434631  0.8686867731  0.8967521462  0.6147970094  0.9360786566  1.367128671  0.8112004672 | 2.010766654  2.202538241  2.576104349  2.683198468  2.699411867  2.207148261  2.031185916  3.358279109  2.622680577 | 1.588392078  1.741207463  2.037407998  2.123903232  2.13725791  1.748430344  1.610309204  2.664379855  2.082101651 | 1.265913298  1.264948772  1.264402786  1.263333671  1.263025793  1.262359847  1.261363911  1.260435558  1.259631381 | 0.2055441249  0.2058896924  0.2060854936  0.2064692902  0.2065799103  0.2068193306  0.2071777644  0.2075122813  0.2078023697 | 0.4161616846  0.4166954758  0.4169737127  0.4175694196  0.4177334258  0.4180398386  0.4185261661  0.4190485567  0.419415137 | 0  31.63116169  0  0  0  0  0  0  0 | 0  36.69606656  0  0  0  0  0.917401664  0  0 | 0.9913102377  48.57420165  0  0  0  0  0  0  0 | 0  33.10201871  0  0  0  0  0  0  0 | 0  446.7195991  0  0  0  0.7299339855  1.459867971  0  2.189801957 | 1.038333469  250.2383661  0  0  0  1.038333469  1.038333469  0  0 | 2.762748711  419.0168878  0  0  0  1.841832474  0  0  3.683664948 | 3.510573564  264.6972467  2.106344138  0  0  2.106344138  1.404229425  0  2.106344138 | 0  0  0  3.322240092  4.983360138  1.661120046  1.661120046  0  0 | 1.754594564  0  1.754594564  1.754594564  1.754594564  0  0  7.018378255  1.754594564 | 1.357711801  0  3.394279503  2.036567702  2.036567702  0  4.751991304  4.751991304  0 | 0  0  1.986503352  3.31083892  1.986503352  0  0  4.635174488  0 |
| 19151 ENSG00000171234 1.210654823 2.329434505 1.85100151 1.258472504 0.2082209251 0.4200624219 0 0 0.9913102377 0 0 0 0 0 3.322240092 3.509189128 3.394279503 3.31083892 | | | | | | | | | | | | | | | | | | | |
| 19152  19160  19166  19182  19196  19220  19223  19261  19265 | ENSG00000168772 ENSG00000166111 ENSG00000227913 ENSG00000237672 ENSG00000221826 ENSG00000250891 ENSG00000272456 ENSG00000139874 ENSG00000154143 | 80.85496665  0.7418655741  0.6033351229  0.5987629006  5.639771881  2.143065629  1.236379422  0.9128316471  0.8463307283 | 2.205162445  2.320078041  2.184582487  2.174970694  2.052120324  2.460669583  2.394890109  2.747298995  2.703085808 | 1.752702489  1.845127402  1.738659004  1.734372776  1.638939885  1.969261546  1.917278862  2.208841369  2.173435301 | 1.258149891  1.257408046  1.256475526  1.254038765  1.252102254  1.249539243  1.24910891  1.243773787  1.243692787 | 0.208337553  0.2086059172  0.2089436139  0.2098279157  0.2105326066  0.2114679093  0.2116252423  0.2135828303  0.2136126515 | 0.4202757601  0.4206414201  0.421202425  0.4226125003  0.4237303217  0.4250813048  0.425331178  0.4284053316  0.4284061449 | 43.01837989  0  0  0  1.265246467  0  0  0  0 | 10.0914183  0  0  0  3.669606656  0  0  0  0 | 34.69585832  0  0  0  0  1.982620475  0.9913102377  0  0 | 7.093289725  0  0  0  2.364429908  0  0  0  0 | 0  0.7299339855  0  0  21.16808558  0  0  0  4.379603913 | 0  0  1.038333469  2.076666938  6.230000815  0  0  1.038333469  4.153333876 | 0  0  1.841832474  0  29.46931958  0  0  0  0.9209162369 | 0  0  0  2.106344138  3.510573564  0  0  0  0.7021147127 | 247.5068868  3.322240092  1.661120046  1.661120046  0  3.322240092  1.661120046  0  0 | 236.8702661  3.509189128  0  0  0  7.018378255  3.509189128  5.263783691  0 | 200.9413466  0.6788559006  2.036567702  0.6788559006  0  6.109703105  2.715423602  0.6788559006  0 | 190.042154  0.6621677839  0.6621677839  0.6621677839  0  7.283845623  5.959510055  3.973006704  0 |
| 19266 ENSG00000233725 0.9017603366 2.706744355 2.176905128 1.243391051 0.2137237665 0.4285678995 0 0 0 0 0 0 0 0 3.322240092 3.509189128 0.6788559006 3.31083892 | | | | | | | | | | | | | | | | | | | |
| 19267 | ENSG00000232010 | 0.9467954725 | 2.748868317 | 2.210739583 | 1.243415705 | 0.213714686 | 0.4285678995 | 0 | 0 | 0 | 0 | 0 | 0 | 0 | 0 | 1.661120046 | 7.018378255 | 1.357711801 | 1.324335568 |

| 19278  19282  19287  19294  19298  19306  19308  19316 | ENSG00000256943 ENSG00000204022 ENSG00000270954 ENSG00000270765 ENSG00000251303 ENSG00000189275 ENSG00000224747 ENSG00000213171 | 0.8636769671  0.6835304619  0.5712436304  0.8434027527  0.7327482119  0.7551663937  0.7551663937  0.8332822366 | 2.754721432  2.442308549  2.131707479  2.672585261  2.53637899  2.550317666  2.550317666  2.686640562 | 2.217433912  1.966593716  1.717342971  2.154347449  2.045880761  2.058732168  2.058732168  2.170363485 | 1.24230148  1.241897871  1.241282327  1.240554425  1.239749177  1.238780695  1.238780695  1.237875858 | 0.2141253494  0.2142742458  0.214501471  0.2147703968  0.2150681816  0.2154267249  0.2154267249  0.2157620952 | 0.4291281707  0.4293374902  0.429681357  0.4300639723  0.4305710019  0.4310775227  0.4310775227  0.4315395872 | 0  0  0  0  0  0  0  0 | 0  0  0  0  0  0  0  0 | 0  0  0  0  0  0  0  0 | 0  0  0  0  0  0  0  0 | 7.299339855  2.919735942  2.189801957  0  2.919735942  2.919735942  2.919735942  0 | 1.038333469  0  0  0  1.038333469  3.115000407  3.115000407  0 | 0  1.841832474  0.9209162369  0  0  0.9209162369  0.9209162369  0.9209162369 | 0.7021147127  1.404229425  1.404229425  0  3.510573564  2.106344138  2.106344138  0 | 0  0  1.661120046  1.661120046  0  0  0  1.661120046 | 0  0  0  1.754594564  0  0  0  0 | 0  2.036567702  0.6788559006  3.394279503  0  0  0  5.430847205 | 1.324335568  0  0  3.31083892  1.324335568  0  0  1.986503352 |
| --- | --- | --- | --- | --- | --- | --- | --- | --- | --- | --- | --- | --- | --- | --- | --- | --- | --- | --- | --- |
| 19318 ENSG00000250862 0.8347323942 2.606597819 2.105814285 1.237809923 0.2157865481 0.4315395872 0 0 0 0 0.7299339855 0 0 0 0 5.263783691 2.036567702 1.986503352 | | | | | | | | | | | | | | | | | | | |
| 19319  19322  19325  19330  19339  19345  19350  19358  19360 | ENSG00000054938 ENSG00000283365 ENSG00000118849 ENSG00000224758 ENSG00000235192 ENSG00000275649 ENSG00000186479 ENSG00000275512 ENSG00000101746 | 0.7785480779  0.8420120763  3.727996225  0.5615440653  1.879849181  0.8317860049  1.269782626  0.5537545221  0.8493610407 | 2.585257169  2.670342039  2.149901759  2.103014912  2.15518649  2.637780291  2.39726402  2.099333624  2.601722581 | 2.088482661  2.158589839  1.738716564  1.701290367  1.745051253  2.137163696  1.94349194  1.703314179  2.111496584 | 1.237863841  1.237077091  1.236487765  1.236129325  1.235027617  1.234243449  1.233482872  1.232499353  1.232169922 | 0.2157665517  0.2160584638  0.2162773109  0.2164104961  0.2168202263  0.2171122015  0.2173956633  0.2177626081  0.2178856164 | 0.4315395872  0.4320162896  0.432386748  0.4325411027  0.4331583557  0.4336071287  0.4340451477  0.4346140286  0.4348146069 | 0  0  1.265246467  0  0  0  0  0  0 | 0  0  0  0  0  0  0  0  0 | 0  0  1.982620475  0  0.9913102377  0  0  0  0 | 0  0  1.182214954  0  1.182214954  0  1.182214954  0  0 | 1.459867971  0  0  0  0  0  0  0  0 | 4.153333876  0  1.038333469  0  0  4.153333876  0  0  0 | 0.9209162369  0  0  0.9209162369  0  2.762748711  0  0.9209162369  0 | 2.808458851  0  0  0.7021147127  0  1.404229425  0  0.7021147127  0 | 0  1.661120046  19.93344055  0  6.644480183  1.661120046  4.983360138  1.661120046  3.322240092 | 0  1.754594564  14.03675651  1.754594564  7.018378255  0  1.754594564  0  3.509189128 | 0  2.715423602  0  2.036567702  4.073135404  0  1.357711801  2.036567702  2.036567702 | 0  3.973006704  5.297342272  1.324335568  2.648671136  0  5.959510055  1.324335568  1.324335568 |
| 19361 ENSG00000145794 0.8871316901 2.70861674 2.198364099 1.232105611 0.2179096358 0.4348400795 0 0 0 0 0.7299339855 0 0 0 0 5.263783691 0.6788559006 3.973006704 | | | | | | | | | | | | | | | | | | | |
| 19367  19369  19374  19376  19377  19406  19409  19414  19421 | ENSG00000231359 ENSG00000159708 ENSG00000129451 ENSG00000258435 ENSG00000060566 ENSG00000166317 ENSG00000271850 ENSG00000243307 ENSG00000183632 | 0.8479703644  0.8479703644  1.027117614  0.5459723024  0.735596855  0.5675705795  0.8406213999  0.8441374718  0.822686344 | 2.599208666  2.599208666  2.019207892  2.113508836  2.521333411  2.193527503  2.668046387  2.672191257  2.59035044 | 2.11059901  2.11059901  1.640102041  1.717242415  2.048585955  1.786987421  2.174006635  2.179495835  2.11668418 | 1.231502836  1.231502836  1.231147722  1.230757415  1.230767694  1.227500248  1.227248503  1.226059354  1.223777484 | 0.2181348567  0.2181348567  0.2182676198  0.218413607  0.2184097617  0.2196346286  0.2197292045  0.2201763399  0.2210361805 | 0.4351097212  0.4351097212  0.4352621807  0.4354858703  0.4354858703  0.4372209335  0.4373866669  0.4381638452  0.4397164325 | 0  0  0  0  0  0  0  0  0 | 0  0  0  0  0  0  0  0  0 | 0  0  0  0  0  0  0  0  0 | 0  0  1.182214954  0  0  0  0  0  0 | 0  0  0  2.189801957  2.919735942  0  0  0  0 | 0  0  2.076666938  2.076666938  1.038333469  1.038333469  0  4.153333876  0 | 0  0  0  0.9209162369  2.762748711  0.9209162369  0  0.9209162369  0 | 0  0  0  0.7021147127  2.106344138  3.510573564  0  0  0 | 3.322240092  3.322240092  3.322240092  0  0  0  1.661120046  1.661120046  1.661120046 | 3.509189128  3.509189128  1.754594564  0  0  0  1.754594564  0  3.509189128 | 1.357711801  1.357711801  0.6788559006  0  0  0.6788559006  2.036567702  3.394279503  2.715423602 | 1.986503352  1.986503352  3.31083892  0.6621677839  0  0.6621677839  4.635174488  0  1.986503352 |
| 19446 ENSG00000271524 0.7382719789 2.501678155 2.048202975 1.221401485 0.2219340452 0.440934991 0 0 0 0 1.459867971 4.153333876 1.841832474 1.404229425 0 0 0 0 | | | | | | | | | | | | | | | | | | | |
| 19452  19455  19459  19482  19484  19490  19496  19499  19510 | ENSG00000241622 ENSG00000231863 ENSG00000269696 ENSG00000249906 ENSG00000272181 ENSG00000205634 ENSG00000283141 ENSG00000162383 ENSG00000172073 | 0.8127544411  1.252724476  0.8135061244  1.226546303  2.1138797  0.5234180118  0.7057825949  0.727148164  0.6079789996 | 2.580770989  3.243870627  2.578194453  3.228430333  2.339925379  2.075981523  2.455962056  2.506086477  2.189910761 | 2.114214115  2.657631986  2.112824481  2.650376263  1.921230946  1.705126503  2.01878515  2.060800455  1.803407569 | 1.220676265  1.22058684  1.220259646  1.218102643  1.217930298  1.217494139  1.21655445  1.216074303  1.214318271 | 0.2222086179  0.2222424916  0.2223664623  0.2231849695  0.2232504615  0.2234162652  0.2237737825  0.2239566185  0.2246262114 | 0.4413155756  0.4413208606  0.4414989604  0.4426009289  0.4426853613  0.4428777529  0.4434499428  0.4437439846  0.4448151515 | 0  0  0  0  2.530492935  0  0  0  0 | 0  0  0  0  0  0  0  0  0 | 0  0  0  0  0  0  0  0  0 | 0  0  0  0  0  0  0  0  0 | 0.7299339855  0  0  2.919735942  4.379603913  0  0  2.919735942  2.919735942 | 0  0  0  0  7.268334284  1.038333469  0  2.076666938  1.038333469 | 0  0  0  8.288246132  2.762748711  1.841832474  0  0.9209162369  0.9209162369 | 0  0  0  3.510573564  8.425376553  0.7021147127  2.106344138  2.808458851  0 | 4.983360138  4.983360138  3.322240092  0  0  0  1.661120046  0  0 | 0  0  1.754594564  0  0  0  0  0  1.754594564 | 2.715423602  4.751991304  2.036567702  0  0  2.036567702  2.715423602  0  0 | 1.324335568  5.297342272  2.648671136  0  0  0.6621677839  1.986503352  0  0.6621677839 |
| 19521 ENSG00000281887 0.7187024402 2.471493289 2.0370829 1.213251208 0.2250337912 0.4453940559 0 0 0 0 1.459867971 2.076666938 3.683664948 1.404229425 0 0 0 0 | | | | | | | | | | | | | | | | | | | |
| 19526  19535  19536  19537  19541  19548  19566  19574  19582 | ENSG00000235267 ENSG00000204165 ENSG00000285171 ENSG00000214814 ENSG00000160791 ENSG00000226770 ENSG00000262873 ENSG00000213994 ENSG00000211694 | 0.6377993628  0.8240770204  0.8240770204  0.8240770204  0.8736949635  1.241628866  0.506231069  0.6298982298  0.8401808212 | 2.168574763  2.593868409  2.593868409  2.593868409  2.690926018  3.237848009  2.051509237  2.150131629  2.592561794 | 1.788973559  2.142131245  2.142131245  2.142131245  2.222585446  2.675194579  1.698744891  1.781563684  2.149579621 | 1.212189388  1.210882113  1.210882113  1.210882113  1.210718815  1.210322432  1.207661755  1.206878905  1.206078514 | 0.2254398928  0.2259405888  0.2259405888  0.2259405888  0.2260031888  0.2261551933  0.2271773946  0.2274787826  0.2277872186 | 0.4460652596  0.4467724898  0.4467724898  0.4467724898  0.446836559  0.4469960001  0.4486020818  0.4489957478  0.4494208555 | 0  0  0  0  0  0  0  0  0 | 0  0  0  0  0  0  0  0  0 | 0  0  0  0  0  0  0  0  0 | 0  0  0  0  0  0  0  0  0 | 0  0  0  0  0  0  2.189801957  0  0 | 1.038333469  0  0  0  0  0  0  2.076666938  0 | 1.841832474  0  0  0  0  0  1.841832474  0  0 | 0  0  0  0  0  0  0.7021147127  1.404229425  0 | 1.661120046  1.661120046  1.661120046  1.661120046  1.661120046  0  0  1.661120046  4.983360138 | 1.754594564  3.509189128  3.509189128  3.509189128  3.509189128  3.509189128  0  1.754594564  1.754594564 | 1.357711801  3.394279503  3.394279503  3.394279503  0.6788559006  5.430847205  0.6788559006  0  1.357711801 | 0  1.324335568  1.324335568  1.324335568  4.635174488  5.959510055  0.6621677839  0.6621677839  1.986503352 |
| 19621 ENSG00000258527 0.6616254086 2.219879974 1.848735841 1.200755633 0.2298460068 0.4526032377 0 0 0 0 0.7299339855 3.115000407 0 0 1.661120046 1.754594564 0.6788559006 0 | | | | | | | | | | | | | | | | | | | |
| 19627  19630  19642  19651  19690  19707  19722  19724  19725 | ENSG00000229996 ENSG00000182557 ENSG00000267142 ENSG00000213424 ENSG00000206034 ENSG00000211691 ENSG00000231646 ENSG00000185559 ENSG00000280269 | 0.846579688  0.846579688  0.7610026932  0.788222104  1.722058901  0.9845886232  1.945371562  1.171903327  0.7225116185 | 2.601398062  2.601398062  2.501848564  2.569574704  2.100873041  2.795931786  2.152641904  2.24495302  2.496413203 | 2.167245219  2.167245219  2.087204805  2.145220496  1.761198997  2.347914826  1.809375488  1.887288053  2.099238872 | 1.200324743  1.200324743  1.198659833  1.197813795  1.192865227  1.190814826  1.189715412  1.189512654  1.189199207 | 0.2300132438  0.2300132438  0.2306602424  0.2309895155  0.2329221596  0.2337262872  0.2341582656  0.2342379947  0.2343612864 | 0.4527094456  0.4527094456  0.4536991378  0.4541387171  0.4570313681  0.4581903342  0.4587113169  0.4588209757  0.459039204 | 0  0  0  0  1.265246467  0  1.265246467  1.265246467  0 | 0  0  0  0  0.917401664  0  0  0  0 | 0  0  0  0  0  0  0  0  0 | 0  0  0  0  0  0  1.182214954  0  0 | 0  0  0  0  0  0  0  0  1.459867971 | 0  0  0  0  0  0  0  0  2.076666938 | 0  0  0.9209162369  0  0  0  0  0  0.9209162369 | 0  0  0  0  0.7021147127  0.7021147127  0  0  4.212688276 | 3.322240092  3.322240092  0  1.661120046  0  1.661120046  1.661120046  4.983360138  0 | 3.509189128  3.509189128  3.509189128  1.754594564  7.018378255  8.772972819  10.52756738  1.754594564  0 | 0.6788559006  0.6788559006  2.715423602  3.394279503  6.788559006  0.6788559006  4.073135404  4.073135404  0 | 2.648671136  2.648671136  1.986503352  2.648671136  3.973006704  0  4.635174488  1.986503352  0 |
| 19726 ENSG00000251665 0.7854407513 2.564814099 2.157028012 1.189049973 0.2344200029 0.4591309343 0 0 0 0 0 0 0 0 1.661120046 1.754594564 2.036567702 3.973006704 | | | | | | | | | | | | | | | | | | | |
| 19763  19773  19775  19778  19779  19808  19818  19821  19827 | ENSG00000238118 ENSG00000153707 ENSG00000231704 ENSG00000165863 ENSG00000259070 ENSG00000268985 ENSG00000178462 ENSG00000235821 ENSG00000256824 | 0.6943385616  1.702630478  0.5915476728  0.5804292189  0.7885278397  0.842962174  2.20713568  1.03384953  0.5663952659 | 2.428930848  2.091548301  2.071323191  2.062939307  2.511858224  2.601586548  2.396943788  2.148409746  2.05359658 | 2.050130801  1.767510492  1.75050341  1.743845484  2.123344196  2.206206251  2.035034439  1.82426476  1.744406534 | 1.184768722  1.183330063  1.183272868  1.182982853  1.182972703  1.179212753  1.177839423  1.177685275  1.177246554 | 0.2361089027  0.2366783629  0.2367010221  0.236815943  0.2368199659  0.2383134689  0.2388606289  0.2389220992  0.2390971124 | 0.461573013  0.4624522606  0.4624615361  0.4625651861  0.4625651861  0.4648243575  0.4656329986  0.4656941245  0.4659059332 | 0  0  0  0  0  0  0  0  0 | 0  0.917401664  0  0  0  0  0  0  0 | 0  0  0  0  0  0  0  0  0 | 0  1.182214954  0  0  0  0  2.364429908  1.182214954  0 | 2.189801957  0  0  0  1.459867971  0  0  4.379603913  1.459867971 | 3.115000407  0  0  0  0  0  0  3.115000407  1.038333469 | 0.9209162369  0  0.9209162369  0.9209162369  0  0  0  0.9209162369  1.841832474 | 2.106344138  0  1.404229425  0.7021147127  0.7021147127  0  0  2.808458851  0.7021147127 | 0  1.661120046  1.661120046  3.322240092  0  4.983360138  4.983360138  0  0 | 0  5.263783691  1.754594564  0  5.263783691  1.754594564  1.754594564  0  1.754594564 | 0  6.109703105  1.357711801  1.357711801  2.036567702  2.715423602  6.788559006  0  0 | 0  5.297342272  0  0.6621677839  0  0.6621677839  10.59468454  0  0 |
| 19833 ENSG00000132464 0.5428475249 2.063925589 1.754068308 1.176650635 0.2393349785 0.466204845 0 0 0 0 0.7299339855 0 0 0.7021147127 0 1.754594564 0.6788559006 2.648671136 | | | | | | | | | | | | | | | | | | | |
| 19843 | ENSG00000253508 | 0.7576843044 | 2.495255071 | 2.122384836 | 1.175684555 | 0.2397209528 | 0.4667657545 | 0 | 0 | 0 | 0 | 1.459867971 | 3.115000407 | 2.762748711 | 0 | 0 | 1.754594564 | 0 | 0 |

| 19880  19895  19897  19904  19906  19907  19910  19911 | ENSG00000124469 ENSG00000258922 ENSG00000242082 ENSG00000120563 ENSG00000205644 ENSG00000280360 ENSG00000277218 ENSG00000223823 | 0.8185143148  0.7306035352  1.112214902  0.7583254758  0.5453731214  0.594090449  0.594090449  1.165306425 | 2.586882051  2.457246551  2.182324824  2.463855154  2.024717524  2.120065657  2.120065657  3.148774042 | 2.206985616  2.098735075  1.864998008  2.107224858  1.732413164  1.814174869  1.814174869  2.694619684 | 1.172133625  1.170822644  1.170148609  1.16924169  1.168726702  1.168611523  1.168611523  1.168541172 | 0.2411434117  0.2416700737  0.2419411698  0.2423062687  0.2425137602  0.2425601833  0.2425601833  0.242588542 | 0.4686406293  0.4693100427  0.4697892695  0.4703327317  0.4706881907  0.4706960061  0.4706960061  0.4707010685 | 0  0  1.265246467  0  0  0  0  0 | 0  0  0  0  0  0  0  0 | 0  0  0  0  0  0  0  0 | 0  0  0  0  0  0  0  0 | 0  0.7299339855  0  0  0  0.7299339855  0.7299339855  0 | 0  0  1.038333469  0  1.038333469  3.115000407  3.115000407  0 | 0  0  0  0  1.841832474  0.9209162369  0.9209162369  0 | 0  1.404229425  0  0  0  0.7021147127  0.7021147127  0 | 1.661120046  3.322240092  4.983360138  3.322240092  1.661120046  1.661120046  1.661120046  3.322240092 | 3.509189128  0  0  1.754594564  0  0  0  0 | 0.6788559006  0  4.073135404  2.036567702  0.6788559006  0  0  2.715423602 | 3.973006704  3.31083892  1.986503352  1.986503352  1.324335568  0  0  7.946013407 |
| --- | --- | --- | --- | --- | --- | --- | --- | --- | --- | --- | --- | --- | --- | --- | --- | --- | --- | --- | --- |
| 19918 ENSG00000279791 0.5800931176 2.027323064 1.735691743 1.168020227 0.2427986059 0.4709334877 0 0 0 0 0.7299339855 0 0 0.7021147127 0 3.509189128 1.357711801 0.6621677839 | | | | | | | | | | | | | | | | | | | |
| 19919  19929  19933  19938  19970  19997  20000  20009  20024 | ENSG00000280432 ENSG00000197408 ENSG00000226242 ENSG00000272202 ENSG00000243967 ENSG00000205022 ENSG00000168925 ENSG00000148541 ENSG00000164744 | 0.5800931176  0.566656391  2.336952974  0.5402533412  0.701448207  0.7250295292  0.6616553428  1.12418687  0.5725280779 | 2.027323064  2.012864072  2.045926039  2.012858605  2.461848383  2.438551576  2.37549617  2.164789445  2.077898251 | 1.735691743  1.725725801  1.754842089  1.727093357  2.118002171  2.103002164  2.049965417  1.869698813  1.798107783 | 1.168020227  1.166386961  1.165874726  1.165460221  1.162344598  1.159557331  1.158798168  1.157827897  1.155602724 | 0.2427986059  0.2434580281  0.243665099  0.243832753  0.2450955177  0.2462290819  0.2465384643  0.2469342764  0.2478436935 | 0.4709334877  0.471975559  0.4722822004  0.472472246  0.4741496051  0.4757243877  0.4762506784  0.4767769  0.478198217 | 0  0  0  0  0  0  0  0  0 | 0  0  1.834803328  0  0  0  0  0  0 | 0  0  0.9913102377  0  0  0  0  0.9913102377  0 | 0  0  0  0  0  0  0  0  0 | 0.7299339855  0  4.379603913  0  3.649669928  0  2.189801957  0  0.7299339855 | 0  0  7.268334284  2.076666938  1.038333469  2.076666938  0  0  3.115000407 | 0  0  0  0  0.9209162369  2.762748711  3.683664948  0  0 | 0.7021147127  0.7021147127  0  1.404229425  2.808458851  2.106344138  1.404229425  0  0.7021147127 | 0  1.661120046  8.305600229  1.661120046  0  0  0  4.983360138  1.661120046 | 3.509189128  1.754594564  5.263783691  0  0  1.754594564  0  3.509189128  0 | 1.357711801  1.357711801  0  0.6788559006  0  0  0  1.357711801  0 | 0.6621677839  1.324335568  0  0.6621677839  0  0  0.6621677839  2.648671136  0.6621677839 |
| 20031 ENSG00000163207 1.165820195 2.246057843 1.945343512 1.15458161 0.2482618012 0.478815913 0 0 0 1.182214954 0 0 0 0 3.322240092 3.509189128 0.6788559006 5.297342272 | | | | | | | | | | | | | | | | | | | |
| 20039  20045  20060  20070  20085  20096  20113  20123  20124 | ENSG00000259364 ENSG00000267526 ENSG00000082929 ENSG00000263177 ENSG00000244222 ENSG00000230013 ENSG00000227076 ENSG00000284719 ENSG00000116014 | 0.8005792589  1.002353838  0.803951735  0.6506019462  0.7913990393  0.6742700633  1.658596519  0.7302601026  0.7302601026 | 2.500041313  2.116426083  2.551241983  2.335855773  2.486462059  2.393228418  2.158570379  2.452611278  2.452611278 | 2.166805868  1.835260606  2.214900748  2.029632558  2.16274842  2.083420361  1.881769666  2.139976799  2.139976799 | 1.153791094  1.153201936  1.151853863  1.150876184  1.149676974  1.148701656  1.147095959  1.146092462  1.146092462 | 0.2485858263  0.2488275093  0.2493811297  0.249783177  0.2502769414  0.2506790229  0.2513419639  0.2517568962  0.2517568962 | 0.4792710913  0.4795934558  0.4802953976  0.4808357272  0.481402451  0.48193591  0.4828020074  0.483334709  0.483334709 | 0  0  0  0  0  0  0  0  0 | 0  0.917401664  0  0  0  0  1.834803328  0  0 | 0  0  0  0  0  0  0  0  0 | 0  0  0  0  0  0  0  0  0 | 0  2.189801957  3.649669928  0  0  0  4.379603913  0  0 | 0  5.191667345  0  0  0  2.076666938  6.230000815  0  0 | 0  0.9209162369  0.9209162369  0  0  1.841832474  1.841832474  0  0 | 0  2.808458851  0  2.106344138  0  3.510573564  5.616917702  0  0 | 1.661120046  0  3.322240092  1.661120046  3.322240092  0  0  1.661120046  1.661120046 | 5.263783691  0  1.754594564  0  3.509189128  0  0  1.754594564  1.754594564 | 1.357711801  0  0  2.715423602  0.6788559006  0  0  2.036567702  2.036567702 | 1.324335568  0  0  1.324335568  1.986503352  0.6621677839  0  3.31083892  3.31083892 |
| 20133 ENSG00000283991 0.9978767623 2.081607084 1.818624723 1.144605073 0.2523727897 0.484300538 1.265246467 0 0 0 4.379603913 0 0 0 1.661120046 0 1.357711801 3.31083892 | | | | | | | | | | | | | | | | | | | |
| 20147  20164  20175  20180  20204  20218  20223  20252  20268 | ENSG00000173662 ENSG00000246225 ENSG00000125337 ENSG00000235232 ENSG00000181013 ENSG00000197870 ENSG00000258051 ENSG00000225756 ENSG00000136315 | 0.4831591003  2.152851556  0.5703653401  0.5695222216  0.808676691  0.8116977127  0.7041627885  1.706309731  1.087053398 | 2.002792117  2.307948448  2.02567832  2.023392382  2.531420398  2.522735203  2.353087905  2.0191159  3.012556985 | 1.751899554  2.023256366  1.777552837  1.776640411  2.230141268  2.226461416  2.077293593  1.787338999  2.670442101 | 1.143211729  1.140709841  1.139588246  1.138886839  1.135094191  1.133069357  1.132766169  1.129677079  1.128111702 | 0.2529506938  0.2539906899  0.2544578844  0.2547503554  0.2563358553  0.2571851287  0.2573124621  0.2586123189  0.2592727475 | 0.4850722219  0.4866559365  0.4872852721  0.4877244788  0.4901769833  0.4914604534  0.4916097266  0.4933336761  0.4942337577 | 0  2.530492935  0  0  0  0  0  1.265246467  0 | 0  0  0  0  0  0  0  0  0 | 0  0  0  0  0  0  0  0  0 | 0  0  0  0  0  0  0  1.182214954  0 | 0.7299339855  0  0.7299339855  0.7299339855  1.459867971  0  1.459867971  9.489141812  0 | 1.038333469  0  0  0  0  0  2.076666938  4.153333876  0 | 0  0  0  0  0.9209162369  0  0  3.683664948  0 | 0.7021147127  0  0  0.7021147127  0  0.7021147127  1.404229425  0.7021147127  0 | 0  3.322240092  1.661120046  1.661120046  6.644480183  0  0  0  0 | 0  10.52756738  1.754594564  1.754594564  0  7.018378255  3.509189128  0  7.018378255 | 0.6788559006  7.467414906  2.036567702  0  0.6788559006  1.357711801  0  0  2.715423602 | 2.648671136  1.986503352  0.6621677839  1.986503352  0  0.6621677839  0  0  3.31083892 |
| 20298 ENSG00000232136 0.7302427839 2.404587443 2.139158479 1.124081019 0.2609786567 0.4967440341 0 0 0 0 1.459867971 3.115000407 0 0 0 3.509189128 0.6788559006 0 | | | | | | | | | | | | | | | | | | | |
| 20303  20304  20305  20306  20309  20310  20330  20333  20340 | ENSG00000264217 ENSG00000230166 ENSG00000226003 ENSG00000256826 ENSG00000158516 ENSG00000234832 ENSG00000188280 ENSG00000223720 ENSG00000266575 | 1.410492785  1.410492785  0.7152447599  0.6845932498  0.6442776932  0.6442776932  1.030214189  1.15364292  0.725311748 | 2.602448648  2.602448648  2.422984751  2.317543115  2.316231288  2.316231288  2.139676733  2.187360875  2.385607071 | 2.316467796  2.316467796  2.157460596  2.064134809  2.06406563  2.06406563  1.909990035  1.952945643  2.131796077 | 1.12345557  1.12345557  1.12307254  1.122767324  1.122169399  1.122169399  1.120255443  1.120031621  1.119059696 | 0.2612440602  0.2612440602  0.2614066876  0.2615363267  0.2617904209  0.2617904209  0.2626049237  0.2627002872  0.2631146724 | 0.497102259  0.497102259  0.4973872137  0.4975848713  0.4979947273  0.4979947273  0.499052692  0.4991602615  0.4997755835 | 0  0  0  0  0  0  0  0  0 | 0.917401664  0.917401664  0  0  0  0  0.917401664  0  0 | 0  0  0  0  0  0  0  0.9913102377  0 | 0  0  0  0  0  0  0  0  0 | 4.379603913  4.379603913  0  1.459867971  2.189801957  2.189801957  0  0  0 | 8.306667753  8.306667753  1.038333469  0  2.076666938  2.076666938  0  0  1.038333469 | 0  0  3.683664948  1.841832474  2.762748711  2.762748711  0  0  0 | 0  0  2.106344138  1.404229425  0.7021147127  0.7021147127  0  0  0 | 3.322240092  3.322240092  0  0  0  0  1.661120046  6.644480183  4.983360138 | 0  0  1.754594564  3.509189128  0  0  1.754594564  3.509189128  0 | 0  0  0  0  0  0  3.394279503  2.036567702  1.357711801 | 0  0  0  0  0  0  4.635174488  0.6621677839  1.324335568 |
| 20358 ENSG00000254867 0.6347860441 2.072349414 1.85479681 1.11729188 0.2638695464 0.5007662798 0 0 0 0 0.7299339855 1.038333469 0 0 1.661120046 3.509189128 0.6788559006 0 | | | | | | | | | | | | | | | | | | | |
| 20365  20370  20396  20404  20427  20436  20448  20464  20467 | ENSG00000229312 ENSG00000206728 ENSG00000185518 ENSG00000279705 ENSG00000242540 ENSG00000227634 ENSG00000232675 ENSG00000163273 ENSG00000278333 | 1.018050228  0.6319525335  0.6213493174  0.6928761075  0.7095436939  0.9421379918  0.7031448271  0.6283625056  0.6283625056 | 2.955695686  2.292045159  2.052522394  2.384110564  2.346965181  2.004316811  2.338863352  2.286664416  2.286664416 | 2.649002313  2.054874187  1.845512481  2.145512584  2.11776089  1.810814015  2.115405838  2.072215763  2.072215763 | 1.115776936  1.115418731  1.112169337  1.111207914  1.108229542  1.106859564  1.105633402  1.103487608  1.103487608 | 0.2645176285  0.2646710263  0.2660653489  0.2664788655  0.2677626955  0.2683546509  0.2688852264  0.26981547  0.26981547 | 0.501799007  0.5019421314  0.5039926826  0.5046111101  0.5064133415  0.507334211  0.5080389634  0.5092713488  0.5092713488 | 0  0  0  0  0  1.265246467  0  0  0 | 0  0  0  0  0  0  0  0  0 | 0  0  0  0  0  0  0  0  0 | 0  0  0  0  0  0  0  0  0 | 4.379603913  2.189801957  0  0.7299339855  0  3.649669928  0  2.919735942  2.919735942 | 4.153333876  3.115000407  1.038333469  3.115000407  0  1.038333469  0  2.076666938  2.076666938 | 3.683664948  0.9209162369  0  0  0  1.841832474  0  1.841832474  1.841832474 | 0  0  0  2.808458851  0  3.510573564  0  0.7021147127  0.7021147127 | 0  0  3.322240092  1.661120046  1.661120046  0  3.322240092  0  0 | 0  0  1.754594564  0  3.509189128  0  1.754594564  0  0 | 0  1.357711801  0.6788559006  0  1.357711801  0  2.036567702  0  0 | 0  0  0.6621677839  0  1.986503352  0  1.324335568  0  0 |
| 20469 ENSG00000276294 0.6283625056 2.286664416 2.072215763 1.103487608 0.26981547 0.5092713488 0 0 0 0 2.919735942 2.076666938 1.841832474 0.7021147127 0 0 0 0 | | | | | | | | | | | | | | | | | | | |
| 20471  20476  20480  20489  20514  20517  20535  20536  20547 | ENSG00000278775 ENSG00000257743 ENSG00000261122 ENSG00000180251 ENSG00000281731 ENSG00000204710 ENSG00000282033 ENSG00000251165 ENSG00000249956 | 0.9941980279  1.026439176  0.6078107725  1.949658818  0.6671857755  1.006476608  0.5980260031  0.5980260031  1.031872749 | 2.92298626  2.94912088  2.249263464  2.253883598  2.356882771  2.051712013  2.232480523  2.232480523  2.932631423 | 2.649190727  2.673526823  2.039292862  2.045328265  2.143181563  1.866106591  2.033491838  2.033491838  2.674080182 | 1.103350631  1.103082585  1.102962456  1.10196668  1.099712135  1.099461318  1.097855659  1.097855659  1.096687917 | 0.2698749272  0.2699913027  0.2700434696  0.2704761575  0.2714575659  0.2715668974  0.2722675225  0.2722675225  0.2727778404 | 0.5093360272  0.5094363866  0.5094498973  0.5100222727  0.5112490524  0.5114032091  0.5122251525  0.5122251525  0.5128605706 | 0  0  0  0  0  0  0  0  0 | 0  0  0  0  0  0  0  0  0 | 0  0  0  0.9913102377  0  0.9913102377  0  0  0 | 0  0  0  1.182214954  0  0  0  0  0 | 2.189801957  0  2.189801957  0  0  0  2.189801957  2.189801957  0 | 6.230000815  0  2.076666938  0  0  0  1.038333469  1.038333469  0 | 0  0  0.9209162369  0  0.9209162369  0  1.841832474  1.841832474  0 | 3.510573564  0  2.106344138  0  0  0  2.106344138  2.106344138  0 | 0  4.983360138  0  0  0  3.322240092  0  0  0 | 0  0  0  10.52756738  1.754594564  1.754594564  0  0  7.018378255 | 0  2.036567702  0  4.073135404  1.357711801  2.036567702  0  0  2.715423602 | 0  5.297342272  0  6.621677839  3.973006704  3.973006704  0  0  2.648671136 |
| 20548 ENSG00000230635 0.6750794539 2.329604799 2.124150702 1.096722938 0.2727625261 0.5128605706 0 0 0 0 0 0 0 0 1.661120046 1.754594564 2.036567702 2.648671136 | | | | | | | | | | | | | | | | | | | |
| 20581 | ENSG00000164794 | 1.030482073 | 2.930819024 | 2.680522966 | 1.093375831 | 0.2742288209 | 0.5147869635 | 0 | 0 | 0 | 0 | 0 | 0 | 0 | 0 | 0 | 7.018378255 | 2.036567702 | 3.31083892 |

| 20636  20649  20654  20657  20658  20659  20670  20696 | ENSG00000232893 ENSG00000236546 ENSG00000188389 ENSG00000251687 ENSG00000246528 ENSG00000270164 ENSG00000154438 ENSG00000263586 | 0.6003442759  2.967474728  1.021505522  0.6778608067  0.5957077304  0.6778608067  0.9946271567  1.027389274 | 2.235979273  2.080490458  2.966542489  2.335636171  2.226929441  2.335636171  2.894919843  2.920373469 | 2.054711746  1.914903639  2.731969418  2.151631352  2.051403197  2.151631352  2.672771911  2.704865839 | 1.088220417  1.086472664  1.085862261  1.085518748  1.085563991  1.085518748  1.083115185  1.079674055 | 0.2764978134  0.2772699285  0.2775399356  0.2776919648  0.2776719383  0.2776919648  0.2787572997  0.2802873513 | 0.517665296  0.518756595  0.5191413085  0.5193198634  0.5193198634  0.5193198634  0.5210347495  0.5232364619 | 0  0  0  0  0  0  0  0 | 0  3.669606656  0  0  0  0  0  0 | 0  0  0  0  0  0  0  0 | 0  0  0  0  0  0  0  0 | 2.919735942  7.299339855  5.109537899  0  1.459867971  0  0  0 | 1.038333469  13.4983351  0  0  1.038333469  0  0  0 | 1.841832474  5.525497422  6.446413659  0  1.841832474  0  0  0 | 1.404229425  5.616917702  0.7021147127  0  2.808458851  0  0  0 | 0  0  0  1.661120046  0  1.661120046  0  8.305600229 | 0  0  0  1.754594564  0  1.754594564  5.263783691  0 | 0  0  0  3.394279503  0  3.394279503  2.036567702  2.036567702 | 0  0  0  1.324335568  0  1.324335568  4.635174488  1.986503352 |
| --- | --- | --- | --- | --- | --- | --- | --- | --- | --- | --- | --- | --- | --- | --- | --- | --- | --- | --- | --- |
| 20698 ENSG00000257137 0.6236945493 2.30200764 2.132453084 1.079511506 0.2803597675 0.5233210754 0 0 0 0 4.379603913 1.038333469 0 1.404229425 0 0 0 0.6621677839 | | | | | | | | | | | | | | | | | | | |
| 20700  20704  20711  20715  20732  20736  20742  20744  20757 | ENSG00000260249 ENSG00000284188 ENSG00000089558 ENSG00000211695 ENSG00000163492 ENSG00000185448 ENSG00000165617 ENSG00000135312 ENSG00000204709 | 0.7081530175  0.6492507195  0.9768212328  0.9615535932  0.7440079338  0.9822700019  1.00547667  0.94244423  0.9487558596 | 2.348367035  2.247500885  2.874471543  2.863160551  2.377552582  2.911971052  2.937117528  2.853979477  2.848182001 | 2.176767876  2.084795749  2.669676207  2.661425834  2.215243421  2.713539511  2.738570205  2.6612199  2.659188923 | 1.078832089  1.078043681  1.076711676  1.075799488  1.073269221  1.073126461  1.072500359  1.072432788  1.0710717 | 0.280662587  0.281014263  0.2816090945  0.2820169411  0.2831503407  0.2832143801  0.2834953528  0.2835256876  0.2841371916 | 0.5238357028  0.5243907481  0.525323131  0.5259664341  0.5276377472  0.5276557402  0.5280517581  0.5280570256  0.5288413259 | 0  0  0  0  0  0  0  0  0 | 0  0  0  0  0  0  0  0  0 | 0  0  0  0  0  0  0  0  0 | 0  0  0  0  0  0  0  0  0 | 0  0  0  0  0  0  0  4.379603913  0 | 0  0  0  0  0  0  6.230000815  0  0 | 0  0.9209162369  0  0  0  0  0.9209162369  5.525497422  0 | 0  0  0  0  0  0  4.914802989  1.404229425  0 | 1.661120046  0  4.983360138  0  1.661120046  0  0  0  3.322240092 | 3.509189128  3.509189128  0  3.509189128  5.263783691  1.754594564  0  0  0 | 0.6788559006  2.036567702  4.751991304  3.394279503  0.6788559006  4.073135404  0  0  4.751991304 | 2.648671136  1.324335568  1.986503352  4.635174488  1.324335568  5.959510055  0  0  3.31083892 |
| 20766 ENSG00000136155 1.102191906 3.007434364 2.810662022 1.070009251 0.2846151449 0.5295245171 0 0 0 0 0 0 0 0 0 10.52756738 2.036567702 0.6621677839 | | | | | | | | | | | | | | | | | | | |
| 20772  20781  20791  20802  20804  20827  20838  20852  20862 | ENSG00000231112 ENSG00000156574 ENSG00000064218 ENSG00000261963 ENSG00000266958 ENSG00000263883 ENSG00000237685 ENSG00000179284 ENSG00000231105 | 0.641031832  1.009618885  0.6319448555  0.5652164007  0.6133958032  0.6109848638  1.689077721  0.5543738578  0.6567375943 | 2.290384581  2.901345341  2.212135882  2.13571455  2.231622388  2.202135097  2.142445617  2.148578889  2.290670682 | 2.141871284  2.715171426  2.071648198  2.003808806  2.094917997  2.072931405  2.018072962  2.026814754  2.16461106 | 1.069338106  1.068568015  1.067814451  1.06582751  1.065255247  1.062328976  1.061629414  1.060076598  1.058236616 | 0.2849173461  0.2852643675  0.2856042183  0.2865016229  0.286760439  0.2880863644  0.2884039548  0.2891097534  0.2899475819 | 0.5299336446  0.5303493017  0.5307294285  0.5321118257  0.5325413172  0.5344223097  0.534719589  0.5356682967  0.5369631304 | 0  0  0  0  0  0  0  0  0 | 0  0  0  0  0  0  0.917401664  0  0 | 0  0  0  0  0  0  0.9913102377  0  0 | 0  0  0  0  0  0  0  0  0 | 0  0  0.7299339855  1.459867971  0  2.189801957  0  1.459867971  0 | 0  2.076666938  0  2.076666938  0  2.076666938  0  0  4.153333876 | 1.841832474  0  0  1.841832474  0.9209162369  0  0  1.841832474  0 | 3.510573564  0  0  1.404229425  0  1.404229425  0  0.7021147127  1.404229425 | 1.661120046  6.644480183  0  0  0  1.661120046  4.983360138  0  1.661120046 | 0  0  3.509189128  0  1.754594564  0  0  0  0 | 0.6788559006  3.394279503  1.357711801  0  2.036567702  0  5.430847205  0  0 | 0  0  1.986503352  0  2.648671136  0  7.946013407  2.648671136  0.6621677839 |
| 20905 ENSG00000181123 0.929870672 2.834627487 2.687122832 1.054893157 0.2914741924 0.538680001 0 0 0 0 0 2.076666938 2.762748711 6.319032415 0 0 0 0 | | | | | | | | | | | | | | | | | | | |
| 20912  20932  20939  20962  20971  20978  20983  21004  21036 | ENSG00000259017 ENSG00000284664 ENSG00000218596 ENSG00000186971 ENSG00000258919 ENSG00000162374 ENSG00000260156 ENSG00000109943 ENSG00000266964 | 0.5951623428  0.581666454  0.5802757776  0.5811315883  0.5801209503  0.6042155836  0.6449733314  1.07105942  0.5675346734 | 2.196197392  2.198663758  2.195821385  2.165204016  2.162974729  2.217490385  2.248222009  2.960954282  2.138687683 | 2.082749211  2.091273768  2.090464933  2.065238547  2.064510271  2.118482499  2.148474812  2.837947454  2.057046302 | 1.054470399  1.051351474  1.050398574  1.048403837  1.047693857  1.046735286  1.04642698  1.043343589  1.039688645 | 0.2916676059  0.2930971943  0.293534901  0.2944525866  0.2947796788  0.2952216846  0.2953639416  0.2967891899  0.2984845776 | 0.5388743162  0.5409807997  0.5415958031  0.5427046887  0.5430484881  0.5436812901  0.54386302  0.5459174611  0.5482076993 | 0  0  0  0  0  0  0  0  0 | 0  0  0  0  0  0  0  0  0 | 0  0  0  0  0  0  0  0  0 | 0  0  0  0  0  0  0  0  0 | 0  0.7299339855  0.7299339855  0.7299339855  1.459867971  0  2.919735942  0  2.189801957 | 0  0  0  2.076666938  2.076666938  0  0  0  2.076666938 | 0  2.762748711  2.762748711  2.762748711  2.762748711  0.9209162369  0  0.9209162369  1.841832474 | 0.7021147127  2.808458851  2.808458851  1.404229425  0  0  1.404229425  1.404229425  0.7021147127 | 0  0  0  0  0  1.661120046  1.661120046  0  0 | 1.754594564  0  0  0  0  0  1.754594564  10.52756738  0 | 2.036567702  0.6788559006  0  0  0  1.357711801  0  0  0 | 2.648671136  0  0.6621677839  0  0.6621677839  3.31083892  0  0  0 |
| 21038 ENSG00000276633 0.6189345874 2.23680817 2.151807299 1.039502083 0.2985712898 0.5483079086 0 0 0 0 0.7299339855 4.153333876 1.841832474 0.7021147127 0 0 0 0 | | | | | | | | | | | | | | | | | | | |
| 21047  21048  21081  21082  21120  21141  21149  21185  21195 | ENSG00000225606 ENSG00000095777 ENSG00000136487 ENSG00000204011 ENSG00000102445 ENSG00000252213 ENSG00000227367 ENSG00000251685 ENSG00000244502 | 0.6198988053  0.6198988053  0.8894031818  0.931051923  0.5507636751  0.5493012131  1.141726343  0.9201207757  0.539350387 | 2.193414939  2.193414939  2.753379084  2.827225187  2.139561705  2.104158458  2.293163755  2.75486839  2.11502967 | 2.112681909  2.112681909  2.660705531  2.732189841  2.076433015  2.046235903  2.23150993  2.692059642  2.06926921 | 1.038213529  1.038213529  1.034830443  1.034783581  1.030402469  1.02830688  1.027628748  1.023331113  1.02211431 | 0.2991706557  0.2991706557  0.3007481029  0.3007699925  0.3028211148  0.3038054956  0.3041244961  0.306151318  0.3067268011 | 0.5491475808  0.5491475808  0.5511789268  0.5511928972  0.5539533036  0.5552019924  0.5555747274  0.5583269374  0.5591125247 | 0  0  0  0  0  0  0  0  0 | 0  0  0  0  0  0  0  0  0 | 0  0  0  0  0  0  0.9913102377  0  0 | 0  0  0  0  0  0  0  0  0 | 0  0  0  0.7299339855  2.919735942  2.189801957  0  0  0 | 0  0  0  6.230000815  0  2.076666938  0  0  1.038333469 | 0  0  0  0  0.9209162369  0.9209162369  0  0  0 | 0  0  0  4.212688276  2.106344138  1.404229425  0  0  2.106344138 | 1.661120046  1.661120046  3.322240092  0  0  0  3.322240092  0  0 | 1.754594564  1.754594564  0  0  0  0  0  7.018378255  0 | 2.036567702  2.036567702  2.715423602  0  0  0  4.751991304  2.036567702  0.6788559006 | 1.986503352  1.986503352  4.635174488  0  0.6621677839  0  4.635174488  1.986503352  2.648671136 |
| 21199 ENSG00000254338 0.5395164437 2.086957566 2.042355508 1.021838538 0.3068573261 0.5592185281 0 0 0 0 2.189801957 1.038333469 1.841832474 1.404229425 0 0 0 0 | | | | | | | | | | | | | | | | | | | |
| 21200  21213  21214  21215  21216  21229  21230  21243  21257 | ENSG00000277901 ENSG00000234089 ENSG00000211892 ENSG00000280220 ENSG00000215834 ENSG00000260782 ENSG00000269935 ENSG00000263639 ENSG00000259467 | 0.5395164437  0.537198171  0.537198171  0.537198171  0.6529723688  0.5726828973  0.5726828973  0.9366651553  0.6421128696 | 2.086957566  2.082294769  2.082294769  2.082294769  2.209259437  2.145510557  2.145510557  2.805264883  2.196098465 | 2.042355508  2.041040545  2.041040545  2.041040545  2.166236006  2.107422546  2.107422546  2.759636905  2.163086757 | 1.021838538  1.020212349  1.020212349  1.020212349  1.019860915  1.018073267  1.018073267  1.016534051  1.015261389 | 0.3068573261  0.307627762  0.307627762  0.307627762  0.3077944282  0.3086431385  0.3086431385  0.3093751408  0.3099812445 | 0.5592185281  0.5602261883  0.5602261883  0.5602261883  0.5604772754  0.561678175  0.561678175  0.5626657517  0.5633702785 | 0  0  0  0  0  0  0  0  0 | 0  0  0  0  0  0  0  0  0 | 0  0  0  0  0  0  0  0  0 | 0  0  0  0  0  0  0  0  0 | 2.189801957  1.459867971  1.459867971  1.459867971  0  0.7299339855  0.7299339855  0  0 | 1.038333469  1.038333469  1.038333469  1.038333469  0  3.115000407  3.115000407  0  0 | 1.841832474  1.841832474  1.841832474  1.841832474  0  0.9209162369  0.9209162369  0  0 | 1.404229425  2.106344138  2.106344138  2.106344138  0  2.106344138  2.106344138  0  0.7021147127 | 0  0  0  0  1.661120046  0  0  0  4.983360138 | 0  0  0  0  3.509189128  0  0  5.263783691  0 | 0  0  0  0  0.6788559006  0  0  0.6788559006  1.357711801 | 0  0  0  0  1.986503352  0  0  5.297342272  0.6621677839 |
| 21260 ENSG00000250740 0.886621829 2.748803507 2.70771782 1.015173548 0.3100231073 0.5633746474 0 0 0 0 0 0 0 0 3.322240092 0 1.357711801 5.959510055 | | | | | | | | | | | | | | | | | | | |
| 21264  21267  21272  21280  21281  21305  21318  21322  21335 | ENSG00000150594 ENSG00000205444 ENSG00000169174 ENSG00000284407 ENSG00000259422 ENSG00000121335 ENSG00000221857 ENSG00000249231 ENSG00000176204 | 0.6125526848  0.873299381  0.5791387559  0.5268112457  0.5268112457  0.5225547386  0.5577499041  0.882875183  0.8498016195 | 2.227694221  2.742303169  2.156396522  2.060729997  2.060729997  2.052533427  2.12000153  2.713878764  2.67970578 | 2.19508323  2.703218865  2.127600724  2.034321319  2.034321319  2.031763966  2.101116177  2.690460143  2.658204136 | 1.01485638  1.014458431  1.013534399  1.012981567  1.012981567  1.010222379  1.008988248  1.008704318  1.008088786 | 0.3101742936  0.310364055  0.3108049738  0.3110689656  0.3110689656  0.3123887598  0.3129802703  0.3131164603  0.3134118407 | 0.5635620688  0.5638228157  0.5644425197  0.5647361255  0.5647361255  0.566473191  0.5672198491  0.5673351822  0.5675210285 | 0  0  0  0  0  0  0  0  0 | 0  0  0  0  0  0  0  0  0 | 0  0  0  0  0  0  0  0  0 | 0  0  0  0  0  0  0  0  0 | 0  0  0.7299339855  1.459867971  1.459867971  0.7299339855  2.189801957  0  0 | 0  0  4.153333876  2.076666938  2.076666938  2.076666938  1.038333469  0  0 | 0.9209162369  0  0  0  0  0  2.762748711  0  0 | 0.7021147127  0  1.404229425  2.106344138  2.106344138  2.106344138  0.7021147127  0  0 | 0  0  0  0  0  0  0  0  0 | 1.754594564  1.754594564  0  0  0  0  0  5.263783691  3.509189128 | 0  4.751991304  0  0.6788559006  0.6788559006  1.357711801  0  1.357711801  2.715423602 | 3.973006704  3.973006704  0.6621677839  0  0  0  0  3.973006704  3.973006704 |
| 21336 ENSG00000136689 0.8498016195 2.67970578 2.658204136 1.008088786 0.3134118407 0.5675210285 0 0 0 0 0 0 0 0 0 3.509189128 2.715423602 3.973006704 | | | | | | | | | | | | | | | | | | | |
| 21377 | ENSG00000257830 | 1.189397804 | 2.266949448 | 2.256887353 | 1.004458395 | 0.3151577154 | 0.5695896681 | 1.265246467 | 0 | 0 | 0 | 0 | 0 | 0 | 0 | 8.305600229 | 0 | 2.715423602 | 1.986503352 |

| 21383  21384  21391  21421  21429  21430  21446  21449 | ENSG00000169900 ENSG00000284654 ENSG00000188897 ENSG00000237766 ENSG00000275688 ENSG00000275718 ENSG00000254605 ENSG00000258695 | 0.681037742  0.5582151545  0.5915448288  0.6190556868  0.6171174525  0.6171174525  0.8328318568  1.106592361 | 2.226456848  2.084519864  2.190873674  2.193893916  2.189597802  2.189597802  2.656222414  2.210722674 | 2.217954654  2.077705968  2.186072501  2.198867883  2.197602873  2.197602873  2.669276085  2.221728683 | 1.003833349  1.003279528  1.002196255  0.997737942  0.9963573621  0.9963573621  0.9951096585  0.995046196 | 0.3154589471  0.3157260106  0.3162488162  0.3184064497  0.319076542  0.319076542  0.3196829336  0.3197137969 | 0.5699795188  0.5704299672  0.5711875561  0.5742791271  0.5752191778  0.5752191778  0.5758824143  0.5758843091 | 0  0  0  0  0  0  0  1.265246467 | 0  0  0  0  0  0  0  0 | 0  0  0  0  0  0  0  0 | 0  0  0  0  0  0  0  0 | 0  0  0  0  0  0  0  0 | 0  0  0  0  0  0  0  0 | 0  0.9209162369  0  0  0  0  0  0 | 0  0  0.7021147127  0.7021147127  0  0  0  0 | 3.322240092  0  1.661120046  1.661120046  1.661120046  1.661120046  3.322240092  3.322240092 | 3.509189128  1.754594564  0  1.754594564  1.754594564  1.754594564  0  0 | 0.6788559006  2.036567702  4.073135404  0  0.6788559006  0.6788559006  2.036567702  3.394279503 | 0.6621677839  1.986503352  0.6621677839  3.31083892  3.31083892  3.31083892  4.635174488  5.297342272 |
| --- | --- | --- | --- | --- | --- | --- | --- | --- | --- | --- | --- | --- | --- | --- | --- | --- | --- | --- | --- |
| 21451 ENSG00000237622 0.660486001 2.200097204 2.211655375 0.9947739731 0.3198462074 0.5760585627 0 0 0 0 0 2.076666938 0 0 1.661120046 3.509189128 0.6788559006 0 | | | | | | | | | | | | | | | | | | | |
| 21473  21530  21533  21534  21539  21540  21555  21568  21580 | ENSG00000273618 ENSG00000236172 ENSG00000271623 ENSG00000270424 ENSG00000180708 ENSG00000204136 ENSG00000235749 ENSG00000249192 ENSG00000279973 | 0.5568244781  0.9907851738  0.5393975304  0.5393975304  0.5212829834  0.5744459341  0.8304758871  0.5466522079  0.6116647615 | 2.080739045  2.12039858  2.060191226  2.060191226  2.050098518  2.033257983  2.61864227  2.017848608  2.169432033 | 2.095418292  2.153367056  2.092638769  2.092638769  2.083717646  2.067047721  2.666199457  2.058829857  2.217075916 | 0.992994598  0.984689802  0.9844944367  0.9844944367  0.9838657953  0.9836531408  0.9821629301  0.9800948831  0.9785104867 | 0.320712587  0.3247764635  0.3248724655  0.3248724655  0.3251815035  0.3252860871  0.3260195869  0.3270392847  0.3278219073 | 0.577010841  0.5827958356  0.5828787266  0.5828787266  0.5832855467  0.5834460526  0.5843276461  0.5858291342  0.5869045129 | 0  0  0  0  0  0  0  0  0 | 0  0  0  0  0  0  0  0  0 | 0  0.9913102377  0  0  0  0  0  0  0 | 0  0  0  0  0  0  0  0  0 | 0  2.189801957  0  0  2.189801957  0  0  0  0.7299339855 | 0  0  0  0  1.038333469  0  0  2.076666938  4.153333876 | 0.9209162369  0  0  0  0.9209162369  0  0  0  0 | 0  0  2.808458851  2.808458851  2.106344138  0.7021147127  0  1.404229425  0.7021147127 | 0  0  1.661120046  1.661120046  0  0  0  0  0 | 1.754594564  0  0  0  0  3.509189128  5.263783691  1.754594564  1.754594564 | 1.357711801  4.073135404  0.6788559006  0.6788559006  0  1.357711801  2.715423602  0  0 | 2.648671136  4.635174488  1.324335568  1.324335568  0  1.324335568  1.986503352  1.324335568  0 |
| 21592 ENSG00000250640 0.5757096318 2.077024643 2.126067372 0.9769326553 0.3286024938 0.587920596 0 0 0 0 0 0 0.9209162369 0 3.322240092 0 0.6788559006 1.986503352 | | | | | | | | | | | | | | | | | | | |
| 21595  21600  21601  21611  21646  21650  21652  21692  21700 | ENSG00000255807 ENSG00000248449 ENSG00000259692 ENSG00000279536 ENSG00000283982 ENSG00000177354 ENSG00000262503 ENSG00000146453 ENSG00000222630 | 0.6331269192  0.5236012561  0.5236012561  0.786520323  0.8057165813  0.8057165813  0.6292577818  0.7932302944  1.029903084 | 2.144924656  2.053722238  2.053722238  2.616885447  2.582580848  2.582580848  2.127361043  2.576619411  2.183412007 | 2.196111685  2.103793582  2.103793582  2.684180755  2.660055057  2.660055057  2.19166731  2.665571068  2.259896134 | 0.9766919736  0.976199498  0.976199498  0.9749289207  0.9708749602  0.9708749602  0.9706587459  0.9666294184  0.9661559105 | 0.32872167  0.328965612  0.328965612  0.3295955192  0.3316105486  0.3316105486  0.3317182416  0.3337293227  0.333966171 | 0.5881065858  0.5883795388  0.5883795388  0.5892333942  0.5917811588  0.5917811588  0.5919053328  0.5943957396  0.5945982957 | 0  0  0  0  0  0  0  0  0 | 0  0  0  0  0  0  0  0  0.917401664 | 0  0  0  0  0  0  0  0  0 | 0  0  0  0  0  0  0  0  0 | 0  2.919735942  2.919735942  0.7299339855  0  0  1.459867971  0  0.7299339855 | 0  1.038333469  1.038333469  0  0  0  0  0  0 | 1.841832474  0.9209162369  0.9209162369  0  0  0  0.9209162369  0  0 | 0  1.404229425  1.404229425  0  0  0  0  0  0 | 3.322240092  0  0  0  4.983360138  4.983360138  1.661120046  0  0 | 1.754594564  0  0  0  0  0  3.509189128  3.509189128  0 | 0.6788559006  0  0  4.073135404  2.036567702  2.036567702  0  2.036567702  4.751991304 | 0  0  0  4.635174488  2.648671136  2.648671136  0  3.973006704  5.959510055 |
| 21707 ENSG00000214919 0.5088590573 2.022108312 2.094432339 0.9654684348 0.3343102387 0.5950189373 0 0 0 0 0 2.076666938 0 0.7021147127 0 0 0.6788559006 2.648671136 | | | | | | | | | | | | | | | | | | | |
| 21716  21733  21739  21740  21748  21762  21796  21815  21859 | ENSG00000262358 ENSG00000137473 ENSG00000236975 ENSG00000227757 ENSG00000243141 ENSG00000172572 ENSG00000105143 ENSG00000187080 ENSG00000145920 | 0.5488209534  0.5647181566  0.6207447676  0.5730552577  0.5633274802  0.830050504  0.7599607336  0.7832139136  0.7394090005 | 2.04627023  2.029929542  2.099359006  2.03157895  2.025872106  2.651272773  2.544822779  2.564060428  2.50949139 | 2.122122426  2.11177333  2.185300111  2.114691469  2.110392616  2.766540926  2.666087444  2.693426729  2.646822099 | 0.9642564466  0.9612440467  0.9606730882  0.9606975671  0.9599503384  0.9583349188  0.9545158711  0.9519696233  0.9481148696 | 0.3349173708  0.3364294742  0.3367165667  0.3367042549  0.3370802096  0.3378939012  0.3398225795  0.3411123877  0.3430709792 | 0.5958524875  0.5980744829  0.5983921139  0.5983921139  0.5988180016  0.5998773492  0.6023603119  0.6041143044  0.6063379359 | 0  0  0  0  0  0  0  0  0 | 0  0  0  0  0  0  0  0  0 | 0  0  0  0  0  0  0  0  0 | 0  0  0  0  0  0  0  0  0 | 1.459867971  0  0  0  0  0  2.189801957  0  1.459867971 | 0  0  0  0  0  0  0  0  0 | 2.762748711  0  0.9209162369  0  0  0  5.525497422  0  4.604581185 | 0.7021147127  0  0  0.7021147127  0  0  1.404229425  0  2.808458851 | 1.661120046  1.661120046  1.661120046  0  1.661120046  3.322240092  0  3.322240092  0 | 0  1.754594564  3.509189128  3.509189128  1.754594564  0  0  0  0 | 0  2.036567702  1.357711801  0.6788559006  1.357711801  0.6788559006  0  4.751991304  0 | 0  1.324335568  0  1.986503352  1.986503352  5.959510055  0  1.324335568  0 |
| 21919 ENSG00000141431 0.7615474072 2.544121834 2.696032651 0.9436539402 0.3453465184 0.608716965 0 0 0 0 0 0 0 0 0 1.754594564 4.073135404 3.31083892 | | | | | | | | | | | | | | | | | | | |
| 21935  21942  21946  21955  21957  21958  22001  22004  22013 | ENSG00000278022 ENSG00000271560 ENSG00000260989 ENSG00000164893 ENSG00000279249 ENSG00000182376 ENSG00000234521 ENSG00000230872 ENSG00000105610 | 0.7739115325  0.6044495416  0.5717560165  0.7537578641  0.7537578641  0.5947217641  0.7509765113  0.566108833  0.5997299545 | 2.565344542  2.045733188  2.053225769  2.532878744  2.532878744  2.037650336  2.527786644  2.036873006  2.027527408 | 2.722293468  2.173450951  2.181853491  2.694656517  2.694656517  2.167648088  2.70118755  2.176996473  2.169581798 | 0.9423468016  0.9412373385  0.9410465815  0.9399634899  0.9399634899  0.9400282027  0.9358056771  0.9356344995  0.9345245292 | 0.3460151141  0.3465832462  0.3466809885  0.3472362886  0.3472362886  0.3472030945  0.349373248  0.3494614054  0.350033389 | 0.609450373  0.6102562991  0.6103718925  0.6109328298  0.6109328298  0.6109328298  0.6134912933  0.6135903199  0.6143433419 | 0  0  0  0  0  0  0  0  0 | 0  0  0  0  0  0  0  0  0 | 0  0  0  0  0  0  0  0  0 | 0  0  0  0  0  0  0  0  0 | 0  0  0.7299339855  0  0  0  0  0  0 | 4.153333876  0  0  0  0  0  0  0  0 | 0.9209162369  0  0  0  0  0  0  0  0 | 4.212688276  1.404229425  0  0  0  0.7021147127  0  0  0.7021147127 | 0  1.661120046  1.661120046  1.661120046  1.661120046  3.322240092  1.661120046  1.661120046  1.661120046 | 0  3.509189128  1.754594564  0  0  1.754594564  0  1.754594564  3.509189128 | 0  0.6788559006  2.715423602  4.073135404  4.073135404  1.357711801  2.715423602  2.715423602  0 | 0  0  0  3.31083892  3.31083892  0  4.635174488  0.6621677839  1.324335568 |
| 22025 ENSG00000230314 0.590002177 2.019384832 2.163714676 0.9332953436 0.3506674987 0.6150650933 0 0 0 0 0 0 0 0 3.322240092 1.754594564 0.6788559006 1.324335568 | | | | | | | | | | | | | | | | | | | |
| 22026  22027  22037  22047  22050  22057  22103  22105  22108 | ENSG00000260784 ENSG00000261375 ENSG00000200057 ENSG00000221937 ENSG00000285177 ENSG00000272942 ENSG00000145808 ENSG00000235713 ENSG00000205076 | 0.590002177  0.590002177  0.973331759  0.5977917202  0.7904489416  0.5619368038  0.7380496458  0.7380496458  0.7380496458 | 2.019384832  2.019384832  2.098803197  2.020044546  2.572029427  2.024560437  2.467639797  2.467639797  2.467639797 | 2.163714676  2.163714676  2.250788742  2.167988029  2.761260121  2.173223242  2.659641863  2.659641863  2.659641863 | 0.9332953436  0.9332953436  0.9324745403  0.9317600092  0.9314694429  0.9315934035  0.9278090527  0.9278090527  0.9278090527 | 0.3506674987  0.3506674987  0.3510913382  0.3514605661  0.351610784  0.3515466935  0.3535066273  0.3535066273  0.3535066273 | 0.6150650933  0.6150650933  0.6155305848  0.6158410345  0.6159294587  0.6159294587  0.6177803019  0.6177803019  0.6177803019 | 0  0  0  0  0  0  0  0  0 | 0  0  0.917401664  0  0  0  0  0  0 | 0  0  0  0  0  0  0  0  0 | 0  0  0  0  0  0  0  0  0 | 0  0  0.7299339855  0  0  0  0  0  0 | 0  0  0  0  0  0  0  0  0 | 0  0  0  0  0  0  0  0  0 | 0  0  0  0  0  0  0  0  0 | 3.322240092  3.322240092  0  1.661120046  0  1.661120046  0  0  0 | 1.754594564  1.754594564  0  3.509189128  3.509189128  1.754594564  3.509189128  3.509189128  3.509189128 | 0.6788559006  0.6788559006  4.073135404  0.6788559006  0.6788559006  0.6788559006  2.036567702  2.036567702  2.036567702 | 1.324335568  1.324335568  5.959510055  1.324335568  5.297342272  2.648671136  3.31083892  3.31083892  3.31083892 |
| 22152 ENSG00000201136 0.7238612358 2.446494116 2.648858851 0.9236030506 0.355693025 0.6203593364 0 0 0 0 0 0 0 0 3.322240092 0 2.715423602 2.648671136 | | | | | | | | | | | | | | | | | | | |
| 22177  22357  22381  22421  22436  22458  22465  22490  22518 | ENSG00000279587 ENSG00000110848 ENSG00000205457 ENSG00000280057 ENSG00000253944 ENSG00000233538 ENSG00000137392 ENSG00000205596 ENSG00000284078 | 0.7422216749  1.026310044  0.697186539  0.7541534466  0.7021947294  0.6994867855  0.6944051863  0.7187239134  0.6422280001 | 2.475758396  2.934725011  2.420097077  2.47932008  2.426393026  2.421767695  2.414389305  2.392253572  2.316093435 | 2.688317813  3.252607108  2.689933966  2.766136858  2.710072932  2.713069671  2.70855301  2.690695161  2.613249219 | 0.9209321843  0.9022685229  0.899686426  0.896311429  0.8953238851  0.8926301157  0.8913945183  0.8890838347  0.8862887697 | 0.3570858341  0.3669142423  0.368287149  0.3700864567  0.3706139757  0.372055286  0.3727175575  0.373958026  0.3754619391 | 0.6220851539  0.6340476457  0.6357601274  0.637718668  0.6382007021  0.640055035  0.6409945619  0.6424659339  0.6441945118 | 0  0  0  0  0  0  0  0  0 | 0  0  0  0  0  0  0  0  0 | 0  0  0  0  0  0  0  0  0 | 0  0  0  0  0  0  0  0  0 | 0  0  0  0  0  0.7299339855  0  0  0 | 0  0  0  0  0  4.153333876  0  0  0 | 0  0  0  0  0  0  0  0  3.683664948 | 0  0  0  0  0  3.510573564  0  0  0 | 0  0  1.661120046  3.322240092  0  0  1.661120046  0  0 | 3.509189128  7.018378255  0  1.754594564  1.754594564  0  0  5.263783691  0 | 4.073135404  0  3.394279503  0  2.036567702  0  2.036567702  2.036567702  2.036567702 | 1.324335568  5.297342272  3.31083892  3.973006704  4.635174488  0  4.635174488  1.324335568  1.986503352 |
| 22538 ENSG00000156486 0.6513873386 2.334746994 2.641791149 0.8837742508 0.376818089 0.6459475937 0 0 0 0 3.649669928 0 2.762748711 1.404229425 0 0 0 0 | | | | | | | | | | | | | | | | | | | |
| 22554 | ENSG00000202512 | 0.6252348841 | 2.298064667 | 2.605521729 | 0.8819978898 | 0.3777779484 | 0.6471049008 | 0 | 0 | 0 | 0 | 1.459867971 | 0 | 0 | 0 | 0 | 0 | 3.394279503 | 2.648671136 |

| 22587  22590  22592  22606  22613  22625  22753  22772 | ENSG00000233041 ENSG00000196166 ENSG00000129988 ENSG00000270973 ENSG00000255425 ENSG00000274019 ENSG00000177504 ENSG00000276662 | 0.6625111535  0.6856503499  0.6856503499  0.6953552839  0.6713137285  0.7467892866  0.693024311  0.6689954558 | 2.340060082  2.355015601  2.355015601  2.353705184  2.354729902  2.427572706  2.37605056  2.349748236 | 2.663157778  2.68068251  2.68068251  2.684752894  2.688003271  2.774946658  2.752111433  2.72736747 | 0.8786787253  0.8785134355  0.8785134355  0.8766934154  0.8760145227  0.8748177912  0.8633555063  0.8615444242 | 0.379575497  0.3796651498  0.3796651498  0.3806531842  0.3810221388  0.3816730553  0.3879420347  0.388938265 | 0.6492628206  0.6493089166  0.6493089166  0.6505337686  0.650987942  0.6517541874  0.6587324973  0.659873084 | 0  0  0  0  0  0  0  0 | 0  0  0  0  0  0  0  0 | 0  0  0  0  0  0  0  0 | 0  0  0  0  0  0  0  0 | 2.189801957  0  0  0  1.459867971  0  0  0.7299339855 | 2.076666938  0  0  0  5.191667345  0  0  5.191667345 | 3.683664948  0  0  0  0  0  0.9209162369  0 | 0  0  0  0  1.404229425  0  0  2.106344138 | 0  0  0  4.983360138  0  1.661120046  3.322240092  0 | 0  3.509189128  3.509189128  0  0  5.263783691  0  0 | 0  3.394279503  3.394279503  2.036567702  0  2.036567702  4.073135404  0 | 0  1.324335568  1.324335568  1.324335568  0  0  0  0 |
| --- | --- | --- | --- | --- | --- | --- | --- | --- | --- | --- | --- | --- | --- | --- | --- | --- | --- | --- | --- |
| 22793 ENSG00000231533 0.6497954335 2.312870765 2.690194426 0.8597411188 0.3899317632 0.66092014 0 0 0 0 0 0 0 0 0 1.754594564 3.394279503 2.648671136 | | | | | | | | | | | | | | | | | | | |
| 22794  22798  22822  22824  22856  22876  22882  22991  23058 | ENSG00000146809 ENSG00000279302 ENSG00000249816 ENSG00000242048 ENSG00000074706 ENSG00000229399 ENSG00000266311 ENSG00000273588 ENSG00000280068 | 0.6497954335  0.7059261799  0.6511861099  0.6568924137  0.6470140808  0.758432455  0.6306807783  0.5853925907  0.6276883484 | 2.312870765  2.374767559  2.315557635  2.326520429  2.306936459  2.438316747  2.276366438  2.201950565  2.218753485 | 2.690194426  2.76341836  2.70303795  2.71626977  2.701470277  2.861641864  2.674340268  2.616948079  2.658786488 | 0.8597411188  0.859358682  0.8566500645  0.8565130219  0.8539558916  0.8520691486  0.8511880351  0.8414192789  0.8344985562 | 0.3899317632  0.3901426579  0.3916383083  0.3917140731  0.3931294293  0.394175714  0.3946649082  0.4001130889  0.4040001006 | 0.66092014  0.661161575  0.6629982491  0.6630684024  0.6645325299  0.6657186008  0.666370017  0.6723661079  0.6768418478 | 0  0  0  0  0  0  0  0  0 | 0  0  0  0  0  0  0  0  0 | 0  0  0  0  0  0  0  0  0 | 0  0  0  0  0  0  0  0  0 | 0  0  0  0  0  0  3.649669928  2.189801957  0 | 0  0  0  4.153333876  0  0  2.076666938  0  0 | 0  0  0  0.9209162369  0  0  1.841832474  0  0 | 0  0  0  2.808458851  0  0.7021147127  0  3.510573564  0 | 0  3.322240092  0  0  0  6.644480183  0  0  0 | 1.754594564  1.754594564  1.754594564  0  1.754594564  1.754594564  0  0  3.509189128 | 3.394279503  3.394279503  4.073135404  0  2.036567702  0  0  0  2.036567702 | 2.648671136  0  1.986503352  0  3.973006704  0  0  1.324335568  1.986503352 |
| 23072 ENSG00000123454 0.6212528601 2.238992631 2.687047222 0.8332539199 0.4047015386 0.6776891445 0 0 0 0 1.459867971 4.153333876 1.841832474 0 0 0 0 0 | | | | | | | | | | | | | | | | | | | |
| 23100  23107  23143  23165  23172  23183  23220  23252  23255 | ENSG00000107831 ENSG00000182632 ENSG00000167083 ENSG00000249444 ENSG00000277655 ENSG00000254971 ENSG00000146678 ENSG00000278872 ENSG00000225285 | 0.6166163146  0.6446345443  0.6087929669  0.6001737238  0.6902179615  0.6134999385  0.626401084  0.5928777793  0.563492148 | 2.228941091  2.286625748  2.232141315  2.194262529  2.298489816  2.194851296  2.24854975  2.19857688  2.13402774 | 2.685182888  2.75758956  2.701350813  2.663220585  2.79217017  2.671011065  2.751645702  2.703780439  2.625200389 | 0.8300891166  0.8292117801  0.8263056039  0.8239131755  0.8231911652  0.821730514  0.8171654326  0.8131491923  0.8129008928 | 0.4064884  0.4069845802  0.4086307554  0.4099888935  0.4103992922  0.4112302885  0.413833894  0.4161325254  0.4162748826 | 0.6798562482  0.680479909  0.6821695215  0.6837867861  0.6842644853  0.6852951257  0.6885647069  0.6914364407  0.6915837493 | 0  0  0  0  0  0  0  0  0 | 0  0  0  0  0  0  0  0  0 | 0  0  0  0  0  0  0  0  0 | 0  0  0  0  0  0  0  0  0 | 0  0  2.919735942  0  0  0  0  3.649669928  0 | 4.153333876  5.191667345  0  3.115000407  0  0  5.191667345  0  2.076666938 | 1.841832474  1.841832474  3.683664948  2.762748711  0  0  0.9209162369  2.762748711  0 | 1.404229425  0.7021147127  0.7021147127  0  0  0  1.404229425  0.7021147127  0 | 0  0  0  0  1.661120046  3.322240092  0  0  0 | 0  0  0  0  5.263783691  0  0  0  0 | 0  0  0  0  1.357711801  2.715423602  0  0  2.036567702 | 0  0  0  1.324335568  0  1.324335568  0  0  2.648671136 |
| 23280 ENSG00000204952 0.6401746353 2.224161091 2.743059959 0.8108321087 0.4174620926 0.692811338 0 0 0 0 0 0 0 0 4.983360138 0 2.036567702 0.6621677839 | | | | | | | | | | | | | | | | | | | |
| 23304  23305  23355  23415  23420  23431  23467  23468  23531 | ENSG00000270540 ENSG00000271306 ENSG00000165164 ENSG00000178934 ENSG00000255491 ENSG00000230126 ENSG00000164485 ENSG00000274175 ENSG00000205106 | 0.5676895007  0.5676895007  0.5840438889  0.5904427557  0.7864358451  0.7836544924  0.5826532126  0.5826532126  0.8055874492 | 2.14527984  2.14527984  2.162429356  2.17252743  2.601452727  2.596663521  2.158784748  2.158784748  2.564041437 | 2.654076714  2.654076714  2.692191306  2.720717628  3.259438483  3.259548278  2.719825923  2.719825923  3.260305647 | 0.8082960937  0.8082960937  0.8032227691  0.798512645  0.7981291072  0.7966329379  0.7937216605  0.7937216605  0.7864420438 | 0.4189201507  0.4189201507  0.4218459874  0.4245730581  0.4247955718  0.4256642421  0.4273574875  0.4273574875  0.4316085602 | 0.6944853046  0.6944853046  0.6978385666  0.7005522323  0.7007675883  0.70187094  0.7035649118  0.7035649118  0.708648027 | 0  0  0  0  0  0  0  0  0 | 0  0  0  0  0  0  0  0  0 | 0  0  0  0  0  0  0  0  0 | 0  0  0  0  0  0  0  0  0 | 1.459867971  1.459867971  0  0  0  0  0  0  0 | 0  0  0  0  0  0  0  0  0 | 1.841832474  1.841832474  0  0  0  0  0  0  0 | 3.510573564  3.510573564  0  0  0  0  0  0  0 | 0  0  1.661120046  0  0  0  1.661120046  1.661120046  0 | 0  0  0  1.754594564  0  0  0  0  7.018378255 | 0  0  2.036567702  1.357711801  6.788559006  5.430847205  1.357711801  1.357711801  0 | 0  0  3.31083892  3.973006704  2.648671136  3.973006704  3.973006704  3.973006704  2.648671136 |
| 23658 ENSG00000253774 0.5274134017 2.044773924 2.645069677 0.7730510622 0.4394921617 0.7177183053 0 0 0 0 1.459867971 0 2.762748711 2.106344138 0 0 0 0 | | | | | | | | | | | | | | | | | | | |
| 23683  23707  23708  23710  23714  23718  23734  23750  23771 | ENSG00000228235 ENSG00000179799 ENSG00000280089 ENSG00000259237 ENSG00000204661 ENSG00000254670 ENSG00000185940 ENSG00000234718 ENSG00000227688 | 0.5479651347  0.5725076998  0.5725076998  0.5725076998  0.5107015577  0.57979551  0.5711170234  0.5743189554  0.6258570933 | 2.085931309  2.049476237  2.049476237  2.049476237  2.028146846  2.151195311  2.045113517  2.092694948  2.137498754 | 2.709094292  2.670396993  2.670396993  2.670396993  2.643145539  2.804222691  2.669250619  2.737910884  2.805455417 | 0.7699736829  0.7674799821  0.7674799821  0.7674799821  0.7673231821  0.7671271322  0.7661751589  0.7643400522  0.7619079386 | 0.4413155039  0.4427961905  0.4427961905  0.4427961905  0.4428893886  0.4430059316  0.4435720871  0.4446646231  0.4461149516 | 0.7199351642  0.7214672242  0.7214672242  0.7214672242  0.7215141572  0.7216112522  0.7220614976  0.7233523247  0.7250945012 | 0  0  0  0  0  0  0  0  0 | 0  0  0  0  0  0  0  0  0 | 0  0  0  0  0  0  0  0  0 | 0  0  0  0  0  0  0  0  0 | 2.189801957  0  0  0  1.459867971  0.7299339855  0  0  0 | 0  0  0  0  0  0  0  0  0 | 3.683664948  0  0  0  0  5.525497422  0  0.9209162369  0 | 0.7021147127  0  0  0  0  0.7021147127  0  0  0 | 0  0  0  0  0  0  0  3.322240092  3.322240092 | 0  3.509189128  3.509189128  3.509189128  0  0  3.509189128  0  3.509189128 | 0  2.036567702  2.036567702  2.036567702  1.357711801  0  1.357711801  0  0.6788559006 | 0  1.324335568  1.324335568  1.324335568  3.31083892  0  1.986503352  2.648671136  0 |
| 23854 ENSG00000243955 0.5836033102 2.080984614 2.757598997 0.7546364125 0.4504671694 0.7295603867 0 0 0 0 0 0 0 0 4.983360138 0 1.357711801 0.6621677839 | | | | | | | | | | | | | | | | | | | |
| 23860  23870  23872  23883  23884  23911  23912  23954  23965 | ENSG00000260650 ENSG00000237372 ENSG00000229688 ENSG00000231655 ENSG00000224557 ENSG00000226969 ENSG00000222750 ENSG00000277435 ENSG00000002933 | 0.5366527834  0.5822126338  0.5302539167  0.5738983762  0.5738983762  0.5069081306  0.5234307041  0.5316445931  0.6166768738 | 2.023546769  2.077715838  2.026953923  2.055336947  2.055336947  2.018648176  2.014130338  2.030145766  2.120642191 | 2.683545557  2.75708871  2.690702049  2.730243012  2.730243012  2.688106671  2.682182744  2.714760299  2.84116213 | 0.75405717  0.7535904921  0.753317865  0.7528036654  0.7528036654  0.7509553833  0.7509295712  0.7478176862  0.7463995697 | 0.4508148938  0.4510951553  0.4512589259  0.4515679038  0.4515679038  0.4526795076  0.4526950426  0.4545701327  0.4554260791 | 0.7299762541  0.7301240605  0.7302827599  0.7304295343  0.7304295343  0.7314266047  0.7314266047  0.733168451  0.7342118325 | 0  0  0  0  0  0  0  0  0 | 0  0  0  0  0  0  0  0  0 | 0  0  0  0  0  0  0  0  0 | 0  0  0  0  0  0  0  0  0 | 0  0  0  0  0  0  0  0  0 | 0  0  0  0  0  0  3.115000407  0  0 | 0  0  0  0  0  0  1.841832474  0  0 | 0  0  0  0  0  0.7021147127  0  0  0 | 0  4.983360138  1.661120046  0  0  0  0  1.661120046  4.983360138 | 1.754594564  0  0  3.509189128  3.509189128  0  0  0  1.754594564 | 2.036567702  0.6788559006  2.715423602  2.715423602  2.715423602  3.394279503  0  3.394279503  0 | 2.648671136  1.324335568  1.986503352  0.6621677839  0.6621677839  1.986503352  1.324335568  1.324335568  0.6621677839 |
| 23967 ENSG00000248517 0.5352621071 2.01972003 2.707059908 0.7460935844 0.4556108846 0.7344484719 0 0 0 0 0 0 0 0 0 1.754594564 1.357711801 3.31083892 | | | | | | | | | | | | | | | | | | | |
| 23985  24092  24216  24239  24268  24325  24352  24598  24620 | ENSG00000261509 ENSG00000214992 ENSG00000285165 ENSG00000251416 ENSG00000228615 ENSG00000278607 ENSG00000257654 ENSG00000260963 ENSG00000254864 | 0.5274725639  0.5239581073  0.5260818875  0.5287524472  0.5143281652  1.021385952  0.6952261519  0.6153311936  0.6125498408 | 2.019645113  2.014708708  2.0152278  2.026945227  2.013899741  2.031282121  2.330991561  2.255423083  2.249399363 | 2.712710793  2.741759496  2.781723796  2.806277407  2.796413477  2.836790741  3.266227535  3.268355243  3.268529525 | 0.7445117697  0.7348232811  0.7244528744  0.722289686  0.7201723771  0.7160493343  0.7136647818  0.6900789281  0.6881991873 | 0.4565669235  0.4624471265  0.4687877489  0.4701163928  0.4714188691  0.4739608869  0.4754344888  0.4901445537  0.4913273557 | 0.735437277  0.7416007277  0.7478961525  0.7492367583  0.750484093  0.7527843315  0.7542875934  0.7698650075  0.77101675 | 0  0  0  0  0  1.265246467  0  0  0 | 0  0  0  0  0  0  0  0  0 | 0  0  0  0  0  0  0  0  0 | 0  0  0  0  0  0  0  0  0 | 0  0.7299339855  0  0  0  0  0  0  0 | 0  4.153333876  0  1.038333469  1.038333469  0  0  0  0 | 0  0  0  4.604581185  0.9209162369  0  0  0  0 | 0  1.404229425  0  0.7021147127  4.212688276  0  0  0  0 | 1.661120046  0  1.661120046  0  0  0  0  0  0 | 0  0  0  0  0  7.018378255  7.018378255  0  0 | 1.357711801  0  0.6788559006  0  0  0  0  4.073135404  2.715423602 | 3.31083892  0  3.973006704  0  0  3.973006704  1.324335568  3.31083892  4.635174488 |
| 25042 ENSG00000149972 0.610278007 2.136259428 3.271896914 0.6529115937 0.5138132743 0.7927152724 0 0 0 0 0 0 0 0 6.644480183 0 0.6788559006 0 | | | | | | | | | | | | | | | | | | | |
| 25112 | ENSG00000124785 | 0.5573691922 | 2.118905809 | 3.272336174 | 0.6475208221 | 0.5172949228 | 0.7958621115 | 0 | 0 | 0 | 0 | 0 | 0 | 0 | 0 | 0 | 0 | 2.715423602 | 3.973006704 |

| 25419 | ENSG00000254758 | 0.542215489 | 2.032223341 | 3.274411944 | 0.6206376521 | 0.5348380602 | 0.8129142947 | 0 | 0 | 0 | 0 | 0 | 0 | 0 | 0 | 0 | 1.754594564 | 4.751991304 | 0 |
| --- | --- | --- | --- | --- | --- | --- | --- | --- | --- | --- | --- | --- | --- | --- | --- | --- | --- | --- | --- |
|  |  |  |  |  |  |  |  |  |  |  |  |  |  |  |  |  |  |  |  |
