# Supplementary material for: Analysis of H3K4me3-ChIP-Seq and RNA-Seq data to understand the putative role of miRNAs and their target genes in breast cancer cell lines
Source: Genomics Inform. 2021 Jun 30;19(2):e17. doi: 10.5808/gi.21020 (PMC8261273; doi:10.5808/gi.21020)
Supplement: Supplementary Table 11. — List of genes downregulated in normal-like vs. luminal-A cell lines [file gi-21020suppl11.pdf]

List of genes downregulated in Normal-like vs Luminal-A cell-lines

Gene	baseMean	log2FoldChange	lfcSE	stat	pvalue	padj	MCF10A.RNA.Seq.Rep1_sorted	MCF10A.RNA.Seq.Rep2_sorted	MCF10A.RNA.Seq.Rep3_sorted	MCF10A.RNA.Seq.Rep4_sorted	MCF7.RNA.Seq.Rep1_sorted	MCF7.RNA.Seq.Rep2_sorted	MCF7.RNA.Seq.Rep3_sorted	MCF7.RNA.Seq.Rep4_sorted	ZR751.RNA.Seq.Rep1_sorted	ZR751.RNA.Seq.Rep2_sorted	ZR751.RNA.Seq.Rep3_sorted	ZR751.RNA.Seq.Rep4_sorted
ENSG00000167601	4792.003	-2.385	1.597	-1.493	0.135	0.302	11158.209	9939.130	10935.143	9549.932	4284.712	3839.757	4224.243	3568.147	1.661	1.755	0.679	0.662
ENSG00000177469	6530.355	-2.172	1.432	-1.517	0.129	0.291	9571.590	18098.500	9318.316	17286.347	5997.138	6196.774	5849.660	6014.315	11.628	5.264	6.789	7.946
ENSG00000173706	2385.381	-2.063	1.468	-1.405	0.160	0.344	6003.594	3754.008	5930.018	3670.777	2956.233	1691.445	2950.616	1648.565	9.967	0.000	3.394	5.960
ENSG00000229807	1473.107	-2.759	1.660	-1.662	0.096	0.231	3859.002	2934.768	3862.145	2988.639	0.730	0.000	0.000	0.702	1217.601	893.089	1117.397	803.210
ENSG00000117519	1648.463	-2.111	1.225	-1.724	0.085	0.209	2245.812	4431.050	2276.048	4569.261	8.029	22.843	4.605	16.149	1559.792	1549.307	1550.507	1548.148
ENSG00000104081	703.342	-2.274	1.282	-1.774	0.076	0.191	2221.773	770.617	2142.221	835.826	7.299	1.038	3.684	2.808	546.508	668.501	602.824	637.005
ENSG00000089327	1267.229	-2.167	1.276	-1.698	0.090	0.218	2220.508	3141.183	2098.604	3060.755	1213.150	1211.735	1130.885	1107.235	8.306	7.018	3.394	3.973
ENSG00000270641	757.787	-2.781	1.442	-1.929	0.054	0.144	2004.150	1471.512	2021.282	1546.337	1.460	2.077	0.000	0.702	614.614	470.231	522.719	438.355
ENSG00000134533	507.492	-3.199	1.748	-1.831	0.067	0.173	1842.199	563.285	1982.620	612.387	0.000	0.000	0.000	0.000	272.424	285.999	266.790	264.205
ENSG00000115935	670.305	-2.439	1.264	-1.929	0.054	0.144	1724.531	1195.374	1781.384	1175.122	627.013	484.902	541.499	502.714	3.322	1.755	3.394	2.649
ENSG00000128739	742.325	-2.096	1.702	-1.232	0.218	0.435	1566.375	1480.686	1592.044	1429.298	0.000	0.000	1.842	0.000	561.459	822.905	639.482	813.804
ENSG00000197343	560.041	-2.407	1.249	-1.927	0.054	0.145	1353.814	1064.186	1346.199	1116.011	1.460	3.115	1.842	4.915	451.825	410.575	503.032	463.517
ENSG00000122861	729.443	-2.573	1.668	-1.542	0.123	0.280	1181.740	2108.189	1132.076	2129.169	469.348	623.000	522.160	586.968	0.000	0.000	0.000	0.662
ENSG00000273173	488.670	-2.152	1.647	-1.307	0.191	0.394	1056.481	974.281	1066.650	946.954	0.000	0.000	1.842	0.000	337.207	543.924	420.212	516.491
ENSG00000124813	439.752	-2.071	1.526	-1.357	0.175	0.367	1043.828	726.582	1075.572	729.427	476.647	393.528	416.254	412.843	1.661	0.000	0.679	0.000
ENSG00000243943	315.720	-2.768	1.661	-1.667	0.096	0.230	905.916	566.954	867.396	588.743	240.878	175.478	240.359	202.911	0.000	0.000	0.000	0.000
ENSG00000214265	380.675	-2.030	1.772	-1.146	0.252	0.484	833.797	764.196	797.013	671.498	0.000	0.000	0.000	0.000	282.390	450.931	329.924	438.355
ENSG00000135457	298.237	-2.313	1.490	-1.553	0.120	0.276	575.687	647.686	622.543	705.782	0.000	1.038	0.000	0.702	247.507	243.889	254.571	279.435
ENSG00000165983	244.149	-2.607	1.380	-1.890	0.059	0.155	560.504	532.093	601.725	511.899	199.272	155.750	211.811	151.657	3.322	1.755	0.000	0.000
ENSG00000167779	306.228	-2.391	1.433	-1.669	0.095	0.229	544.056	871.532	545.221	699.871	317.521	207.667	283.642	202.209	1.661	0.000	1.358	0.000
ENSG00000188015	275.947	-2.278	1.337	-1.705	0.088	0.215	432.714	703.647	445.098	763.711	251.827	223.242	272.591	214.847	1.661	0.000	1.358	0.662
ENSG00000163071	179.313	-2.404	1.603	-1.500	0.134	0.299	389.696	384.391	346.959	440.966	156.206	116.293	176.816	140.423	0.000	0.000	0.000	0.000
ENSG00000164188	107.605	-2.587	1.578	-1.640	0.101	0.240	369.452	124.767	373.724	100.488	0.000	0.000	0.000	0.000	66.445	107.030	68.564	80.784
ENSG00000237651	132.196	-2.358	1.305	-1.807	0.071	0.180	326.434	219.259	331.098	264.816	121.169	118.370	97.617	102.509	3.322	1.755	0.000	0.000
ENSG00000128274	118.335	-2.047	1.075	-1.904	0.057	0.151	320.107	188.067	306.315	143.048	145.257	96.565	111.431	96.892	3.322	7.018	0.679	1.324
ENSG00000269652	129.570	-2.235	1.566	-1.427	0.154	0.333	304.924	271.551	284.506	230.532	136.498	107.987	126.166	92.679	0.000	0.000	0.000	0.000
ENSG00000243649	112.123	-2.244	1.176	-1.909	0.056	0.150	265.702	183.480	302.350	195.065	125.549	80.990	113.273	73.722	3.322	0.000	1.358	0.662
ENSG00000196196	116.182	-2.449	1.530	-1.601	0.109	0.256	242.927	297.238	235.932	244.718	103.651	76.837	111.431	81.445	0.000	0.000	0.000	0.000
ENSG00000212978	97.102	-2.049	1.312	-1.561	0.118	0.272	237.866	151.371	247.828	148.959	82.483	93.450	87.487	114.445	0.000	0.000	0.679	0.662
ENSG00000126970	89.787	-2.453	1.306	-1.878	0.060	0.159	235.336	173.389	224.036	156.052	0.000	1.038	0.000	0.702	66.445	66.675	86.894	66.879
ENSG00000197951	89.131	-2.312	1.542	-1.500	0.134	0.299	187.256	190.820	165.549	218.710	0.000	0.000	0.000	0.000	78.073	59.656	80.784	88.730
ENSG00000222047	123.875	-2.548	1.540	-1.655	0.098	0.234	177.135	371.548	202.227	357.029	80.293	99.680	91.171	107.424	0.000	0.000	0.000	0.000
ENSG00000174607	79.229	-2.391	1.226	-1.950	0.051	0.139	163.217	175.224	156.627	192.701	0.000	2.077	0.000	1.404	69.767	59.656	65.849	64.230
ENSG00000270069	52.041	-2.289	1.288	-1.777	0.076	0.190	103.750	99.997	111.027	128.861	31.387	43.610	39.599	64.595	1.661	0.000	0.000	0.000
ENSG00000175294	45.581	-2.145	1.277	-1.680	0.093	0.225	103.750	83.484	124.905	65.022	49.636	34.265	39.599	45.637	0.000	0.000	0.679	0.000
ENSG00000104324	61.635	-2.007	1.341	-1.496	0.135	0.301	99.954	127.519	130.853	135.955	78.103	48.802	57.097	59.680	1.661	0.000	0.000	0.000
ENSG00000108342	29.103	-2.282	1.316	-1.734	0.083	0.205	86.037	35.779	88.227	37.831	21.168	31.150	20.260	28.787	0.000	0.000	0.000	0.000
ENSG00000171889	33.147	-2.500	1.293	-1.934	0.053	0.143	75.915	71.557	72.366	74.480	26.278	31.150	19.339	26.680	0.000	0.000	0.000	0.000
ENSG00000274565	35.302	-2.068	1.097	-1.885	0.059	0.156	50.610	95.410	51.548	89.848	37.957	25.958	38.678	31.595	0.000	0.000	1.358	0.662
ENSG00000277577	16.460	-2.142	1.281	-1.672	0.094	0.227	50.610	20.183	38.661	26.009	0.000	0.000	0.000	0.000	16.611	15.791	21.045	8.608
ENSG00000171044	20.305	-2.360	1.212	-1.948	0.051	0.139	49.345	40.366	43.618	42.560	17.518	10.383	23.023	16.851	0.000	0.000	0.000	0.000
ENSG00000197635	10.707	-2.142	1.207	-1.774	0.076	0.191	37.957	7.339	37.670	5.911	12.409	9.345	10.130	7.723	0.000	0.000	0.000	0.000
ENSG00000107736	5.123	-2.052	1.092	-1.880	0.060	0.158	22.774	0.917	15.861	2.364	0.000	2.077	0.000	0.702	1.661	1.755	4.752	8.608
ENSG00000166396	20.256	-2.164	1.281	-1.690	0.091	0.221	21.509	63.301	25.774	57.929	13.139	21.805	10.130	29.489	0.000	0.000	0.000	0.000
ENSG00000279773	5.703	-2.023	1.093	-1.852	0.064	0.166	18.979	6.422	17.844	2.364	0.000	0.000	0.000	0.702	6.644	3.509	2.037	9.933
ENSG00000144583	35.793	-2.011	1.458	-1.379	0.168	0.356	17.713	135.775	16.852	117.039	45.986	38.418	33.153	24.574	0.000	0.000	0.000	0.000
ENSG00000243137	14.644	-2.092	1.132	-1.848	0.065	0.167	15.183	50.457	11.896	42.560	18.248	8.307	22.102	6.319	0.000	0.000	0.000	0.662
ENSG00000201405	2.191	-2.667	1.566	-1.704	0.088	0.216	13.918	0.000	4.957	1.182	3.650	0.000	0.921	0.000	1.661	0.000	0.000	0.000
ENSG00000271347	4.139	-2.087	1.139	-1.832	0.067	0.172	12.652	3.670	11.896	5.911	0.000	0.000	0.000	0.000	1.661	1.755	8.146	3.973
ENSG00000258171	2.446	-2.407	1.285	-1.873	0.061	0.160	11.387	0.917	6.939	2.364	0.000	0.000	0.000	0.000	0.000	1.755	1.358	4.635
ENSG00000086696	3.075	-2.088	1.262	-1.654	0.098	0.234	11.387	0.917	12.887	0.000	0.000	0.000	0.921	0.000	1.661	1.755	3.394	3.973
ENSG00000283848	2.938	-2.011	1.221	-1.648	0.099	0.237	11.387	6.422	5.948	0.000	1.460	3.115	5.525	1.404	0.000	0.000	0.000	0.000
ENSG00000248243	1.914	-2.109	1.076	-1.960	0.050	0.136	10.122	4.587	0.991	0.000	2.190	1.038	0.921	0.702	0.000	1.755	0.000	0.662
ENSG00000136011	1.695	-2.518	1.290	-1.952	0.051	0.138	10.122	0.917	2.974	1.182	0.000	0.000	0.000	2.808	1.661	0.000	0.679	0.000
ENSG00000281186	1.882	-2.732	1.484	-1.841	0.066	0.169	8.857	0.917	7.930	0.000	2.920	1.038	0.921	0.000	0.000	0.000	0.000	0.000
ENSG00000277007	2.215	-2.362	1.335	-1.769	0.077	0.193	8.857	0.000	8.922	1.182	0.000	0.000	0.921	0.000	3.322	0.000	2.715	0.662
ENSG00000225125	1.169	-2.843	1.693	-1.680	0.093	0.225	8.857	0.000	1.983	0.000	0.730	0.000	0.000	0.702	0.000	1.755	0.000	0.000
ENSG00000222032	12.492	-2.408	1.238	-1.945	0.052	0.140	7.591	43.118	8.922	49.653	5.110	14.537	5.525	15.447	0.000	0.000	0.000	0.000
ENSG00000253414	1.176	-2.914	1.572	-1.853	0.064	0.166	7.591	2.752	0.991	0.000	0.000	2.077	0.000	0.702	0.000	0.000	0.000	0.000
ENSG00000204866	10.542	-2.134	1.162	-1.836	0.066	0.171	7.591	35.779	10.904	33.102	11.679	7.268	11.051	9.127	0.000	0.000	0.000	0.000
ENSG00000227925	1.707	-4.878	2.893	-1.686	0.092	0.222	7.591	0.000	12.887	0.000	0.000	0.000	0.000	0.000	0.000	0.000	0.000	0.000
ENSG00000226674	1.230	-2.604	1.669	-1.560	0.119	0.273	7.591	0.000	3.965	0.000	0.000	0.000	1.842	0.702	0.000	0.000	0.000	0.662
ENSG00000185385	1.188	-2.425	1.252	-1.937	0.053	0.142	6.326	0.917	2.974	0.000	0.000	1.038	0.000	0.000	1.661	0.000	0.679	0.662
ENSG00000212237	1.466	-2.460	1.286	-1.914	0.056	0.148	6.326	0.917	3.965	2.364	0.000	0.000	0.000	0.000	0.000	0.000	2.037	1.987
ENSG00000231738	1.039	-4.157	2.174	-1.913	0.056	0.149	6.326	0.000	4.957	1.182	0.000	0.000	0.000	0.000	0.000	0.000	0.000	0.000
ENSG00000272824	1.017	-4.128	2.171	-1.901	0.057	0.152	6.326	0.917	4.957	0.000	0.000	0.000	0.000	0.000	0.000	0.000	0.000	0.000
ENSG00000171049	0.849	-3.391	1.826	-1.857	0.063	0.165	6.326	0.000	1.983	1.182	0.000	0.000	0.000	0.702	0.000	0.000	0.000	0.000
ENSG00000230710	0.934	-4.004	2.194	-1.825	0.068	0.174	6.326	0.917	3.965	0.000	0.000	0.000	0.000	0.000	0.000	0.000	0.000	0.000
ENSG00000268834	0.934	-4.004	2.194	-1.825	0.068	0.174	6.326	0.917	3.965	0.000	0.000	0.000	0.000	0.000	0.000	0.000	0.000	0.000
ENSG00000251539	2.316	-2.004	1.111	-1.803	0.071	0.181	6.326	1.835	1.983	8.276	0.730	0.000	0.921	4.213	0.000	3.509	0.000	0.000
ENSG00000274655	4.437	-2.145	1.192	-1.800	0.072	0.183	6.326	7.339	9.913	13.004	0.000	0.000	0.000	0.000	6.644	0.000	3.394	6.622
ENSG00000235505	0.874	-3.904	2.230	-1.751	0.080	0.199	6.326	0.000	2.974	1.182	0.000	0.000	0.000	0.000	0.000	0.000	0.000	0.000
ENSG00000206262	0.995	-3.178	1.843	-1.724	0.085	0.208	6.326	0.000	2.974	1.182	1.460	0.000	0.000	0.000	0.000	0.000	0.000	0.000
ENSG00000199332	0.741	-3.186	1.909	-1.669	0.095	0.229	6.326	0.917	0.991	0.000	0.000	0.000	0.000	0.000	0.000	0.000	0.000	0.662
ENSG00000274835	1.340	-2.186	1.429	-1.529	0.126	0.286	6.326	0.000	1.983	2.364	0.000	0.000	0.000	0.000	1.661	1.755	0.000	1.987
ENSG00000276980	1.694	-2.542	1.699	-1.496	0.135	0.301	6.326	0.000	7.930	1.182	0.000	2.077	0.000	2.808	0.000	0.000	0.000	0.000
ENSG00000221365	0.665	-3.019	2.533	-1.192	0.233	0.458	6.326	0.000	0.991	0.000	0.000	0.000	0.000	0.000	0.000	0.000	0.000	0.662
ENSG00000284134	0.775	-3.735	3.183	-1.174	0.241	0.468	6.326	0.000	2.974	0.000	0.000	0.000	0.000	0.000	0.000	0.000	0.000	0.000
ENSG00000261741	0.788	-2.286	2.078	-1.100	0.271	0.511	6.326	0.000	0.991	0.000	0.730	0.000	0.000	1.404	0.000	0.000	0.000	0.000
ENSG00000248973	0.604	-3.371	3.194	-1.055	0.291	0.538	6.326	0.917	0.000	0.000	0.000	0.000	0.000	0.000	0.000	0.000	0.000	0.000
ENSG00000010379	0.527	-3.171	3.201	-0.991	0.322	0.579	6.326	0.000	0.000	0.000	0.000	0.000	0.000	0.000	0.000	0.000	0.000	0.000
ENSG00000283156	1.339	-2.039	1.046	-1.949	0.051	0.139	5.061	1.835	3.965	0.000	0.730	0.000	0.000	0.702	0.000	1.755	1.358	0.662
ENSG00000214856	13.196	-2.104	1.081	-1.946	0.052	0.140	5.061	51.374	5.948	46.106	14.599	9.345	16.576	7.021	1.661	0.000	0.000	0.662
ENSG00000274987	1.469	-2.394	1.263	-1.895	0.058	0.154	5.061	0.000	5.948	1.182	0.000	0.000	0.000	0.000	1.661	1.755	1.358	0.662
ENSG00000236393	0.801	-3.315	1.781	-1.861	0.063	0.163	5.061	0.917	2.974	0.000	0.000	0.000	0.000	0.000	0.000	0.000	0.000	0.662
ENSG00000186652	0.790	-3.294	1.777	-1.854	0.064	0.166	5.061	2.752	0.991	0.000	0.000	0.000	0.000	0.000	0.000	0.000	0.679	0.000
ENSG00000092051	1.031	-2.291	1.243	-1.843	0.065	0.169	5.061	0.000	2.974	1.182	0.730	1.038	0.000	0.702	0.000	0.000	0.679	0.000
ENSG00000228330	0.867	-3.893	2.138	-1.821	0.069	0.176	5.061	0.000	2.974	2.364	0.000	0.000	0.000	0.000	0.000	0.000	0.000	0.000
ENSG00000269552	0.911	-3.971	2.192	-1.812	0.070	0.178	5.061	0.917	4.957	0.000	0.000	0.000	0.000	0.000	0.000	0.000	0.000	0.000
ENSG00000280639	0.970	-3.089	1.729	-1.786	0.074	0.187	5.061	2.752	1.983	0.000	0.000	0.000	1.842	0.000	0.000	0.000	0.000	0.000

List of genes downregulated in Normal-like vs Luminal-A cell-lines

Gene	baseMean	log2FoldChange	lfcSE	stat	pvalue	padj	MCF10A.RNA.Seq.Rep1_sorted	MCF10A.RNA.Seq.Rep2_sorted	MCF10A.RNA.Seq.Rep3_sorted	MCF10A.RNA.Seq.Rep4_sorted	MCF7.RNA.Seq.Rep1_sorted	MCF7.RNA.Seq.Rep2_sorted	MCF7.RNA.Seq.Rep3_sorted	MCF7.RNA.Seq.Rep4_sorted	ZR751.RNA.Seq.Rep1_sorted	ZR751.RNA.Seq.Rep2_sorted	ZR751.RNA.Seq.Rep3_sorted	ZR751.RNA.Seq.Rep4_sorted
ENSG00000285413	0.885	-2.396	1.376	-1.742	0.082	0.203	5.061	0.917	1.983	0.000	0.000	1.038	0.921	0.702	0.000	0.000	0.000	0.000
ENSG00000138722	0.829	-3.833	2.214	-1.731	0.083	0.206	5.061	0.917	3.965	0.000	0.000	0.000	0.000	0.000	0.000	0.000	0.000	0.000
ENSG00000232909	1.576	-2.120	1.236	-1.715	0.086	0.212	5.061	0.917	4.957	2.364	3.650	1.038	0.921	0.000	0.000	0.000	0.000	0.000
ENSG00000223387	0.977	-2.677	1.583	-1.692	0.091	0.220	5.061	0.000	0.991	3.547	1.460	0.000	0.000	0.000	0.000	0.000	0.000	0.662
ENSG00000283240	0.800	-3.774	2.254	-1.674	0.094	0.227	5.061	0.000	0.991	3.547	0.000	0.000	0.000	0.000	0.000	0.000	0.000	0.000
ENSG00000141505	1.000	-2.265	1.376	-1.645	0.100	0.238	5.061	0.917	2.974	0.000	1.460	0.000	0.921	0.000	0.000	0.000	0.000	0.662
ENSG00000254300	0.972	-3.120	1.897	-1.644	0.100	0.238	5.061	0.000	4.957	0.000	0.730	0.000	0.921	0.000	0.000	0.000	0.000	0.000
ENSG00000243305	1.704	-2.062	1.273	-1.620	0.105	0.248	5.061	0.000	7.930	1.182	0.730	0.000	2.763	2.106	0.000	0.000	0.679	0.000
ENSG00000169194	0.784	-2.575	1.600	-1.610	0.107	0.252	5.061	0.917	0.991	0.000	0.000	0.000	0.000	0.000	0.000	1.755	0.679	0.000
ENSG00000263120	0.658	-2.993	1.915	-1.562	0.118	0.271	5.061	0.000	0.991	1.182	0.000	0.000	0.000	0.000	0.000	0.000	0.000	0.662
ENSG00000263797	0.673	-3.527	2.306	-1.529	0.126	0.286	5.061	1.835	0.000	1.182	0.000	0.000	0.000	0.000	0.000	0.000	0.000	0.000
ENSG00000174482	0.657	-3.495	2.305	-1.516	0.129	0.292	5.061	1.835	0.991	0.000	0.000	0.000	0.000	0.000	0.000	0.000	0.000	0.000
ENSG00000241114	1.036	-2.058	1.367	-1.505	0.132	0.297	5.061	0.917	2.974	0.000	1.460	0.000	0.000	0.000	0.000	0.000	1.358	0.662
ENSG00000242611	0.950	-2.697	1.906	-1.415	0.157	0.339	5.061	0.000	1.983	2.364	0.000	0.000	0.000	0.000	0.000	0.000	0.000	1.987
ENSG00000255958	1.242	-2.803	2.018	-1.389	0.165	0.351	5.061	0.000	5.948	1.182	0.000	0.000	0.000	0.000	0.000	0.000	2.715	0.000
ENSG00000279205	1.107	-2.125	1.670	-1.272	0.203	0.413	5.061	0.000	0.991	2.364	0.000	0.000	0.000	0.000	0.000	3.509	1.358	0.000
ENSG00000256651	0.695	-2.356	2.003	-1.176	0.239	0.466	5.061	0.917	0.000	0.000	0.000	0.000	0.000	0.702	1.661	0.000	0.000	0.000
ENSG00000200033	0.752	-3.694	3.184	-1.160	0.246	0.475	5.061	0.000	3.965	0.000	0.000	0.000	0.000	0.000	0.000	0.000	0.000	0.000
ENSG00000207034	0.643	-2.743	2.503	-1.096	0.273	0.513	5.061	0.000	0.991	0.000	0.000	0.000	0.000	0.000	1.661	0.000	0.000	0.000
ENSG00000275343	0.560	-2.744	2.562	-1.071	0.284	0.529	5.061	0.000	0.991	0.000	0.000	0.000	0.000	0.000	0.000	0.000	0.000	0.662
ENSG00000184313	0.504	-3.110	3.204	-0.971	0.332	0.592	5.061	0.000	0.991	0.000	0.000	0.000	0.000	0.000	0.000	0.000	0.000	0.000
ENSG00000229859	0.504	-3.110	3.204	-0.971	0.332	0.592	5.061	0.000	0.991	0.000	0.000	0.000	0.000	0.000	0.000	0.000	0.000	0.000
ENSG00000229183	0.504	-3.110	3.204	-0.971	0.332	0.592	5.061	0.000	0.991	0.000	0.000	0.000	0.000	0.000	0.000	0.000	0.000	0.000
ENSG00000256713	0.504	-3.110	3.204	-0.971	0.332	0.592	5.061	0.000	0.991	0.000	0.000	0.000	0.000	0.000	0.000	0.000	0.000	0.000
ENSG00000187242	0.504	-3.110	3.204	-0.971	0.332	0.592	5.061	0.000	0.991	0.000	0.000	0.000	0.000	0.000	0.000	0.000	0.000	0.000
ENSG00000269873	0.565	-2.117	2.664	-0.795	0.427	0.703	5.061	0.000	0.000	0.000	0.000	1.038	0.000	0.000	0.000	0.000	0.679	0.000
ENSG00000260454	0.867	-3.435	1.766	-1.945	0.052	0.140	3.796	0.917	4.957	0.000	0.730	0.000	0.000	0.000	0.000	0.000	0.000	0.000
ENSG00000267675	0.876	-3.919	2.066	-1.897	0.058	0.153	3.796	2.752	3.965	0.000	0.000	0.000	0.000	0.000	0.000	0.000	0.000	0.000
ENSG00000146755	0.916	-2.552	1.349	-1.892	0.058	0.154	3.796	0.917	3.965	0.000	0.730	0.000	0.921	0.000	0.000	0.000	0.000	0.662
ENSG00000234997	1.208	-2.163	1.143	-1.891	0.059	0.155	3.796	2.752	0.000	3.547	0.000	0.000	0.000	1.404	1.661	0.000	0.679	0.662
ENSG00000203327	1.415	-2.004	1.063	-1.886	0.059	0.156	3.796	2.752	2.974	2.364	1.460	0.000	0.921	0.000	0.000	0.000	2.715	0.000
ENSG00000177306	0.776	-2.723	1.455	-1.872	0.061	0.160	3.796	1.835	1.983	0.000	0.000	1.038	0.000	0.000	0.000	0.000	0.000	0.662
ENSG00000255328	0.772	-2.723	1.456	-1.870	0.061	0.161	3.796	1.835	1.983	0.000	0.730	0.000	0.921	0.000	0.000	0.000	0.000	0.000
ENSG00000177459	1.604	-2.008	1.079	-1.860	0.063	0.164	3.796	1.835	4.957	2.364	1.460	2.077	2.763	0.000	0.000	0.000	0.000	0.000
ENSG00000280367	1.651	-2.887	1.553	-1.859	0.063	0.164	3.796	0.000	10.904	0.000	0.000	1.038	0.000	0.000	1.661	1.755	0.000	0.662
ENSG00000212172	1.235	-3.007	1.639	-1.834	0.067	0.172	3.796	0.000	7.930	0.000	0.000	0.000	0.000	0.000	0.000	1.755	0.679	0.662
ENSG00000278668	0.773	-3.086	1.693	-1.823	0.068	0.175	3.796	1.835	1.983	0.000	0.000	0.000	0.000	0.000	1.661	0.000	0.000	0.000
ENSG00000267922	0.940	-2.399	1.324	-1.813	0.070	0.178	3.796	1.835	0.000	2.364	0.000	0.000	0.921	0.702	1.661	0.000	0.000	0.000
ENSG00000274294	0.844	-3.857	2.135	-1.807	0.071	0.180	3.796	0.000	3.965	2.364	0.000	0.000	0.000	0.000	0.000	0.000	0.000	0.000
ENSG00000257732	0.693	-3.086	1.725	-1.789	0.074	0.186	3.796	1.835	1.983	0.000	0.000	0.000	0.000	0.702	0.000	0.000	0.000	0.000
ENSG00000251050	0.953	-3.064	1.723	-1.778	0.075	0.190	3.796	1.835	3.965	0.000	0.000	0.000	1.842	0.000	0.000	0.000	0.000	0.000
ENSG00000230838	0.885	-3.922	2.231	-1.758	0.079	0.197	3.796	0.917	0.000	5.911	0.000	0.000	0.000	0.000	0.000	0.000	0.000	0.000
ENSG00000130307	0.918	-2.364	1.357	-1.742	0.082	0.202	3.796	2.752	0.000	1.182	0.000	0.000	0.921	0.702	1.661	0.000	0.000	0.000
ENSG00000217702	1.303	-2.183	1.257	-1.737	0.082	0.204	3.796	0.917	5.948	0.000	0.000	1.038	0.921	0.000	1.661	0.000	1.358	0.000
ENSG00000257509	0.806	-3.796	2.224	-1.707	0.088	0.215	3.796	0.917	4.957	0.000	0.000	0.000	0.000	0.000	0.000	0.000	0.000	0.000
ENSG00000112706	1.280	-2.205	1.313	-1.679	0.093	0.225	3.796	1.835	0.991	4.729	0.000	0.000	0.000	0.000	0.000	0.000	1.358	2.649
ENSG00000236542	0.767	-2.515	1.513	-1.663	0.096	0.231	3.796	1.835	0.991	0.000	0.000	0.000	0.921	0.000	1.661	0.000	0.000	0.000
ENSG00000277095	0.705	-2.547	1.541	-1.653	0.098	0.235	3.796	1.835	0.000	1.182	0.730	0.000	0.921	0.000	0.000	0.000	0.000	0.000
ENSG00000275767	0.705	-2.547	1.541	-1.653	0.098	0.235	3.796	1.835	0.000	1.182	0.730	0.000	0.921	0.000	0.000	0.000	0.000	0.000
ENSG00000254703	0.721	-3.633	2.247	-1.617	0.106	0.249	3.796	3.670	0.000	1.182	0.000	0.000	0.000	0.000	0.000	0.000	0.000	0.000
ENSG00000189350	1.233	-2.052	1.277	-1.606	0.108	0.254	3.796	3.670	2.974	0.000	0.000	0.000	0.921	2.106	0.000	0.000	0.000	1.324
ENSG00000154678	1.492	-2.079	1.341	-1.551	0.121	0.276	3.796	5.504	0.000	2.364	0.000	0.000	0.000	1.404	0.000	3.509	0.000	1.324
ENSG00000117507	0.663	-3.507	2.285	-1.535	0.125	0.283	3.796	0.000	2.974	1.182	0.000	0.000	0.000	0.000	0.000	0.000	0.000	0.000
ENSG00000142615	0.748	-2.725	1.788	-1.524	0.127	0.288	3.796	1.835	1.983	0.000	0.000	0.000	0.000	0.000	0.000	0.000	1.358	0.000
ENSG00000262681	0.641	-3.462	2.284	-1.516	0.130	0.292	3.796	0.917	2.974	0.000	0.000	0.000	0.000	0.000	0.000	0.000	0.000	0.000
ENSG00000214694	1.435	-2.487	1.706	-1.457	0.145	0.319	3.796	0.917	7.930	0.000	2.920	0.000	0.000	0.000	1.661	0.000	0.000	0.000
ENSG00000250629	0.766	-2.751	1.920	-1.433	0.152	0.331	3.796	0.000	3.965	0.000	0.730	0.000	0.000	0.702	0.000	0.000	0.000	0.000
ENSG00000158815	0.574	-2.713	1.912	-1.419	0.156	0.337	3.796	0.000	0.991	1.182	0.000	0.000	0.921	0.000	0.000	0.000	0.000	0.000
ENSG00000118990	0.614	-2.656	1.874	-1.417	0.156	0.338	3.796	0.917	0.991	0.000	0.000	0.000	0.000	0.000	1.661	0.000	0.000	0.000
ENSG00000284289	0.595	-2.288	1.615	-1.417	0.157	0.338	3.796	0.917	0.991	0.000	0.730	0.000	0.000	0.702	0.000	0.000	0.000	0.000
ENSG00000229172	0.558	-2.713	1.920	-1.413	0.158	0.340	3.796	0.000	0.991	1.182	0.730	0.000	0.000	0.000	0.000	0.000	0.000	0.000
ENSG00000237324	0.553	-2.713	1.923	-1.411	0.158	0.341	3.796	0.000	0.991	1.182	0.000	0.000	0.000	0.000	0.000	0.000	0.000	0.662
ENSG00000134539	0.675	-2.535	1.879	-1.349	0.177	0.372	3.796	0.917	1.983	0.000	0.000	0.000	0.000	1.404	0.000	0.000	0.000	0.000
ENSG00000197919	0.497	-3.085	2.459	-1.254	0.210	0.422	3.796	0.000	0.991	1.182	0.000	0.000	0.000	0.000	0.000	0.000	0.000	0.000
ENSG00000263812	0.714	-2.313	1.920	-1.204	0.228	0.451	3.796	0.000	1.983	0.000	0.000	1.038	0.000	0.000	0.000	1.755	0.000	0.000
ENSG00000163746	0.568	-2.681	2.473	-1.084	0.278	0.520	3.796	0.000	1.983	0.000	0.000	1.038	0.000	0.000	0.000	0.000	0.000	0.000
ENSG00000249664	0.537	-2.682	2.498	-1.074	0.283	0.527	3.796	0.000	1.983	0.000	0.000	0.000	0.000	0.000	0.000	0.000	0.000	0.662
ENSG00000199072	0.564	-3.278	3.197	-1.025	0.305	0.557	3.796	0.000	2.974	0.000	0.000	0.000	0.000	0.000	0.000	0.000	0.000	0.000
ENSG00000273289	0.602	-2.029	1.999	-1.015	0.310	0.563	3.796	0.000	0.991	0.000	0.000	0.000	0.000	0.000	0.000	1.755	0.679	0.000
ENSG00000259644	0.594	-2.030	2.003	-1.013	0.311	0.565	3.796	0.000	0.991	0.000	0.000	0.000	0.000	0.000	1.661	0.000	0.679	0.000
ENSG00000274765	0.677	-2.010	2.021	-0.995	0.320	0.576	3.796	0.917	0.000	0.000	0.000	0.000	0.000	0.000	1.661	1.755	0.000	0.000
ENSG00000279164	0.492	-2.455	2.582	-0.951	0.342	0.605	3.796	0.000	0.000	1.182	0.000	0.000	0.921	0.000	0.000	0.000	0.000	0.000
ENSG00000273129	0.482	-3.047	3.206	-0.950	0.342	0.605	3.796	0.000	1.983	0.000	0.000	0.000	0.000	0.000	0.000	0.000	0.000	0.000
ENSG00000240687	0.537	-2.402	2.529	-0.950	0.342	0.605	3.796	0.000	0.991	0.000	0.000	0.000	0.000	0.000	1.661	0.000	0.000	0.000
ENSG00000124440	0.655	-2.321	2.494	-0.931	0.352	0.616	3.796	0.000	1.983	0.000	0.000	2.077	0.000	0.000	0.000	0.000	0.000	0.000
ENSG00000231762	0.460	-2.403	2.599	-0.925	0.355	0.620	3.796	0.000	0.991	0.000	0.730	0.000	0.000	0.000	0.000	0.000	0.000	0.000
ENSG00000267504	0.457	-2.404	2.602	-0.924	0.356	0.620	3.796	0.000	0.991	0.000	0.000	0.000	0.000	0.702	0.000	0.000	0.000	0.000
ENSG00000235070	0.415	-2.824	3.216	-0.878	0.380	0.650	3.796	0.000	0.000	1.182	0.000	0.000	0.000	0.000	0.000	0.000	0.000	0.000
ENSG00000227158	0.399	-2.770	3.219	-0.861	0.389	0.660	3.796	0.000	0.991	0.000	0.000	0.000	0.000	0.000	0.000	0.000	0.000	0.000
ENSG00000267361	0.399	-2.770	3.219	-0.861	0.389	0.660	3.796	0.000	0.991	0.000	0.000	0.000	0.000	0.000	0.000	0.000	0.000	0.000
ENSG00000271811	0.316	-2.426	3.239	-0.749	0.454	0.732	3.796	0.000	0.000	0.000	0.000	0.000	0.000	0.000	0.000	0.000	0.000	0.000
ENSG00000248112	0.316	-2.426	3.239	-0.749	0.454	0.732	3.796	0.000	0.000	0.000	0.000	0.000	0.000	0.000	0.000	0.000	0.000	0.000
ENSG00000225698	0.316	-2.426	3.239	-0.749	0.454	0.732	3.796	0.000	0.000	0.000	0.000	0.000	0.000	0.000	0.000	0.000	0.000	0.000
ENSG00000281264	0.316	-2.426	3.239	-0.749	0.454	0.732	3.796	0.000	0.000	0.000	0.000	0.000	0.000	0.000	0.000	0.000	0.000	0.000
ENSG00000201644	0.463	-2.066	3.239	-0.638	0.524	0.802	3.796	0.000	0.000	0.000	0.000	0.000	0.000	0.000	0.000	1.755	0.000	0.000
ENSG00000207460	0.741	-2.712	1.403	-1.933	0.053	0.143	2.530	1.835	1.983	1.182	0.000	0.000	0.000	0.000	0.000	0.000	1.358	0.000
ENSG00000235033	0.816	-2.879	1.536	-1.875	0.061	0.160	2.530	0.917	4.957	0.000	0.730	0.000	0.000	0.000	0.000	0.000	0.000	0.662
ENSG00000224459	0.775	-2.728	1.457	-1.872	0.061	0.160	2.530	2.752	0.000	2.364	0.730	0.000	0.921	0.000	0.000	0.000	0.000	0.000
ENSG00000283360	0.975	-2.558	1.371	-1.866	0.062	0.162	2.530	0.000	4.957	1.182	0.000	0.000	0.000	0.702	1.661	0.000	0.000	0.662
ENSG00000250934	0.732	-3.127	1.727	-1.811	0.070	0.179	2.530	0.000	2.974	2.364	0.000	0.000	0.921	0.000	0.000	0.000	0.000	0.000

List of genes downregulated in Normal-like vs Luminal-A cell-lines

Gene	baseMean	log2FoldChange	lfcSE	stat	pvalue	padj	MCF10A.RNA.Seq.Rep1_sorted	MCF10A.RNA.Seq.Rep2_sorted	MCF10A.RNA.Seq.Rep3_sorted	MCF10A.RNA.Seq.Rep4_sorted	MCF7.RNA.Seq.Rep1_sorted	MCF7.RNA.Seq.Rep2_sorted	MCF7.RNA.Seq.Rep3_sorted	MCF7.RNA.Seq.Rep4_sorted	ZR751.RNA.Seq.Rep1_sorted	ZR751.RNA.Seq.Rep2_sorted	ZR751.RNA.Seq.Rep3_sorted	ZR751.RNA.Seq.Rep4_sorted
ENSG00000278626	1.030	-2.019	1.127	-1.792	0.073	0.185	2.530	0.917	2.974	1.182	0.000	0.000	0.000	0.000	1.661	1.755	0.679	0.662
ENSG00000278611	1.125	-2.833	1.582	-1.790	0.073	0.186	2.530	0.917	6.939	0.000	0.000	0.000	0.000	0.000	0.000	1.755	1.358	0.000
ENSG00000273096	0.842	-2.140	1.217	-1.758	0.079	0.197	2.530	0.917	1.983	1.182	0.000	1.038	0.000	0.702	0.000	1.755	0.000	0.000
ENSG00000236591	1.022	-2.207	1.263	-1.748	0.080	0.200	2.530	0.917	2.974	2.364	0.000	2.077	0.000	1.404	0.000	0.000	0.000	0.000
ENSG00000157399	0.529	-2.631	1.517	-1.734	0.083	0.205	2.530	0.917	0.991	1.182	0.730	0.000	0.000	0.000	0.000	0.000	0.000	0.000
ENSG00000252147	0.962	-2.017	1.194	-1.690	0.091	0.221	2.530	0.917	2.974	1.182	0.000	0.000	0.921	0.000	1.661	0.000	1.358	0.000
ENSG00000155897	0.758	-3.700	2.192	-1.688	0.091	0.222	2.530	1.835	0.000	4.729	0.000	0.000	0.000	0.000	0.000	0.000	0.000	0.000
ENSG00000118520	0.691	-2.263	1.344	-1.684	0.092	0.223	2.530	0.917	0.991	1.182	0.000	0.000	0.921	0.000	0.000	1.755	0.000	0.000
ENSG00000175518	0.671	-2.263	1.347	-1.680	0.093	0.225	2.530	0.917	0.991	1.182	0.000	0.000	0.000	0.000	0.000	1.755	0.679	0.000
ENSG00000277050	1.543	-2.051	1.221	-1.680	0.093	0.225	2.530	0.917	6.939	2.364	0.000	0.000	3.684	1.404	0.000	0.000	0.679	0.000
ENSG00000229454	0.738	-3.666	2.189	-1.675	0.094	0.227	2.530	0.000	3.965	2.364	0.000	0.000	0.000	0.000	0.000	0.000	0.000	0.000
ENSG00000267372	0.738	-3.666	2.189	-1.675	0.094	0.227	2.530	0.000	3.965	2.364	0.000	0.000	0.000	0.000	0.000	0.000	0.000	0.000
ENSG00000267743	0.715	-2.579	1.550	-1.664	0.096	0.231	2.530	0.917	0.000	3.547	0.000	0.000	0.921	0.000	0.000	0.000	0.000	0.662
ENSG00000263264	0.468	-2.999	1.805	-1.661	0.097	0.232	2.530	0.917	0.991	1.182	0.000	0.000	0.000	0.000	0.000	0.000	0.000	0.000
ENSG00000267696	0.468	-2.999	1.805	-1.661	0.097	0.232	2.530	0.917	0.991	1.182	0.000	0.000	0.000	0.000	0.000	0.000	0.000	0.000
ENSG00000232528	0.638	-2.900	1.756	-1.651	0.099	0.236	2.530	1.835	0.000	2.364	0.000	0.000	0.921	0.000	0.000	0.000	0.000	0.000
ENSG00000277548	0.808	-2.163	1.313	-1.647	0.100	0.237	2.530	0.917	1.983	1.182	0.000	0.000	0.000	0.000	0.000	1.755	0.000	1.324
ENSG00000250725	0.796	-2.145	1.313	-1.633	0.102	0.243	2.530	1.835	0.991	1.182	0.000	0.000	0.000	0.000	1.661	0.000	1.358	0.000
ENSG00000154451	0.682	-2.490	1.527	-1.631	0.103	0.243	2.530	2.752	0.000	1.182	0.000	1.038	0.000	0.000	0.000	0.000	0.679	0.000
ENSG00000236098	0.683	-2.484	1.524	-1.630	0.103	0.244	2.530	0.917	2.974	0.000	0.730	1.038	0.000	0.000	0.000	0.000	0.000	0.000
ENSG00000275476	1.257	-2.241	1.410	-1.590	0.112	0.260	2.530	3.670	1.983	1.182	0.730	0.000	0.000	0.000	4.983	0.000	0.000	0.000
ENSG00000136542	0.622	-2.845	1.803	-1.578	0.115	0.265	2.530	0.917	2.974	0.000	0.000	1.038	0.000	0.000	0.000	0.000	0.000	0.000
ENSG00000284363	0.656	-3.493	2.225	-1.570	0.116	0.268	2.530	0.000	2.974	2.364	0.000	0.000	0.000	0.000	0.000	0.000	0.000	0.000
ENSG00000224260	0.596	-2.845	1.816	-1.567	0.117	0.269	2.530	0.917	2.974	0.000	0.730	0.000	0.000	0.000	0.000	0.000	0.000	0.000
ENSG00000271522	0.637	-3.454	2.223	-1.554	0.120	0.275	2.530	2.752	0.000	2.364	0.000	0.000	0.000	0.000	0.000	0.000	0.000	0.000
ENSG00000034239	0.637	-3.454	2.223	-1.554	0.120	0.275	2.530	2.752	0.000	2.364	0.000	0.000	0.000	0.000	0.000	0.000	0.000	0.000
ENSG00000175728	0.612	-3.401	2.220	-1.532	0.126	0.285	2.530	1.835	2.974	0.000	0.000	0.000	0.000	0.000	0.000	0.000	0.000	0.000
ENSG00000250433	0.727	-2.669	1.773	-1.505	0.132	0.297	2.530	2.752	1.983	0.000	1.460	0.000	0.000	0.000	0.000	0.000	0.000	0.000
ENSG00000273403	0.786	-2.358	1.577	-1.495	0.135	0.301	2.530	0.917	3.965	0.000	0.000	0.000	0.000	0.000	0.000	0.000	1.358	0.662
ENSG00000249731	0.618	-3.414	2.304	-1.482	0.138	0.307	2.530	0.917	3.965	0.000	0.000	0.000	0.000	0.000	0.000	0.000	0.000	0.000
ENSG00000267711	0.618	-3.414	2.304	-1.482	0.138	0.307	2.530	0.917	3.965	0.000	0.000	0.000	0.000	0.000	0.000	0.000	0.000	0.000
ENSG00000232642	0.652	-2.235	1.528	-1.463	0.143	0.316	2.530	0.917	1.983	0.000	0.730	0.000	0.000	0.000	1.661	0.000	0.000	0.000
ENSG00000261000	0.613	-2.654	1.816	-1.462	0.144	0.317	2.530	0.000	1.983	1.182	0.000	0.000	0.000	0.000	1.661	0.000	0.000	0.000
ENSG00000162367	0.582	-2.257	1.558	-1.448	0.148	0.323	2.530	1.835	0.000	1.182	0.730	0.000	0.000	0.702	0.000	0.000	0.000	0.000
ENSG00000231845	0.735	-2.694	1.860	-1.448	0.148	0.323	2.530	0.917	3.965	0.000	0.000	0.000	0.000	1.404	0.000	0.000	0.000	0.000
ENSG00000234807	0.574	-2.257	1.561	-1.446	0.148	0.324	2.530	1.835	0.000	1.182	0.000	0.000	0.000	0.000	0.000	0.000	0.679	0.662
ENSG00000101938	0.547	-2.695	1.865	-1.445	0.148	0.324	2.530	0.000	0.991	2.364	0.000	0.000	0.000	0.000	0.000	0.000	0.679	0.000
ENSG00000277152	0.543	-2.679	1.861	-1.439	0.150	0.327	2.530	0.917	0.000	2.364	0.000	0.000	0.000	0.702	0.000	0.000	0.000	0.000
ENSG00000254974	0.531	-2.655	1.859	-1.428	0.153	0.333	2.530	0.000	1.983	1.182	0.000	0.000	0.000	0.000	0.000	0.000	0.679	0.000
ENSG00000231307	0.513	-2.598	1.849	-1.405	0.160	0.344	2.530	0.917	1.983	0.000	0.730	0.000	0.000	0.000	0.000	0.000	0.000	0.000
ENSG00000230978	0.508	-2.599	1.852	-1.403	0.161	0.345	2.530	0.917	1.983	0.000	0.000	0.000	0.000	0.000	0.000	0.000	0.000	0.662
ENSG00000259367	0.529	-3.190	2.282	-1.398	0.162	0.347	2.530	1.835	1.983	0.000	0.000	0.000	0.000	0.000	0.000	0.000	0.000	0.000
ENSG00000250075	0.502	-2.582	1.850	-1.396	0.163	0.348	2.530	1.835	0.991	0.000	0.000	0.000	0.000	0.000	0.000	0.000	0.000	0.662
ENSG00000261568	0.557	-3.259	2.343	-1.391	0.164	0.351	2.530	0.000	2.974	1.182	0.000	0.000	0.000	0.000	0.000	0.000	0.000	0.000
ENSG00000229694	0.557	-3.259	2.343	-1.391	0.164	0.351	2.530	0.000	2.974	1.182	0.000	0.000	0.000	0.000	0.000	0.000	0.000	0.000
ENSG00000170276	0.557	-3.259	2.343	-1.391	0.164	0.351	2.530	0.000	2.974	1.182	0.000	0.000	0.000	0.000	0.000	0.000	0.000	0.000
ENSG00000150175	0.557	-3.259	2.343	-1.391	0.164	0.351	2.530	0.000	2.974	1.182	0.000	0.000	0.000	0.000	0.000	0.000	0.000	0.000
ENSG00000111405	0.557	-3.259	2.343	-1.391	0.164	0.351	2.530	0.000	2.974	1.182	0.000	0.000	0.000	0.000	0.000	0.000	0.000	0.000
ENSG00000268401	1.231	-3.321	2.439	-1.362	0.173	0.365	2.530	0.000	8.922	0.000	0.000	0.000	0.000	0.000	3.322	0.000	0.000	0.000
ENSG00000259616	0.763	-3.247	2.418	-1.343	0.179	0.375	2.530	0.000	5.948	0.000	0.000	0.000	0.000	0.000	0.000	0.000	0.679	0.000
ENSG00000275763	1.251	-2.204	1.711	-1.288	0.198	0.404	2.530	1.835	4.957	0.000	0.000	0.000	0.000	0.702	4.983	0.000	0.000	0.000
ENSG00000229976	0.475	-3.023	2.406	-1.256	0.209	0.421	2.530	0.000	1.983	1.182	0.000	0.000	0.000	0.000	0.000	0.000	0.000	0.000
ENSG00000221836	0.538	-2.367	1.893	-1.250	0.211	0.425	2.530	0.000	0.991	1.182	0.000	0.000	0.000	0.000	0.000	1.755	0.000	0.000
ENSG00000198670	0.462	-2.988	2.407	-1.241	0.214	0.430	2.530	1.835	0.000	1.182	0.000	0.000	0.000	0.000	0.000	0.000	0.000	0.000
ENSG00000174325	0.453	-2.961	2.408	-1.229	0.219	0.436	2.530	0.917	1.983	0.000	0.000	0.000	0.000	0.000	0.000	0.000	0.000	0.000
ENSG00000282804	0.453	-2.961	2.408	-1.229	0.219	0.436	2.530	0.917	1.983	0.000	0.000	0.000	0.000	0.000	0.000	0.000	0.000	0.000
ENSG00000271265	0.469	-2.368	1.935	-1.224	0.221	0.440	2.530	0.000	0.991	1.182	0.000	0.000	0.921	0.000	0.000	0.000	0.000	0.000
ENSG00000273240	0.446	-2.943	2.409	-1.222	0.222	0.441	2.530	1.835	0.991	0.000	0.000	0.000	0.000	0.000	0.000	0.000	0.000	0.000
ENSG00000175779	0.516	-2.299	1.884	-1.221	0.222	0.441	2.530	0.917	0.991	0.000	0.000	0.000	0.000	0.000	0.000	1.755	0.000	0.000
ENSG00000255165	0.453	-2.369	1.946	-1.217	0.224	0.443	2.530	0.000	0.991	1.182	0.730	0.000	0.000	0.000	0.000	0.000	0.000	0.000
ENSG00000228763	0.444	-2.349	1.946	-1.207	0.227	0.449	2.530	0.917	0.000	1.182	0.000	0.000	0.000	0.702	0.000	0.000	0.000	0.000
ENSG00000236166	0.428	-2.300	1.941	-1.185	0.236	0.462	2.530	0.917	0.991	0.000	0.000	0.000	0.000	0.702	0.000	0.000	0.000	0.000
ENSG00000275982	1.128	-2.066	1.748	-1.182	0.237	0.463	2.530	0.000	6.939	0.000	0.000	1.038	0.921	2.106	0.000	0.000	0.000	0.000
ENSG00000229635	0.588	-2.204	1.936	-1.138	0.255	0.488	2.530	2.752	0.000	0.000	0.730	1.038	0.000	0.000	0.000	0.000	0.000	0.000
ENSG00000083067	0.562	-2.749	2.497	-1.101	0.271	0.511	2.530	0.000	0.000	3.547	0.000	0.000	0.000	0.000	0.000	0.000	0.000	0.662
ENSG00000255882	0.392	-2.741	2.531	-1.083	0.279	0.521	2.530	0.000	0.991	1.182	0.000	0.000	0.000	0.000	0.000	0.000	0.000	0.000
ENSG00000237876	0.392	-2.741	2.531	-1.083	0.279	0.521	2.530	0.000	0.991	1.182	0.000	0.000	0.000	0.000	0.000	0.000	0.000	0.000
ENSG00000264705	0.392	-2.741	2.531	-1.083	0.279	0.521	2.530	0.000	0.991	1.182	0.000	0.000	0.000	0.000	0.000	0.000	0.000	0.000
ENSG00000215595	0.392	-2.741	2.531	-1.083	0.279	0.521	2.530	0.000	0.991	1.182	0.000	0.000	0.000	0.000	0.000	0.000	0.000	0.000
ENSG00000230542	0.386	-2.720	2.533	-1.074	0.283	0.527	2.530	0.917	0.000	1.182	0.000	0.000	0.000	0.000	0.000	0.000	0.000	0.000
ENSG00000263958	0.386	-2.720	2.533	-1.074	0.283	0.527	2.530	0.917	0.000	1.182	0.000	0.000	0.000	0.000	0.000	0.000	0.000	0.000
ENSG00000211976	0.624	-3.427	3.192	-1.074	0.283	0.527	2.530	0.000	4.957	0.000	0.000	0.000	0.000	0.000	0.000	0.000	0.000	0.000
ENSG00000123689	0.545	-2.618	2.473	-1.059	0.290	0.536	2.530	0.000	2.974	0.000	0.000	1.038	0.000	0.000	0.000	0.000	0.000	0.000
ENSG00000250990	0.535	-2.619	2.481	-1.056	0.291	0.538	2.530	0.000	2.974	0.000	0.000	0.000	0.921	0.000	0.000	0.000	0.000	0.000
ENSG00000234663	0.370	-2.666	2.538	-1.050	0.294	0.542	2.530	0.917	0.991	0.000	0.000	0.000	0.000	0.000	0.000	0.000	0.000	0.000
ENSG00000253851	0.520	-2.619	2.494	-1.050	0.294	0.542	2.530	0.000	2.974	0.000	0.730	0.000	0.000	0.000	0.000	0.000	0.000	0.000
ENSG00000283791	0.517	-2.619	2.496	-1.049	0.294	0.542	2.530	0.000	2.974	0.000	0.000	0.000	0.000	0.702	0.000	0.000	0.000	0.000
ENSG00000257221	0.517	-2.619	2.496	-1.049	0.294	0.542	2.530	0.000	2.974	0.000	0.000	0.000	0.000	0.702	0.000	0.000	0.000	0.000
ENSG00000261838	0.515	-2.619	2.498	-1.048	0.294	0.543	2.530	0.000	2.974	0.000	0.000	0.000	0.000	0.000	0.000	0.000	0.679	0.000
ENSG00000213512	0.514	-2.619	2.499	-1.048	0.295	0.543	2.530	0.000	2.974	0.000	0.000	0.000	0.000	0.000	0.000	0.000	0.000	0.662
ENSG00000237480	0.514	-2.619	2.499	-1.048	0.295	0.543	2.530	0.000	2.974	0.000	0.000	0.000	0.000	0.000	0.000	0.000	0.000	0.662
ENSG00000237422	0.541	-3.221	3.199	-1.007	0.314	0.568	2.530	0.000	3.965	0.000	0.000	0.000	0.000	0.000	0.000	0.000	0.000	0.000
ENSG00000258096	0.541	-3.221	3.199	-1.007	0.314	0.568	2.530	0.000	3.965	0.000	0.000	0.000	0.000	0.000	0.000	0.000	0.000	0.000
ENSG00000244376	0.541	-3.221	3.199	-1.007	0.314	0.568	2.530	0.000	3.965	0.000	0.000	0.000	0.000	0.000	0.000	0.000	0.000	0.000
ENSG00000225655	0.466	-2.433	2.551	-0.954	0.340	0.603	2.530	0.000	0.000	2.364	0.000	0.000	0.000	0.702	0.000	0.000	0.000	0.000
ENSG00000240893	0.515	-2.326	2.476	-0.940	0.347	0.611	2.530	0.000	1.983	0.000	0.000	0.000	0.000	0.000	1.661	0.000	0.000	0.000
ENSG00000236780	0.459	-2.981	3.209	-0.929	0.353	0.617	2.530	0.000	2.974	0.000	0.000	0.000	0.000	0.000	0.000	0.000	0.000	0.000
ENSG00000080293	0.459	-2.981	3.209	-0.929	0.353	0.617	2.530	0.000	2.974	0.000	0.000	0.000	0.000	0.000	0.000	0.000	0.000	0.000
ENSG00000249635	0.459	-2.981	3.209	-0.929	0.353	0.617	2.530	0.000	2.974	0.000	0.000	0.000	0.000	0.000	0.000	0.000	0.000	0.000
ENSG00000246790	0.459	-2.981	3.209	-0.929	0.353	0.617	2.530	0.000	2.974	0.000	0.000	0.000	0.000	0.000	0.000	0.000	0.000	0.000

List of genes downregulated in Normal-like vs Luminal-A cell-lines

Gene	baseMean	log2FoldChange	lfcSE	stat	pvalue	padj	MCF10A.RNA.Seq.Rep1_sorted	MCF10A.RNA.Seq.Rep2_sorted	MCF10A.RNA.Seq.Rep3_sorted	MCF10A.RNA.Seq.Rep4_sorted	MCF7.RNA.Seq.Rep1_sorted	MCF7.RNA.Seq.Rep2_sorted	MCF7.RNA.Seq.Rep3_sorted	MCF7.RNA.Seq.Rep4_sorted	ZR751.RNA.Seq.Rep1_sorted	ZR751.RNA.Seq.Rep2_sorted	ZR751.RNA.Seq.Rep3_sorted	ZR751.RNA.Seq.Rep4_sorted
ENSG00000272282	0.463	-2.327	2.525	-0.921	0.357	0.622	2.530	0.000	1.983	0.000	0.000	1.038	0.000	0.000	0.000	0.000	0.000	0.000
ENSG00000265799	0.463	-2.327	2.525	-0.921	0.357	0.622	2.530	0.000	1.983	0.000	0.000	1.038	0.000	0.000	0.000	0.000	0.000	0.000
ENSG00000267590	0.435	-2.327	2.555	-0.911	0.362	0.628	2.530	0.000	1.983	0.000	0.000	0.000	0.000	0.702	0.000	0.000	0.000	0.000
ENSG00000280232	0.433	-2.327	2.557	-0.910	0.363	0.629	2.530	0.000	1.983	0.000	0.000	0.000	0.000	0.000	0.000	0.000	0.679	0.000
ENSG00000232398	0.376	-2.691	3.223	-0.835	0.404	0.677	2.530	0.000	1.983	0.000	0.000	0.000	0.000	0.000	0.000	0.000	0.000	0.000
ENSG00000263426	0.376	-2.691	3.223	-0.835	0.404	0.677	2.530	0.000	1.983	0.000	0.000	0.000	0.000	0.000	0.000	0.000	0.000	0.000
ENSG00000244167	0.376	-2.691	3.223	-0.835	0.404	0.677	2.530	0.000	1.983	0.000	0.000	0.000	0.000	0.000	0.000	0.000	0.000	0.000
ENSG00000203286	0.376	-2.691	3.223	-0.835	0.404	0.677	2.530	0.000	1.983	0.000	0.000	0.000	0.000	0.000	0.000	0.000	0.000	0.000
ENSG00000258084	0.376	-2.691	3.223	-0.835	0.404	0.677	2.530	0.000	1.983	0.000	0.000	0.000	0.000	0.000	0.000	0.000	0.000	0.000
ENSG00000284388	0.376	-2.691	3.223	-0.835	0.404	0.677	2.530	0.000	1.983	0.000	0.000	0.000	0.000	0.000	0.000	0.000	0.000	0.000
ENSG00000283627	0.376	-2.691	3.223	-0.835	0.404	0.677	2.530	0.000	1.983	0.000	0.000	0.000	0.000	0.000	0.000	0.000	0.000	0.000
ENSG00000260741	0.376	-2.691	3.223	-0.835	0.404	0.677	2.530	0.000	1.983	0.000	0.000	0.000	0.000	0.000	0.000	0.000	0.000	0.000
ENSG00000188992	0.376	-2.691	3.223	-0.835	0.404	0.677	2.530	0.000	1.983	0.000	0.000	0.000	0.000	0.000	0.000	0.000	0.000	0.000
ENSG00000231636	0.561	-2.072	2.550	-0.813	0.416	0.692	2.530	0.000	0.000	2.364	0.000	0.000	1.842	0.000	0.000	0.000	0.000	0.000
ENSG00000284393	0.448	-2.025	2.573	-0.787	0.431	0.708	2.530	0.000	0.000	1.182	0.000	0.000	0.000	0.000	1.661	0.000	0.000	0.000
ENSG00000231256	0.386	-2.027	2.643	-0.767	0.443	0.722	2.530	0.000	0.000	1.182	0.000	0.000	0.921	0.000	0.000	0.000	0.000	0.000
ENSG00000241129	0.365	-2.027	2.671	-0.759	0.448	0.727	2.530	0.000	0.000	1.182	0.000	0.000	0.000	0.000	0.000	0.000	0.000	0.662
ENSG00000259946	0.309	-2.397	3.241	-0.740	0.460	0.739	2.530	0.000	0.000	1.182	0.000	0.000	0.000	0.000	0.000	0.000	0.000	0.000
ENSG00000228707	0.309	-2.397	3.241	-0.740	0.460	0.739	2.530	0.000	0.000	1.182	0.000	0.000	0.000	0.000	0.000	0.000	0.000	0.000
ENSG00000172148	0.309	-2.397	3.241	-0.740	0.460	0.739	2.530	0.000	0.000	1.182	0.000	0.000	0.000	0.000	0.000	0.000	0.000	0.000
ENSG00000231100	0.293	-2.327	3.246	-0.717	0.473	0.752	2.530	0.000	0.991	0.000	0.000	0.000	0.000	0.000	0.000	0.000	0.000	0.000
ENSG00000171094	0.293	-2.327	3.246	-0.717	0.473	0.752	2.530	0.000	0.991	0.000	0.000	0.000	0.000	0.000	0.000	0.000	0.000	0.000
ENSG00000241358	0.293	-2.327	3.246	-0.717	0.473	0.752	2.530	0.000	0.991	0.000	0.000	0.000	0.000	0.000	0.000	0.000	0.000	0.000
ENSG00000200469	0.293	-2.327	3.246	-0.717	0.473	0.752	2.530	0.000	0.991	0.000	0.000	0.000	0.000	0.000	0.000	0.000	0.000	0.000
ENSG00000236318	0.293	-2.327	3.246	-0.717	0.473	0.752	2.530	0.000	0.991	0.000	0.000	0.000	0.000	0.000	0.000	0.000	0.000	0.000
ENSG00000242948	0.293	-2.327	3.246	-0.717	0.473	0.752	2.530	0.000	0.991	0.000	0.000	0.000	0.000	0.000	0.000	0.000	0.000	0.000
ENSG00000233942	0.293	-2.327	3.246	-0.717	0.473	0.752	2.530	0.000	0.991	0.000	0.000	0.000	0.000	0.000	0.000	0.000	0.000	0.000
ENSG00000276405	0.293	-2.327	3.246	-0.717	0.473	0.752	2.530	0.000	0.991	0.000	0.000	0.000	0.000	0.000	0.000	0.000	0.000	0.000
ENSG00000207563	0.293	-2.327	3.246	-0.717	0.473	0.752	2.530	0.000	0.991	0.000	0.000	0.000	0.000	0.000	0.000	0.000	0.000	0.000
ENSG00000231483	0.293	-2.327	3.246	-0.717	0.473	0.752	2.530	0.000	0.991	0.000	0.000	0.000	0.000	0.000	0.000	0.000	0.000	0.000
ENSG00000273108	0.293	-2.327	3.246	-0.717	0.473	0.752	2.530	0.000	0.991	0.000	0.000	0.000	0.000	0.000	0.000	0.000	0.000	0.000
ENSG00000257595	0.293	-2.327	3.246	-0.717	0.473	0.752	2.530	0.000	0.991	0.000	0.000	0.000	0.000	0.000	0.000	0.000	0.000	0.000
ENSG00000080166	0.293	-2.327	3.246	-0.717	0.473	0.752	2.530	0.000	0.991	0.000	0.000	0.000	0.000	0.000	0.000	0.000	0.000	0.000
ENSG00000234177	0.287	-2.298	3.248	-0.708	0.479	0.758	2.530	0.917	0.000	0.000	0.000	0.000	0.000	0.000	0.000	0.000	0.000	0.000
ENSG00000272485	0.287	-2.298	3.248	-0.708	0.479	0.758	2.530	0.917	0.000	0.000	0.000	0.000	0.000	0.000	0.000	0.000	0.000	0.000
ENSG00000274507	0.287	-2.298	3.248	-0.708	0.479	0.758	2.530	0.917	0.000	0.000	0.000	0.000	0.000	0.000	0.000	0.000	0.000	0.000
ENSG00000269050	0.287	-2.298	3.248	-0.708	0.479	0.758	2.530	0.917	0.000	0.000	0.000	0.000	0.000	0.000	0.000	0.000	0.000	0.000
ENSG00000268036	0.287	-2.298	3.248	-0.708	0.479	0.758	2.530	0.917	0.000	0.000	0.000	0.000	0.000	0.000	0.000	0.000	0.000	0.000
ENSG00000275127	0.732	-2.489	1.284	-1.939	0.052	0.142	1.265	1.835	0.991	2.364	0.000	0.000	0.000	0.000	1.661	0.000	0.000	0.662
ENSG00000123560	0.727	-2.464	1.276	-1.932	0.053	0.144	1.265	1.835	1.983	1.182	0.000	0.000	0.000	0.702	0.000	1.755	0.000	0.000
ENSG00000257662	0.723	-2.464	1.276	-1.931	0.054	0.144	1.265	1.835	1.983	1.182	0.000	0.000	0.000	0.000	0.000	1.755	0.000	0.662
ENSG00000205325	1.115	-2.597	1.349	-1.925	0.054	0.145	1.265	0.917	4.957	3.547	0.000	0.000	0.000	0.702	0.000	0.000	0.000	1.987
ENSG00000202079	0.544	-3.226	1.699	-1.899	0.058	0.153	1.265	0.917	1.983	2.364	0.000	0.000	0.000	0.000	0.000	0.000	0.000	0.000
ENSG00000279469	0.571	-2.807	1.480	-1.897	0.058	0.153	1.265	2.752	0.991	1.182	0.000	0.000	0.000	0.000	0.000	0.000	0.000	0.662
ENSG00000224475	0.979	-2.262	1.194	-1.894	0.058	0.154	1.265	1.835	0.991	4.729	0.000	0.000	0.921	0.000	0.000	0.000	0.679	1.324
ENSG00000223774	0.538	-3.212	1.696	-1.894	0.058	0.154	1.265	1.835	0.991	2.364	0.000	0.000	0.000	0.000	0.000	0.000	0.000	0.000
ENSG00000277734	0.538	-3.212	1.696	-1.894	0.058	0.154	1.265	1.835	0.991	2.364	0.000	0.000	0.000	0.000	0.000	0.000	0.000	0.000
ENSG00000273664	0.538	-3.212	1.696	-1.894	0.058	0.154	1.265	1.835	0.991	2.364	0.000	0.000	0.000	0.000	0.000	0.000	0.000	0.000
ENSG00000224376	1.131	-2.560	1.352	-1.893	0.058	0.154	1.265	0.917	4.957	3.547	2.190	0.000	0.000	0.702	0.000	0.000	0.000	0.000
ENSG00000145423	0.980	-4.079	2.167	-1.882	0.060	0.157	1.265	4.587	0.000	5.911	0.000	0.000	0.000	0.000	0.000	0.000	0.000	0.000
ENSG00000277795	0.522	-3.178	1.689	-1.882	0.060	0.158	1.265	1.835	1.983	1.182	0.000	0.000	0.000	0.000	0.000	0.000	0.000	0.000
ENSG00000237484	0.816	-3.363	1.796	-1.872	0.061	0.160	1.265	5.504	0.000	2.364	0.000	0.000	0.000	0.000	0.000	0.000	0.000	0.662
ENSG00000254656	1.013	-2.424	1.296	-1.870	0.061	0.161	1.265	3.670	0.991	3.547	0.000	0.000	0.000	0.000	0.000	0.000	1.358	1.324
ENSG00000283952	1.143	-2.644	1.416	-1.867	0.062	0.162	1.265	7.339	0.000	2.364	0.730	0.000	0.000	0.000	0.000	0.000	1.358	0.662
ENSG00000271974	0.820	-3.346	1.820	-1.839	0.066	0.170	1.265	0.000	1.983	5.911	0.000	0.000	0.000	0.000	0.000	0.000	0.679	0.000
ENSG00000203462	0.905	-2.047	1.119	-1.829	0.067	0.173	1.265	0.917	1.983	3.547	0.730	1.038	0.000	0.702	0.000	0.000	0.679	0.000
ENSG00000234263	0.704	-2.644	1.519	-1.740	0.082	0.203	1.265	1.835	3.965	0.000	0.000	0.000	0.000	0.702	0.000	0.000	0.679	0.000
ENSG00000267260	0.852	-2.490	1.442	-1.727	0.084	0.208	1.265	0.917	5.948	0.000	0.730	0.000	0.000	0.702	0.000	0.000	0.000	0.662
ENSG00000267353	0.852	-2.490	1.442	-1.727	0.084	0.208	1.265	0.917	5.948	0.000	0.730	0.000	0.000	0.702	0.000	0.000	0.000	0.662
ENSG00000250678	0.518	-2.617	1.516	-1.726	0.084	0.208	1.265	0.917	0.991	2.364	0.000	0.000	0.000	0.000	0.000	0.000	0.679	0.000
ENSG00000172460	0.526	-2.568	1.495	-1.717	0.086	0.211	1.265	1.835	0.991	1.182	0.000	1.038	0.000	0.000	0.000	0.000	0.000	0.000
ENSG00000125675	0.805	-3.795	2.212	-1.715	0.086	0.212	1.265	3.670	0.000	4.729	0.000	0.000	0.000	0.000	0.000	0.000	0.000	0.000
ENSG00000224295	0.501	-2.582	1.510	-1.709	0.087	0.214	1.265	0.917	1.983	1.182	0.000	0.000	0.000	0.000	0.000	0.000	0.000	0.662
ENSG00000238406	0.495	-2.567	1.508	-1.703	0.089	0.216	1.265	1.835	0.991	1.182	0.000	0.000	0.000	0.000	0.000	0.000	0.000	0.662
ENSG00000260823	0.669	-3.035	1.799	-1.687	0.092	0.222	1.265	3.670	0.000	2.364	0.730	0.000	0.000	0.000	0.000	0.000	0.000	0.000
ENSG00000251139	0.865	-3.902	2.340	-1.668	0.095	0.229	1.265	0.000	7.930	1.182	0.000	0.000	0.000	0.000	0.000	0.000	0.000	0.000
ENSG00000196553	0.669	-2.222	1.335	-1.664	0.096	0.231	1.265	0.917	1.983	1.182	0.000	0.000	0.921	0.000	0.000	1.755	0.000	0.000
ENSG00000227260	0.446	-2.942	1.798	-1.636	0.102	0.241	1.265	0.917	1.983	1.182	0.000	0.000	0.000	0.000	0.000	0.000	0.000	0.000
ENSG00000255885	0.446	-2.942	1.798	-1.636	0.102	0.241	1.265	0.917	1.983	1.182	0.000	0.000	0.000	0.000	0.000	0.000	0.000	0.000
ENSG00000140274	0.446	-2.942	1.798	-1.636	0.102	0.241	1.265	0.917	1.983	1.182	0.000	0.000	0.000	0.000	0.000	0.000	0.000	0.000
ENSG00000152910	0.446	-2.942	1.798	-1.636	0.102	0.241	1.265	0.917	1.983	1.182	0.000	0.000	0.000	0.000	0.000	0.000	0.000	0.000
ENSG00000170209	0.439	-2.926	1.796	-1.629	0.103	0.244	1.265	1.835	0.991	1.182	0.000	0.000	0.000	0.000	0.000	0.000	0.000	0.000
ENSG00000257879	0.439	-2.926	1.796	-1.629	0.103	0.244	1.265	1.835	0.991	1.182	0.000	0.000	0.000	0.000	0.000	0.000	0.000	0.000
ENSG00000256732	0.439	-2.926	1.796	-1.629	0.103	0.244	1.265	1.835	0.991	1.182	0.000	0.000	0.000	0.000	0.000	0.000	0.000	0.000
ENSG00000212327	0.779	-2.114	1.301	-1.625	0.104	0.246	1.265	1.835	1.983	1.182	0.000	0.000	0.000	0.000	0.000	1.755	0.000	1.324
ENSG00000178690	0.644	-2.427	1.514	-1.603	0.109	0.255	1.265	1.835	2.974	0.000	0.730	0.000	0.921	0.000	0.000	0.000	0.000	0.000
ENSG00000226149	0.627	-2.880	1.814	-1.588	0.112	0.261	1.265	0.000	2.974	2.364	0.000	0.000	0.921	0.000	0.000	0.000	0.000	0.000
ENSG00000230461	0.611	-2.880	1.821	-1.581	0.114	0.263	1.265	0.000	2.974	2.364	0.730	0.000	0.000	0.000	0.000	0.000	0.000	0.000
ENSG00000272995	0.790	-2.127	1.350	-1.575	0.115	0.266	1.265	2.752	0.000	2.364	0.000	0.000	0.000	0.000	0.000	1.755	0.679	0.662
ENSG00000212724	0.685	-3.566	2.277	-1.566	0.117	0.270	1.265	4.587	0.000	2.364	0.000	0.000	0.000	0.000	0.000	0.000	0.000	0.000
ENSG00000271178	0.671	-3.541	2.277	-1.555	0.120	0.275	1.265	1.835	4.957	0.000	0.000	0.000	0.000	0.000	0.000	0.000	0.000	0.000
ENSG00000252516	0.826	-2.392	1.565	-1.528	0.126	0.286	1.265	0.000	2.974	3.547	1.460	0.000	0.000	0.000	0.000	0.000	0.000	0.662
ENSG00000274963	0.709	-2.130	1.431	-1.488	0.137	0.305	1.265	0.000	3.965	1.182	0.730	0.000	0.000	0.702	0.000	0.000	0.000	0.662
ENSG00000258945	0.608	-3.394	2.304	-1.473	0.141	0.312	1.265	3.670	0.000	2.364	0.000	0.000	0.000	0.000	0.000	0.000	0.000	0.000
ENSG00000271200	0.768	-2.078	1.412	-1.472	0.141	0.312	1.265	0.917	3.965	0.000	0.730	0.000	0.000	0.000	1.661	0.000	0.679	0.000
ENSG00000163219	0.501	-2.276	1.551	-1.468	0.142	0.314	1.265	0.917	0.991	1.182	0.000	0.000	0.000	0.000	1.661	0.000	0.000	0.000
ENSG00000263575	0.682	-2.085	1.423	-1.466	0.143	0.315	1.265	3.670	0.000	1.182	0.730	0.000	0.000	0.000	0.000	0.000	0.679	0.662
ENSG00000223403	0.615	-2.257	1.547	-1.459	0.145	0.318	1.265	0.917	0.991	2.364	0.000	0.000	1.842	0.000	0.000	0.000	0.000	0.000
ENSG00000234683	0.585	-2.272	1.562	-1.454	0.146	0.320	1.265	0.000	1.983	2.364	0.730	0.000	0.000	0.000	0.000	0.000	0.679	0.000
ENSG00000237033	0.918	-2.014	1.385	-1.454	0.146	0.320	1.265	0.000	3.965	2.364	0.000	2.077	0.000	0.000	0.000	0.000	0.679	0.662

List of genes downregulated in Normal-like vs Luminal-A cell-lines

Gene	baseMean	log2FoldChange	lfcSE	stat	pvalue	padj	MCF10A.RNA.Seq.Rep1_sorted	MCF10A.RNA.Seq.Rep2_sorted	MCF10A.RNA.Seq.Rep3_sorted	MCF10A.RNA.Seq.Rep4_sorted	MCF7.RNA.Seq.Rep1_sorted	MCF7.RNA.Seq.Rep2_sorted	MCF7.RNA.Seq.Rep3_sorted	MCF7.RNA.Seq.Rep4_sorted	ZR751.RNA.Seq.Rep1_sorted	ZR751.RNA.Seq.Rep2_sorted	ZR751.RNA.Seq.Rep3_sorted	ZR751.RNA.Seq.Rep4_sorted
ENSG00000204869	0.440	-2.277	1.577	-1.443	0.149	0.325	1.265	0.917	0.991	1.182	0.000	0.000	0.921	0.000	0.000	0.000	0.000	0.000
ENSG00000183169	0.440	-2.277	1.577	-1.443	0.149	0.325	1.265	0.917	0.991	1.182	0.000	0.000	0.921	0.000	0.000	0.000	0.000	0.000
ENSG00000265069	0.576	-3.324	2.303	-1.443	0.149	0.325	1.265	3.670	1.983	0.000	0.000	0.000	0.000	0.000	0.000	0.000	0.000	0.000
ENSG00000279667	0.567	-2.242	1.558	-1.439	0.150	0.328	1.265	1.835	0.000	2.364	0.000	0.000	0.000	0.000	0.000	0.000	0.679	0.662
ENSG00000258609	0.420	-2.277	1.587	-1.435	0.151	0.330	1.265	0.917	0.991	1.182	0.000	0.000	0.000	0.000	0.000	0.000	0.679	0.000
ENSG00000144671	0.648	-2.191	1.566	-1.399	0.162	0.347	1.265	2.752	0.000	1.182	0.000	0.000	0.921	0.000	1.661	0.000	0.000	0.000
ENSG00000251239	0.589	-2.236	1.601	-1.397	0.162	0.347	1.265	0.000	2.974	1.182	0.730	0.000	0.921	0.000	0.000	0.000	0.000	0.000
ENSG00000272542	0.635	-2.191	1.571	-1.395	0.163	0.349	1.265	2.752	0.000	1.182	0.000	0.000	0.000	0.000	0.000	1.755	0.000	0.662
ENSG00000277129	0.623	-2.185	1.572	-1.389	0.165	0.351	1.265	0.917	2.974	0.000	0.000	0.000	0.000	0.000	1.661	0.000	0.000	0.662
ENSG00000231648	0.363	-2.640	1.901	-1.389	0.165	0.352	1.265	0.917	0.991	1.182	0.000	0.000	0.000	0.000	0.000	0.000	0.000	0.000
ENSG00000259649	0.363	-2.640	1.901	-1.389	0.165	0.352	1.265	0.917	0.991	1.182	0.000	0.000	0.000	0.000	0.000	0.000	0.000	0.000
ENSG00000185390	0.554	-3.250	2.343	-1.387	0.165	0.352	1.265	1.835	0.000	3.547	0.000	0.000	0.000	0.000	0.000	0.000	0.000	0.000
ENSG00000226699	0.567	-2.184	1.592	-1.372	0.170	0.360	1.265	0.917	2.974	0.000	0.730	0.000	0.921	0.000	0.000	0.000	0.000	0.000
ENSG00000269989	0.582	-3.317	2.418	-1.372	0.170	0.360	1.265	0.000	0.991	4.729	0.000	0.000	0.000	0.000	0.000	0.000	0.000	0.000
ENSG00000180230	0.806	-2.151	1.568	-1.372	0.170	0.360	1.265	0.000	2.974	2.364	0.000	0.000	0.000	1.404	1.661	0.000	0.000	0.000
ENSG00000135577	0.576	-2.540	1.853	-1.371	0.170	0.360	1.265	0.917	2.974	0.000	0.000	0.000	0.000	0.000	0.000	1.755	0.000	0.000
ENSG00000274184	0.546	-2.184	1.600	-1.365	0.172	0.363	1.265	0.917	2.974	0.000	0.730	0.000	0.000	0.000	0.000	0.000	0.000	0.662
ENSG00000225857	0.562	-2.154	1.583	-1.360	0.174	0.366	1.265	2.752	0.991	0.000	0.000	1.038	0.000	0.702	0.000	0.000	0.000	0.000
ENSG00000266821	0.491	-2.540	1.901	-1.336	0.182	0.379	1.265	0.917	2.974	0.000	0.730	0.000	0.000	0.000	0.000	0.000	0.000	0.000
ENSG00000260125	0.500	-3.118	2.345	-1.330	0.184	0.382	1.265	2.752	1.983	0.000	0.000	0.000	0.000	0.000	0.000	0.000	0.000	0.000
ENSG00000172967	0.764	-2.660	2.003	-1.328	0.184	0.383	1.265	0.000	5.948	0.000	0.000	1.038	0.921	0.000	0.000	0.000	0.000	0.000
ENSG00000258225	0.534	-3.205	2.421	-1.324	0.186	0.385	1.265	0.000	3.965	1.182	0.000	0.000	0.000	0.000	0.000	0.000	0.000	0.000
ENSG00000279174	0.904	-2.226	1.724	-1.291	0.197	0.402	1.265	0.917	5.948	0.000	0.730	0.000	0.000	0.000	0.000	0.000	0.000	1.987
ENSG00000259477	0.679	-2.105	1.642	-1.282	0.200	0.407	1.265	0.917	3.965	0.000	0.000	0.000	0.000	0.000	0.000	0.000	0.679	1.324
ENSG00000250295	0.665	-2.052	1.633	-1.256	0.209	0.421	1.265	3.670	0.991	0.000	0.730	0.000	0.000	0.000	0.000	0.000	0.000	1.324
ENSG00000168843	0.769	-2.102	1.693	-1.242	0.214	0.429	1.265	0.000	4.957	0.000	0.000	0.000	0.000	0.000	1.661	0.000	0.679	0.662
ENSG00000270689	0.525	-2.328	1.888	-1.233	0.218	0.434	1.265	0.917	0.000	2.364	0.000	0.000	0.000	0.000	0.000	1.755	0.000	0.000
ENSG00000115297	0.849	-3.874	3.179	-1.219	0.223	0.442	1.265	0.000	8.922	0.000	0.000	0.000	0.000	0.000	0.000	0.000	0.000	0.000
ENSG00000158764	0.440	-2.349	1.948	-1.205	0.228	0.450	1.265	0.000	0.991	2.364	0.000	0.000	0.000	0.000	0.000	0.000	0.000	0.662
ENSG00000225649	0.452	-2.960	2.465	-1.201	0.230	0.453	1.265	0.000	2.974	1.182	0.000	0.000	0.000	0.000	0.000	0.000	0.000	0.000
ENSG00000266217	0.424	-2.877	2.412	-1.193	0.233	0.457	1.265	1.835	1.983	0.000	0.000	0.000	0.000	0.000	0.000	0.000	0.000	0.000
ENSG00000273540	0.424	-2.877	2.412	-1.193	0.233	0.457	1.265	1.835	1.983	0.000	0.000	0.000	0.000	0.000	0.000	0.000	0.000	0.000
ENSG00000260497	0.883	-3.007	2.529	-1.189	0.234	0.459	1.265	0.000	7.930	0.000	0.000	0.000	0.000	1.404	0.000	0.000	0.000	0.000
ENSG00000234773	0.605	-2.320	1.963	-1.182	0.237	0.463	1.265	0.000	0.991	3.547	1.460	0.000	0.000	0.000	0.000	0.000	0.000	0.000
ENSG00000225243	0.430	-2.896	2.469	-1.173	0.241	0.469	1.265	0.917	2.974	0.000	0.000	0.000	0.000	0.000	0.000	0.000	0.000	0.000
ENSG00000271225	0.430	-2.896	2.469	-1.173	0.241	0.469	1.265	0.917	2.974	0.000	0.000	0.000	0.000	0.000	0.000	0.000	0.000	0.000
ENSG00000280078	0.415	-2.259	1.938	-1.166	0.244	0.472	1.265	1.835	0.000	1.182	0.000	0.000	0.000	0.702	0.000	0.000	0.000	0.000
ENSG00000283980	0.413	-2.259	1.940	-1.165	0.244	0.473	1.265	1.835	0.000	1.182	0.000	0.000	0.000	0.000	0.000	0.000	0.679	0.000
ENSG00000278110	0.412	-2.259	1.941	-1.164	0.244	0.473	1.265	1.835	0.000	1.182	0.000	0.000	0.000	0.000	0.000	0.000	0.000	0.662
ENSG00000274717	0.412	-2.259	1.941	-1.164	0.244	0.473	1.265	1.835	0.000	1.182	0.000	0.000	0.000	0.000	0.000	0.000	0.000	0.662
ENSG00000230630	0.417	-2.858	2.472	-1.156	0.248	0.478	1.265	2.752	0.991	0.000	0.000	0.000	0.000	0.000	0.000	0.000	0.000	0.000
ENSG00000282890	0.417	-2.858	2.472	-1.156	0.248	0.478	1.265	2.752	0.991	0.000	0.000	0.000	0.000	0.000	0.000	0.000	0.000	0.000
ENSG00000235076	0.418	-2.209	1.920	-1.151	0.250	0.481	1.265	1.835	0.991	0.000	0.000	0.000	0.921	0.000	0.000	0.000	0.000	0.000
ENSG00000254254	0.402	-2.209	1.933	-1.143	0.253	0.485	1.265	1.835	0.991	0.000	0.730	0.000	0.000	0.000	0.000	0.000	0.000	0.000
ENSG00000211772	0.684	-3.562	3.188	-1.117	0.264	0.501	1.265	0.000	6.939	0.000	0.000	0.000	0.000	0.000	0.000	0.000	0.000	0.000
ENSG00000271555	0.575	-2.804	2.538	-1.105	0.269	0.508	1.265	0.000	4.957	0.000	0.000	0.000	0.000	0.000	0.000	0.000	0.679	0.000
ENSG00000230020	0.385	-2.719	2.533	-1.073	0.283	0.528	1.265	0.000	0.991	2.364	0.000	0.000	0.000	0.000	0.000	0.000	0.000	0.000
ENSG00000258808	0.385	-2.719	2.533	-1.073	0.283	0.528	1.265	0.000	0.991	2.364	0.000	0.000	0.000	0.000	0.000	0.000	0.000	0.000
ENSG00000278998	0.379	-2.697	2.535	-1.064	0.287	0.533	1.265	0.917	0.000	2.364	0.000	0.000	0.000	0.000	0.000	0.000	0.000	0.000
ENSG00000268674	0.379	-2.697	2.535	-1.064	0.287	0.533	1.265	0.917	0.000	2.364	0.000	0.000	0.000	0.000	0.000	0.000	0.000	0.000
ENSG00000264384	0.369	-2.665	2.538	-1.050	0.294	0.542	1.265	0.000	1.983	1.182	0.000	0.000	0.000	0.000	0.000	0.000	0.000	0.000
ENSG00000236153	0.369	-2.665	2.538	-1.050	0.294	0.542	1.265	0.000	1.983	1.182	0.000	0.000	0.000	0.000	0.000	0.000	0.000	0.000
ENSG00000188037	0.369	-2.665	2.538	-1.050	0.294	0.542	1.265	0.000	1.983	1.182	0.000	0.000	0.000	0.000	0.000	0.000	0.000	0.000
ENSG00000249086	0.369	-2.665	2.538	-1.050	0.294	0.542	1.265	0.000	1.983	1.182	0.000	0.000	0.000	0.000	0.000	0.000	0.000	0.000
ENSG00000139648	0.369	-2.665	2.538	-1.050	0.294	0.542	1.265	0.000	1.983	1.182	0.000	0.000	0.000	0.000	0.000	0.000	0.000	0.000
ENSG00000277977	0.369	-2.665	2.538	-1.050	0.294	0.542	1.265	0.000	1.983	1.182	0.000	0.000	0.000	0.000	0.000	0.000	0.000	0.000
ENSG00000178403	0.357	-2.620	2.542	-1.031	0.303	0.554	1.265	1.835	0.000	1.182	0.000	0.000	0.000	0.000	0.000	0.000	0.000	0.000
ENSG00000253357	0.357	-2.620	2.542	-1.031	0.303	0.554	1.265	1.835	0.000	1.182	0.000	0.000	0.000	0.000	0.000	0.000	0.000	0.000
ENSG00000224992	0.357	-2.620	2.542	-1.031	0.303	0.554	1.265	1.835	0.000	1.182	0.000	0.000	0.000	0.000	0.000	0.000	0.000	0.000
ENSG00000070031	0.357	-2.620	2.542	-1.031	0.303	0.554	1.265	1.835	0.000	1.182	0.000	0.000	0.000	0.000	0.000	0.000	0.000	0.000
ENSG00000258553	0.357	-2.620	2.542	-1.031	0.303	0.554	1.265	1.835	0.000	1.182	0.000	0.000	0.000	0.000	0.000	0.000	0.000	0.000
ENSG00000164265	0.347	-2.586	2.546	-1.016	0.310	0.563	1.265	0.917	1.983	0.000	0.000	0.000	0.000	0.000	0.000	0.000	0.000	0.000
ENSG00000124818	0.347	-2.586	2.546	-1.016	0.310	0.563	1.265	0.917	1.983	0.000	0.000	0.000	0.000	0.000	0.000	0.000	0.000	0.000
ENSG00000101812	0.347	-2.586	2.546	-1.016	0.310	0.563	1.265	0.917	1.983	0.000	0.000	0.000	0.000	0.000	0.000	0.000	0.000	0.000
ENSG00000259584	0.347	-2.586	2.546	-1.016	0.310	0.563	1.265	0.917	1.983	0.000	0.000	0.000	0.000	0.000	0.000	0.000	0.000	0.000
ENSG00000280180	0.347	-2.586	2.546	-1.016	0.310	0.563	1.265	0.917	1.983	0.000	0.000	0.000	0.000	0.000	0.000	0.000	0.000	0.000
ENSG00000120337	0.341	-2.563	2.548	-1.006	0.314	0.569	1.265	1.835	0.991	0.000	0.000	0.000	0.000	0.000	0.000	0.000	0.000	0.000
ENSG00000232377	0.341	-2.563	2.548	-1.006	0.314	0.569	1.265	1.835	0.991	0.000	0.000	0.000	0.000	0.000	0.000	0.000	0.000	0.000
ENSG00000277474	0.341	-2.563	2.548	-1.006	0.314	0.569	1.265	1.835	0.991	0.000	0.000	0.000	0.000	0.000	0.000	0.000	0.000	0.000
ENSG00000239511	0.341	-2.563	2.548	-1.006	0.314	0.569	1.265	1.835	0.991	0.000	0.000	0.000	0.000	0.000	0.000	0.000	0.000	0.000
ENSG00000174680	0.491	-2.553	2.570	-0.993	0.320	0.577	1.265	0.000	3.965	0.000	0.000	0.000	0.000	0.000	0.000	0.000	0.000	0.662
ENSG00000231170	0.518	-3.163	3.201	-0.988	0.323	0.581	1.265	0.000	4.957	0.000	0.000	0.000	0.000	0.000	0.000	0.000	0.000	0.000
ENSG00000212407	0.518	-3.163	3.201	-0.988	0.323	0.581	1.265	0.000	4.957	0.000	0.000	0.000	0.000	0.000	0.000	0.000	0.000	0.000
ENSG00000183674	0.500	-3.098	3.204	-0.967	0.334	0.594	1.265	0.000	0.000	4.729	0.000	0.000	0.000	0.000	0.000	0.000	0.000	0.000
ENSG00000198573	0.488	-2.411	2.575	-0.937	0.349	0.613	1.265	0.000	0.000	3.547	0.000	1.038	0.000	0.000	0.000	0.000	0.000	0.000
ENSG00000180613	0.436	-2.912	3.212	-0.907	0.365	0.631	1.265	0.000	3.965	0.000	0.000	0.000	0.000	0.000	0.000	0.000	0.000	0.000
ENSG00000260510	0.436	-2.912	3.212	-0.907	0.365	0.631	1.265	0.000	3.965	0.000	0.000	0.000	0.000	0.000	0.000	0.000	0.000	0.000
ENSG00000260532	0.440	-2.248	2.579	-0.872	0.383	0.654	1.265	0.000	2.974	0.000	0.000	1.038	0.000	0.000	0.000	0.000	0.000	0.000
ENSG00000259571	0.401	-2.778	3.219	-0.863	0.388	0.659	1.265	0.000	0.000	3.547	0.000	0.000	0.000	0.000	0.000	0.000	0.000	0.000
ENSG00000272265	0.287	-2.291	2.681	-0.855	0.393	0.664	1.265	0.000	0.991	1.182	0.000	0.000	0.000	0.000	0.000	0.000	0.000	0.000
ENSG00000273997	0.287	-2.291	2.681	-0.855	0.393	0.664	1.265	0.000	0.991	1.182	0.000	0.000	0.000	0.000	0.000	0.000	0.000	0.000
ENSG00000233816	0.287	-2.291	2.681	-0.855	0.393	0.664	1.265	0.000	0.991	1.182	0.000	0.000	0.000	0.000	0.000	0.000	0.000	0.000
ENSG00000135409	0.287	-2.291	2.681	-0.855	0.393	0.664	1.265	0.000	0.991	1.182	0.000	0.000	0.000	0.000	0.000	0.000	0.000	0.000
ENSG00000256705	0.287	-2.291	2.681	-0.855	0.393	0.664	1.265	0.000	0.991	1.182	0.000	0.000	0.000	0.000	0.000	0.000	0.000	0.000
ENSG00000283906	0.287	-2.291	2.681	-0.855	0.393	0.664	1.265	0.000	0.991	1.182	0.000	0.000	0.000	0.000	0.000	0.000	0.000	0.000
ENSG00000278123	0.287	-2.291	2.681	-0.855	0.393	0.664	1.265	0.000	0.991	1.182	0.000	0.000	0.000	0.000	0.000	0.000	0.000	0.000
ENSG00000266654	0.287	-2.291	2.681	-0.855	0.393	0.664	1.265	0.000	0.991	1.182	0.000	0.000	0.000	0.000	0.000	0.000	0.000	0.000
ENSG00000273112	0.280	-2.263	2.685	-0.843	0.399	0.671	1.265	0.917	0.000	1.182	0.000	0.000	0.000	0.000	0.000	0.000	0.000	0.000
ENSG00000226819	0.280	-2.263	2.685	-0.843	0.399	0.671	1.265	0.917	0.000	1.182	0.000	0.000	0.000	0.000	0.000	0.000	0.000	0.000
ENSG00000267784	0.280	-2.263	2.685	-0.843	0.399	0.671	1.265	0.917	0.000	1.182	0.000	0.000	0.000	0.000	0.000	0.000	0.000	0.000

List of genes downregulated in Normal-like vs Luminal-A cell-lines

Gene	baseMean	log2FoldChange	lfcSE	stat	pvalue	padj	MCF10A.RNA.Seq.Rep1_sorted	MCF10A.RNA.Seq.Rep2_sorted	MCF10A.RNA.Seq.Rep3_sorted	MCF10A.RNA.Seq.Rep4_sorted	MCF7.RNA.Seq.Rep1_sorted	MCF7.RNA.Seq.Rep2_sorted	MCF7.RNA.Seq.Rep3_sorted	MCF7.RNA.Seq.Rep4_sorted	ZR751.RNA.Seq.Rep1_sorted	ZR751.RNA.Seq.Rep2_sorted	ZR751.RNA.Seq.Rep3_sorted	ZR751.RNA.Seq.Rep4_sorted
ENSG00000213126	0.280	-2.263	2.685	-0.843	0.399	0.671	1.265	0.917	0.000	1.182	0.000	0.000	0.000	0.000	0.000	0.000	0.000	0.000
ENSG00000270497	0.280	-2.263	2.685	-0.843	0.399	0.671	1.265	0.917	0.000	1.182	0.000	0.000	0.000	0.000	0.000	0.000	0.000	0.000
ENSG00000255532	0.280	-2.263	2.685	-0.843	0.399	0.671	1.265	0.917	0.000	1.182	0.000	0.000	0.000	0.000	0.000	0.000	0.000	0.000
ENSG00000264015	0.280	-2.263	2.685	-0.843	0.399	0.671	1.265	0.917	0.000	1.182	0.000	0.000	0.000	0.000	0.000	0.000	0.000	0.000
ENSG00000229162	0.264	-2.191	2.697	-0.812	0.417	0.692	1.265	0.917	0.991	0.000	0.000	0.000	0.000	0.000	0.000	0.000	0.000	0.000
ENSG00000259056	0.264	-2.191	2.697	-0.812	0.417	0.692	1.265	0.917	0.991	0.000	0.000	0.000	0.000	0.000	0.000	0.000	0.000	0.000
ENSG00000223466	0.264	-2.191	2.697	-0.812	0.417	0.692	1.265	0.917	0.991	0.000	0.000	0.000	0.000	0.000	0.000	0.000	0.000	0.000
ENSG00000279964	0.264	-2.191	2.697	-0.812	0.417	0.692	1.265	0.917	0.991	0.000	0.000	0.000	0.000	0.000	0.000	0.000	0.000	0.000
ENSG00000235908	0.264	-2.191	2.697	-0.812	0.417	0.692	1.265	0.917	0.991	0.000	0.000	0.000	0.000	0.000	0.000	0.000	0.000	0.000
ENSG00000272609	0.264	-2.191	2.697	-0.812	0.417	0.692	1.265	0.917	0.991	0.000	0.000	0.000	0.000	0.000	0.000	0.000	0.000	0.000
ENSG00000249928	0.264	-2.191	2.697	-0.812	0.417	0.692	1.265	0.917	0.991	0.000	0.000	0.000	0.000	0.000	0.000	0.000	0.000	0.000
ENSG00000236536	0.264	-2.191	2.697	-0.812	0.417	0.692	1.265	0.917	0.991	0.000	0.000	0.000	0.000	0.000	0.000	0.000	0.000	0.000
ENSG00000248100	0.264	-2.191	2.697	-0.812	0.417	0.692	1.265	0.917	0.991	0.000	0.000	0.000	0.000	0.000	0.000	0.000	0.000	0.000
ENSG00000257572	0.264	-2.191	2.697	-0.812	0.417	0.692	1.265	0.917	0.991	0.000	0.000	0.000	0.000	0.000	0.000	0.000	0.000	0.000
ENSG00000257759	0.264	-2.191	2.697	-0.812	0.417	0.692	1.265	0.917	0.991	0.000	0.000	0.000	0.000	0.000	0.000	0.000	0.000	0.000
ENSG00000279147	0.353	-2.608	3.228	-0.808	0.419	0.695	1.265	0.000	2.974	0.000	0.000	0.000	0.000	0.000	0.000	0.000	0.000	0.000
ENSG00000285081	0.353	-2.608	3.228	-0.808	0.419	0.695	1.265	0.000	2.974	0.000	0.000	0.000	0.000	0.000	0.000	0.000	0.000	0.000
ENSG00000106128	0.353	-2.608	3.228	-0.808	0.419	0.695	1.265	0.000	2.974	0.000	0.000	0.000	0.000	0.000	0.000	0.000	0.000	0.000
ENSG00000273584	0.353	-2.608	3.228	-0.808	0.419	0.695	1.265	0.000	2.974	0.000	0.000	0.000	0.000	0.000	0.000	0.000	0.000	0.000
ENSG00000276030	0.353	-2.608	3.228	-0.808	0.419	0.695	1.265	0.000	2.974	0.000	0.000	0.000	0.000	0.000	0.000	0.000	0.000	0.000
ENSG00000224916	0.353	-2.608	3.228	-0.808	0.419	0.695	1.265	0.000	2.974	0.000	0.000	0.000	0.000	0.000	0.000	0.000	0.000	0.000
ENSG00000269902	0.335	-2.536	3.232	-0.785	0.433	0.710	1.265	2.752	0.000	0.000	0.000	0.000	0.000	0.000	0.000	0.000	0.000	0.000
ENSG00000233509	0.302	-2.367	3.243	-0.730	0.465	0.744	1.265	0.000	0.000	2.364	0.000	0.000	0.000	0.000	0.000	0.000	0.000	0.000
ENSG00000239602	0.302	-2.367	3.243	-0.730	0.465	0.744	1.265	0.000	0.000	2.364	0.000	0.000	0.000	0.000	0.000	0.000	0.000	0.000
ENSG00000261618	0.302	-2.367	3.243	-0.730	0.465	0.744	1.265	0.000	0.000	2.364	0.000	0.000	0.000	0.000	0.000	0.000	0.000	0.000
ENSG00000229291	0.271	-2.221	3.253	-0.683	0.495	0.774	1.265	0.000	1.983	0.000	0.000	0.000	0.000	0.000	0.000	0.000	0.000	0.000
ENSG00000234584	0.271	-2.221	3.253	-0.683	0.495	0.774	1.265	0.000	1.983	0.000	0.000	0.000	0.000	0.000	0.000	0.000	0.000	0.000
ENSG00000243572	0.271	-2.221	3.253	-0.683	0.495	0.774	1.265	0.000	1.983	0.000	0.000	0.000	0.000	0.000	0.000	0.000	0.000	0.000
ENSG00000249036	0.271	-2.221	3.253	-0.683	0.495	0.774	1.265	0.000	1.983	0.000	0.000	0.000	0.000	0.000	0.000	0.000	0.000	0.000
ENSG00000145642	0.271	-2.221	3.253	-0.683	0.495	0.774	1.265	0.000	1.983	0.000	0.000	0.000	0.000	0.000	0.000	0.000	0.000	0.000
ENSG00000272810	0.271	-2.221	3.253	-0.683	0.495	0.774	1.265	0.000	1.983	0.000	0.000	0.000	0.000	0.000	0.000	0.000	0.000	0.000
ENSG00000221845	0.271	-2.221	3.253	-0.683	0.495	0.774	1.265	0.000	1.983	0.000	0.000	0.000	0.000	0.000	0.000	0.000	0.000	0.000
ENSG00000199051	0.271	-2.221	3.253	-0.683	0.495	0.774	1.265	0.000	1.983	0.000	0.000	0.000	0.000	0.000	0.000	0.000	0.000	0.000
ENSG00000212273	0.271	-2.221	3.253	-0.683	0.495	0.774	1.265	0.000	1.983	0.000	0.000	0.000	0.000	0.000	0.000	0.000	0.000	0.000
ENSG00000228322	0.271	-2.221	3.253	-0.683	0.495	0.774	1.265	0.000	1.983	0.000	0.000	0.000	0.000	0.000	0.000	0.000	0.000	0.000
ENSG00000237385	0.271	-2.221	3.253	-0.683	0.495	0.774	1.265	0.000	1.983	0.000	0.000	0.000	0.000	0.000	0.000	0.000	0.000	0.000
ENSG00000269256	0.271	-2.221	3.253	-0.683	0.495	0.774	1.265	0.000	1.983	0.000	0.000	0.000	0.000	0.000	0.000	0.000	0.000	0.000
ENSG00000223576	0.271	-2.221	3.253	-0.683	0.495	0.774	1.265	0.000	1.983	0.000	0.000	0.000	0.000	0.000	0.000	0.000	0.000	0.000
ENSG00000279050	0.271	-2.221	3.253	-0.683	0.495	0.774	1.265	0.000	1.983	0.000	0.000	0.000	0.000	0.000	0.000	0.000	0.000	0.000
ENSG00000259763	0.271	-2.221	3.253	-0.683	0.495	0.774	1.265	0.000	1.983	0.000	0.000	0.000	0.000	0.000	0.000	0.000	0.000	0.000
ENSG00000184471	0.271	-2.221	3.253	-0.683	0.495	0.774	1.265	0.000	1.983	0.000	0.000	0.000	0.000	0.000	0.000	0.000	0.000	0.000
ENSG00000265331	0.271	-2.221	3.253	-0.683	0.495	0.774	1.265	0.000	1.983	0.000	0.000	0.000	0.000	0.000	0.000	0.000	0.000	0.000
ENSG00000283385	0.271	-2.221	3.253	-0.683	0.495	0.774	1.265	0.000	1.983	0.000	0.000	0.000	0.000	0.000	0.000	0.000	0.000	0.000
ENSG00000234906	0.271	-2.221	3.253	-0.683	0.495	0.774	1.265	0.000	1.983	0.000	0.000	0.000	0.000	0.000	0.000	0.000	0.000	0.000
ENSG00000207550	0.271	-2.221	3.253	-0.683	0.495	0.774	1.265	0.000	1.983	0.000	0.000	0.000	0.000	0.000	0.000	0.000	0.000	0.000
ENSG00000066032	0.258	-2.159	3.258	-0.663	0.508	0.786	1.265	1.835	0.000	0.000	0.000	0.000	0.000	0.000	0.000	0.000	0.000	0.000
ENSG00000224963	0.258	-2.159	3.258	-0.663	0.508	0.786	1.265	1.835	0.000	0.000	0.000	0.000	0.000	0.000	0.000	0.000	0.000	0.000
ENSG00000224269	0.258	-2.159	3.258	-0.663	0.508	0.786	1.265	1.835	0.000	0.000	0.000	0.000	0.000	0.000	0.000	0.000	0.000	0.000
ENSG00000231713	0.258	-2.159	3.258	-0.663	0.508	0.786	1.265	1.835	0.000	0.000	0.000	0.000	0.000	0.000	0.000	0.000	0.000	0.000
ENSG00000120664	1.270	-2.511	1.301	-1.931	0.053	0.144	0.000	5.504	3.965	2.364	0.000	0.000	0.000	0.702	0.000	0.000	2.037	0.662
ENSG00000282849	2.208	-5.252	2.799	-1.876	0.061	0.159	0.000	14.678	0.000	11.822	0.000	0.000	0.000	0.000	0.000	0.000	0.000	0.000
ENSG00000253746	1.002	-4.108	2.198	-1.869	0.062	0.161	0.000	2.752	0.991	8.276	0.000	0.000	0.000	0.000	0.000	0.000	0.000	0.000
ENSG00000267279	1.612	-2.906	1.555	-1.869	0.062	0.161	0.000	8.257	3.965	3.547	0.000	0.000	0.921	0.000	0.000	0.000	0.000	2.649
ENSG00000284883	0.755	-2.652	1.428	-1.856	0.063	0.165	0.000	2.752	1.983	2.364	0.000	1.038	0.921	0.000	0.000	0.000	0.000	0.000
ENSG00000236120	0.760	-3.048	1.680	-1.814	0.070	0.178	0.000	1.835	1.983	3.547	0.000	0.000	0.000	0.000	0.000	1.755	0.000	0.000
ENSG00000230309	0.705	-2.637	1.516	-1.739	0.082	0.203	0.000	3.670	0.991	2.364	0.730	0.000	0.000	0.702	0.000	0.000	0.000	0.000
ENSG00000227201	0.818	-2.366	1.370	-1.727	0.084	0.208	0.000	1.835	0.991	4.729	0.000	0.000	0.921	0.000	0.000	0.000	0.679	0.662
ENSG00000285215	0.697	-3.060	1.774	-1.725	0.085	0.208	0.000	0.917	2.974	3.547	0.000	0.000	0.921	0.000	0.000	0.000	0.000	0.000
ENSG00000229122	1.052	-2.052	1.203	-1.705	0.088	0.215	0.000	2.752	4.957	1.182	0.730	0.000	0.921	1.404	0.000	0.000	0.679	0.000
ENSG00000163017	3.480	-2.392	1.415	-1.690	0.091	0.221	0.000	13.761	0.000	16.551	0.730	2.077	2.763	4.213	1.661	0.000	0.000	0.000
ENSG00000249279	0.988	-3.163	1.881	-1.682	0.093	0.224	0.000	5.504	0.000	4.729	0.000	0.000	0.921	0.702	0.000	0.000	0.000	0.000
ENSG00000231683	1.064	-2.800	1.669	-1.678	0.093	0.225	0.000	0.000	7.930	2.364	0.730	1.038	0.000	0.702	0.000	0.000	0.000	0.000
ENSG00000284977	0.668	-2.985	1.779	-1.677	0.094	0.226	0.000	1.835	3.965	1.182	0.000	1.038	0.000	0.000	0.000	0.000	0.000	0.000
ENSG00000138152	0.696	-3.589	2.160	-1.662	0.097	0.231	0.000	1.835	2.974	3.547	0.000	0.000	0.000	0.000	0.000	0.000	0.000	0.000
ENSG00000230623	1.108	-3.819	2.299	-1.661	0.097	0.232	0.000	5.504	0.000	7.093	0.000	0.000	0.000	0.702	0.000	0.000	0.000	0.000
ENSG00000231123	0.654	-2.803	1.706	-1.643	0.100	0.239	0.000	1.835	1.983	2.364	0.000	0.000	0.000	0.000	1.661	0.000	0.000	0.000
ENSG00000180660	0.668	-3.535	2.181	-1.621	0.105	0.247	0.000	3.670	1.983	2.364	0.000	0.000	0.000	0.000	0.000	0.000	0.000	0.000
ENSG00000275759	1.418	-2.236	1.389	-1.610	0.107	0.252	0.000	0.000	8.922	2.364	0.730	0.000	0.921	0.000	1.661	1.755	0.000	0.662
ENSG00000120729	0.706	-3.607	2.246	-1.606	0.108	0.254	0.000	2.752	0.991	4.729	0.000	0.000	0.000	0.000	0.000	0.000	0.000	0.000
ENSG00000044524	1.175	-2.238	1.395	-1.605	0.109	0.254	0.000	4.587	0.991	4.729	2.190	0.000	0.921	0.000	0.000	0.000	0.679	0.000
ENSG00000280011	0.896	-3.027	1.889	-1.603	0.109	0.255	0.000	4.587	0.000	4.729	0.730	0.000	0.000	0.702	0.000	0.000	0.000	0.000
ENSG00000253668	0.592	-2.851	1.820	-1.567	0.117	0.270	0.000	0.917	1.983	3.547	0.000	0.000	0.000	0.000	0.000	0.000	0.000	0.662
ENSG00000248373	0.588	-2.837	1.817	-1.562	0.118	0.272	0.000	1.835	0.991	3.547	0.000	0.000	0.000	0.000	0.000	0.000	0.679	0.000
ENSG00000249267	0.582	-2.816	1.811	-1.555	0.120	0.275	0.000	0.917	2.974	2.364	0.730	0.000	0.000	0.000	0.000	0.000	0.000	0.000
ENSG00000257126	0.716	-3.639	2.352	-1.547	0.122	0.278	0.000	6.422	0.991	1.182	0.000	0.000	0.000	0.000	0.000	0.000	0.000	0.000
ENSG00000122254	0.662	-3.522	2.276	-1.547	0.122	0.278	0.000	4.587	0.991	2.364	0.000	0.000	0.000	0.000	0.000	0.000	0.000	0.000
ENSG00000267099	0.622	-2.384	1.582	-1.507	0.132	0.296	0.000	3.670	0.991	1.182	0.000	0.000	0.921	0.702	0.000	0.000	0.000	0.000
ENSG00000254418	0.757	-2.154	1.432	-1.505	0.132	0.297	0.000	0.917	0.991	4.729	0.730	1.038	0.000	0.000	0.000	0.000	0.679	0.000
ENSG00000205898	0.640	-2.207	1.521	-1.451	0.147	0.322	0.000	0.917	1.983	2.364	0.000	0.000	0.000	0.000	0.000	1.755	0.000	0.662
ENSG00000278715	1.278	-2.258	1.574	-1.434	0.152	0.330	0.000	1.835	5.948	3.547	0.000	0.000	0.000	0.000	0.000	0.000	1.358	2.649
ENSG00000110900	1.307	-2.041	1.437	-1.421	0.155	0.336	0.000	0.917	4.957	4.729	0.000	0.000	0.000	0.702	1.661	0.000	2.715	0.000
ENSG00000235182	0.683	-2.596	1.846	-1.406	0.160	0.343	0.000	3.670	1.983	1.182	0.000	0.000	0.000	0.000	0.000	0.000	1.358	0.000
ENSG00000259645	0.537	-3.211	2.344	-1.370	0.171	0.361	0.000	0.917	1.983	3.547	0.000	0.000	0.000	0.000	0.000	0.000	0.000	0.000
ENSG00000227660	0.569	-2.523	1.850	-1.364	0.173	0.364	0.000	0.917	2.974	1.182	0.000	0.000	0.000	0.000	0.000	1.755	0.000	0.000
ENSG00000222112	0.695	-2.575	1.889	-1.363	0.173	0.364	0.000	2.752	3.965	0.000	0.000	0.000	0.921	0.702	0.000	0.000	0.000	0.000
ENSG00000275426	0.542	-2.169	1.596	-1.359	0.174	0.366	0.000	0.917	2.974	1.182	0.730	0.000	0.000	0.702	0.000	0.000	0.000	0.000
ENSG00000280744	0.548	-2.139	1.584	-1.350	0.177	0.371	0.000	2.752	0.991	1.182	0.730	0.000	0.921	0.000	0.000	0.000	0.000	0.000
ENSG00000275646	0.544	-2.139	1.585	-1.349	0.177	0.372	0.000	2.752	0.991	1.182	0.000	0.000	0.921	0.000	0.000	0.000	0.679	0.000
ENSG00000251330	0.725	-2.093	1.562	-1.339	0.180	0.377	0.000	0.917	2.974	2.364	0.000	1.038	0.000	1.404	0.000	0.000	0.000	0.000
ENSG00000271290	0.825	-2.036	1.526	-1.335	0.182	0.379	0.000	1.835	2.974	2.364	0.000	0.000	0.000	1.404	0.000	0.000	0.000	1.324

List of genes downregulated in Normal-like vs Luminal-A cell-lines

Gene	baseMean	log2FoldChange	lfcSE	stat	pvalue	padj	MCF10A.RNA.Seq.Rep1_sorted	MCF10A.RNA.Seq.Rep2_sorted	MCF10A.RNA.Seq.Rep3_sorted	MCF10A.RNA.Seq.Rep4_sorted	MCF7.RNA.Seq.Rep1_sorted	MCF7.RNA.Seq.Rep2_sorted	MCF7.RNA.Seq.Rep3_sorted	MCF7.RNA.Seq.Rep4_sorted	ZR751.RNA.Seq.Rep1_sorted	ZR751.RNA.Seq.Rep2_sorted	ZR751.RNA.Seq.Rep3_sorted	ZR751.RNA.Seq.Rep4_sorted
ENSG00000254510	0.995	-4.100	3.084	-1.330	0.184	0.382	0.000	3.670	0.000	8.276	0.000	0.000	0.000	0.000	0.000	0.000	0.000	0.000
ENSG00000200742	0.497	-2.488	1.879	-1.324	0.186	0.385	0.000	2.752	0.991	1.182	0.000	1.038	0.000	0.000	0.000	0.000	0.000	0.000
ENSG00000204577	0.493	-3.101	2.345	-1.322	0.186	0.386	0.000	2.752	1.983	1.182	0.000	0.000	0.000	0.000	0.000	0.000	0.000	0.000
ENSG00000267138	0.870	-2.158	1.638	-1.318	0.188	0.388	0.000	2.752	4.957	0.000	0.730	0.000	0.000	0.000	0.000	0.000	0.679	1.324
ENSG00000224884	0.467	-2.487	1.899	-1.310	0.190	0.392	0.000	2.752	0.991	1.182	0.000	0.000	0.000	0.000	0.000	0.000	0.679	0.000
ENSG00000272243	0.505	-3.132	2.423	-1.293	0.196	0.402	0.000	0.917	3.965	1.182	0.000	0.000	0.000	0.000	0.000	0.000	0.000	0.000
ENSG00000221461	0.670	-2.461	1.911	-1.288	0.198	0.404	0.000	2.752	0.000	3.547	0.000	1.038	0.000	0.702	0.000	0.000	0.000	0.000
ENSG00000228877	0.682	-3.066	2.408	-1.273	0.203	0.412	0.000	2.752	0.000	4.729	0.000	0.000	0.000	0.702	0.000	0.000	0.000	0.000
ENSG00000267019	0.487	-3.084	2.425	-1.272	0.203	0.413	0.000	3.670	0.991	1.182	0.000	0.000	0.000	0.000	0.000	0.000	0.000	0.000
ENSG00000230080	0.851	-2.073	1.638	-1.265	0.206	0.417	0.000	0.917	2.974	3.547	0.000	0.000	0.000	2.106	0.000	0.000	0.000	0.662
ENSG00000135960	0.614	-2.911	2.405	-1.210	0.226	0.447	0.000	3.670	2.974	0.000	0.730	0.000	0.000	0.000	0.000	0.000	0.000	0.000
ENSG00000136872	0.621	-2.939	2.438	-1.206	0.228	0.450	0.000	1.835	4.957	0.000	0.000	0.000	0.000	0.000	0.000	0.000	0.000	0.662
ENSG00000175746	0.417	-2.857	2.414	-1.184	0.237	0.462	0.000	1.835	1.983	1.182	0.000	0.000	0.000	0.000	0.000	0.000	0.000	0.000
ENSG00000266289	0.479	-2.210	1.877	-1.178	0.239	0.466	0.000	0.917	1.983	1.182	0.000	0.000	0.000	0.000	1.661	0.000	0.000	0.000
ENSG00000280655	0.433	-2.259	1.925	-1.174	0.241	0.468	0.000	0.917	0.991	2.364	0.000	0.000	0.921	0.000	0.000	0.000	0.000	0.000
ENSG00000270356	0.433	-2.259	1.925	-1.174	0.241	0.468	0.000	0.917	0.991	2.364	0.000	0.000	0.921	0.000	0.000	0.000	0.000	0.000
ENSG00000284387	0.480	-2.190	1.869	-1.172	0.241	0.469	0.000	1.835	0.991	1.182	0.000	0.000	0.000	0.000	0.000	1.755	0.000	0.000
ENSG00000047648	0.472	-2.190	1.874	-1.169	0.243	0.471	0.000	1.835	0.991	1.182	0.000	0.000	0.000	0.000	1.661	0.000	0.000	0.000
ENSG00000207617	0.759	-3.714	3.183	-1.167	0.243	0.472	0.000	0.000	7.930	1.182	0.000	0.000	0.000	0.000	0.000	0.000	0.000	0.000
ENSG00000177994	0.423	-2.876	2.471	-1.164	0.245	0.473	0.000	0.917	2.974	1.182	0.000	0.000	0.000	0.000	0.000	0.000	0.000	0.000
ENSG00000225315	0.421	-2.189	1.911	-1.146	0.252	0.484	0.000	1.835	0.991	1.182	0.000	1.038	0.000	0.000	0.000	0.000	0.000	0.000
ENSG00000227868	0.628	-2.730	2.481	-1.100	0.271	0.511	0.000	0.917	4.957	0.000	0.000	0.000	0.000	0.000	1.661	0.000	0.000	0.000
ENSG00000201183	0.570	-2.787	2.537	-1.099	0.272	0.512	0.000	0.000	4.957	1.182	0.000	0.000	0.000	0.702	0.000	0.000	0.000	0.000
ENSG00000257042	0.645	-3.474	3.190	-1.089	0.276	0.517	0.000	1.835	0.000	5.911	0.000	0.000	0.000	0.000	0.000	0.000	0.000	0.000
ENSG00000238390	0.538	-2.625	2.481	-1.058	0.290	0.537	0.000	0.000	1.983	3.547	0.000	0.000	0.921	0.000	0.000	0.000	0.000	0.000
ENSG00000178033	0.601	-3.376	3.194	-1.057	0.290	0.538	0.000	3.670	0.000	3.547	0.000	0.000	0.000	0.000	0.000	0.000	0.000	0.000
ENSG00000284459	0.594	-3.361	3.194	-1.052	0.293	0.541	0.000	0.000	5.948	1.182	0.000	0.000	0.000	0.000	0.000	0.000	0.000	0.000
ENSG00000050730	0.791	-2.070	1.971	-1.050	0.294	0.542	0.000	1.835	0.991	3.547	0.000	3.115	0.000	0.000	0.000	0.000	0.000	0.000
ENSG00000232310	0.525	-2.589	2.481	-1.044	0.297	0.546	0.000	1.835	0.000	3.547	0.000	0.000	0.921	0.000	0.000	0.000	0.000	0.000
ENSG00000213489	0.535	-2.670	2.563	-1.042	0.297	0.547	0.000	0.000	0.991	4.729	0.000	0.000	0.000	0.702	0.000	0.000	0.000	0.000
ENSG00000271507	0.509	-2.589	2.495	-1.038	0.299	0.549	0.000	1.835	0.000	3.547	0.730	0.000	0.000	0.000	0.000	0.000	0.000	0.000
ENSG00000272121	0.356	-2.619	2.543	-1.030	0.303	0.554	0.000	0.917	0.991	2.364	0.000	0.000	0.000	0.000	0.000	0.000	0.000	0.000
ENSG00000257925	0.569	-3.290	3.197	-1.029	0.303	0.555	0.000	0.917	0.000	5.911	0.000	0.000	0.000	0.000	0.000	0.000	0.000	0.000
ENSG00000278884	0.569	-3.290	3.197	-1.029	0.303	0.555	0.000	0.917	0.000	5.911	0.000	0.000	0.000	0.000	0.000	0.000	0.000	0.000
ENSG00000276012	0.554	-3.264	3.198	-1.021	0.307	0.560	0.000	3.670	2.974	0.000	0.000	0.000	0.000	0.000	0.000	0.000	0.000	0.000
ENSG00000134365	0.543	-3.227	3.199	-1.009	0.313	0.567	0.000	0.000	2.974	3.547	0.000	0.000	0.000	0.000	0.000	0.000	0.000	0.000
ENSG00000241679	0.340	-2.562	2.549	-1.005	0.315	0.569	0.000	0.917	1.983	1.182	0.000	0.000	0.000	0.000	0.000	0.000	0.000	0.000
ENSG00000279288	0.340	-2.562	2.549	-1.005	0.315	0.569	0.000	0.917	1.983	1.182	0.000	0.000	0.000	0.000	0.000	0.000	0.000	0.000
ENSG00000272689	0.856	-2.013	2.014	-1.000	0.318	0.573	0.000	3.670	0.000	2.364	0.730	0.000	0.000	0.000	0.000	3.509	0.000	0.000
ENSG00000185031	0.334	-2.539	2.551	-0.995	0.320	0.576	0.000	1.835	0.991	1.182	0.000	0.000	0.000	0.000	0.000	0.000	0.000	0.000
ENSG00000226012	0.334	-2.539	2.551	-0.995	0.320	0.576	0.000	1.835	0.991	1.182	0.000	0.000	0.000	0.000	0.000	0.000	0.000	0.000
ENSG00000170683	0.525	-3.179	3.201	-0.993	0.321	0.577	0.000	2.752	0.000	3.547	0.000	0.000	0.000	0.000	0.000	0.000	0.000	0.000
ENSG00000267685	0.503	-3.121	3.203	-0.974	0.330	0.590	0.000	3.670	0.000	2.364	0.000	0.000	0.000	0.000	0.000	0.000	0.000	0.000
ENSG00000251388	0.496	-3.102	3.204	-0.968	0.333	0.593	0.000	0.000	5.948	0.000	0.000	0.000	0.000	0.000	0.000	0.000	0.000	0.000
ENSG00000254871	0.484	-2.466	2.553	-0.966	0.334	0.595	0.000	0.917	3.965	0.000	0.000	0.000	0.921	0.000	0.000	0.000	0.000	0.000
ENSG00000252034	0.501	-2.281	2.475	-0.921	0.357	0.622	0.000	0.000	1.983	2.364	0.000	0.000	0.000	0.000	1.661	0.000	0.000	0.000
ENSG00000235419	0.448	-2.950	3.210	-0.919	0.358	0.623	0.000	1.835	0.000	3.547	0.000	0.000	0.000	0.000	0.000	0.000	0.000	0.000
ENSG00000205358	0.518	-2.314	2.521	-0.918	0.359	0.624	0.000	0.917	0.000	3.547	0.000	0.000	0.000	0.000	0.000	1.755	0.000	0.000
ENSG00000224167	0.455	-2.336	2.586	-0.903	0.366	0.634	0.000	0.000	0.991	3.547	0.000	0.000	0.921	0.000	0.000	0.000	0.000	0.000
ENSG00000227941	0.426	-2.882	3.214	-0.897	0.370	0.637	0.000	2.752	0.000	2.364	0.000	0.000	0.000	0.000	0.000	0.000	0.000	0.000
ENSG00000237819	0.426	-2.882	3.214	-0.897	0.370	0.637	0.000	2.752	0.000	2.364	0.000	0.000	0.000	0.000	0.000	0.000	0.000	0.000
ENSG00000127529	0.413	-2.841	3.216	-0.883	0.377	0.646	0.000	0.000	4.957	0.000	0.000	0.000	0.000	0.000	0.000	0.000	0.000	0.000
ENSG00000232492	0.485	-2.225	2.528	-0.880	0.379	0.648	0.000	0.000	2.974	1.182	0.000	0.000	0.000	0.000	1.661	0.000	0.000	0.000
ENSG00000158022	0.411	-2.236	2.556	-0.875	0.382	0.652	0.000	1.835	0.000	2.364	0.730	0.000	0.000	0.000	0.000	0.000	0.000	0.000
ENSG00000151418	0.404	-2.811	3.217	-0.874	0.382	0.652	0.000	3.670	0.000	1.182	0.000	0.000	0.000	0.000	0.000	0.000	0.000	0.000
ENSG00000105664	0.404	-2.811	3.217	-0.874	0.382	0.652	0.000	3.670	0.000	1.182	0.000	0.000	0.000	0.000	0.000	0.000	0.000	0.000
ENSG00000268669	0.404	-2.811	3.217	-0.874	0.382	0.652	0.000	3.670	0.000	1.182	0.000	0.000	0.000	0.000	0.000	0.000	0.000	0.000
ENSG00000271334	0.395	-2.779	3.219	-0.863	0.388	0.659	0.000	2.752	1.983	0.000	0.000	0.000	0.000	0.000	0.000	0.000	0.000	0.000
ENSG00000198914	0.388	-2.758	3.220	-0.857	0.392	0.663	0.000	3.670	0.991	0.000	0.000	0.000	0.000	0.000	0.000	0.000	0.000	0.000
ENSG00000221858	0.582	-2.742	3.204	-0.856	0.392	0.664	0.000	0.000	5.948	0.000	0.000	1.038	0.000	0.000	0.000	0.000	0.000	0.000
ENSG00000278134	0.403	-2.224	2.614	-0.851	0.395	0.667	0.000	0.000	2.974	1.182	0.000	0.000	0.000	0.000	0.000	0.000	0.679	0.000
ENSG00000228784	0.395	-2.119	2.542	-0.833	0.405	0.678	0.000	1.835	1.983	0.000	0.000	0.000	0.921	0.000	0.000	0.000	0.000	0.000
ENSG00000276136	0.458	-2.095	2.518	-0.832	0.405	0.678	0.000	2.752	0.991	0.000	0.000	0.000	0.000	0.000	0.000	1.755	0.000	0.000
ENSG00000254717	0.372	-2.677	3.224	-0.830	0.406	0.680	0.000	0.917	0.000	3.547	0.000	0.000	0.000	0.000	0.000	0.000	0.000	0.000
ENSG00000081237	0.375	-2.119	2.568	-0.825	0.409	0.683	0.000	1.835	1.983	0.000	0.000	0.000	0.000	0.000	0.000	0.000	0.679	0.000
ENSG00000250362	0.375	-2.119	2.568	-0.825	0.409	0.683	0.000	1.835	1.983	0.000	0.000	0.000	0.000	0.000	0.000	0.000	0.679	0.000
ENSG00000271584	0.362	-2.642	3.226	-0.819	0.413	0.687	0.000	0.000	1.983	2.364	0.000	0.000	0.000	0.000	0.000	0.000	0.000	0.000
ENSG00000228755	0.381	-2.142	2.620	-0.818	0.414	0.688	0.000	0.917	2.974	0.000	0.000	0.000	0.000	0.000	0.000	0.000	0.679	0.000
ENSG00000279951	0.381	-2.142	2.620	-0.818	0.414	0.688	0.000	0.917	2.974	0.000	0.000	0.000	0.000	0.000	0.000	0.000	0.679	0.000
ENSG00000196979	0.350	-2.595	3.229	-0.804	0.422	0.697	0.000	1.835	0.000	2.364	0.000	0.000	0.000	0.000	0.000	0.000	0.000	0.000
ENSG00000228553	0.350	-2.595	3.229	-0.804	0.422	0.697	0.000	1.835	0.000	2.364	0.000	0.000	0.000	0.000	0.000	0.000	0.000	0.000
ENSG00000207864	0.346	-2.582	3.229	-0.800	0.424	0.700	0.000	0.000	2.974	1.182	0.000	0.000	0.000	0.000	0.000	0.000	0.000	0.000
ENSG00000215127	0.258	-2.160	2.703	-0.799	0.424	0.700	0.000	0.917	0.991	1.182	0.000	0.000	0.000	0.000	0.000	0.000	0.000	0.000
ENSG00000250331	0.258	-2.160	2.703	-0.799	0.424	0.700	0.000	0.917	0.991	1.182	0.000	0.000	0.000	0.000	0.000	0.000	0.000	0.000
ENSG00000227681	0.258	-2.160	2.703	-0.799	0.424	0.700	0.000	0.917	0.991	1.182	0.000	0.000	0.000	0.000	0.000	0.000	0.000	0.000
ENSG00000279810	0.258	-2.160	2.703	-0.799	0.424	0.700	0.000	0.917	0.991	1.182	0.000	0.000	0.000	0.000	0.000	0.000	0.000	0.000
ENSG00000252509	0.258	-2.160	2.703	-0.799	0.424	0.700	0.000	0.917	0.991	1.182	0.000	0.000	0.000	0.000	0.000	0.000	0.000	0.000
ENSG00000212421	0.258	-2.160	2.703	-0.799	0.424	0.700	0.000	0.917	0.991	1.182	0.000	0.000	0.000	0.000	0.000	0.000	0.000	0.000
ENSG00000255227	0.258	-2.160	2.703	-0.799	0.424	0.700	0.000	0.917	0.991	1.182	0.000	0.000	0.000	0.000	0.000	0.000	0.000	0.000
ENSG00000278520	0.258	-2.160	2.703	-0.799	0.424	0.700	0.000	0.917	0.991	1.182	0.000	0.000	0.000	0.000	0.000	0.000	0.000	0.000
ENSG00000257859	0.258	-2.160	2.703	-0.799	0.424	0.700	0.000	0.917	0.991	1.182	0.000	0.000	0.000	0.000	0.000	0.000	0.000	0.000
ENSG00000230839	0.258	-2.160	2.703	-0.799	0.424	0.700	0.000	0.917	0.991	1.182	0.000	0.000	0.000	0.000	0.000	0.000	0.000	0.000
ENSG00000277173	0.330	-2.521	3.233	-0.780	0.436	0.713	0.000	0.000	3.965	0.000	0.000	0.000	0.000	0.000	0.000	0.000	0.000	0.000
ENSG00000283990	0.330	-2.521	3.233	-0.780	0.436	0.713	0.000	0.000	3.965	0.000	0.000	0.000	0.000	0.000	0.000	0.000	0.000	0.000
ENSG00000225166	0.328	-2.509	3.234	-0.776	0.438	0.716	0.000	2.752	0.000	1.182	0.000	0.000	0.000	0.000	0.000	0.000	0.000	0.000
ENSG00000273464	0.328	-2.509	3.234	-0.776	0.438	0.716	0.000	2.752	0.000	1.182	0.000	0.000	0.000	0.000	0.000	0.000	0.000	0.000
ENSG00000274663	0.324	-2.496	3.235	-0.772	0.440	0.719	0.000	0.917	2.974	0.000	0.000	0.000	0.000	0.000	0.000	0.000	0.000	0.000
ENSG00000214511	0.318	-2.470	3.236	-0.763	0.445	0.724	0.000	1.835	1.983	0.000	0.000	0.000	0.000	0.000	0.000	0.000	0.000	0.000
ENSG00000226520	0.312	-2.445	3.238	-0.755	0.450	0.729	0.000	2.752	0.991	0.000	0.000	0.000	0.000	0.000	0.000	0.000	0.000	0.000
ENSG00000177938	0.312	-2.445	3.238	-0.755	0.450	0.729	0.000	2.752	0.991	0.000	0.000	0.000	0.000	0.000	0.000	0.000	0.000	0.000

List of genes downregulated in Normal-like vs Luminal-A cell-lines

Gene	baseMean	log2FoldChange	lfcSE	stat	pvalue	padj	MCF10A.RNA.Seq.Rep1_sorted	MCF10A.RNA.Seq.Rep2_sorted	MCF10A.RNA.Seq.Rep3_sorted	MCF10A.RNA.Seq.Rep4_sorted	MCF7.RNA.Seq.Rep1_sorted	MCF7.RNA.Seq.Rep2_sorted	MCF7.RNA.Seq.Rep3_sorted	MCF7.RNA.Seq.Rep4_sorted	ZR751.RNA.Seq.Rep1_sorted	ZR751.RNA.Seq.Rep2_sorted	ZR751.RNA.Seq.Rep3_sorted	ZR751.RNA.Seq.Rep4_sorted
ENSG00000167910	0.306	-2.418	3.240	-0.747	0.455	0.734	0.000	3.670	0.000	0.000	0.000	0.000	0.000	0.000	0.000	0.000	0.000	0.000
ENSG00000238288	0.296	-2.337	3.245	-0.720	0.471	0.750	0.000	0.000	0.000	3.547	0.000	0.000	0.000	0.000	0.000	0.000	0.000	0.000
ENSG00000240733	0.280	-2.264	3.250	-0.697	0.486	0.765	0.000	0.000	0.991	2.364	0.000	0.000	0.000	0.000	0.000	0.000	0.000	0.000
ENSG00000150783	0.280	-2.264	3.250	-0.697	0.486	0.765	0.000	0.000	0.991	2.364	0.000	0.000	0.000	0.000	0.000	0.000	0.000	0.000
ENSG00000260613	0.280	-2.264	3.250	-0.697	0.486	0.765	0.000	0.000	0.991	2.364	0.000	0.000	0.000	0.000	0.000	0.000	0.000	0.000
ENSG00000232912	0.273	-2.234	3.252	-0.687	0.492	0.772	0.000	0.917	0.000	2.364	0.000	0.000	0.000	0.000	0.000	0.000	0.000	0.000
ENSG00000233072	0.273	-2.234	3.252	-0.687	0.492	0.772	0.000	0.917	0.000	2.364	0.000	0.000	0.000	0.000	0.000	0.000	0.000	0.000
ENSG00000228939	0.273	-2.234	3.252	-0.687	0.492	0.772	0.000	0.917	0.000	2.364	0.000	0.000	0.000	0.000	0.000	0.000	0.000	0.000
ENSG00000260635	0.273	-2.234	3.252	-0.687	0.492	0.772	0.000	0.917	0.000	2.364	0.000	0.000	0.000	0.000	0.000	0.000	0.000	0.000
ENSG00000264452	0.273	-2.234	3.252	-0.687	0.492	0.772	0.000	0.917	0.000	2.364	0.000	0.000	0.000	0.000	0.000	0.000	0.000	0.000
ENSG00000231063	0.264	-2.188	3.256	-0.672	0.502	0.780	0.000	0.000	1.983	1.182	0.000	0.000	0.000	0.000	0.000	0.000	0.000	0.000
ENSG00000251189	0.264	-2.188	3.256	-0.672	0.502	0.780	0.000	0.000	1.983	1.182	0.000	0.000	0.000	0.000	0.000	0.000	0.000	0.000
ENSG00000139329	0.264	-2.188	3.256	-0.672	0.502	0.780	0.000	0.000	1.983	1.182	0.000	0.000	0.000	0.000	0.000	0.000	0.000	0.000
ENSG00000125788	0.264	-2.188	3.256	-0.672	0.502	0.780	0.000	0.000	1.983	1.182	0.000	0.000	0.000	0.000	0.000	0.000	0.000	0.000
ENSG00000276017	0.264	-2.188	3.256	-0.672	0.502	0.780	0.000	0.000	1.983	1.182	0.000	0.000	0.000	0.000	0.000	0.000	0.000	0.000
ENSG00000263361	0.389	-2.160	3.233	-0.668	0.504	0.783	0.000	0.000	3.965	0.000	0.000	0.000	0.000	0.702	0.000	0.000	0.000	0.000
ENSG00000256083	0.407	-2.160	3.233	-0.668	0.504	0.783	0.000	0.000	3.965	0.000	0.000	0.000	0.921	0.000	0.000	0.000	0.000	0.000
ENSG00000176378	0.251	-2.125	3.261	-0.652	0.515	0.793	0.000	1.835	0.000	1.182	0.000	0.000	0.000	0.000	0.000	0.000	0.000	0.000
ENSG00000202078	0.251	-2.125	3.261	-0.652	0.515	0.793	0.000	1.835	0.000	1.182	0.000	0.000	0.000	0.000	0.000	0.000	0.000	0.000
ENSG00000213045	0.251	-2.125	3.261	-0.652	0.515	0.793	0.000	1.835	0.000	1.182	0.000	0.000	0.000	0.000	0.000	0.000	0.000	0.000
ENSG00000143768	0.251	-2.125	3.261	-0.652	0.515	0.793	0.000	1.835	0.000	1.182	0.000	0.000	0.000	0.000	0.000	0.000	0.000	0.000
ENSG00000223965	0.251	-2.125	3.261	-0.652	0.515	0.793	0.000	1.835	0.000	1.182	0.000	0.000	0.000	0.000	0.000	0.000	0.000	0.000
ENSG00000265657	0.251	-2.125	3.261	-0.652	0.515	0.793	0.000	1.835	0.000	1.182	0.000	0.000	0.000	0.000	0.000	0.000	0.000	0.000
ENSG00000235205	0.251	-2.125	3.261	-0.652	0.515	0.793	0.000	1.835	0.000	1.182	0.000	0.000	0.000	0.000	0.000	0.000	0.000	0.000
ENSG00000266466	0.251	-2.125	3.261	-0.652	0.515	0.793	0.000	1.835	0.000	1.182	0.000	0.000	0.000	0.000	0.000	0.000	0.000	0.000
ENSG00000213417	0.251	-2.125	3.261	-0.652	0.515	0.793	0.000	1.835	0.000	1.182	0.000	0.000	0.000	0.000	0.000	0.000	0.000	0.000
ENSG00000278816	0.251	-2.125	3.261	-0.652	0.515	0.793	0.000	1.835	0.000	1.182	0.000	0.000	0.000	0.000	0.000	0.000	0.000	0.000
ENSG00000136546	0.248	-2.109	3.262	-0.646	0.518	0.796	0.000	0.000	2.974	0.000	0.000	0.000	0.000	0.000	0.000	0.000	0.000	0.000
ENSG00000272922	0.248	-2.109	3.262	-0.646	0.518	0.796	0.000	0.000	2.974	0.000	0.000	0.000	0.000	0.000	0.000	0.000	0.000	0.000
ENSG00000241868	0.248	-2.109	3.262	-0.646	0.518	0.796	0.000	0.000	2.974	0.000	0.000	0.000	0.000	0.000	0.000	0.000	0.000	0.000
ENSG00000248692	0.248	-2.109	3.262	-0.646	0.518	0.796	0.000	0.000	2.974	0.000	0.000	0.000	0.000	0.000	0.000	0.000	0.000	0.000
ENSG00000277402	0.248	-2.109	3.262	-0.646	0.518	0.796	0.000	0.000	2.974	0.000	0.000	0.000	0.000	0.000	0.000	0.000	0.000	0.000
ENSG00000274988	0.248	-2.109	3.262	-0.646	0.518	0.796	0.000	0.000	2.974	0.000	0.000	0.000	0.000	0.000	0.000	0.000	0.000	0.000
ENSG00000276266	0.248	-2.109	3.262	-0.646	0.518	0.796	0.000	0.000	2.974	0.000	0.000	0.000	0.000	0.000	0.000	0.000	0.000	0.000
ENSG00000270105	0.248	-2.109	3.262	-0.646	0.518	0.796	0.000	0.000	2.974	0.000	0.000	0.000	0.000	0.000	0.000	0.000	0.000	0.000
ENSG00000230300	0.248	-2.109	3.262	-0.646	0.518	0.796	0.000	0.000	2.974	0.000	0.000	0.000	0.000	0.000	0.000	0.000	0.000	0.000
ENSG00000258420	0.248	-2.109	3.262	-0.646	0.518	0.796	0.000	0.000	2.974	0.000	0.000	0.000	0.000	0.000	0.000	0.000	0.000	0.000
ENSG00000258415	0.248	-2.109	3.262	-0.646	0.518	0.796	0.000	0.000	2.974	0.000	0.000	0.000	0.000	0.000	0.000	0.000	0.000	0.000
ENSG00000225638	0.242	-2.076	3.265	-0.636	0.525	0.803	0.000	0.917	1.983	0.000	0.000	0.000	0.000	0.000	0.000	0.000	0.000	0.000
ENSG00000276573	0.242	-2.076	3.265	-0.636	0.525	0.803	0.000	0.917	1.983	0.000	0.000	0.000	0.000	0.000	0.000	0.000	0.000	0.000
ENSG00000258122	0.242	-2.076	3.265	-0.636	0.525	0.803	0.000	0.917	1.983	0.000	0.000	0.000	0.000	0.000	0.000	0.000	0.000	0.000
ENSG00000266441	0.242	-2.076	3.265	-0.636	0.525	0.803	0.000	0.917	1.983	0.000	0.000	0.000	0.000	0.000	0.000	0.000	0.000	0.000
ENSG00000252412	0.242	-2.076	3.265	-0.636	0.525	0.803	0.000	0.917	1.983	0.000	0.000	0.000	0.000	0.000	0.000	0.000	0.000	0.000
ENSG00000242578	0.236	-2.043	3.267	-0.625	0.532	0.810	0.000	1.835	0.991	0.000	0.000	0.000	0.000	0.000	0.000	0.000	0.000	0.000
ENSG00000175538	0.236	-2.043	3.267	-0.625	0.532	0.810	0.000	1.835	0.991	0.000	0.000	0.000	0.000	0.000	0.000	0.000	0.000	0.000
ENSG00000254563	0.236	-2.043	3.267	-0.625	0.532	0.810	0.000	1.835	0.991	0.000	0.000	0.000	0.000	0.000	0.000	0.000	0.000	0.000
ENSG00000265917	0.236	-2.043	3.267	-0.625	0.532	0.810	0.000	1.835	0.991	0.000	0.000	0.000	0.000	0.000	0.000	0.000	0.000	0.000
ENSG00000229266	0.236	-2.043	3.267	-0.625	0.532	0.810	0.000	1.835	0.991	0.000	0.000	0.000	0.000	0.000	0.000	0.000	0.000	0.000
ENSG00000162267	0.229	-2.009	3.270	-0.614	0.539	0.817	0.000	2.752	0.000	0.000	0.000	0.000	0.000	0.000	0.000	0.000	0.000	0.000
ENSG00000226673	0.229	-2.009	3.270	-0.614	0.539	0.817	0.000	2.752	0.000	0.000	0.000	0.000	0.000	0.000	0.000	0.000	0.000	0.000
ENSG00000222004	0.229	-2.009	3.270	-0.614	0.539	0.817	0.000	2.752	0.000	0.000	0.000	0.000	0.000	0.000	0.000	0.000	0.000	0.000
ENSG00000173077	0.229	-2.009	3.270	-0.614	0.539	0.817	0.000	2.752	0.000	0.000	0.000	0.000	0.000	0.000	0.000	0.000	0.000	0.000
ENSG00000255008	0.229	-2.009	3.270	-0.614	0.539	0.817	0.000	2.752	0.000	0.000	0.000	0.000	0.000	0.000	0.000	0.000	0.000	0.000
ENSG00000123243	0.229	-2.009	3.270	-0.614	0.539	0.817	0.000	2.752	0.000	0.000	0.000	0.000	0.000	0.000	0.000	0.000	0.000	0.000
ENSG00000212725	0.229	-2.009	3.270	-0.614	0.539	0.817	0.000	2.752	0.000	0.000	0.000	0.000	0.000	0.000	0.000	0.000	0.000	0.000
ENSG00000264880	0.229	-2.009	3.270	-0.614	0.539	0.817	0.000	2.752	0.000	0.000	0.000	0.000	0.000	0.000	0.000	0.000	0.000	0.000
ENSG00000203971	0.229	-2.009	3.270	-0.614	0.539	0.817	0.000	2.752	0.000	0.000	0.000	0.000	0.000	0.000	0.000	0.000	0.000	0.000
