# Supplementary material for: Analysis of H3K4me3-ChIP-Seq and RNA-Seq data to understand the putative role of miRNAs and their target genes in breast cancer cell lines
Source: Genomics Inform. 2021 Jun 30;19(2):e17. doi: 10.5808/gi.21020 (PMC8261273; doi:10.5808/gi.21020)
Supplement: Supplementary Table 12. — List of genes upregulated in MCF10A vs. triple-negative breast cancer cell lines [file gi-21020suppl12.docx]

**Supplementary Table 12.** List of genes upregulated in MCF10A vs. triple-negative breast cancer cell lines

| List of genes upregulated in MCF10A vs TNBC subtype cell lines | | | | | | pvalue  0.0500485897  0.05008535966  0.05020787021  0.05026225155  0.05038980085  0.05049698244 | padj  0.1113563418  0.1113996584  0.1116142287  0.1116935924  0.1119044173  0.1120808896 | MCF10A.RNA.S  0.9967698847  0  0  0  0  0 | MCF10A.RNA.S  0  0.7173025769  0  0  0  0 | MCF10A.RNA.S  0  0  0.780045262  0  0  0 | MCF10A.RNA.S  0  0  0  0  0  0 | MB231.RNA.Seq  1.937960227  1.937960227  3.875920454  0  0  3.875920454 | MB231.RNA.Seq  0.9729249532  0.9729249532  0  0  0.9729249532  1.945849906 | MB231.RNA.Seq  0.7489427904  2.246828371  2.246828371  0.7489427904  0.7489427904  0.7489427904 | MB231.RNA.Seq  0.615839528  3.695037168  0  0  0  0.615839528 | MB436.RNA.Seq  8.365588755  0  2.788529585  1.394264792  4.182794377  0 | MB436.RNA.Seq  1.390846572  0  2.781693144  2.781693144  1.390846572  2.781693144 | MB436.RNA.Seq  0  4.14083481  0  8.281669619  4.14083481  0 |  |
| --- | --- | --- | --- | --- | --- | --- | --- | --- | --- | --- | --- | --- | --- | --- | --- | --- | --- | --- | --- |
| 12722  12727  12733  12738  12746  12752 | Gene ENSG00000203321 ENSG00000258591  ENSG00000254328 ENSG00000255363 ENSG00000235887 ENSG00000226416 | baseMean  1.332486279  1.262694338  1.399779056  1.500948626  1.113189064  0.8707272617 | log2FoldChange  2.85116178  2.749542558  3.03809391  3.682280424  3.20985835  3.140380609 | lfcSE  1.455009574  1.403376118  1.551481659  1.880897372  1.640495053  1.605733304 | stat  1.959548467  1.959234251  1.958188737  1.957725328  1.956640067  1.955729884 |  |  |  |  |  |  |  |  |  |  |  |  |  | MB436.RNA.Seq |
|  |  |  |  |  |  |  |  |  |  |  |  |  |  |  |  |  |  |  | 0.9609626337  1.441443951  4.324331852  4.804813169  1.921925267  0.4804813169 |
| 12763 ENSG00000223417 1.076751814 3.188382542 1.632258461 1.953356418 0.05077737661 0.1126137414 0 0 0 0 0.9689801135 0 0.7489427904 0 2.788529585 1.390846572 4.14083481 2.882887901 | | | | | | | | | | | | | | | | | | | |
| 12778  12781  12783  12784  12794  12798  12800  12804  12821 | ENSG00000179299 ENSG00000155754 ENSG00000169618 ENSG00000105219 ENSG00000211751 ENSG00000147257 ENSG00000112320 ENSG00000165617 ENSG00000269938 | 9.289736866  10.0435646  2.579847489  2.708032959  1.958036444  3.530391502  13.79509486  1.391903269  2.146041593 | 2.213107525  2.459042905  3.146793178  3.25498955  3.51586756  3.537613377  2.458833483  3.690014251  2.937083946 | 1.13417607  1.260351353  1.613049176  1.668773596  1.803461915  1.815402555  1.261856644  1.893923224  1.510628167 | 1.951290971  1.951077293  1.950835241  1.950527955  1.949510289  1.948666078  1.948583854  1.948344159  1.944279876 | 0.0510224425  0.05104785194  0.05107664815  0.05111322479  0.05123451529  0.05133531535  0.05134514189  0.05137379665  0.05186171003 | 0.1130079231  0.1130553554  0.1131014318  0.1131735717  0.1133662176  0.1135332821  0.1135449677  0.1135772777  0.1144877713 | 1.993539769  3.987079539  0  0  0  0  4.983849424  0  0 | 2.151907731  1.434605154  1.434605154  0.7173025769  0  0.7173025769  2.151907731  0  1.434605154 | 2.340135786  3.90022631  0  0  0  0.780045262  4.680271572  0  0 | 4.635928197  0.9271856394  0  0.9271856394  0.9271856394  0  1.854371279  0  0 | 30.03838352  15.50368182  0.9689801135  13.56572159  7.751840908  0  0  0  0.9689801135 | 13.62094935  30.16067355  0.9729249532  0.9729249532  4.864624766  0  0.9729249532  0  2.91877486 | 34.45136836  24.71511208  0  13.48097023  7.489427904  0  0.7489427904  0.7489427904  0.7489427904 | 20.32270442  39.41372979  0  0  2.463358112  0  0  0  1.847518584 | 0  0  9.759853547  0  0  11.15411834  39.03941419  1.394264792  12.54838313 | 0  0  6.95423286  1.390846572  0  8.345079432  30.59862459  4.172539716  0 | 0  0  4.14083481  0  0  16.56333924  37.26751329  4.14083481  0 | 1.921925267  0.4804813169  6.726738436  1.441443951  0  4.804813169  43.24331852  6.246257119  5.285294486 |
| 12824 ENSG00000187664 2.02227258 4.214465159 2.167669503 1.94423788 0.05186677183 0.1144877713 0 0 0 0 0 0 0 0 1.394264792 8.345079432 8.281669619 6.246257119 | | | | | | | | | | | | | | | | | | | |
| 12826  12833  12836  12838  12840  12843  12844  12846  12866 | ENSG00000109321 ENSG00000277011 ENSG00000165164 ENSG00000231808 ENSG00000250072 ENSG00000258794 ENSG00000258667 ENSG00000103522 ENSG00000251978 | 2941.368669  1.523765102  2.012052163  1.909715478  1.061269822  1.869540368  8.644222424  5.162296228  0.8359579773 | 2.091210311  3.190416019  4.143507317  3.38257919  2.712274762  4.13526024  2.185515943  4.187520802  3.183230832 | 1.075854158  1.64294345  2.134394162  1.742841844  1.39783036  2.131823549  1.126720063  2.159086123  1.643592961 | 1.943767466  1.941890343  1.941303716  1.940841162  1.940346153  1.939776039  1.93971512  1.939487618  1.936751317 | 0.05192349873  0.05215037674  0.05222144909  0.05227754651  0.05233763586  0.0524069138  0.05241432098  0.05244199076  0.05277574947 | 0.1145911863  0.115025745  0.1151589543  0.115264701  0.115379215  0.1155118538  0.1155122835  0.1155552694  0.1161114064 | 1160.240146  0  0  0  0  0  1.993539769  0  0 | 718.0198795  0  0  0  0  0  5.021118038  0.7173025769  0 | 1138.086037  0  0  0.780045262  0.780045262  0  1.560090524  0  0 | 690.7533014  0.9271856394  0  0  0  0  1.854371279  0.9271856394  0 | 6799.333457  0.9689801135  0  0  0.9689801135  0  31.00736363  31.97634375  2.906940341 | 8951.882495  3.891699813  0  0  1.945849906  0  13.62094935  0  0.9729249532 | 6559.989901  5.242599533  0  0  3.744713952  0  27.71088325  27.71088325  2.246828371 | 8842.223942  6.774234807  0  0.615839528  2.463358112  0  19.09102537  0.615839528  2.463358112 | 133.8494201  0  5.57705917  2.788529585  0  4.182794377  0  0  0 | 79.27825461  0  2.781693144  8.345079432  1.390846572  8.345079432  1.390846572  0  0 | 144.9292183  0  12.42250443  4.14083481  0  4.14083481  0  0  0 | 77.83797333  0.4804813169  3.363369218  6.246257119  1.441443951  5.765775802  0.4804813169  0  1.441443951 |
| 12867 ENSG00000179593 165.9129694 3.327323007 1.718037973 1.936699339 0.05278210661 0.1161148916 45.8514147 7.890328346 34.32199153 6.490299476 846.8886192 120.6426942 802.8666714 123.1679056 1.394264792 0 0 1.441443951 | | | | | | | | | | | | | | | | | | | |
| 12870  12874  12878  12888  12894  12895  12896  12901  12905 | ENSG00000254912 ENSG00000139973 ENSG00000202415 ENSG00000169688 ENSG00000283491 ENSG00000239002 ENSG00000245534 ENSG00000150893 ENSG00000230707 | 2.070942787  1.393468108  1.037996477  2.340060011  2.528095099  1.654933903  6.971657763  3.514295099  1.838218007 | 2.685191854  3.012355588  2.60576654  2.747529294  2.048306749  2.938901671  3.028186163  2.882800296  4.122154579 | 1.386876305  1.556497661  1.347119233  1.421523116  1.060279884  1.521332935  1.567776407  1.493153824  2.136481719 | 1.936143725  1.935342187  1.934325096  1.932806623  1.931854768  1.931793892  1.931516605  1.930678708  1.929412521 | 0.05285010055  0.05294831863  0.05307316953  0.05326002413  0.05337743393  0.05338495032  0.05341919782  0.05352279757  0.05367967058 | 0.1162404941  0.1164172058  0.1166554695  0.1169753447  0.1171786602  0.1171860724  0.1172521567  0.1174340212  0.1177234644 | 0  0  0.9967698847  0  0.9967698847  0  2.990309654  0.9967698847  0 | 0.7173025769  0  0  0  0.7173025769  0  0  0.7173025769  0 | 0  0.780045262  0  0  1.560090524  0  0  0  0 | 0.9271856394  0  0  1.854371279  0  0.9271856394  1.854371279  0.9271856394  0 | 0.9689801135  0  1.937960227  0  0.9689801135  0.9689801135  1.937960227  0  0 | 0.9729249532  0  1.945849906  0.9729249532  0  0  0  0  0 | 0.7489427904  2.246828371  1.497885581  0.7489427904  3.744713952  0  0  0.7489427904  0 | 0  2.463358112  1.847518584  1.231679056  3.07919764  1.231679056  0  0  0 | 5.57705917  1.394264792  2.788529585  8.365588755  2.788529585  1.394264792  26.49103106  11.15411834  5.57705917 | 6.95423286  6.95423286  0  2.781693144  4.172539716  4.172539716  11.12677258  9.735926004  4.172539716 | 4.14083481  0  0  8.281669619  4.14083481  8.281669619  24.84500886  8.281669619  4.14083481 | 3.843850535  2.882887901  1.441443951  3.843850535  8.168182387  2.882887901  14.41443951  9.609626337  8.168182387 |
| 12906 ENSG00000258077 1.338736518 2.988058236 1.54868731 1.929413522 0.05367954637 0.1177234644 0.9967698847 0 0 0 4.844900568 2.91877486 3.744713952 3.07919764 0 0 0 0.4804813169 | | | | | | | | | | | | | | | | | | | |
| 12907  12912  12913  12914  12915  12918  12919  12922  12928 | ENSG00000229891 ENSG00000204818 ENSG00000176268 ENSG00000153930 ENSG00000198155 ENSG00000206603 ENSG00000260903 ENSG00000277669 ENSG00000223393 | 2.365185536  21.70763111  1.195831982  2.860371305  21.57487514  1.040155281  2.419423962  2.860535843  0.7836703308 | 2.940564674  4.294354527  2.629030352  2.847152837  2.040099024  3.356165223  3.753658729  4.795201822  3.008637649 | 1.524039369  2.226582091  1.36310952  1.476181353  1.057861133  1.740383857  1.946553845  2.488163989  1.561819162 | 1.929454536  1.92867559  1.928700748  1.928728358  1.928513074  1.928405168  1.928361108  1.927204896  1.926367484 | 0.05367445902  0.05377114848  0.05376802334  0.05376459378  0.05379133968  0.05380474957  0.05381022588  0.05395410008  0.05405850457 | 0.1177234644  0.1178667927  0.1178667927  0.1178667927  0.1178861614  0.1178972938  0.1179001667  0.1181879552  0.1183525432 | 0.9967698847  4.983849424  0  0  5.980619308  0  0  0  0 | 0  0  0  2.151907731  7.890328346  0  0  0  0 | 0.780045262  1.560090524  0.780045262  0  9.360543144  0  0  0  0 | 0  0  0  0  4.635928197  0  0.9271856394  0  0 | 0.9689801135  59.10778693  0.9689801135  9.689801135  2.906940341  0  0  0  1.937960227 | 0  54.48379738  0.9729249532  5.837549719  0  0.9729249532  0  0  0 | 0.7489427904  79.38793579  1.497885581  11.23414186  3.744713952  0  0  0  0.7489427904 | 0  60.96811327  1.231679056  1.231679056  1.231679056  1.231679056  0  0  0.615839528 | 4.182794377  0  1.394264792  2.788529585  46.01073815  4.182794377  6.971323962  4.182794377  2.788529585 | 6.95423286  0  0  1.390846572  77.88740804  4.172539716  8.345079432  16.69015886  1.390846572 | 4.14083481  0  4.14083481  0  37.26751329  0  4.14083481  0  0 | 9.609626337  0  3.363369218  0  61.98208988  1.921925267  8.648663704  13.45347687  1.921925267 |
| 12930 ENSG00000225706 1.882474565 4.166234317 2.163023023 1.926116492 0.05408982986 0.1183801442 0 0 0 0 0 0 0 0 5.57705917 2.781693144 4.14083481 10.09010765 | | | | | | | | | | | | | | | | | | | |
| 12931  12953  12956  12963  12967  12969  12978  12988  13006 | ENSG00000259543 ENSG00000168135 ENSG00000196664 ENSG00000272572 ENSG00000207357 ENSG00000179314 ENSG00000225594 ENSG00000122176 ENSG00000104967 | 2.74155492  1.885556922  0.9012391951  1.560464504  1.092527232  13.53085669  1.294143079  4.356600088  3.460215719 | 4.733446336  3.36491272  3.139136987  2.418684928  2.766907045  2.876736416  2.873335575  3.43795117  2.858208171 | 2.457470739  1.749130228  1.632389058  1.258338936  1.439899494  1.497137479  1.496635631  1.792485758  1.492488489 | 1.926145553  1.923763404  1.923032363  1.922125159  1.921597345  1.92149115  1.919863136  1.917979629  1.915062121 | 0.05408620209  0.05438424656  0.05447598564  0.05459001099  0.0546564429  0.054669817  0.05487518945  0.05511359421  0.05548458176 | 0.1183801442  0.1188563729  0.1190180032  0.1192027194  0.1193109642  0.119321755  0.1196869404  0.1200958736  0.1207555414 | 0  0.9967698847  0  0  0  1.993539769  0.9967698847  0  0 | 0  0  0  0  0  0  0  0.7173025769  0 | 0  0  0  0.780045262  0.780045262  5.460316834  0  1.560090524  0 | 0  0  0  0.9271856394  0  2.781556918  0  0  2.781556918 | 0  0.9689801135  2.906940341  1.937960227  2.906940341  0.9689801135  0.9689801135  19.37960227  0 | 0  0  0.9729249532  0.9729249532  0.9729249532  0  0  1.945849906  0 | 0  0  0.7489427904  4.493656743  0.7489427904  0  0.7489427904  26.21299767  2.246828371 | 0  0  0.615839528  3.07919764  2.463358112  0  0.615839528  2.463358112  1.231679056 | 9.759853547  2.788529585  2.788529585  4.182794377  1.394264792  36.2508846  4.182794377  0  6.971323962 | 11.12677258  5.563386288  2.781693144  1.390846572  0  34.7711643  4.172539716  0  9.735926004 | 0  4.14083481  0  0  0  45.54918291  0  0  4.14083481 | 12.01203292  8.168182387  0  0.9609626337  3.843850535  34.59465481  3.843850535  0  14.41443951 |
| 13009 ENSG00000275567 1.794246301 4.076331932 2.129007618 1.914662915 0.05553550592 0.1208384988 0 0 0 0 0 0 0 0 6.971323962 4.172539716 4.14083481 6.246257119 | | | | | | | | | | | | | | | | | | | |
| 13010  13011  13014  13022  13024  13028  13029  13036  13040 | ENSG00000188257 ENSG00000231566 ENSG00000248599 ENSG00000235899 ENSG00000232422 ENSG00000267175 ENSG00000283211 ENSG00000002745 ENSG00000183092 | 1.883461525  2.976476416  1.234324427  2.945949472  0.8720956647  1.707049305  1.358500195  4.220947771  95.74354071 | 4.222337037  2.682383638  2.885995476  2.728086519  3.099158822  2.573905079  3.779191681  3.17464661  2.179814792 | 2.205452042  1.401077955  1.508111846  1.426128166  1.620715028  1.346116143  1.976585048  1.661993168  1.141539967 | 1.914499593  1.914514198  1.913648171  1.912932221  1.912216996  1.912097327  1.911980304  1.9101442  1.909538741 | 0.05555635106  0.05555448672  0.05566512556  0.05575673005  0.05584836701  0.05586371166  0.0558787204  0.05611464938  0.05619262909 | 0.1208652735  0.1208652735  0.1210585329  0.1211987407  0.1213792903  0.1213956548  0.1213986537  0.1218457552  0.1219776502 | 0  0.9967698847  0.9967698847  0.9967698847  0  0  0  0.9967698847  5.980619308 | 0  0  0  0.7173025769  0  0  0  0.7173025769  48.05927265 | 0  0  0  0  0  0.780045262  0  0  11.70067893 | 0  1.854371279  0  0.9271856394  0  0.9271856394  0  0.9271856394  48.21365325 | 6.782860795  5.813880681  1.937960227  12.59674148  1.937960227  3.875920454  4.844900568  0  6.782860795 | 0.9729249532  7.783399626  0  1.945849906  0.9729249532  1.945849906  2.91877486  0  0.9729249532 | 14.22991302  5.991542324  2.246828371  11.23414186  0.7489427904  5.991542324  2.995771162  0  7.489427904 | 0.615839528  12.31679056  4.926716224  5.542555752  1.231679056  3.695037168  5.542555752  0  2.463358112 | 0  0  0  0  4.182794377  2.788529585  0  20.91397189  178.4658934 | 0  0  2.781693144  1.390846572  1.390846572  0  0  11.12677258  299.032013 | 0  0  0  0  0  0  0  8.281669619  223.6050797 | 0  0.9609626337  1.921925267  0  0  0.4804813169  0  7.68770107  316.1567065 |
| 13046 ENSG00000130755 6.372771156 2.355520301 1.234094904 1.908702721 0.05630045191 0.1221554953 2.990309654 1.434605154 0.780045262 1.854371279 21.3175625 12.64802439 26.96194046 8.005913863 0 0 0 0.4804813169 | | | | | | | | | | | | | | | | | | | |
| 13050 | ENSG00000254703 | 10.43322536 | 3.105554587 | 1.62764301 | 1.908007203 | 0.05639028531 | 0.1223129054 | 2.990309654 | 2.869210308 | 0 | 0.9271856394 | 0 | 0 | 0 | 0 | 39.03941419 | 18.08100544 | 37.26751329 | 24.02406584 |

| 13058  13059  13068  13069  13079  13081  13086  13087 | ENSG00000256538 ENSG00000169744 ENSG00000141668 ENSG00000126752 ENSG00000273980 ENSG00000182759 ENSG00000181585 ENSG00000205669 | 2.098639207  1.447807989  1.830639666  1.330021585  1.10675671  3.427365489  1.347160844  2.126535735 | 2.734712513  3.862072735  4.102163577  3.747313208  3.190534122  2.204862179  3.755524733  2.844415893 | 1.433879866  2.025230133  2.153162901  1.967162236  1.67707558  1.15900916  1.975365691  1.496153135 | 1.90721174  1.906979692  1.905180317  1.904933482  1.902439079  1.902368208  1.901179488  1.901152914 | 0.05649317385  0.0565232173  0.05675663592  0.05678871831  0.05711377611  0.05712303425  0.05727850681  0.05728198643 | 0.1224610031  0.1225167462  0.1229379658  0.1229980458  0.1236075041  0.1236084917  0.1238952908  0.1238952908 | 0  0  0  0  0  0.9967698847  0  1.993539769 | 0  0  0  0  0  1.434605154  0  0 | 0.780045262  0  0  0  0  1.560090524  0  0 | 0.9271856394  0  0  0  0  0  0  0 | 0.9689801135  2.906940341  0  2.906940341  0.9689801135  0.9689801135  2.906940341  4.844900568 | 0  6.810474673  0  4.864624766  0  0  0  5.837549719 | 1.497885581  1.497885581  0  4.493656743  1.497885581  1.497885581  5.242599533  5.242599533 | 1.847518584  6.15839528  0  3.695037168  0.615839528  1.231679056  0  6.15839528 | 0  0  9.759853547  0  5.57705917  5.57705917  0  0 | 9.735926004  0  2.781693144  0  0  8.345079432  4.172539716  0 | 4.14083481  0  4.14083481  0  4.14083481  4.14083481  0  0 | 5.285294486  0  5.285294486  0  0.4804813169  15.37540214  3.843850535  1.441443951 |
| --- | --- | --- | --- | --- | --- | --- | --- | --- | --- | --- | --- | --- | --- | --- | --- | --- | --- | --- | --- |
| 13090 ENSG00000222000 1.772073423 2.424081958 1.275428103 1.900602592 0.0573540858 0.1240232813 0.9967698847 0 0.780045262 0 0 1.945849906 0.7489427904 1.847518584 2.788529585 4.172539716 4.14083481 3.843850535 | | | | | | | | | | | | | | | | | | | |
| 13092  13100  13101  13106  13114  13116  13144  13146  13149 | ENSG00000137491 ENSG00000220884 ENSG00000186417 ENSG00000166148 ENSG00000213872 ENSG00000217455 ENSG00000278318 ENSG00000134612 ENSG00000129910 | 8.956523373  1.355103123  4.797451095  1.754206191  2.615867853  1.590724682  65.06140014  1.787102925  1.997560708 | 4.839702891  3.776567261  2.576346897  4.040003488  2.038140871  3.986766243  2.930074845  4.007116458  3.552528465 | 2.546895386  1.98842642  1.356700864  2.127791829  1.07439858  2.102120216  1.548871024  2.118382201  1.878528049 | 1.900236232  1.899274333  1.89897933  1.898683618  1.897006296  1.896545313  1.891748764  1.891592771  1.89112346 | 0.05740212549  0.05752841583  0.05756719376  0.05760608681  0.05782710714  0.05788797417  0.05852446276  0.05854525978  0.05860786547 | 0.1241082007  0.124305293  0.1243772934  0.1244183131  0.1248173017  0.1249296277  0.1260443019  0.1260597994  0.1261658103 | 0  0  0.9967698847  0  0  0  14.95154827  0  0 | 0  0  0  0  0.7173025769  0  10.04223608  0  0.7173025769 | 0  0  0  0  0.780045262  0  15.60090524  0  0 | 1.854371279  0  3.708742558  0  1.854371279  0  7.417485115  0  0 | 0  2.906940341  21.3175625  0  0  4.844900568  0  0  11.62776136 | 0  2.91877486  3.891699813  0  2.91877486  3.891699813  1.945849906  0  1.945849906 | 0  6.740485114  21.71934092  0  3.744713952  9.736256276  0  0  8.238370695 | 0  3.695037168  1.231679056  0  1.231679056  0.615839528  1.231679056  0  0 | 8.365588755  0  0  6.971323962  8.365588755  0  177.0716286  5.57705917  0 | 40.33455059  0  2.781693144  4.172539716  1.390846572  0  208.6269858  2.781693144  0 | 16.56333924  0  0  4.14083481  4.14083481  0  144.9292183  8.281669619  0 | 40.36043062  0  1.921925267  5.765775802  6.246257119  0  198.9192652  4.804813169  1.441443951 |
| 13151 ENSG00000199347 0.9431265587 2.937306853 1.553529904 1.890730809 0.05866028754 0.1262594555 0 0 0 0 0 0.9729249532 0.7489427904 1.231679056 0 2.781693144 4.14083481 1.441443951 | | | | | | | | | | | | | | | | | | | |
| 13159  13165  13189  13199  13200  13206  13209  13216  13218 | ENSG00000275591 ENSG00000104043 ENSG00000147655 ENSG00000232936 ENSG00000259700 ENSG00000166105 ENSG00000118526 ENSG00000259929 ENSG00000255559 | 2.378893719  2.184841024  2.530486524  1.129760876  1.314935838  10.13792423  1.899644913  2.322573305  2.332497048 | 3.67600457  2.825564589  3.743266987  3.49226029  3.355923111  2.285380539  4.103124564  3.664583901  2.23975729 | 1.94538993  1.495782916  1.985476824  1.854538708  1.782241758  1.213890074  2.179575639  1.948127763  1.190949893 | 1.889597819  1.889020497  1.885323939  1.88308838  1.882978611  1.882691513  1.882533687  1.881079861  1.880647795 | 0.05881176906  0.05888908223  0.05938611603  0.05968839175  0.05970326664  0.05974218631  0.05976359051  0.05996105605  0.06001984555 | 0.1265085443  0.1266171182  0.1274437756  0.128007928  0.128007928  0.1280525765  0.1280693613  0.1284244592  0.1285309236 | 0.9967698847  1.993539769  0.9967698847  0  0  1.993539769  0  0  0 | 0  0  0  0  0  1.434605154  0  0.7173025769  0 | 0  0  0  0  0  2.340135786  0  0  1.560090524 | 0  0  0  0  0  5.563113837  0  0  0.9271856394 | 0  0  0  0  1.937960227  0  0  0  2.906940341 | 0  1.945849906  0  0  0  2.91877486  0  0  0.9729249532 | 0  1.497885581  0  0.7489427904  2.246828371  0.7489427904  0  0  1.497885581 | 0  0.615839528  0  0.615839528  0  0  0  0  1.847518584 | 9.759853547  4.182794377  1.394264792  1.394264792  0  16.73117751  9.759853547  2.788529585  2.788529585 | 2.781693144  9.735926004  8.345079432  6.95423286  1.390846572  30.59862459  1.390846572  6.95423286  0 | 8.281669619  0  12.42250443  0  8.281669619  16.56333924  8.281669619  8.281669619  8.281669619 | 6.726738436  6.246257119  7.207219753  3.843850535  1.921925267  42.7628372  3.363369218  9.129145021  7.207219753 |
| 13220 ENSG00000271192 1.448136726 3.860686583 2.05326474 1.880267317 0.06007165519 0.1286126822 0 0 0 0 1.937960227 7.783399626 1.497885581 6.15839528 0 0 0 0 | | | | | | | | | | | | | | | | | | | |
| 13221  13222  13223  13228  13249  13260  13262  13273  13275 | ENSG00000259326 ENSG00000164591 ENSG00000231107 ENSG00000203523 ENSG00000221676 ENSG00000173401 ENSG00000268951 ENSG00000207181 ENSG00000229311 | 2.38368189  0.8628510714  1.764809879  1.27178294  1.437580391  1.07975774  2.973836835  2.156459981  1.903284111 | 3.062422397  3.172501486  4.109924297  3.523127679  2.388626653  2.698578413  3.32801532  2.203136512  3.281338769 | 1.628701238  1.6874201  2.185974046  1.874330443  1.272636539  1.439399131  1.775467583  1.176506876  1.752480999 | 1.880284932  1.8800899  1.880134078  1.879672655  1.876911891  1.874795083  1.874444429  1.872608275  1.872396203 | 0.06006925572  0.06009582676  0.06008980716  0.06015270459  0.06053017085  0.06082091992  0.06086919464  0.06112249779  0.06115180996 | 0.1286126822  0.1286309342  0.1286309342  0.1287129546  0.1293240231  0.1298338582  0.1299173144  0.1303440523  0.1303828813 | 0  0  0  0  0  0  0.9967698847  0  0 | 0.7173025769  0  0  0  0.7173025769  0  0  2.151907731  0 | 0.780045262  0  0  0  0  0.780045262  0.780045262  0  0.780045262 | 0  0  0  0  0.9271856394  0  0  0  0 | 0  0  0.9689801135  0  2.906940341  3.875920454  0  0.9689801135  0 | 1.945849906  2.91877486  0  0  1.945849906  0.9729249532  0  0.9729249532  0.9729249532 | 0.7489427904  0.7489427904  0  0.7489427904  4.493656743  3.744713952  1.497885581  1.497885581  0 | 0  2.463358112  0  0  2.463358112  1.231679056  0  3.07919764  0.615839528 | 13.94264792  0  1.394264792  1.394264792  1.394264792  0  6.971323962  6.971323962  0 | 2.781693144  2.781693144  11.12677258  4.172539716  0  1.390846572  13.90846572  4.172539716  8.345079432 | 0  0  0  4.14083481  0  0  0  4.14083481  8.281669619 | 7.68770107  1.441443951  7.68770107  4.804813169  2.402406584  0.9609626337  11.5315516  1.921925267  3.843850535 |
| 13276 ENSG00000267649 0.8362839128 3.142519786 1.678333816 1.872404498 0.06115066318 0.1303828813 0 0 0 0 0.9689801135 0 2.995771162 1.847518584 0 2.781693144 0 1.441443951 | | | | | | | | | | | | | | | | | | | |
| 13280  13283  13288  13294  13299  13305  13308  13319  13341 | ENSG00000171243 ENSG00000015413 ENSG00000005001 ENSG00000237687 ENSG00000277595 ENSG00000158077 ENSG00000278934 ENSG00000230634 ENSG00000234465 | 0.9350024856  7.038250535  1.898177934  2.50102941  0.7495994225  1.456590976  1.713311526  1.255264413  2.348128083 | 3.273614702  2.926859571  2.740550453  4.598754834  2.945391018  2.383757178  4.002758749  2.928899898  2.658121048 | 1.748864543  1.563979035  1.465016632  2.461511183  1.577813972  1.277945415  2.14662907  1.572212328  1.429696085 | 1.871851491  1.871418674  1.870661666  1.868264855  1.866754301  1.865304379  1.864671826  1.862916252  1.859221044 | 0.06122715229  0.06128707252  0.06139199133  0.06172516281  0.06193590693  0.0621387517  0.06222741817  0.0624740499  0.06299581317 | 0.1305041998  0.1306024147  0.130776769  0.1314271445  0.1318262863  0.1321983845  0.132357176  0.1327720141  0.133660107 | 0  0.9967698847  0  0  0  0.9967698847  0  0  0 | 0  0  0  0  0  0  0  0  0 | 0  1.560090524  1.560090524  0  0  0.780045262  0  0  0 | 0  2.781556918  0  0  0  0  0  0.9271856394  1.854371279 | 2.906940341  37.79022443  8.720821022  0  0.9689801135  1.937960227  0  0.9689801135  0 | 1.945849906  4.864624766  2.91877486  0  2.91877486  1.945849906  0  2.91877486  0.9729249532 | 3.744713952  28.45982604  5.991542324  0  0.7489427904  1.497885581  0  3.744713952  0.7489427904 | 1.231679056  8.005913863  1.231679056  0  0.615839528  3.695037168  0  5.542555752  1.847518584 | 0  0  1.394264792  8.365588755  0  0  2.788529585  0  5.57705917 | 1.390846572  0  0  12.51761915  2.781693144  2.781693144  8.345079432  0  1.390846572 | 0  0  0  0  0  0  4.14083481  0  12.42250443 | 0  0  0.9609626337  9.129145021  0.9609626337  3.843850535  5.285294486  0.9609626337  3.363369218 |
| 13342 ENSG00000226423 1.688823258 4.06547201 2.186766223 1.859125117 0.06300940585 0.1336789268 0 0 0 0 9.689801135 0.9729249532 8.987313485 0.615839528 0 0 0 0 | | | | | | | | | | | | | | | | | | | |
| 13345  13361  13378  13383  13391  13396  13404  13414  13426 | ENSG00000256633 ENSG00000004848 ENSG00000264672 ENSG00000170743 ENSG00000273237 ENSG00000268658 ENSG00000214076 ENSG00000249328 ENSG00000172425 | 0.9370134213  2.433756533  2.856680307  1.180895633  5.298958569  1.26449279  6.902316613  2.544446154  0.9650444561 | 3.268046758  2.945257658  2.633993284  3.378984797  2.050879767  2.936864445  2.451573323  2.16309193  3.307726187 | 1.758376119  1.587142138  1.422150405  1.82539869  1.108484364  1.587884623  1.326307106  1.171673885  1.793339566 | 1.858559567  1.855698735  1.852120053  1.851094128  1.850165716  1.849545239  1.84842056  1.846155281  1.844450571 | 0.06308959276  0.06349651128  0.06400858559  0.06415601265  0.06428966868  0.06437912172  0.0645415267  0.06486966199  0.06511750313 | 0.1338189594  0.134520788  0.1354333251  0.1356945449  0.1358960019  0.1360342953  0.136296065  0.1368868833  0.1372870582 | 0  1.993539769  1.993539769  0  1.993539769  0  1.993539769  0  0 | 0  0  0  0  1.434605154  0.7173025769  1.434605154  1.434605154  0 | 0  0  0.780045262  0  3.120181048  0  2.340135786  0.780045262  0 | 0  0  0  0  0  0  0.9271856394  0.9271856394  0 | 0.9689801135  0  13.56572159  0  0  1.937960227  0  5.813880681  2.906940341 | 0.9729249532  2.91877486  0  0  3.891699813  4.864624766  0  0  2.91877486 | 2.246828371  0.7489427904  8.987313485  0  3.744713952  2.246828371  0  8.238370695  3.744713952 | 0  0  1.847518584  0.615839528  0  4.926716224  0.615839528  0.615839528  0.615839528 | 0  2.788529585  0  2.788529585  13.94264792  0  18.1254423  1.394264792  1.394264792 | 4.172539716  5.563386288  2.781693144  2.781693144  13.90846572  0  16.69015886  5.563386288  0 | 0  4.14083481  0  4.14083481  12.42250443  0  24.84500886  0  0 | 2.882887901  11.05107029  4.324331852  3.843850535  9.129145021  0.4804813169  15.85588346  5.765775802  0 |
| 13428 ENSG00000156475 1.698745648 3.187446057 1.728559377 1.843989914 0.06518461016 0.137403979 0 0.7173025769 0 0 0.9689801135 0 0 0 6.971323962 2.781693144 4.14083481 4.804813169 | | | | | | | | | | | | | | | | | | | |
| 13430  13440  13441  13442  13444  13445  13452  13465  13468 | ENSG00000283162 ENSG00000235595 ENSG00000225096 ENSG00000229821 ENSG00000142609 ENSG00000089558 ENSG00000179082 ENSG00000153495 ENSG00000171815 | 3.11936322  1.561768346  3.063449566  1.78345618  1.486132254  1.666697744  1.024739964  1.126677608  1.329772442 | 2.109118667  2.979784683  3.31103577  4.000693911  3.078210368  3.882417045  2.598159601  2.705542782  2.78937326 | 1.143844602  1.617966922  1.797967025  2.172585758  1.672453575  2.109633792  1.412765932  1.473420377  1.519494745 | 1.843885666  1.84168455  1.841544213  1.841443495  1.840535615  1.840327482  1.839058786  1.836232771  1.83572419 | 0.06519980445  0.06552130538  0.06554184775  0.06555659381  0.06568964049  0.06572017291  0.06590654005  0.06632323782  0.06639845846 | 0.137409401  0.1379944993  0.1380274937  0.1380482774  0.138307867  0.1383618605  0.1386820192  0.1394241047  0.1395511409 | 0  0  0.9967698847  0  0  0  0.9967698847  0  0 | 1.434605154  0  0.7173025769  0  0  0  0  0  0 | 0.780045262  0  0  0  0.780045262  0  0  0.780045262  0 | 1.854371279  0.9271856394  0  0  0  0  0  0  0.9271856394 | 3.875920454  0  0  0  0  0  2.906940341  0  1.937960227 | 4.864624766  0.9729249532  0  0  0.9729249532  0  1.945849906  2.91877486  0 | 12.73202744  0  0  0  0  0  2.995771162  0.7489427904  0.7489427904 | 8.621753391  2.463358112  0  0  0.615839528  0  0.615839528  0.615839528  0.615839528 | 2.788529585  8.365588755  6.971323962  8.365588755  5.57705917  4.182794377  1.394264792  4.182794377  0 | 0  1.390846572  5.563386288  1.390846572  5.563386288  4.172539716  0  1.390846572  2.781693144 | 0  4.14083481  12.42250443  8.281669619  0  8.281669619  0  0  4.14083481 | 0.4804813169  0.4804813169  10.09010765  3.363369218  4.324331852  3.363369218  1.441443951  2.882887901  4.804813169 |
| 13476 ENSG00000277358 8.174702281 2.798167942 1.525103908 1.834739212 0.06654433929 0.1397747156 0 0.7173025769 4.680271572 0.9271856394 0 0 0.7489427904 1.847518584 1.394264792 1.390846572 45.54918291 40.84091193 | | | | | | | | | | | | | | | | | | | |
| 13487 | ENSG00000255650 | 1.829768482 | 2.004506114 | 1.093929562 | 1.832390479 | 0.0668932656 | 0.1403930285 | 0.9967698847 | 0 | 0 | 1.854371279 | 0.9689801135 | 2.91877486 | 2.995771162 | 1.847518584 | 2.788529585 | 2.781693144 | 0 | 4.804813169 |

| 13492  13493  13495  13532  13544  13546  13561  13567 | ENSG00000258534 ENSG00000179178 ENSG00000135298 ENSG00000257444 ENSG00000207922 ENSG00000217236 ENSG00000206832 ENSG00000249868 | 1.174997083  0.8992226176  0.7529334101  0.9570031196  1.213466691  3.894616346  0.9408414982  0.7643540853 | 2.846199942  2.91091695  2.956528104  3.263009437  2.626523677  3.602798319  3.248803169  2.973986734 | 1.554099578  1.58968991  1.614709659  1.786328087  1.440605763  1.976224444  1.783775628  1.63365635 | 1.83141414  1.831122492  1.830996729  1.82665741  1.823207809  1.823071428  1.821307073  1.820448183 | 0.06703875236  0.06708226207  0.0671010313  0.06775129559  0.06827192277  0.06829257339  0.06856019184  0.06869078046 | 0.1406462292  0.1407270815  0.1407436294  0.141721523  0.1426750364  0.1427055649  0.1431052469  0.1433154885 | 0  0  0  0  0.9967698847  0  0  0 | 0.7173025769  0  0  0  0  0  0  0 | 0  0  0  0  0  0  0  0 | 0  0  0  0  0  1.854371279  0  0 | 3.875920454  0.9689801135  2.906940341  0  1.937960227  0  0  1.937960227 | 0.9729249532  0.9729249532  0  0  0  0  1.945849906  0.9729249532 | 4.493656743  2.995771162  2.246828371  1.497885581  0.7489427904  0  0.7489427904  2.995771162 | 3.07919764  1.231679056  0.615839528  0.615839528  2.463358112  0  0.615839528  0 | 0  0  1.394264792  5.57705917  0  5.57705917  5.57705917  1.394264792 | 0  0  1.390846572  1.390846572  1.390846572  13.90846572  0  1.390846572 | 0  4.14083481  0  0  4.14083481  12.42250443  0  0 | 0.9609626337  0.4804813169  0.4804813169  2.402406584  2.882887901  12.97299556  2.402406584  0.4804813169 |
| --- | --- | --- | --- | --- | --- | --- | --- | --- | --- | --- | --- | --- | --- | --- | --- | --- | --- | --- | --- |
| 13573 ENSG00000249502 1.183084972 2.721792306 1.495714701 1.819726921 0.06880060155 0.1434811632 0.9967698847 0 0 0 0.9689801135 0.9729249532 2.246828371 0.615839528 0 6.95423286 0 1.441443951 | | | | | | | | | | | | | | | | | | | |
| 13576  13580  13582  13586  13596  13601  13614  13621  13626 | ENSG00000047617 ENSG00000251654 ENSG00000273706 ENSG00000261395 ENSG00000219790 ENSG00000187536 ENSG00000231057 ENSG00000189152 ENSG00000226083 | 0.7472224309  1.371041315  7.723386849  2.384678077  1.297611431  1.579268856  0.992403672  1.815186419  0.8446513913 | 3.015268576  3.772746514  2.410684495  2.164149337  2.662956778  2.39788604  2.5116473  2.700642978  3.026545886 | 1.657345177  2.074351935  1.325593595  1.190296425  1.465568212  1.320122527  1.384414285  1.489235417  1.67029641 | 1.819336502  1.818759127  1.818569812  1.818159991  1.817013194  1.816411727  1.814230991  1.813442621  1.811981316 | 0.06886010778  0.06894818665  0.06897708694  0.06903968306  0.06921509308  0.06930723762  0.06964217082  0.06976358088  0.06998908359 | 0.1435735276  0.1437148285  0.1437538966  0.1438419894  0.1441119194  0.1442401785  0.1447988311  0.1449767213  0.1453919712 | 0  0  0  0  0.9967698847  0  0.9967698847  0  0 | 0  0  3.586512885  1.434605154  0  0.7173025769  0  0  0 | 0  0  2.340135786  0  0  0  0  1.560090524  0 | 0  0  1.854371279  0.9271856394  0  0.9271856394  0  0  0 | 0  5.813880681  0.9689801135  1.937960227  0  0  0  2.906940341  0.9689801135 | 0.9729249532  6.810474673  0  1.945849906  0  1.945849906  1.945849906  5.837549719  0.9729249532 | 0.7489427904  0.7489427904  0.7489427904  0.7489427904  0.7489427904  0.7489427904  1.497885581  4.493656743  0.7489427904 | 0.615839528  3.07919764  0  1.231679056  1.231679056  2.463358112  1.847518584  5.542555752  0 | 1.394264792  0  32.06809023  8.365588755  2.788529585  4.182794377  2.788529585  0  4.182794377 | 1.390846572  0  19.47185201  2.781693144  2.781693144  5.563386288  1.390846572  0  2.781693144 | 0  0  12.42250443  8.281669619  4.14083481  0  0  0  0 | 3.843850535  0  19.21925267  0.9609626337  2.882887901  2.402406584  1.441443951  1.441443951  0.4804813169 |
| 13631 ENSG00000186094 2.450903621 4.56104979 2.518122643 1.811289773 0.07009600797 0.1455592691 0 0 0 0 0 0 0 0 20.91397189 4.172539716 0 4.324331852 | | | | | | | | | | | | | | | | | | | |
| 13647  13654  13659  13661  13669  13680  13682  13685  13688 | ENSG00000125872 ENSG00000118194 ENSG00000136689 ENSG00000276434 ENSG00000285367 ENSG00000267670 ENSG00000244503 ENSG00000078579 ENSG00000086967 | 1.988234978  1.452308539  1.345004636  1.55372079  2.013759893  4.559525685  2.160565937  0.9343464784  1.174500354 | 3.525957971  3.809470832  3.44516618  3.840246868  2.048681375  2.974745378  2.131051651  3.23246305  3.391775252 | 1.948506503  2.106375043  1.905261801  2.123755351  1.133284722  1.646450338  1.179600236  1.790047839  1.879425311 | 1.809569516  1.808543471  1.80823768  1.808234111  1.807737575  1.806762894  1.806588017  1.805797018  1.80468744 | 0.07036257077  0.07052195748  0.07056951645  0.07057007182  0.07064735358  0.07079925655  0.07082653928  0.07095005142  0.07112360638 | 0.1459528162  0.1461878087  0.1462232965  0.1462232965  0.1462977534  0.1464944266  0.1465294563  0.1467528064  0.1470795443 | 0.9967698847  0  0  0  0  0.9967698847  0  0  0 | 0  0  0  0  0.7173025769  0  0  0  0 | 0  0  0  0  0.780045262  2.340135786  1.560090524  0  0 | 0  0  0  0  0.9271856394  0  0.9271856394  0  0 | 0.9689801135  0  0  0  0.9689801135  16.47266193  0.9689801135  0  0.9689801135 | 13.62094935  0  0  0  1.945849906  6.810474673  0  0  0 | 1.497885581  0  0.7489427904  0  1.497885581  19.47251255  1.497885581  0.7489427904  0 | 6.774234807  0.615839528  0  0  4.310876696  8.621753391  1.847518584  0.615839528  0 | 0  8.365588755  1.394264792  5.57705917  0  0  5.57705917  4.182794377  2.788529585 | 0  5.563386288  1.390846572  5.563386288  6.95423286  0  5.563386288  2.781693144  1.390846572 | 0  0  8.281669619  4.14083481  4.14083481  0  4.14083481  0  4.14083481 | 0  2.882887901  4.324331852  3.363369218  1.921925267  0  3.843850535  2.882887901  4.804813169 |
| 13701 ENSG00000136688 1.553435939 3.840387943 2.129697169 1.80325541 0.07134811192 0.1473822999 0 0 0 0 0 0 0 0 4.182794377 6.95423286 4.14083481 3.363369218 | | | | | | | | | | | | | | | | | | | |
| 13703  13720  13722  13728  13730  13743  13749  13767  13772 | ENSG00000184106 ENSG00000250764 ENSG00000174899 ENSG00000203785 ENSG00000262769 ENSG00000203364 ENSG00000215045 ENSG00000227076 ENSG00000276232 | 1.565800432  1.112111102  1.049815277  2.237614435  3.957252869  1.888000238  2.915091356  1.418566198  1.563573833 | 3.883956884  2.657832785  3.202551876  4.44636644  2.309096311  4.036354682  3.224626579  2.3545847  2.803642996 | 2.153816364  1.475762913  1.778591669  2.470714118  1.283377275  2.24680005  1.796913156  1.313053937  1.564043794 | 1.803290637  1.800989009  1.800611086  1.799628054  1.799234221  1.796490383  1.794536686  1.793212475  1.792560417 | 0.07134258227  0.07170461239  0.07176420062  0.07191938834  0.07198163832  0.07241656083  0.07272754852  0.07293895616  0.0730432405 | 0.1473822999  0.1479351865  0.1480365444  0.1482918274  0.1483985619  0.1491539817  0.1497291431  0.1499680463  0.1501279383 | 0  0.9967698847  0  0  0  0  0.9967698847  0  0 | 0  0  0  0  0.7173025769  0  0  1.434605154  0 | 0  0  0  0  0.780045262  0  0.780045262  0  0 | 0  0  0  0  2.781556918  0  0  0  0.9271856394 | 0  0  2.906940341  0  0  0  0  5.813880681  0.9689801135 | 0  0.9729249532  0.9729249532  0  0  0  0  0.9729249532  0 | 0  3.744713952  1.497885581  0  0.7489427904  0  0  2.246828371  0 | 0  0.615839528  3.07919764  0  1.231679056  0  0  1.847518584  0.615839528 | 2.788529585  4.182794377  0  8.365588755  8.365588755  6.971323962  5.57705917  1.394264792  1.394264792 | 4.172539716  1.390846572  0  6.95423286  11.12677258  2.781693144  9.735926004  1.390846572  4.172539716 | 4.14083481  0  4.14083481  0  8.281669619  12.42250443  8.281669619  0  8.281669619 | 7.68770107  1.441443951  0  11.5315516  13.45347687  0.4804813169  9.609626337  1.921925267  2.402406584 |
| 13778 ENSG00000222750 1.219979095 3.618996698 2.02114286 1.79056947 0.07336241 0.1507182739 0 0 0 0 5.813880681 4.864624766 1.497885581 2.463358112 0 0 0 0 | | | | | | | | | | | | | | | | | | | |
| 13781  13786  13793  13795  13796  13797  13815  13819  13825 | ENSG00000236754 ENSG00000241484 ENSG00000223951 ENSG00000215182 ENSG00000253955 ENSG00000237181 ENSG00000251230 ENSG00000153923 ENSG00000129295 | 1.932074101  17.59064972  1.594892498  2.906729864  1.376671076  1.526045174  1.497507192  1.100815414  31.57327023 | 2.595047042  2.223951467  3.934066433  4.083790967  3.00984564  3.842523263  3.890007448  3.235137286  2.036078784 | 1.450509213  1.243881397  2.20142968  2.285467892  1.684515773  2.1507372  2.179255149  1.813068477  1.141836665 | 1.789059331  1.787912796  1.787050692  1.786851166  1.786772014  1.786607523  1.785016982  1.784343684  1.783161152 | 0.07360526089  0.07379007808  0.07392929584  0.07396154715  0.07397434433  0.07400094488  0.07425856244  0.07436783592  0.07456007422 | 0.1511842765  0.1515089185  0.1517177299  0.151771767  0.1517771666  0.1518207397  0.1521507686  0.1523259132  0.1526514248 | 0  6.977389193  0  0  0  0  0  0  9.967698847 | 0  5.021118038  0  0  0  0  0  0  13.62874896 | 0  6.240362096  0  0.780045262  0  0  0  0  12.48072419 | 1.854371279  1.854371279  0  0  0.9271856394  0  0  0  4.635928197 | 2.906940341  1.937960227  0  58.13880681  1.937960227  0  0  0  0 | 4.864624766  0  0.9729249532  1.945849906  0  0  0.9729249532  0  1.945849906 | 5.242599533  0  0  28.45982604  0.7489427904  0  0  0  2.246828371 | 3.695037168  0.615839528  0  3.695037168  0.615839528  0  0  0.615839528  1.231679056 | 0  39.03941419  13.94264792  0  0  4.182794377  1.394264792  2.788529585  80.86735796 | 0  38.94370402  2.781693144  0  5.563386288  2.781693144  6.95423286  2.781693144  72.32402175 | 4.14083481  66.25335696  0  0  0  4.14083481  0  4.14083481  111.8025399 | 0.4804813169  44.20428115  1.441443951  0  6.726738436  7.207219753  8.648663704  2.882887901  67.74786568 |
| 13827 ENSG00000134343 0.9278420521 2.98795878 1.676003401 1.782788017 0.07462081713 0.1527603131 0 0 0 0 1.937960227 0 2.246828371 1.847518584 0 0 4.14083481 0.9609626337 | | | | | | | | | | | | | | | | | | | |
| 13833  13834  13835  13853  13858  13860  13861  13867  13869 | ENSG00000267939 ENSG00000255326 ENSG00000005513 ENSG00000251468 ENSG00000248771 ENSG00000278104 ENSG00000141505 ENSG00000065320 ENSG00000278041 | 2.298655429  0.7604201416  1.597122793  0.8972370899  1.299570555  1.251401144  5.889485902  29.69047576  1.550793863 | 2.337473061  3.048336641  3.898986297  2.489263088  3.706379383  2.887763142  2.139030723  2.062799313  3.763359987 | 1.312009653  1.711232267  2.188768398  1.400250616  2.085604962  1.625081461  1.203766958  1.16204373  2.120371 | 1.781597457  1.781369309  1.781360833  1.77772683  1.77712436  1.77699593  1.776947531  1.77514775  1.774859205 | 0.07481489938  0.07485213866  0.07485352235  0.07544873091  0.07554778098  0.07556890945  0.07557687302  0.07587349442  0.07592113762 | 0.153091198  0.1531480885  0.1531480885  0.1541652911  0.154311985  0.154332868  0.1543379964  0.1548766953  0.1549515986 | 0.9967698847  0  0  0  0  0.9967698847  3.987079539  15.94831816  0 | 0  0  0  0.7173025769  0  0  0.7173025769  7.890328346  0 | 0.780045262  0  0  0  0  0  2.340135786  8.580497882  0 | 0.9271856394  0  0  0  0  0  0  5.563113837  0 | 2.906940341  0.9689801135  0  1.937960227  6.782860795  0.9689801135  0.9689801135  0.9689801135  0 | 7.783399626  0  0  0.9729249532  0.9729249532  4.864624766  0  0  0 | 5.242599533  2.995771162  0  1.497885581  5.991542324  5.242599533  0.7489427904  2.246828371  0 | 6.15839528  1.847518584  0  1.847518584  1.847518584  2.463358112  1.231679056  1.231679056  0 | 2.788529585  0  1.394264792  0  0  0  5.57705917  79.47309317  4.182794377 | 0  1.390846572  8.345079432  1.390846572  0  0  23.64439173  90.40502718  2.781693144 | 0  0  4.14083481  0  0  0  16.56333924  57.97168734  8.281669619 | 0  1.921925267  5.285294486  2.402406584  0  0.4804813169  14.89492082  86.00615572  3.363369218 |
| 13871 ENSG00000146006 1.281468901 2.891611551 1.629434372 1.774610626 0.07596220141 0.1550130541 0 0 0 0.9271856394 0.9689801135 0 0 1.231679056 4.182794377 2.781693144 0 5.285294486 | | | | | | | | | | | | | | | | | | | |
| 13876  13886  13889  13896  13907  13916  13924  13929  13933 | ENSG00000271509 ENSG00000111886 ENSG00000196581 ENSG00000101134 ENSG00000157766 ENSG00000205403 ENSG00000180152 ENSG00000272379 ENSG00000009950 | 0.8486501162  1.415114683  1.060261456  1.594480718  1.485720212  53.17936106  1.200467686  3.344704402  98.89236271 | 3.154857009  2.859906901  3.189489642  3.830173538  3.799153049  3.034013445  2.457602791  2.133832229  2.103480454 | 1.77808018  1.613519752  1.799787751  2.162432771  2.147615846  1.716638971  1.391470883  1.208818803  1.192147651 | 1.774305256  1.77246476  1.772147655  1.77123358  1.7690096  1.767414987  1.766190598  1.765220911  1.764446251 | 0.07601267163  0.07631744002  0.07637005013  0.07652186739  0.07689227253  0.07715875484  0.07736387789  0.07752664555  0.07765687705 | 0.155060153  0.1555697434  0.1556433609  0.1558742069  0.1565048297  0.1569291767  0.157272474  0.1575467893  0.1577661352 | 0  0.9967698847  0  0  0  9.967698847  0  0.9967698847  53.82557377 | 0  0  0  0  0  7.890328346  0  1.434605154  7.173025769 | 0  0  0  0  0  8.580497882  0.780045262  0.780045262  54.60316834 | 0  0  0  0  0  10.19904203  0  0.9271856394  8.344670755 | 0  3.875920454  1.937960227  0  0  0  0  0.9689801135  496.1178181 | 0.9729249532  0  0.9729249532  0  0  0.9729249532  0.9729249532  1.945849906  17.51264916 | 2.246828371  3.744713952  0  0  0  0  0.7489427904  0.7489427904  485.3149282 | 3.695037168  0  0  0  0  0  1.231679056  0  21.55438348 | 2.788529585  0  2.788529585  1.394264792  2.788529585  147.792068  2.788529585  16.73117751  12.54838313 | 0  2.781693144  0  4.172539716  4.172539716  139.0846572  2.781693144  6.95423286  15.29931229 | 0  4.14083481  4.14083481  8.281669619  4.14083481  186.3375664  4.14083481  0  0 | 0.4804813169  1.441443951  2.882887901  5.285294486  6.726738436  127.327549  0.9609626337  8.648663704  14.41443951 |
| 13937 ENSG00000138207 17.37100479 2.709951842 1.536136377 1.764134932 0.07770926453 0.1578272542 5.980619308 0.7173025769 6.240362096 1.854371279 0 0 0 0 32.06809023 61.19724917 33.12667848 67.26738436 | | | | | | | | | | | | | | | | | | | |
| 13942 | ENSG00000272282 | 2.682998326 | 2.01197634 | 1.141128317 | 1.763146449 | 0.07787579284 | 0.15810875 | 1.993539769 | 0 | 1.560090524 | 0 | 2.906940341 | 0.9729249532 | 0 | 1.847518584 | 4.182794377 | 8.345079432 | 4.14083481 | 6.246257119 |

| 13944  13945  13954  13960  13962  13974  13994  14014 | ENSG00000237115 ENSG00000223601 ENSG00000167755 ENSG00000235947 ENSG00000225792 ENSG00000157782 ENSG00000230538 ENSG00000132185 | 1.964084071  0.9485103587  2.330628851  1.441748506  2.231048857  0.9282799357  0.9000379483  1.399145064 | 2.636418812  3.215639979  4.511825403  3.737316392  2.920147076  3.243629418  2.389813881  3.788961415 | 1.495445017  1.824178901  2.563369092  2.12464712  1.660409443  1.846092423  1.363232233  2.165888421 | 1.762966061  1.762787618  1.760115395  1.759029232  1.75869096  1.757024392  1.753049718  1.749379783 | 0.07790621389  0.0779363166  0.07838824295  0.07857254359  0.07863001374  0.07891365168  0.07959347339  0.0802253932 | 0.1581478263  0.1581974888  0.1590341767  0.1593176518  0.1593999262  0.1598489927  0.1609956308  0.1620334363 | 0  0  0  0  0  0  0  0 | 0.7173025769  0  0  0  1.434605154  0  0  0 | 0  0  0  0  0  0  0.780045262  0 | 0.9271856394  0  0  0  0  0  0  0 | 0  0  0  0  0  0  0.9689801135  0.9689801135 | 0  0.9729249532  0  0  0  0.9729249532  0.9729249532  0 | 0.7489427904  0  0  0  0  0.7489427904  0.7489427904  0.7489427904 | 0.615839528  0.615839528  0  0  2.463358112  0  1.231679056  0 | 2.788529585  2.788529585  9.759853547  4.182794377  8.365588755  4.182794377  1.394264792  0 | 8.345079432  5.563386288  1.390846572  4.172539716  5.563386288  1.390846572  2.781693144  8.345079432 | 4.14083481  0  0  4.14083481  4.14083481  0  0  0 | 5.285294486  1.441443951  16.81684609  4.804813169  4.804813169  3.843850535  1.921925267  6.726738436 |
| --- | --- | --- | --- | --- | --- | --- | --- | --- | --- | --- | --- | --- | --- | --- | --- | --- | --- | --- | --- |
| 14015 ENSG00000175946 3.987359479 2.009825444 1.148884045 1.749371882 0.08022675791 0.1620334363 0 2.869210308 1.560090524 0.9271856394 16.47266193 0 14.22991302 1.847518584 1.394264792 2.781693144 0 5.765775802 | | | | | | | | | | | | | | | | | | | |
| 14019  14029  14031  14032  14040  14043  14047  14051  14066 | ENSG00000100987 ENSG00000225889 ENSG00000260306 ENSG00000277109 ENSG00000276231 ENSG00000188089 ENSG00000214429 ENSG00000197705 ENSG00000132692 | 0.8199450291  0.92356965  1.963509278  0.9825306175  2.272013838  1.315604532  0.8179406592  1.551078715  29.25914527 | 3.104688347  2.453304414  3.350108453  2.636599933  4.464716899  3.69355721  3.145630109  3.76927188  2.036551784 | 1.775350568  1.403761933  1.917582476  1.509249409  2.556960493  2.116636341  1.803634925  2.161465233  1.169756732 | 1.748774807  1.747664156  1.747047908  1.746961051  1.7461032  1.745012659  1.744050343  1.74385034  1.741004543 | 0.08032995318  0.08052219892  0.08062902821  0.0806440946  0.08079302091  0.08098266545  0.08115031182  0.08118518995  0.08168278225 | 0.1621955671  0.1624678425  0.1626602005  0.1626674084  0.162890894  0.163234019  0.1635253596  0.1635733838  0.1643760013 | 0  0  0  0  0  0  0  0  17.94185792 | 0  0.7173025769  0  0.7173025769  0  0  0  0  6.455723192 | 0  0  0.780045262  0  0  0  0  0  10.92063367 | 0  0  0  0  0  0  0  0  2.781556918 | 3.875920454  0.9689801135  0  1.937960227  0  0  0.9689801135  0  4.844900568 | 0  0.9729249532  0  1.945849906  0  0  0  0  0 | 2.995771162  0.7489427904  0  1.497885581  0  0.7489427904  2.995771162  0  8.987313485 | 0.615839528  0.615839528  0  1.847518584  0  0  0.615839528  0  0 | 0  1.394264792  4.182794377  0  1.394264792  6.971323962  0  5.57705917  51.58779732 | 1.390846572  2.781693144  6.95423286  0  15.29931229  2.781693144  1.390846572  1.390846572  89.01418061 | 0  0  8.281669619  0  0  0  0  8.281669619  53.83085253 | 0.9609626337  2.882887901  3.363369218  3.843850535  10.57058897  5.285294486  3.843850535  3.363369218  104.7449271 |
| 14077 ENSG00000273032 2.245895312 2.607621953 1.499352365 1.739165532 0.08200565048 0.1648967779 0.9967698847 0 0.780045262 0 1.937960227 0.9729249532 0 0 1.394264792 5.563386288 12.42250443 2.882887901 | | | | | | | | | | | | | | | | | | | |
| 14078  14081  14083  14084  14088  14092  14094  14101  14104 | ENSG00000100206 ENSG00000243709 ENSG00000276598 ENSG00000174403 ENSG00000258595 ENSG00000141753 ENSG00000250159 ENSG00000180251 ENSG00000281128 | 17.01439345  1.261266282  2.101077971  1.534193218  1.347198797  4669.688621  2.634083331  2.954269045  1.799231738 | 2.207728403  2.908028678  2.059088991  3.870744576  2.904731559  2.247002368  3.088995922  3.231790434  3.029488127 | 1.269492983  1.672366434  1.184278748  2.227124829  1.671832516  1.293786716  1.778972072  1.863269635  1.747138559 | 1.7390631  1.738870512  1.738686095  1.738000729  1.737453681  1.736764137  1.736393713  1.734472764  1.733971305 | 0.08202366435  0.08205754219  0.08208999315  0.08221068537  0.08230712304  0.08242881172  0.08249424313  0.08283423417  0.08292317473 | 0.1649212845  0.1649542496  0.1649960482  0.1652269  0.1653737525  0.1655712422  0.1656791575  0.1662794009  0.1664225315 | 6.977389193  0  0  0  0  1337.665185  0.9967698847  0  0 | 5.021118038  0  0  0  0  1392.284302  0.7173025769  0  0 | 2.340135786  0.780045262  0  0  0  1267.573551  0  0.780045262  0 | 5.563113837  0  2.781556918  0  0.9271856394  1342.564806  0  0.9271856394  0.9271856394 | 0  5.813880681  0.9689801135  0  0  12567.67207  0  0  0 | 0.9729249532  0.9729249532  0.9729249532  0  1.945849906  13404.96001  0  0  0 | 0.7489427904  5.991542324  0.7489427904  0  0.7489427904  11694.74167  0  0  0.7489427904 | 0  0.615839528  4.310876696  0  1.847518584  12673.36165  0  0  0 | 36.2508846  0  4.182794377  2.788529585  0  73.896034  6.971323962  1.394264792  2.788529585 | 63.97894232  0  2.781693144  1.390846572  9.735926004  80.66910118  6.95423286  6.95423286  2.781693144 | 28.98584367  0  4.14083481  4.14083481  0  107.6617051  8.281669619  12.42250443  12.42250443 | 53.33342617  0.9609626337  4.324331852  10.09010765  0.9609626337  93.21337547  7.68770107  12.97299556  1.921925267 |
| 14105 ENSG00000269058 1.246516549 2.861506331 1.650320842 1.733909103 0.0829342124 0.1664328831 0.9967698847 0 0 0 0.9689801135 2.91877486 0 0.615839528 0 4.172539716 0 5.285294486 | | | | | | | | | | | | | | | | | | | |
| 14108  14113  14119  14123  14124  14125  14133  14136  14146 | ENSG00000247324 ENSG00000226856 ENSG00000173320 ENSG00000274286 ENSG00000184302 ENSG00000227487 ENSG00000132703 ENSG00000171847 ENSG00000263644 | 0.9838878461  1.865229787  42.10373725  1.405924844  1.405924844  1.405639993  2.198723591  3.292036154  1.399885504 | 3.02695117  2.125519163  2.106127643  3.706693036  3.706693036  3.706407362  4.427437255  2.389866762  2.199666879 | 1.746795417  1.227025077  1.216379976  2.141231255  2.141231255  2.141186189  2.560410685  1.382313069  1.273070351 | 1.732859578  1.732254054  1.731471813  1.731103555  1.731103555  1.731006571  1.729190274  1.728889653  1.72784393 | 0.08312063033  0.08322833866  0.08336764802  0.08343329651  0.08343329651  0.08345059248  0.08377504647  0.08382884633  0.08401620901 | 0.166771517  0.1669284599  0.1671368117  0.1672092106  0.1672092106  0.1672201947  0.1677871977  0.167859244  0.1681155671 | 0  0.9967698847  11.96123862  0  0  0  0  0.9967698847  0 | 0  0.7173025769  12.19414381  0  0  0  0  0.7173025769  0.7173025769 | 0  0.780045262  12.48072419  0  0  0  0  0.780045262  0 | 0  0  15.76215587  0  0  0  0  0.9271856394  0.9271856394 | 1.937960227  2.906940341  0.9689801135  0  0  0  14.5347017  0.9689801135  2.906940341 | 0  0.9729249532  0.9729249532  0  0  0  0  0  0.9729249532 | 1.497885581  4.493656743  2.246828371  0  0  0  11.23414186  0  1.497885581 | 0  0.615839528  0  0  0  0  0.615839528  0  1.847518584 | 2.788529585  0  83.65588755  4.182794377  4.182794377  2.788529585  0  8.365588755  5.57705917 | 0  4.172539716  129.3487312  2.781693144  2.781693144  4.172539716  0  12.51761915  1.390846572 | 4.14083481  0  103.5208702  4.14083481  4.14083481  4.14083481  0  4.14083481  0 | 1.441443951  6.726738436  132.1323621  5.765775802  5.765775802  5.765775802  0  10.09010765  0.9609626337 |
| 14148 ENSG00000213590 2.051759496 2.088592672 1.209109896 1.727380347 0.08409937786 0.1682581983 0 0.7173025769 1.560090524 0 0 0.9729249532 1.497885581 1.231679056 4.182794377 6.95423286 4.14083481 3.363369218 | | | | | | | | | | | | | | | | | | | |
| 14162  14163  14165  14166  14171  14172  14179  14186  14187 | ENSG00000130383 ENSG00000254311 ENSG00000254859 ENSG00000237863 ENSG00000273355 ENSG00000007062 ENSG00000187808 ENSG00000225214 ENSG00000281103 | 1.397491948  1.780274101  4.404722264  2.219546202  1.662119859  1.754983455  1.586388664  0.7528286219  1.208229683 | 3.667605809  2.094899489  2.08219298  4.35552709  2.475812099  3.306688212  2.526579134  3.020798071  3.603888632 | 2.127714762  1.215398675  1.208066423  2.527646902  1.43691299  1.919698322  1.467504852  1.755834411  2.094727084 | 1.72373002  1.72363154  1.723574912  1.723154878  1.723007667  1.72250409  1.721683666  1.720434486  1.720457362 | 0.08475659306  0.08477438099  0.0847846108  0.08486052092  0.08488713855  0.08497824249  0.08512683777  0.0853534929  0.0853493378 | 0.1694054599  0.1694171542  0.1694255696  0.1695413541  0.1695634669  0.1697194564  0.1699414818  0.1702978762  0.1702978762 | 0  0  0  0  0  0  0  0  0 | 0  2.151907731  1.434605154  0  0  0.7173025769  0.7173025769  0  0 | 0  0  3.120181048  0  0  0  0.780045262  0  0 | 0  0  0.9271856394  0  1.854371279  0  0  0  0 | 0  1.937960227  0  0  4.844900568  0  2.906940341  0.9689801135  7.751840908 | 0  6.810474673  0  0  2.91877486  0  4.864624766  3.891699813  2.91877486 | 0  2.246828371  5.242599533  0  6.740485114  0.7489427904  3.744713952  0.7489427904  0.7489427904 | 0  3.07919764  1.847518584  0  1.231679056  0  5.542555752  2.463358112  3.07919764 | 4.182794377  1.394264792  2.788529585  0  1.394264792  8.365588755  0  0  0 | 5.563386288  2.781693144  20.86269858  5.563386288  0  8.345079432  0  0  0 | 4.14083481  0  4.14083481  12.42250443  0  0  0  0  0 | 2.882887901  0.9609626337  12.49251424  8.648663704  0.9609626337  2.882887901  0.4804813169  0.9609626337  0 |
| 14190 ENSG00000254019 4.068725799 2.055758113 1.195839281 1.719092312 0.0855975645 0.1707487428 0 1.434605154 2.340135786 0.9271856394 0.9689801135 0.9729249532 0.7489427904 0 4.182794377 12.51761915 16.56333924 8.168182387 | | | | | | | | | | | | | | | | | | | |
| 14200  14204  14215  14217  14218  14220  14222  14225  14227 | ENSG00000273171 ENSG00000277624 ENSG00000228549 ENSG00000260585 ENSG00000223495 ENSG00000139515 ENSG00000270491 ENSG00000204894 ENSG00000074706 | 1.015075895  1.21968367  1.089572098  1.224147522  1.44110015  2.197439588  1.857529678  1.134226436  0.7654455373 | 3.400086993  2.593773342  2.765669237  3.592206135  3.615423215  4.399046606  2.020733349  3.531025383  3.071964335 | 1.97924283  1.51068204  1.612829983  2.09476785  2.108546101  2.565658482  1.178842166  2.060254113  1.792786537 | 1.717872584  1.716955172  1.714792796  1.714846891  1.714652202  1.71458775  1.714167858  1.713878575  1.713513724 | 0.08581985856  0.08598736297  0.08638322275  0.08637330184  0.0864090119  0.08642083619  0.08649790219  0.08655102858  0.08661807078 | 0.1710783753  0.1713572442  0.1719894624  0.1719894624  0.1720158584  0.1720272988  0.1721564913  0.1722258991  0.172335075 | 0  0.9967698847  0  0  0  0  0.9967698847  0  0 | 0  0  0.7173025769  0  0  0  0  0  0 | 0  0  0  0  0  0  0.780045262  0  0 | 0  0  0  0  0  0  0.9271856394  0  0 | 3.875920454  0.9689801135  0.9689801135  0  0  0  2.906940341  5.813880681  0.9689801135 | 1.945849906  1.945849906  0  0  1.945849906  0  5.837549719  0.9729249532  0.9729249532 | 2.995771162  1.497885581  1.497885581  0  0  0  4.493656743  3.744713952  3.744713952 | 0  3.695037168  3.695037168  0.615839528  0  0  3.07919764  3.07919764  0.615839528 | 0  0  0  5.57705917  4.182794377  0  2.788529585  0  0 | 0  1.390846572  1.390846572  4.172539716  0  9.735926004  0  0  0 | 0  4.14083481  0  0  8.281669619  4.14083481  0  0  0 | 3.363369218  0  4.804813169  4.324331852  2.882887901  12.49251424  0.4804813169  0  2.882887901 |
| 14228 ENSG00000101098 4.426428577 3.482069163 2.032575207 1.71313177 0.08668830041 0.1724626814 0 2.151907731 0 0 0 0 0 0 1.394264792 22.25354515 12.42250443 14.89492082 | | | | | | | | | | | | | | | | | | | |
| 14233  14246  14249  14256  14258  14260  14264  14265  14267 | ENSG00000242221 ENSG00000166265 ENSG00000280025 ENSG00000181617 ENSG00000113946 ENSG00000213023 ENSG00000123610 ENSG00000170231 ENSG00000168124 | 2.552169711  1.397207096  1.737269555  1.365884735  1.299427888  2.34677197  124.6688638  1.337183031  3.137376368 | 2.123558332  3.668693738  3.898270057  3.654799045  2.60740659  3.743256471  2.689674643  2.229806375  2.80690821 | 1.240442064  2.144546218  2.279611  2.13807729  1.525775561  2.190810002  1.574703774  1.305474409  1.644015725 | 1.711936731  1.710708637  1.710059329  1.70938584  1.708905724  1.708617574  1.708051183  1.708042961  1.70734876 | 0.08690832876  0.08713491251  0.08725490255  0.08737950209  0.08746841403  0.08752181115  0.08762684588  0.08762837124  0.08775724814 | 0.1728396792  0.1731321658  0.173340408  0.1734963655  0.1736485431  0.1737301814  0.1738807344  0.1738807344  0.1741130175 | 0.9967698847  0  0  0  0.9967698847  0  27.90955677  0  0 | 1.434605154  0  0  0  0  0  25.82289277  0  0.7173025769 | 0  0  0  0  0  0  24.18140312  1.560090524  0.780045262 | 0.9271856394  0  0  0  0  0.9271856394  29.66994046  0  0.9271856394 | 7.751840908  0  0  0  2.906940341  0  2.906940341  4.844900568  0 | 3.891699813  0  0.9729249532  0  0  0  0  0.9729249532  0 | 9.736256276  0  0  0  1.497885581  0  5.242599533  1.497885581  0 | 4.926716224  0  0  0  0  0  0  2.463358112  0 | 0  2.788529585  6.971323962  4.182794377  2.788529585  9.759853547  269.0931049  1.394264792  4.182794377 | 0  6.95423286  0  2.781693144  2.781693144  8.345079432  485.4054537  1.390846572  8.345079432 | 0  4.14083481  12.42250443  4.14083481  4.14083481  0  190.4784012  0  8.281669619 | 0.9609626337  2.882887901  0.4804813169  5.285294486  0.4804813169  9.129145021  435.3160731  1.921925267  14.41443951 |
| 14270 ENSG00000231512 1.604261844 2.184331779 1.279739537 1.706856525 0.0878487231 0.1742568995 1.993539769 0 0 0 0.9689801135 2.91877486 2.995771162 0.615839528 1.394264792 2.781693144 4.14083481 1.441443951 | | | | | | | | | | | | | | | | | | | |
| 14283 | ENSG00000123901 | 0.9501781489 | 2.511837354 | 1.473004757 | 1.705247279 | 0.08814831571 | 0.1746920272 | 0 | 0.7173025769 | 0 | 0 | 3.875920454 | 0.9729249532 | 1.497885581 | 2.463358112 | 1.394264792 | 0 | 0 | 0.4804813169 |

| 14284  14290  14299  14305  14306  14314  14322  14335 | ENSG00000184368 ENSG00000170074 ENSG00000197599 ENSG00000234638 ENSG00000260086 ENSG00000135914 ENSG00000156140 ENSG00000128713 | 18.02137626  1.253027532  1.063582668  1.478861688  2.054254865  0.794138031  6.952037231  1.436962354 | 2.104866574  2.87578154  2.658392255  3.701391604  2.145280775  3.043254976  2.46105707  3.723766134 | 1.23442176  1.687290896  1.560730516  2.173890596  1.259946013  1.788441079  1.446782752  2.191389944 | 1.70514377  1.704378034  1.703299979  1.702657719  1.702676743  1.701624399  1.701055024  1.699271344 | 0.08816761405  0.08831048502  0.08851194455  0.08863214165  0.08862857945  0.08882580066  0.08893265525  0.08926806935 | 0.17471804  0.1749154425  0.1752163859  0.1753684749  0.1753684749  0.1756288847  0.1757664949  0.1762330145 | 4.983849424  0  0  0  0  0  0  0 | 7.890328346  0.7173025769  0  0  0.7173025769  0  2.151907731  0 | 4.680271572  0  0.780045262  0  0.780045262  0  0.780045262  0 | 4.635928197  0  0  0  0.9271856394  0  3.708742558  0 | 1.937960227  0  0  0  0.9689801135  0  0  0 | 0.9729249532  0.9729249532  2.91877486  0  0  0.9729249532  1.945849906  0 | 0  0  3.744713952  0  0.7489427904  1.497885581  0  0 | 0  0.615839528  0.615839528  0  1.231679056  0  0  0 | 68.31897483  4.182794377  0  2.788529585  5.57705917  1.394264792  25.09676626  1.394264792 | 26.42608487  2.781693144  2.781693144  1.390846572  1.390846572  2.781693144  5.563386288  8.345079432 | 57.97168734  0  0  8.281669619  4.14083481  0  33.12667848  4.14083481 | 38.43850535  5.765775802  1.921925267  5.285294486  8.168182387  2.882887901  11.05107029  3.363369218 |
| --- | --- | --- | --- | --- | --- | --- | --- | --- | --- | --- | --- | --- | --- | --- | --- | --- | --- | --- | --- |
| 14360 ENSG00000100739 5.440044579 2.986282772 1.760001385 1.696750239 0.08974389005 0.1769004562 0 2.869210308 0.780045262 0 0 0 0 0 6.971323962 12.51761915 24.84500886 17.29732741 | | | | | | | | | | | | | | | | | | | |
| 14362  14366  14369  14370  14375  14388  14394  14402  14403 | ENSG00000226330 ENSG00000283839 ENSG00000124657 ENSG00000200788 ENSG00000120645 ENSG00000232358 ENSG00000265962 ENSG00000154856 ENSG00000250711 | 1.163727694  1.005068091  1.19000074  1.086600827  1.35773669  21.54892065  1.130244767  1.625852689  0.8643573221 | 3.571548557  2.616870255  2.731257183  3.16222953  3.613819956  2.334951756  3.539727365  3.213529116  2.335044294 | 2.105250973  1.543010764  1.610700395  1.86487768  2.132051099  1.379006995  2.091698543  1.899639646  1.380432895 | 1.696495383  1.695950744  1.695695359  1.695676646  1.694996878  1.693212409  1.692274146  1.691651953  1.691530463 | 0.08979210375  0.08989520847  0.08994358798  0.08994713363  0.09007601312  0.09041504352  0.09059371505  0.09071235457  0.09073553467 | 0.1769708459  0.1771009148  0.1771653722  0.1771653722  0.1773385091  0.1778765792  0.1781537931  0.1782880092  0.1783211862 | 0  0  0.9967698847  0  0  5.980619308  0  0  0 | 0  0  0  0  0  7.173025769  0  0.7173025769  0.7173025769 | 0  0.780045262  0  0  0  5.460316834  0  0  0 | 0  0  0  0  0  4.635928197  0  0  0 | 2.906940341  0.9689801135  2.906940341  0  0  0  1.937960227  0  1.937960227 | 2.91877486  3.891699813  0  0.9729249532  0  0.9729249532  0.9729249532  0.9729249532  0.9729249532 | 0.7489427904  2.995771162  0.7489427904  0  0  0  4.493656743  0  2.246828371 | 7.390074335  2.463358112  1.231679056  0  0  0  6.15839528  0  1.231679056 | 0  0  0  4.182794377  5.57705917  33.46235502  0  6.971323962  1.394264792 | 0  0  6.95423286  2.781693144  4.172539716  66.76063546  0  5.563386288  1.390846572 | 0  0  0  4.14083481  4.14083481  41.4083481  0  0  0 | 0  0.9609626337  1.441443951  0.9609626337  2.402406584  92.73289416  0  5.285294486  0.4804813169 |
| 14407 ENSG00000229677 1.4955827 2.291270183 1.355262882 1.690646304 0.09090437537 0.1786034045 1.993539769 0 0 0 0.9689801135 0.9729249532 1.497885581 1.231679056 1.394264792 5.563386288 0 4.324331852 | | | | | | | | | | | | | | | | | | | |
| 14408  14426  14427  14431  14435  14438  14439  14445  14451 | ENSG00000180178 ENSG00000169783 ENSG00000130768 ENSG00000219758 ENSG00000177875 ENSG00000141052 ENSG00000251455 ENSG00000228522 ENSG00000064218 | 1.115022192  2.698187368  13.83040806  1.876432792  2.374421887  1.470998495  1.25156374  1.042838476  4.541322567 | 3.454872656  3.072959305  2.283792173  3.247200654  2.836676863  3.654890187  2.158753979  3.413036478  5.440351653 | 2.043680556  1.821784196  1.354183426  1.926682219  1.683522428  2.169485362  1.281511549  2.027515629  3.233791382 | 1.69051501  1.686785576  1.686471809  1.685384658  1.684965294  1.684680731  1.68453728  1.6833589  1.682344657 | 0.09092946904  0.09164459001  0.09170496045  0.09191438162  0.09199526761  0.09205018605  0.09207788104  0.09230563456  0.09250202629 | 0.1786403075  0.1798311815  0.179926569  0.1802874705  0.1803961237  0.1804663088  0.1804956025  0.1808794248  0.1811890081 | 0  0.9967698847  7.974159078  0  0.9967698847  0  0  0  0 | 0  0  1.434605154  0.7173025769  0  0  0.7173025769  0  0 | 0  0  6.240362096  0  0  0  0  0  0 | 0  0.9271856394  0  0  0.9271856394  0  0.9271856394  0  0 | 0  0  72.67350851  0  0  0  1.937960227  5.813880681  0 | 0  0  3.891699813  0  0  0  1.945849906  3.891699813  0 | 0  0  63.66013719  2.995771162  0.7489427904  0  1.497885581  0  0 | 1.231679056  0  6.774234807  0.615839528  0  0  1.847518584  1.847518584  0 | 4.182794377  5.57705917  1.394264792  0  5.57705917  5.57705917  0  0  0 | 5.563386288  11.12677258  0  0  1.390846572  1.390846572  2.781693144  0  13.90846572 | 0  4.14083481  0  12.42250443  8.281669619  8.281669619  0  0  16.56333924 | 2.402406584  9.609626337  1.921925267  5.765775802  10.57058897  2.402406584  3.363369218  0.9609626337  24.02406584 |
| 14452 ENSG00000251187 2.950630575 3.387961698 2.013912681 1.682278348 0.0925148777 0.1811998077 0 0.7173025769 0.780045262 0 0 0 0 0 9.759853547 9.735926004 0 14.41443951 | | | | | | | | | | | | | | | | | | | |
| 14454  14458  14481  14483  14487  14509  14512  14519  14520 | ENSG00000173626 ENSG00000139304 ENSG00000234393 ENSG00000113263 ENSG00000214313 ENSG00000257017 ENSG00000227091 ENSG00000227120 ENSG00000235959 | 0.8328913294  1.696153236  1.144353233  1.475776838  0.8459011277  6.531160266  1.840575231  1.413581788  1.413581788 | 3.043118185  2.624466543  3.502368625  3.757015094  3.080935707  5.23903449  4.16350961  3.049231689  3.049231689 | 1.809032878  1.560741135  2.086723217  2.238914895  1.836731707  3.131835361  2.489681606  1.824228966  1.824228966 | 1.68217959  1.681551466  1.678405932  1.678051766  1.677401057  1.672832025  1.672306049  1.671518075  1.671518075 | 0.09253402064  0.09265584869  0.09326788042  0.09333699367  0.0934640828  0.0943603684  0.09446398747  0.09461939159  0.09461939159 | 0.1812140576  0.1813773481  0.1823106569  0.1824205581  0.1826185082  0.1840901915  0.1842630776  0.1844556817  0.1844556817 | 0  0  0  0  0  0.9967698847  0  0  0 | 0  1.434605154  0  0  0  0  0  0  0 | 0  0  0  0  0  0  0  0  0 | 0  0  0  0  0  0  0  0.9271856394  0.9271856394 | 0  3.875920454  0  0  0.9689801135  37.79022443  0  4.844900568  4.844900568 | 0.9729249532  8.756324579  3.891699813  0.9729249532  0  0  0  2.91877486  2.91877486 | 0  2.246828371  0  0  2.995771162  38.19608231  0  1.497885581  1.497885581 | 0.615839528  3.07919764  0  0  0.615839528  0  0  6.774234807  6.774234807 | 4.182794377  0  1.394264792  11.15411834  2.788529585  0  4.182794377  0  0 | 2.781693144  0  5.563386288  0  2.781693144  1.390846572  9.735926004  0  0 | 0  0  0  4.14083481  0  0  0  0  0 | 1.441443951  0.9609626337  2.882887901  1.441443951  0  0  8.168182387  0  0 |
| 14523 ENSG00000233932 2.159208058 2.22595871 1.33196033 1.671189945 0.09468416565 0.1845311204 0.9967698847 1.434605154 0 0 0 0.9729249532 0.7489427904 0.615839528 2.788529585 5.563386288 4.14083481 8.648663704 | | | | | | | | | | | | | | | | | | | |
| 14524  14538  14543  14546  14552  14558  14561  14562  14568 | ENSG00000103489 ENSG00000198092 ENSG00000099834 ENSG00000250927 ENSG00000185037 ENSG00000279379 ENSG00000280414 ENSG00000279062 ENSG00000233802 | 1.145923817  1.832427186  1.303555511  1.00219457  0.898295067  1.972775201  1.784003324  1.877067377  0.9960034715 | 3.522596208  4.148130431  2.738172687  3.379256962  2.446316105  4.274677131  3.174733057  2.685858978  3.063876314 | 2.107805529  2.485116168  1.641286651  2.026430835  1.468004867  2.56574754  1.905994261  1.612663935  1.840944246 | 1.671214996  1.669189748  1.668308632  1.667590575  1.666422339  1.666055239  1.665657196  1.665479657  1.664296092 | 0.09467921934  0.09507978115  0.09525447523  0.09539703053  0.09562932442  0.09570241262  0.09578171197  0.09581709891  0.09605327299 | 0.1845311204  0.1851169333  0.1854000671  0.1856392373  0.1859826121  0.1860799898  0.1861958065  0.1862518062  0.1866339886 | 0  0  0  0  0.9967698847  0  0  0.9967698847  0 | 0  0  0.7173025769  0  0  0  0  0  0 | 0  0  0  0  0  0  0  0.780045262  0 | 0  0  0  0  0  0  0.9271856394  0  0 | 0  0  0  2.906940341  0.9689801135  0  0  0  0 | 0  0  0  1.945849906  1.945849906  0  0  0.9729249532  0 | 1.497885581  0  0  2.246828371  1.497885581  0  0  2.246828371  0.7489427904 | 0  0  1.847518584  4.926716224  0.615839528  0  0  0  0 | 5.57705917  5.57705917  4.182794377  0  0  1.394264792  4.182794377  1.394264792  2.788529585 | 1.390846572  11.12677258  1.390846572  0  1.390846572  8.345079432  4.172539716  5.563386288  1.390846572 | 0  0  4.14083481  0  0  0  8.281669619  0  4.14083481 | 5.285294486  5.285294486  3.363369218  0  3.363369218  13.93395819  3.843850535  10.57058897  2.882887901 |
| 14570 ENSG00000087589 1.426612924 2.897581692 1.74120948 1.664120099 0.09608843127 0.1866766737 0 0.7173025769 0 0 0.9689801135 0 0 0 5.57705917 1.390846572 4.14083481 4.324331852 | | | | | | | | | | | | | | | | | | | |
| 14572  14574  14592  14600  14609  14611  14614  14618  14619 | ENSG00000226242 ENSG00000285471 ENSG00000235172 ENSG00000222012 ENSG00000203413 ENSG00000252699 ENSG00000237074 ENSG00000235097 ENSG00000259881 | 3.560564562  2.62430532  1.179494066  1.792671928  1.586219893  1.020481778  0.9312125693  1.108180824  1.326129477 | 3.129142573  2.979870719  2.749246331  4.115369209  3.139798397  2.589981941  3.296921167  2.719101091  3.603135532 | 1.880669834  1.791215009  1.656186415  2.481097766  1.895164603  1.56325707  1.9904994  1.641646403  2.175586394 | 1.66384472  1.663603032  1.659986041  1.658688853  1.65674179  1.656785689  1.656328642  1.656325677  1.656167524 | 0.09614346462  0.09619178559  0.09691726075  0.09717850665  0.09757169018  0.09756281145  0.09765528324  0.09765588326  0.097687898 | 0.1867579543  0.1868261756  0.1879901311  0.1884064938  0.189026368  0.189026368  0.1891363133  0.1891363133  0.1891479336 | 0  0.9967698847  0  0  0.9967698847  0  0  0  0 | 1.434605154  0  0.7173025769  0  0  0.7173025769  0  0.7173025769  0 | 0.780045262  0.780045262  0  0  0  0  0  0  0 | 0  0  0  0  0  0  0  0  0 | 0.9689801135  0  0.9689801135  0  0  0.9689801135  2.906940341  3.875920454  0 | 1.945849906  0  0  0  0  0.9729249532  0  0.9729249532  0 | 0  0  0.7489427904  0  0.7489427904  0  2.995771162  4.493656743  0 | 0  0  0  0  0  4.926716224  4.310876696  1.847518584  0 | 18.1254423  8.365588755  4.182794377  6.971323962  6.971323962  2.788529585  0  0  5.57705917 | 19.47185201  5.563386288  4.172539716  9.735926004  6.95423286  1.390846572  0  1.390846572  1.390846572 | 0  12.42250443  0  0  0  0  0  0  4.14083481 | 0  3.363369218  3.363369218  4.804813169  3.363369218  0.4804813169  0.9609626337  0  4.804813169 |
| 14627 ENSG00000273597 0.8503595298 2.732780486 1.651342284 1.654884341 0.0979479613 0.1895347958 0 0 0 0 0.9689801135 0.9729249532 2.246828371 0 1.394264792 0 4.14083481 0.4804813169 | | | | | | | | | | | | | | | | | | | |
| 14631  14640  14652  14675  14676  14680  14682  14686  14696 | ENSG00000281162 ENSG00000268182 ENSG00000254528 ENSG00000242600 ENSG00000164946 ENSG00000105707 ENSG00000238062 ENSG00000258904 ENSG00000133107 | 0.7982306215  1.371492457  1.836073931  1.133170723  1.250214011  1.041822417  1.137485591  1.689569347  11.34054956 | 2.624860088  2.503587817  4.155308559  3.475196346  3.60524368  3.420503703  3.490507263  2.514502855  2.260760698 | 1.586474338  1.514304385  2.515051815  2.106146428  2.185049439  2.073459659  2.116235725  1.524898686  1.372486006 | 1.654524139  1.653292324  1.652176124  1.650025991  1.649959774  1.649660117  1.649394357  1.64896388  1.647201275 | 0.09802106291  0.09827138444  0.09849865227  0.09893762031  0.09895116372  0.09901247179  0.09906687012  0.09915503468  0.09951668187 | 0.1896373595  0.1900086766  0.1902869674  0.1908366801  0.1908497983  0.1909238867  0.1909911713  0.1911127885  0.1916861453 | 0  0  0  0  0  0  0  0  3.987079539 | 0  0  0  0  0  0  0  0.7173025769  1.434605154 | 0  0.780045262  0  0  0  0  0  0  6.240362096 | 0  0  0  0  0  0  0  0.9271856394  0.9271856394 | 0.9689801135  1.937960227  0  0.9689801135  0.9689801135  4.844900568  1.937960227  0  0 | 0  0  0  0  0  0.9729249532  0  0.9729249532  0 | 1.497885581  0  0  0  0  5.242599533  0  0  0 | 0.615839528  1.231679056  0  0  0  0  0  1.231679056  0.615839528 | 1.394264792  1.394264792  2.788529585  4.182794377  9.759853547  0  1.394264792  5.57705917  39.03941419 | 0  1.390846572  12.51761915  5.563386288  1.390846572  0  6.95423286  5.563386288  22.25354515 | 4.14083481  8.281669619  0  0  0  0  0  0  41.4083481 | 0.9609626337  1.441443951  6.726738436  2.882887901  2.882887901  1.441443951  3.363369218  5.285294486  20.18021531 |
| 14697 ENSG00000268842 0.9476707564 3.2937192 1.999924321 1.646921918 0.09957409606 0.1917768499 0 0 0 0 0 0 1.497885581 1.847518584 4.182794377 0 0 3.843850535 | | | | | | | | | | | | | | | | | | | |
| 14699 | ENSG00000266989 | 1.900371672 | 2.413423731 | 1.465896647 | 1.646380553 | 0.09968543443 | 0.1919523007 | 0 | 0.7173025769 | 0 | 0.9271856394 | 0 | 0 | 1.497885581 | 0.615839528 | 6.971323962 | 1.390846572 | 8.281669619 | 2.402406584 |

| 14702  14733  14741  14747  14750  14753  14754  14755 | ENSG00000280269 ENSG00000139915 ENSG00000278330 ENSG00000169218 ENSG00000224271 ENSG00000089116 ENSG00000232118 ENSG00000071909 | 1.068801838  1.693006521  1.063126558  2.954676395  1.796033821  1.245764406  1.43346006  3.510086326 | 3.456370934  3.190947487  2.669830463  3.226723802  4.122620775  3.484147968  2.082857882  2.428684495 | 2.099779366  1.944191746  1.627573461  1.968475744  2.516767841  2.127955744  1.272135343  1.483412182 | 1.646063863  1.641272006  1.640374782  1.639199168  1.638061608  1.637321631  1.637292677  1.637228361 | 0.09975061148  0.1007409644  0.1009272653  0.101171787  0.1014088428  0.1015632836  0.1015693303  0.1015827633 | 0.1920514766  0.1935501078  0.1938028066  0.1941933006  0.1946087257  0.1948638649  0.1948638649  0.1948764281 | 0  0  0  0  0  0  0  0.9967698847 | 0  0.7173025769  0  0  0  0  0  0 | 0  0  0.780045262  1.560090524  0  0  0.780045262  1.560090524 | 0  0  0  0  0  0  0.9271856394  0.9271856394 | 1.937960227  0  5.813880681  0  0  0  0.9689801135  0 | 4.864624766  0  0.9729249532  0  0  0  0.9729249532  0 | 0  0  2.246828371  0  0  0  2.995771162  0 | 5.542555752  0  2.463358112  0  0  0  1.231679056  0.615839528 | 0  2.788529585  0  4.182794377  2.788529585  4.182794377  0  2.788529585 | 0  8.345079432  0  8.345079432  12.51761915  2.781693144  2.781693144  6.95423286 | 0  4.14083481  0  16.56333924  0  4.14083481  4.14083481  12.42250443 | 0.4804813169  4.324331852  0.4804813169  4.804813169  6.246257119  3.843850535  2.402406584  15.85588346 |
| --- | --- | --- | --- | --- | --- | --- | --- | --- | --- | --- | --- | --- | --- | --- | --- | --- | --- | --- | --- |
| 14766 ENSG00000214336 2.854112089 3.316293398 2.027994793 1.635257354 0.1019951095 0.19552171 0 1.434605154 0 0 0 0 0 0 1.394264792 11.12677258 8.281669619 12.01203292 | | | | | | | | | | | | | | | | | | | |
| 14773  14788  14792  14806  14822  14824  14835  14850  14851 | ENSG00000272892 ENSG00000140678 ENSG00000118733 ENSG00000236235 ENSG00000175664 ENSG00000284957 ENSG00000247925 ENSG00000171136 ENSG00000178162 | 1.08636292  5.481789009  1.286089367  0.9479769478  1.241547957  0.9251953472  20.38721601  1.966022641  1.402713066 | 2.60629043  2.090375199  3.546627769  3.294588216  3.458625888  2.370067439  2.04230288  3.514053122  3.599423431 | 1.594061396  1.28048685  2.172925933  2.021093631  2.124579481  1.455949267  1.255418876  2.162790087  2.215535707 | 1.63500003  1.632484707  1.632189904  1.630101726  1.627910803  1.627850292  1.626790005  1.624777709  1.624628942 | 0.1020490413  0.1025774176  0.102639487  0.103079999  0.1035438004  0.1035566337  0.1037817052  0.1042099324  0.1042416464 | 0.1955324012  0.1963454411  0.1964111221  0.1970675706  0.1977355887  0.1977384022  0.1980212301  0.1986374645  0.1986845359 | 0.9967698847  3.987079539  0  0  0  0  4.983849424  0  0 | 0  0.7173025769  0  0  0  0  7.890328346  0  0 | 0  2.340135786  0  0  0  0.780045262  5.460316834  0.780045262  0 | 0  0  0  0  0  0  8.344670755  0  0 | 0  0.9689801135  0  2.906940341  0  0  84.30126988  11.62776136  0 | 2.91877486  0.9729249532  0  3.891699813  0  1.945849906  24.32312383  0  0 | 0.7489427904  0.7489427904  0  1.497885581  0  0.7489427904  83.13264974  7.489427904  0 | 1.847518584  0  0  3.07919764  0  0.615839528  25.24942065  3.695037168  0 | 0  5.57705917  5.57705917  0  4.182794377  2.788529585  0  0  1.394264792 | 5.563386288  20.86269858  1.390846572  0  4.172539716  2.781693144  0  0  1.390846572 | 0  4.14083481  4.14083481  0  4.14083481  0  0  0  8.281669619 | 0.9609626337  25.46550979  4.324331852  0  2.402406584  1.441443951  0.9609626337  0  5.765775802 |
| 14854 ENSG00000235319 0.9247784646 2.350794766 1.447511905 1.624024478 0.1043705844 0.1988586582 0.9967698847 0 0 0 0 0.9729249532 0.7489427904 1.847518584 2.788529585 2.781693144 0 0.9609626337 | | | | | | | | | | | | | | | | | | | |
| 14855  14868  14884  14888  14899  14908  14916  14917  14932 | ENSG00000284176 ENSG00000271109 ENSG00000205879 ENSG00000104112 ENSG00000170152 ENSG00000224065 ENSG00000254724 ENSG00000181449 ENSG00000229587 | 2.346284802  0.8526591953  2.286861821  2.388898541  0.9037731935  2.363254011  1.681554199  2.200408411  2.773364529 | 2.087622036  2.36096987  2.922526092  2.917711805  2.440671568  2.382007141  4.029790489  2.880689476  2.01380455 | 1.285479119  1.455952757  1.804719693  1.802398752  1.508950976  1.473102236  2.493182461  1.782340441  1.24732546 | 1.624003071  1.621597856  1.61937951  1.618793733  1.617462467  1.617000561  1.616323937  1.616239754  1.614498071 | 0.1043751532  0.104889482  0.1053656328  0.1054916509  0.1057784912  0.1058781598  0.1060242942  0.1060424868  0.1064194348 | 0.1988586582  0.1996907235  0.2003766762  0.2005673476  0.2009218535  0.2010321433  0.2012016406  0.2012226742  0.2017351006 | 0.9967698847  0  0  0  0  0.9967698847  0  0.9967698847  0 | 0.7173025769  0  0.7173025769  0.7173025769  0.7173025769  1.434605154  0  0  1.434605154 | 0.780045262  0.780045262  0  0  0  0  0  0.780045262  0.780045262 | 0  0  0.9271856394  0.9271856394  0  0  0  0  0.9271856394 | 0  0.9689801135  0  0  0  0  0  0  0.9689801135 | 0  2.91877486  0  0  1.945849906  1.945849906  0  0  0 | 0.7489427904  0.7489427904  0  0  2.246828371  1.497885581  0  0  0 | 3.07919764  2.463358112  0  0  1.231679056  0.615839528  0  0.615839528  1.231679056 | 1.394264792  0  4.182794377  11.15411834  0  18.1254423  9.759853547  5.57705917  5.57705917 | 4.172539716  1.390846572  8.345079432  2.781693144  2.781693144  2.781693144  4.172539716  8.345079432  4.172539716 | 12.42250443  0  4.14083481  8.281669619  0  0  0  0  12.42250443 | 3.843850535  0.9609626337  9.129145021  4.804813169  1.921925267  0.9609626337  6.246257119  10.09010765  5.765775802 |
| 14933 ENSG00000160963 0.9804102331 2.986829556 1.850272937 1.614264304 0.1064701092 0.2018176463 0 0 0 0 0 0 0 0.615839528 1.394264792 4.172539716 4.14083481 1.441443951 | | | | | | | | | | | | | | | | | | | |
| 14934  14939  14942  14949  14956  14958  14970  14971  14980 | ENSG00000225025 ENSG00000236056 ENSG00000139874 ENSG00000229221 ENSG00000103449 ENSG00000282164 ENSG00000070886 ENSG00000168631 ENSG00000183784 | 1.00347937  2.044798784  1.749839628  1.085657468  102.327747  1.69272167  1.231457978  2.319217974  1.205439444 | 2.559622837  4.21083501  4.074117431  2.424026798  3.236736226  3.193310114  3.479573899  2.073356045  3.421123838 | 1.585744176  2.609181901  2.525072772  1.503064605  2.007691807  1.981059969  2.160831507  1.287607457  2.12585652 | 1.614146138  1.613852606  1.613465353  1.612722959  1.612167871  1.611919964  1.610293948  1.610239234  1.609291975 | 0.1064957317  0.1065594009  0.1066434446  0.1068047102  0.1069254147  0.1069793572  0.1073336998  0.1073456394  0.107552513 | 0.2018526974  0.2019057769  0.2020143824  0.2022352081  0.2023690017  0.2024440223  0.2029517506  0.2029607687  0.2032297352 | 0  0  0  0  25.916017  0  0  1.993539769  0 | 0  0  0  0.7173025769  11.47684123  0.7173025769  0  0.7173025769  0 | 0.780045262  0  0  0  17.94104103  0  0  0  0 | 0  0  0  0  6.490299476  0  0  0  0 | 0  0  0  0.9689801135  0  0  0  0  0 | 1.945849906  0  0  0  0  0  0  0.9729249532  0 | 0  0  0  2.246828371  0  0  0.7489427904  0.7489427904  0 | 3.695037168  0  0  3.07919764  0  0  0  0.615839528  0 | 2.788529585  11.15411834  13.94264792  1.394264792  239.8135443  1.394264792  0  4.182794377  2.788529585 | 1.390846572  0  4.172539716  0  307.3770924  9.735926004  5.563386288  6.95423286  4.172539716 | 0  12.42250443  0  4.14083481  302.2809411  4.14083481  4.14083481  8.281669619  4.14083481 | 1.441443951  0.9609626337  2.882887901  0.4804813169  316.6371878  4.324331852  4.324331852  3.363369218  3.363369218 |
| 14983 ENSG00000271687 3.859631521 2.148807128 1.335512478 1.608975703 0.1076216544 0.2033196657 0.9967698847 0.7173025769 0.780045262 1.854371279 0 0 0 0.615839528 12.54838313 6.95423286 16.56333924 5.285294486 | | | | | | | | | | | | | | | | | | | |
| 14988  14994  14995  14998  15021  15023  15026  15027  15031 | ENSG00000214872 ENSG00000275558 ENSG00000254144 ENSG00000168539 ENSG00000213608 ENSG00000135638 ENSG00000227582 ENSG00000177551 ENSG00000151952 | 1.732774242  2.848559951  2.848559951  1.173749548  1.491830535  1.086617982  15.05524318  0.9428831108  0.9594916698 | 2.226678803  2.752301448  2.752301448  3.381938695  2.392303748  2.677996154  2.256434804  2.371902877  2.982784136 | 1.384506862  1.712273553  1.712273553  2.104632142  1.492125112  1.670483602  1.408121342  1.480429865  1.862023528 | 1.608282967  1.607395876  1.607395876  1.606902521  1.603286299  1.603126275  1.602443437  1.60217173  1.601904644 | 0.1077732194  0.1079675541  0.1079675541  0.1080757533  0.1088714601  0.1089067781  0.1090575859  0.1091176397  0.1091766974 | 0.2035380804  0.203809909  0.203809909  0.203973348  0.2051604786  0.2051997111  0.2054428342  0.2055422846  0.2056231443 | 0  0  0  0  0  0  10.96446873  0.9967698847  0 | 0.7173025769  0  0  0  1.434605154  0.7173025769  1.434605154  0  0 | 0.780045262  2.340135786  2.340135786  0  0  0  3.120181048  0  0 | 0  0  0  0  0  0  1.854371279  0  0 | 0.9689801135  0  0  0  0  3.875920454  31.00736363  1.937960227  0 | 0.9729249532  0  0  0  1.945849906  1.945849906  35.99822327  0  0.9729249532 | 0  4.493656743  4.493656743  1.497885581  0.7489427904  4.493656743  53.17493812  0.7489427904  2.995771162 | 0.615839528  0.615839528  0.615839528  0  0.615839528  0.615839528  43.10876696  0.615839528  0.615839528 | 4.182794377  1.394264792  1.394264792  0  2.788529585  0  0  4.182794377  2.788529585 | 1.390846572  16.69015886  16.69015886  5.563386288  5.563386288  1.390846572  0  1.390846572  0 | 8.281669619  0  0  4.14083481  0  0  0  0  4.14083481 | 2.882887901  8.648663704  8.648663704  2.882887901  4.804813169  0  0  1.441443951  0 |
| 15034 ENSG00000198914 4.719935637 2.804606271 1.751581041 1.60118556 0.1093358261 0.2058383703 0 2.869210308 0.780045262 0 0 0 0 0 9.759853547 16.69015886 8.281669619 18.25829004 | | | | | | | | | | | | | | | | | | | |
| 15039  15047  15048  15055  15057  15064  15076  15077  15078 | ENSG00000228933 ENSG00000231165 ENSG00000171385 ENSG00000253214 ENSG00000283235 ENSG00000107018 ENSG00000174482 ENSG00000182107 ENSG00000247416 | 1.641514089  1.503610378  2.891963569  0.838018114  1.436619218  0.9461531349  5.598633432  1.817843766  1.943521068 | 3.993942415  3.5510156  2.254260161  2.207012638  3.038841397  2.933474835  2.285604476  2.452892298  2.482351783 | 2.495159654  2.219366823  1.409078571  1.380416359  1.900902822  1.835781281  1.431927245  1.536897652  1.555443332 | 1.600676096  1.600012924  1.599811542  1.598802147  1.598630588  1.597943538  1.596173608  1.596002372  1.59591271 | 0.1094486782  0.1095957163  0.1096403976  0.1098645719  0.109902709  0.1100555434  0.1104500384  0.1104882638  0.1105082835 | 0.2060013488  0.2061684287  0.2062387755  0.2065665726  0.2066086259  0.2067998017  0.2073758813  0.2074338923  0.207457718 | 0  0  0.9967698847  0.9967698847  0.9967698847  0  3.987079539  0.9967698847  0.9967698847 | 0  0  1.434605154  0  0  0  1.434605154  0.7173025769  0 | 0  0  0.780045262  0  0  0  0.780045262  0  0.780045262 | 0  0  0  0  0  0  0  0  0 | 0  0  1.937960227  0.9689801135  0  0  1.937960227  0  0 | 0  0  0  0.9729249532  1.945849906  0.9729249532  0  1.945849906  0 | 0  0  0  0.7489427904  0  0  2.246828371  2.246828371  1.497885581 | 0  0  0  1.231679056  0.615839528  0.615839528  0  0.615839528  0 | 9.759853547  2.788529585  9.759853547  1.394264792  0  4.182794377  0  9.759853547  2.788529585 | 4.172539716  1.390846572  5.563386288  2.781693144  6.95423286  0  16.69015886  1.390846572  4.172539716 | 0  12.42250443  4.14083481  0  0  4.14083481  16.56333924  4.14083481  8.281669619 | 5.765775802  1.441443951  10.09010765  0.9609626337  6.726738436  1.441443951  23.54358453  0  4.804813169 |
| 15085 ENSG00000253372 0.8311103892 2.814047312 1.764263407 1.595026741 0.1107062561 0.2077191626 0 0 0 0 0.9689801135 0 0.7489427904 1.231679056 0 0 4.14083481 2.882887901 | | | | | | | | | | | | | | | | | | | |
| 15094  15099  15102  15116  15118  15138  15157  15170  15192 | ENSG00000055955 ENSG00000274677 ENSG00000142303 ENSG00000110484 ENSG00000280890 ENSG00000277066 ENSG00000261305 ENSG00000235269 ENSG00000231937 | 17.98083411  1.309854488  1.637582492  0.7336320818  0.9144929587  1.12622365  0.9397462329  1.182720997  1.696710881 | 2.103686014  2.751081353  3.984778277  2.890602444  3.258967018  2.650351931  3.283255195  2.799668433  4.061185266 | 1.320378813  1.72789437  2.503897379  1.818161021  2.050281155  1.670254087  2.071094485  1.768775806  2.571285429 | 1.593244298  1.592158294  1.591430348  1.58984953  1.589522008  1.586795657  1.585275427  1.582828318  1.579437748 | 0.1111053972  0.1113491415  0.111512759  0.1118687264  0.1119425895  0.1125589328  0.1129037696  0.1134606005  0.1142356849 | 0.208357584  0.2087455328  0.2090107374  0.2094811444  0.2095943205  0.2104756  0.2108500429  0.2117083558  0.2128459252 | 6.977389193  0  0  0  0  0.9967698847  0  0  0 | 5.738420615  0.7173025769  0  0  0  0  0  0  0 | 6.240362096  0  0  0  0  0  0  0  0 | 3.708742558  0  0  0  0  0  0  0.9271856394  0 | 80.42534942  0.9689801135  0  1.937960227  0  0  4.844900568  2.906940341  0 | 13.62094935  0  0  0.9729249532  3.891699813  0.9729249532  0.9729249532  2.91877486  0 | 86.87736369  0  0  0.7489427904  0  1.497885581  2.995771162  3.744713952  0 | 11.70095103  0  0  0  1.847518584  3.07919764  2.463358112  3.695037168  0 | 0  1.394264792  11.15411834  4.182794377  0  5.57705917  0  0  1.394264792 | 0  4.172539716  4.172539716  0  1.390846572  1.390846572  0  0  6.95423286 | 0  4.14083481  0  0  0  0  0  0  0 | 0.4804813169  4.324331852  4.324331852  0.9609626337  3.843850535  0  0  0  12.01203292 |
| 15195 ENSG00000236304 1.165684186 3.35538261 2.12513395 1.578904055 0.1143580658 0.2130318796 0 0 0 0 0 0 0 0 4.182794377 2.781693144 4.14083481 2.882887901 | | | | | | | | | | | | | | | | | | | |
| 15201 | ENSG00000283743 | 0.8687363429 | 3.187768184 | 2.02016113 | 1.57797719 | 0.1145708501 | 0.2133440223 | 0 | 0 | 0 | 0 | 4.844900568 | 1.945849906 | 0 | 1.231679056 | 0 | 0 | 0 | 2.402406584 |

| 15202  15203  15211  15212  15223  15225  15230  15234 | ENSG00000275856 ENSG00000240128 ENSG00000260179 ENSG00000235529 ENSG00000273618 ENSG00000198670 ENSG00000144891 ENSG00000124479 | 0.8471228835  1.039460239  1.613418666  1.37171173  0.850584517  3.895843973  1.561149018  0.9587765878 | 2.646573332  3.084495075  2.18614851  2.258213597  2.817683057  2.128553719  3.919081308  3.313451352 | 1.677321911  1.955232403  1.386922799  1.43270282  1.78903207  1.351891818  2.490046141  2.105458758 | 1.577856531  1.577559307  1.576258254  1.576191214  1.57497627  1.574500038  1.573899071  1.573743175 | 0.1145985732  0.1146668871  0.1149662982  0.1149817427  0.1152619221  0.1153718928  0.1155107847  0.115546836 | 0.2133816086  0.2134947646  0.2139396513  0.2139468479  0.2143206967  0.2144848086  0.2146903  0.2146953353 | 0  0  0  0  0  1.993539769  0  0 | 0  0  0  0  0  1.434605154  0  0 | 0  0  0.780045262  0.780045262  0  0  0  0 | 0  0  0.9271856394  0.9271856394  0  0.9271856394  0  0 | 0  0  0.9689801135  2.906940341  0.9689801135  2.906940341  0  1.937960227 | 0  0  0.9729249532  4.864624766  1.945849906  0  0  3.891699813 | 0.7489427904  0.7489427904  0.7489427904  0.7489427904  0.7489427904  3.744713952  0  0.7489427904 | 0.615839528  0.615839528  2.463358112  4.310876696  0  0.615839528  0  4.926716224 | 2.788529585  5.57705917  5.57705917  0  0  13.94264792  8.365588755  0 | 1.390846572  1.390846572  2.781693144  0  0  0  5.563386288  0 | 4.14083481  4.14083481  4.14083481  0  4.14083481  20.70417405  0  0 | 0.4804813169  0  0  1.921925267  2.402406584  0.4804813169  4.804813169  0 |
| --- | --- | --- | --- | --- | --- | --- | --- | --- | --- | --- | --- | --- | --- | --- | --- | --- | --- | --- | --- |
| 15242 ENSG00000205832 1.402797125 2.985401697 1.897997077 1.572922179 0.1157368389 0.2149355046 0.9967698847 0 0 0 1.937960227 0 0.7489427904 0 0 8.345079432 0 4.804813169 | | | | | | | | | | | | | | | | | | | |
| 15247  15249  15251  15253  15255  15257  15269  15272  15287 | ENSG00000234323 ENSG00000225964 ENSG00000215796 ENSG00000215246 ENSG00000117971 ENSG00000235010 ENSG00000101282 ENSG00000069696 ENSG00000267408 | 0.8306764105  1.8923668  2.167979102  3.980464986  1.560351473  2.840904445  1.542291088  1.424602808  0.7679330649 | 2.6272125  4.053378181  2.726094542  2.297781982  3.030246714  3.116729531  3.030466944  2.011194915  2.939232962 | 1.671415571  2.579399163  1.734857757  1.46241363  1.928754913  1.984167046  1.931205794  1.281881498  1.876754314 | 1.571848765  1.571442776  1.571364874  1.571225771  1.571089563  1.570799967  1.569209741  1.568939811  1.566125592 | 0.1159856291  0.1160798366  0.1160979201  0.1161302161  0.1161618465  0.1162291198  0.1165990751  0.1166619643  0.1173192179 | 0.2153268983  0.2154735297  0.2154788358  0.2155018532  0.2155409522  0.2156361598  0.2161504818  0.2162279703  0.2172327979 | 0  0  0  2.990309654  0.9967698847  0  0  0  0 | 0  0  0.7173025769  0.7173025769  0  0  0  0  0 | 0  0  0.780045262  0.780045262  0  0  0.780045262  0.780045262  0 | 0  0  0  0  0  1.854371279  0  0.9271856394  0 | 0  0  2.906940341  0  0  0  0  1.937960227  0.9689801135 | 1.945849906  0  4.864624766  0  0  0  0  1.945849906  0.9729249532 | 0  0  0  1.497885581  0  0  0  2.246828371  0.7489427904 | 0.615839528  0  0  0  0  0  0  1.847518584  0 | 1.394264792  0  0  4.182794377  2.788529585  2.788529585  2.788529585  2.788529585  0 | 1.390846572  2.781693144  0  18.08100544  6.95423286  12.51761915  6.95423286  0  5.563386288 | 4.14083481  16.56333924  12.42250443  4.14083481  4.14083481  8.281669619  4.14083481  4.14083481  0 | 0.4804813169  3.363369218  4.324331852  15.37540214  3.843850535  8.648663704  3.843850535  0.4804813169  0.9609626337 |
| 15297 ENSG00000228626 0.9762377734 3.339735061 2.135840967 1.563662797 0.1178967789 0.218159523 0 0 0 0 1.937960227 0 0.7489427904 0 0 2.781693144 0 6.246257119 | | | | | | | | | | | | | | | | | | | |
| 15308  15317  15324  15341  15345  15380  15387  15390  15394 | ENSG00000198691 ENSG00000260458 ENSG00000248810 ENSG00000260516 ENSG00000112041 ENSG00000239605 ENSG00000277218 ENSG00000259417 ENSG00000019102 | 1.269231588  1.37540636  1.075648725  1.371751996  0.8266086857  3.167200012  0.72088197  1.205481947  1.679877169 | 2.875795212  2.998220739  3.402930479  2.194602681  2.331609301  2.022534066  2.864403135  2.051905985  2.040804655 | 1.84122881  1.92076824  2.181522369  1.409555703  1.497910837  1.302759452  1.846056089  1.322762746  1.315826494 | 1.561889102  1.560948727  1.559887961  1.556946403  1.556574159  1.552500013  1.55163386  1.551227529  1.5509679 | 0.1183141163  0.118535849  0.11878636  0.119483211  0.119571623  0.1205426296  0.1207498564  0.1208471672  0.1209093768 | 0.2187744563  0.2190556729  0.2194183442  0.220460972  0.2205665923  0.2218517343  0.2221320229  0.2222677008  0.2223243355 | 0.9967698847  0.9967698847  0  0.9967698847  0  1.993539769  0  0  0 | 0  0  0  0  0  0.7173025769  0  0  2.151907731 | 0  0  0  0  0.780045262  0.780045262  0  0.780045262  0 | 0  0  0  0.9271856394  0  0.9271856394  0  0.9271856394  0 | 5.813880681  1.937960227  0  0  0.9689801135  8.720821022  0  1.937960227  4.844900568 | 1.945849906  0  0  1.945849906  0.9729249532  8.756324579  0  3.891699813  4.864624766 | 5.242599533  0.7489427904  0.7489427904  2.246828371  2.246828371  7.489427904  0.7489427904  1.497885581  2.246828371 | 1.231679056  0  0  1.847518584  3.07919764  8.621753391  1.847518584  3.07919764  3.695037168 | 0  0  8.365588755  0  0  0  4.182794377  0  1.394264792 | 0  4.172539716  1.390846572  4.172539716  1.390846572  0  1.390846572  1.390846572  0 | 0  0  0  0  0  0  0  0  0 | 0  8.648663704  2.402406584  4.324331852  0.4804813169  0  0.4804813169  0.9609626337  0.9609626337 |
| 15404 ENSG00000176320 1.165114483 3.358291439 2.169056644 1.548272817 0.1215566263 0.2233693758 0 0 0 0 0 0 0 0 1.394264792 5.563386288 4.14083481 2.882887901 | | | | | | | | | | | | | | | | | | | |
| 15407  15409  15412  15414  15426  15432  15436  15441  15444 | ENSG00000244327 ENSG00000271225 ENSG00000124134 ENSG00000214212 ENSG00000159708 ENSG00000248968 ENSG00000204086 ENSG00000136883 ENSG00000113905 | 1.543350522  3.051996443  0.7079103089  2.833515433  1.002577144  1.308009486  2.496662159  1.358233162  1.480783946 | 2.833889901  2.068710431  2.855348901  2.061892409  3.335958812  2.927928195  2.044513437  2.195034756  3.839687268 | 1.831359226  1.337187396  1.846897897  1.334116622  2.162135591  1.899107348  1.326525561  1.425498276  2.494296597 | 1.547424372  1.547060971  1.546024231  1.545511371  1.542899911  1.541739175  1.54125446  1.539836837  1.539386805 | 0.121760948  0.1218485442  0.1220987159  0.1222226206  0.1228550624  0.1231369882  0.1232548677  0.1236001302  0.1237098935 | 0.2237012652  0.2238186172  0.2242490431  0.2244474827  0.2254333849  0.2258551125  0.2260204901  0.226565554  0.2267373896 | 0  0.9967698847  0  1.993539769  0  0  0.9967698847  0.9967698847  0 | 0  0.7173025769  0  0  0  0  0  0.7173025769  0 | 0.780045262  2.340135786  0  0.780045262  0  0.780045262  2.340135786  0  0 | 0  0  0  0.9271856394  0  0  0  0  0 | 0  1.937960227  0.9689801135  0  0  0  0  1.937960227  0 | 0  9.729249532  0  0.9729249532  0  0  2.91877486  0  0 | 0  7.489427904  2.246828371  0.7489427904  0.7489427904  0  0  0.7489427904  0 | 0.615839528  12.93263009  0.615839528  0  0  1.231679056  4.926716224  0.615839528  0 | 6.971323962  0  4.182794377  4.182794377  1.394264792  1.394264792  5.57705917  1.394264792  6.971323962 | 1.390846572  0  0  11.12677258  5.563386288  5.563386288  6.95423286  5.563386288  6.95423286 | 8.281669619  0  0  4.14083481  0  0  0  0  0 | 0.4804813169  0.4804813169  0.4804813169  9.129145021  4.324331852  6.726738436  6.246257119  4.324331852  3.843850535 |
| 15446 ENSG00000182487 1.363527504 2.560335082 1.663408804 1.53920977 0.1237530933 0.2267871978 0 0.7173025769 0 0 0 0 2.246828371 1.847518584 2.788529585 0 8.281669619 0.4804813169 | | | | | | | | | | | | | | | | | | | |
| 15447  15448  15455  15456  15468  15471  15482  15485  15501 | ENSG00000077092 ENSG00000158639 ENSG00000267478 ENSG00000140030 ENSG00000188133 ENSG00000250254 ENSG00000256980 ENSG00000224272 ENSG00000215351 | 8.241931339  0.8298982446  1.34813399  0.8884262722  1.236761806  0.9266820674  0.9323732011  0.8625296902  0.7103141274 | 2.081583854  3.131314531  2.069282544  3.206759001  3.444413138  2.455872768  2.48317427  2.756953209  2.822434161 | 1.35243918  2.034440306  1.345328346  2.085463244  2.24217133  1.598990709  1.619605516  1.798927083  1.843520672 | 1.539133061  1.539152818  1.53812454  1.53767227  1.536195335  1.535889329  1.53319697  1.532554174  1.531002177 | 0.1237718153  0.123766993  0.1240181668  0.124128767  0.1244904797  0.1245655256  0.1252273305  0.12538574  0.1257688547 | 0.2267921417  0.2267921417  0.2271406165  0.227328473  0.2278140366  0.2279054363  0.2289552265  0.229188507  0.22967061 | 0.9967698847  0  0  0  0  0.9967698847  0  0  0 | 5.021118038  0  1.434605154  0  0  0  0  0  0 | 1.560090524  0  0  0  0  0  0.780045262  0  0 | 2.781556918  0  0  0  0  0  0  0  0 | 0.9689801135  1.937960227  0  4.844900568  0  0.9689801135  0.9689801135  0.9689801135  0 | 0  1.945849906  0.9729249532  0.9729249532  0  0  4.864624766  0  0.9729249532 | 0  2.995771162  1.497885581  2.995771162  0  0.7489427904  2.246828371  1.497885581  0.7489427904 | 0  3.07919764  2.463358112  1.847518584  0  3.695037168  1.847518584  0  1.231679056 | 26.49103106  0  1.394264792  0  1.394264792  2.788529585  0  0  2.788529585 | 27.81693144  0  1.390846572  0  8.345079432  0  0  2.781693144  2.781693144 | 8.281669619  0  4.14083481  0  4.14083481  0  0  4.14083481  0 | 24.98502848  0  2.882887901  0  0.9609626337  1.921925267  0.4804813169  0.9609626337  0 |
| 15512 ENSG00000258088 0.9026160824 2.449538381 1.60206584 1.528987336 0.1262675835 0.2304106638 0 0 0.780045262 0 0.9689801135 3.891699813 2.246828371 2.463358112 0 0 0 0.4804813169 | | | | | | | | | | | | | | | | | | | |
| 15524  15534  15543  15547  15551  15557  15560  15567  15570 | ENSG00000226984 ENSG00000272843 ENSG00000201457 ENSG00000183690 ENSG00000225024 ENSG00000206675 ENSG00000172005 ENSG00000127324 ENSG00000235244 | 1.267847236  1.317331188  0.9831886289  1.260880039  1.044523695  1.120657306  1.51859727  0.8456657632  2.207885508 | 2.260288717  2.112292802  2.497373736  2.809610858  2.188178386  2.334663724  3.900166937  3.137275154  2.166159207 | 1.480044126  1.384760945  1.639335677  1.844892271  1.437383603  1.534313362  2.563608077  2.063534627  1.425189324 | 1.527176574  1.525384442  1.523405957  1.522913236  1.522334318  1.521634225  1.521358499  1.520340445  1.519909791 | 0.1267171099  0.1271632369  0.1276571726  0.127780414  0.1279253334  0.1281007569  0.1281698974  0.128425434  0.1285336494 | 0.2310522103  0.2317249129  0.2324817556  0.2326313608  0.232850266  0.2330796442  0.2331454994  0.2335202887  0.2336720283 | 0  0  0  0.9967698847  0  0  0  0  0.9967698847 | 0.7173025769  0  0  0  0  0  0  0  0 | 0  0  0.780045262  0  0.780045262  0  0  0  0 | 0  1.854371279  0  0  0  0.9271856394  0  0  1.854371279 | 0.9689801135  0.9689801135  0  0  0  0  0  1.937960227  0 | 0  0.9729249532  1.945849906  0  0.9729249532  0  0  4.864624766  0 | 0.7489427904  1.497885581  0  1.497885581  0.7489427904  0.7489427904  0.7489427904  1.497885581  2.246828371 | 1.231679056  0.615839528  0.615839528  0  1.231679056  0.615839528  0  1.847518584  1.231679056 | 1.394264792  4.182794377  4.182794377  6.971323962  2.788529585  4.182794377  0  0  4.182794377 | 1.390846572  1.390846572  1.390846572  2.781693144  1.390846572  1.390846572  8.345079432  0  9.735926004 | 8.281669619  0  0  0  4.14083481  4.14083481  0  0  0 | 0.4804813169  4.324331852  2.882887901  2.882887901  0.4804813169  1.441443951  9.129145021  0  6.246257119 |
| 15574 ENSG00000142405 2.090007942 2.92793793 1.927067056 1.519375219 0.128668076 0.2338563349 0 1.434605154 0 0 8.720821022 0 12.73202744 1.231679056 0 0 0 0.9609626337 | | | | | | | | | | | | | | | | | | | |
| 15577  15592  15597  15598  15602  15612  15618  15620  15627 | ENSG00000214402 ENSG00000258413 ENSG00000214376 ENSG00000255028 ENSG00000264365 ENSG00000265800 ENSG00000271461 ENSG00000279791 ENSG00000256292 | 1.598412314  0.8862927856  0.8154357889  0.8406614465  1.043241984  1.360046108  1.310268353  0.9632490421  0.721362181 | 3.87283949  2.393483494  2.697195517  3.144369557  2.241967612  2.175643884  3.362800698  3.27126701  2.845471717 | 2.550391685  1.578829771  1.780063697  2.075427682  1.480221794  1.437919021  2.223603581  2.164058572  1.883745726 | 1.518527336  1.515985788  1.515224158  1.515046553  1.514616  1.513050354  1.512320238  1.511635153  1.510539176 | 0.1288815137  0.1295229461  0.1297156472  0.1297606152  0.1298696779  0.1302668688  0.1304524149  0.1306267038  0.1309059008 | 0.234199148  0.2351341076  0.2354126505  0.2354791623  0.2356166583  0.2361858819  0.2364430409  0.236716996  0.2371166844 | 0  0  0  0  0  0  0  0  0 | 0  0.7173025769  0  0  0  0  0  0  0 | 0  0  0  0  0.780045262  1.560090524  0  0  0 | 0  0  0  0  0  0  0  0  0 | 0  0  0.9689801135  2.906940341  0  0.9689801135  0  0  0.9689801135 | 0  1.945849906  1.945849906  0.9729249532  0  2.91877486  0  0.9729249532  0 | 0  2.246828371  1.497885581  3.744713952  0.7489427904  0  0  0.7489427904  1.497885581 | 0  2.463358112  1.231679056  2.463358112  1.231679056  2.463358112  0  0  0.615839528 | 0  0  0  0  1.394264792  5.57705917  2.788529585  0  4.182794377 | 4.172539716  2.781693144  0  0  2.781693144  1.390846572  4.172539716  6.95423286  1.390846572 | 8.281669619  0  4.14083481  0  4.14083481  0  8.281669619  0  0 | 6.726738436  0.4804813169  0  0  1.441443951  1.441443951  0.4804813169  2.882887901  0 |
| 15631 ENSG00000235631 1.25322586 2.110090565 1.397193612 1.510234908 0.1309834944 0.2371965193 0.9967698847 0.7173025769 0 0 0.9689801135 0 0.7489427904 1.231679056 2.788529585 2.781693144 0 4.804813169 | | | | | | | | | | | | | | | | | | | |
| 15633 | ENSG00000257480 | 1.072809646 | 2.555477558 | 1.692283109 | 1.510076857 | 0.1310238141 | 0.2372391788 | 0.9967698847 | 0 | 0 | 0 | 1.937960227 | 3.891699813 | 0 | 0 | 1.394264792 | 4.172539716 | 0 | 0.4804813169 |

| 15634  15640  15650  15653  15659  15663  15668  15669 | ENSG00000177807 ENSG00000148600 ENSG00000134258 ENSG00000104055 ENSG00000249019 ENSG00000233261 ENSG00000064655 ENSG00000275294 | 1.837262801  1.404635323  0.9387970712  0.9901360747  2.45327948  1.530679649  9.561285087  0.8445438077 | 2.497668489  3.767353183  3.205720986  2.215303149  2.071517685  3.888878431  2.192985166  2.364040108 | 1.654047337  2.496287963  2.125634208  1.469118366  1.374332165  2.580765785  1.456135093  1.570034212 | 1.510034467  1.509182129  1.508124481  1.507913318  1.507290404  1.506869959  1.506031395  1.505725219 | 0.1310346295  0.1312522458  0.1315226707  0.1315767137  0.1317362361  0.1318439931  0.1320591143  0.1321377272 | 0.237243586  0.2375338071  0.2378687412  0.2379427925  0.23813308  0.238267003  0.2385796075  0.2387063952 | 0.9967698847  0  0  0  0.9967698847  0  2.990309654  0 | 0  0  0  0  0  0  2.869210308  0.7173025769 | 0.780045262  0  0  0.780045262  0.780045262  0  0.780045262  0 | 0  0  0  0  0.9271856394  0  4.635928197  0 | 0  0  0  1.937960227  0  0  0  0 | 0.9729249532  0  1.945849906  1.945849906  0.9729249532  0  0  0 | 0  0  0  0.7489427904  0  0  0  2.246828371 | 0.615839528  0  0  1.847518584  1.231679056  0.615839528  0  2.463358112 | 0  5.57705917  5.57705917  0  8.365588755  0  33.46235502  1.394264792 | 9.735926004  6.95423286  2.781693144  0  2.781693144  13.90846572  23.64439173  1.390846572 | 4.14083481  0  0  4.14083481  12.42250443  0  16.56333924  0 | 4.804813169  4.324331852  0.9609626337  0.4804813169  0.9609626337  3.843850535  29.78984165  1.921925267 |
| --- | --- | --- | --- | --- | --- | --- | --- | --- | --- | --- | --- | --- | --- | --- | --- | --- | --- | --- | --- |
| 15671 ENSG00000259031 11.45295535 2.185826798 1.451886232 1.505508317 0.1321934403 0.2387765631 1.993539769 3.586512885 2.340135786 5.563113837 0 0 0 0 13.94264792 44.50709031 28.98584367 36.51658008 | | | | | | | | | | | | | | | | | | | |
| 15679  15681  15686  15705  15712  15719  15722  15725  15728 | ENSG00000258423 ENSG00000145113 ENSG00000026559 ENSG00000162415 ENSG00000142512 ENSG00000250068 ENSG00000215380 ENSG00000251032 ENSG00000275025 | 1.201642847  0.8664963222  105.3630271  13.13105194  1.27548314  0.8647912038  0.8183098035  3.569057909  0.8130812464 | 2.518834621  2.339934998  2.156894223  2.032187861  2.122905687  2.74617231  2.232020394  2.055201726  2.23205737 | 1.674821598  1.556091321  1.434494224  1.353902119  1.414897995  1.831009109  1.488729617  1.371110712  1.489628588 | 1.503942046  1.503726013  1.503592128  1.500985804  1.500394865  1.499813571  1.499278558  1.498932003  1.498398586 | 0.1325962895  0.1326519284  0.1326864192  0.1333592333  0.1335121489  0.133662701  0.1338013825  0.133891273  0.1340297237 | 0.2393820123  0.239428661  0.2394378288  0.2403533062  0.2405292061  0.2406932003  0.2408969555  0.241012806  0.2412288416 | 0  0.9967698847  34.88694596  5.980619308  0  0  0  0  0 | 0  0  38.73433915  1.434605154  0  0  0  0.7173025769  0.7173025769 | 0.780045262  0  24.18140312  8.580497882  1.560090524  0  0.780045262  0.780045262  0 | 0  0  29.66994046  0.9271856394  0  0  0  2.781556918  0 | 0  1.937960227  1.937960227  0  4.844900568  0  0.9689801135  0  0.9689801135 | 0  2.91877486  0.9729249532  0  1.945849906  0  1.945849906  0  1.945849906 | 0  0  0.7489427904  0  0.7489427904  0  2.246828371  1.497885581  2.246828371 | 0.615839528  1.231679056  0.615839528  1.231679056  2.463358112  0.615839528  0.615839528  1.847518584  0.615839528 | 2.788529585  0  170.1003047  27.88529585  0  2.788529585  0  19.51970709  0 | 4.172539716  1.390846572  374.1377279  40.33455059  2.781693144  1.390846572  2.781693144  2.781693144  2.781693144 | 4.14083481  0  124.2250443  41.4083481  0  4.14083481  0  12.42250443  0 | 1.921925267  1.921925267  464.1449521  29.78984165  0.9609626337  1.441443951  0.4804813169  0.4804813169  0.4804813169 |
| 15730 ENSG00000125462 0.9014068463 3.177779921 2.121024705 1.498228622 0.1340738616 0.2412647633 0 0 0 0 0 0.9729249532 0 0 2.788529585 4.172539716 0 2.882887901 | | | | | | | | | | | | | | | | | | | |
| 15739  15753  15758  15778  15782  15783  15798  15808  15812 | ENSG00000248257 ENSG00000114547 ENSG00000231441 ENSG00000169297 ENSG00000101076 ENSG00000102243 ENSG00000272551 ENSG00000275234 ENSG00000046889 | 1.940341859  0.9556020652  1.288006272  1.52097417  1.236248071  0.9483680539  1.151141543  1.324809301  1.448037326 | 2.149743792  2.48080064  2.178513184  3.840670049  2.829831543  3.237976594  2.023560403  3.702018741  3.819092878 | 1.436273548  1.659169627  1.458259551  2.575300138  1.898055248  2.171968592  1.359148105  2.489081861  2.568459844 | 1.496750946  1.495206156  1.493913194  1.49134852  1.49091105  1.490802678  1.488844663  1.487302928  1.486919442 | 0.1344580745  0.1348606474  0.1351983093  0.1358700165  0.1359848497  0.1360133085  0.1365282781  0.1369348214  0.1370360887 | 0.2418177939  0.2423262544  0.2428411632  0.2437531175  0.243897298  0.243924884  0.2445947591  0.2451971821  0.2453011689 | 0.9967698847  0  0.9967698847  0  0.9967698847  0  0  0  0 | 1.434605154  0  0.7173025769  0  0  0  0.7173025769  0  0 | 0  0.780045262  0  0  0  0  0.780045262  0  0 | 0  0  0  0  0  0  0  0  0 | 8.720821022  4.844900568  3.875920454  0  6.782860795  0  2.906940341  0  0 | 2.91877486  0.9729249532  1.945849906  0  1.945849906  0  1.945849906  0  0 | 1.497885581  2.246828371  3.744713952  0  4.493656743  0  3.744713952  2.246828371  0 | 4.926716224  1.231679056  3.695037168  0  0.615839528  0.615839528  1.847518584  0  0 | 2.788529585  0  0  0  0  6.971323962  0  8.365588755  1.394264792 | 0  1.390846572  0  8.345079432  0  1.390846572  1.390846572  0  9.735926004 | 0  0  0  4.14083481  0  0  0  0  0 | 0  0  0.4804813169  5.765775802  0  2.402406584  0.4804813169  5.285294486  6.246257119 |
| 15822 ENSG00000182103 1.376674855 3.759207667 2.529140675 1.486357681 0.1371845373 0.2454114588 0 0 0 0 0 0 0 0 2.788529585 5.563386288 0 8.168182387 | | | | | | | | | | | | | | | | | | | |
| 15825  15827  15832  15842  15843  15856  15861  15864  15866 | ENSG00000266801 ENSG00000285095 ENSG00000162344 ENSG00000251402 ENSG00000237186 ENSG00000130988 ENSG00000255390 ENSG00000232110 ENSG00000070808 | 0.8107661899  1.258113531  1.097683608  2.250318342  1.313931916  0.8334169414  1.045070019  0.7297993591  1.213304489 | 2.320825686  2.058346797  3.309021594  2.941013395  2.027948827  3.133693833  2.255277676  2.968518917  2.545292286 | 1.561933842  1.385347104  2.228157738  1.982255031  1.366878154  2.115537526  1.523086105  2.005824919  1.720561925 | 1.485866829  1.485798607  1.48509306  1.483670541  1.483635407  1.481275465  1.480728942  1.479949166  1.479337796 | 0.137314349  0.1373323987  0.1375191741  0.1378963451  0.1379056708  0.1385331837  0.1386788184  0.1388868132  0.1390500561 | 0.2455983681  0.24561388  0.2458509366  0.2463889626  0.2463900724  0.2473082932  0.247490236  0.2478145572  0.2480745549 | 0  0  0  0  0.9967698847  0  0  0  0 | 0  0  0  0.7173025769  0  0  0.7173025769  0  0 | 0.780045262  0  0  0  0  0  0  0  0.780045262 | 0  1.854371279  0  0.9271856394  0.9271856394  0  0  0  0 | 0  3.875920454  0  0  0  1.937960227  0.9689801135  2.906940341  0 | 1.945849906  0.9729249532  0  0  0.9729249532  0.9729249532  0  1.945849906  0 | 1.497885581  1.497885581  0  0  0.7489427904  5.242599533  0  0  0.7489427904 | 1.231679056  1.231679056  0  0  1.847518584  1.847518584  0.615839528  2.463358112  0 | 0  0  1.394264792  8.365588755  2.788529585  0  1.394264792  0  5.57705917 | 1.390846572  2.781693144  1.390846572  8.345079432  5.563386288  0  2.781693144  0  1.390846572 | 0  0  4.14083481  0  0  0  4.14083481  0  4.14083481 | 2.882887901  2.882887901  6.246257119  8.648663704  1.921925267  0  1.921925267  1.441443951  1.921925267 |
| 15870 ENSG00000238160 1.128521416 2.745247358 1.857303738 1.478082072 0.139385812 0.2486108882 0 0.7173025769 0 0 2.906940341 1.945849906 6.740485114 1.231679056 0 0 0 0 | | | | | | | | | | | | | | | | | | | |
| 15871  15873  15879  15883  15887  15892  15894  15906  15907 | ENSG00000255441 ENSG00000265487 ENSG00000238649 ENSG00000227056 ENSG00000136352 ENSG00000204780 ENSG00000213265 ENSG00000274215 ENSG00000015592 | 0.888056363  1.085034263  0.8418370957  1.064139503  0.8180608064  0.8001411258  0.786036678  0.8229994194  1.329056404 | 2.346541018  3.215355679  2.341063922  2.407180098  3.06698427  3.087315785  2.289876355  2.71972563  3.694857957 | 1.587608765  2.175505176  1.584781321  1.630011253  2.077339395  2.0932036  1.552961129  1.845370001  2.507175631 | 1.478034809  1.477981167  1.477215746  1.476787411  1.476400186  1.474923789  1.474522647  1.473810471  1.473713254 | 0.1393984614  0.1394128191  0.1396178142  0.1397326323  0.1398364927  0.1402330339  0.1403409247  0.1405326283  0.1405588127 | 0.2486177839  0.2486208182  0.2488856539  0.2490005578  0.2491478418  0.2497757524  0.2499094358  0.2500890592  0.2501199317 | 0.9967698847  0  0  0  0  0  0  0  0 | 0  0  0  0  0  0  0.7173025769  0  0 | 0  0  0  0.780045262  0  0  0  0  0 | 0  0  0.9271856394  0  0  0  0  0  0 | 0.9689801135  0  0.9689801135  0  0  1.937960227  1.937960227  0.9689801135  0 | 0  0  1.945849906  0.9729249532  0  0.9729249532  0.9729249532  0.9729249532  0 | 0.7489427904  0  0  0  0  2.995771162  2.995771162  0  0 | 1.847518584  0  2.463358112  3.07919764  1.847518584  3.695037168  1.847518584  0  0 | 0  1.394264792  1.394264792  1.394264792  1.394264792  0  0  0  6.971323962 | 4.172539716  5.563386288  0  0  4.172539716  0  0  1.390846572  4.172539716 | 0  4.14083481  0  4.14083481  0  0  0  4.14083481  0 | 1.921925267  1.921925267  2.402406584  2.402406584  2.402406584  0  0.9609626337  2.402406584  4.804813169 |
| 15915 ENSG00000228824 2.660870756 2.392708693 1.624360193 1.473016086 0.1407466985 0.2503387817 1.993539769 0 0.780045262 0 0 0 0.7489427904 0 16.73117751 4.172539716 4.14083481 3.363369218 | | | | | | | | | | | | | | | | | | | |
| 15917  15919  15927  15929  15930  15952  15961  15968  15970 | ENSG00000222328 ENSG00000237927 ENSG00000256777 ENSG00000278090 ENSG00000143556 ENSG00000215867 ENSG00000241770 ENSG00000270426 ENSG00000163377 | 0.9421674969  1.464539885  6.532355182  0.7848310628  1.661406748  0.8013995026  0.7729175446  1.394147411  1.121997331 | 3.224142607  3.82156624  2.558719984  2.199400821  3.225696475  2.52912612  2.14119509  2.144360483  3.265327827 | 2.188983272  2.595124112  1.738789887  1.494842231  2.192571462  1.722339477  1.458597019  1.461791233  2.226574486 | 1.472895041  1.472594788  1.471552143  1.471326389  1.471193314  1.468424868  1.467982632  1.466940309  1.466525305 | 0.1407793393  0.1408603312  0.1411418571  0.14120287  0.1412388449  0.1419888506  0.1421089408  0.142392294  0.1425052326 | 0.2503549651  0.2504588588  0.2508420547  0.2509189805  0.2509671527  0.2518887193  0.2520186081  0.2524145963  0.2525831631 | 0  0  0  0  0  0  0.9967698847  1.993539769  0 | 0  0  1.434605154  0  0  0  0  0  0 | 0  0  0  0  0.780045262  0  0  0  0 | 0  0  4.635928197  0.9271856394  0  0  0  0  0 | 0  0  0  0.9689801135  0  0.9689801135  1.937960227  2.906940341  0 | 0  0  0  0.9729249532  0  0.9729249532  0  5.837549719  0 | 0  0.7489427904  0  0.7489427904  0  0.7489427904  1.497885581  1.497885581  0 | 2.463358112  0  0  0.615839528  0  0  0.615839528  1.231679056  0 | 6.971323962  13.94264792  18.1254423  0  5.57705917  1.394264792  1.394264792  0  6.971323962 | 1.390846572  0  22.25354515  2.781693144  9.735926004  1.390846572  1.390846572  2.781693144  1.390846572 | 0  0  16.56333924  0  0  4.14083481  0  0  4.14083481 | 0.4804813169  2.882887901  15.37540214  2.402406584  3.843850535  0  1.441443951  0.4804813169  0.9609626337 |
| 15987 ENSG00000216101 0.8716459483 2.304664539 1.57435493 1.46387863 0.1432271132 0.2535927106 0 0 0 0.9271856394 1.937960227 0 0.7489427904 1.231679056 0 4.172539716 0 1.441443951 | | | | | | | | | | | | | | | | | | | |
| 16018  16024  16025  16026  16039  16042  16045  16059  16060 | ENSG00000198788 ENSG00000274248 ENSG00000283283 ENSG00000283167 ENSG00000140955 ENSG00000212190 ENSG00000230747 ENSG00000279949 ENSG00000253661 | 0.7866659189  0.7395522499  0.8739372621  0.8739372621  1.198296069  0.8600609076  0.900899717  1.288446591  1.288446591 | 2.614354976  2.978625236  3.185822254  3.185822254  3.150359894  2.727060195  2.795798317  3.651789089  3.651789089 | 1.791674679  2.042925212  2.185223242  2.185223242  2.162504877  1.872272103  1.91997752  2.511496746  2.511496746 | 1.459168345  1.458019715  1.457893268  1.457893268  1.456810539  1.456551209  1.456162006  1.454028995  1.454028995 | 0.144518779  0.1448351089  0.1448699645  0.1448699645  0.145168686  0.1452403043  0.1453478398  0.1459382676  0.1459382676 | 0.2553844774  0.2558578837  0.2558613099  0.2558613099  0.256181099  0.25627553  0.2564173234  0.2572184684  0.2572184684 | 0  0  0  0  0  0  0  0  0 | 0  0  0  0  0  0  0  0  0 | 0  0  0  0  0  0  0  0  0 | 0  0  0  0  0  0  0  0  0 | 0.9689801135  0  2.906940341  2.906940341  0  1.937960227  0  0  0 | 0  0  0.9729249532  0.9729249532  0  0.9729249532  0  0  0 | 1.497885581  1.497885581  5.991542324  5.991542324  0  0  0  0  0 | 0  1.231679056  0.615839528  0.615839528  0  0  0.615839528  0  0 | 0  0  0  0  1.394264792  2.788529585  4.182794377  4.182794377  4.182794377 | 1.390846572  2.781693144  0  0  2.781693144  0  1.390846572  6.95423286  6.95423286 | 4.14083481  0  0  0  8.281669619  4.14083481  4.14083481  0  0 | 1.441443951  3.363369218  0  0  1.921925267  0.4804813169  0.4804813169  4.324331852  4.324331852 |
| 16061 ENSG00000128709 1.009455344 3.087642796 2.123762412 1.453855092 0.1459864857 0.2572553984 0 0 0 0 0 0 0 0 2.788529585 2.781693144 4.14083481 2.402406584 | | | | | | | | | | | | | | | | | | | |
| 16076 | ENSG00000281189 | 3.737164357 | 2.16282238 | 1.48872786 | 1.452799022 | 0.1462795636 | 0.2575153509 | 1.993539769 | 2.151907731 | 0 | 0 | 0 | 0 | 2.995771162 | 0 | 2.788529585 | 5.563386288 | 20.70417405 | 8.648663704 |

| 16094  16096  16100  16110  16119  16121  16123  16137 | ENSG00000283345 ENSG00000131668 ENSG00000124215 ENSG00000201801 ENSG00000111262 ENSG00000225173 ENSG00000284837 ENSG00000229298 | 1.506947627  1.277781321  0.9376503593  1.013770212  1.300811084  0.8001076344  0.8392575765  1.013671792 | 2.303440584  2.74138018  2.387394932  3.117518673  3.687259143  3.029753233  2.31929313  3.129224607 | 1.586963676  1.889113237  1.645368063  2.150723495  2.545470217  2.092014455  1.601578885  2.163592812 | 1.451476564  1.451146563  1.450979258  1.449520908  1.448557173  1.448246796  1.448129188  1.446309393 | 0.1466472034  0.1467390532  0.1467856359  0.1471921658  0.1474612885  0.1475480413  0.147580924  0.1480904423 | 0.2579528328  0.2580514189  0.2580949731  0.2586233052  0.2589515002  0.2590692699  0.2590972917  0.2597501586 | 0  0  0  0  0  0  0  0 | 0.7173025769  0.7173025769  0  0  0  0  0  0 | 0  0  0.780045262  0  0  0  0.780045262  0 | 0.9271856394  0  0  0  0  0  0  0 | 0.9689801135  0  0  0.9689801135  0  0  0.9689801135  0 | 0.9729249532  0  0.9729249532  0  0  0  0  0 | 0  1.497885581  0  0  0  0.7489427904  2.995771162  0 | 0  0  0.615839528  0  0  1.847518584  0.615839528  0 | 2.788529585  0  2.788529585  0  2.788529585  0  2.788529585  2.788529585 | 8.345079432  4.172539716  4.172539716  4.172539716  4.172539716  5.563386288  0  1.390846572 | 0  4.14083481  0  4.14083481  0  0  0  4.14083481 | 3.363369218  4.804813169  1.921925267  2.882887901  8.648663704  1.441443951  1.921925267  3.843850535 |
| --- | --- | --- | --- | --- | --- | --- | --- | --- | --- | --- | --- | --- | --- | --- | --- | --- | --- | --- | --- |
| 16154 ENSG00000249241 1.459568793 2.76559145 1.915598181 1.443722111 0.1488171591 0.2607662811 0 0 0.780045262 0 0 0 0 0 2.788529585 2.781693144 8.281669619 2.882887901 | | | | | | | | | | | | | | | | | | | |
| 16159  16160  16163  16167  16174  16179  16186  16187  16211 | ENSG00000183378 ENSG00000204363 ENSG00000163508 ENSG00000235563 ENSG00000242082 ENSG00000172794 ENSG00000102962 ENSG00000215853 ENSG00000188620 | 0.7345028283  0.8413957274  3.307513597  1.33284637  1.368298969  7.57596475  0.7666287993  0.8411162966  1.248691333 | 2.963036526  2.687511681  2.271716344  3.719747867  2.808828674  2.094349809  2.225819263  3.088901344  3.608008523 | 2.053212135  1.862395372  1.575043615  2.57988568  1.94908635  1.453923021  1.546002411  2.146277358  2.511303255 | 1.44312245  1.443040356  1.442319643  1.441826626  1.441100172  1.440481909  1.43972561  1.439190202  1.43670762 | 0.1489859803  0.1490091033  0.1492122214  0.1493512894  0.1495563849  0.1497311046  0.1499450449  0.1500966408  0.1508010888 | 0.2609813206  0.2610056732  0.2613129455  0.2614917794  0.261759357  0.2619623368  0.262223183  0.2624576443  0.2633135291 | 0  0  1.993539769  0  0.9967698847  4.983849424  0  0  0 | 0  0  0  0  0  0  0.7173025769  0  0 | 0  0  0.780045262  0  0  4.680271572  0  0  0 | 0  0  0.9271856394  0  0  0  0  0  0 | 2.906940341  1.937960227  0  6.782860795  0  34.88328409  0.9689801135  0.9689801135  0 | 1.945849906  0  0  0.9729249532  0  3.891699813  0  1.945849906  0 | 1.497885581  0.7489427904  0  8.238370695  0  33.70242557  0.7489427904  2.995771162  0 | 2.463358112  0  0  0  0  1.847518584  0.615839528  0  0 | 0  2.788529585  6.971323962  0  1.394264792  0  1.394264792  4.182794377  5.57705917 | 0  0  11.12677258  0  5.563386288  2.781693144  1.390846572  0  5.563386288 | 0  4.14083481  8.281669619  0  4.14083481  4.14083481  0  0  0 | 0  0.4804813169  9.609626337  0  4.324331852  0  3.363369218  0  3.843850535 |
| 16212 ENSG00000254403 0.8349025613 3.118714958 2.170892905 1.436604703 0.1508303464 0.2633483707 0 0 0 0 0.9689801135 5.837549719 0.7489427904 2.463358112 0 0 0 0 | | | | | | | | | | | | | | | | | | | |
| 16223  16224  16234  16241  16242  16243  16248  16253  16255 | ENSG00000165325 ENSG00000228705 ENSG00000246316 ENSG00000163132 ENSG00000273272 ENSG00000160183 ENSG00000230516 ENSG00000267188 ENSG00000100253 | 1.044994153  0.7644890851  1.163184415  213.0999174  0.889977874  1.453684595  0.8358430326  5.000545029  0.7496205016 | 3.137545513  2.175684262  3.080658072  2.532315558  2.341621146  2.169125131  2.656442819  2.037374036  2.989308426 | 2.18616125  1.516099533  2.149572298  1.767745578  1.63460436  1.514287959  1.854983382  1.423049565  2.088107999 | 1.435184853  1.435053711  1.433149318  1.432511324  1.432530833  1.432439001  1.432057475  1.431695766  1.431587076 | 0.1512344287  0.1512717926  0.1518151699  0.1519975397  0.1519919604  0.1520182236  0.1521273732  0.1522309088  0.1522620304 | 0.2638748529  0.2639237772  0.2646923437  0.2648960941  0.2648960941  0.2649158307  0.2650166616  0.2651232452  0.2651448189 | 0  0  0  30.89986643  0  1.993539769  0  1.993539769  0 | 0  0  0  72.44756027  0.7173025769  0  0  2.151907731  0 | 0  0.780045262  0  35.10203679  0  0  0  2.340135786  0 | 0  0  0  64.90299476  0  0  0  0  0 | 0  1.937960227  0  0.9689801135  0.9689801135  0  0  0  1.937960227 | 0  0.9729249532  0.9729249532  0  0  0.9729249532  0  0.9729249532  0 | 0  2.995771162  0  0  1.497885581  0.7489427904  0.7489427904  0  3.744713952 | 0  0.615839528  0  0.615839528  0  0.615839528  0  0  0 | 1.394264792  0  0  539.5804747  4.182794377  5.57705917  2.788529585  12.54838313  0 | 5.563386288  1.390846572  2.781693144  611.9724917  1.390846572  4.172539716  1.390846572  18.08100544  1.390846572 | 4.14083481  0  8.281669619  542.4493601  0  0  4.14083481  4.14083481  0 | 1.441443951  0.4804813169  1.921925267  658.2594041  1.921925267  3.363369218  0.9609626337  17.77780872  1.921925267 |
| 16261 ENSG00000201558 0.8980449532 3.150711037 2.201797413 1.430972268 0.1524381634 0.2653535854 0 0 0 0 0 0.9729249532 0 0 6.971323962 1.390846572 0 1.441443951 | | | | | | | | | | | | | | | | | | | |
| 16263  16266  16268  16278  16280  16282  16288  16289  16296 | ENSG00000180660 ENSG00000249650 ENSG00000117600 ENSG00000224646 ENSG00000236491 ENSG00000218153 ENSG00000134539 ENSG00000183476 ENSG00000249077 | 5.607744391  1.532070671  1.252338078  1.026623276  1.08167237  0.9451484656  4.673747542  8.698979631  0.7534061742 | 2.197316034  2.869918186  3.620410185  2.326316304  3.189058449  3.206747492  2.315593217  2.246007216  2.961318485 | 1.53563221  2.006243342  2.531131823  1.627264885  2.231326868  2.243862627  1.62095535  1.572212352  2.075473391 | 1.430886914  1.430493563  1.430352284  1.429586741  1.429220655  1.429119347  1.42853609  1.428564795  1.426815925 | 0.1524626281  0.1525754121  0.1526159359  0.1528356631  0.1529408227  0.1529699337  0.1531376146  0.1531293588  0.1536329598 | 0.2653635338  0.2655081919  0.2655487263  0.2657676791  0.2659015372  0.2659358152  0.2661129178  0.2661129178  0.2668590182 | 0  0  0  0  0  0  2.990309654  2.990309654  0 | 2.869210308  0  0  0  0  0  0.7173025769  4.303815461  0 | 1.560090524  0.780045262  0  0  0  0  1.560090524  2.340135786  0 | 1.854371279  0  0  0.9271856394  0  0  0  0  0 | 0  0  0  0.9689801135  0  0  21.3175625  0  2.906940341 | 0  0  0  0  0  0.9729249532  0  0  0 | 0  0  0  3.744713952  0  0  26.96194046  0  1.497885581 | 0  0  0  0.615839528  0  0.615839528  0.615839528  0  1.847518584 | 11.15411834  6.971323962  2.788529585  0  5.57705917  6.971323962  0  15.33691272  2.788529585 | 12.51761915  1.390846572  6.95423286  0  2.781693144  2.781693144  0  25.0352383  0 | 24.84500886  8.281669619  0  4.14083481  4.14083481  0  0  41.4083481  0 | 12.49251424  0.9609626337  5.285294486  1.921925267  0.4804813169  0  1.921925267  12.97299556  0 |
| 16302 ENSG00000177519 1.300526232 3.685996998 2.584381844 1.426258665 0.1537936914 0.2670398864 0 0 0 0 0 0 0 0 1.394264792 5.563386288 0 8.648663704 | | | | | | | | | | | | | | | | | | | |
| 16314  16317  16333  16337  16345  16347  16362  16370  16371 | ENSG00000284664 ENSG00000229142 ENSG00000137948 ENSG00000206828 ENSG00000163554 ENSG00000212385 ENSG00000235821 ENSG00000179460 ENSG00000256943 | 0.7706006915  0.8429878886  0.7462522401  0.7656027595  1.212867671  1.816697883  0.7932439423  6.259097143  0.7380579633 | 3.009100093  3.076108582  2.979971786  3.011199879  3.576163275  2.119481377  2.242808003  2.241649068  2.982407535 | 2.111636336  2.159555088  2.095549315  2.119065174  2.517829271  1.492673724  1.581022718  1.581632946  2.104588137 | 1.425008673  1.424417742  1.422048035  1.421003901  1.420335889  1.419922748  1.418580503  1.417300439  1.41709795 | 0.154154694  0.1543255812  0.1550123068  0.1553156254  0.1555099176  0.1556301726  0.1560213533  0.1563951069  0.1564542918 | 0.2674698277  0.2677170989  0.2686449738  0.2690954335  0.2693116958  0.2694682936  0.2698981479  0.2704288269  0.2705146407 | 0  0  0  0  0  0  0  1.993539769  0 | 0  0  0  0  0  0.7173025769  0  1.434605154  0 | 0  0  0  0  0  0  0  0.780045262  0 | 0  0  0  0  0  0.9271856394  0.9271856394  2.781556918  0 | 0  0.9689801135  0.9689801135  0  0  0  1.937960227  0  0.9689801135 | 3.891699813  0  3.891699813  1.945849906  0  0  1.945849906  0.9729249532  1.945849906 | 1.497885581  0  2.246828371  0  0  0.7489427904  2.995771162  0  2.246828371 | 2.463358112  1.231679056  1.847518584  0.615839528  0  1.847518584  1.231679056  0  3.695037168 | 1.394264792  0  0  0  5.57705917  1.394264792  0  2.788529585  0 | 0  6.95423286  0  2.781693144  4.172539716  2.781693144  0  0  0 | 0  0  0  0  0  12.42250443  0  33.12667848  0 | 0  0.9609626337  0  3.843850535  4.804813169  0.9609626337  0.4804813169  31.2312856  0 |
| 16374 ENSG00000251127 0.7577007811 3.00075961 2.118148444 1.416689949 0.156573597 0.2706701755 0 0 0 0 0.9689801135 0 1.497885581 0 0 2.781693144 0 3.843850535 | | | | | | | | | | | | | | | | | | | |
| 16382  16383  16401  16406  16414  16418  16422  16432  16434 | ENSG00000279078 ENSG00000244556 ENSG00000255129 ENSG00000284293 ENSG00000267316 ENSG00000197253 ENSG00000066336 ENSG00000284034 ENSG00000279476 | 1.089537113  0.7832441614  1.40706712  0.9768201188  0.9675241405  1.502053568  0.9436978844  0.7873340422  1.054432981 | 2.329271439  2.602923846  2.034997241  2.528725787  2.522637195  2.512102402  2.079818269  2.583652377  2.669521559 | 1.646359717  1.839868901  1.440125923  1.790438343  1.787333748  1.7809685  1.475130593  1.834779496  1.896064296 | 1.41480104  1.414733324  1.41306896  1.412350108  1.411396835  1.410526015  1.409921453  1.408154158  1.407927761 | 0.1571268393  0.1571467  0.1576354487  0.1578468993  0.1581276356  0.1583844202  0.1585628774  0.159085427  0.1591524617 | 0.2714950746  0.2714993461  0.2720583508  0.2723402615  0.2726916566  0.2730679376  0.2733090249  0.2740550626  0.2741249593 | 0  0  0.9967698847  0  0  0  0  0  0 | 0  0  0  0  0.7173025769  0  0  0  0.7173025769 | 0  0  0.780045262  0  0  0.780045262  0.780045262  0  0 | 0.9271856394  0  0  0.9271856394  0  0  0  0  0 | 0  0.9689801135  0.9689801135  2.906940341  2.906940341  0.9689801135  1.937960227  1.937960227  0.9689801135 | 0  0.9729249532  0  1.945849906  3.891699813  0  0  0  1.945849906 | 2.246828371  0  1.497885581  2.246828371  2.246828371  1.497885581  1.497885581  1.497885581  2.246828371 | 0.615839528  0  0  3.695037168  1.847518584  0  0.615839528  0  6.774234807 | 4.182794377  1.394264792  1.394264792  0  0  1.394264792  0  0  0 | 0  0  2.781693144  0  0  0  1.390846572  1.390846572  0 | 4.14083481  4.14083481  4.14083481  0  0  12.42250443  4.14083481  4.14083481  0 | 0.9609626337  1.921925267  4.324331852  0  0  0.9609626337  0.9609626337  0.4804813169  0 |
| 16435 ENSG00000177257 0.8065930431 3.047646695 2.164746712 1.407853712 0.1591743918 0.2741460502 0 0 0 0 0 0 0.7489427904 0 1.394264792 4.172539716 0 3.363369218 | | | | | | | | | | | | | | | | | | | |
| 16439  16449  16450  16464  16475  16480  16489  16502  16507 | ENSG00000167157 ENSG00000224363 ENSG00000228997 ENSG00000230997 ENSG00000271727 ENSG00000228661 ENSG00000171234 ENSG00000242021 ENSG00000230698 | 0.7851742286  0.7566374701  0.7164951214  0.8431287129  0.9861893207  0.7532456722  1.434060057  1.268919019  4.083849329 | 3.002239136  2.095011305  2.934669062  3.076552823  2.543393199  2.191069307  3.057110851  3.666053545  2.287954553 | 2.133736475  1.491174468  2.088827702  2.192997198  1.81473385  1.564380986  2.185810261  2.62461638  1.638879988 | 1.407033705  1.404940435  1.404935916  1.402898657  1.401524085  1.400598273  1.398616754  1.396795956  1.396047649 | 0.1594173947  0.1600389914  0.1600403352  0.1606470615  0.1610574104  0.1613342378  0.1619279396  0.1624749388  0.162700147 | 0.2744977659  0.2753861233  0.2753861233  0.2761950755  0.276715694  0.2771072169  0.2779582934  0.2786944381  0.2789962053 | 0  0  0  0  0  0  0  0  3.987079539 | 0  0.7173025769  0  0  0.7173025769  0  0  0  0 | 0  0  0  0  0  0.780045262  0.780045262  0  0.780045262 | 0  0  0  0  0  0  0  0  0 | 0.9689801135  0  0.9689801135  0  2.906940341  0  4.844900568  0  14.5347017 | 0  0.9729249532  2.91877486  0.9729249532  4.864624766  1.945849906  0  0  5.837549719 | 0  1.497885581  2.246828371  0.7489427904  1.497885581  1.497885581  9.736256276  0  16.47674139 | 0  1.231679056  2.463358112  0  1.847518584  2.463358112  1.847518584  0  7.390074335 | 2.788529585  2.788529585  0  0  0  0  0  1.394264792  0 | 2.781693144  1.390846572  0  6.95423286  0  1.390846572  0  2.781693144  0 | 0  0  0  0  0  0  0  0  0 | 2.882887901  0.4804813169  0  1.441443951  0  0.9609626337  0  11.05107029  0 |
| 16520 ENSG00000156269 0.7126112498 2.911244145 2.087611926 1.394533202 0.1631566504 0.2795428945 0 0 0 0 3.875920454 1.945849906 1.497885581 1.231679056 0 0 0 0 | | | | | | | | | | | | | | | | | | | |
| 16522 | ENSG00000235415 | 0.7126112498 | 2.911244145 | 2.087611926 | 1.394533202 | 0.1631566504 | 0.2795428945 | 0 | 0 | 0 | 0 | 3.875920454 | 1.945849906 | 1.497885581 | 1.231679056 | 0 | 0 | 0 | 0 |

| 16566  16571  16572  16596  16614  16621  16629  16634 | ENSG00000246363 ENSG00000270090 ENSG00000231956 ENSG00000267554 ENSG00000258884 ENSG00000250897 ENSG00000069188 ENSG00000253671 | 1.208022572  1.358171656  1.032150045  0.7511421113  1.821298833  1.080795261  0.9819342614  1.123098059 | 3.59013607  3.545306999  2.248451403  2.106033642  2.039409919  2.33088986  2.561271807  3.496559645 | 2.589001745  2.557915231  1.622216226  1.523391654  1.478566779  1.691363122  1.85967065  2.540793883 | 1.386687389  1.386014265  1.386036811  1.382463687  1.379315394  1.378113209  1.377271726  1.376168161 | 0.1655371076  0.1657425477  0.1657356636  0.166829373  0.1677975323  0.1681683378  0.1684282532  0.1687695769 | 0.2828500161  0.2830985128  0.2830985128  0.2845541597  0.2858840104  0.2863951008  0.2867057037  0.2871845831 | 0  0  0  0  0.9967698847  0  0.9967698847  0 | 0  0  0  0.7173025769  1.434605154  0.7173025769  0  0 | 0  0  0.780045262  0  0  0  0  0 | 0  0  0  0  0  0  0  0 | 6.782860795  0  0  0.9689801135  0  0  2.906940341  7.751840908 | 0.9729249532  0  0  1.945849906  0.9729249532  0  0  0 | 6.740485114  0  0  0.7489427904  1.497885581  0  1.497885581  4.493656743 | 0  0  1.847518584  1.847518584  0  0.615839528  0.615839528  1.231679056 | 0  0  1.394264792  1.394264792  4.182794377  4.182794377  0  0 | 0  4.172539716  2.781693144  1.390846572  5.563386288  1.390846572  0  0 | 0  8.281669619  4.14083481  0  0  4.14083481  0  0 | 0  3.843850535  1.441443951  0  7.207219753  1.921925267  5.765775802  0 |
| --- | --- | --- | --- | --- | --- | --- | --- | --- | --- | --- | --- | --- | --- | --- | --- | --- | --- | --- | --- |
| 16644 ENSG00000226913 1.140650645 3.509859667 2.552168352 1.375246137 0.1690551491 0.2874557007 0 0 0 0 0 0 0 0 2.788529585 4.172539716 0 6.726738436 | | | | | | | | | | | | | | | | | | | |
| 16663  16666  16669  16670  16673  16676  16685  16688  16695 | ENSG00000273908 ENSG00000231133 ENSG00000124302 ENSG00000278434 ENSG00000174792 ENSG00000047648 ENSG00000261678 ENSG00000156466 ENSG00000271021 | 0.9335915723  0.9333067206  1.180405903  1.02846524  2.003273286  2.784101141  1.220597979  2.638107835  1.176901463 | 2.957704103  2.957252494  3.555478888  3.106389187  2.150645339  2.150548228  2.648447137  2.666434584  3.539653755 | 2.153945162  2.153857503  2.590980696  2.263813815  1.567731903  1.567807947  1.931945055  1.945204484  2.584307129 | 1.373156641  1.373002851  1.372252172  1.372192875  1.371819591  1.371691114  1.370870839  1.37077341  1.369672248 | 0.1697036557  0.1697514603  0.169984949  0.170003403  0.1701196069  0.170159616  0.1704152233  0.1704456022  0.1707892355 | 0.2882697564  0.2883106225  0.2886552263  0.2886692457  0.2887972648  0.2888343947  0.2890810893  0.2891079348  0.2895693381 | 0  0  0  0  0  0  0  0  0 | 0  0  0  0  0.7173025769  1.434605154  0  2.151907731  0 | 0  0  0  0  0  0.780045262  0.780045262  0  0 | 0  0  0  0  1.854371279  0.9271856394  0  0  0 | 0  0  0  0  4.844900568  0  0  0  0 | 0  0  0  0  5.837549719  0  0  0  0.9729249532 | 0  0  0  0.7489427904  5.242599533  0  0.7489427904  0  0 | 0  0  0  0  5.542555752  0  0  0  0 | 2.788529585  1.394264792  1.394264792  6.971323962  0  4.182794377  0  2.788529585  0 | 1.390846572  2.781693144  5.563386288  0  0  12.51761915  4.172539716  8.345079432  8.345079432 | 4.14083481  4.14083481  0  4.14083481  0  8.281669619  4.14083481  8.281669619  0 | 2.882887901  2.882887901  7.207219753  0.4804813169  0  5.285294486  4.804813169  10.09010765  4.804813169 |
| 16696 ENSG00000236305 1.66646119 2.126033745 1.552279645 1.369620321 0.170805453 0.2895794892 0 0 0 1.854371279 5.813880681 0.9729249532 1.497885581 0.615839528 0 0 8.281669619 0.9609626337 | | | | | | | | | | | | | | | | | | | |
| 16706  16707  16709  16713  16714  16716  16717  16720  16722 | ENSG00000148541 ENSG00000254744 ENSG00000280055 ENSG00000184408 ENSG00000270640 ENSG00000231105 ENSG00000204252 ENSG00000227113 ENSG00000196361 | 1.061105663  1.025674067  1.263579291  1.164394665  0.760409219  0.7597517457  1.159929009  0.780775638  1.368956189 | 2.301934087  2.221595029  2.29571303  3.500531819  2.538444472  2.537100971  3.540149948  2.14698084  2.696143591 | 1.681804106  1.623241082  1.677957475  2.560803418  1.857365119  1.857009515  2.591351089  1.571922315  1.974186451 | 1.368729021  1.368616808  1.368159244  1.36696624  1.366691151  1.366229387  1.366140607  1.365831389  1.365698559 | 0.1710839952  0.1711190872  0.1712622359  0.1716358877  0.1717221328  0.1718669764  0.1718948351  0.1719918917  0.1720335969 | 0.2898796354  0.289920206  0.2901280058  0.2906914042  0.2908200724  0.2910305476  0.29106031  0.2911723975  0.2912081685 | 0  0.9967698847  0  0  0  0  0  0.9967698847  0 | 0  0  0  0  0  0  0  0  0.7173025769 | 0.780045262  0  0  0  0  0  0  0  0 | 0  0  0.9271856394  0  0  0  0  0  0 | 0.9689801135  0.9689801135  0  0  0.9689801135  2.906940341  5.813880681  1.937960227  0 | 0  0  0.9729249532  0  2.91877486  0.9729249532  0  0.9729249532  0 | 0.7489427904  1.497885581  0  0  0  0  7.489427904  0  0.7489427904 | 0  0  1.231679056  0  0.615839528  0.615839528  0.615839528  1.231679056  0 | 0  0  2.788529585  5.57705917  0  0  0  2.788529585  1.394264792 | 4.172539716  2.781693144  0  6.95423286  0  0  0  0  0 | 4.14083481  4.14083481  8.281669619  0  4.14083481  4.14083481  0  0  8.281669619 | 1.921925267  1.921925267  0.9609626337  1.441443951  0.4804813169  0.4804813169  0  1.441443951  5.285294486 |
| 16724 ENSG00000228290 0.9375231688 3.003096887 2.200317992 1.364846762 0.172301218 0.2916088655 0 0 0 0 0 0 0 0 1.394264792 1.390846572 4.14083481 4.324331852 | | | | | | | | | | | | | | | | | | | |
| 16727  16808  16818  16824  16826  16827  16841  16844  16852 | ENSG00000182397 ENSG00000255807 ENSG00000092621 ENSG00000187486 ENSG00000279711 ENSG00000224518 ENSG00000261594 ENSG00000238109 ENSG00000145506 | 2.001577356  0.7174822787  978.6893206  1.375727196  1.16524922  1.385458505  1.136149345  0.8374010618  31.97667252 | 2.739045095  2.419104791  2.077268126  2.948087227  3.501846145  2.63183069  3.494694058  2.210621722  2.481439557 | 2.007685787  1.787126008  1.536853826  2.181938616  2.59225582  1.948377406  2.589862342  1.638878555  1.841462337 | 1.364279765  1.353628553  1.35163676  1.351132065  1.350887562  1.350780748  1.349374444  1.348862437  1.347537502 | 0.1724795318  0.1758549128  0.1764915447  0.1766531317  0.176731453  0.1767656769  0.1772167251  0.1773811554  0.1778071838 | 0.2918904887  0.2961535675  0.2970313136  0.2972149041  0.2973113342  0.2973512361  0.297862159  0.29808543  0.2986595149 | 0.9967698847  0  363.8210079  0.9967698847  0  0  0  0  16.94508804 | 0  0  292.6594514  0  0  0.7173025769  0  0  2.869210308 | 0.780045262  0  330.7391911  0  0  0  0  0.780045262  7.020407358 | 0  0  256.8304221  0  0  0  0  0  4.635928197 | 0  0  22.28654261  0  0  0  0  0.9689801135  0 | 0  0  0.9729249532  0  0  1.945849906  0  0  0 | 0  1.497885581  11.23414186  0  0  1.497885581  0  1.497885581  0 | 0  0.615839528  0.615839528  0  0  0  0  1.231679056  0 | 5.57705917  1.394264792  1643.83819  4.182794377  9.759853547  4.182794377  1.394264792  2.788529585  103.1755946 | 4.172539716  0  3468.771351  5.563386288  2.781693144  0  6.95423286  2.781693144  75.10571489 | 0  4.14083481  1573.517228  0  0  8.281669619  0  0  107.6617051 | 12.49251424  0.9609626337  3778.985557  5.765775802  1.441443951  0  5.285294486  0  66.30642173 |
| 16860 ENSG00000164122 2.54402961 2.367040071 1.758581005 1.345994335 0.1783043435 0.2993524761 0.9967698847 0.7173025769 0 0.9271856394 0 0 0 0 20.91397189 1.390846572 4.14083481 1.441443951 | | | | | | | | | | | | | | | | | | | |
| 16867  16868  16870  16872  16897  16901  16910  16962  16970 | ENSG00000172572 ENSG00000138798 ENSG00000251175 ENSG00000263711 ENSG00000254238 ENSG00000248936 ENSG00000215887 ENSG00000284108 ENSG00000122224 | 1.093032194  1.814213334  1.006805459  1.540119998  1.313915098  0.8686620784  1.056638829  0.782718906  0.724867608 | 3.431079677  2.027879406  3.054771852  2.409238346  3.447947695  2.243466357  3.394876924  2.169183844  2.431440751 | 2.551663631  1.508495684  2.273161541  1.793873346  2.574702007  1.676431313  2.539169838  1.631082781  1.828895041 | 1.34464419  1.34430574  1.343842836  1.343037038  1.339163789  1.338239353  1.337002698  1.329904202  1.329458879 | 0.1787401656  0.1788495402  0.1789992144  0.179259981  0.1805173579  0.1808184244  0.1812217561  0.1835498369  0.1836966236 | 0.2999706756  0.3001253903  0.3003409462  0.3007428296  0.3024042335  0.3028518154  0.3033363453  0.306293382  0.3064064012 | 0  0  0  0  0  0  0  0  0 | 0  1.434605154  0  0  0  0  0  0  0 | 0  0  0  1.560090524  0  0.780045262  0  0  0 | 0  0.9271856394  0  0  0  0  0  0.9271856394  0 | 0  0  0  5.813880681  0  0  0  0.9689801135  0 | 0  0.9729249532  0.9729249532  2.91877486  0  0.9729249532  0  0  0.9729249532 | 0  0  0  4.493656743  0  0.7489427904  0  2.995771162  0.7489427904 | 0  0.615839528  0  3.695037168  0  0  0  1.231679056  0 | 6.971323962  6.971323962  5.57705917  0  0  2.788529585  4.182794377  2.788529585  1.394264792 | 2.781693144  5.563386288  1.390846572  0  5.563386288  4.172539716  4.172539716  0  0 | 0  0  4.14083481  0  8.281669619  0  0  0  4.14083481 | 3.363369218  5.285294486  0  0  1.921925267  0.9609626337  4.324331852  0.4804813169  1.441443951 |
| 16972 ENSG00000273133 0.7242540197 2.4302895 1.828562091 1.329071357 0.1838244291 0.3065834486 0 0 0 0 0.9689801135 0 0.7489427904 0 0 1.390846572 4.14083481 1.441443951 | | | | | | | | | | | | | | | | | | | |
| 16976  16985  16986  17007  17024  17033  17059  17069  17108 | ENSG00000100678 ENSG00000231416 ENSG00000233651 ENSG00000228022 ENSG00000278261 ENSG00000225636 ENSG00000110076 ENSG00000243033 ENSG00000272189 | 0.8974830591  1.268046197  1.007908548  0.7712696194  0.9921492215  0.7847109843  1.334406334  0.7228660788  0.780365523 | 2.912949099  2.015845175  2.579847277  2.153001717  2.17816061  2.505815064  2.169174402  2.854093787  2.192220372 | 2.193077637  1.519612927  1.944730048  1.627328317  1.648763362  1.898351883  1.646755495  2.168741544  1.673601179 | 1.32824714  1.326551741  1.326583748  1.323028484  1.321087465  1.319995037  1.317241332  1.316013794  1.309882187 | 0.184096476  0.1846570086  0.1846464147  0.1858259055  0.1864722022  0.1868366751  0.1877577436  0.1881694129  0.1902356942 | 0.3069648239  0.3077181966  0.3077181966  0.3092636501  0.3100453024  0.310491336  0.3115464382  0.3120465992  0.3147403707 | 0  0  0  0.9967698847  0  0  0  0  0 | 0  0.7173025769  0  0  0.7173025769  0  0  0  0.7173025769 | 0  0  0  0  0  0  1.560090524  0  0 | 0  0.9271856394  0  0  0  0  0  0  0 | 0  0  0.9689801135  1.937960227  1.937960227  0  1.937960227  0  0.9689801135 | 0  0  0.9729249532  0.9729249532  0  0  0  0  0 | 0  1.497885581  0  2.995771162  0  0  0.7489427904  0.7489427904  0 | 0  1.847518584  0  0  1.847518584  0.615839528  0  0  0.615839528 | 1.394264792  4.182794377  0  0  0  2.788529585  2.788529585  4.182794377  2.788529585 | 1.390846572  5.563386288  1.390846572  1.390846572  2.781693144  1.390846572  4.172539716  2.781693144  1.390846572 | 4.14083481  0  8.281669619  0  4.14083481  4.14083481  0  0  0 | 3.843850535  0.4804813169  0.4804813169  0.9609626337  0.4804813169  0.4804813169  4.804813169  0.9609626337  2.882887901 |
| 17116 ENSG00000241149 0.7328427426 2.151800434 1.643889285 1.308969195 0.1905447858 0.3151154473 0 0.7173025769 0 0 0 2.91877486 0.7489427904 0.615839528 0 1.390846572 0 2.402406584 | | | | | | | | | | | | | | | | | | | |
| 17118  17120  17136  17138  17142  17144  17148  17155  17162 | ENSG00000246465 ENSG00000250519 ENSG00000253141 ENSG00000101331 ENSG00000201085 ENSG00000187323 ENSG00000172733 ENSG00000138650 ENSG00000199700 | 1.164755428  0.8583959244  0.9623576631  1.299578573  1.164829631  1.056069125  1.950218636  2.101183024  1.285916693 | 2.066951702  2.797615689  2.128115821  2.873987125  3.383637586  3.394033039  2.224542281  2.04572492  2.839876603 | 1.579187041  2.138095841  1.62868791  2.200051741  2.591634154  2.599519671  1.704347149  1.568655339  2.179271002 | 1.308870735  1.308461312  1.306644329  1.306327061  1.305600013  1.305638529  1.305216653  1.304126451  1.30313146 | 0.1905781414  0.1907168886  0.1913335341  0.1914413583  0.1916886159  0.1916755114  0.1918190852  0.1921904715  0.1925298847 | 0.3151363986  0.3153289865  0.3160531638  0.3161943686  0.3164919484  0.3164919484  0.3166334865  0.3171007726  0.3175475421 | 0  0  0  0.9967698847  0  0  0  1.993539769  0 | 1.434605154  0  0  0  0  0  1.434605154  0.7173025769  0 | 0  0  0  0  0  0  0.780045262  0  0 | 0  0  0.9271856394  0  0  0  0  0  0.9271856394 | 0  0.9689801135  1.937960227  0  0  0  0.9689801135  0.9689801135  0 | 4.864624766  0  1.945849906  0  0  0  0  0  0 | 2.246828371  0  0.7489427904  0  0  0  0  0  0 | 3.07919764  0  1.847518584  0  0  0  0  0  0 | 0  2.788529585  0  2.788529585  0  1.394264792  5.57705917  8.365588755  5.57705917 | 1.390846572  0  0  5.563386288  6.95423286  6.95423286  6.95423286  2.781693144  5.563386288 | 0  4.14083481  4.14083481  0  4.14083481  0  0  4.14083481  0 | 0.9609626337  2.402406584  0  6.246257119  2.882887901  4.324331852  7.68770107  6.246257119  3.363369218 |
| 17169 ENSG00000254777 0.9838945647 3.339731675 2.564673676 1.302205308 0.1928462111 0.3179395918 0 0 0 0 0 2.91877486 1.497885581 7.390074335 0 0 0 0 | | | | | | | | | | | | | | | | | | | |
| 17172 | ENSG00000158553 | 0.8893350144 | 2.807046831 | 2.155991089 | 1.301975155 | 0.1929248791 | 0.3179952033 | 0 | 0 | 0 | 0 | 0 | 0 | 0 | 0 | 2.788529585 | 2.781693144 | 4.14083481 | 0.9609626337 |

| 17173  17176  17178  17197  17208  17218  17229  17244 | ENSG00000101746 ENSG00000261701 ENSG00000270137 ENSG00000226738 ENSG00000265118 ENSG00000232732 ENSG00000124212 ENSG00000102445 | 0.8893350144  1.681250017  0.9422215384  1.14161912  243.1516362  0.9356413905  0.9477454379  1.064439016 | 2.807046831  4.037337394  2.933820906  3.370745822  2.17416628  2.451977262  2.42787215  2.80528028 | 2.155991089  3.10167979  2.254099819  2.593754901  1.675372992  1.890373232  1.874112962  2.168771615 | 1.301975155  1.301661573  1.301548796  1.299562199  1.297720741  1.297086322  1.29547802  1.293488102 | 0.1929248791  0.1930321012  0.1930706733  0.1937510626  0.1943833139  0.1946014874  0.1951553788  0.1958422965 | 0.3179952033  0.3181163634  0.318142885  0.3189112972  0.3197474479  0.3199094052  0.3206261624  0.3214748344 | 0  0  0  0  110.6414572  0  0  0 | 0  0  0  0  40.88624688  0.7173025769  0  0 | 0  0  0  0  105.3061104  0  0.780045262  0 | 0  0  0  0  34.30586866  0  0  0 | 0  9.689801135  0  5.813880681  782.9359317  0  0  0 | 0  0  0.9729249532  0  519.541925  0  0  0 | 0  10.48519907  0  3.744713952  809.6071565  0  0.7489427904  0.7489427904 | 0  0  0.615839528  0  511.7626477  0.615839528  0  0 | 2.788529585  0  5.57705917  0  0  2.788529585  2.788529585  0 | 2.781693144  0  0  0  1.390846572  2.781693144  4.172539716  2.781693144 | 4.14083481  0  4.14083481  4.14083481  0  0  0  8.281669619 | 0.9609626337  0  0  0  1.441443951  4.324331852  2.882887901  0.9609626337 |
| --- | --- | --- | --- | --- | --- | --- | --- | --- | --- | --- | --- | --- | --- | --- | --- | --- | --- | --- | --- |
| 17248 ENSG00000246225 1.928419544 2.31144506 1.787381683 1.293201716 0.1959413022 0.3215693548 1.993539769 0 0 0 0 0 0.7489427904 0 4.182794377 1.390846572 12.42250443 2.402406584 | | | | | | | | | | | | | | | | | | | |
| 17256  17257  17276  17277  17279  17283  17284  17286  17287 | ENSG00000242279 ENSG00000093072 ENSG00000165092 ENSG00000224817 ENSG00000139988 ENSG00000233791 ENSG00000154783 ENSG00000163273 ENSG00000184221 | 0.7514746879  0.7386434504  1.238905882  1.021100019  1.310333211  1.020245464  1.020245464  1.027024015  1.255891719 | 2.148874547  2.063128976  3.305821463  3.35838791  2.871264847  3.357442175  3.357442175  3.354710163  2.809690783 | 1.662658343  1.596404495  2.563002954  2.603713299  2.226165803  2.603731273  2.603731273  2.601906125  2.179148889 | 1.292433023  1.292359789  1.289823508  1.289845511  1.289780322  1.289473384  1.289473384  1.289327901  1.289352369 | 0.1962072268  0.1962325755  0.1971119437  0.1971043028  0.197126942  0.1972335637  0.1972335637  0.1972841151  0.1972756124 | 0.3218498935  0.3218728215  0.3229222525  0.3229222525  0.3229281335  0.3230093296  0.3230093296  0.323036048  0.323036048 | 0  0  0  0  0  0  0  0  0.9967698847 | 0.7173025769  0  0  0  0  0  0  0  0 | 0  0  0  0  0.780045262  0  0  0  0 | 0  0.9271856394  0  0  0  0  0  0  0 | 3.875920454  1.937960227  0  0  0  0  0  0  0 | 0.9729249532  0  0  0  0  0  0  0  0 | 0  1.497885581  0  0  0  0  0  0  0 | 0.615839528  1.231679056  0  0  0  0  0  0.615839528  0 | 1.394264792  2.788529585  4.182794377  5.57705917  9.759853547  1.394264792  1.394264792  0  5.57705917 | 0  0  0  1.390846572  2.781693144  5.563386288  5.563386288  8.345079432  4.172539716 | 0  0  8.281669619  0  0  0  0  0  0 | 1.441443951  0.4804813169  2.402406584  5.285294486  2.402406584  5.285294486  5.285294486  3.363369218  4.324331852 |
| 17290 ENSG00000198692 0.7865573291 2.113521071 1.639459593 1.289157159 0.1973434553 0.3230771455 0.9967698847 0 0 0 1.937960227 0 0 1.847518584 1.394264792 2.781693144 0 0.4804813169 | | | | | | | | | | | | | | | | | | | |
| 17292  17294  17298  17305  17309  17313  17316  17340  17342 | ENSG00000162975 ENSG00000251385 ENSG00000181215 ENSG00000150873 ENSG00000149654 ENSG00000110169 ENSG00000253227 ENSG00000256603 ENSG00000235397 | 2.225741722  0.7231509305  0.8574429494  0.8289675422  1.047236649  1.479013655  1.981104271  1.906431026  0.7108838308 | 2.202467223  2.857218712  2.815514234  2.734424032  2.792397141  2.57477813  2.500285531  2.275748421  2.846384936 | 1.708930953  2.217530313  2.18596556  2.124253618  2.169499469  2.000835907  1.94390862  1.774104262  2.219242234 | 1.288798251  1.288468841  1.287995697  1.287239908  1.287115844  1.286851221  1.286215569  1.28275912  1.282593172 | 0.1974682343  0.1975828086  0.1977474612  0.1980106814  0.1980539137  0.1981461501  0.198367839  0.1995764804  0.1996346435 | 0.3232405909  0.3233941819  0.3235888331  0.3238513957  0.3238843424  0.323965289  0.3242665772  0.3257780097  0.3258481271 | 0.9967698847  0  0  0  0  0  0  1.993539769  0 | 1.434605154  0  0  0  0  0  0  0  0 | 0  0  0  0  0  0.780045262  0  0  0 | 0  0  0  0  0  0  1.854371279  0  0 | 0  0  0  0  0.9689801135  0  0  0.9689801135  0 | 0  0  0  0  0  0  0  0  0.9729249532 | 0  0.7489427904  0  0  0  0.7489427904  0  0  0.7489427904 | 0  0  0  0.615839528  0  0  0  0  1.231679056 | 5.57705917  5.57705917  1.394264792  2.788529585  1.394264792  1.394264792  2.788529585  2.788529585  5.57705917 | 4.172539716  1.390846572  1.390846572  0  0  0  5.563386288  2.781693144  0 | 8.281669619  0  4.14083481  4.14083481  8.281669619  12.42250443  8.281669619  12.42250443  0 | 6.246257119  0.9609626337  3.363369218  2.402406584  1.921925267  2.402406584  5.285294486  1.921925267  0 |
| 17347 ENSG00000223979 1.053916781 2.774389273 2.163734547 1.282222571 0.1997645807 0.3260159334 0 0 0 0 0 0 0 0.615839528 2.788529585 0 8.281669619 0.9609626337 | | | | | | | | | | | | | | | | | | | |
| 17354  17359  17383  17391  17408  17412  17414  17415  17426 | ENSG00000165973 ENSG00000232893 ENSG00000234883 ENSG00000158748 ENSG00000251323 ENSG00000145832 ENSG00000275291 ENSG00000201699 ENSG00000230113 | 0.8535113528  0.712058992  1.818922254  1.124789521  0.8002072834  1.780679367  1.378639743  1.378639743  0.8499811751 | 2.757261304  2.836867755  2.481050565  3.317088269  2.646008266  4.005319377  2.220917328  2.220917328  2.341525426 | 2.15154314  2.214618094  1.941065113  2.597889501  2.075840423  3.143228915  1.743193719  1.743193719  1.840578449 | 1.281527316  1.2809738  1.278190282  1.27683963  1.274668436  1.274269067  1.274050786  1.274050786  1.272168229 | 0.2000085117  0.200202869  0.2011823419  0.2016588712  0.2024266233  0.2025680751  0.2026454182  0.2026454182  0.2033133532 | 0.3262326227  0.3264555798  0.3275998026  0.3282247143  0.3291525734  0.329306911  0.3293718661  0.3293718661  0.3302669232 | 0  0  1.993539769  0  0  0  0  0  0 | 0  0  0  0  0  0  0.7173025769  0.7173025769  0 | 0  0  0  0  0  0  0.780045262  0.780045262  0.780045262 | 0  0  0  0  0  0  0  0  0 | 0  0  0  0  0  0  0.9689801135  0.9689801135  4.844900568 | 0  0  0  0  0  0  0  0  0 | 0  0  0  0  0  0  0  0  2.246828371 | 0  0.615839528  0.615839528  0  1.231679056  0  0  0  1.847518584 | 2.788529585  5.57705917  9.759853547  0  2.788529585  0  6.971323962  6.971323962  0 | 1.390846572  1.390846572  4.172539716  6.95423286  0  0  2.781693144  2.781693144  0 | 4.14083481  0  0  4.14083481  4.14083481  16.56333924  0  0  0 | 1.921925267  0.9609626337  5.285294486  2.402406584  1.441443951  4.804813169  4.324331852  4.324331852  0.4804813169 |
| 17428 ENSG00000254397 1.000652392 2.043135357 1.606134691 1.272082204 0.2033439134 0.3302786356 0.9967698847 0 0 0 0.9689801135 0.9729249532 0.7489427904 0 2.788529585 1.390846572 4.14083481 0 | | | | | | | | | | | | | | | | | | | |
| 17429  17434  17441  17452  17454  17468  17483  17485  17486 | ENSG00000253508 ENSG00000221585 ENSG00000173114 ENSG00000279414 ENSG00000078549 ENSG00000241202 ENSG00000266835 ENSG00000253569 ENSG00000277504 | 0.9726270128  1.379701929  0.9020726224  0.7514173518  1.270513095  1.013236826  0.8226161478  1.202797369  1.202797369 | 3.266254549  2.091617737  3.21465617  2.120917909  3.348020451  3.324906607  2.751672678  3.244489102  3.244489102 | 2.567826185  1.644987412  2.529722859  1.6708998  2.638410441  2.623104219  2.174002614  2.563751155  2.563751155 | 1.271992071  1.271509874  1.270754288  1.269326808  1.268953609  1.267546513  1.265717281  1.265524189  1.265524189 | 0.2033759363  0.203547317  0.2038160757  0.2043245289  0.2044576104  0.2049599431  0.205614319  0.2056834829  0.2056834829 | 0.3302845871  0.3304623088  0.3307978147  0.3314009921  0.3315788426  0.3320890755  0.3329016138  0.3329564604  0.3329564604 | 0  0.9967698847  0  0  0  0  0  0  0 | 0  0  0  0.7173025769  0  0  0  0  0 | 0  0  0  0  0  0  0  0  0 | 0  0.9271856394  0  0  0  0  0  0  0 | 0  0  0  0.9689801135  0  0  0  0  0 | 0  3.891699813  0  0  0  0  0.9729249532  0  0 | 0  0  0  0.7489427904  0  0  0  0  0 | 0  1.847518584  1.847518584  0  0  0  0  0  0 | 5.57705917  6.971323962  0  2.788529585  4.182794377  8.365588755  1.394264792  2.788529585  2.788529585 | 4.172539716  0  4.172539716  1.390846572  2.781693144  1.390846572  0  0  0 | 0  0  0  0  8.281669619  0  4.14083481  8.281669619  8.281669619 | 1.921925267  1.921925267  4.804813169  2.402406584  0  2.402406584  3.363369218  3.363369218  3.363369218 |
| 17490 ENSG00000253730 0.7680142603 2.126935331 1.681182545 1.265142406 0.2058202838 0.3330826684 0 0.7173025769 0 0 0 0.9729249532 2.246828371 0.615839528 4.182794377 0 0 0.4804813169 | | | | | | | | | | | | | | | | | | | |
| 17514  17528  17534  17537  17550  17556  17563  17581  17597 | ENSG00000134802 ENSG00000125285 ENSG00000183454 ENSG00000271964 ENSG00000254979 ENSG00000226308 ENSG00000278464 ENSG00000174016 ENSG00000270350 | 2553.110492  0.8509058983  0.8544643279  0.8539385094  1949.940646  1.233834878  0.9087602003  0.9362336479  1.742546139 | 2.088307643  3.152281236  2.742523642  2.741642654  2.065901432  3.280700131  3.208167637  3.225946752  4.062667239 | 1.6545448  2.50124312  2.177316512  2.177157431  1.642355915  2.610354031  2.5546741  2.571906281  3.242848735 | 1.26216446  1.26028582  1.259588869  1.259276254  1.257888996  1.256802752  1.255803093  1.254301829  1.252808124 | 0.2068896144  0.2075662736  0.207817713  0.2079305673  0.2084319039  0.2088250692  0.2091873698  0.2097323193  0.2102755445 | 0.3343734969  0.3352183523  0.3354903721  0.3355959994  0.3361751266  0.336694145  0.3371438643  0.3376951133  0.3382428575 | 606.0360899  0  0  0  475.459235  0  0  0  0 | 1003.506305  0  0  0  773.9694805  0  0  0  0 | 616.235757  0  0  0  457.8865688  0  0  0  0 | 997.651748  0  0  0  789.0349791  0  0  0  0 | 7232.467567  0  0.9689801135  0  5431.133536  0  0  0  0 | 6499.138688  4.864624766  0  0.9729249532  5044.615883  0  0  0  0 | 7187.60396  0  0  0  5423.094746  0  1.497885581  0  0 | 6466.930883  2.463358112  0  0  4981.525942  0  0  0  0 | 5.57705917  0  4.182794377  0  4.182794377  0  0  2.788529585  19.51970709 | 11.12677258  0  0  4.172539716  9.735926004  5.563386288  5.563386288  5.563386288  1.390846572 | 0  0  4.14083481  4.14083481  0  8.281669619  0  0  0 | 11.05107029  2.882887901  0.9609626337  0.9609626337  8.648663704  0.9609626337  3.843850535  2.882887901  0 |
| 17608 ENSG00000270988 0.7085623613 2.320894285 1.853641343 1.252073004 0.2105432634 0.3384619272 0 0 0 0 0 0 0 0.615839528 1.394264792 1.390846572 4.14083481 0.9609626337 | | | | | | | | | | | | | | | | | | | |
| 17623  17639  17643  17671  17681  17705  17710  17719  17724 | ENSG00000236182 ENSG00000277020 ENSG00000206432 ENSG00000254980 ENSG00000269495 ENSG00000196711 ENSG00000230520 ENSG00000248746 ENSG00000258642 | 0.9753112847  0.8520373423  1.053996753  0.890616726  1.501866188  0.9323020513  0.8466635706  0.8546054113  1.078497925 | 2.604869665  2.326931913  3.222383005  2.806046026  3.878054774  3.206085736  2.755456539  2.242798299  2.346945606 | 2.085313573  1.867412111  2.586384499  2.258226284  3.124840282  2.588482841  2.225673899  1.813253742  1.89867599 | 1.249150104  1.246073055  1.245902535  1.242588507  1.241040957  1.23859648  1.238032463  1.236891587  1.2360959 | 0.2116101779  0.2127375761  0.2128001792  0.2140195074  0.2145906192  0.2154949722  0.215704024  0.2161273337  0.2164229188 | 0.339887516  0.341388391  0.3414114307  0.342842454  0.3435527424  0.3445047262  0.3447610448  0.3452621654  0.345636828 | 0  0.9967698847  0  0  0  0  0  0.9967698847  0.9967698847 | 0  0  0  0  0  0  0  0  0 | 0  0  0  0  0  0  0  0  0 | 0  0  0  0  0  0  0  0  0 | 0  0  0  0  0  0  3.875920454  0  0 | 0  0  0  0.9729249532  0  0  0  0  0 | 0.7489427904  0  0  0  0  0  0.7489427904  2.246828371  0.7489427904 | 1.231679056  3.07919764  0  0  0  0  0  0  0 | 0  1.394264792  4.182794377  4.182794377  6.971323962  4.182794377  1.394264792  2.788529585  0 | 0  1.390846572  0  1.390846572  0  5.563386288  0  2.781693144  4.172539716 | 8.281669619  0  4.14083481  4.14083481  0  0  4.14083481  0  4.14083481 | 1.441443951  3.363369218  4.324331852  0  11.05107029  1.441443951  0  1.441443951  2.882887901 |
| 17726 ENSG00000173930 1.650365641 2.368997635 1.917086858 1.235727857 0.2165597391 0.3457968058 1.993539769 0 0 0 5.813880681 3.891699813 7.489427904 0.615839528 0 0 0 0 | | | | | | | | | | | | | | | | | | | |
| 17734 | ENSG00000101204 | 1.347096684 | 3.260159316 | 2.639493751 | 1.235145684 | 0.2167762896 | 0.3460311969 | 0 | 0 | 0 | 0 | 0 | 0 | 0 | 0 | 0 | 2.781693144 | 12.42250443 | 0.9609626337 |

| 17740  17741  17742  17773  17777  17782  17808  17812 | ENSG00000165556 ENSG00000258743 ENSG00000261649 ENSG00000267385 ENSG00000221500 ENSG00000102387 ENSG00000211714 ENSG00000224215 | 1.04892575  1.04892575  0.7889274325  0.8202296465  1.011134085  1.613849438  0.8027329984  1.162187555 | 3.196380022  3.196380022  2.620090408  2.272977724  2.452446417  2.333519112  2.276680821  3.146840317 | 2.589109355  2.589109355  2.122476619  1.846096708  1.992603504  1.89643134  1.853754517  2.563612101 | 1.234548095  1.234548095  1.234449597  1.231234373  1.23077492  1.230479091  1.228145798  1.227502521 | 0.2169987367  0.2169987367  0.2170354172  0.2182352189  0.2184070584  0.218517752  0.2193922413  0.2196337748 | 0.3462048194  0.3462048194  0.3462048194  0.3475702529  0.3477656632  0.3478049644  0.3487262344  0.3490317556 | 0  0  0  0.9967698847  0  0  0  0 | 0  0  0  0  0  0  0  0 | 0  0  0  0  0  0  0.780045262  0 | 0  0  0  0  0.9271856394  1.854371279  0  0 | 0  0  0  1.937960227  0  0  1.937960227  0 | 0  0  0  0.9729249532  0.9729249532  1.945849906  0.9729249532  0 | 0  0  0  4.493656743  0  0  2.246828371  0 | 0  0  0.615839528  0  0  0  3.695037168  0 | 0  0  2.788529585  0  6.971323962  5.57705917  0  0 | 5.563386288  5.563386288  0  0  2.781693144  2.781693144  0  2.781693144 | 4.14083481  4.14083481  4.14083481  0  0  0  0  8.281669619 | 2.882887901  2.882887901  1.921925267  1.441443951  0.4804813169  7.207219753  0  2.882887901 |
| --- | --- | --- | --- | --- | --- | --- | --- | --- | --- | --- | --- | --- | --- | --- | --- | --- | --- | --- | --- |
| 17825 ENSG00000279699 0.7920591699 2.239662577 1.826968258 1.225890251 0.2202399779 0.3497398494 0 0 0 0.9271856394 1.937960227 0.9729249532 3.744713952 0 0 0 0 1.921925267 | | | | | | | | | | | | | | | | | | | |
| 17835  17836  17837  17838  17861  17875  17884  17920  17923 | ENSG00000145808 ENSG00000183908 ENSG00000178363 ENSG00000180440 ENSG00000237202 ENSG00000273727 ENSG00000244573 ENSG00000277968 ENSG00000270154 | 0.8134712431  0.8134712431  1.69449523  0.8134712431  1.081621175  1.298559523  0.8783569817  0.7407764501  1.019385321 | 2.642973105  2.642973105  2.323135977  2.642973105  2.671727429  2.11867378  3.184286937  2.18874843  2.31346558 | 2.157781197  2.157781197  1.896513135  2.157781197  2.185638932  1.737302174  2.613449308  1.80514596  1.908548802 | 1.224856862  1.224856862  1.224951166  1.224856862  1.222401098  1.219519443  1.218423073  1.21250496  1.21215951 | 0.2206291563  0.2206291563  0.2205936205  0.2206291563  0.2215559844  0.2226471009  0.2230632416  0.2253191451  0.2254513275 | 0.3501025282  0.3501025282  0.3501025282  0.3501025282  0.3511205381  0.3525411176  0.3530545804  0.3559086898  0.3560380092 | 0  0  0.9967698847  0  0  0  0  0  0 | 0  0  0  0  0.7173025769  0.7173025769  0  0.7173025769  0.7173025769 | 0  0  0.780045262  0  0  0.780045262  0  0  0 | 0  0  0  0  0  0  0  0  0 | 0  0  0  0  0  0.9689801135  0  2.906940341  0 | 0  0  0  0  5.837549719  0  4.864624766  0  0 | 0  0  0  0  1.497885581  0  0.7489427904  2.246828371  0.7489427904 | 0  0  0  0  4.926716224  0  4.926716224  0.615839528  0 | 2.788529585  2.788529585  11.15411834  2.788529585  0  6.971323962  0  0  0 | 1.390846572  1.390846572  2.781693144  1.390846572  0  2.781693144  0  0  2.781693144 | 4.14083481  4.14083481  4.14083481  4.14083481  0  0  0  0  4.14083481 | 1.441443951  1.441443951  0.4804813169  1.441443951  0  3.363369218  0  2.402406584  3.843850535 |
| 17934 ENSG00000280587 0.7954712543 2.240852736 1.850445268 1.210980284 0.2259029611 0.3565523149 0 0 0.780045262 0 3.875920454 1.945849906 0 2.463358112 0 0 0 0.4804813169 | | | | | | | | | | | | | | | | | | | |
| 17943  17945  17946  17948  17955  17964  17965  17969  17976 | ENSG00000133665 ENSG00000269699 ENSG00000278921 ENSG00000274173 ENSG00000203650 ENSG00000134595 ENSG00000284713 ENSG00000264078 ENSG00000260886 | 0.8606547283  0.8606547283  0.8603698766  1.198944319  2.154349639  1.152964962  0.7698806945  1.230017642  0.7601671737 | 3.122687556  3.122687556  3.122318879  2.031918611  2.11412526  2.686544988  2.210130951  2.770204661  2.523121173 | 2.582530529  2.582530529  2.582540463  1.680920052  1.750691193  2.227242532  1.832307957  2.298117347  2.095190796 | 1.209158041  1.209158041  1.209010633  1.208813357  1.207594616  1.206220225  1.206200597  1.205423502  1.204244109 | 0.2266021342  0.2266021342  0.2266587605  0.226734559  0.2272032311  0.2277325877  0.2277401538  0.2280398532  0.2284952421 | 0.3574366124  0.3574366124  0.3575060111  0.3575857157  0.3581452725  0.3588317725  0.3588317725  0.3592240017  0.3598011972 | 0  0  0  0  1.993539769  0  0  0  0 | 0  0  0  1.434605154  0  0  0  0  0 | 0  0  0  0  0.780045262  0.780045262  0.780045262  0  0 | 0  0  0  0  0  0  0  0  0 | 0  0  0  0.9689801135  0  0  1.937960227  0  0 | 0  0  0  0  0  0  0  0.9729249532  0 | 0  0  0  0.7489427904  0  0  2.246828371  0.7489427904  0 | 0  0  0  0  0.615839528  0  0  0.615839528  1.231679056 | 4.182794377  4.182794377  2.788529585  2.788529585  2.788529585  2.788529585  0  0  2.788529585 | 2.781693144  2.781693144  4.172539716  5.563386288  13.90846572  8.345079432  1.390846572  0  0 | 0  0  0  0  0  0  0  12.42250443  4.14083481 | 3.363369218  3.363369218  3.363369218  2.882887901  5.765775802  1.921925267  2.882887901  0  0.9609626337 |
| 17999 ENSG00000253986 1.536129458 2.185554708 1.819847191 1.200955069 0.229768631 0.3613440118 0 0 1.560090524 0 0 0.9729249532 0 0.615839528 6.971323962 4.172539716 4.14083481 0 | | | | | | | | | | | | | | | | | | | |
| 18013  18014  18022  18025  18026  18044  18055  18068  18079 | ENSG00000205890 ENSG00000226947 ENSG00000108242 ENSG00000232237 ENSG00000261586 ENSG00000285476 ENSG00000224760 ENSG00000205869 ENSG00000254187 | 0.7783157049  0.8564382801  0.7203224232  0.8689377728  1.620901673  1.4228452  1.082242073  1.080532963  1.037216983 | 2.568637242  3.099635462  2.318954679  2.386819027  2.429472289  2.086301179  3.23185081  3.229212735  2.610473236 | 2.142773521  2.585823289  1.935786583  1.993480613  2.029072156  1.745590903  2.706572966  2.706419209  2.190372263 | 1.198744159  1.198703514  1.197939225  1.197312385  1.197331638  1.195183347  1.194074888  1.193167978  1.191794326 | 0.2306274417  0.2306432513  0.2309406777  0.2311848189  0.2311773174  0.2320154033  0.2324486754  0.232803593  0.2333419011 | 0.3624127222  0.3624174459  0.3627237167  0.3630064617  0.3630064617  0.3639674133  0.3644249352  0.3647187571  0.3653194608 | 0  0  0  0  0  0  0  0  0 | 0  0  0  0  0.7173025769  0.7173025769  0  0  0.7173025769 | 0  0  0  0.780045262  0  0  0  0  0 | 0  0  0  0  0.9271856394  0.9271856394  0  0  0 | 0.9689801135  0  0.9689801135  0  0  0  0  0  1.937960227 | 0  0  0  0  0  0  0  0  4.864624766 | 0  0  0.7489427904  0  0  0  0  0  0 | 0  0  0  0.615839528  0  0  0  0  4.926716224 | 2.788529585  4.182794377  1.394264792  1.394264792  1.394264792  4.182794377  8.365588755  0  0 | 0  4.172539716  1.390846572  1.390846572  11.12677258  2.781693144  0  8.345079432  0 | 4.14083481  0  4.14083481  0  0  4.14083481  4.14083481  4.14083481  0 | 1.441443951  1.921925267  0  6.246257119  5.285294486  4.324331852  0.4804813169  0.4804813169  0 |
| 18080 ENSG00000139209 53.56231955 2.114809676 1.774446227 1.191813899 0.2333342248 0.3653194608 19.93539769 13.62874896 16.3809505 16.68934151 144.3780369 159.5596923 128.0692172 144.1064495 0 0 0 0 | | | | | | | | | | | | | | | | | | | |
| 18084  18096  18113  18120  18132  18170  18171  18183  18188 | ENSG00000271404 ENSG00000260498 ENSG00000238015 ENSG00000130234 ENSG00000188095 ENSG00000153266 ENSG00000073067 ENSG00000107807 ENSG00000283265 | 1.056221092  0.7913880094  0.9774833058  0.7819522932  1.257791124  0.8206146186  1.234249328  0.7705149476  1.002311968 | 2.29780498  2.251769766  2.564235135  3.021774569  2.116570176  3.056003259  2.287853853  2.58521472  2.5997679 | 1.929249514  1.893443683  2.160864721  2.548089338  1.786957126  2.591436266  1.940072112  2.195601516  2.20925532 | 1.191035666  1.189245704  1.186670831  1.185898204  1.184454929  1.179270082  1.179262276  1.177451692  1.176762086 | 0.2336395835  0.2343429945  0.2353574848  0.2356625026  0.2362330275  0.2382906474  0.238293755  0.2390152681  0.2392904793 | 0.3657046036  0.3665623785  0.3678037302  0.3681381235  0.3687851355  0.3712037328  0.3712037328  0.3720896025  0.3724079781 | 0  0  0  0  0  0  0  0  0 | 0  0.7173025769  0  0  0.7173025769  0  0  0  0 | 0.780045262  0  0  0  0  0  0  0  0 | 0  0  0  0  0.9271856394  0  0.9271856394  0  0 | 0  0  0  0  0  0  0  3.875920454  0 | 0  0.9729249532  0  0  0.9729249532  0  0.9729249532  0  0 | 0.7489427904  4.493656743  0  0  0  0  0  0.7489427904  0 | 0  0  0.615839528  1.847518584  0.615839528  0  1.847518584  0  0 | 0  0  0  0  0  4.182794377  0  0  1.394264792 | 5.563386288  1.390846572  1.390846572  4.172539716  4.172539716  2.781693144  2.781693144  0  1.390846572 | 4.14083481  0  8.281669619  0  0  0  8.281669619  4.14083481  8.281669619 | 1.441443951  1.921925267  1.441443951  3.363369218  7.68770107  2.882887901  0  0.4804813169  0.9609626337 |
| 18198 ENSG00000237870 31.58219844 2.086434509 1.775513123 1.175116355 0.2399481682 0.3732263353 7.974159078 12.19414381 7.80045262 12.05341331 17.44164204 161.5055422 20.22145534 139.7955728 0 0 0 0 | | | | | | | | | | | | | | | | | | | |
| 18226  18246  18247  18248  18256  18260  18276  18280  18287 | ENSG00000111218 ENSG00000180318 ENSG00000113525 ENSG00000260278 ENSG00000158113 ENSG00000156413 ENSG00000171560 ENSG00000085563 ENSG00000162624 | 0.8570079835  0.8242613634  0.7790715981  0.7928898448  1.067296064  0.9688455305  1.306778622  1.704035282  0.8163981703 | 3.101567798  3.078997615  2.549330564  2.142298706  2.613393067  3.036252096  3.681095173  2.282713269  3.03136248 | 2.648131628  2.635587254  2.182635701  1.834317746  2.238934524  2.603131895  3.161513546  1.961507285  2.607005416 | 1.171228713  1.168239682  1.168005528  1.16789946  1.167248545  1.16638427  1.164345849  1.163754673  1.162775674 | 0.2415068569  0.2427100983  0.2428045352  0.2428473219  0.2431100114  0.2434591153  0.2442838831  0.2445234457  0.2449205293 | 0.3750736909  0.3765292142  0.376655076  0.3767008052  0.3769430316  0.3774016275  0.3783486318  0.3786160853  0.3790738764 | 0  0  0  0  0  0  0  0  0 | 0  0  0  0  0  0  0  0  0 | 0  0  0  0  0.780045262  0  0  0  0 | 0  0  0  0.9271856394  0  0  0  1.854371279  0 | 0  0  0  0  0  0  9.689801135  0  0 | 0  0  1.945849906  1.945849906  0  0  0  0  0 | 0  0  0  1.497885581  0.7489427904  0  5.991542324  0  0 | 0  0  0  0  0  0  0  0  0 | 6.971323962  1.394264792  0  4.182794377  0  0  0  5.57705917  4.182794377 | 1.390846572  4.172539716  2.781693144  0  6.95423286  5.563386288  0  6.95423286  4.172539716 | 0  0  4.14083481  0  0  4.14083481  0  4.14083481  0 | 1.921925267  4.324331852  0.4804813169  0.9609626337  4.324331852  1.921925267  0  1.921925267  1.441443951 |
| 18288 ENSG00000225650 0.7952332194 2.5839868 2.222284249 1.162761605 0.2449262392 0.3790738764 0 0 0 0 0 0 0.7489427904 0 0 4.172539716 4.14083481 0.4804813169 | | | | | | | | | | | | | | | | | | | |
| 18289  18294  18309  18312  18315  18326  18353  18367  18369 | ENSG00000144015 ENSG00000259954 ENSG00000116981 ENSG00000205867 ENSG00000174600 ENSG00000229391 ENSG00000225480 ENSG00000204099 ENSG00000129673 | 0.7952332194  1.789592857  0.8208994703  0.8200449152  169.5912103  0.7927021193  0.8751633247  0.7238707481  0.7412479235 | 2.5839868  2.057142147  3.056798774  3.055634332  2.108768688  3.039198742  3.115239744  2.474785873  2.970778248 | 2.222284249  1.770331774  2.636612992  2.636664829  1.82035154  2.627523916  2.701398217  2.1503841  2.581743705 | 1.162761605  1.162009391  1.15936574  1.158901313  1.158440357  1.156677861  1.153195306  1.150857595  1.15068674 | 0.2449262392  0.2452316502  0.2463071353  0.2464964137  0.2466843779  0.2474039983  0.2488302297  0.2497908257  0.2498611334 | 0.3790738764  0.3794727457  0.3807946786  0.3810248737  0.3812574086  0.3821356311  0.3837731424  0.3849610232  0.3849858011 | 0  0  0  0  28.90632666  0  0  0  0 | 0  1.434605154  0  0  63.12262677  0  0  0  0 | 0  0  0  0  49.92289677  0  0  0  0 | 0  0.9271856394  0  0  69.53892296  0  0  0  0 | 0  6.782860795  0  0  681.1930198  6.782860795  0  0  1.937960227 | 0  0  0  0  215.9893396  0  0  0  0 | 0.7489427904  11.23414186  0  0  712.2445937  1.497885581  0.7489427904  0.7489427904  4.493656743 | 0  0.615839528  0  0  213.6963162  1.231679056  0  0  2.463358112 | 0  0  5.57705917  1.394264792  0  0  6.971323962  1.394264792  0 | 4.172539716  0  1.390846572  5.563386288  0  0  2.781693144  0  0 | 4.14083481  0  0  0  0  0  0  4.14083481  0 | 0.4804813169  0.4804813169  2.882887901  2.882887901  0.4804813169  0  0  2.402406584  0 |
| 18370 ENSG00000227401 0.7602335199 2.21000228 1.920557146 1.150708941 0.249851997 0.3849858011 0 0.7173025769 0 0 1.937960227 0 0.7489427904 0 1.394264792 0 0 4.324331852 | | | | | | | | | | | | | | | | | | | |
| 18371 | ENSG00000128253 | 1.023799105 | 2.526793319 | 2.195823676 | 1.150726876 | 0.2498446161 | 0.3849858011 | 0.9967698847 | 0 | 0 | 0 | 0 | 0 | 0 | 0 | 4.182794377 | 2.781693144 | 0 | 4.324331852 |

| 18399  18405  18418  18431  18452  18464  18466  18467 | ENSG00000235196 ENSG00000159516 ENSG00000201643 ENSG00000232792 ENSG00000235123 ENSG00000217258 ENSG00000178217 ENSG00000266925 | 0.7805745088  1.257693933  0.9967393467  1.394187065  1.006761574  0.7884377018  0.8527915353  0.8527915353 | 2.985539959  3.625985713  2.200611335  2.664043368  3.094407221  3.034734475  3.080268277  3.080268277 | 2.602927636  3.162677281  1.920490411  2.328276401  2.709344767  2.660945078  2.700738685  2.700738685 | 1.14699307  1.146492478  1.145859059  1.144212675  1.14212383  1.140472421  1.140528069  1.140528069 | 0.2513844852  0.2515914378  0.2518534735  0.2525354479  0.2534025516  0.2540895363  0.2540663656  0.2540663656 | 0.386770471  0.3869354653  0.3870798153  0.3878183805  0.3887441245  0.3894156254  0.3894156254  0.3894156254 | 0  0  0.9967698847  0.9967698847  0  0  0  0 | 0  0  0  0  0  0  0  0 | 0  0  0  0  0  0  0  0 | 0  0  0  0  0  0  0  0 | 0  0  4.844900568  0  0.9689801135  0  0  0 | 0  0  0  0  0  0  0  0 | 0  0  1.497885581  0  0  0  0  0 | 0  0  0  0  0  0  0  0 | 4.182794377  8.365588755  0  6.971323962  6.971323962  1.394264792  6.971323962  6.971323962 | 2.781693144  0  0  0  0  2.781693144  2.781693144  2.781693144 | 0  0  4.14083481  8.281669619  4.14083481  0  0  0 | 2.402406584  6.726738436  0.4804813169  0.4804813169  0  5.285294486  0.4804813169  0.4804813169 |
| --- | --- | --- | --- | --- | --- | --- | --- | --- | --- | --- | --- | --- | --- | --- | --- | --- | --- | --- | --- |
| 18478 ENSG00000148948 0.7842212536 3.010695576 2.642453677 1.139356047 0.2545546804 0.3899461404 0 0 0 0 0 0 0 0 1.394264792 4.172539716 0 3.843850535 | | | | | | | | | | | | | | | | | | | |
| 18484  18495  18496  18497  18498  18501  18507  18520  18573 | ENSG00000276070 ENSG00000184599 ENSG00000144619 ENSG00000230077 ENSG00000100191 ENSG00000181908 ENSG00000236283 ENSG00000267052 ENSG00000166143 | 0.7846045253  1.013499118  0.7704845299  0.7704845299  0.7704845299  1.188980672  1.016164015  0.7373226201  0.7597104805 | 3.004614008  2.500013385  3.001328395  3.001328395  3.001328395  2.59581009  2.541150918  2.463679177  2.957981075 | 2.638255956  2.197158493  2.638305513  2.638305513  2.638305513  2.283593614  2.23679881  2.171529742  2.6252339 | 1.138863726  1.137839347  1.137596984  1.137596984  1.137596984  1.136721558  1.136065929  1.134536235  1.126749534 | 0.2547599972  0.2551875716  0.2552888066  0.2552888066  0.2552888066  0.2556547044  0.2559289728  0.2565696834  0.2598483928 | 0.3901389876  0.3905563342  0.3906479057  0.3906479057  0.3906479057  0.3911443739  0.391409643  0.3921415474  0.3960146254 | 0  0  0  0  0  0  0  0  0 | 0  0  0  0  0  0  0  0  0 | 0  0  0  0  0  0  0.780045262  0  0 | 0  0.9271856394  0  0  0  0  0  0  0 | 0.9689801135  0  0  0  0  0  0  0  1.937960227 | 0  0  0  0  0  0  0  0  0 | 0  0  0.7489427904  0.7489427904  0.7489427904  0.7489427904  0  0  2.995771162 | 0  0  0  0  0  0.615839528  0.615839528  0  0 | 0  2.788529585  0  0  0  0  0  1.394264792  4.182794377 | 5.563386288  5.563386288  4.172539716  4.172539716  4.172539716  0  6.95423286  1.390846572  0 | 0  0  0  0  0  12.42250443  0  4.14083481  0 | 2.882887901  2.882887901  4.324331852  4.324331852  4.324331852  0.4804813169  3.843850535  1.921925267  0 |
| 18576 ENSG00000268955 0.9393051019 2.127408125 1.889249925 1.126059658 0.2601402682 0.3964002171 0 0.7173025769 0 0 0 0 0.7489427904 0 0 2.781693144 4.14083481 2.882887901 | | | | | | | | | | | | | | | | | | | |
| 18583  18611  18629  18666  18667  18680  18681  18692  18711 | ENSG00000095917 ENSG00000253721 ENSG00000284082 ENSG00000226870 ENSG00000234442 ENSG00000139292 ENSG00000276490 ENSG00000199568 ENSG00000148584 | 0.8971982074  0.977960308  0.7412783883  0.9646290822  0.9646290822  0.9705320808  0.7740992567  0.7405343991  1.144524626 | 2.923543307  2.46460766  2.082795421  3.011860077  3.011860077  2.116448708  2.502648455  2.910872129  2.500835965 | 2.598505807  2.196244003  1.860203363  2.702590419  2.702590419  1.901321702  2.248414981  2.618348858  2.253299437 | 1.125086309  1.122192095  1.119660067  1.114434528  1.114434528  1.113146032  1.113072309  1.111720511  1.109855141 | 0.2605524623  0.2617807729  0.2628586483  0.2650928178  0.2650928178  0.2656457166  0.2656773753  0.2662583382  0.2670614535 | 0.3968728866  0.3981498338  0.3994188573  0.4020075809  0.4020075809  0.4025357417  0.402562164  0.4032050353  0.4040105554 | 0  0  0  0  0  0  0  0  0 | 0  0  0.7173025769  0  0  0  0  0  0.7173025769 | 0  0  0  0  0  0  0  0  0 | 0  0.9271856394  0  0  0  0.9271856394  0  0  0 | 0  0  1.937960227  0  0  0  0.9689801135  0  0 | 0  0  0  0  0  0  0  0  0 | 0  0  0  0  0  0  0  0  0 | 0  0  0.615839528  0  0  0  0  0  0 | 0  4.182794377  4.182794377  0  0  1.394264792  2.788529585  4.182794377  0 | 2.781693144  2.781693144  0  6.95423286  6.95423286  2.781693144  1.390846572  2.781693144  6.95423286 | 4.14083481  0  0  4.14083481  4.14083481  4.14083481  4.14083481  0  4.14083481 | 3.843850535  3.843850535  1.441443951  0.4804813169  0.4804813169  2.402406584  0  1.921925267  1.921925267 |
| 18715 ENSG00000260905 0.7447508473 2.939250763 2.65065382 1.108877644 0.2674829693 0.4045617382 0 0 0 0 0 0 0 0 4.182794377 1.390846572 0 3.363369218 | | | | | | | | | | | | | | | | | | | |
| 18718  18767  18768  18772  18774  18781  18804  18818  18832 | ENSG00000186998 ENSG00000259200 ENSG00000254872 ENSG00000261754 ENSG00000227066 ENSG00000224221 ENSG00000232618 ENSG00000230345 ENSG00000096996 | 0.7441811439  0.7610791417  0.8997320998  0.749936008  0.7162028228  1.090025294  1.263869544  0.776927764  0.7557628131 | 2.938418242  2.170334594  2.415711593  2.92763753  2.926538974  2.926283482  2.441477721  2.96226917  2.45204053 | 2.650710646  1.968130927  2.191151291  2.65682888  2.656183623  2.658249413  2.223992398  2.701779141  2.240496499 | 1.108539797  1.10273893  1.102485074  1.101929278  1.101783382  1.100831046  1.097790498  1.096414257  1.094418372 | 0.2676287618  0.2701405553  0.270250844  0.2704924188  0.2705558568  0.270970197  0.2722959818  0.2728975275  0.2737715301 | 0.4047199344  0.4074656269  0.4075937974  0.4078712127  0.407923409  0.4083903624  0.4098491015  0.4104919445  0.4115004742 | 0  0  0  0  0  0  0  0  0 | 0  0  0.7173025769  0  0  0  0  0  0 | 0  0  0  0  0  0  0.780045262  0  0 | 0  0.9271856394  0  0  0  0  0  0  0 | 0  0  3.875920454  1.937960227  0  0  0  0  0 | 0  0  0  0  2.91877486  0  0  0  0 | 0  0  5.242599533  1.497885581  0.7489427904  0  0  0  0.7489427904 | 0  0.615839528  0  0  4.926716224  0.615839528  0  0  0 | 1.394264792  1.394264792  0  0  0  4.182794377  4.182794377  6.971323962  2.788529585 | 4.172539716  1.390846572  0  5.563386288  0  0  0  1.390846572  1.390846572 | 0  0  0  0  0  8.281669619  8.281669619  0  4.14083481 | 3.363369218  4.804813169  0.9609626337  0  0  0  1.921925267  0.9609626337  0 |
| 18841 ENSG00000206633 1.162640447 2.024382192 1.851540718 1.09335008 0.2742401224 0.4120275603 0 1.434605154 0 0 0 0 0 1.231679056 2.788529585 4.172539716 0 4.324331852 | | | | | | | | | | | | | | | | | | | |
| 18845  18860  18864  18915  18924  18947  18950  18963  18981 | ENSG00000182674 ENSG00000258819 ENSG00000270296 ENSG00000229245 ENSG00000248240 ENSG00000230647 ENSG00000229536 ENSG00000224854 ENSG00000226435 | 0.7408192508  1.369198387  0.7995844052  1.090367558  1.284597081  0.7086423341  0.7083574824  0.9622718584  0.7718342067 | 2.912260285  2.201342902  3.00204657  2.409924528  2.076039979  2.890883352  2.890456959  2.439007451  2.096946972 | 2.664334868  2.016920944  2.752513427  2.220500101  1.914658039  2.673755616  2.673796028  2.258674501  1.946490721 | 1.093053399  1.091437376  1.090656467  1.085307101  1.084287605  1.081207024  1.081031211  1.079840167  1.077296156 | 0.2743703548  0.2750804739  0.2754240729  0.2777856617  0.2782372993  0.2796050343  0.2796832302  0.2802133609  0.2813479789 | 0.412116066  0.4128540771  0.4132821144  0.415680838  0.4161357789  0.4177178499  0.4177538038  0.418273448  0.4195688263 | 0  0.9967698847  0  0  0  0  0  0  0 | 0  0.7173025769  0  0  0  0  0  0  0.7173025769 | 0  0  0  0  1.560090524  0  0  0  0 | 0  0  0  0.9271856394  0  0  0  0  0 | 0  7.751840908  0  0  0  0  0  0  0 | 0  0.9729249532  0  0  0  0  0  0  0 | 0  5.991542324  0.7489427904  0  0.7489427904  0  0  0  0 | 0  0  0  0  0  0  0  0  0.615839528 | 5.57705917  0  8.365588755  0  2.788529585  2.788529585  1.394264792  1.394264792  5.57705917 | 1.390846572  0  0  4.172539716  6.95423286  1.390846572  2.781693144  1.390846572  1.390846572 | 0  0  0  4.14083481  0  0  0  8.281669619  0 | 1.921925267  0  0.4804813169  3.843850535  3.363369218  4.324331852  4.324331852  0.4804813169  0.9609626337 |
| 19030 ENSG00000189348 0.8537962045 2.789369894 2.601105944 1.072378424 0.2835500948 0.4217640033 0 0 0 0 0 0 0 0 4.182794377 0 4.14083481 1.921925267 | | | | | | | | | | | | | | | | | | | |
| 19031  19062  19070  19074  19075  19076  19163  19180  19224 | ENSG00000259557 ENSG00000233930 ENSG00000240280 ENSG00000113303 ENSG00000100433 ENSG00000260608 ENSG00000259485 ENSG00000075891 ENSG00000269793 | 0.9498168463  0.9250887967  1.447171596  0.7331061719  0.7331061719  1.383774901  0.7123438437  1.055784274  0.8171179879 | 2.417764297  2.398695275  2.034740951  2.380278973  2.380278973  3.474199778  2.855602675  3.391831395  2.724363851 | 2.254865363  2.243405361  1.904683134  2.229070633  2.229070633  3.254197552  2.704485644  3.218873226  2.598661365 | 1.072243308  1.069220622  1.068283178  1.067834701  1.067834701  1.067605676  1.055876441  1.05373252  1.048372015 | 0.2836107628  0.2849702686  0.2853927943  0.285595082  0.285595082  0.285698422  0.291024669  0.2920053959  0.294467242 | 0.4218320768  0.4231648528  0.4236144959  0.4238036377  0.4238036377  0.4239347628  0.4298775912  0.4309439383  0.4335824882 | 0  0  0  0  0  0  0  0  0 | 0  0  1.434605154  0  0  0  0  0  0 | 0  0.780045262  0  0  0  0  0  0  0 | 0  0  0  0  0  0  0  0  0 | 0  0  0  0  0  0  0  0  0 | 0.9729249532  0  0  0  0  0  0  0  0 | 0.7489427904  0  1.497885581  0  0  0  0  0  0 | 0  0  0  0  0  0  0.615839528  0  0 | 1.394264792  1.394264792  2.788529585  1.394264792  1.394264792  4.182794377  6.971323962  0  0 | 0  5.563386288  0  2.781693144  2.781693144  0  0  8.345079432  2.781693144 | 8.281669619  0  8.281669619  4.14083481  4.14083481  12.42250443  0  0  4.14083481 | 0  3.363369218  3.363369218  0.4804813169  0.4804813169  0  0.9609626337  4.324331852  2.882887901 |
| 19264 ENSG00000128815 0.7368876543 2.887652293 2.764991204 1.044362199 0.2963178458 0.4354014194 0 0 0 0 0 0 0 0 6.971323962 1.390846572 0 0.4804813169 | | | | | | | | | | | | | | | | | | | |
| 19289  19316  19324  19358  19415  19440  19524  19574  19603 | ENSG00000282943 ENSG00000250862 ENSG00000233424 ENSG00000123500 ENSG00000197641 ENSG00000131951 ENSG00000279851 ENSG00000271627 ENSG00000184672 | 1.308147521  0.9816997541  0.9271602051  0.8816867939  0.8129015397  1.792649172  0.8316639184  0.7812943264  0.7770778782 | 2.022958564  2.673038088  2.326088778  2.296960978  2.678830722  2.00036292  2.213606726  2.660520979  2.610987043 | 1.94344868  2.57630529  2.244564707  2.225443445  2.613900362  1.957893457  2.184729457  2.641496485  2.600962635 | 1.040911748  1.037547102  1.03632066  1.032136307  1.024840411  1.021691407  1.013217778  1.007202165  1.003854115 | 0.2979165087  0.299480956  0.3000525709  0.3020082677  0.305438495  0.3069269796  0.3109561506  0.3138376329  0.3154489364 | 0.4371879854  0.4388645651  0.4395201859  0.4416079153  0.4453124924  0.4469071546  0.4508258962  0.4538326818  0.45547325 | 0  0  0  0  0  0  0.9967698847  0  0 | 0  0  0  0  0  1.434605154  0  0  0 | 0  0  0  0.780045262  0  0  0  0  0 | 1.854371279  0  0  0  0  0.9271856394  0  0  0 | 0.9689801135  0  0  0  0  0  0  0  0 | 0  0  0.9729249532  0  0  0  0  0  0 | 8.238370695  0  0  0  0  0  1.497885581  0  0 | 1.847518584  0.615839528  0  0  0  0  0  0  0 | 2.788529585  0  0  5.57705917  0  2.788529585  0  0  0 | 0  0  1.390846572  2.781693144  4.172539716  12.51761915  5.563386288  1.390846572  2.781693144 | 0  8.281669619  8.281669619  0  4.14083481  0  0  4.14083481  4.14083481 | 0  2.882887901  0.4804813169  1.441443951  1.441443951  3.843850535  1.921925267  3.843850535  2.402406584 |
| 19617 ENSG00000124089 0.9764596189 2.60295854 2.597560193 1.002078238 0.3163058076 0.4564078193 0 0 0 0 1.937960227 0 1.497885581 0 0 0 8.281669619 0 | | | | | | | | | | | | | | | | | | | |
| 19721 | ENSG00000008118 | 0.9662034549 | 2.569749056 | 2.601229715 | 0.9878977782 | 0.3232027137 | 0.4639002086 | 0 | 0 | 0 | 0 | 0 | 0 | 0 | 0 | 0 | 1.390846572 | 8.281669619 | 1.921925267 |

| 19724 | ENSG00000243116 | 0.9334414581 | 3.217173594 | 3.257941423 | 0.9874866294 | 0.3234041344 | 0.4641187097 | 0 | 0 | 0 | 0 | 0 | 0 | 0 | 0 | 9.759853547 | 0 | 0 | 1.441443951 |
| --- | --- | --- | --- | --- | --- | --- | --- | --- | --- | --- | --- | --- | --- | --- | --- | --- | --- | --- | --- |
| 19747 | ENSG00000259587 | 0.8092547949 | 2.636276496 | 2.680430251 | 0.9835273628 | 0.325347955 | 0.4663729386 | 0 | 0 | 0 | 0 | 0 | 0 | 0 | 0 | 2.788529585 | 2.781693144 | 4.14083481 | 0 |
| 19750 | ENSG00000230526 | 0.7344490979 | 2.113801522 | 2.149497952 | 0.9833931317 | 0.3254139893 | 0.466406673 | 0.9967698847 | 0 | 0 | 0 | 1.937960227 | 0 | 2.995771162 | 0 | 0 | 0 | 0 | 2.882887901 |
| 19761 | ENSG00000169469 | 0.9286553065 | 3.201811573 | 3.258186374 | 0.9826974904 | 0.3257563462 | 0.4666190545 | 0 | 0 | 0 | 0 | 0 | 0 | 0 | 0 | 6.971323962 | 4.172539716 | 0 | 0 |
| 19793 | ENSG00000105851 | 0.8998402829 | 3.184560589 | 3.258464477 | 0.9773194126 | 0.3284110454 | 0.4696374179 | 0 | 0 | 0 | 0 | 0 | 0 | 0 | 0 | 0 | 6.95423286 | 0 | 3.843850535 |
| 19830 | ENSG00000224506 | 0.7737159851 | 2.561590551 | 2.638696569 | 0.9707787476 | 0.3316584679 | 0.4734203022 | 0 | 0 | 0 | 0 | 0 | 0 | 0 | 0 | 4.182794377 | 0 | 4.14083481 | 0.9609626337 |
| 19834 | ENSG00000185615 | 0.77286143 | 2.559589763 | 2.638529138 | 0.970082053 | 0.332005594 | 0.4738202251 | 0 | 0 | 0 | 0 | 0 | 0 | 0 | 0 | 0 | 4.172539716 | 4.14083481 | 0.9609626337 |
| 19859 | ENSG00000056291 | 0.8682330696 | 3.154467503 | 3.258957391 | 0.967937633 | 0.3330755181 | 0.4747248548 | 0 | 0 | 0 | 0 | 0 | 0 | 0 | 0 | 0 | 4.172539716 | 0 | 6.246257119 |
| 19939 | ENSG00000225072 | 0.8598001732 | 3.123318712 | 3.259478196 | 0.9582266009 | 0.3379485063 | 0.4797377342 | 0 | 0 | 0 | 0 | 0 | 0 | 0 | 0 | 0 | 6.95423286 | 0 | 3.363369218 |
| 19940 | ENSG00000279694 | 0.8598001732 | 3.123318712 | 3.259478196 | 0.9582266009 | 0.3379485063 | 0.4797377342 | 0 | 0 | 0 | 0 | 0 | 0 | 0 | 0 | 0 | 6.95423286 | 0 | 3.363369218 |
| 19979 | ENSG00000238061 | 0.7376074718 | 2.48714074 | 2.606456161 | 0.9542231236 | 0.3399707121 | 0.4815782142 | 0 | 0 | 0 | 0 | 0 | 0 | 0 | 0 | 2.788529585 | 0 | 4.14083481 | 1.921925267 |
| 19981 | ENSG00000165379 | 0.7376074718 | 2.48714074 | 2.606456161 | 0.9542231236 | 0.3399707121 | 0.4815782142 | 0 | 0 | 0 | 0 | 0 | 0 | 0 | 0 | 2.788529585 | 0 | 4.14083481 | 1.921925267 |
| 19987 | ENSG00000278057 | 0.7370377684 | 2.485790049 | 2.606330519 | 0.9537508887 | 0.3402097542 | 0.481812043 | 0 | 0 | 0 | 0 | 0 | 0 | 0 | 0 | 0 | 2.781693144 | 4.14083481 | 1.921925267 |
| 20005 | ENSG00000168913 | 0.769144806 | 2.136060188 | 2.243795756 | 0.9519851273 | 0.3411045247 | 0.4826445726 | 0 | 0 | 0.780045262 | 0 | 0 | 0 | 0 | 0 | 1.394264792 | 4.172539716 | 0 | 2.882887901 |
| 20026 | ENSG00000257696 | 0.8622475482 | 2.101298856 | 2.212943556 | 0.9495492329 | 0.3423413446 | 0.4838968177 | 0 | 0 | 0 | 0 | 0.9689801135 | 0 | 0 | 0.615839528 | 0 | 0 | 8.281669619 | 0.4804813169 |
| 20033 | ENSG00000267886 | 0.8281929599 | 3.092448029 | 3.260005204 | 0.9486021755 | 0.3428229841 | 0.4843870428 | 0 | 0 | 0 | 0 | 0 | 0 | 0 | 0 | 0 | 4.172539716 | 0 | 5.765775802 |
| 20088 | ENSG00000256995 | 0.7654980612 | 2.093567543 | 2.220026458 | 0.9430372038 | 0.3456618736 | 0.4870721324 | 0 | 0 | 0.780045262 | 0 | 0 | 0 | 0 | 0 | 4.182794377 | 2.781693144 | 0 | 1.441443951 |
| 20098 | ENSG00000188755 | 0.9625567101 | 2.502980408 | 2.656588875 | 0.9421783066 | 0.3461013585 | 0.4874487538 | 0 | 0 | 0 | 0 | 0 | 0 | 0 | 0 | 2.788529585 | 0 | 8.281669619 | 0.4804813169 |
| 20132 | ENSG00000263403 | 0.7330401561 | 2.115411489 | 2.255199991 | 0.9380150307 | 0.3482366858 | 0.4896278377 | 0 | 0 | 0.780045262 | 0 | 2.906940341 | 0 | 4.493656743 | 0.615839528 | 0 | 0 | 0 | 0 |
| 20160 | ENSG00000269096 | 0.7923692984 | 3.044638029 | 3.260843229 | 0.9336965366 | 0.3504604521 | 0.4920701169 | 0 | 0 | 0 | 0 | 0 | 0 | 0 | 0 | 0 | 2.781693144 | 0 | 6.726738436 |
| 20173 | ENSG00000255177 | 0.7965495528 | 3.039879109 | 3.260928123 | 0.9322128532 | 0.3512265343 | 0.4927967866 | 0 | 0 | 0 | 0 | 5.813880681 | 0 | 3.744713952 | 0 | 0 | 0 | 0 | 0 |
| 20207 | ENSG00000226644 | 0.7881528501 | 3.027236319 | 3.261154976 | 0.9282712235 | 0.3532668972 | 0.494856871 | 0 | 0 | 0 | 0 | 0 | 0 | 0 | 0 | 0 | 4.172539716 | 0 | 5.285294486 |
| 20219 | ENSG00000266692 | 0.7333211445 | 2.093987175 | 2.258652728 | 0.9270956745 | 0.3538768618 | 0.4954171052 | 0 | 0 | 0.780045262 | 0 | 0 | 0 | 0 | 0 | 1.394264792 | 2.781693144 | 0 | 3.843850535 |
| 20220 | ENSG00000277049 | 0.8439111046 | 2.040802085 | 2.201574818 | 0.9269737592 | 0.3539401588 | 0.4954812134 | 0 | 0 | 0 | 0 | 0 | 0 | 0.7489427904 | 0.615839528 | 0 | 0 | 8.281669619 | 0.4804813169 |
| 20258 | ENSG00000259834 | 0.7839364019 | 3.009350613 | 3.261479202 | 0.9226950188 | 0.3561661624 | 0.4976621282 | 0 | 0 | 0 | 0 | 0 | 0 | 0 | 0 | 0 | 5.563386288 | 0 | 3.843850535 |
| 20309 | ENSG00000253841 | 0.7797199537 | 2.99094885 | 3.261816858 | 0.9169579348 | 0.3591646833 | 0.5005423428 | 0 | 0 | 0 | 0 | 0 | 0 | 0 | 0 | 0 | 6.95423286 | 0 | 2.402406584 |
| 20311 | ENSG00000260467 | 0.7797199537 | 2.99094885 | 3.261816858 | 0.9169579348 | 0.3591646833 | 0.5005423428 | 0 | 0 | 0 | 0 | 0 | 0 | 0 | 0 | 0 | 6.95423286 | 0 | 2.402406584 |
| 20330 | ENSG00000241499 | 0.7449557262 | 2.467676817 | 2.698938731 | 0.9143137592 | 0.3605520028 | 0.5020061481 | 0 | 0 | 0 | 0 | 0 | 0 | 0 | 0.615839528 | 4.182794377 | 0 | 4.14083481 | 0 |
| 20356 | ENSG00000274390 | 0.7772126157 | 2.974622245 | 3.262119939 | 0.9118678348 | 0.3618382956 | 0.5031536056 | 0 | 0 | 0 | 0 | 0 | 0 | 0 | 0 | 8.365588755 | 0 | 0 | 0.9609626337 |
| 20370 | ENSG00000271202 | 0.9016195346 | 2.34713058 | 2.579187703 | 0.9100270511 | 0.3628082439 | 0.5041556284 | 0 | 0 | 0 | 0 | 0 | 0 | 0 | 0.615839528 | 0 | 0 | 8.281669619 | 1.921925267 |
| 20382 | ENSG00000284202 | 0.9270402579 | 2.445547045 | 2.691423997 | 0.9086442893 | 0.3635379202 | 0.5048721602 | 0 | 0.7173025769 | 0 | 0 | 0 | 4.864624766 | 0 | 5.542555752 | 0 | 0 | 0 | 0 |
| 20437 | ENSG00000275327 | 0.7328213202 | 2.429157762 | 2.695694601 | 0.9011249868 | 0.367521869 | 0.5090313658 | 0 | 0 | 0 | 0 | 0 | 0 | 0 | 0 | 0 | 4.172539716 | 4.14083481 | 0.4804813169 |
| 20440 | ENSG00000153820 | 0.7438962922 | 2.939054813 | 3.262791766 | 0.9007791559 | 0.3677057523 | 0.5092107949 | 0 | 0 | 0 | 0 | 0 | 0 | 0 | 0 | 0 | 5.563386288 | 0 | 3.363369218 |
| 20441 | ENSG00000255372 | 0.7438962922 | 2.939054813 | 3.262791766 | 0.9007791559 | 0.3677057523 | 0.5092107949 | 0 | 0 | 0 | 0 | 0 | 0 | 0 | 0 | 0 | 5.563386288 | 0 | 3.363369218 |
| 20514 | ENSG00000260469 | 0.7122890789 | 2.906248435 | 3.26342576 | 0.890551417 | 0.3731698727 | 0.5148889309 | 0 | 0 | 0 | 0 | 0 | 0 | 0 | 0 | 0 | 2.781693144 | 0 | 5.765775802 |
| 20538 | ENSG00000231465 | 0.7354633958 | 2.898323279 | 3.263581011 | 0.8880806911 | 0.3744973483 | 0.5161418805 | 0 | 0 | 0 | 0 | 0 | 0 | 0 | 0 | 0 | 8.345079432 | 0 | 0.4804813169 |
| 20549 | ENSG00000203446 | 0.8655909941 | 2.894135123 | 3.263663387 | 0.886775007 | 0.3752000461 | 0.5168335444 | 0 | 0 | 0 | 0 | 0 | 0 | 0 | 0 | 0 | 0 | 4.14083481 | 6.246257119 |
| 20627 | ENSG00000260973 | 0.724888248 | 2.006244522 | 2.289048333 | 0.8764535431 | 0.3807835214 | 0.5225412496 | 0 | 0 | 0.780045262 | 0 | 0 | 0 | 0 | 0 | 1.394264792 | 5.563386288 | 0 | 0.9609626337 |
| 20692 | ENSG00000125820 | 1.195369141 | 2.800766958 | 3.222182883 | 0.8692141507 | 0.3847300089 | 0.5262984551 | 0 | 0 | 0 | 0 | 0 | 0 | 0 | 0 | 0 | 0 | 12.42250443 | 1.921925267 |
| 20862 | ENSG00000108244 | 1.309100701 | 2.014148432 | 2.374758158 | 0.8481488628 | 0.3963550721 | 0.5377828909 | 0.9967698847 | 0 | 0.780045262 | 0 | 0 | 0 | 0 | 0 | 9.759853547 | 4.172539716 | 0 | 0 |
| 21250 | ENSG00000283098 | 0.8867913588 | 2.169965866 | 2.70008574 | 0.8036655407 | 0.4215901604 | 0.5615779332 | 0 | 0 | 0 | 0 | 0.9689801135 | 0 | 0 | 0 | 0 | 1.390846572 | 8.281669619 | 0 |
| 21553 | ENSG00000249192 | 0.8687397669 | 2.080734039 | 2.694190065 | 0.772304102 | 0.4399343365 | 0.5777479217 | 0 | 0 | 0 | 0 | 0 | 0 | 0.7489427904 | 0 | 1.394264792 | 0 | 8.281669619 | 0 |
| 21688 | ENSG00000248206 | 0.7299592513 | 2.060600207 | 2.726983896 | 0.7556334344 | 0.4498690042 | 0.5871175265 | 0 | 0 | 0.780045262 | 0 | 0 | 0 | 0 | 0 | 5.57705917 | 0 | 0 | 2.402406584 |
| 21759 | ENSG00000173702 | 0.7563379076 | 2.085241435 | 2.792237679 | 0.7467994042 | 0.4551846553 | 0.5921437958 | 0 | 0.7173025769 | 0 | 0 | 0 | 0 | 0 | 0 | 5.57705917 | 2.781693144 | 0 | 0 |
| 21969 | ENSG00000238283 | 0.9225166004 | 2.375459809 | 3.275860878 | 0.7251406265 | 0.468365763 | 0.6035119652 | 0 | 0 | 0 | 0 | 0 | 0 | 0 | 0 | 2.788529585 | 0 | 8.281669619 | 0 |
| 22067 | ENSG00000283578 | 0.8903396837 | 2.311063549 | 3.240008279 | 0.7132893961 | 0.4756666978 | 0.6101518805 | 0 | 0 | 0 | 0 | 0 | 0 | 0 | 0 | 0 | 0 | 8.281669619 | 2.402406584 |
|  |  |  |  |  |  |  |  |  |  |  |  |  |  |  |  |  |  |  |  |
