# Supplementary material for: Analysis of H3K4me3-ChIP-Seq and RNA-Seq data to understand the putative role of miRNAs and their target genes in breast cancer cell lines
Source: Genomics Inform. 2021 Jun 30;19(2):e17. doi: 10.5808/gi.21020 (PMC8261273; doi:10.5808/gi.21020)
Supplement: Supplementary Table 13. — Differentially expressed genes in normal-like and cancer cell lines [file gi-21020suppl13.docx]

**Supplementary Table 13.** Differentially expressed genes in normal-like and cancer cell lines

| Normal-like | Luminal-A (MCF7, ZR751) | | TNBC (MB231, MB436) | |
| --- | --- | --- | --- | --- |
|  | Up-regulated | Down-regulated | Up-regulated | Down-regulated |
| MCF10A | 1,189 | 687 | 954 | 167 |
