# Supplementary material for: Analysis of H3K4me3-ChIP-Seq and RNA-Seq data to understand the putative role of miRNAs and their target genes in breast cancer cell lines
Source: Genomics Inform. 2021 Jun 30;19(2):e17. doi: 10.5808/gi.21020 (PMC8261273; doi:10.5808/gi.21020)
Supplement: Supplementary Table 14. — List of genes downregulated in normal-like vs. triple-negative breast cancer cell lines [file gi-21020suppl14.pdf]

List of genes downregulated in Normal-like vs TNBC cell-lines

Gene	baseMean	log2FoldChange	lfcSE	stat	pvalue	padj	MCF10A.RNA.Seq.Rep1_sorted	MCF10A.RNA.Seq.Rep2_sorted	MCF10A.RNA.Seq.Rep3_sorted	MCF10A.RNA.Seq.Rep4_sorted	MB231.RNA.Seq.Rep1_sorted	MB231.RNA.Seq.Rep2_sorted	MB231.RNA.Seq.Rep3_sorted	MB231.RNA.Seq.Rep4_sorted	MB436.RNA.Seq.Rep1_sorted	MB436.RNA.Seq.Rep2_sorted	MB436.RNA.Seq.Rep3_sorted	MB436.RNA.Seq.Rep4_sorted
ENSG00000118898	2244.885	-2.565	1.388	-1.848	0.065	0.136	7855.543	2131.823	7929.940	2216.901	3070.698	330.794	2944.843	400.912	18.125	18.081	4.141	16.817
ENSG00000113739	2908.398	-2.342	1.343	-1.744	0.081	0.164	5863.000	6650.112	5929.124	6583.945	30.038	4.865	34.451	5.543	1779.082	3193.384	1706.024	3121.207
ENSG00000168916	662.680	-2.639	1.613	-1.636	0.102	0.195	2182.926	913.126	2024.998	899.370	733.518	278.257	653.827	262.348	0.000	1.391	0.000	2.402
ENSG00000170558	1655.169	-2.310	1.376	-1.679	0.093	0.182	1842.031	5159.557	1925.932	5225.618	13.566	3.892	3.745	9.238	1742.831	1175.265	1594.221	1166.128
ENSG00000170561	382.938	-2.444	1.428	-1.712	0.087	0.173	1068.537	612.576	1109.224	571.146	342.050	284.094	324.292	273.433	5.577	0.000	0.000	4.324
ENSG00000050327	318.147	-2.729	1.417	-1.926	0.054	0.118	896.096	614.728	820.608	602.671	271.314	162.478	264.377	179.825	2.789	0.000	0.000	2.883
ENSG00000121858	253.412	-2.166	1.764	-1.228	0.219	0.349	834.296	254.642	761.324	254.049	398.251	76.861	405.927	54.194	1.394	0.000	0.000	0.000
ENSG00000038427	1126.580	-2.057	1.544	-1.332	0.183	0.305	818.348	3740.733	799.546	3770.864	2.907	2.919	0.749	1.232	1312.003	904.050	1180.138	985.467
ENSG00000173530	506.436	-2.152	1.584	-1.358	0.174	0.294	790.439	1289.710	799.546	1311.968	435.072	507.867	431.391	507.452	0.000	1.391	0.000	2.402
ENSG00000169594	361.537	-2.895	1.947	-1.487	0.137	0.245	629.959	1104.646	605.315	1078.317	0.000	0.000	0.000	0.000	225.871	197.500	281.577	215.256
ENSG00000176046	217.166	-2.166	1.106	-1.959	0.050	0.111	584.107	351.478	531.991	336.568	380.809	3.892	360.241	4.311	8.366	20.863	4.141	19.219
ENSG00000245067	278.419	-2.549	1.837	-1.387	0.165	0.283	584.107	719.454	528.091	659.229	168.603	261.717	172.257	247.567	0.000	0.000	0.000	0.000
ENSG00000279806	243.760	-3.019	1.805	-1.672	0.094	0.184	528.288	642.703	543.692	632.341	223.834	52.538	238.913	62.816	0.000	0.000	0.000	0.000
ENSG00000196159	211.139	-2.016	1.050	-1.921	0.055	0.119	470.475	354.347	446.966	425.578	195.734	216.962	177.499	231.556	2.789	6.954	0.000	4.805
ENSG00000145703	104.856	-2.572	1.432	-1.796	0.072	0.149	342.889	109.747	382.222	104.772	0.969	0.000	2.247	0.000	86.444	62.588	115.943	50.451
ENSG00000147027	162.557	-2.631	1.520	-1.731	0.083	0.167	297.037	397.386	301.097	478.428	0.000	0.973	0.000	0.616	136.638	127.958	95.239	115.316
ENSG00000123364	115.856	-3.049	1.728	-1.765	0.078	0.158	277.102	306.288	245.714	289.282	0.000	0.000	0.000	0.000	52.982	61.197	95.239	62.463
ENSG00000118495	162.590	-2.365	1.494	-1.583	0.114	0.212	277.102	435.403	281.596	410.743	2.907	0.000	0.000	0.616	136.638	136.303	103.521	166.247
ENSG00000128510	121.750	-2.082	1.074	-1.938	0.053	0.116	271.121	215.908	276.136	230.869	90.115	135.237	89.124	145.954	4.183	1.391	0.000	0.961
ENSG00000116132	81.310	-2.193	1.575	-1.392	0.164	0.280	178.422	149.916	176.290	173.384	0.000	0.000	0.000	0.616	50.194	77.887	78.676	90.330
ENSG00000178031	94.452	-2.017	1.688	-1.195	0.232	0.364	177.425	201.562	195.791	184.510	58.139	117.724	72.647	125.631	0.000	0.000	0.000	0.000
ENSG00000181218	43.612	-2.186	1.678	-1.303	0.193	0.318	128.583	49.494	131.828	53.777	0.000	0.000	0.000	0.000	34.857	54.243	12.423	58.138
ENSG00000247993	86.567	-2.929	1.713	-1.710	0.087	0.173	118.616	291.225	113.107	299.481	0.000	0.000	0.000	0.000	62.742	73.715	28.986	50.931
ENSG00000118402	46.524	-2.979	1.582	-1.883	0.060	0.128	115.625	101.140	120.907	107.554	0.000	0.000	0.000	0.000	15.337	45.898	12.423	39.399
ENSG00000177822	49.084	-2.157	1.142	-1.889	0.059	0.127	80.738	106.161	81.125	137.223	0.000	0.973	1.498	0.000	54.376	40.335	41.408	45.165
ENSG00000284695	19.228	-2.091	1.122	-1.864	0.062	0.132	68.777	12.911	66.304	8.345	0.000	0.000	0.749	0.616	19.520	19.472	12.423	21.622
ENSG00000227279	51.920	-2.276	1.193	-1.908	0.056	0.122	56.816	171.435	57.723	152.986	0.000	0.000	0.749	0.616	59.953	33.380	53.831	35.556
ENSG00000232821	16.210	-2.392	1.276	-1.874	0.061	0.130	55.819	13.629	52.263	15.762	0.000	0.000	0.000	0.000	18.125	4.173	28.986	5.766
ENSG00000283445	17.202	-2.107	1.076	-1.959	0.050	0.111	36.880	35.865	38.222	31.524	22.287	8.756	18.724	14.164	0.000	0.000	0.000	0.000
ENSG00000183542	8.094	-2.182	1.146	-1.904	0.057	0.123	30.900	2.152	29.642	5.563	14.535	1.946	8.987	0.616	0.000	2.782	0.000	0.000
ENSG00000261069	17.324	-2.196	1.150	-1.910	0.056	0.122	24.919	29.409	43.683	42.651	0.000	0.000	0.749	0.000	12.548	4.173	37.268	12.493
ENSG00000258590	9.079	-2.037	1.045	-1.949	0.051	0.113	22.926	14.346	21.841	13.908	0.000	0.000	0.749	0.000	13.943	5.563	4.141	11.532
ENSG00000107742	2.563	-2.137	1.095	-1.952	0.051	0.113	12.958	0.717	7.800	0.000	1.938	0.973	0.000	1.232	1.394	2.782	0.000	0.961
ENSG00000238042	2.340	-2.401	1.474	-1.628	0.103	0.198	12.958	0.717	5.460	1.854	4.845	0.000	2.247	0.000	0.000	0.000	0.000	0.000
ENSG00000274864	4.678	-2.368	1.220	-1.941	0.052	0.115	11.961	6.456	15.601	7.417	0.000	0.000	0.000	0.000	1.394	4.173	0.000	9.129
ENSG00000123405	3.213	-2.164	1.323	-1.635	0.102	0.196	11.961	2.869	11.701	0.927	3.876	0.000	6.740	0.000	0.000	0.000	0.000	0.480
ENSG00000042781	1.585	-2.538	1.313	-1.934	0.053	0.117	10.964	2.152	1.560	0.000	0.000	0.973	0.749	1.232	0.000	1.391	0.000	0.000
ENSG00000262585	2.824	-2.057	1.089	-1.888	0.059	0.127	10.964	3.587	5.460	2.782	2.907	0.000	0.749	0.000	0.000	6.954	0.000	0.480
ENSG00000250961	2.738	-2.852	1.572	-1.815	0.070	0.145	9.968	0.717	15.601	0.000	4.845	0.973	0.749	0.000	0.000	0.000	0.000	0.000
ENSG00000283848	2.110	-2.251	1.316	-1.711	0.087	0.173	8.971	5.021	4.680	0.000	1.938	0.000	2.247	2.463	0.000	0.000	0.000	0.000
ENSG00000284601	1.451	-2.156	1.289	-1.672	0.094	0.184	8.971	1.435	2.340	0.000	0.969	0.973	1.498	1.232	0.000	0.000	0.000	0.000
ENSG00000228013	1.496	-2.623	1.446	-1.814	0.070	0.145	7.974	0.717	5.460	0.000	0.000	0.973	0.000	0.000	0.000	1.391	0.000	1.441
ENSG00000279807	1.514	-2.103	1.238	-1.699	0.089	0.176	7.974	0.717	3.900	0.927	0.000	0.000	1.498	1.232	0.000	0.000	0.000	1.922
ENSG00000228216	0.919	-3.805	2.236	-1.702	0.089	0.176	6.977	0.000	3.120	0.927	0.000	0.000	0.000	0.000	0.000	0.000	0.000	0.000
ENSG00000120471	0.901	-3.779	2.239	-1.688	0.091	0.179	6.977	0.717	3.120	0.000	0.000	0.000	0.000	0.000	0.000	0.000	0.000	0.000
ENSG00000115705	0.841	-2.177	1.841	-1.182	0.237	0.370	6.977	0.000	0.780	0.000	0.969	0.000	0.749	0.616	0.000	0.000	0.000	0.000
ENSG00000272459	1.782	-2.153	1.137	-1.894	0.058	0.126	5.981	1.435	6.240	0.927	0.000	0.000	0.749	0.000	2.789	2.782	0.000	0.480
ENSG00000258360	2.355	-2.063	1.092	-1.889	0.059	0.127	5.981	3.587	5.460	5.563	0.000	0.000	2.247	0.616	0.000	0.000	0.000	4.805
ENSG00000175899	2.625	-2.102	1.148	-1.831	0.067	0.141	5.981	2.869	5.460	5.563	0.000	0.000	0.000	0.000	0.000	5.563	4.141	1.922
ENSG00000182050	1.501	-2.623	1.480	-1.772	0.076	0.156	5.981	0.000	7.020	0.927	1.938	0.000	0.749	0.000	1.394	0.000	0.000	0.000
ENSG00000227925	1.872	-2.179	2.077	-1.049	0.294	0.433	5.981	0.000	10.141	0.000	4.845	0.000	1.498	0.000	0.000	0.000	0.000	0.000
ENSG00000260647	1.396	-2.303	1.206	-1.910	0.056	0.122	4.984	3.587	3.900	0.000	1.938	0.973	0.749	0.616	0.000	0.000	0.000	0.000
ENSG00000212237	1.418	-2.352	1.233	-1.908	0.056	0.122	4.984	0.717	3.120	1.854	0.000	0.973	0.000	1.232	0.000	0.000	4.141	0.000
ENSG00000259005	1.198	-2.147	1.145	-1.876	0.061	0.130	4.984	0.717	3.900	0.927	0.000	0.000	0.749	1.232	0.000	1.391	0.000	0.480
ENSG00000258303	1.167	-2.435	1.316	-1.851	0.064	0.136	4.984	2.152	2.340	1.854	0.000	0.000	0.749	0.000	0.000	0.000	0.000	1.922
ENSG00000225269	2.683	-2.287	1.275	-1.794	0.073	0.150	4.984	3.587	8.580	6.490	0.000	4.865	0.000	3.695	0.000	0.000	0.000	0.000
ENSG00000231738	1.042	-2.478	1.456	-1.702	0.089	0.176	4.984	0.000	3.900	0.927	0.969	0.973	0.749	0.000	0.000	0.000	0.000	0.000
ENSG00000259932	1.014	-2.145	1.261	-1.702	0.089	0.176	4.984	2.152	0.780	0.927	0.969	0.000	0.000	0.000	0.000	1.391	0.000	0.961
ENSG00000268834	0.851	-3.117	1.865	-1.671	0.095	0.185	4.984	0.717	3.120	0.000	0.000	0.000	0.000	0.000	0.000	1.391	0.000	0.000
ENSG00000206262	1.073	-2.679	1.648	-1.626	0.104	0.198	4.984	0.000	2.340	0.927	0.000	0.000	0.000	0.000	0.000	0.000	4.141	0.480
ENSG00000221972	0.818	-3.639	2.262	-1.609	0.108	0.203	4.984	0.000	3.900	0.927	0.000	0.000	0.000	0.000	0.000	0.000	0.000	0.000
ENSG00000185385	0.791	-2.646	1.653	-1.601	0.109	0.206	4.984	0.717	2.340	0.000	0.000	0.973	0.000	0.000	0.000	0.000	0.000	0.480
ENSG00000127249	0.750	-3.015	1.921	-1.570	0.116	0.216	4.984	0.000	2.340	0.927	0.000	0.000	0.749	0.000	0.000	0.000	0.000	0.000
ENSG00000230710	0.735	-3.486	2.303	-1.514	0.130	0.236	4.984	0.717	3.120	0.000	0.000	0.000	0.000	0.000	0.000	0.000	0.000	0.000
ENSG00000272281	0.959	-2.185	1.466	-1.491	0.136	0.244	4.984	0.717	3.120	0.000	0.969	0.000	0.000	1.232	0.000	0.000	0.000	0.480
ENSG00000185527	2.072	-2.099	1.078	-1.948	0.051	0.114	3.987	4.304	6.240	2.782	0.000	0.973	0.000	0.000	0.000	4.173	0.000	2.402
ENSG00000155749	1.949	-2.061	1.063	-1.940	0.052	0.116	3.987	4.304	3.900	3.709	0.000	0.000	0.000	0.000	1.394	4.173	0.000	1.922
ENSG00000214725	1.137	-2.210	1.141	-1.936	0.053	0.116	3.987	2.152	1.560	2.782	0.969	0.000	0.749	0.000	0.000	0.000	0.000	1.441
ENSG00000133454	0.845	-2.669	1.394	-1.914	0.056	0.121	3.987	0.717	0.780	2.782	0.000	0.000	0.000	0.000	0.000	1.391	0.000	0.480
ENSG00000274987	0.983	-2.549	1.449	-1.759	0.079	0.159	3.987	0.000	4.680	0.927	0.969	0.000	0.749	0.000	0.000	0.000	0.000	0.480
ENSG00000254715	0.768	-2.496	1.422	-1.756	0.079	0.160	3.987	0.717	0.780	1.854	0.000	0.000	0.000	0.000	0.000	1.391	0.000	0.480
ENSG00000246331	0.863	-3.719	2.131	-1.745	0.081	0.163	3.987	3.587	0.000	2.782	0.000	0.000	0.000	0.000	0.000	0.000	0.000	0.000
ENSG00000232645	1.458	-2.362	1.362	-1.735	0.083	0.166	3.987	0.717	2.340	6.490	0.000	0.000	1.498	2.463	0.000	0.000	0.000	0.000
ENSG00000281560	0.930	-3.828	2.221	-1.723	0.085	0.170	3.987	0.000	6.240	0.927	0.000	0.000	0.000	0.000	0.000	0.000	0.000	0.000
ENSG00000269552	0.833	-3.084	1.864	-1.655	0.098	0.190	3.987	0.717	3.900	0.000	0.000	0.000	0.000	0.000	0.000	1.391	0.000	0.000
ENSG00000269815	1.090	-2.607	1.594	-1.635	0.102	0.196	3.987	1.435	5.460	0.000	0.000	0.000	0.000	1.232	0.000	0.000	0.000	0.961
ENSG00000228330	0.744	-3.003	1.851	-1.623	0.105	0.199	3.987	0.000	2.340	1.854	0.000	0.000	0.749	0.000	0.000	0.000	0.000	0.000
ENSG00000278039	1.794	-2.206	1.381	-1.598	0.110	0.207	3.987	0.717	4.680	4.636	0.000	0.000	0.000	0.000	0.000	0.000	4.141	3.363
ENSG00000258957	1.288	-2.151	1.368	-1.573	0.116	0.215	3.987	5.021	1.560	0.927	0.000	0.000	2.996	0.000	0.000	0.000	0.000	0.961
ENSG00000211644	0.773	-2.352	1.626	-1.446	0.148	0.260	3.987	2.152	1.560	0.000	0.000	0.000	0.000	0.616	0.000	0.000	0.000	0.961
ENSG00000183833	1.518	-2.048	1.449	-1.414	0.157	0.272	3.987	2.869	5.460	0.000	0.000	0.973	0.749	0.000	4.183	0.000	0.000	0.000
ENSG00000223387	0.835	-2.104	1.692	-1.243	0.214	0.343	3.987	0.000	0.780	2.782	0.969	0.000	1.498	0.000	0.000	0.000	0.000	0.000
ENSG00000272516	0.865	-2.036	1.693	-1.203	0.229	0.361	3.987	0.000	0.780	2.782	0.000	0.000	0.000	0.000	0.000	1.391	0.000	1.441
ENSG00000275343	0.742	-2.218	2.549	-0.870	0.384	0.526	3.987	0.000	0.780	0.000	0.000	0.000	0.000	0.000	0.000	0.000	4.141	0.000
ENSG00000053108	0.797	-2.585	1.326	-1.949	0.051	0.113	2.990	1.435	2.340	0.927	0.000	0.000	0.000	0.000	0.000	1.391	0.000	0.480
ENSG00000236658	1.666	-2.448	1.256	-1.949	0.051	0.114	2.990	1.435	6.240	1.854	1.938	0.000	0.000	0.000	0.000	1.391	4.141	0.000
ENSG00000266158	0.848	-2.898	1.497	-1.936	0.053	0.116	2.990	3.587	0.780	1.854	0.000	0.000	0.000	0.000	0.000	0.000	0.000	0.961
ENSG00000182183	0.762	-2.933	1.534	-1.912	0.056	0.121	2.990	0.717	3.120	0.927	0.000	0.000	0.000	0.000	0.000	1.391	0.000	0.000
ENSG00000279718	1.386	-2.274	1.196	-1.900	0.057	0.124	2.990	5.021	3.900	0.000	0.000	0.973	0.000	0.000	1.394	1.391	0.000	0.961
ENSG00000224337	1.776	-2.073	1.097	-1.890	0.059	0.126	2.990	1.435	7.020	2.782	1.938	0.973	0.000	0.000	1.394	2.782	0.000	0.000

List of genes downregulated in Normal-like vs TNBC cell-lines

Gene	baseMean	log2FoldChange	lfcSE	stat	pvalue	padj	MCF10A.RNA.Seq.Rep1_sorted	MCF10A.RNA.Seq.Rep2_sorted	MCF10A.RNA.Seq.Rep3_sorted	MCF10A.RNA.Seq.Rep4_sorted	MB231.RNA.Seq.Rep1_sorted	MB231.RNA.Seq.Rep2_sorted	MB231.RNA.Seq.Rep3_sorted	MB231.RNA.Seq.Rep4_sorted	MB436.RNA.Seq.Rep1_sorted	MB436.RNA.Seq.Rep2_sorted	MB436.RNA.Seq.Rep3_sorted	MB436.RNA.Seq.Rep4_sorted
ENSG00000276528	0.919	-2.467	1.322	-1.866	0.062	0.132	2.990	0.717	3.120	1.854	0.000	0.000	0.000	0.000	0.000	1.391	0.000	0.961
ENSG00000248925	0.881	-2.778	1.507	-1.843	0.065	0.138	2.990	2.869	3.120	0.000	0.000	0.973	0.000	0.616	0.000	0.000	0.000	0.000
ENSG00000232352	0.819	-2.225	1.223	-1.818	0.069	0.144	2.990	2.152	1.560	0.927	0.000	0.973	0.749	0.000	0.000	0.000	0.000	0.480
ENSG00000187904	0.848	-2.703	1.498	-1.804	0.071	0.147	2.990	0.717	3.120	1.854	0.000	0.000	1.498	0.000	0.000	0.000	0.000	0.000
ENSG00000106540	0.965	-2.121	1.202	-1.765	0.077	0.157	2.990	1.435	3.120	0.927	0.000	0.000	0.000	1.232	0.000	1.391	0.000	0.480
ENSG00000246548	0.936	-2.351	1.341	-1.753	0.080	0.161	2.990	0.717	3.120	1.854	1.938	0.000	0.000	0.616	0.000	0.000	0.000	0.000
ENSG00000267675	0.729	-3.043	1.807	-1.684	0.092	0.181	2.990	2.152	3.120	0.000	0.000	0.000	0.000	0.000	0.000	0.000	0.000	0.480
ENSG00000167434	0.894	-3.774	2.247	-1.680	0.093	0.182	2.990	0.717	7.020	0.000	0.000	0.000	0.000	0.000	0.000	0.000	0.000	0.000
ENSG00000283712	1.435	-2.200	1.344	-1.637	0.102	0.195	2.990	3.587	4.680	1.854	0.000	0.000	0.749	0.000	0.000	0.000	0.000	3.363
ENSG00000229498	0.753	-2.110	1.290	-1.635	0.102	0.196	2.990	0.717	2.340	0.927	0.969	0.000	0.000	0.616	0.000	0.000	0.000	0.480
ENSG00000169340	0.794	-3.599	2.211	-1.628	0.104	0.198	2.990	0.000	4.680	1.854	0.000	0.000	0.000	0.000	0.000	0.000	0.000	0.000
ENSG00000217702	0.901	-2.348	1.452	-1.617	0.106	0.201	2.990	0.717	4.680	0.000	0.969	0.973	0.000	0.000	0.000	0.000	0.000	0.480
ENSG00000280913	0.920	-2.563	1.593	-1.609	0.108	0.203	2.990	1.435	4.680	0.000	0.000	0.973	0.000	0.000	0.000	0.000	0.000	0.961
ENSG00000280117	1.262	-2.051	1.384	-1.482	0.138	0.247	2.990	3.587	1.560	1.854	0.000	0.973	0.000	0.000	4.183	0.000	0.000	0.000
ENSG00000066405	1.095	-2.229	1.589	-1.403	0.161	0.276	2.990	2.152	4.680	0.000	0.000	0.000	0.000	0.000	0.000	1.391	0.000	1.922
ENSG00000175093	0.840	-2.575	1.937	-1.330	0.184	0.306	2.990	2.869	0.000	2.782	0.000	0.000	0.000	0.000	0.000	0.000	0.000	1.441
ENSG00000234997	0.865	-2.080	1.603	-1.297	0.195	0.320	2.990	2.152	0.000	2.782	0.000	0.000	1.498	0.000	0.000	0.000	0.000	0.961
ENSG00000198520	0.819	-2.541	2.027	-1.254	0.210	0.338	2.990	0.717	4.680	0.000	0.000	0.000	0.000	0.000	0.000	0.000	0.000	1.441
ENSG00000212172	0.769	-3.556	3.034	-1.172	0.241	0.375	2.990	0.000	6.240	0.000	0.000	0.000	0.000	0.000	0.000	0.000	0.000	0.000
ENSG00000271795	0.771	-2.133	2.097	-1.018	0.309	0.449	2.990	0.000	1.560	2.782	0.000	0.000	0.000	0.000	0.000	0.000	0.000	1.922
ENSG00000253361	1.605	-2.205	1.131	-1.950	0.051	0.113	1.994	2.869	6.240	2.782	2.907	0.973	1.498	0.000	0.000	0.000	0.000	0.000
ENSG00000221852	0.779	-2.555	1.338	-1.909	0.056	0.122	1.994	1.435	3.120	0.927	0.000	0.000	0.000	0.000	0.000	1.391	0.000	0.480
ENSG00000102678	1.008	-2.555	1.340	-1.906	0.057	0.123	1.994	1.435	3.120	0.927	0.000	0.000	0.000	0.000	0.000	0.000	4.141	0.480
ENSG00000279375	0.733	-2.535	1.344	-1.887	0.059	0.127	1.994	2.869	1.560	0.927	0.969	0.000	0.000	0.000	0.000	0.000	0.000	0.480
ENSG00000171564	1.303	-2.476	1.336	-1.854	0.064	0.135	1.994	5.021	0.780	4.636	0.000	0.000	2.247	0.000	0.000	0.000	0.000	0.961
ENSG00000185837	0.965	-2.259	1.232	-1.833	0.067	0.140	1.994	1.435	4.680	0.927	0.969	0.000	0.000	0.616	0.000	0.000	0.000	0.961
ENSG00000279175	1.064	-2.574	1.429	-1.801	0.072	0.148	1.994	1.435	0.000	6.490	0.969	0.000	0.000	0.000	1.394	0.000	0.000	0.480
ENSG00000173641	0.966	-2.316	1.323	-1.751	0.080	0.162	1.994	1.435	3.900	1.854	0.969	0.000	0.000	0.000	0.000	0.000	0.000	1.441
ENSG00000137875	0.895	-2.340	1.415	-1.653	0.098	0.190	1.994	0.717	4.680	0.927	1.938	0.000	0.000	0.000	0.000	0.000	0.000	0.480
ENSG00000175229	0.854	-2.134	1.309	-1.630	0.103	0.197	1.994	2.152	1.560	1.854	1.938	0.000	0.749	0.000	0.000	0.000	0.000	0.000
ENSG00000266379	0.805	-2.042	1.253	-1.629	0.103	0.197	1.994	0.717	2.340	1.854	0.000	0.000	0.749	0.616	1.394	0.000	0.000	0.000
ENSG00000167614	1.017	-2.183	1.407	-1.552	0.121	0.222	1.994	1.435	5.460	0.000	0.969	0.000	0.000	0.000	0.000	1.391	0.000	0.961
ENSG00000269161	0.723	-2.052	1.380	-1.487	0.137	0.245	1.994	1.435	2.340	0.927	0.000	0.000	1.498	0.000	0.000	0.000	0.000	0.480
ENSG00000284959	0.725	-2.419	1.670	-1.448	0.148	0.259	1.994	0.000	3.900	0.927	0.000	0.000	0.000	0.000	1.394	0.000	0.000	0.480
ENSG00000070193	1.096	-2.314	1.604	-1.443	0.149	0.261	1.994	2.152	3.900	0.927	0.000	0.000	0.000	0.000	4.183	0.000	0.000	0.000
ENSG00000284152	0.840	-2.088	1.492	-1.399	0.162	0.278	1.994	0.717	4.680	0.000	0.969	0.973	0.749	0.000	0.000	0.000	0.000	0.000
ENSG00000154269	0.866	-2.323	1.707	-1.361	0.173	0.293	1.994	0.717	3.900	1.854	0.000	0.000	0.000	0.000	0.000	0.000	0.000	1.922
ENSG00000188517	0.985	-2.041	1.716	-1.189	0.234	0.367	1.994	5.738	0.780	0.000	0.000	0.000	0.000	0.000	0.000	1.391	0.000	1.922
ENSG00000259616	0.753	-2.315	2.030	-1.140	0.254	0.389	1.994	0.000	4.680	0.000	0.969	0.000	0.000	0.000	1.394	0.000	0.000	0.000
ENSG00000259424	0.913	-2.448	1.256	-1.950	0.051	0.113	0.997	2.152	4.680	0.927	0.000	0.973	0.749	0.000	0.000	0.000	0.000	0.480
ENSG00000257642	1.024	-3.535	1.858	-1.902	0.057	0.124	0.997	7.890	0.000	2.782	0.000	0.000	0.000	0.616	0.000	0.000	0.000	0.000
ENSG00000264346	1.294	-2.002	1.063	-1.883	0.060	0.128	0.997	1.435	3.900	4.636	0.000	0.973	0.000	1.232	1.394	0.000	0.000	0.961
ENSG00000226758	1.133	-2.621	1.397	-1.877	0.061	0.129	0.997	2.869	2.340	1.854	0.000	0.000	0.000	0.000	1.394	0.000	4.141	0.000
ENSG00000284488	0.989	-2.115	1.149	-1.840	0.066	0.138	0.997	1.435	1.560	4.636	0.000	0.000	0.749	0.616	1.394	0.000	0.000	0.480
ENSG00000271401	0.769	-2.540	1.384	-1.834	0.067	0.140	0.997	2.152	0.780	3.709	0.000	0.973	0.000	0.616	0.000	0.000	0.000	0.000
ENSG00000227740	0.849	-2.448	1.347	-1.818	0.069	0.144	0.997	1.435	2.340	3.709	0.000	0.000	0.749	0.000	0.000	0.000	0.000	0.961
ENSG00000205325	1.161	-2.665	1.468	-1.816	0.069	0.144	0.997	0.717	3.900	2.782	0.000	0.000	0.000	0.000	1.394	0.000	4.141	0.000
ENSG00000277785	3.077	-2.411	1.333	-1.809	0.070	0.146	0.997	7.173	6.240	7.417	0.000	0.000	0.749	0.000	0.000	0.000	12.423	1.922
ENSG00000260616	3.039	-2.169	1.236	-1.755	0.079	0.161	0.997	8.608	6.240	4.636	0.000	0.973	0.749	1.848	0.000	0.000	12.423	0.000
ENSG00000234698	0.779	-2.325	1.346	-1.728	0.084	0.168	0.997	2.152	1.560	1.854	0.000	0.000	0.000	0.000	1.394	1.391	0.000	0.000
ENSG00000268287	0.910	-2.303	1.387	-1.660	0.097	0.188	0.997	2.869	0.780	3.709	0.000	1.946	0.000	0.616	0.000	0.000	0.000	0.000
ENSG00000166670	1.735	-2.346	1.463	-1.604	0.109	0.205	0.997	4.304	0.000	10.199	0.000	0.000	0.000	0.616	2.789	0.000	0.000	1.922
ENSG00000242531	1.089	-2.078	1.334	-1.558	0.119	0.220	0.997	2.869	2.340	3.709	0.000	0.000	0.000	1.232	0.000	0.000	0.000	1.922
ENSG00000265282	0.721	-2.922	1.910	-1.530	0.126	0.230	0.997	0.000	3.900	2.782	0.000	0.973	0.000	0.000	0.000	0.000	0.000	0.000
ENSG00000267084	0.733	-3.491	2.301	-1.517	0.129	0.235	0.997	5.021	0.000	2.782	0.000	0.000	0.000	0.000	0.000	0.000	0.000	0.000
ENSG00000181240	0.953	-2.666	1.769	-1.507	0.132	0.238	0.997	0.000	7.800	0.927	0.000	0.000	0.000	1.232	0.000	0.000	0.000	0.480
ENSG00000164972	0.956	-2.024	1.348	-1.502	0.133	0.240	0.997	0.717	5.460	0.927	0.000	0.000	1.498	0.000	0.000	1.391	0.000	0.480
ENSG00000237484	0.712	-2.830	1.920	-1.474	0.140	0.250	0.997	4.304	0.000	1.854	0.000	0.000	0.000	0.000	0.000	1.391	0.000	0.000
ENSG00000229585	0.772	-2.338	1.626	-1.437	0.151	0.263	0.997	0.717	1.560	1.854	0.000	0.000	0.000	0.000	0.000	0.000	4.141	0.000
ENSG00000273664	0.767	-2.323	1.624	-1.430	0.153	0.266	0.997	1.435	0.780	1.854	0.000	0.000	0.000	0.000	0.000	0.000	4.141	0.000
ENSG00000200714	0.741	-2.086	1.470	-1.419	0.156	0.270	0.997	0.717	3.900	0.927	0.000	0.000	0.000	0.000	0.000	1.391	0.000	0.961
ENSG00000145423	0.888	-2.676	1.993	-1.343	0.179	0.301	0.997	3.587	0.000	4.636	0.000	0.000	0.000	0.000	0.000	0.000	0.000	1.441
ENSG00000282849	1.729	-4.726	2.603	-1.815	0.069	0.145	0.000	11.477	0.000	9.272	0.000	0.000	0.000	0.000	0.000	0.000	0.000	0.000
ENSG00000163017	2.568	-2.568	1.516	-1.694	0.090	0.178	0.000	10.760	0.000	12.981	0.000	0.973	2.247	2.463	1.394	0.000	0.000	0.000
ENSG00000164385	1.858	-2.180	1.300	-1.677	0.094	0.183	0.000	3.587	7.020	5.563	0.000	2.919	2.247	0.000	0.000	0.000	0.000	0.961
ENSG00000254620	0.933	-2.287	1.370	-1.670	0.095	0.185	0.000	3.587	1.560	2.782	0.000	0.000	0.000	0.000	1.394	1.391	0.000	0.480
ENSG00000044524	0.840	-2.625	1.606	-1.635	0.102	0.196	0.000	3.587	0.780	3.709	0.000	0.000	0.000	0.616	0.000	1.391	0.000	0.000
ENSG00000279769	1.340	-2.398	1.472	-1.629	0.103	0.197	0.000	5.738	0.000	6.490	0.000	0.000	0.749	1.232	0.000	1.391	0.000	0.480
ENSG00000219491	0.825	-2.173	1.363	-1.594	0.111	0.208	0.000	2.152	2.340	2.782	0.000	0.000	0.749	0.000	1.394	0.000	0.000	0.480
ENSG00000129009	1.422	-2.261	1.492	-1.516	0.130	0.235	0.000	4.304	0.000	8.345	0.969	0.000	0.000	0.616	0.000	1.391	0.000	1.441
ENSG00000222915	0.938	-2.025	1.367	-1.481	0.139	0.247	0.000	3.587	1.560	2.782	0.000	0.973	0.000	0.000	0.000	1.391	0.000	0.961
ENSG00000267476	0.750	-2.322	1.635	-1.420	0.155	0.269	0.000	2.152	0.780	3.709	0.969	0.000	0.000	0.000	1.394	0.000	0.000	0.000
ENSG00000230623	0.874	-3.286	2.346	-1.400	0.161	0.277	0.000	4.304	0.000	5.563	0.000	0.000	0.000	0.616	0.000	0.000	0.000	0.000
ENSG00000271333	0.995	-2.278	1.691	-1.347	0.178	0.299	0.000	1.435	3.120	1.854	0.000	0.000	0.000	0.000	1.394	0.000	4.141	0.000
ENSG00000254510	1.103	-2.189	1.704	-1.285	0.199	0.325	0.000	2.869	0.000	6.490	0.000	0.000	0.000	0.616	0.000	2.782	0.000	0.480
ENSG00000215506	0.972	-2.190	1.820	-1.203	0.229	0.360	0.000	0.717	0.780	4.636	0.000	0.000	0.000	0.000	0.000	1.391	4.141	0.000
ENSG00000275759	1.075	-2.054	2.046	-1.004	0.315	0.455	0.000	0.000	7.020	1.854	0.000	0.000	0.000	1.232	2.789	0.000	0.000	0.000
