# Supplementary material for: Analysis of H3K4me3-ChIP-Seq and RNA-Seq data to understand the putative role of miRNAs and their target genes in breast cancer cell lines
Source: Genomics Inform. 2021 Jun 30;19(2):e17. doi: 10.5808/gi.21020 (PMC8261273; doi:10.5808/gi.21020)
Supplement: Supplementary Table 15. — List of five miRNA sequences (exclusively present in triple-negative breast cancer subtype) used for target-gene identification [file gi-21020suppl15.docx]

**Supplementary Table 15.** List of five miRNA sequences (exclusively present in triple-negative breast cancer subtype) used for target-gene identification

| miRNA | miRNA sequence |
| --- | --- |
| hsa-miR-153-1 | UUGCAUAGUCACAAAAGUGAUC |
| hsa-miR-4767 | CGCGGGCGCUCCUGGCCGCCGCC |
| hsa-miR-4487 | AGAGCUGGCUGAAGGGCAG |
| hsa-miR-6720-5p | UUCCAGCCCUGGUAGGCGCCGCG |
| hsa-miR-Let7i-5p | UGAGGUAGUAGUUUGUGCUGUU |
