# Supplementary material for: Analysis of H3K4me3-ChIP-Seq and RNA-Seq data to understand the putative role of miRNAs and their target genes in breast cancer cell lines
Source: Genomics Inform. 2021 Jun 30;19(2):e17. doi: 10.5808/gi.21020 (PMC8261273; doi:10.5808/gi.21020)
Supplement: Supplementary Table 16. — List of eight miRNA sequences present in at least three breast cell lines used for target-gene identification [file gi-21020suppl16.docx]

**Supplementary Table 16.** List of eight miRNA sequences present in at least three breast cancer cell lines used for target-gene identification

| miRNA | miRNA sequence |
| --- | --- |
| hsa-miR-4512 | CAGGGCCUCACUGUAUCGCCCA |
| hsa-miR-6791-5p | CCCCUGGGGCUGGGCAGGCGGA |
| hsa-miR-330-5p | UCUCUGGGCCUGUGUCUUAGGC |
| hsa-miR-3180-5p | CUUCCAGACGCUCCGCCCCACGUCG |
| hsa-miR-6080 | UCUAGUGCGGGCGUUCCCG |
| hsa-miR-5787 | GGGCUGGGGCGCGGGGAGGU |
| hsa-miR-6733 | UGGGAAAGACAAACUCAGAGUU |
| hsa-miR-3613 | UGUUGUACUUUUUUUUUUGUUC |
