# Supplementary material for: Analysis of H3K4me3-ChIP-Seq and RNA-Seq data to understand the putative role of miRNAs and their target genes in breast cancer cell lines
Source: Genomics Inform. 2021 Jun 30;19(2):e17. doi: 10.5808/gi.21020 (PMC8261273; doi:10.5808/gi.21020)
Supplement: Supplementary Table 17. — RNA hybrid analysis of miRNAs (present in at least three breast cancer cell lines) target genes (17 luminal-A and 15 triple-negative breast cancer) previously reported to have role in breast cancer [file gi-21020suppl17.docx]

**Supplementary Table 17.** RNA hybrid analysis of miRNAs (present in at least three breast cancer cell lines) target genes (17 luminal-A and 15 triple-negative breast cancer) previously reported to have role in breast cancer

| **miRNA** | **Putative target gene** | **RNAhybrid result** | **Binding energy (kcal/mol)** |
| --- | --- | --- | --- |
| miR5787 | RERG | **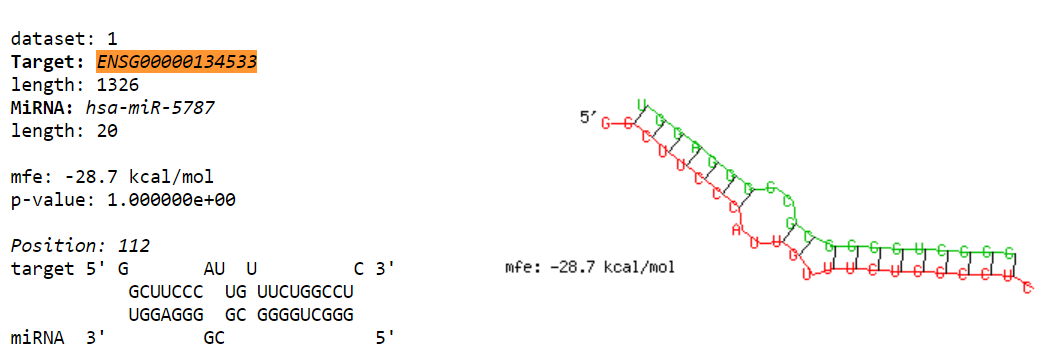** | -28.7 |
| miR5787 | IGFBP6 | **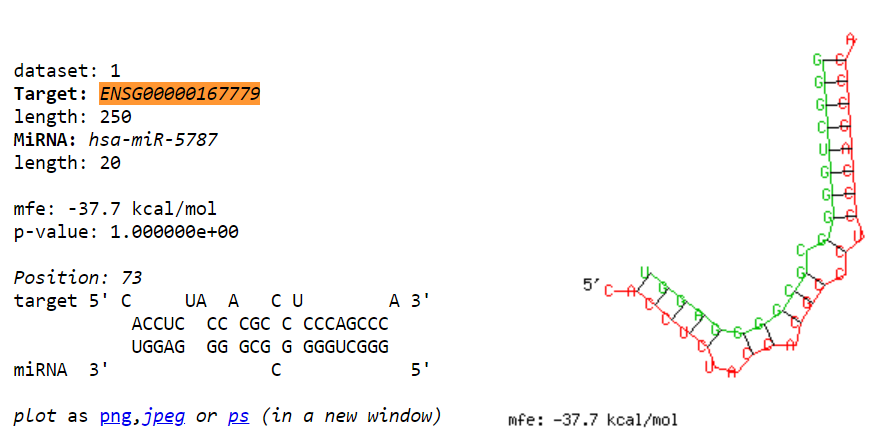** | -37.7 |
| miR6791 | BMF | **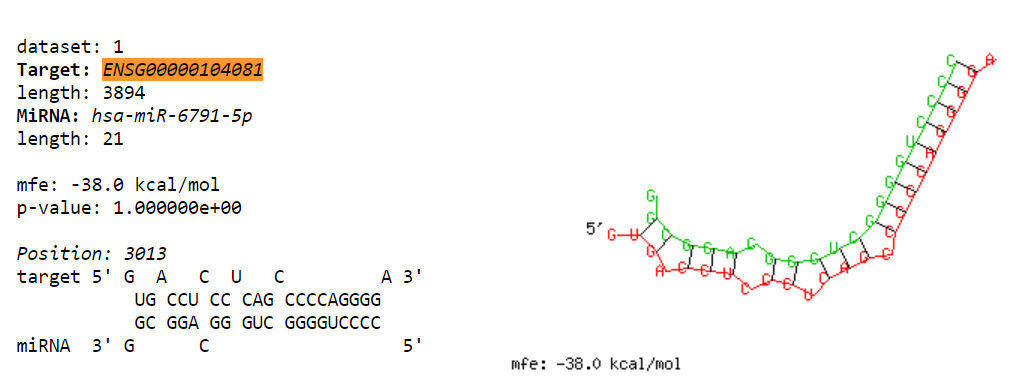** | -38.0 |
| miR5787 | BMF | **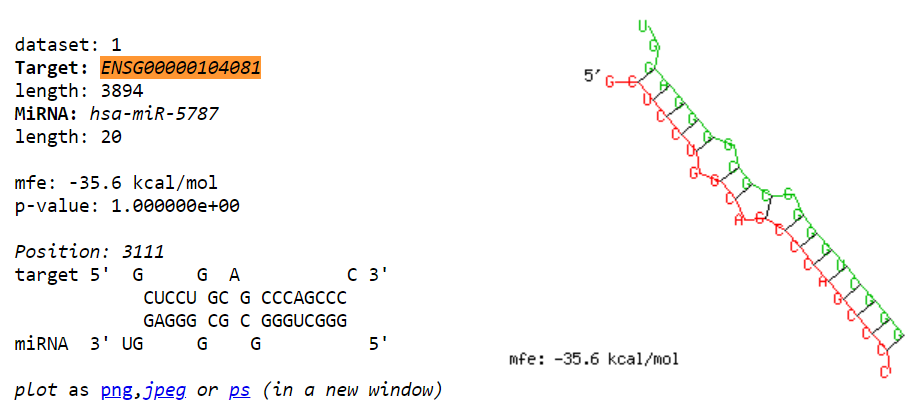** | -35.6 |
| miR4512 | BMF | **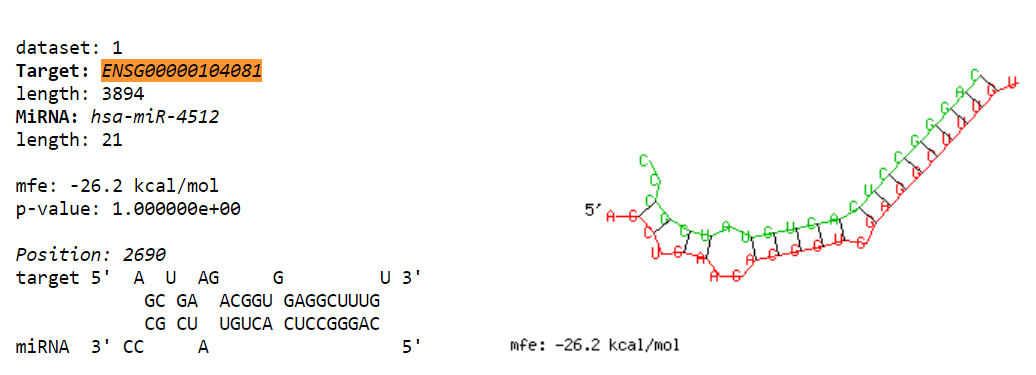** | -26.2 |
| miR3613 | IQGAP2 | **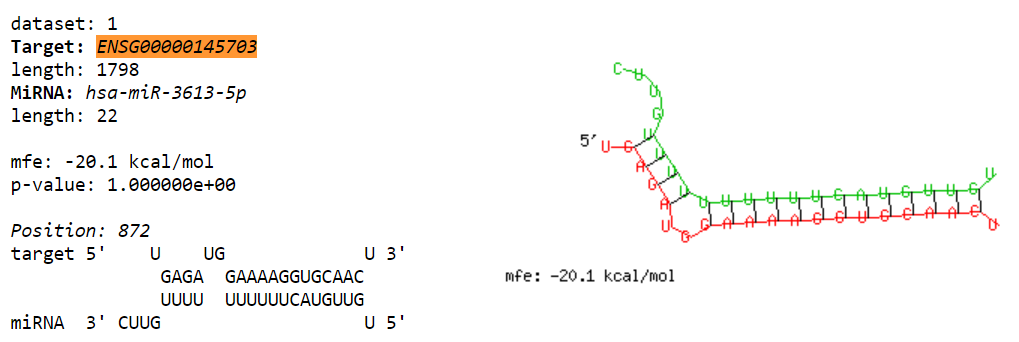** | -20.1 |
| miR5787 | TNFSF10 | **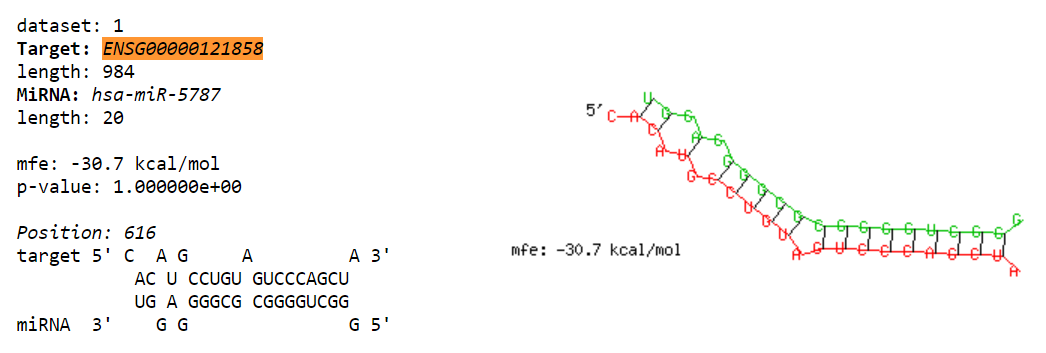** | -30.7 |
| miR3180 | FAT4 | **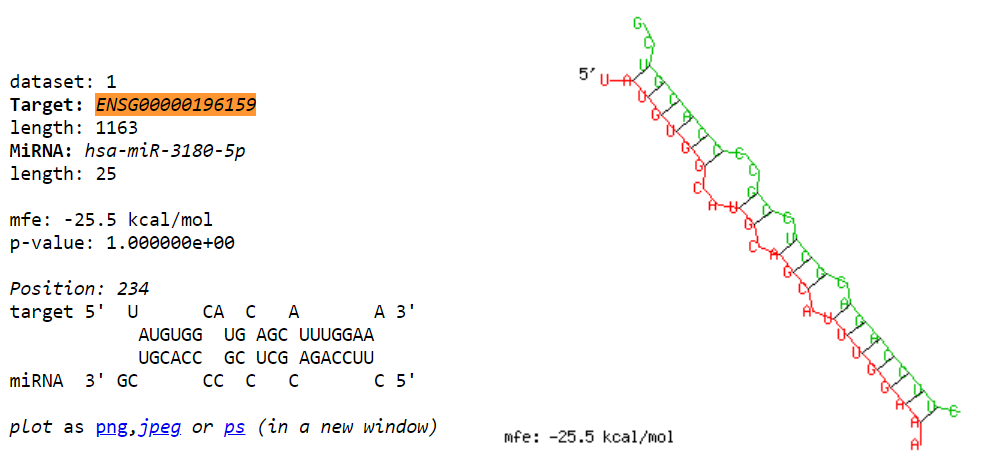** | -25.5 |
| miR330 | STC2 | **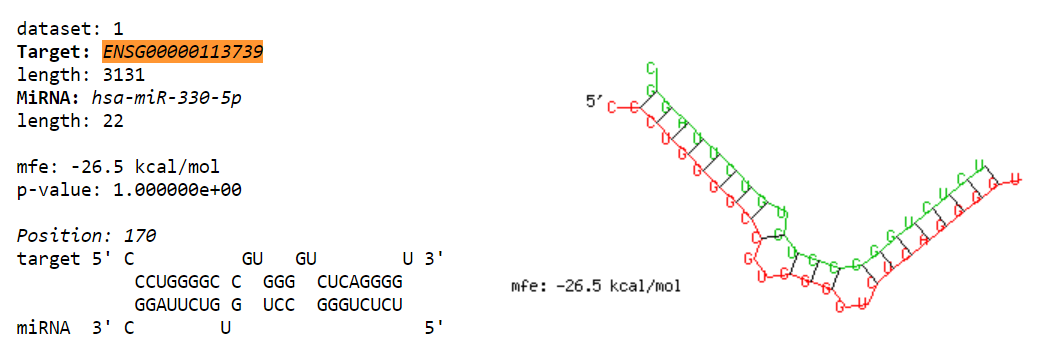** | -26.5 |
| miR6791 | STC2 | **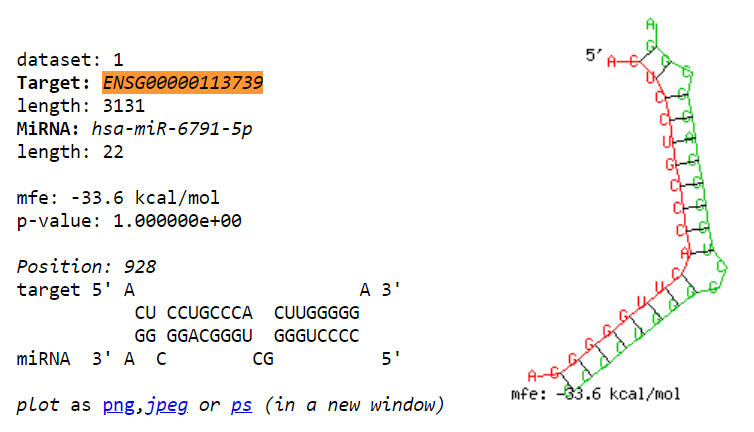** | -33.6 |
| miR5787 | STC2 | **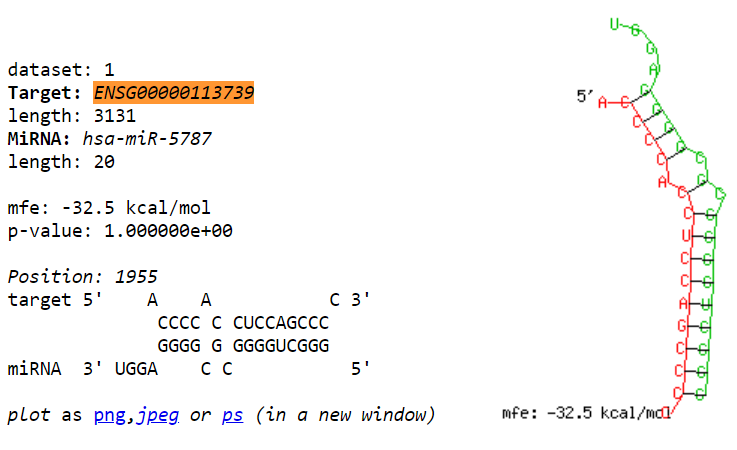** | -32.5 |
| miR5787 | ZNF655 | **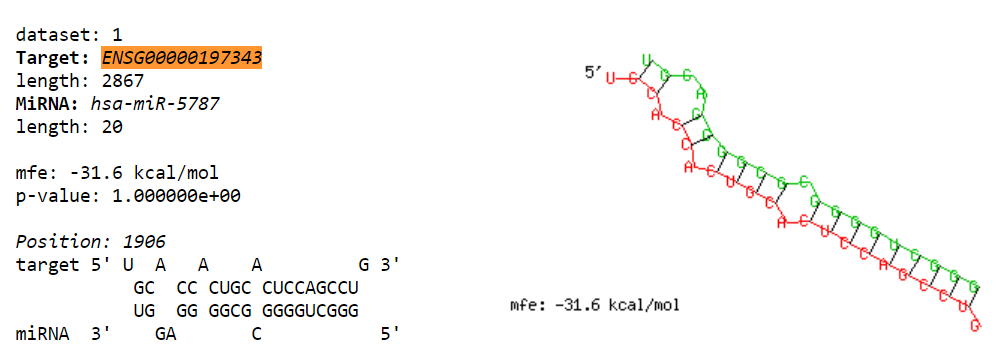** | -31.6 |
| miR5787 | AXL | **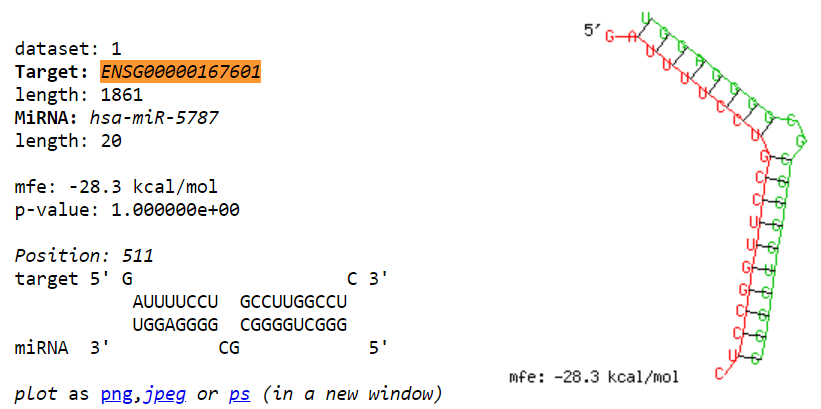** | -28.3 |
| miR6791 | FXYD5 | **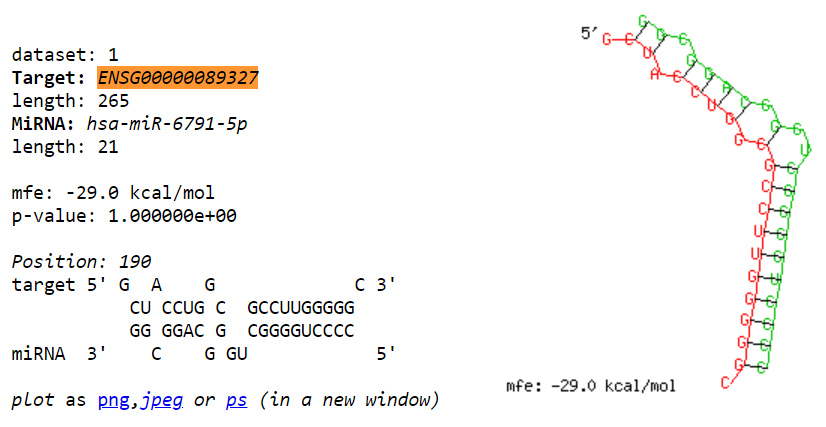** | -29.0 |
| miR330 | FXYD5 | **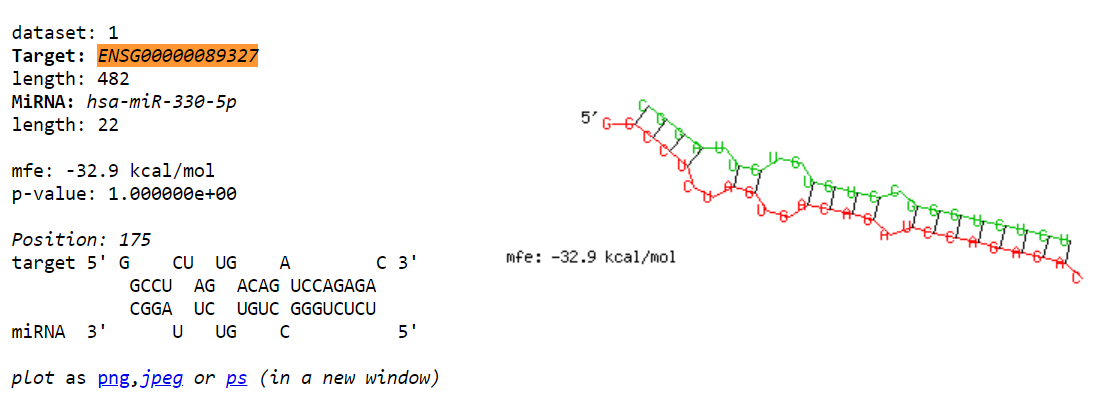** | -32.9 |
| miR5787 | FXYD5 | **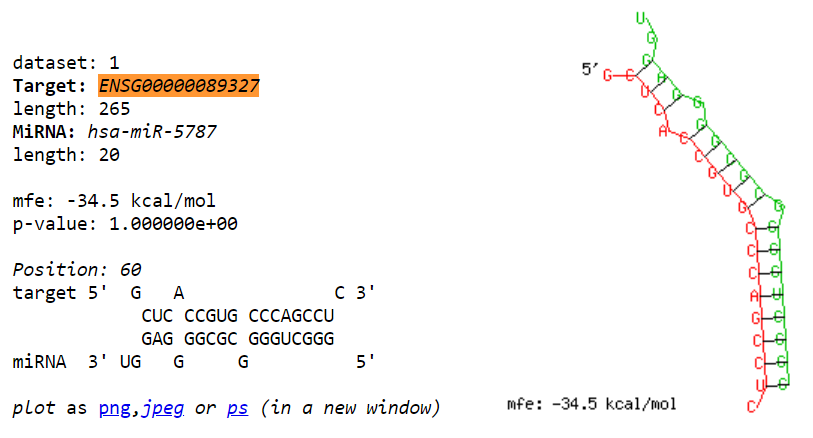** | -34.5 |
| miR6791 | PTRF | **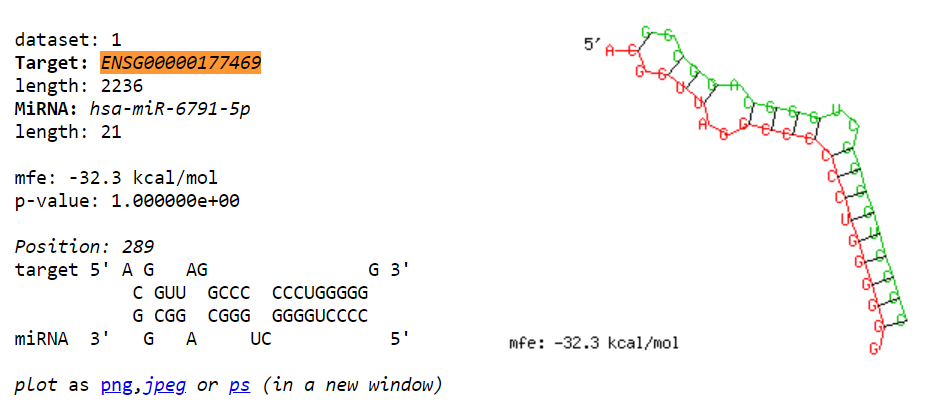** | -32.3 |
| miR330 | PTRF | **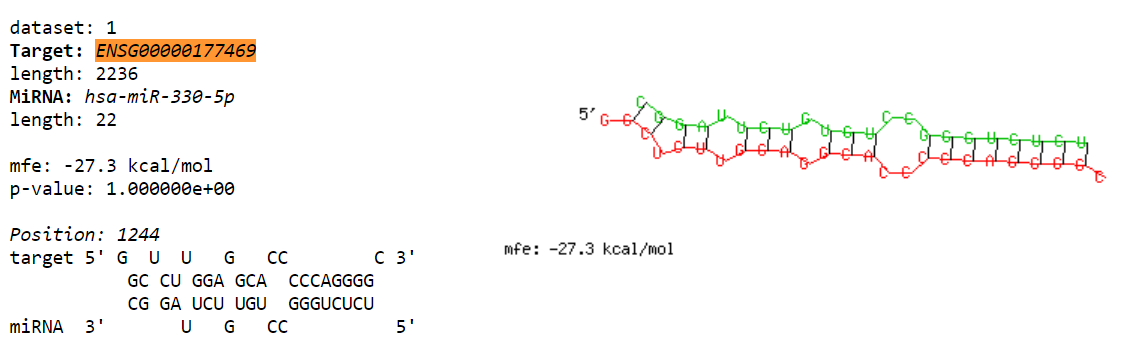** | -27.3 |
| miR5787 | PTRF | **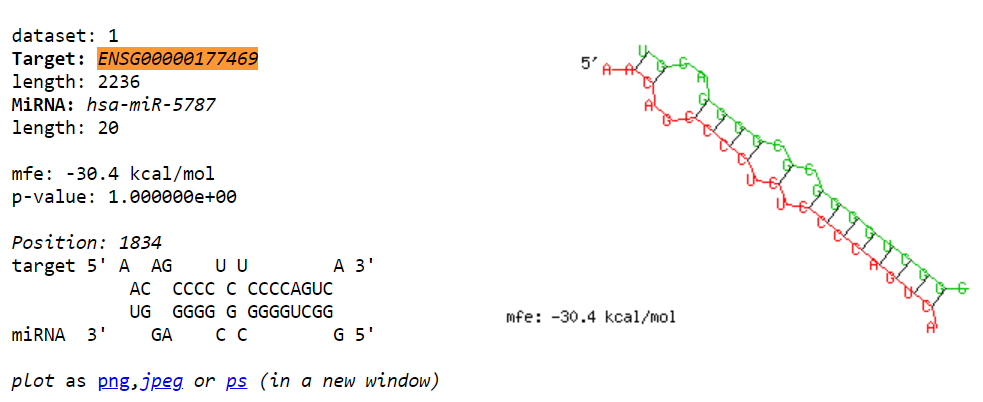** | -30.4 |
| miR5787 | RUNX2 | **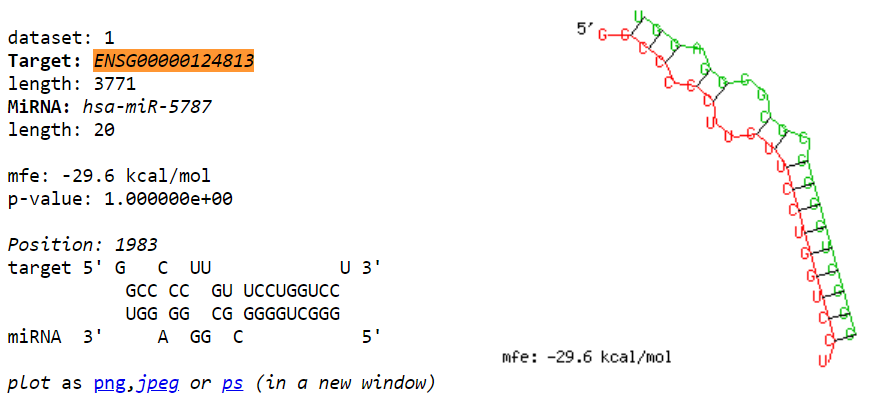** | -29.6 |
| miR6791 | RUNX2 | **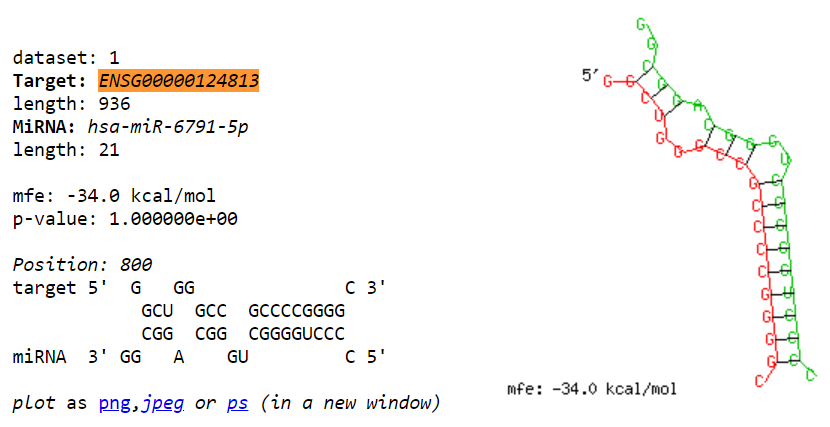** | -34.0 |
| miR5787 | SPATA18 | **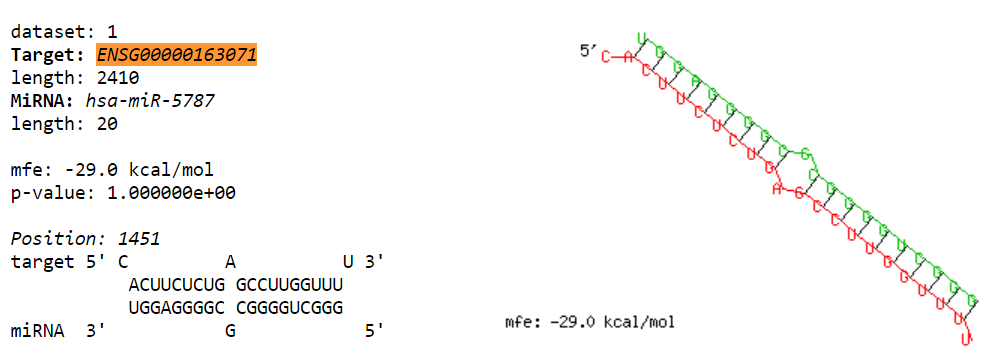** | -29.0 |
| miR5787 | UGT8 | **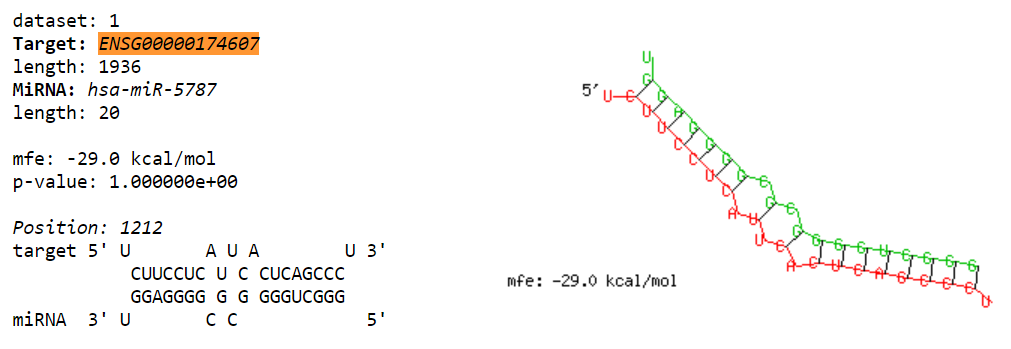** | -29 |
| miR6791 | UGT8 | **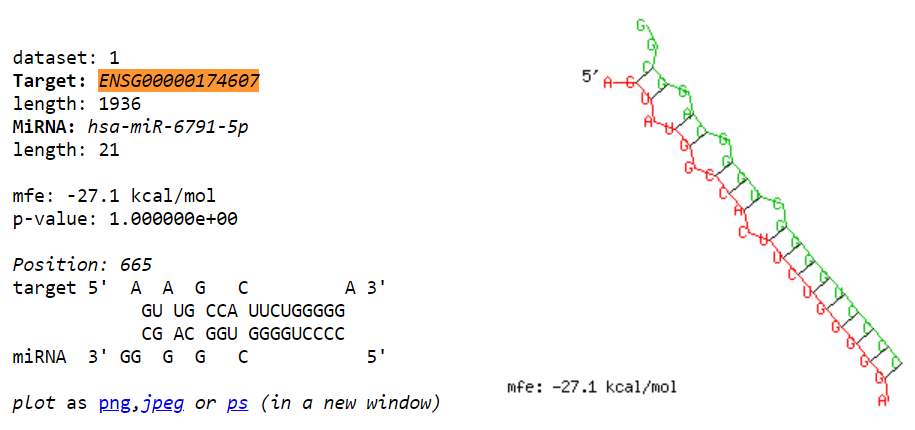** | -27.1 |
| miR3613 | UGT8 | **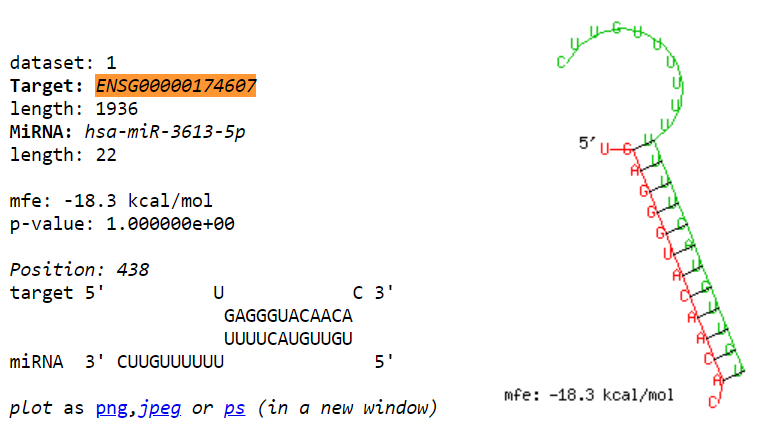** | -18.3 |
| miR5787 | TMEM47 | **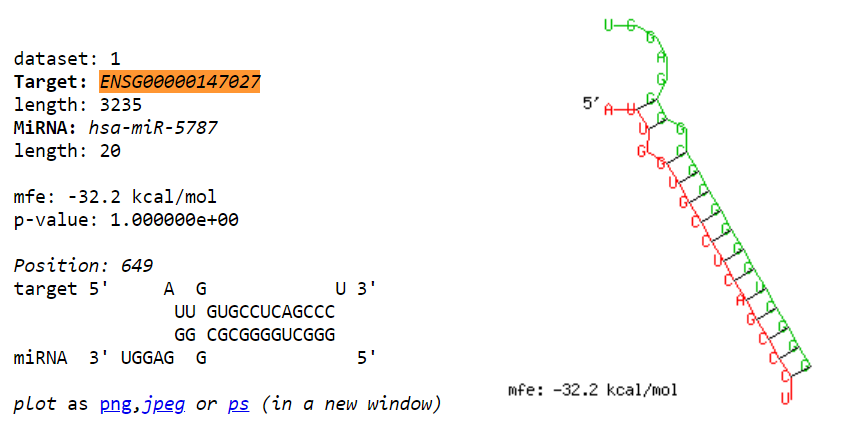** | -32.2 |
| miR5787 | NUPR1 | **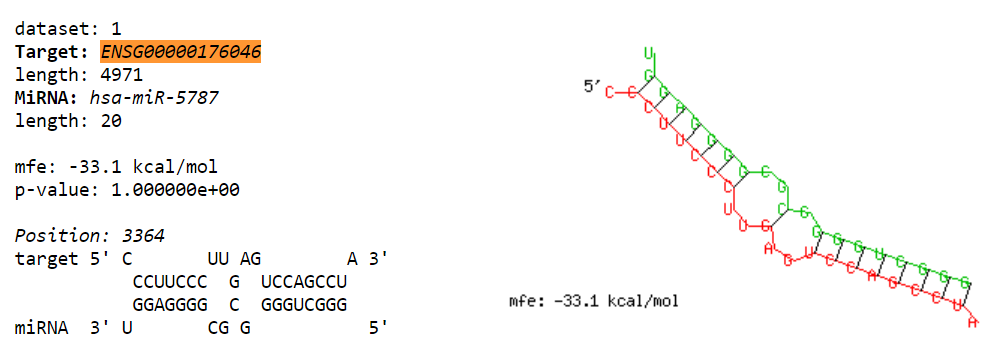** | -33.1 |
| miR3180-3 | NUPR1 | **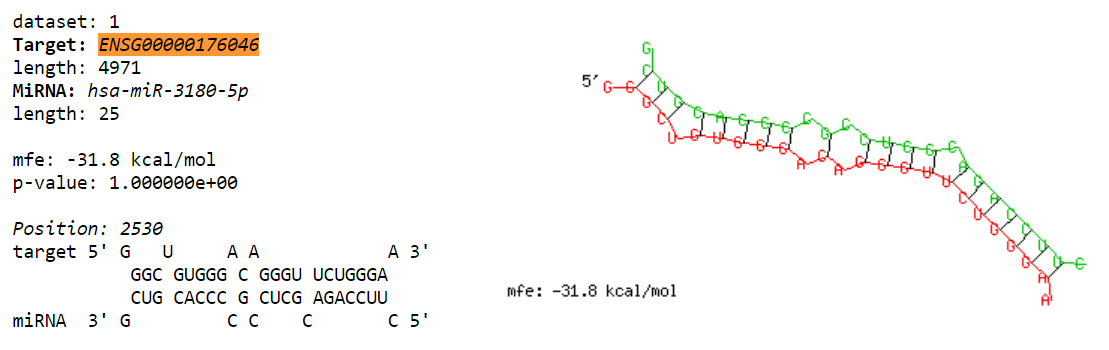** | -31.8 |
| miR330 | NUPR1 | **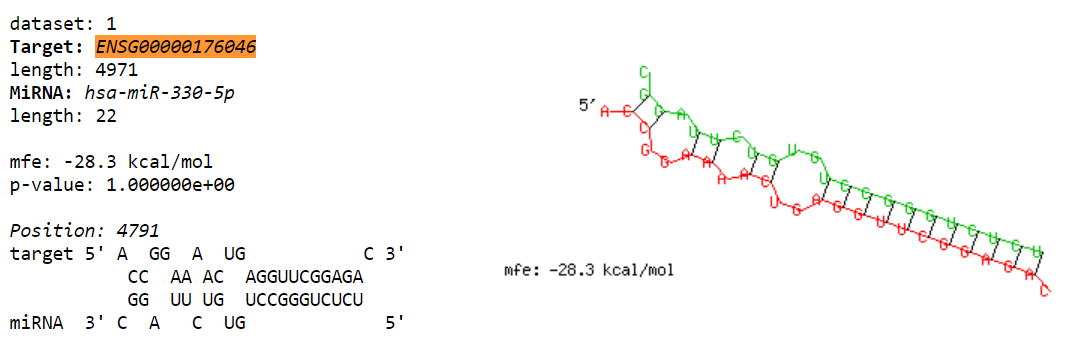** | -28.3 |
| miR6791 | NUPR1 | **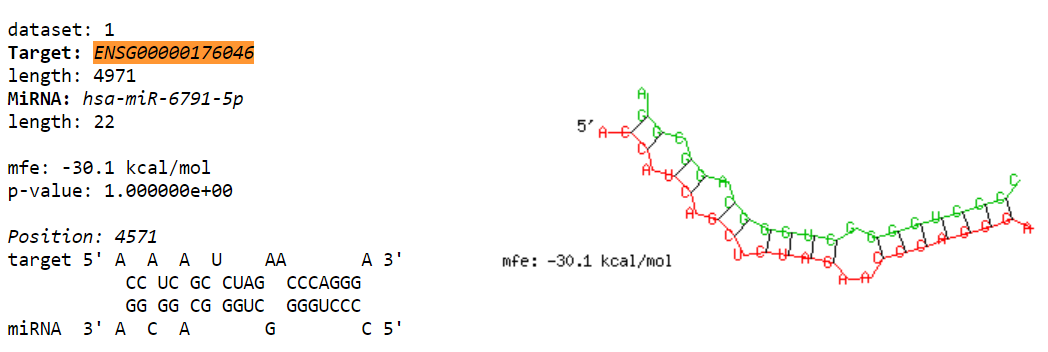** | -30.1 |
| miR5787 | HOXC13 | **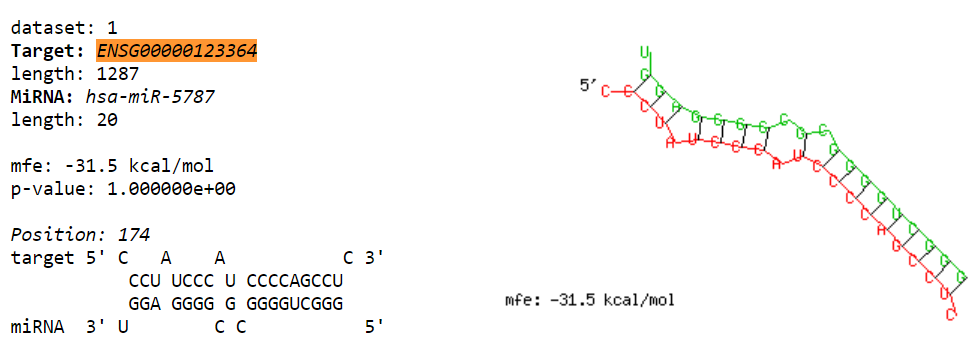** | -31.5 |
| miR330 | HOXC13 | **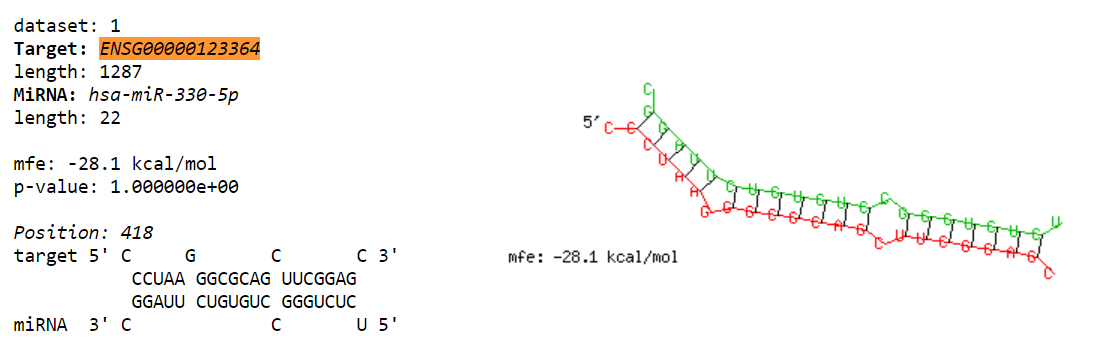** | -28.1 |
| miR4512 | PRRX1 | **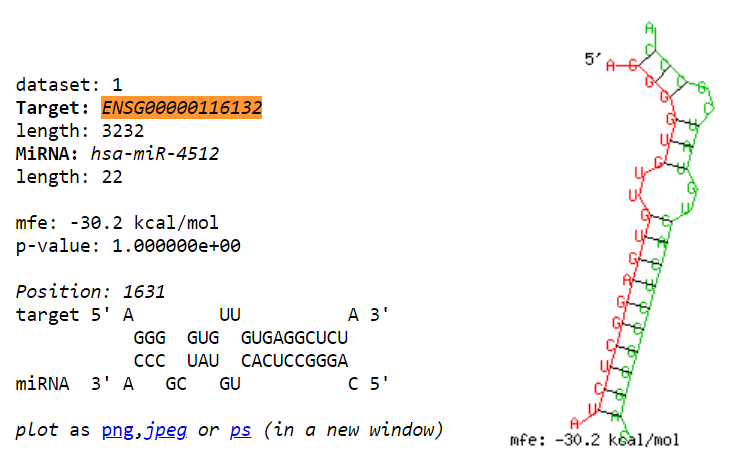** | -30.2 |
| miR5787 | PRRX1 | **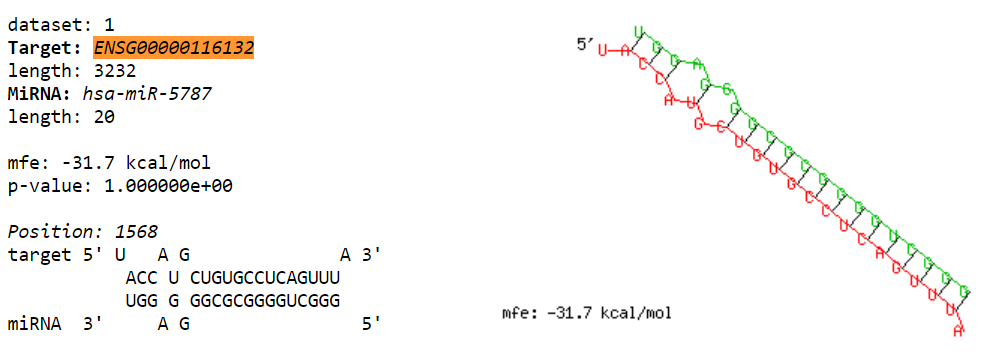** | -31.7 |
| miR6791 | PRRX1 | **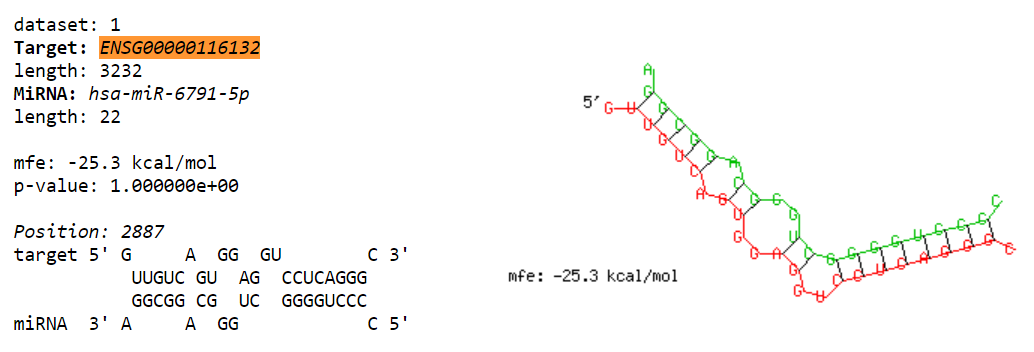** | -25.3 |
| miR3613 | PRRX1 | **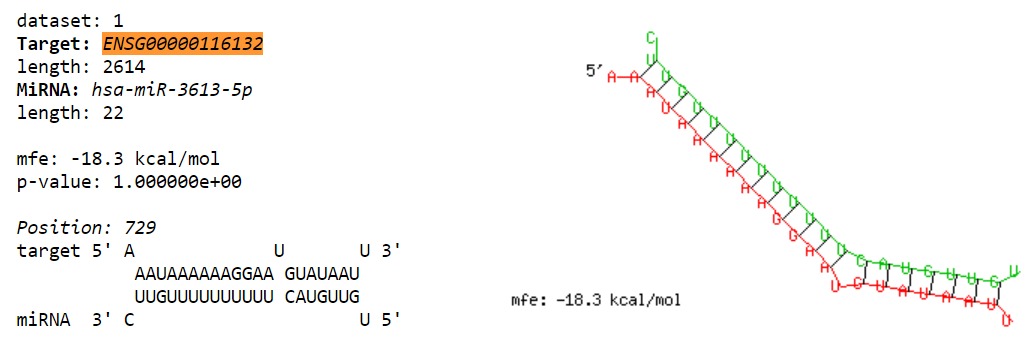** | -18.3 |
| miR330 | CFB | **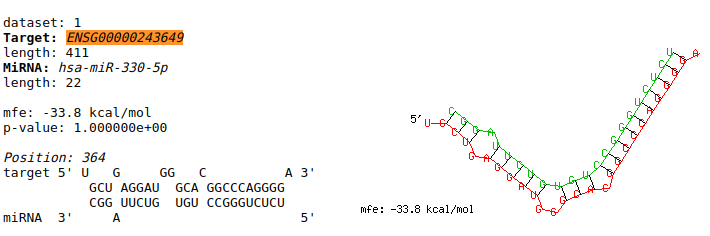** | -33.8 |
| miR5787 | CFB | **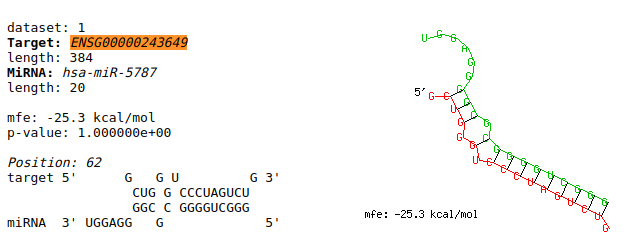** | -25.3 |
| miR6791 | CFB | **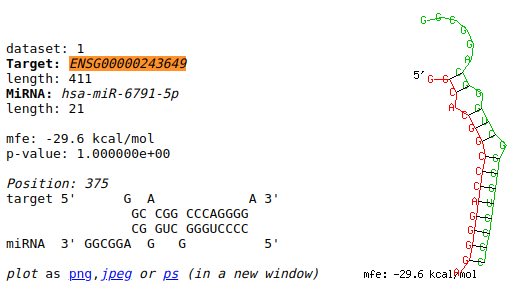** | -29.6 |
| miR330 | CSF3 | **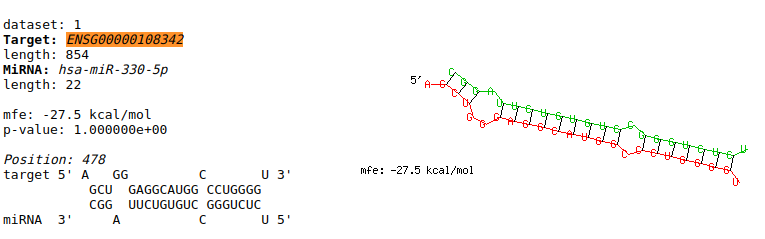** | -27.5 |
| miR4512 | CSF3 | **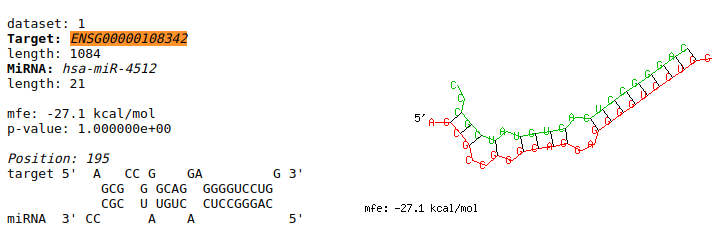** | -27.1 |
| miR6791 | CSF3 | **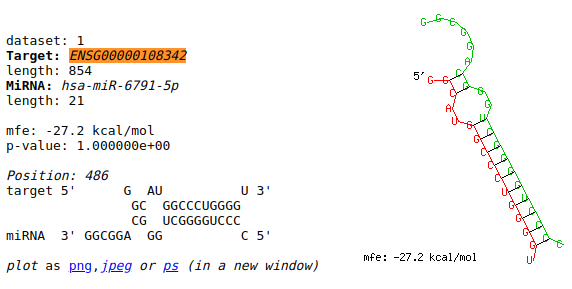** | -27.2 |
| miR330 | HEG1 | **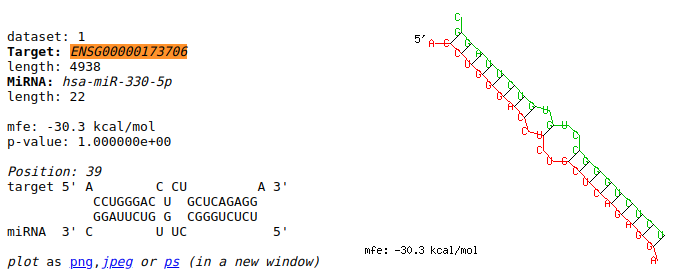** | -30.3 |
| miR3180-3 | HEG1 | **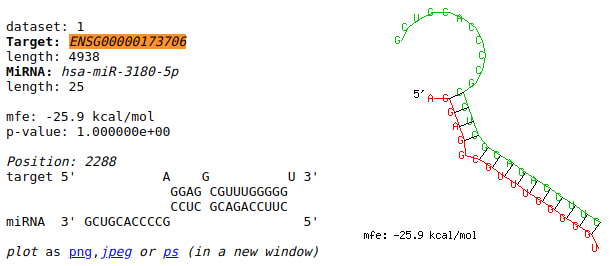** | -25.9 |
| miR5787 | HEG1 | **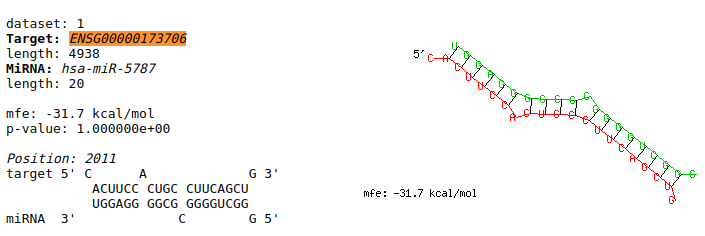** | -31.7 |
| miR6791 | HEG1 | **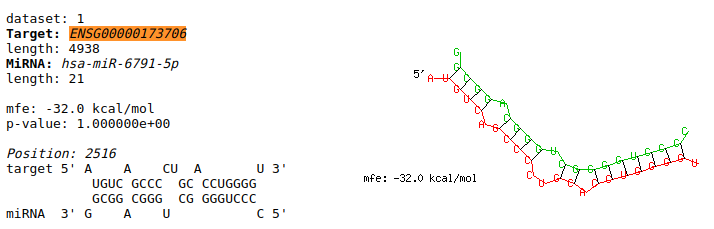** | -32.0 |
| miR3180-3 | PLAU | **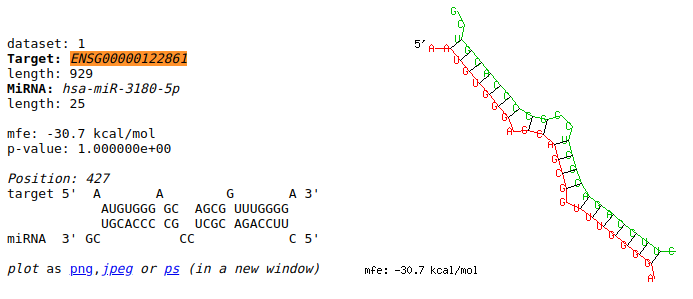** | -30.7 |
| miR5787 | PTER | **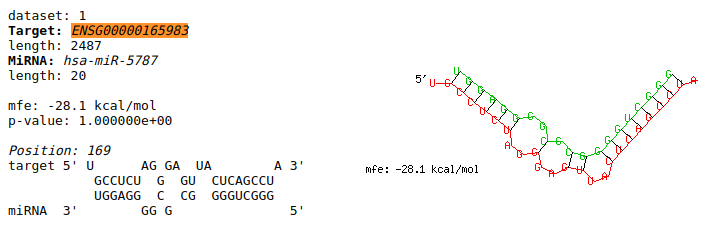** | -28.1 |
| miR330 | S100A3 | **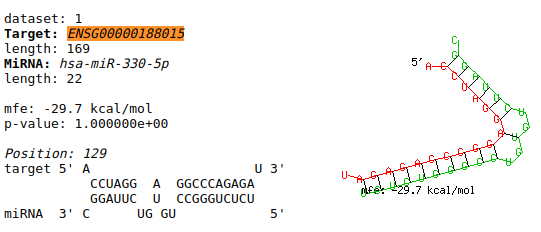** | -29.7 |
| miR3180-3 | S100A3 | **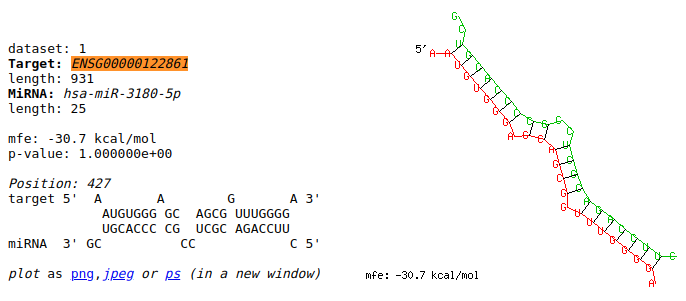** | -30.7 |
| miR4512 | S100A3 | **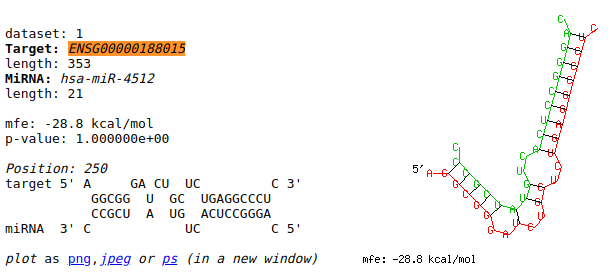** | -28.8 |
| miR5787 | S100A3 | **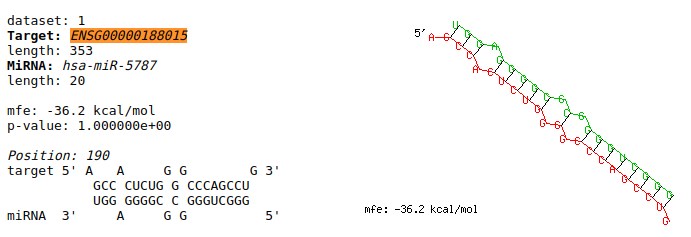** | -36.2 |
| miR6791 | S100A3 | **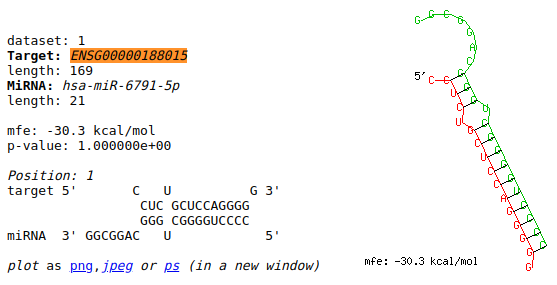** | -30.3 |
| miR4512 | SNURF | **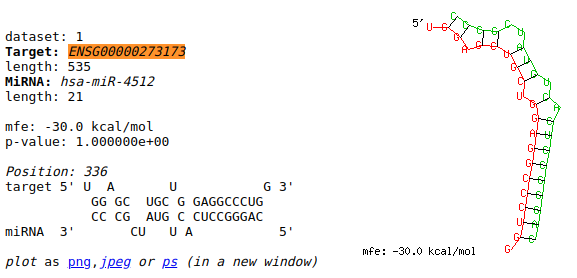** | -30.0 |
| miR6791 | SNURF | **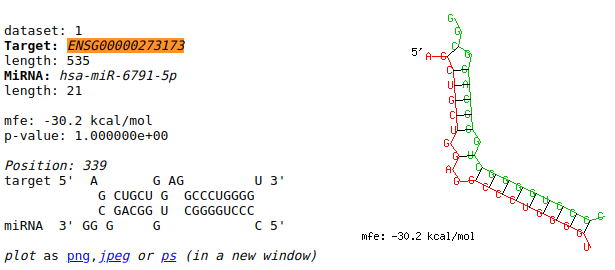** | -30.2 |
| miR3180-3 | WIPF1 | **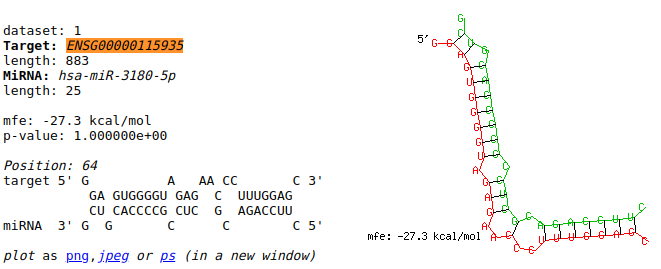** | -27.3 |
| miR3613 | WIPF1 | **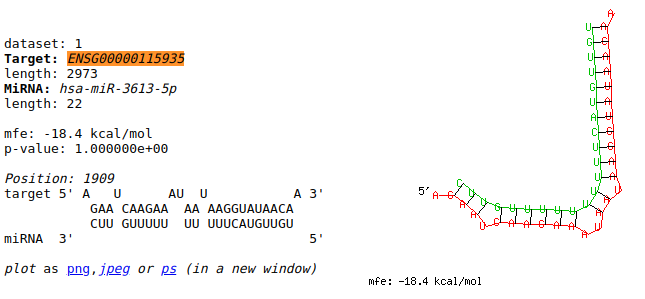** | -18.4 |
| miR5787 | WIPF1 | **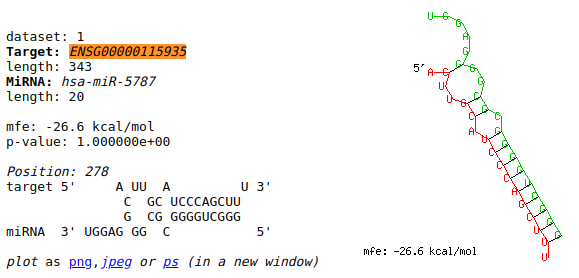** | -26.6 |
| miR6791 | WIPF1 | **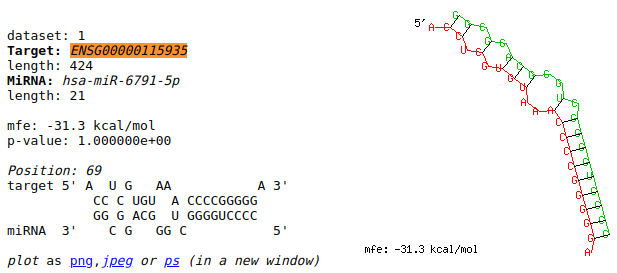** | -31.3 |
| miR6791 | AC108941.2 | **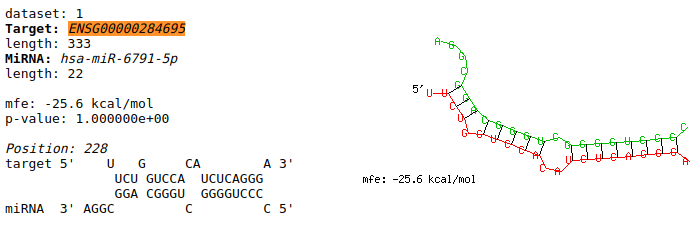** | -25.6 |
| miR330 | ADAMTSL1 | **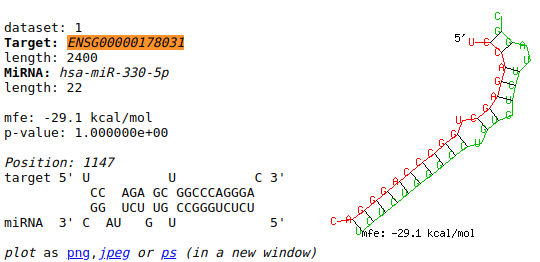** | -29.1 |
| miR3613 | ADAMTSL1 | **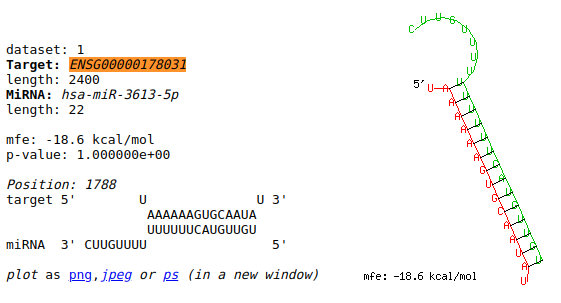** | -18.6 |
| miR5787 | ADAMTSL1 | **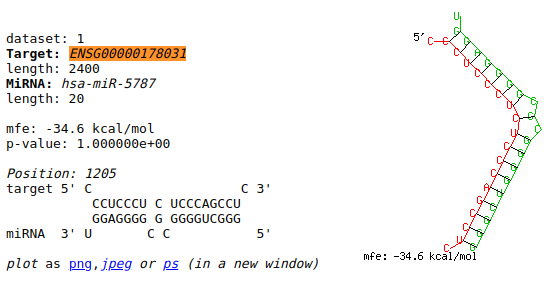** | -34.6 |
| miR6791 | ADAMTSL1 | **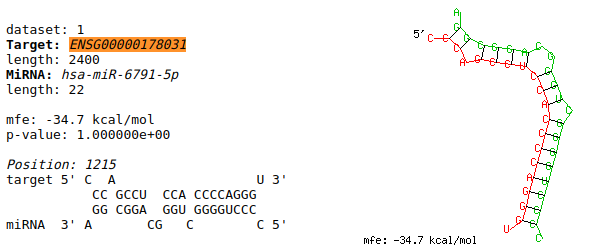** | -34.7 |
| miR330 | ARHGEF5 | **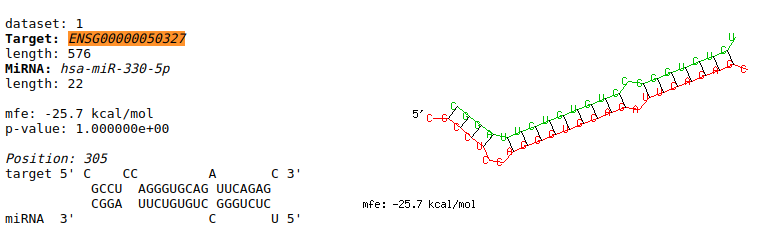** | -25.7 |
| miR5787 | ARHGEF5 | **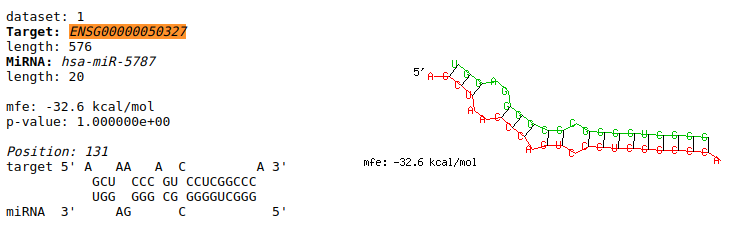** | -32.6 |
| miR6080 | ARHGEF5 | **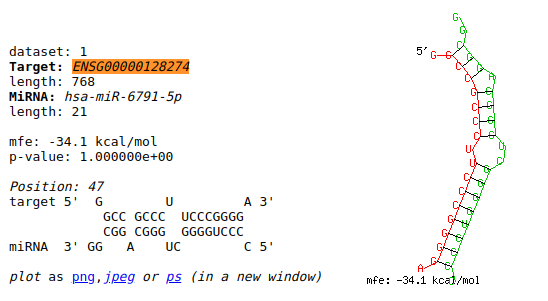** | -34.1 |
| miR330 | BNC1 | **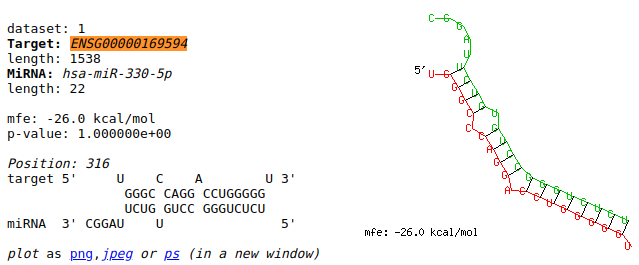** | -26.0 |
| miR5787 | BNC1 | **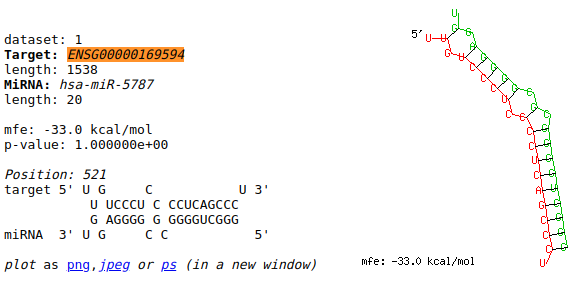** | -33.0 |
| miR6791 | BNC1 | **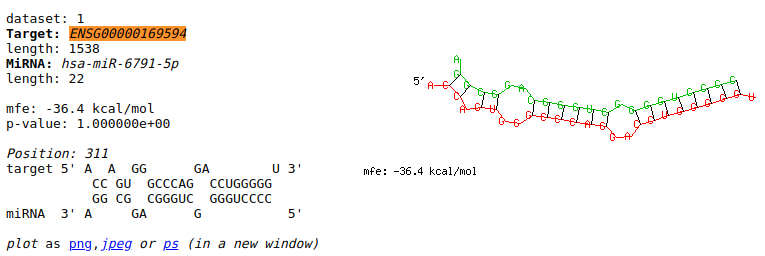** | -36.4 |
| miR5787 | CPA4 | **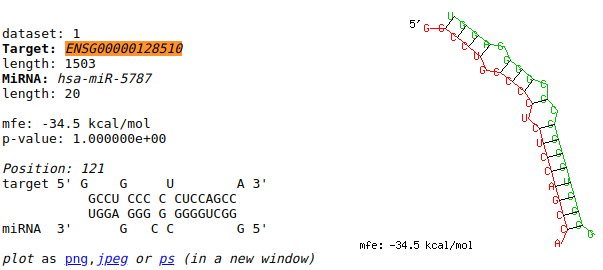** | -34.5 |
| miR5787 | PPL | **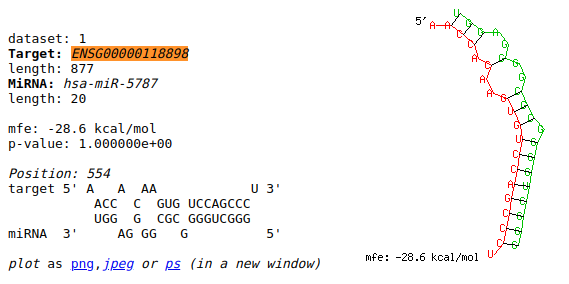** | -28.6 |
| miR5787 | TNFRSF10D | **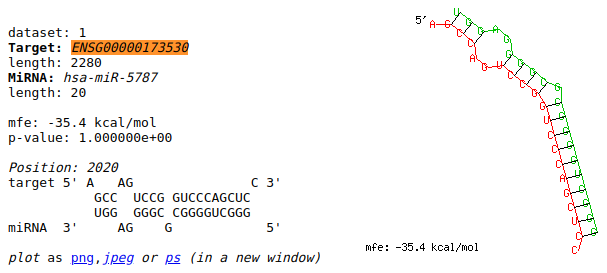** | -35.4 |
| miR6733 | TNFRSF10D | **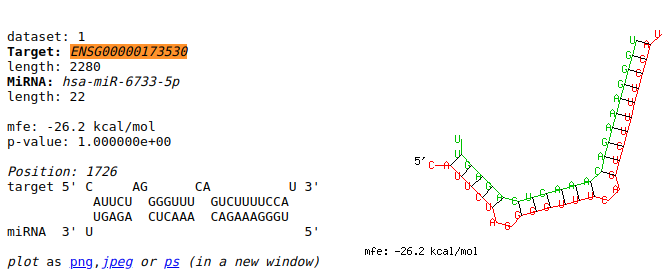** | -26.2 |
| miR6791 | TNFRSF10D | **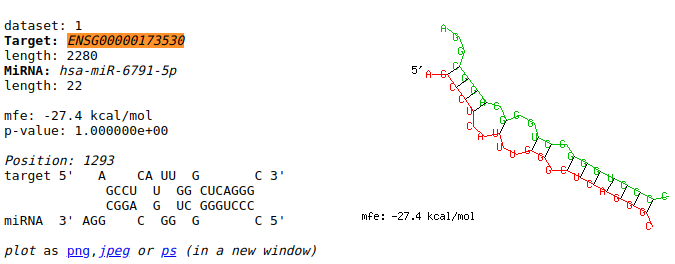** | -27.4 |
