# Supplementary material for: Analysis of H3K4me3-ChIP-Seq and RNA-Seq data to understand the putative role of miRNAs and their target genes in breast cancer cell lines
Source: Genomics Inform. 2021 Jun 30;19(2):e17. doi: 10.5808/gi.21020 (PMC8261273; doi:10.5808/gi.21020)
Supplement: Supplementary Table 18. — RNA hybrid analysis of miRNAs (present in at least three breast cancer cell lines) target genes with a probable role in breast cancer [file gi-21020suppl18.docx]

**Supplementary Table 18.** RNA hybrid analysis of miRNAs (present in at least three breast cancer cell lines) target genes with a probable role in breast cancer

| **miRNA** | **Putative target gene** | **RNA hybrid result** | **Binding energy (kcal/mol)** |
| --- | --- | --- | --- |
| Luminal-A - Nine gene targets by miRNAs | | | |
| miR3180-3 | A4GALT | **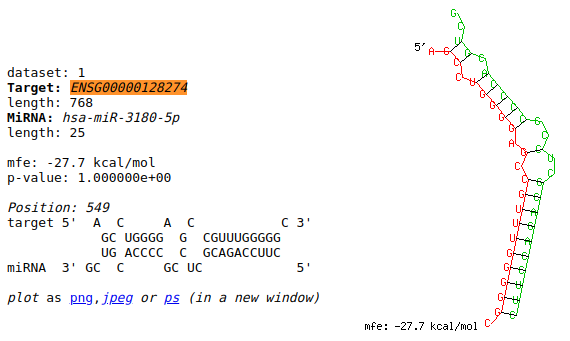** | -27.7 |
| miR4512 | A4GALT | **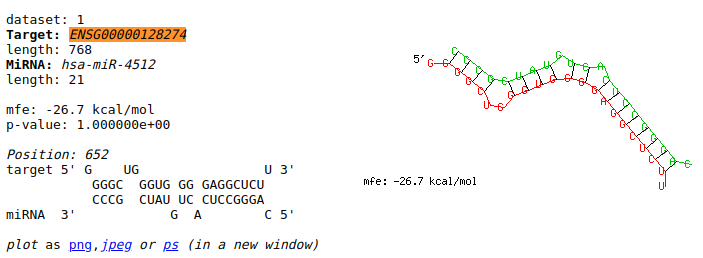** | -26.7 |
| miR6791 | A4GALT | **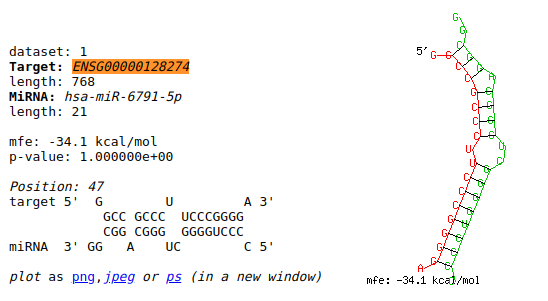** | -34.1 |
| miR330 | C10orf55 | **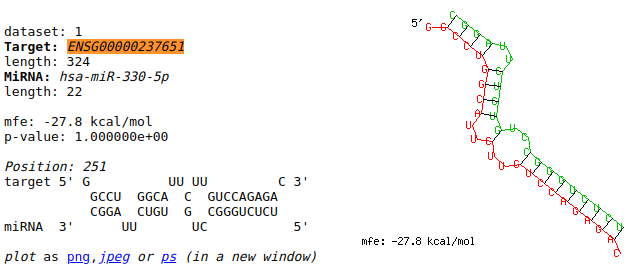** | -27.8 |
| miR3180-3 | C10orf55 | **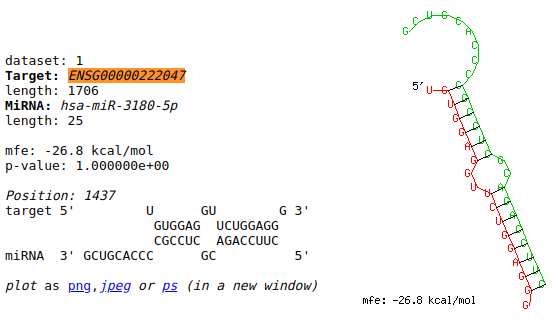** | -26.8 |
| miR5787 | C10orf55 | **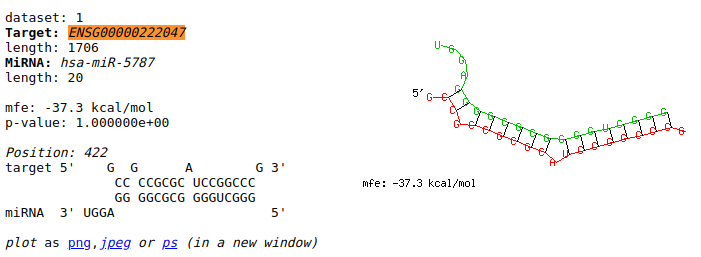** | -37.3 |
| miR6791 | C10orf55 | **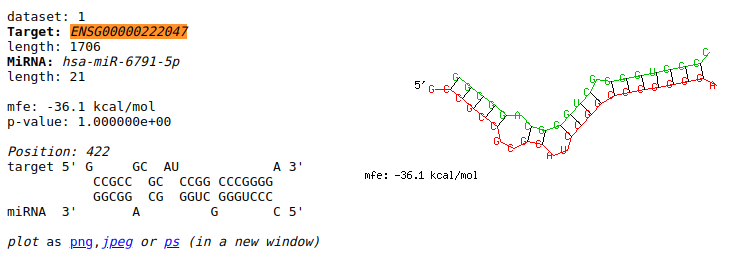** | -36.1 |
| miR330 | C2orf74 | **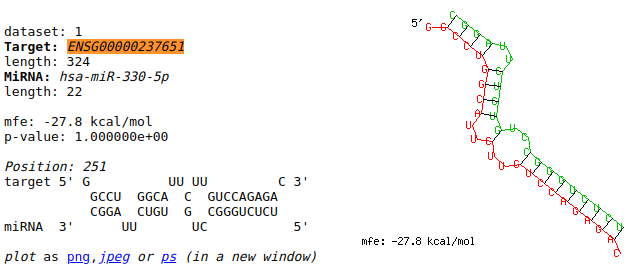** | -27.8 |
| miR5787 | C2orf74 | **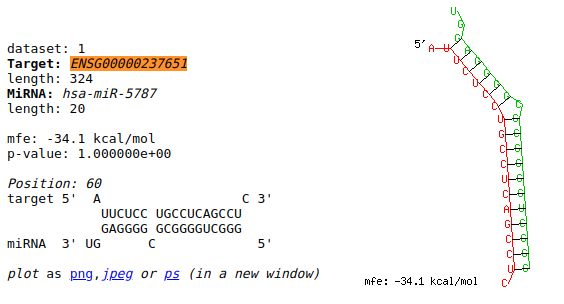** | -34.1 |
| miR330 | ZC4H2 | **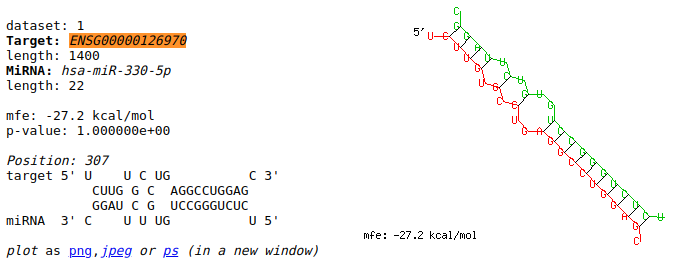** | -27.2 |
| miR5787 | ZC4H2 | **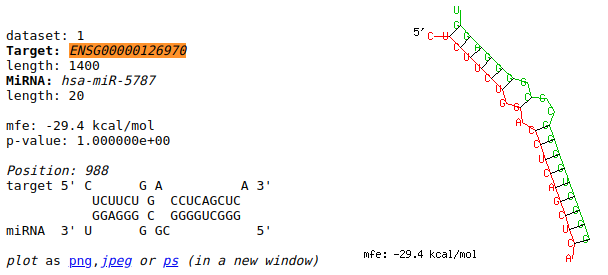** | -29.4 |
| miR3180-3 | ZNF512 | **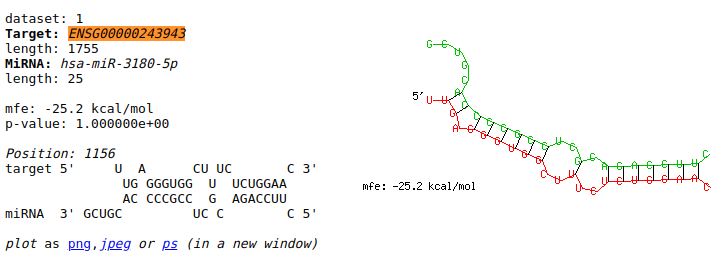** | -25.2 |
| miR5787 | ZNF512 | **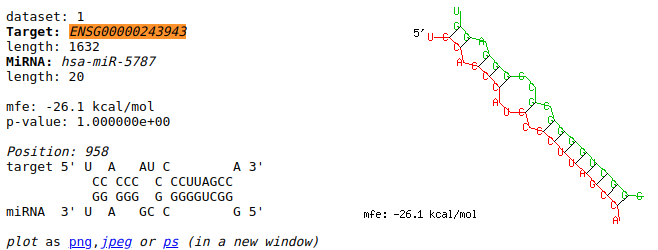** | -26.1 |
| miR6080 | ZNF512 | **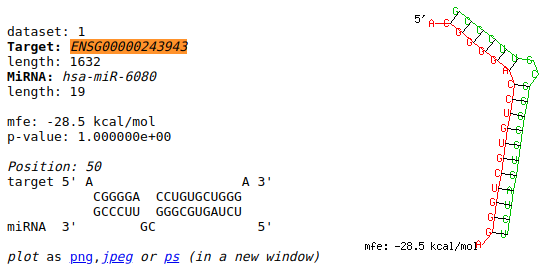** | -28.5 |
| miR6933 | ZNF512 | **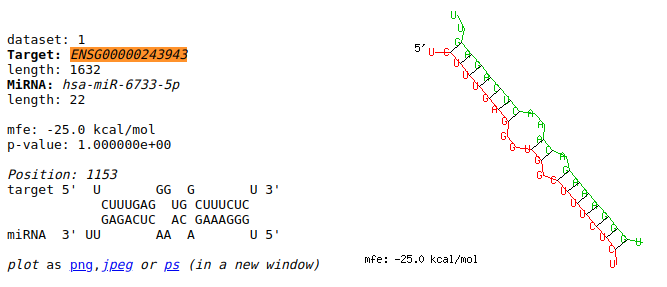** | -25.0 |
| miR5787 | ZNF655 | **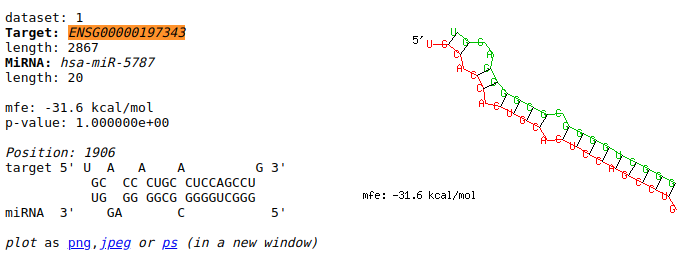** | -31.6 |
| miR5787 | ZNF71 | **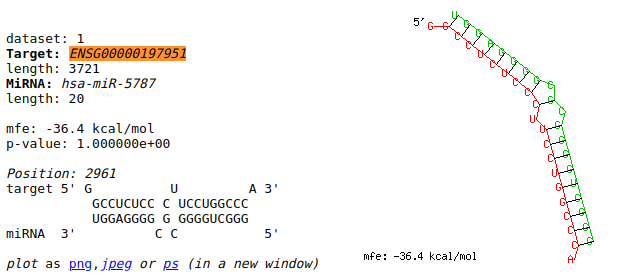** | -36.4 |
| miR6791 | ZNF71 | **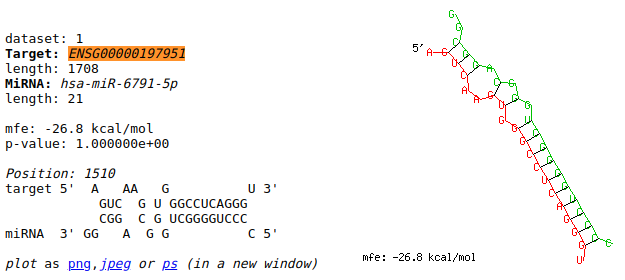** | -26.8 |
| miR6791 | HCG2042738 | **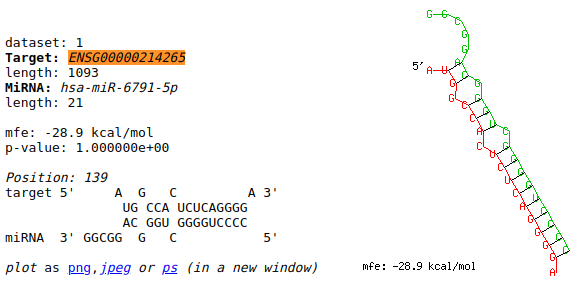** | -28.9 |
| miR4512 | HRCT1 | **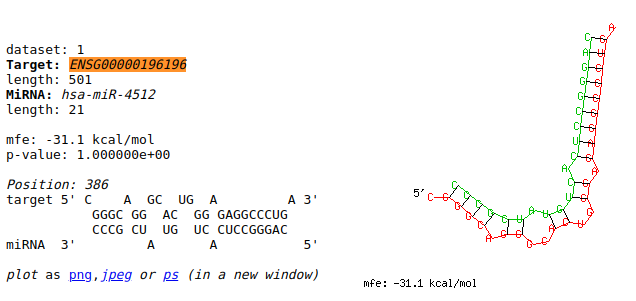** | -31.1 |
| miR5787 | HRCT1 | **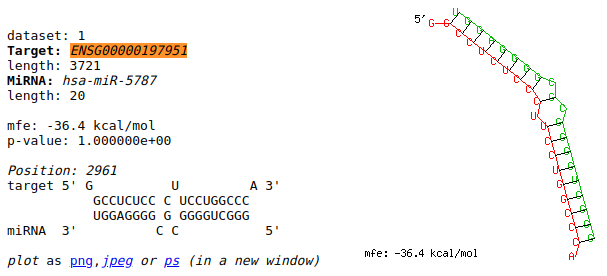** | -36.4 |
| TNBC - Three gene targets by miRNAs | | | |
| miR6791 | HIST3H2A | **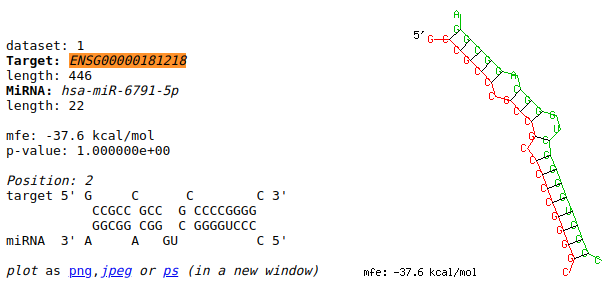** | -37.6 |
| miR5787 | ZNF608 | **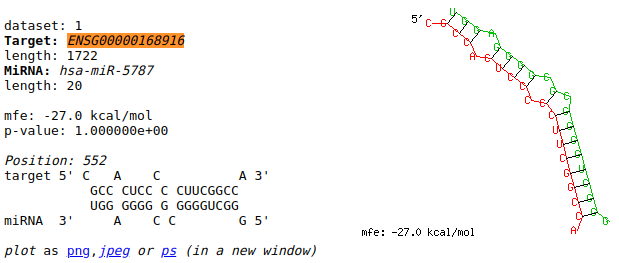** | -27.0 |
| miR5787 | ELOVL4 | **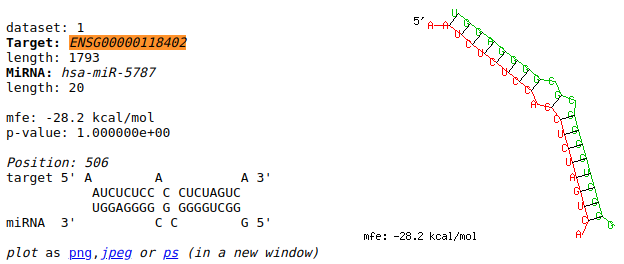** | -28.2 |
