# Supplementary material for: Analysis of H3K4me3-ChIP-Seq and RNA-Seq data to understand the putative role of miRNAs and their target genes in breast cancer cell lines
Source: Genomics Inform. 2021 Jun 30;19(2):e17. doi: 10.5808/gi.21020 (PMC8261273; doi:10.5808/gi.21020)
Supplement: Supplementary Table 19. — Details of eight miRNAs and their 3'-untranslated region targets based on downregulated genes obtained from differential expression analysis of normal-like vs. luminal-A cell lines [file gi-21020suppl19.docx]

**Supplementary Table 19.** Details of eight miRNAs and their 3'-untranslated region targets based on downregulated genes obtained from differential expression analysis of normal-like vs. luminal-A cell lines

Details of eight miRNAs and their 3' UTR targets based on down-regulated genes obtained from differenetial expression analysis of normal-like vs Luminal-A cell-lines

miRNA Gene baseMean log2FoldChang lfcSE stat pvalue padj MCF10A.RNA.S MCF10A.RNA.S MCF10A.RNA.S MCF10A.RNA.S MCF7.RNA.Seq. MCF7.RNA.Seq. MCF7.RNA.Seq. MCF7.RNA.Seq. ZR751.RNA.Seq ZR751.RNA.Seq ZR751.RNA.Seq ZR751.RNA.Seq.Rep4_sorted miR4512

miR3180

miR330

miR5787

miR3613

miR6080 miR6733 miR6791

ENSG00000104081 BMF 703.342 -2.274 1.282 -1.774 0.076 0.191 2221.773 770.617 2142.221 835.826 7.299 1.038 3.684 2.808 546.508 668.501 602.824 637.005

ENSG00000273173 SNURF 488.670 -2.152 1.647 -1.307 0.191 0.394 1056.481 974.281 1066.650 946.954 0.000 0.000 1.842 0.000 337.207 543.924 420.212 516.491

ENSG00000188015 S100A3 275.947 -2.278 1.337 -1.705 0.088 0.215 432.714 703.647 445.098 763.711 251.827 223.242 272.591 214.847 1.661 0.000 1.358 0.662

ENSG00000128274 A4GALT 118.335 -2.047 1.075 -1.904 0.057 0.151 320.107 188.067 306.315 143.048 145.257 96.565 111.431 96.892 3.322 7.018 0.679 1.324

ENSG00000196196 HRCT1 116.182 -2.449 1.530 -1.601 0.109 0.256 242.927 297.238 235.932 244.718 103.651 76.837 111.431 81.445 0.000 0.000 0.000 0.000

ENSG00000108342 CSF3 29.103 -2.282 1.316 -1.734 0.083 0.205 86.037 35.779 88.227 37.831 21.168 31.150 20.260 28.787 0.000 0.000 0.000 0.000

ENSG00000173706 HEG1 2385.381 -2.063 1.468 -1.405 0.160 0.344 6003.594 3754.008 5930.018 3670.777 2956.233 1691.445 2950.616 1648.565 9.967 0.000 3.394 5.960

ENSG00000115935 WIPF1 670.305 -2.439 1.264 -1.929 0.054 0.144 1724.531 1195.374 1781.384 1175.122 627.013 484.902 541.499 502.714 3.322 1.755 3.394 2.649

ENSG00000122861 PLAU 729.443 -2.573 1.668 -1.542 0.123 0.280 1181.740 2108.189 1132.076 2129.169 469.348 623.000 522.160 586.968 0.000 0.000 0.000 0.662

ENSG00000243943 ZNF512 315.720 -2.768 1.661 -1.667 0.096 0.230 905.916 566.954 867.396 588.743 240.878 175.478 240.359 202.911 0.000 0.000 0.000 0.000

ENSG00000188015 S100A3 275.947 -2.278 1.337 -1.705 0.088 0.215 432.714 703.647 445.098 763.711 251.827 223.242 272.591 214.847 1.661 0.000 1.358 0.662

ENSG00000128274 A4GALT 118.335 -2.047 1.075 -1.904 0.057 0.151 320.107 188.067 306.315 143.048 145.257 96.565 111.431 96.892 3.322 7.018 0.679 1.324

ENSG00000222047 C10orf55 123.875 -2.548 1.540 -1.655 0.098 0.234 177.135 371.548 202.227 357.029 80.293 99.680 91.171 107.424 0.000 0.000 0.000 0.000

ENSG00000177469 PTRF 6530.355 -2.172 1.432 -1.517 0.129 0.291 9571.590 18098.500 9318.316 17286.347 5997.138 6196.774 5849.660 6014.315 11.628 5.264 6.789 7.946

ENSG00000173706 HEG1 2385.381 -2.063 1.468 -1.405 0.160 0.344 6003.594 3754.008 5930.018 3670.777 2956.233 1691.445 2950.616 1648.565 9.967 0.000 3.394 5.960

ENSG00000089327 FXYD5 1267.229 -2.167 1.276 -1.698 0.090 0.218 2220.508 3141.183 2098.604 3060.755 1213.150 1211.735 1130.885 1107.235 8.306 7.018 3.394 3.973

ENSG00000188015 S100A3 275.947 -2.278 1.337 -1.705 0.088 0.215 432.714 703.647 445.098 763.711 251.827 223.242 272.591 214.847 1.661 0.000 1.358 0.662

ENSG00000237651 C2orf74 132.196 -2.358 1.305 -1.807 0.071 0.180 326.434 219.259 331.098 264.816 121.169 118.370 97.617 102.509 3.322 1.755 0.000 0.000

ENSG00000243649 CFB 112.123 -2.244 1.176 -1.909 0.056 0.150 265.702 183.480 302.350 195.065 125.549 80.990 113.273 73.722 3.322 0.000 1.358 0.662

ENSG00000126970 ZC4H2 89.787 -2.453 1.306 -1.878 0.060 0.159 235.336 173.389 224.036 156.052 0.000 1.038 0.000 0.702 66.445 66.675 86.894 66.879

ENSG00000222047 C10orf55 123.875 -2.548 1.540 -1.655 0.098 0.234 177.135 371.548 202.227 357.029 80.293 99.680 91.171 107.424 0.000 0.000 0.000 0.000

ENSG00000108342 CSF3 29.103 -2.282 1.316 -1.734 0.083 0.205 86.037 35.779 88.227 37.831 21.168 31.150 20.260 28.787 0.000 0.000 0.000 0.000

ENSG00000167601 AXL 4792.003 -2.385 1.597 -1.493 0.135 0.302 11158.209 9939.130 10935.143 9549.932 4284.712 3839.757 4224.243 3568.147 1.661 1.755 0.679 0.662

ENSG00000177469 PTRF 6530.355 -2.172 1.432 -1.517 0.129 0.291 9571.590 18098.500 9318.316 17286.347 5997.138 6196.774 5849.660 6014.315 11.628 5.264 6.789 7.946

ENSG00000173706 HEG1 2385.381 -2.063 1.468 -1.405 0.160 0.344 6003.594 3754.008 5930.018 3670.777 2956.233 1691.445 2950.616 1648.565 9.967 0.000 3.394 5.960

ENSG00000104081 BMF 703.342 -2.274 1.282 -1.774 0.076 0.191 2221.773 770.617 2142.221 835.826 7.299 1.038 3.684 2.808 546.508 668.501 602.824 637.005

ENSG00000089327 FXYD5 1267.229 -2.167 1.276 -1.698 0.090 0.218 2220.508 3141.183 2098.604 3060.755 1213.150 1211.735 1130.885 1107.235 8.306 7.018 3.394 3.973

ENSG00000134533 RERG 507.492 -3.199 1.748 -1.831 0.067 0.173 1842.199 563.285 1982.620 612.387 0.000 0.000 0.000 0.000 272.424 285.999 266.790 264.205

ENSG00000115935 WIPF1 670.305 -2.439 1.264 -1.929 0.054 0.144 1724.531 1195.374 1781.384 1175.122 627.013 484.902 541.499 502.714 3.322 1.755 3.394 2.649

ENSG00000197343 ZNF655 560.041 -2.407 1.249 -1.927 0.054 0.145 1353.814 1064.186 1346.199 1116.011 1.460 3.115 1.842 4.915 451.825 410.575 503.032 463.517

ENSG00000124813 RUNX2 439.752 -2.071 1.526 -1.357 0.175 0.367 1043.828 726.582 1075.572 729.427 476.647 393.528 416.254 412.843 1.661 0.000 0.679 0.000

ENSG00000243943 ZNF512 315.720 -2.768 1.661 -1.667 0.096 0.230 905.916 566.954 867.396 588.743 240.878 175.478 240.359 202.911 0.000 0.000 0.000 0.000

ENSG00000165983 PTER 244.149 -2.607 1.380 -1.890 0.059 0.155 560.504 532.093 601.725 511.899 199.272 155.750 211.811 151.657 3.322 1.755 0.000 0.000

ENSG00000167779 IGFBP6 306.228 -2.391 1.433 -1.669 0.095 0.229 544.056 871.532 545.221 699.871 317.521 207.667 283.642 202.209 1.661 0.000 1.358 0.000

ENSG00000188015 S100A3 275.947 -2.278 1.337 -1.705 0.088 0.215 432.714 703.647 445.098 763.711 251.827 223.242 272.591 214.847 1.661 0.000 1.358 0.662

ENSG00000163071 SPATA18 179.313 -2.404 1.603 -1.500 0.134 0.299 389.696 384.391 346.959 440.966 156.206 116.293 176.816 140.423 0.000 0.000 0.000 0.000

ENSG00000237651 C2orf74 132.196 -2.358 1.305 -1.807 0.071 0.180 326.434 219.259 331.098 264.816 121.169 118.370 97.617 102.509 3.322 1.755 0.000 0.000

ENSG00000243649 CFB 112.123 -2.244 1.176 -1.909 0.056 0.150 265.702 183.480 302.350 195.065 125.549 80.990 113.273 73.722 3.322 0.000 1.358 0.662

ENSG00000196196 HCRT1 116.182 -2.449 1.530 -1.601 0.109 0.256 242.927 297.238 235.932 244.718 103.651 76.837 111.431 81.445 0.000 0.000 0.000 0.000

ENSG00000126970 ZC4H2 89.787 -2.453 1.306 -1.878 0.060 0.159 235.336 173.389 224.036 156.052 0.000 1.038 0.000 0.702 66.445 66.675 86.894 66.879

ENSG00000197951 ZNF71 89.131 -2.312 1.542 -1.500 0.134 0.299 187.256 190.820 165.549 218.710 0.000 0.000 0.000 0.000 78.073 59.656 80.784 88.730

ENSG00000222047 C10orf55 123.875 -2.548 1.540 -1.655 0.098 0.234 177.135 371.548 202.227 357.029 80.293 99.680 91.171 107.424 0.000 0.000 0.000 0.000

ENSG00000174607 UGT8 79.229 -2.391 1.226 -1.950 0.051 0.139 163.217 175.224 156.627 192.701 0.000 2.077 0.000 1.404 69.767 59.656 65.849 64.230

ENSG00000115935 WIPF1 670.305 -2.439 1.264 -1.929 0.054 0.144 1724.531 1195.374 1781.384 1175.122 627.013 484.902 541.499 502.714 3.322 1.755 3.394 2.649

ENSG00000174607 UGT8 79.229 -2.391 1.226 -1.950 0.051 0.139 163.217 175.224 156.627 192.701 0.000 2.077 0.000 1.404 69.767 59.656 65.849 64.230

ENSG00000243943 ZNF512 315.720 -2.768 1.661 -1.667 0.096 0.230 905.916 566.954 867.396 588.743 240.878 175.478 240.359 202.911 0.000 0.000 0.000 0.000

ENSG00000243943 ZNF512 315.720 -2.768 1.661 -1.667 0.096 0.230 905.916 566.954 867.396 588.743 240.878 175.478 240.359 202.911 0.000 0.000 0.000 0.000

ENSG00000177469 PTRF 6530.355 -2.172 1.432 -1.517 0.129 0.291 9571.590 18098.500 9318.316 17286.347 5997.138 6196.774 5849.660 6014.315 11.628 5.264 6.789 7.946

ENSG00000173706 HEG1 2385.381 -2.063 1.468 -1.405 0.160 0.344 6003.594 3754.008 5930.018 3670.777 2956.233 1691.445 2950.616 1648.565 9.967 0.000 3.394 5.960

ENSG00000104081 BMF 703.342 -2.274 1.282 -1.774 0.076 0.191 2221.773 770.617 2142.221 835.826 7.299 1.038 3.684 2.808 546.508 668.501 602.824 637.005

ENSG00000089327 FXYD5 1267.229 -2.167 1.276 -1.698 0.090 0.218 2220.508 3141.183 2098.604 3060.755 1213.150 1211.735 1130.885 1107.235 8.306 7.018 3.394 3.973

ENSG00000115935 WIPF1 670.305 -2.439 1.264 -1.929 0.054 0.144 1724.531 1195.374 1781.384 1175.122 627.013 484.902 541.499 502.714 3.322 1.755 3.394 2.649

ENSG00000273173 SNURF 488.670 -2.152 1.647 -1.307 0.191 0.394 1056.481 974.281 1066.650 946.954 0.000 0.000 1.842 0.000 337.207 543.924 420.212 516.491

ENSG00000124813 RUNX2 439.752 -2.071 1.526 -1.357 0.175 0.367 1043.828 726.582 1075.572 729.427 476.647 393.528 416.254 412.843 1.661 0.000 0.679 0.000

ENSG00000214265 SNURF 380.675 -2.030 1.772 -1.146 0.252 0.484 833.797 764.196 797.013 671.498 0.000 0.000 0.000 0.000 282.390 450.931 329.924 438.355

ENSG00000188015 S100A3 275.947 -2.278 1.337 -1.705 0.088 0.215 432.714 703.647 445.098 763.711 251.827 223.242 272.591 214.847 1.661 0.000 1.358 0.662

ENSG00000128274 A4GALT 118.335 -2.047 1.075 -1.904 0.057 0.151 320.107 188.067 306.315 143.048 145.257 96.565 111.431 96.892 3.322 7.018 0.679 1.324

ENSG00000243649 CFB 112.123 -2.244 1.176 -1.909 0.056 0.150 265.702 183.480 302.350 195.065 125.549 80.990 113.273 73.722 3.322 0.000 1.358 0.662

ENSG00000197951 ZNF71 89.131 -2.312 1.542 -1.500 0.134 0.299 187.256 190.820 165.549 218.710 0.000 0.000 0.000 0.000 78.073 59.656 80.784 88.730

ENSG00000222047 C10orf55 123.875 -2.548 1.540 -1.655 0.098 0.234 177.135 371.548 202.227 357.029 80.293 99.680 91.171 107.424 0.000 0.000 0.000 0.000

| **Details of eight miRNAs and their 3' UTR targets based on down-regulated genes obtained from differenetial expression analysis of normal-like vs Luminal-A cell-lines** | | | | | | | | | | | | |  |  |  |  |  |  |  |  |
| --- | --- | --- | --- | --- | --- | --- | --- | --- | --- | --- | --- | --- | --- | --- | --- | --- | --- | --- | --- | --- |
| **miRNA** | **Gene** |  | **baseMean** | **log2FoldChang** | **lfcSE** | **stat** | **pvalue** | **padj** | **MCF10A.RNA.S** | **MCF10A.RNA.S** | **MCF10A.RNA.S** | **MCF10A.RNA.S** | **MCF7.RNA.Seq.** | **MCF7.RNA.Seq.** | **MCF7.RNA.Seq.** | **MCF7.RNA.Seq.** | **ZR751.RNA.Seq** | **ZR751.RNA.Seq** | **ZR751.RNA.Seq** | **ZR751.RNA.Seq.Rep4_sorted** |
|  | ENSG00000174607 | UGT8 | 79.229 | -2.391 | 1.226 | -1.950 | 0.051 | 0.139 | 163.217 | 175.224 | 156.627 | 192.701 | 0.000 | 2.077 | 0.000 | 1.404 | 69.767 | 59.656 | 65.849 | 64.230 |
|  | ENSG00000108342 | CSF3 | 29.103 | -2.282 | 1.316 | -1.734 | 0.083 | 0.205 | 86.037 | 35.779 | 88.227 | 37.831 | 21.168 | 31.150 | 20.260 | 28.787 | 0.000 | 0.000 | 0.000 | 0.000 |
|  |  |  |  |  |  |  |  |  |  |  |  |  |  |  |  |  |  |  |  |  |
