# Supplementary material for: Analysis of H3K4me3-ChIP-Seq and RNA-Seq data to understand the putative role of miRNAs and their target genes in breast cancer cell lines
Source: Genomics Inform. 2021 Jun 30;19(2):e17. doi: 10.5808/gi.21020 (PMC8261273; doi:10.5808/gi.21020)
Supplement: Supplementary Table 20. — Details of eight miRNAs and their 3'-untranslated region targets based on downregulated genes obtained from differential expression analysis of normal-like vs. triple-negative breast cancer cell lines [file gi-21020suppl20.docx]

**Supplementary Table 20.** Details of eight miRNAs and their 3'-untranslated region targets based on downregulated genes obtained from differential expression analysis of normal-like vs. triple-negative breast cancer cell lines

| **Details of eight miRNAs and their 3' UTR targets based on down-regulated genes obtained from differenetial expression analysis of normal-like vs TNBC cell-lines** | | | | | | | | | | | | |  |  |  |  |  |  |  |  |
| --- | --- | --- | --- | --- | --- | --- | --- | --- | --- | --- | --- | --- | --- | --- | --- | --- | --- | --- | --- | --- |
|  | **Gene** | **Gene name** | **baseMean** | **log2FoldChang** | **lfcSE** | **stat** | **pvalue** | **padj** | **MCF10A.RNA.Seq.Rep1_sorted** | **MCF10A.RNA.Seq.Rep2_sorted** | **MCF10A.RNA.Seq.Rep3_sorted** | **MCF10A.RNA.Seq.Rep4_sorted** | **MB231.RNA.Seq.Rep1_sorted** | **MB231.RNA.Seq.Rep2_sorted** | **MB231.RNA.Seq.Rep3_sorted** | **MB231.RNA.Seq.Rep4_sorted** | **MB436.RNA.Seq.Rep1_sorted** | **MB436.RNA.Seq.Rep2_sorted** | **MB436.RNA.Seq.Rep3_sorted** | **MB436.RNA.Seq.Rep4_sorted** |
| **miR4512** |  |  |  |  |  |  |  |  |  |  |  |  |  |  |  |  |  |  |  |  |
|  | ENSG00000116132 | PRRX1 | 81.310 | -2.193 | 1.575 | -1.392 | 0.164 | 0.280 | 178.422 | 149.916 | 176.290 | 173.384 | 0.000 | 0.000 | 0.000 | 0.616 | 50.194 | 77.887 | 78.676 | 90.330 |
| **miR3180** |  |  |  |  |  |  |  |  |  |  |  |  |  |  |  |  |  |  |  |  |
|  | ENSG00000176046 | NUPR1 | 217.166 | -2.166 | 1.106 | -1.959 | 0.050 | 0.111 | 584.107 | 351.478 | 531.991 | 336.568 | 380.809 | 3.892 | 360.241 | 4.311 | 8.366 | 20.863 | 4.141 | 19.219 |
|  | ENSG00000196159 | FAT4 | 211.139 | -2.016 | 1.050 | -1.921 | 0.055 | 0.119 | 470.475 | 354.347 | 446.966 | 425.578 | 195.734 | 216.962 | 177.499 | 231.556 | 2.789 | 6.954 | 0.000 | 4.805 |
| **miR330** |  |  |  |  |  |  |  |  |  |  |  |  |  |  |  |  |  |  |  |  |
|  | ENSG00000113739 | STC2 | 2908.398 | -2.342 | 1.343 | -1.744 | 0.081 | 0.164 | 5863.000 | 6650.112 | 5929.124 | 6583.945 | 30.038 | 4.865 | 34.451 | 5.543 | 1779.082 | 3193.384 | 1706.024 | 3121.207 |
|  | ENSG00000050327 | ARHGEF5 | 318.147 | -2.729 | 1.417 | -1.926 | 0.054 | 0.118 | 896.096 | 614.728 | 820.608 | 602.671 | 271.314 | 162.478 | 264.377 | 179.825 | 2.789 | 0.000 | 0.000 | 2.883 |
|  | ENSG00000169594 | BNC1 | 361.537 | -2.895 | 1.947 | -1.487 | 0.137 | 0.245 | 629.959 | 1104.646 | 605.315 | 1078.317 | 0.000 | 0.000 | 0.000 | 0.000 | 225.871 | 197.500 | 281.577 | 215.256 |
|  | ENSG00000176046 | NUPR1 | 217.166 | -2.166 | 1.106 | -1.959 | 0.050 | 0.111 | 584.107 | 351.478 | 531.991 | 336.568 | 380.809 | 3.892 | 360.241 | 4.311 | 8.366 | 20.863 | 4.141 | 19.219 |
|  | ENSG00000123364 | HOXC13 | 115.856 | -3.049 | 1.728 | -1.765 | 0.078 | 0.158 | 277.102 | 306.288 | 245.714 | 289.282 | 0.000 | 0.000 | 0.000 | 0.000 | 52.982 | 61.197 | 95.239 | 62.463 |
|  | ENSG00000178031 | ADAMTSL1 | 94.452 | -2.017 | 1.688 | -1.195 | 0.232 | 0.364 | 177.425 | 201.562 | 195.791 | 184.510 | 58.139 | 117.724 | 72.647 | 125.631 | 0.000 | 0.000 | 0.000 | 0.000 |
| **miR5787** |  |  |  |  |  |  |  |  |  |  |  |  |  |  |  |  |  |  |  |  |
|  | ENSG00000118898 | PPL | 2244.885 | -2.565 | 1.388 | -1.848 | 0.065 | 0.136 | 7855.543 | 2131.823 | 7929.940 | 2216.901 | 3070.698 | 330.794 | 2944.843 | 400.912 | 18.125 | 18.081 | 4.141 | 16.817 |
|  | ENSG00000113739 | STC2 | 2908.398 | -2.342 | 1.343 | -1.744 | 0.081 | 0.164 | 5863.000 | 6650.112 | 5929.124 | 6583.945 | 30.038 | 4.865 | 34.451 | 5.543 | 1779.082 | 3193.384 | 1706.024 | 3121.207 |
|  | ENSG00000168916 | ZNF608 | 662.680 | -2.639 | 1.613 | -1.636 | 0.102 | 0.195 | 2182.926 | 913.126 | 2024.998 | 899.370 | 733.518 | 278.257 | 653.827 | 262.348 | 0.000 | 1.391 | 0.000 | 2.402 |
|  | ENSG00000050327 | ARHGEF5 | 318.147 | -2.729 | 1.417 | -1.926 | 0.054 | 0.118 | 896.096 | 614.728 | 820.608 | 602.671 | 271.314 | 162.478 | 264.377 | 179.825 | 2.789 | 0.000 | 0.000 | 2.883 |
|  | ENSG00000121858 | TNFSF10 | 253.412 | -2.166 | 1.764 | -1.228 | 0.219 | 0.349 | 834.296 | 254.642 | 761.324 | 254.049 | 398.251 | 76.861 | 405.927 | 54.194 | 1.394 | 0.000 | 0.000 | 0.000 |
|  | ENSG00000173530 | TNFRSF10D | 506.436 | -2.152 | 1.584 | -1.358 | 0.174 | 0.294 | 790.439 | 1289.710 | 799.546 | 1311.968 | 435.072 | 507.867 | 431.391 | 507.452 | 0.000 | 1.391 | 0.000 | 2.402 |
|  | ENSG00000169594 | BNC1 | 361.537 | -2.895 | 1.947 | -1.487 | 0.137 | 0.245 | 629.959 | 1104.646 | 605.315 | 1078.317 | 0.000 | 0.000 | 0.000 | 0.000 | 225.871 | 197.500 | 281.577 | 215.256 |
|  | ENSG00000176046 | NUPR1 | 217.166 | -2.166 | 1.106 | -1.959 | 0.050 | 0.111 | 584.107 | 351.478 | 531.991 | 336.568 | 380.809 | 3.892 | 360.241 | 4.311 | 8.366 | 20.863 | 4.141 | 19.219 |
|  | ENSG00000147027 | TMEM47 | 162.557 | -2.631 | 1.520 | -1.731 | 0.083 | 0.167 | 297.037 | 397.386 | 301.097 | 478.428 | 0.000 | 0.973 | 0.000 | 0.616 | 136.638 | 127.958 | 95.239 | 115.316 |
|  | ENSG00000123364 | HOXC13 | 115.856 | -3.049 | 1.728 | -1.765 | 0.078 | 0.158 | 277.102 | 306.288 | 245.714 | 289.282 | 0.000 | 0.000 | 0.000 | 0.000 | 52.982 | 61.197 | 95.239 | 62.463 |
|  | ENSG00000128510 | CPA4 | 121.750 | -2.082 | 1.074 | -1.938 | 0.053 | 0.116 | 271.121 | 215.908 | 276.136 | 230.869 | 90.115 | 135.237 | 89.124 | 145.954 | 4.183 | 1.391 | 0.000 | 0.961 |
|  | ENSG00000116132 | PRRX1 | 81.310 | -2.193 | 1.575 | -1.392 | 0.164 | 0.280 | 178.422 | 149.916 | 176.290 | 173.384 | 0.000 | 0.000 | 0.000 | 0.616 | 50.194 | 77.887 | 78.676 | 90.330 |
|  | ENSG00000178031 | ADAMTSL1 | 94.452 | -2.017 | 1.688 | -1.195 | 0.232 | 0.364 | 177.425 | 201.562 | 195.791 | 184.510 | 58.139 | 117.724 | 72.647 | 125.631 | 0.000 | 0.000 | 0.000 | 0.000 |
|  | ENSG00000118402 | ELOVL4 | 46.524 | -2.979 | 1.582 | -1.883 | 0.060 | 0.128 | 115.625 | 101.140 | 120.907 | 107.554 | 0.000 | 0.000 | 0.000 | 0.000 | 15.337 | 45.898 | 12.423 | 39.399 |
| **miR3613** |  |  |  |  |  |  |  |  |  |  |  |  |  |  |  |  |  |  |  |  |
|  | ENSG00000145703 | IQGAP2 | 104.856 | -2.572 | 1.432 | -1.796 | 0.072 | 0.149 | 342.889 | 109.747 | 382.222 | 104.772 | 0.969 | 0.000 | 2.247 | 0.000 | 86.444 | 62.588 | 115.943 | 50.451 |
|  | ENSG00000116132 | PRRX1 | 81.310 | -2.193 | 1.575 | -1.392 | 0.164 | 0.280 | 178.422 | 149.916 | 176.290 | 173.384 | 0.000 | 0.000 | 0.000 | 0.616 | 50.194 | 77.887 | 78.676 | 90.330 |
|  | ENSG00000178031 | ADAMTSL1 | 94.452 | -2.017 | 1.688 | -1.195 | 0.232 | 0.364 | 177.425 | 201.562 | 195.791 | 184.510 | 58.139 | 117.724 | 72.647 | 125.631 | 0.000 | 0.000 | 0.000 | 0.000 |
| **miR6080** |  |  |  |  |  |  |  |  |  |  |  |  |  |  |  |  |  |  |  |  |
|  | ENSG00000050327 | ARHGEF5 (Rho G | 318.147 | -2.729 | 1.417 | -1.926 | 0.054 | 0.118 | 896.096 | 614.728 | 820.608 | 602.671 | 271.314 | 162.478 | 264.377 | 179.825 | 2.789 | 0.000 | 0.000 | 2.883 |
| **miR6733** |  |  |  |  |  |  |  |  |  |  |  |  |  |  |  |  |  |  |  |  |
|  | ENSG00000173530 | TNFRSF10D | 506.436 | -2.152 | 1.584 | -1.358 | 0.174 | 0.294 | 790.439 | 1289.710 | 799.546 | 1311.968 | 435.072 | 507.867 | 431.391 | 507.452 | 0.000 | 1.391 | 0.000 | 2.402 |
| **miR6791** |  |  |  |  |  |  |  |  |  |  |  |  |  |  |  |  |  |  |  |  |
|  | ENSG00000113739 | STC2 | 2908.398 | -2.342 | 1.343 | -1.744 | 0.081 | 0.164 | 5863.000 | 6650.112 | 5929.124 | 6583.945 | 30.038 | 4.865 | 34.451 | 5.543 | 1779.082 | 3193.384 | 1706.024 | 3121.207 |
|  | ENSG00000173530 | TNFRSF10D | 506.436 | -2.152 | 1.584 | -1.358 | 0.174 | 0.294 | 790.439 | 1289.710 | 799.546 | 1311.968 | 435.072 | 507.867 | 431.391 | 507.452 | 0.000 | 1.391 | 0.000 | 2.402 |
|  | ENSG00000169594 | BNC1 | 361.537 | -2.895 | 1.947 | -1.487 | 0.137 | 0.245 | 629.959 | 1104.646 | 605.315 | 1078.317 | 0.000 | 0.000 | 0.000 | 0.000 | 225.871 | 197.500 | 281.577 | 215.256 |
|  | ENSG00000176046 | NUPR1 | 217.166 | -2.166 | 1.106 | -1.959 | 0.050 | 0.111 | 584.107 | 351.478 | 531.991 | 336.568 | 380.809 | 3.892 | 360.241 | 4.311 | 8.366 | 20.863 | 4.141 | 19.219 |
|  | ENSG00000116132 | PRRX1 | 81.310 | -2.193 | 1.575 | -1.392 | 0.164 | 0.280 | 178.422 | 149.916 | 176.290 | 173.384 | 0.000 | 0.000 | 0.000 | 0.616 | 50.194 | 77.887 | 78.676 | 90.330 |
|  | ENSG00000178031 | ADAMTSL1 | 94.452 | -2.017 | 1.688 | -1.195 | 0.232 | 0.364 | 177.425 | 201.562 | 195.791 | 184.510 | 58.139 | 117.724 | 72.647 | 125.631 | 0.000 | 0.000 | 0.000 | 0.000 |
|  | ENSG00000181218 | HIST3H2A | 43.612 | -2.186 | 1.678 | -1.303 | 0.193 | 0.318 | 128.583 | 49.494 | 131.828 | 53.777 | 0.000 | 0.000 | 0.000 | 0.000 | 34.857 | 54.243 | 12.423 | 58.138 |
|  | ENSG00000284695 | AC108941.2 | 19.228 | -2.091 | 1.122 | -1.864 | 0.062 | 0.132 | 68.777 | 12.911 | 66.304 | 8.345 | 0.000 | 0.000 | 0.749 | 0.616 | 19.520 | 19.472 | 12.423 | 21.622 |
|  |  |  |  |  |  |  |  |  |  |  |  |  |  |  |  |  |  |  |  |  |
