# Supplementary material for: Analysis of H3K4me3-ChIP-Seq and RNA-Seq data to understand the putative role of miRNAs and their target genes in breast cancer cell lines
Source: Genomics Inform. 2021 Jun 30;19(2):e17. doi: 10.5808/gi.21020 (PMC8261273; doi:10.5808/gi.21020)
Supplement: Supplementary Fig. 1. — Workflow for differential expression analysis using RNA-Seq data. [file gi-21020suppl21.pdf]

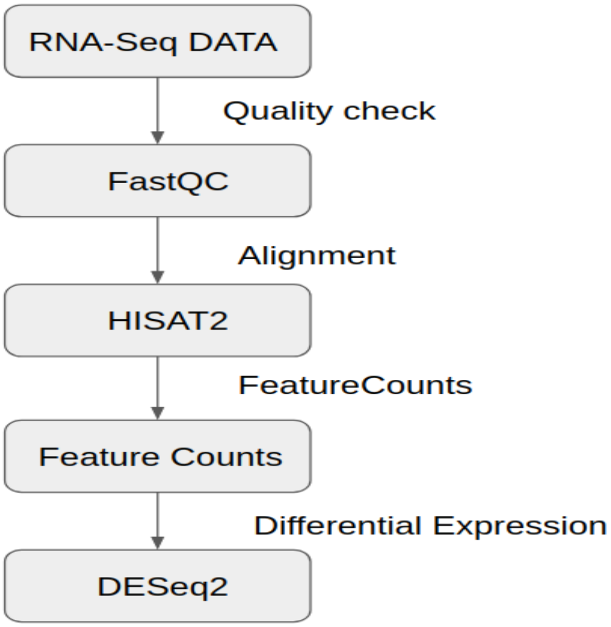

Supplementary Fig. 1. Workflow for differential expression analysis using RNA sequencing (RNA-Seq) data.
