# Supplementary material for: Analysis of H3K4me3-ChIP-Seq and RNA-Seq data to understand the putative role of miRNAs and their target genes in breast cancer cell lines
Source: Genomics Inform. 2021 Jun 30;19(2):e17. doi: 10.5808/gi.21020 (PMC8261273; doi:10.5808/gi.21020)
Supplement: Supplementary Fig. 2. — FastQC output of chromatin immunoprecipitation sequencing data pertaining to each cell line and their corresponding biological replicates. [file gi-21020suppl22.pdf]

<table>
<tr><td>MCF10A-Rep1
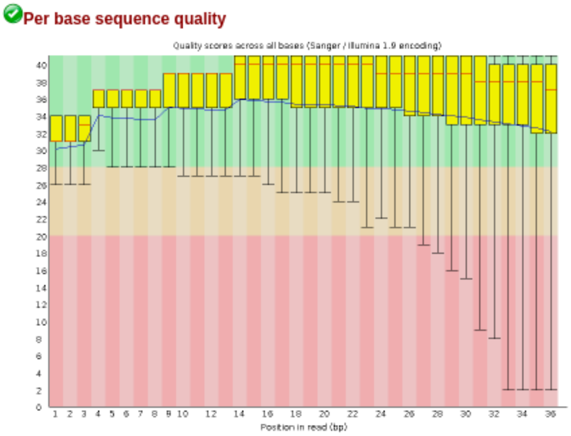
</td><td>MCF10A-Rep2
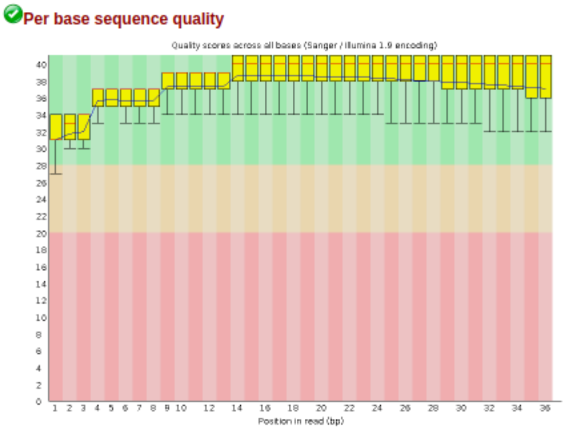
</td></tr>
<tr><td>MCF7-Rep1
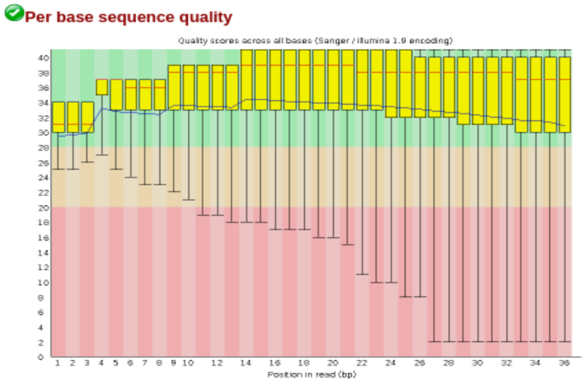
</td><td>MCF7-Rep2
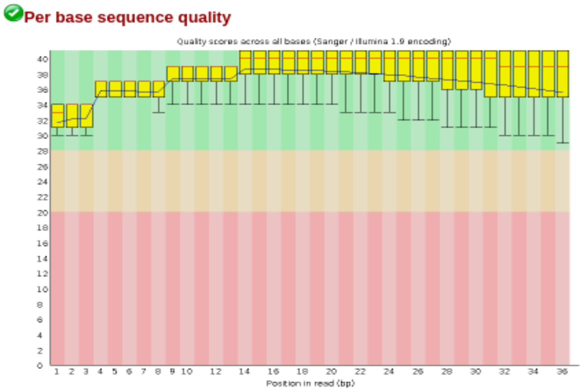
</td></tr>
<tr><td>ZR751-Rep1
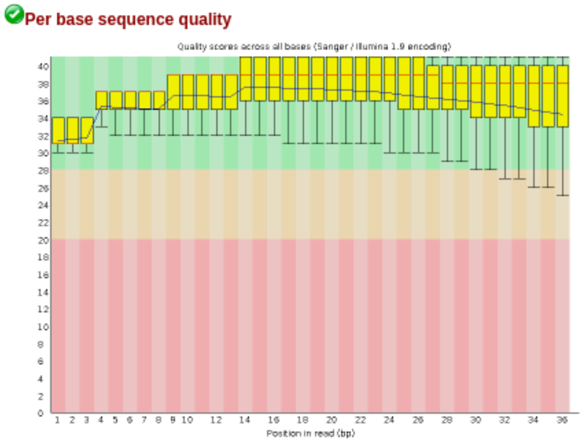
</td><td>ZR751-Rep2
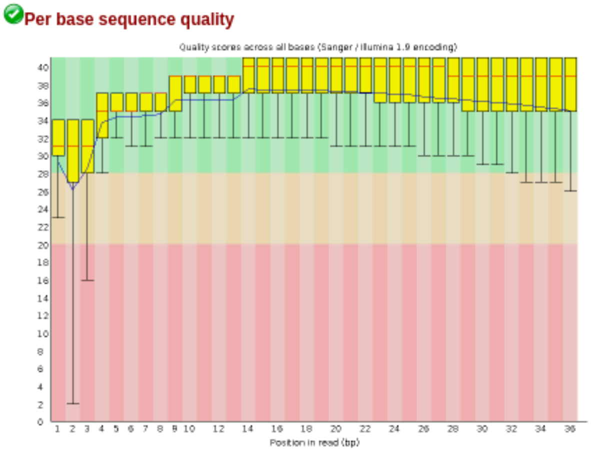
</td></tr>
<tr><td>MB231-Rep1</td><td>MB231-Rep2</td></tr>
</table>

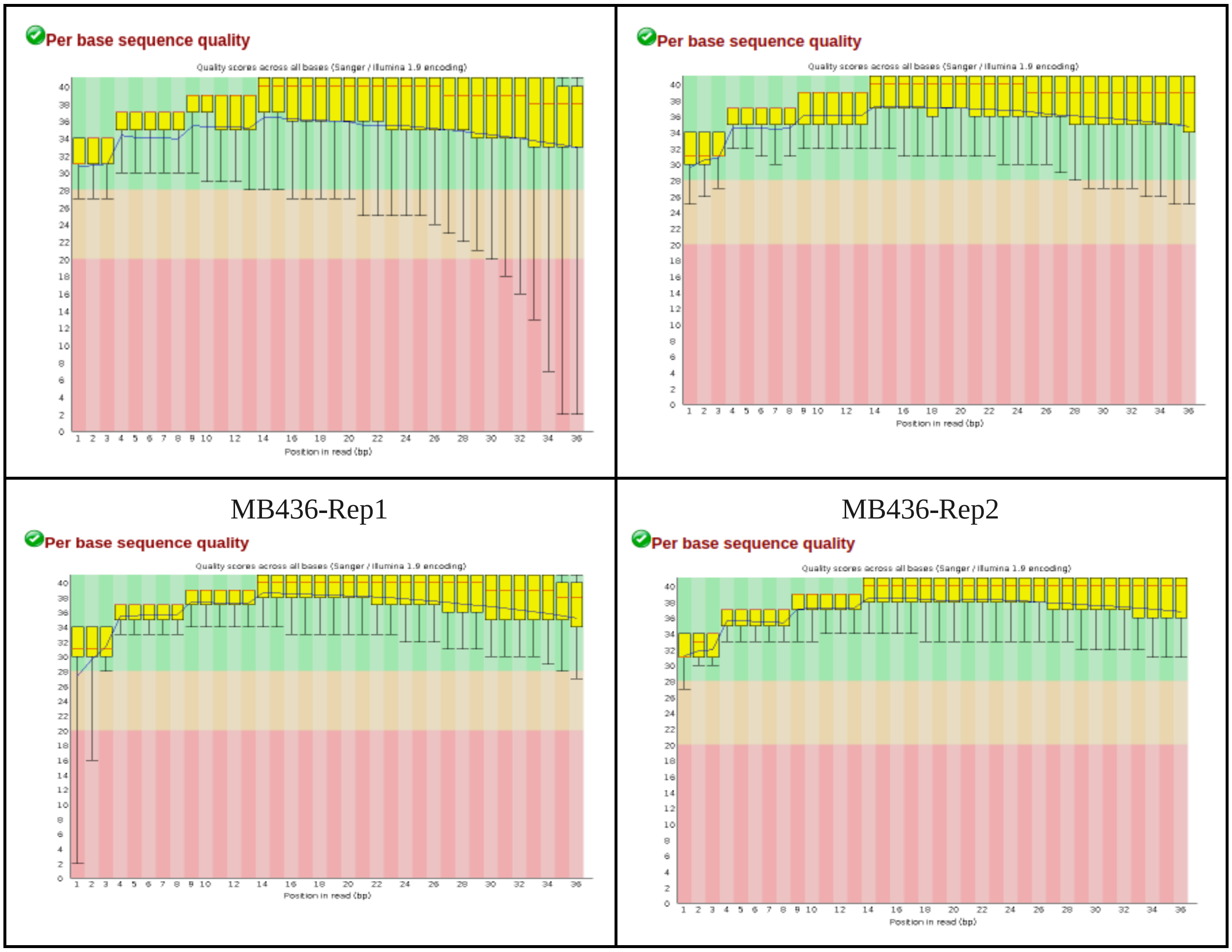

Supplementary Fig. 2. FastQC output of chromatin immunoprecipitation sequencing data pertaining to each cell-line and their corresponding biological replicates.
