# Supplementary material for: Analysis of H3K4me3-ChIP-Seq and RNA-Seq data to understand the putative role of miRNAs and their target genes in breast cancer cell lines
Source: Genomics Inform. 2021 Jun 30;19(2):e17. doi: 10.5808/gi.21020 (PMC8261273; doi:10.5808/gi.21020)
Supplement: Supplementary Fig. 3. — Reproducibility analysis of replicates belonging to normal-like cell line (MCF10A). [file gi-21020suppl23.pdf]

MCF10A

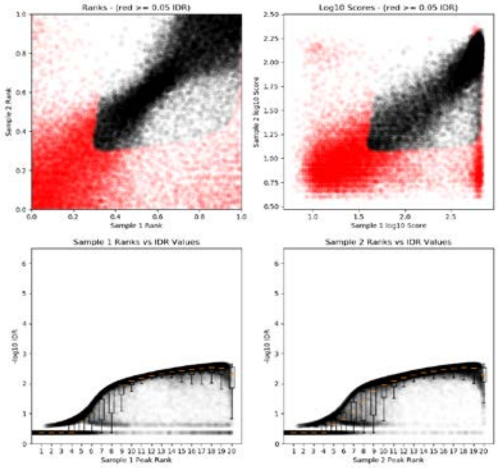

Supplementary Fig. 3. Reproducibility analysis of replicates belonging to normal-like cell line (MCF10A).
