# Supplementary material for: Analysis of H3K4me3-ChIP-Seq and RNA-Seq data to understand the putative role of miRNAs and their target genes in breast cancer cell lines
Source: Genomics Inform. 2021 Jun 30;19(2):e17. doi: 10.5808/gi.21020 (PMC8261273; doi:10.5808/gi.21020)
Supplement: Supplementary Fig. 4. — Reproducibility analysis of replicates belonging to luminal-A and triple-negative breast cancer cell lines. (A) Replicate 1 peak ranks versus Replicate 2 peak ranks - peaks that do not pass the threshold are colored red. (B) Replicate 1 log10 peak scores versus Replicate 2 log10 peak scores - peaks that do not pass the threshold are colored red. (C, D) Peak rank versus IDR scores are plotted in black. [file gi-21020suppl24.pdf]

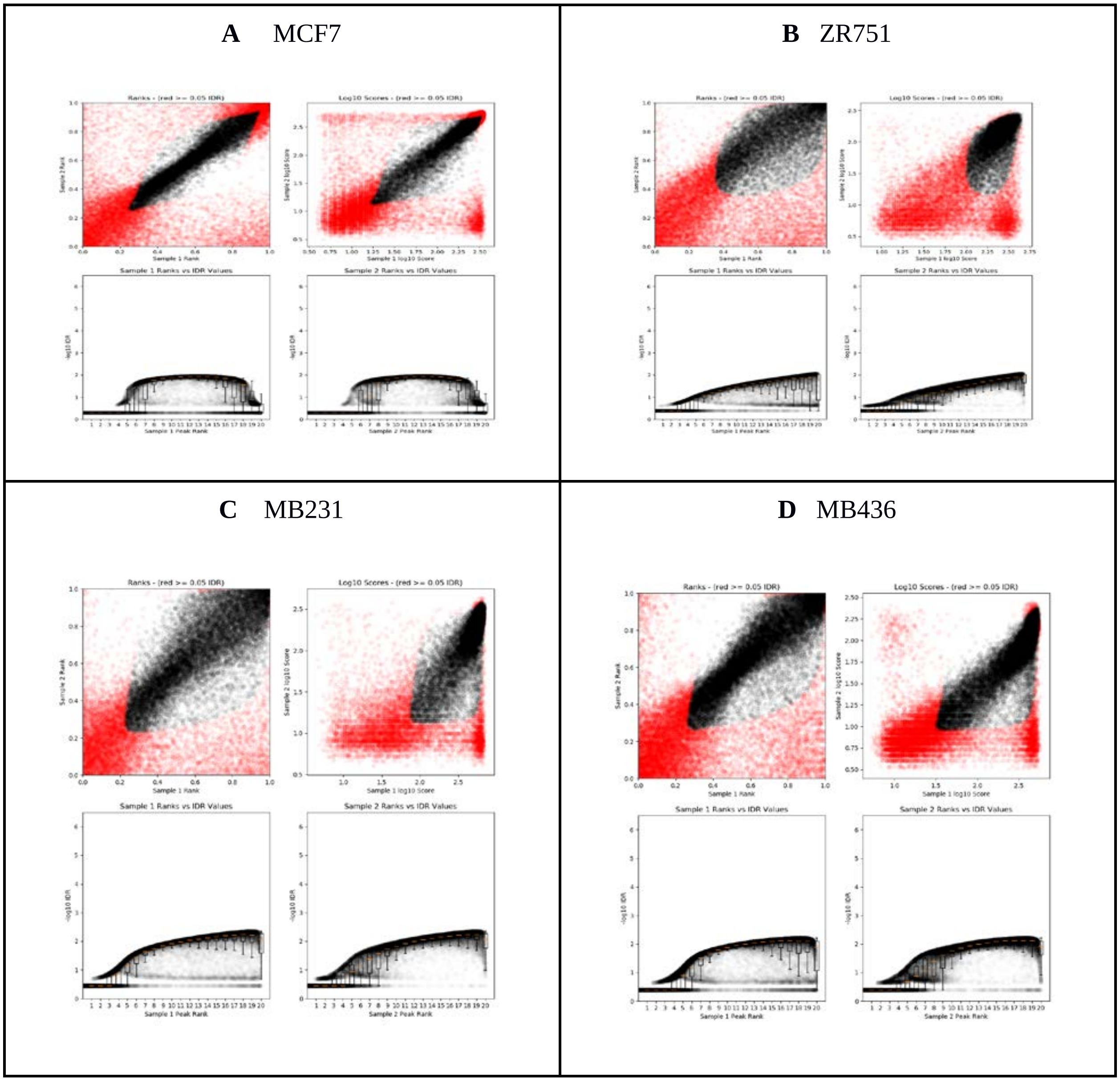

Supplementary Fig. 4. Reproducibility analysis of replicates belonging to luminal-A and triple-negative breast cancer cell lines. (A) Replicate 1 peak ranks versus Replicate 2 peak ranks - peaks that do not pass the threshold are colored red. (B) Replicate 1 log10 peak scores versus Replicate 2 log10 peak scores - peaks that do not pass the threshold are colored red. (C, D) Peak rank versus IDR scores are plotted in black.
