# Supplementary material for: Analysis of H3K4me3-ChIP-Seq and RNA-Seq data to understand the putative role of miRNAs and their target genes in breast cancer cell lines
Source: Genomics Inform. 2021 Jun 30;19(2):e17. doi: 10.5808/gi.21020 (PMC8261273; doi:10.5808/gi.21020)
Supplement: Supplementary Fig. 5. — Annotation of peaks identified for each breast cancer cell line. ChIP, chromatin immunoprecipitation. [file gi-21020suppl25.pdf]

MCF10A
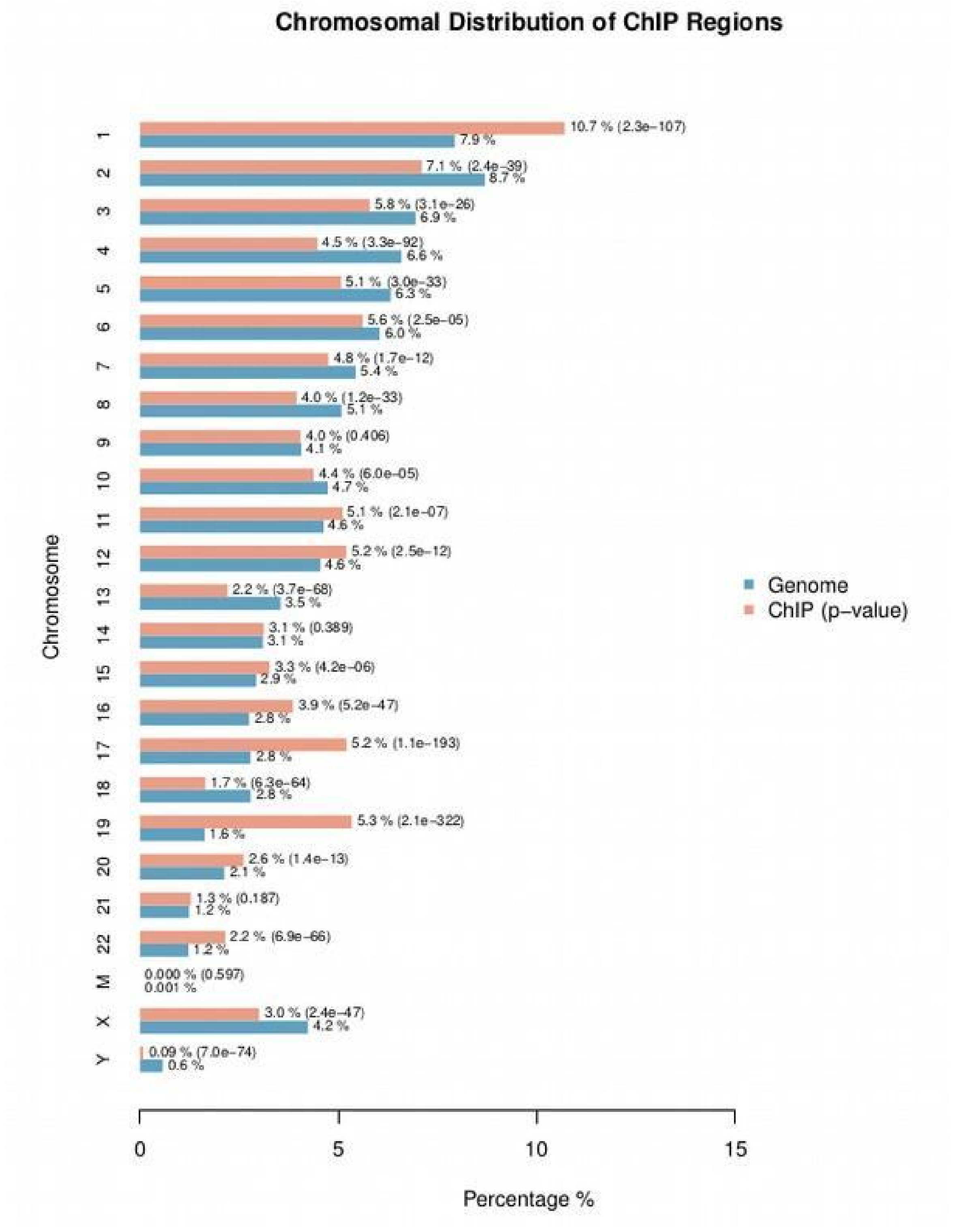Chromosomal Distribution of ChIP Regions
Chromosome
Percentage %
Genome
ChIP (p–value)
0
5
10
15
1
2
3
4
5
6
7
8
9
10
11
12
13
14
15
16
17
18
19
20
21
22
M
X
Y
10.7 % (2.3e–107)
7.9 %
7.1 % (2.4e–39)
8.7 %
5.8 % (3.1e–26)
6.9 %
4.5 % (3.3e–92)
6.6 %
5.1 % (3.0e–33)
6.3 %
5.6 % (2.5e–05)
6.0 %
4.8 % (1.7e–12)
5.4 %
4.0 % (1.2e–33)
5.1 %
4.0 % (0.406)
4.1 %
4.4 % (6.0e–05)
4.7 %
5.1 % (2.1e–07)
4.6 %
5.2 % (2.5e–12)
4.6 %
2.2 % (3.7e–68)
3.5 %
3.1 % (0.389)
3.1 %
3.3 % (4.2e–06)
2.9 %
3.9 % (5.2e–47)
2.8 %
5.2 % (1.1e–193)
2.8 %
1.7 % (6.3e–64)
2.8 %
5.3 % (2.1e–322)
1.6 %
2.6 % (1.4e–13)
2.1 %
1.3 % (0.187)
1.2 %
2.2 % (6.9e–66)
1.2 %
0.000 % (0.597)
0.001 %
3.0 % (2.4e–47)
4.2 %
0.09 % (7.0e–74)
0.6 %
MCF7

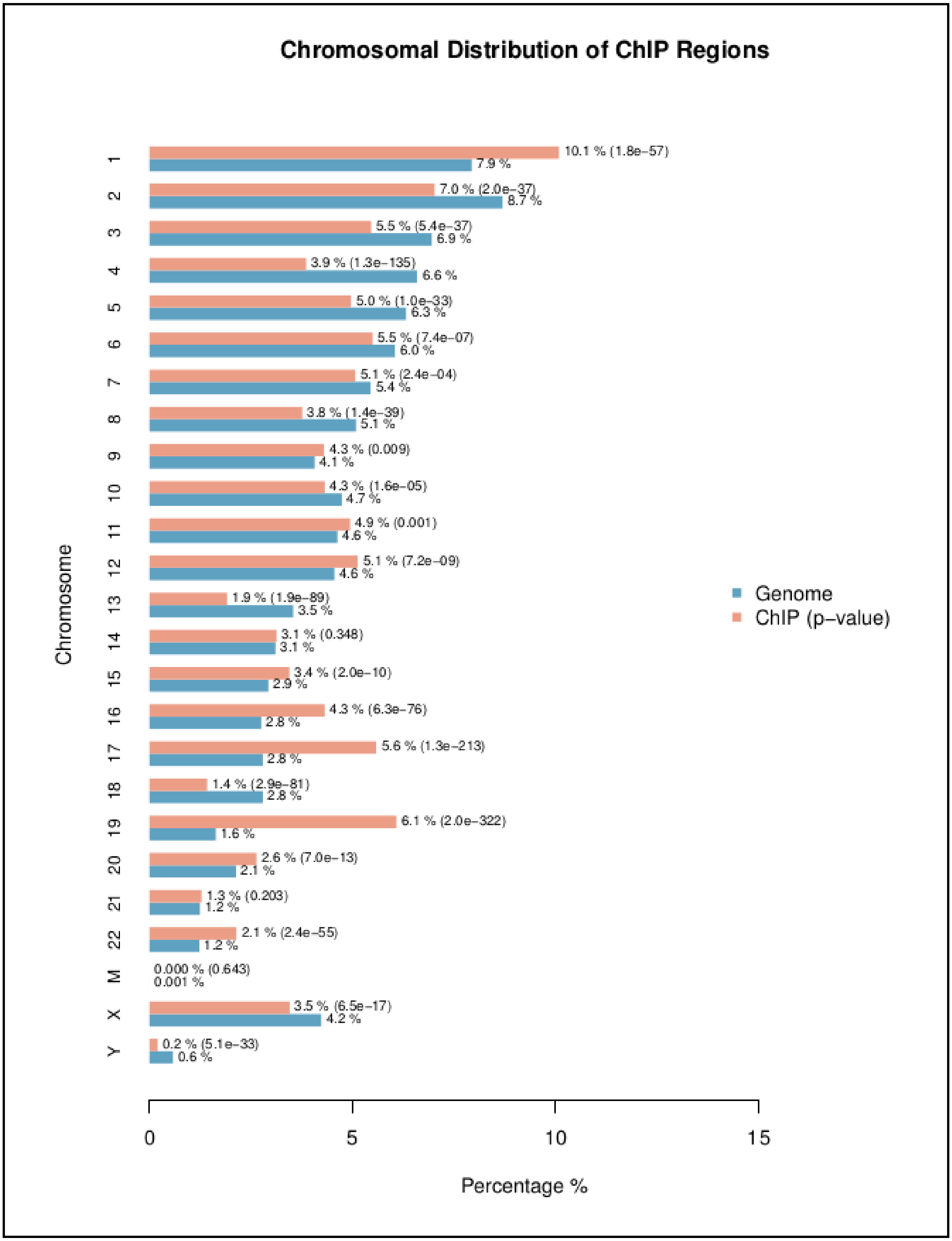

Chromosomal Distribution of ChIP Regions
Chromosome
1
10.1 % (1.8e−57)
7.9 %
2
7.0 % (2.0e−37)
8.7 %
3
5.5 % (5.4e−37)
6.9 %
4
3.9 % (1.3e−135)
6.6 %
5
5.0 % (1.0e−33)
6.3 %
6
5.5 % (7.4e−07)
6.0 %
7
5.1 % (2.4e−04)
5.4 %
8
3.8 % (1.4e−39)
5.1 %
9
4.3 % (0.009)
4.1 %
10
4.3 % (1.6e−05)
4.7 %
11
4.9 % (0.001)
4.6 %
12
5.1 % (7.2e−09)
4.6 %
13
1.9 % (1.9e−89)
3.5 %
14
3.1 % (0.348)
3.1 %
15
3.4 % (2.0e−10)
2.9 %
16
4.3 % (6.3e−76)
2.8 %
17
5.6 % (1.3e−213)
2.8 %
18
1.4 % (2.9e−81)
2.8 %
19
6.1 % (2.0e−322)
1.6 %
20
2.6 % (7.0e−13)
2.1 %
21
1.3 % (0.203)
1.2 %
22
2.1 % (2.4e−55)
1.2 %
M
0.000 % (0.643)
0.001 %
X
3.5 % (6.5e−17)
4.2 %
Y
0.2 % (5.1e−33)
0.6 %
0
5
10
15
Percentage %
Genome
ChIP (p−value)

ZR751

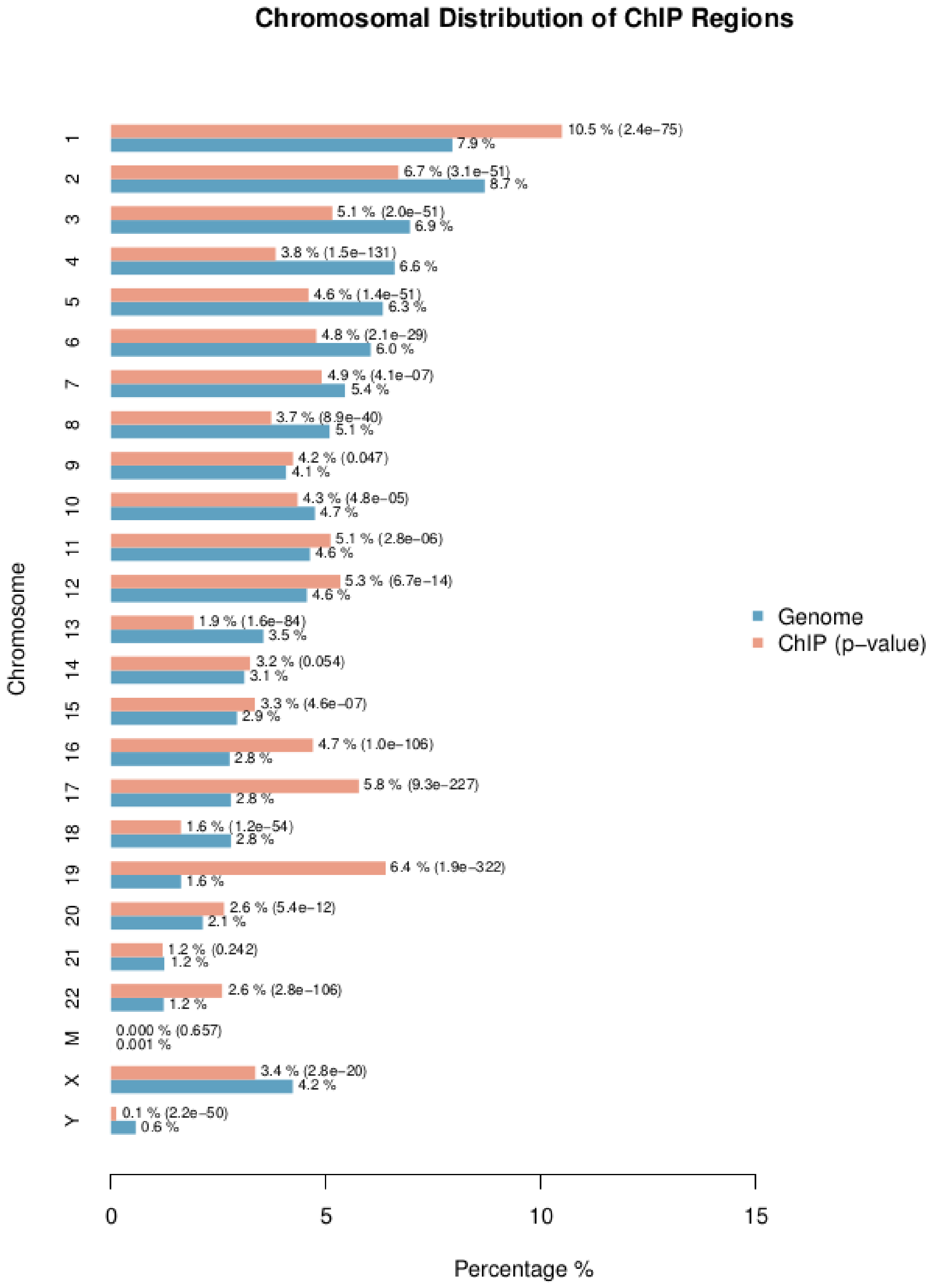
Chromosomal Distribution of ChIP Regions
10.5 % (2.4e−75)
7.9 %
6.7 % (3.1e−51)
8.7 %
5.1 % (2.0e−51)
6.9 %
3.8 % (1.5e−131)
6.6 %
4.6 % (1.4e−51)
6.3 %
4.8 % (2.1e−29)
6.0 %
4.9 % (4.1e−07)
5.4 %
3.7 % (8.9e−40)
5.1 %
4.2 % (0.047)
4.1 %
4.3 % (4.8e−05)
4.7 %
5.1 % (2.8e−06)
4.6 %
5.3 % (6.7e−14)
4.6 %
1.9 % (1.6e−84)
3.5 %
3.2 % (0.054)
3.1 %
3.3 % (4.6e−07)
2.9 %
4.7 % (1.0e−106)
2.8 %
5.8 % (9.3e−227)
2.8 %
1.6 % (1.2e−54)
2.8 %
6.4 % (1.9e−322)
1.6 %
2.6 % (5.4e−12)
2.1 %
1.2 % (0.242)
1.2 %
2.6 % (2.8e−106)
1.2 %
0.000 % (0.657)
0.001 %
3.4 % (2.8e−20)
4.2 %
0.1 % (2.2e−50)
0.6 %
Chromosome
1
2
3
4
5
6
7
8
9
10
11
12
13
14
15
16
17
18
19
20
21
22
M
X
Y
0
5
10
15
Percentage %
Genome
ChIP (p−value)

MB231

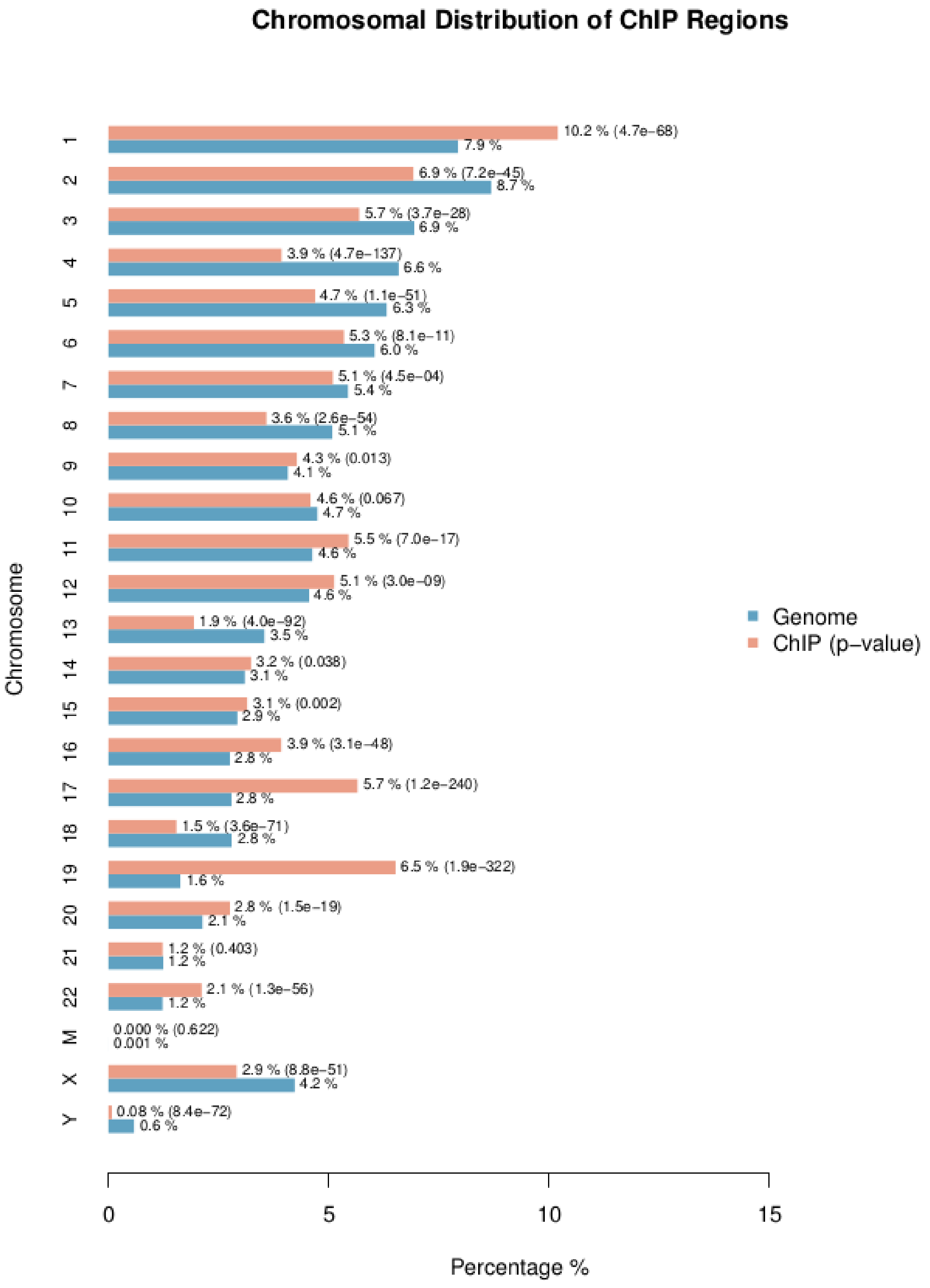
Chromosomal Distribution of ChIP Regions
Chromosome
Percentage %
Genome
ChIP (p–value)
0
5
10
15
1
2
3
4
5
6
7
8
9
10
11
12
13
14
15
16
17
18
19
20
21
22
M
X
Y
10.2 % (4.7e–68)
7.9 %
6.9 % (7.2e–45)
8.7 %
5.7 % (3.7e–28)
6.9 %
3.9 % (4.7e–137)
6.6 %
4.7 % (1.1e–51)
6.3 %
5.3 % (8.1e–11)
6.0 %
5.1 % (4.5e–04)
5.4 %
3.6 % (2.6e–54)
5.1 %
4.3 % (0.013)
4.1 %
4.6 % (0.067)
4.7 %
5.5 % (7.0e–17)
4.6 %
5.1 % (3.0e–09)
4.6 %
1.9 % (4.0e–92)
3.5 %
3.2 % (0.038)
3.1 %
3.1 % (0.002)
2.9 %
3.9 % (3.1e–48)
2.8 %
5.7 % (1.2e–240)
2.8 %
1.5 % (3.6e–71)
2.8 %
6.5 % (1.9e–322)
1.6 %
2.8 % (1.5e–19)
2.1 %
1.2 % (0.403)
1.2 %
2.1 % (1.3e–56)
1.2 %
0.000 % (0.622)
0.001 %
2.9 % (8.8e–51)
4.2 %
0.08 % (8.4e–72)
0.6 %

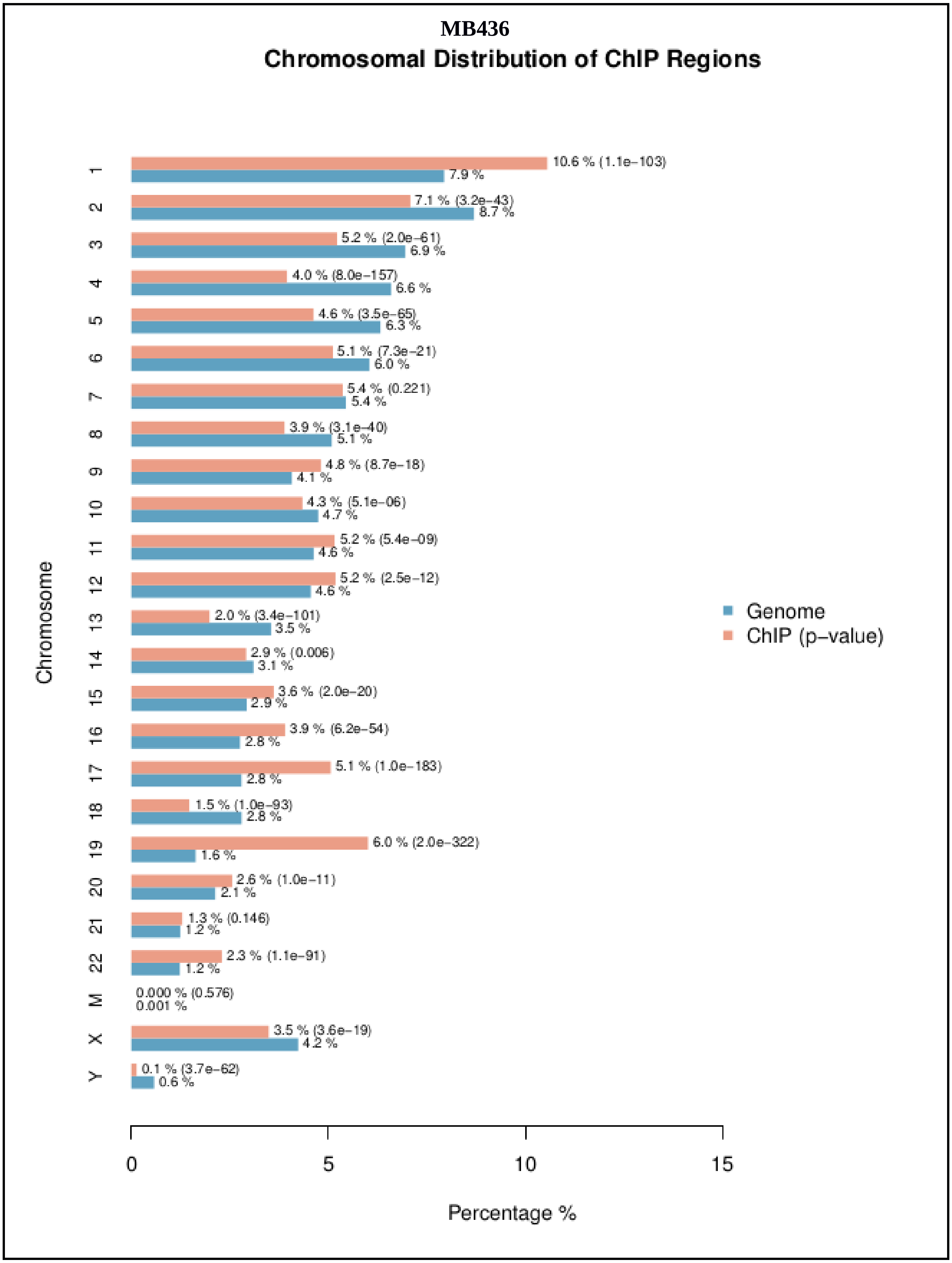

Supplementary Fig. 5. Annotation of peaks identified for each breast cancer cell line. ChIP, chromatin

immunoprecipitation.
