# Supplementary material for: Analysis of H3K4me3-ChIP-Seq and RNA-Seq data to understand the putative role of miRNAs and their target genes in breast cancer cell lines
Source: Genomics Inform. 2021 Jun 30;19(2):e17. doi: 10.5808/gi.21020 (PMC8261273; doi:10.5808/gi.21020)
Supplement: Supplementary Fig. 6. — Relative gene expression of triple-negative breast cancer subtype exclusive miRNAs from The Cancer Genome Atlas (TCGA) data samples. (A) miR-153-1. (B) miR-6720. (C) miR-Let7i. [file gi-21020suppl26.pdf]

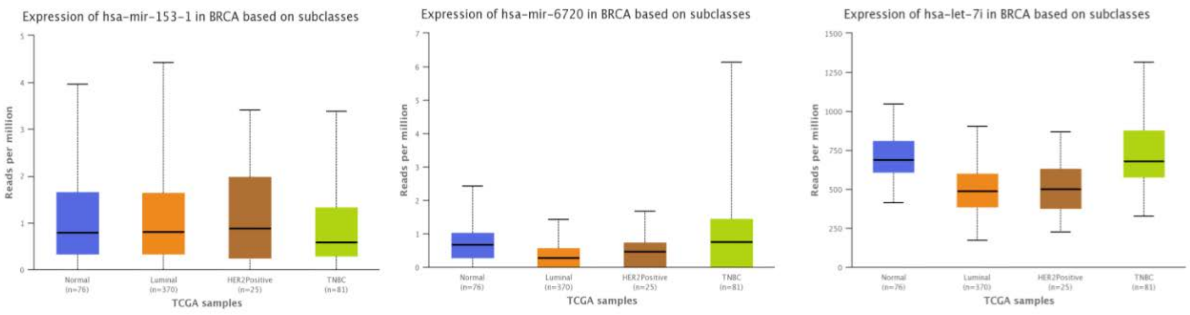

A B C

Supplementary Fig. 6. Relative gene expression of triple-negative breast cancer subtype exclusive miRNAs from The Cancer Genome Atlas (TCGA) data samples. (A) miR-153-1). (B) miR-6720. (C) miR-Let7i.
