# Supplementary material for: Analysis of H3K4me3-ChIP-Seq and RNA-Seq data to understand the putative role of miRNAs and their target genes in breast cancer cell lines
Source: Genomics Inform. 2021 Jun 30;19(2):e17. doi: 10.5808/gi.21020 (PMC8261273; doi:10.5808/gi.21020)
Supplement: Supplementary Fig. 8. — FastQC output of RNA-sequencing (RNA-Seq) data pertaining to each cell-line and their corresponding biological replicates. [file gi-21020suppl28.pdf]

MCF10A-RNA-Seq-Rep1

Per base sequence quality

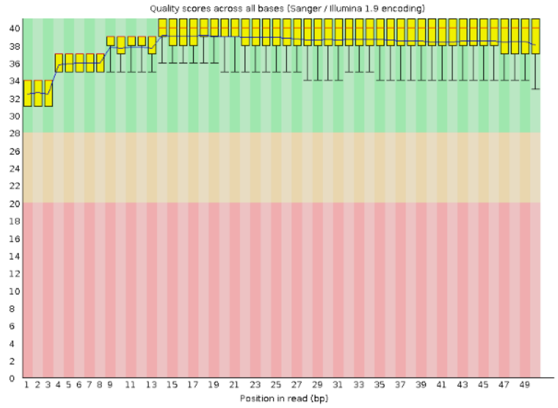

MCF10A-RNA-Seq-Rep2

Per base sequence quality

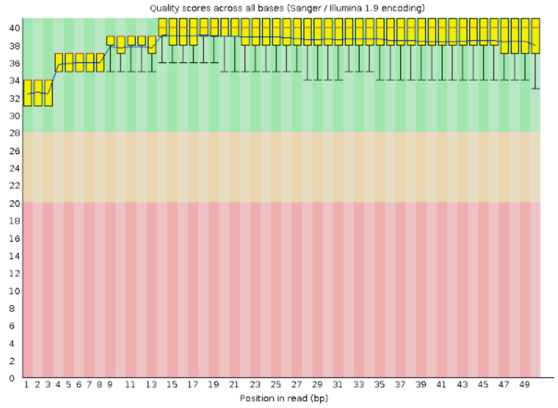

MCF10A-RNA-Seq-Rep3

Per base sequence quality

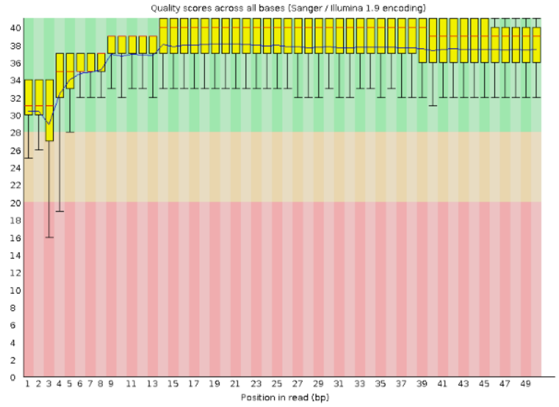

MCF10A-RNA-Seq-Rep4

Per base sequence quality

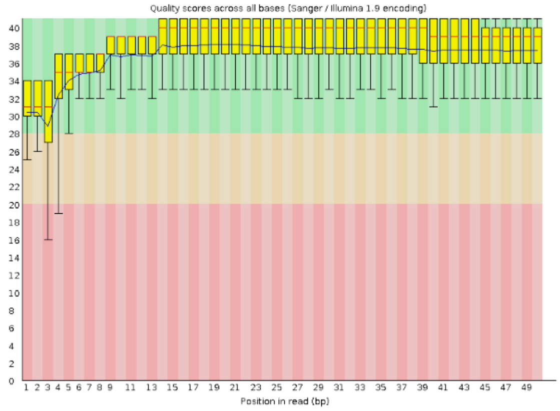

MCF7-RNA-Seq-Rep1

Per base sequence quality

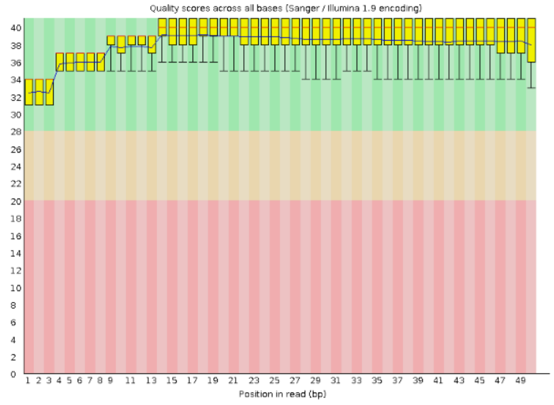

MCF7-RNA-Seq-Rep2

Per base sequence quality

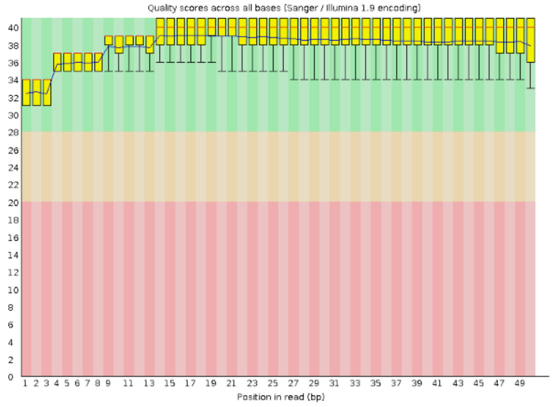

MCF7-RNA-Seq-Rep3

Per base sequence quality

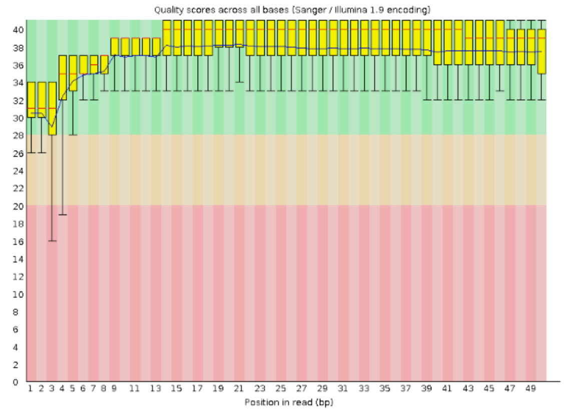

MCF7-RNA-Seq-Rep4

Per base sequence quality

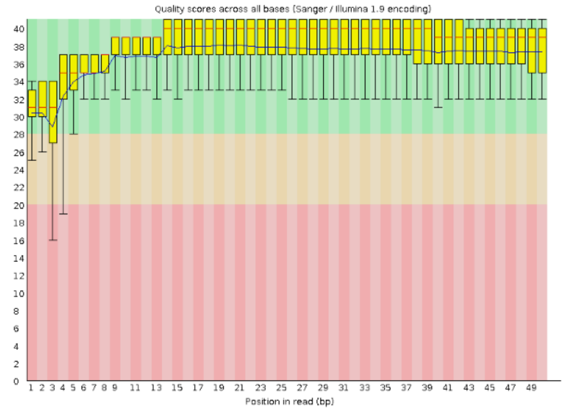

ZR751-RNA-Seq-Rep1

Per base sequence quality

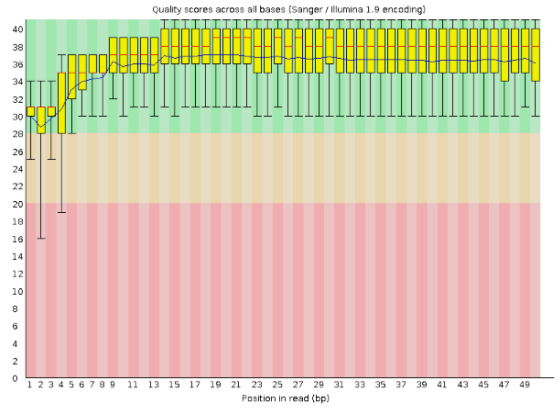

ZR751-RNA-Seq-Rep2

Per base sequence quality

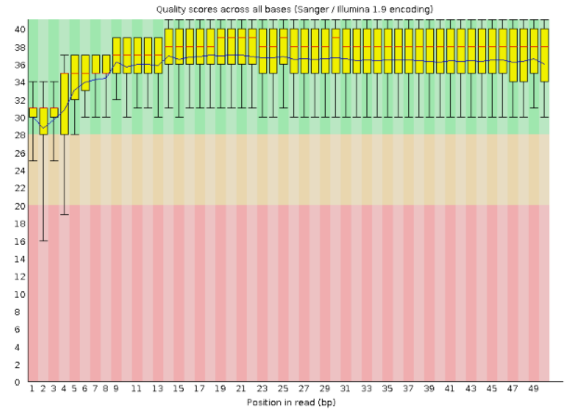

ZR751-RNA-Seq-Rep3

Per base sequence quality

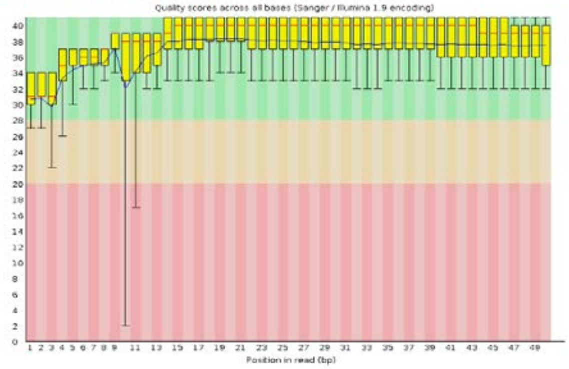

ZR751-RNA-Seq-Rep4

Per base sequence quality

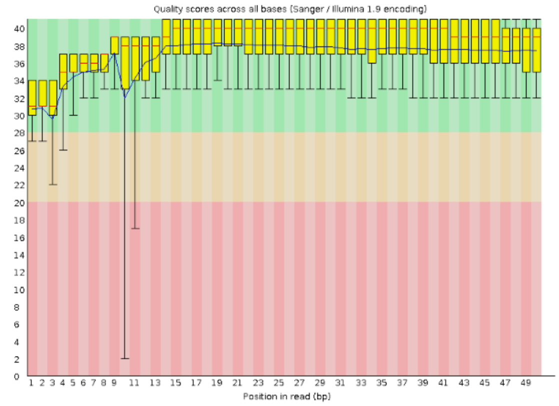

MB231-RNA-Seq-Rep1	MB231-RNA-Seq-Rep2
Per base sequence quality 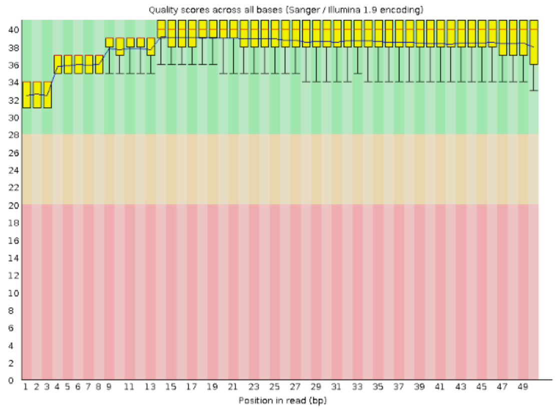	Per base sequence quality 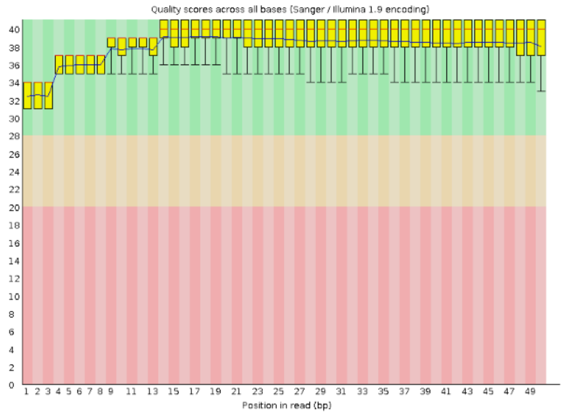
**MB231-RNA-Seq-Rep3**	**MB231-RNA-Seq-Rep4**
Per base sequence quality 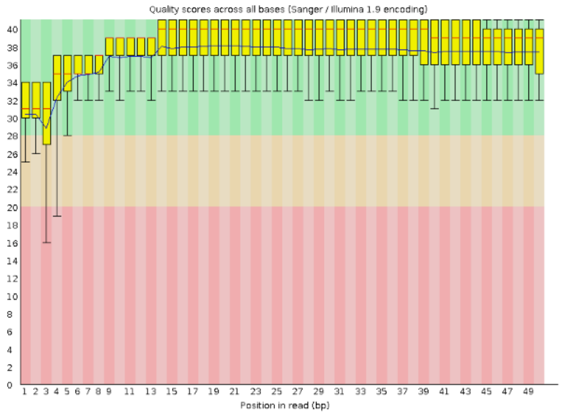	Per base sequence quality 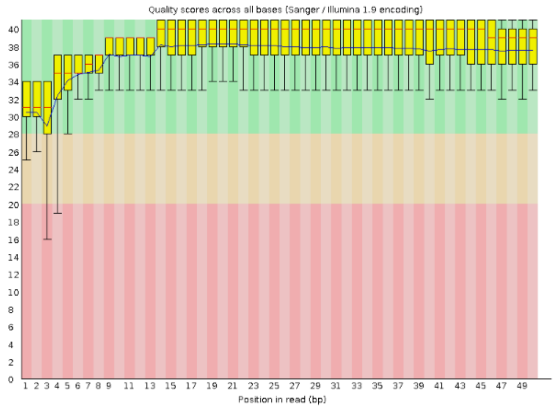
**MB436-RNA-Seq-Rep1**	**MB436-RNA-Seq-Rep2**
Per base sequence quality 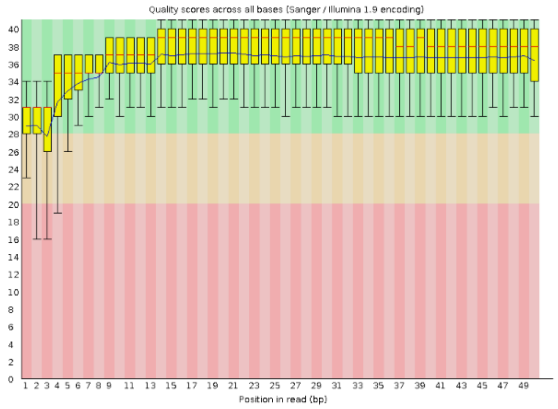	Per base sequence quality 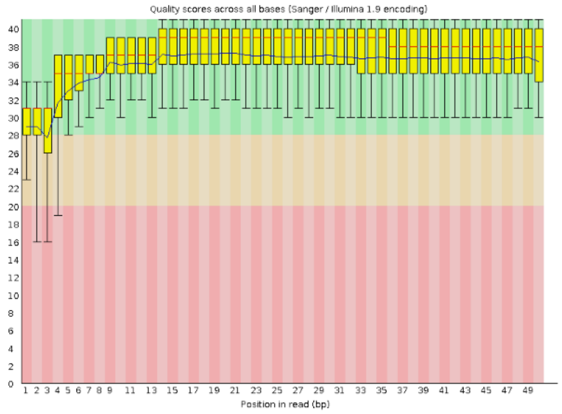
**MB436-RNA-Seq-Rep3**	**MB436-RNA-Seq-Rep4**

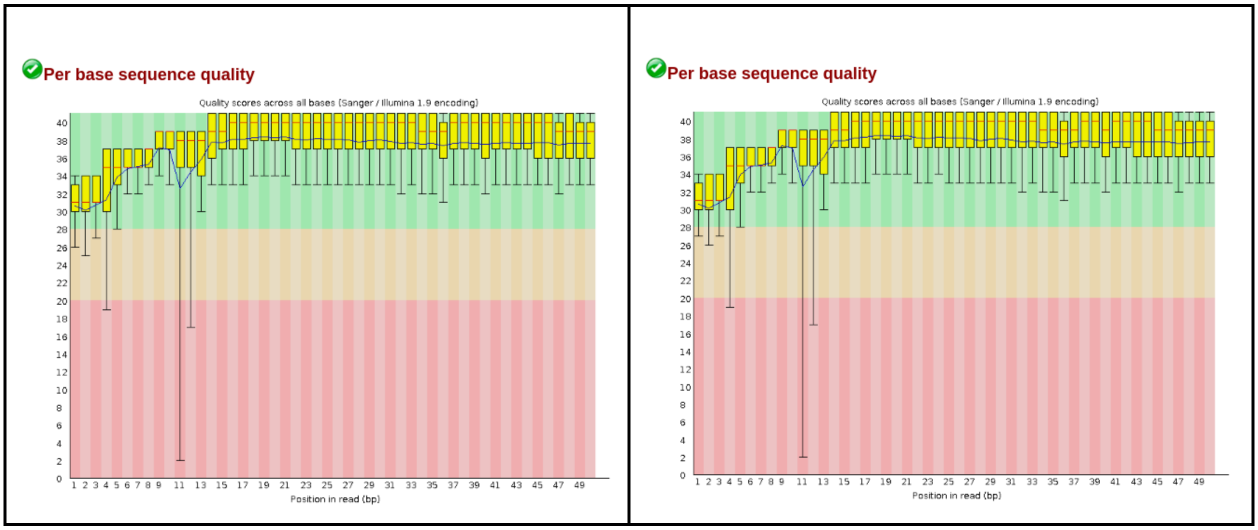

Supplementary Fig. 8. FastQC output of RNA-sequencing (RNA-Seq) data pertaining to each cell-line and their corresponding biological replicates.
