# Supplementary figures and images for: Analysis of H3K4me3-ChIP-Seq and RNA-Seq data to understand the putative role of miRNAs and their target genes in breast cancer cell lines
Source: Genomics Inform. 2021 Jun 30;19(2):e17. doi: 10.5808/gi.21020 (PMC8261273; doi:10.5808/gi.21020)

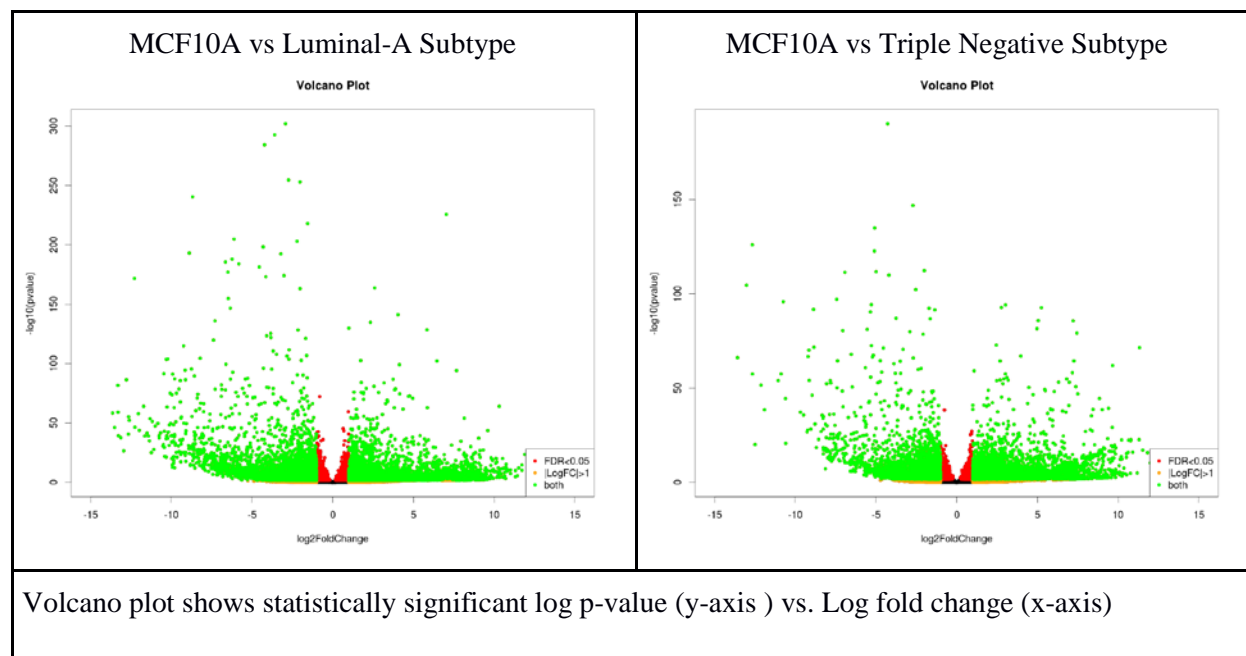

**Supplementary Fig. 9.** Differential expression analysis of RNA sequencing data: Volcano plots.

Supplement: Supplementary Fig. 9. — Differential expression analysis of RNA sequencing data: Volcano plots. [file gi-21020suppl29.pdf]
