# Supplementary material for: Analysis of H3K4me3-ChIP-Seq and RNA-Seq data to understand the putative role of miRNAs and their target genes in breast cancer cell lines
Source: Genomics Inform. 2021 Jun 30;19(2):e17. doi: 10.5808/gi.21020 (PMC8261273; doi:10.5808/gi.21020)
Supplement: Supplementary Fig. 10. — Relative gene expression of triple-negative breast cancer subtype exclusive miRNA targets from the Broad Institute Cancer Cell Line Encyclopedia (CCLE) database. [file gi-21020suppl30.pdf]

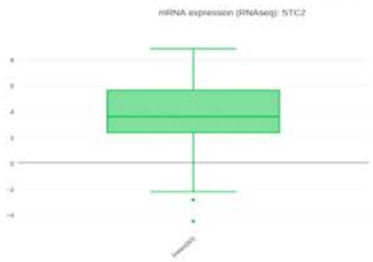

A

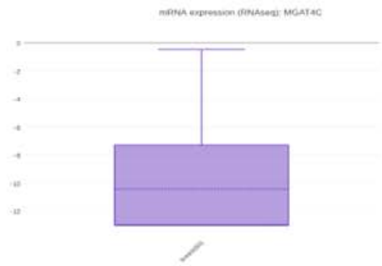

B

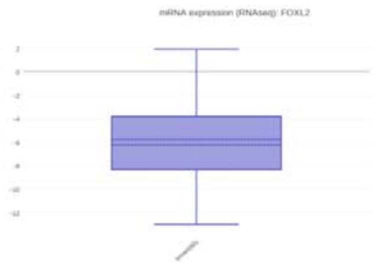

C

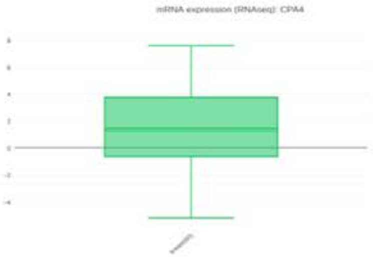

D

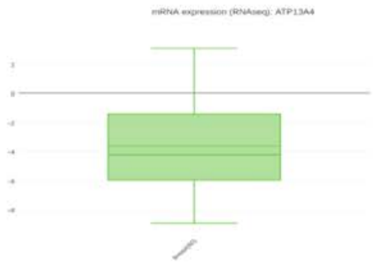

E

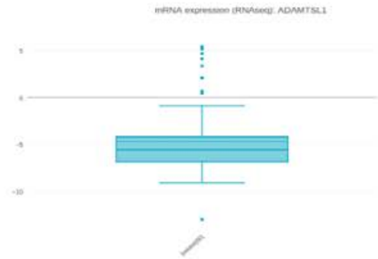

F

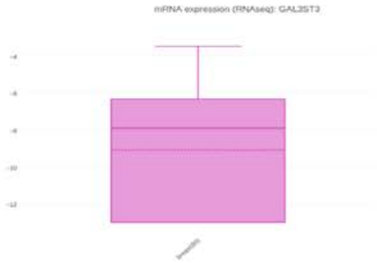

G

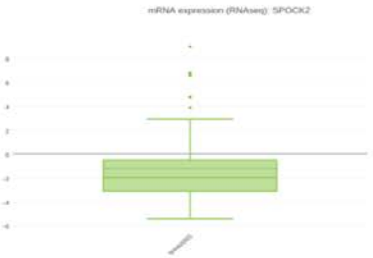

H

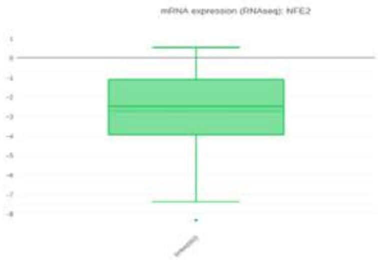

I

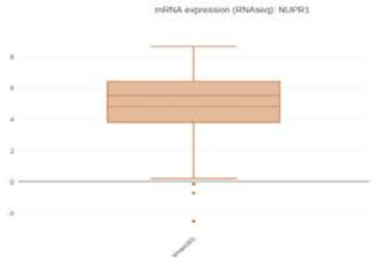

J

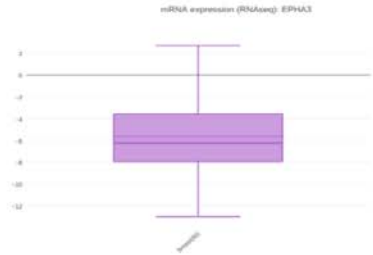

K

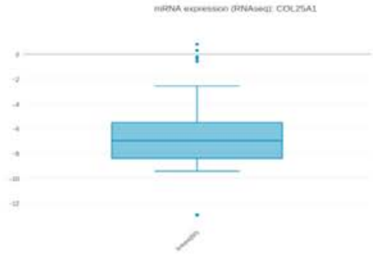

L

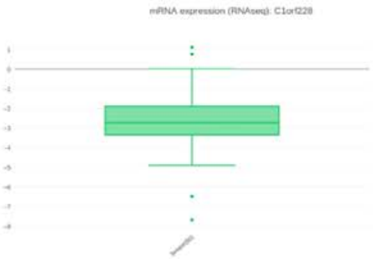

M

Supplementary Fig. 10. Relative gene expression of triple-negative breast cancer subtype exclusive miRNA targets from the Broad Institute Cancer Cell Line Encyclopedia (CCLE) database.
