# Supplementary material for: Analysis of H3K4me3-ChIP-Seq and RNA-Seq data to understand the putative role of miRNAs and their target genes in breast cancer cell lines
Source: Genomics Inform. 2021 Jun 30;19(2):e17. doi: 10.5808/gi.21020 (PMC8261273; doi:10.5808/gi.21020)
Supplement: Supplementary Fig. 11. — Relative gene expression of triple-negative breast cancer subtype exclusive miRNA targets from The Cancer Genome Atlas (TCGA) data samples. [file gi-21020suppl31.pdf]

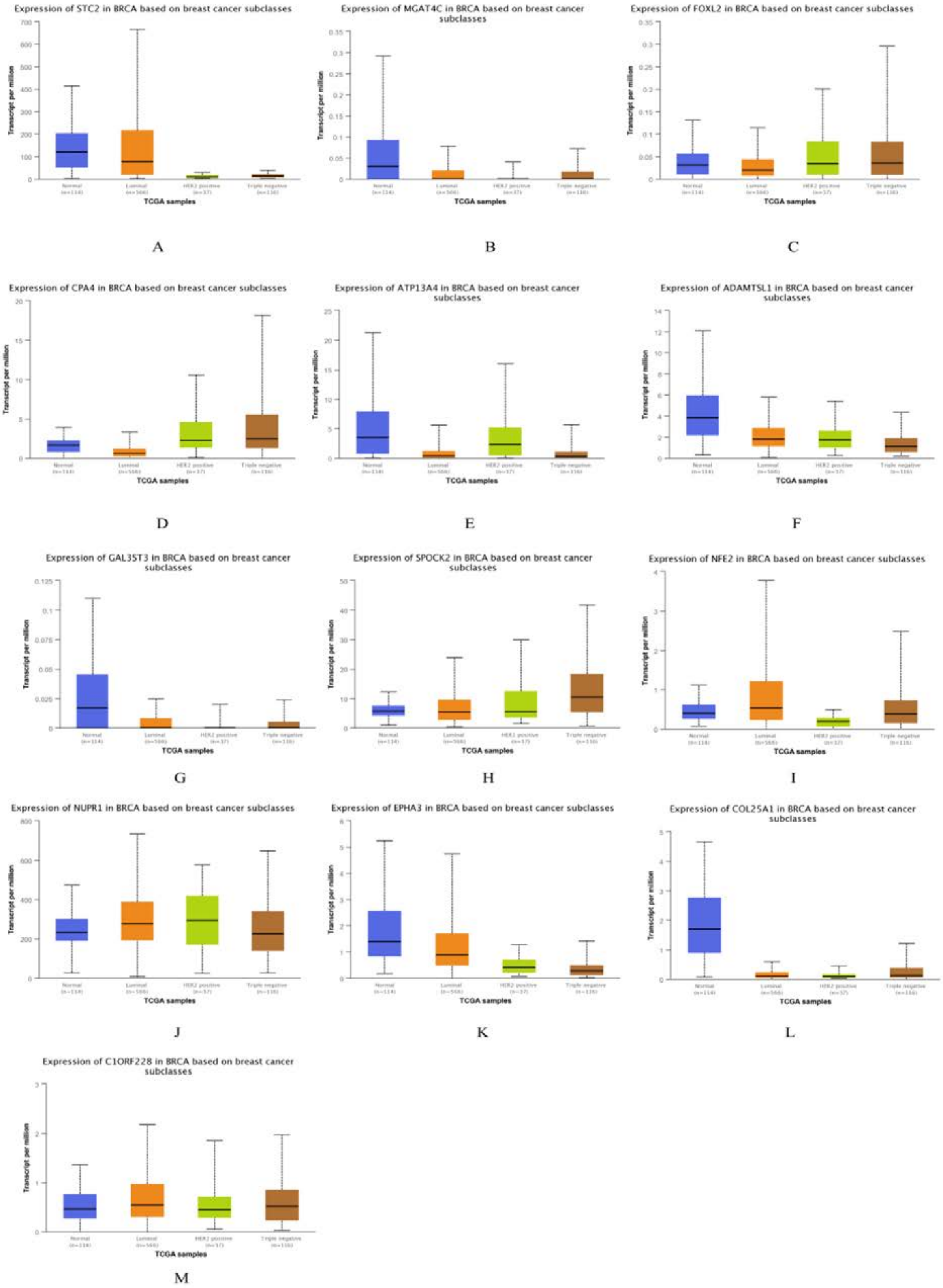

Supplementary Fig. 11. Relative gene expression of triple-negative breast cancer subtype

exclusive miRNA targets from The Cancer Genome Atlas (TCGA) data samples.
