# Supplementary material for: Analysis of H3K4me3-ChIP-Seq and RNA-Seq data to understand the putative role of miRNAs and their target genes in breast cancer cell lines
Source: Genomics Inform. 2021 Jun 30;19(2):e17. doi: 10.5808/gi.21020 (PMC8261273; doi:10.5808/gi.21020)
Supplement: Supplementary Fig. 12. — Five-year KM-survival plots from human protein atlas web server: triple-negative breast cancer subtype exclusive miRNA target genes. [file gi-21020suppl32.pdf]

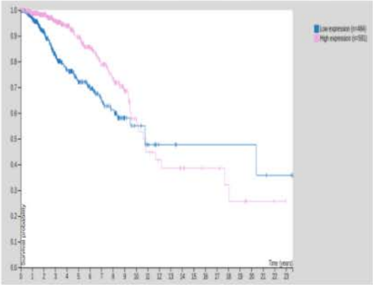

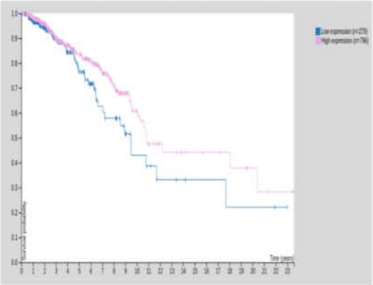

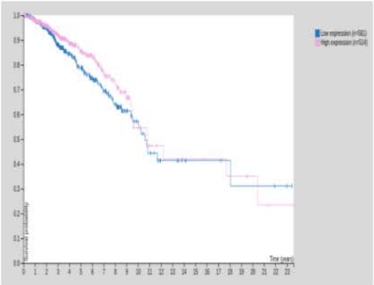

A (STC2)

B(MGAT4C)

C(FOXL2)

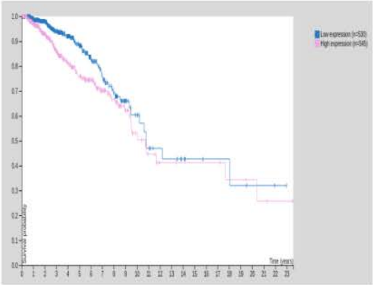

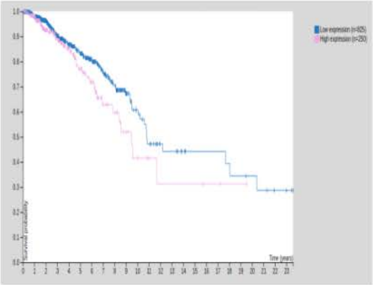

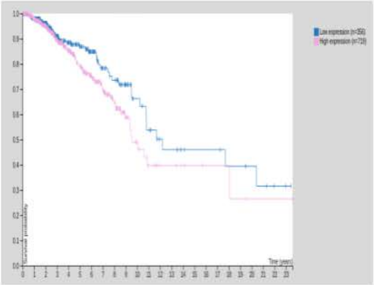

D (CPA4)

E (ATP13A4)

F (ADAMTSL1)

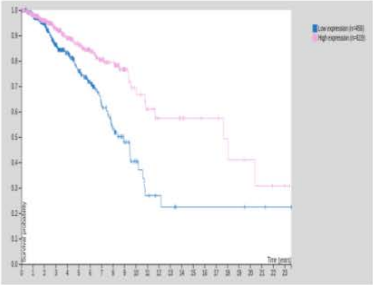

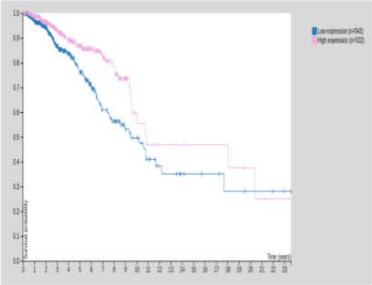

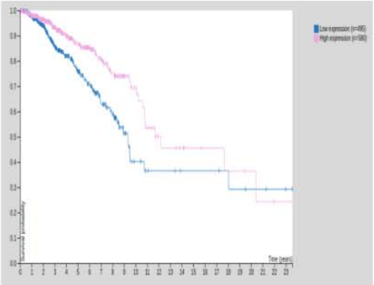

G (C1orf228)

H (SPOCK2)

I (NFE2)

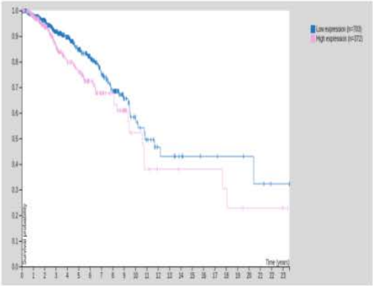

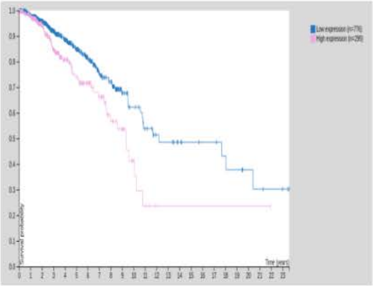

No KM Survival Plot Available In Human Protein Atlas for Breast Cancer

J (NUPR1)

K (EPHA3)

L (COL25A1)

No KM Survival Plot Available In Human Protein Atlas for Breast Cancer

M (GAL3ST3)

Supplementary Fig. 12. Five-year KM-survival plots from human protein atlas web server: triple-negative breast cancer subtype exclusive miRNA target genes.
