# Supplementary material for: Analysis of H3K4me3-ChIP-Seq and RNA-Seq data to understand the putative role of miRNAs and their target genes in breast cancer cell lines
Source: Genomics Inform. 2021 Jun 30;19(2):e17. doi: 10.5808/gi.21020 (PMC8261273; doi:10.5808/gi.21020)
Supplement: Supplementary Fig. 13. — Relative gene expression of (The Cancer Genome Atlas [TCGA] breast cancer samples) luminal-A downregulated gene targets (5 of the total 17 genes) previously reported in breast cancer that do not correlate with predicted miRNA binding analysis. [file gi-21020suppl33.pdf]

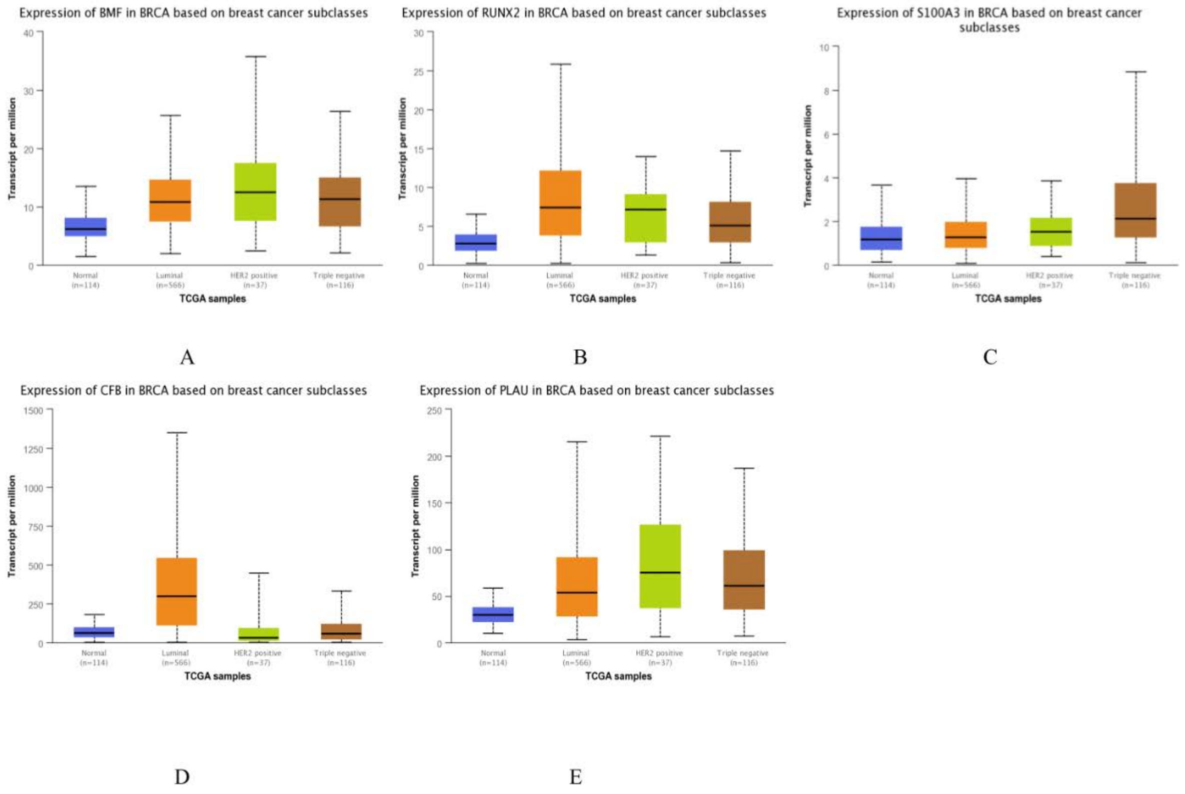

Supplementary Fig. 13. Relative gene expression of (The Cancer Genome Atlas [TCGA] breast cancer samples) luminal-A downregulated gene targets (5 of the total 17 genes) previously reported in breast cancer that do not correlate with predicted miRNA binding analysis.
