# Supplementary material for: Analysis of H3K4me3-ChIP-Seq and RNA-Seq data to understand the putative role of miRNAs and their target genes in breast cancer cell lines
Source: Genomics Inform. 2021 Jun 30;19(2):e17. doi: 10.5808/gi.21020 (PMC8261273; doi:10.5808/gi.21020)
Supplement: Supplementary Fig. 15. — Relative gene expression of triple-negative breast cancer and luminal-A specific miRNAs gene targets from Broad Institute Cancer Cell Line Encyclopedia (CCLE) database (ACTB gene expression as control). [file gi-21020suppl35.pdf]

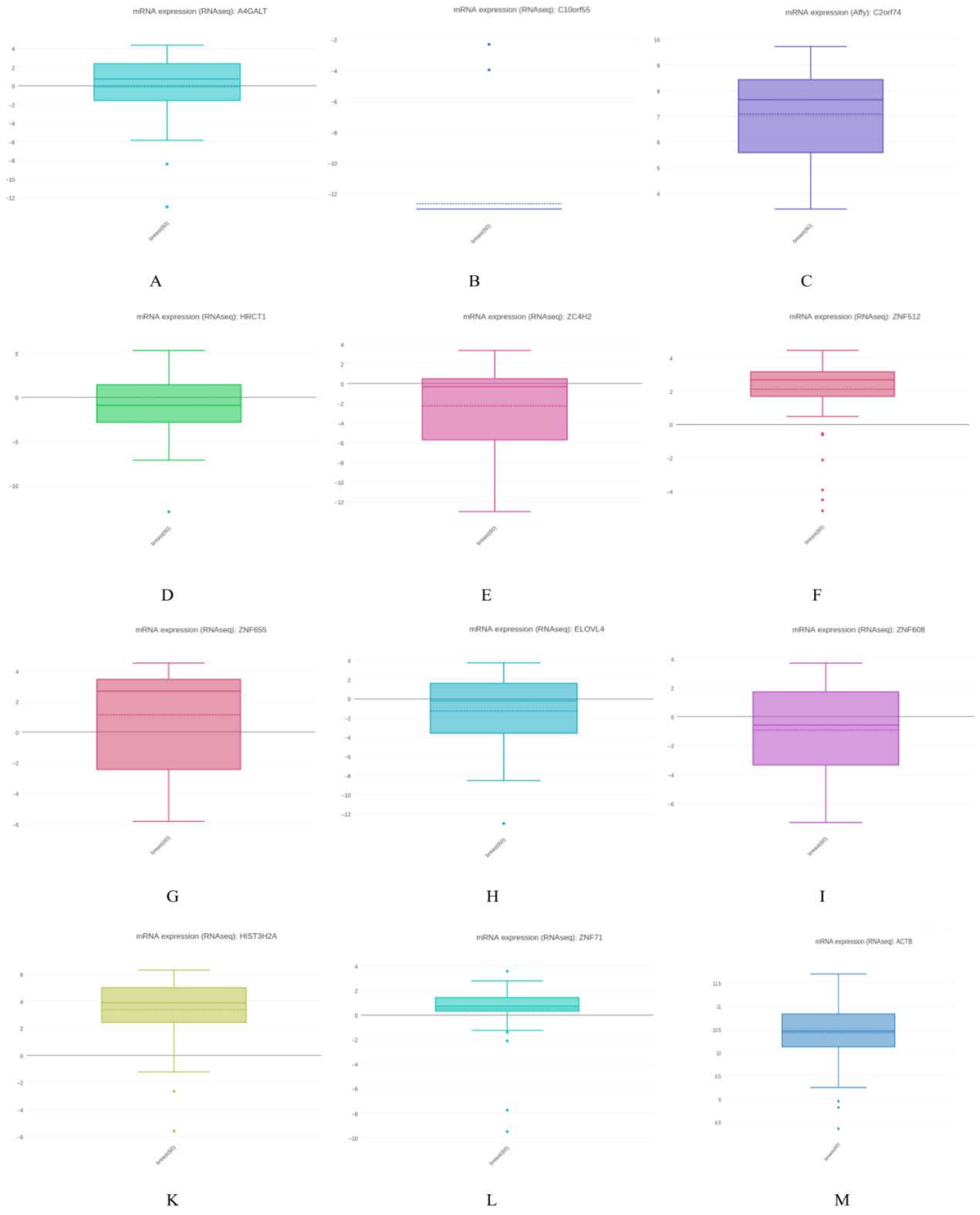

Supplementary Fig. 15. Relative gene expression of triple-negative breast cancer and luminal-A specific miRNAs gene targets from Broad Institute Cancer Cell Line Encyclopedia (CCLE) database (*ACTB* gene expression as control).
