# Supplementary material for: Analysis of H3K4me3-ChIP-Seq and RNA-Seq data to understand the putative role of miRNAs and their target genes in breast cancer cell lines
Source: Genomics Inform. 2021 Jun 30;19(2):e17. doi: 10.5808/gi.21020 (PMC8261273; doi:10.5808/gi.21020)
Supplement: Supplementary Fig. 18. — Relative gene expression of triple-negative breast cancer and luminal-A specific miRNAs gene targets in The Cancer Genome Atlas (TCGA) samples:across cancer subtypes. [file gi-21020suppl38.pdf]

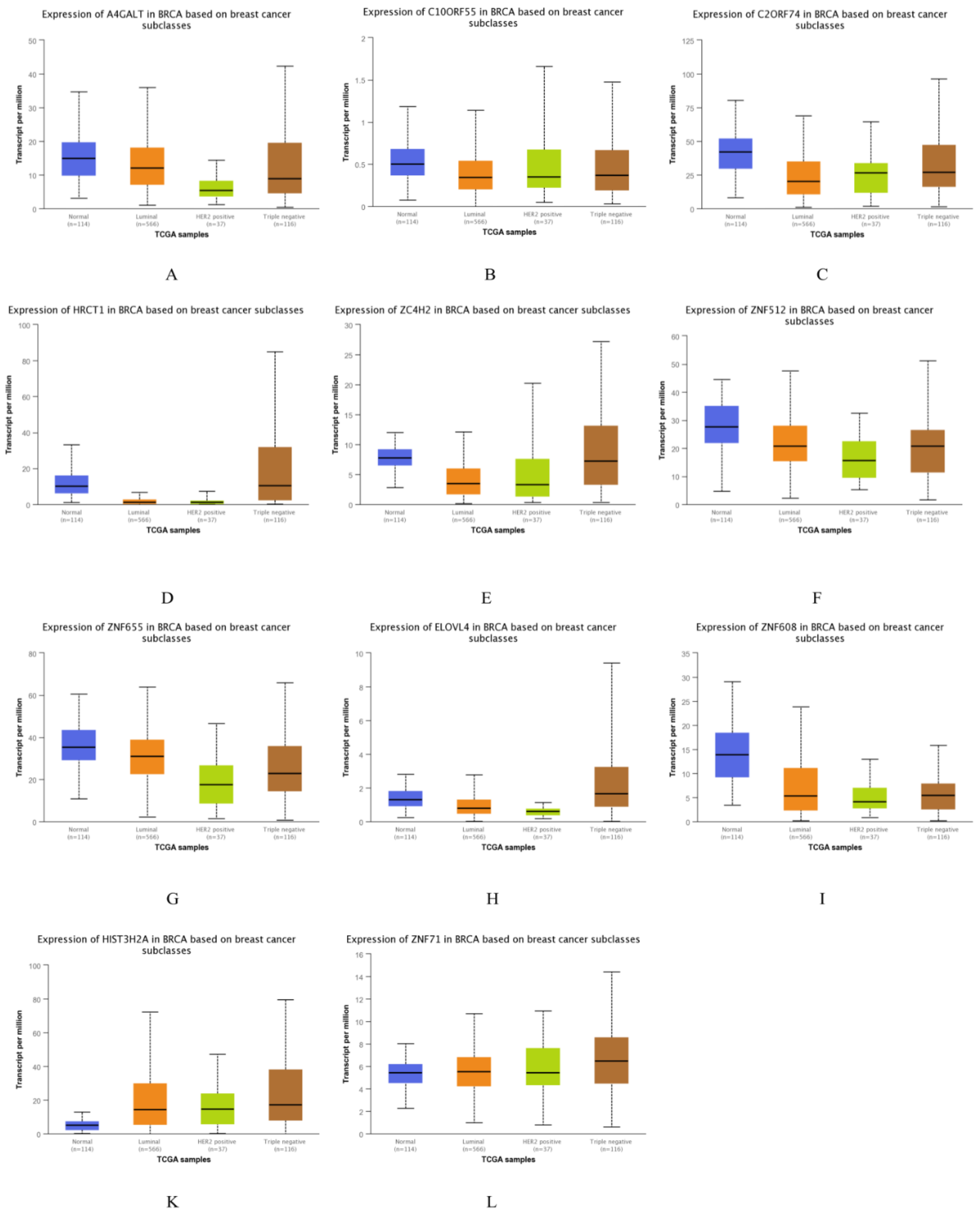

Supplementary Fig. 18. Relative gene expression of triple-negative breast cancer and luminal-A specific miRNAs gene targets in The Cancer Genome Atlas (TCGA) samples:across cancer subtypes.
