# Supplementary material for: Analysis of H3K4me3-ChIP-Seq and RNA-Seq data to understand the putative role of miRNAs and their target genes in breast cancer cell lines
Source: Genomics Inform. 2021 Jun 30;19(2):e17. doi: 10.5808/gi.21020 (PMC8261273; doi:10.5808/gi.21020)
Supplement: Supplementary Fig. 19. — Five-year KM-survival plots from Human Protein Atlas: triple-negative breast cancer and luminal-A specific miRNAs gene targets. [file gi-21020suppl39.pdf]

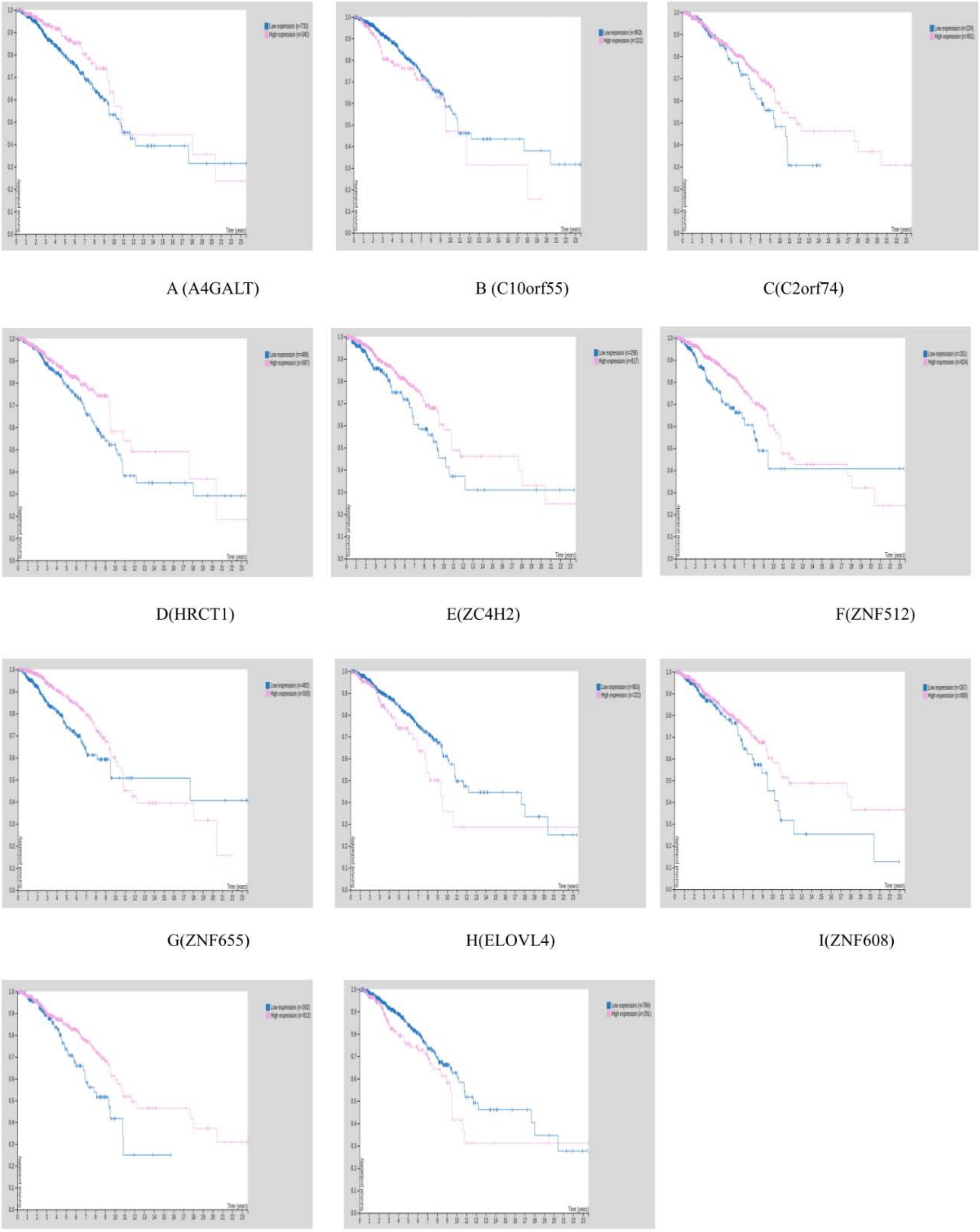

Supplementary Fig. 19. Five-year KM-survival plots from Human Protein Atlas: triple-negative breast cancer and luminal-A specific miRNAs gene targets.
